# Rearing of *Glossina morsitans morsitans* tsetse flies for the sterile insect technique: evaluating the impact of irradiation and transportation during early and late-stage pupal development on the quality of emerging adults[Fn FN2]

**DOI:** 10.1051/parasite/2024068

**Published:** 2024-11-21

**Authors:** Caroline K. Mirieri, Güler Demirbas Uzel, Andrew G. Parker, Jérémy Bouyer, Linda De Vooght, Vera I.D. Ros, Monique M. van Oers, Adly M.M. Abd-Alla

**Affiliations:** 1 Insect Pest Control Laboratory, Joint FAO, IAEA Division of Nuclear Techniques in Food & Agriculture, Vienna International Centre P.O. Box 100 1400 Vienna Austria; 2 Laboratory of Virology, Wageningen University & Research Droevendaalsesteeg 1 6708 PB Wageningen the Netherlands; 3 ASTRE, CIRAD, INRAE, Plate Forme CYROI 2 rue Maxime Rivière 97491 Sainte-Clotilde La Réunion France; 4 Department of Biomedical Sciences, Unit of Veterinary Protozoology, Institute of Tropical Medicine Antwerp (ITM) Antwerp Belgium

**Keywords:** Trypanosomiasis, Irradiation and transportation, Pupal development, Vibration

## Abstract

Human African trypanosomiasis (HAT) and African animal trypanosomosis (AAT) are devastating diseases spread by tsetse flies (*Glossina* spp.), affecting humans and livestock, respectively. Current efforts to manage these diseases by eliminating the vector through the sterile insect technique (SIT) require transportation of irradiated late-stage tsetse pupae under chilling, which has been reported to reduce the biological quality of emerged flies. We therefore evaluated the impact of irradiation and transportation (including vibration and shock) on pupae at early-stage development (22 days of age) under ambient temperature and compared it to that on pupae at the late-stage development (29 days of age) under chilling, the current practice for tsetse in SIT programs. The quality of flies emerging from these transported pupae was assessed by their emergence rates, flight propensity, mating ability, insemination rates and survival rates (over ca. 100 days, and after specified shorter periods). Generally, flies emerging from the 22-day-old pupae had significantly (*p *< 0.05) higher values for the tested quality parameters, as compared to those emerging from 29-day-old pupae. Irradiation, transportation and the combination thereof significantly (*p *< 0.05) reduced all the tested quality parameters as compared with the untreated control within the 22-day-old pupae group. Further, vibration had a significant negative effect on the quality of flies, notwithstanding the age of the pupae. Irradiation and transportation of pupae at 22 days of age resulted in a higher proportion of flies of good biological quality as compared to those of 29 days of age, and hence may be considered for future SIT programs.

## Abstract (fr)

La trypanosomiase humaine africaine (THA) et la trypanosomose animale africaine (TAA) sont des maladies dévastatrices propagées par les mouches tsé-tsé (*Glossina* spp.), qui affectent respectivement les humains et le bétail. Les efforts actuels pour gérer ces maladies en éliminant le vecteur grâce à la technique de l'insecte stérile (TIS) nécessitent le transport de pupes de tsé-tsé irradiées au stade avancé sous réfrigération, ce qui réduirait la qualité biologique des mouches émergées. Nous avons donc évalué l'impact de l'irradiation et du transport (y compris les vibrations et les chocs) des pupes au stade précoce de développement (22 jours d'âge) à température ambiante et l'avons comparé à celui des pupes au stade avancé de développement (29 jours d'âge) sous réfrigération, la pratique actuelle pour les mouches tsé-tsé dans les programmes de TIS. La qualité des mouches émergeant de ces pupes transportées a été évaluée par leurs taux d'émergence, leur propension à voler, leur capacité d'accouplement, leur taux d'insémination et leurs taux de survie (sur environ 100 jours et après des périodes plus courtes spécifiées). En général, les mouches sortant des pupes de 22 jours présentaient des valeurs significativement plus élevées (*P *< 0,05) pour les paramètres de qualité testés, par rapport à celles sortant des pupes de 29 jours. L'irradiation, le transport et leur combinaison ont réduit de manière significative (*P *< 0,05) tous les paramètres de qualité testés par rapport au témoin non traité dans le groupe des pupes de 22 jours. De plus, les vibrations ont eu un effet négatif significatif sur la qualité des mouches, quel que soit l'âge des pupes. L'irradiation et le transport des pupes à 22 jours ont donné lieu à une proportion plus élevée de mouches de bonne qualité biologique par rapport à celles de 29 jours, et peuvent donc être envisagées pour les futurs programmes TIS.

## Introduction

Human African trypanosomiasis (HAT) and African animal trypanosomiasis (AAT) are infectious diseases caused by parasites (*Trypanosoma* spp.) and are spread by several species of tsetse flies, which are present in 38 countries in Sub-Saharan Africa [3, 16, 22, 39, 50, 56, 66, 67, 76]. HAT results in morbidity and mortality in humans, while AAT results in food insecurity and economic losses, a consequence of decreased productivity of milk and draught power, death of livestock and reduced arable land cultivation [1, 14, 33]. In the absence of effective vaccines and the development of drug resistance by trypanosomes, concerted efforts have been directed towards suppression and eradication of the tsetse vectors, using integrated pest management tools [4, 69, 73–75]. These include the sterile insect technique (SIT), which successfully eradicated tsetse flies from Unguja Island, Zanzibar in 1997 [35, 74]. This success has inspired similar attempts to eradicate tsetse flies from various target areas in other parts of Africa, including Ethiopia and Senegal [3, 67]. The optimal use of SIT necessitates continuous innovations of the associated processes and techniques in mass-rearing factories to improve the quality of flies and hence increase the effectiveness of SIT in tsetse control. Due to the expenses related with running a mass-rearing factory for tsetse flies, the sterile males may be produced in mass-rearing facilities located far from the targeted area of release and, therefore, they are irradiated and transported as pupae, to a facility where the adults emerge, located near the targeted release area [30, 52]. Hence, pupae are usually held under low temperatures (10 °C) in order to irradiate a batch of pupae collected over several days at the same time, which are thereafter also transported under the same chilled conditions, to the field. Such procedures are followed by various tsetse mass-rearing facilities including the Slovak Academy of Science (SAS) and the Insectary of Bobo Dioulasso (IBD), that provide and supplement the tsetse sterile males needed for the SIT program in Senegal [20, 52]. Briefly, males and females are separated by self-stocking [49], over an average of 4–5 days, where the females emerge in the first two days (to keep them in the colony), and are immediately followed by the emergence of males. The exact age of emergence of the flies depends on the species and the environmental conditions, such as temperature and humidity [57]. The male pupae are usually kept for several daily collections at 10 °C and kept at the same temperature for irradiation to suppress emergence [47, 52]. After irradiation, the pupae are immediately packed in insulated containers with phase change material (ClimSel™ C7, Climator Sweden AB, Skövde, Sweden) to sustain chilling at ca. 10 °C as they are moved by various means of transport, to emergence facilities near target release areas [20, 52]. The handling processes during preparation and transportation may impair the quality of the insects [34, 43, 47].

The chilling period of pupae has been reported to affect the emergence rate as well as the flight ability, survival and mating competitiveness of tsetse flies [52]. Additionally, although irradiation is the cornerstone of a successful SIT program, as it induces the required dominant lethal mutations resulting in death of the embryos [37], it has also been shown to induce unintended somatic damage [59] that affects flight ability [13]. Moreover, a combination of irradiation and chilling resulted in reduced flight propensity and survival of flies [31, 43, 47, 52].

Furthermore, transportation may affect the quality of flies not only due to the chilling [52, 65], but also because of vibration (extended periods of oscillatory movement, as from the running of an internal combustion engine) and shock (brief periods of rapid acceleration and possible rebound) experienced by the pupae from sources such as turbulence during plane flight, rough and bumpy terrain used by vehicles, and acceleration and halting during transportation switches between various means of transport. Vibration and shock studies are common in the automotive industry (to ensure minimization of their effects), during manufacture of vehicles and trucks, and also in the food industry, to assess the damage caused by these modes of transport on transported material [28, 54]. Furthermore, some studies have assessed the impact of vibration and shock on various aspects of human biology and anatomy [55, 63]. In addition, characteristics of vibration and shock, such as the acceleration and its properties (magnitude, direction, frequency and duration), have been shown to have a negative impact on the quality of vegetables and strawberries [28, 48]. However, documentation of studies evaluating the impact of these characteristics on insects (and specifically tsetse flies, and their transportation as pupae) is lacking. Most studies on transportation [20, 52, 65] have neither broken down nor directly measured these characteristics of transportation, leaving it to speculation what the exact impact is of the applied transport methods on the quality of transported insects.

We hypothesized that with pupae of an earlier age, we may be able to eliminate the need for and the impact of chilling before and during irradiation and transportation. The earlier gender-separation of tsetse flies became possible with the invention of a prototype near infrared imaging pupae sex sorter (NIRPSS). This approach was developed to separate tsetse males from females based on the two day-earlier melanization of female wings as compared to male wings during early pupal development (22–23 days old) [8, 21, 41]. The current work was designed to compare the impact of irradiation and transportation (vibration and shock) on the quality of the adults emerging from pupae transported at the age of 22 days without chilling and at 29 days with chilling, which correspond to the two operational approaches in current SIT programs.

## Materials and methods

### Colony source and maintenance of experimental flies

The male pupae and the virgin females used for the experiments were derived from the *Glossina morsitans morsitans* (*Gmm*) colony originating from Zimbabwe and maintained at the Insect Pest Control Laboratory (IPCL), Joint FAO/IAEA Centre of Nuclear Techniques in Food and Agriculture, Seibersdorf, Austria. The colony at the IPCL is maintained using an *in vitro* feeding system with thawed and warmed bovine blood (Svaman spol, s.r.o., Myjava, Slovak Republic) [26]. The blood was kept frozen at −20 °C and irradiated with 1 kGy in a commercial 220 PBq ^60^Co wet storage panoramic shuffle irradiator. The flies were offered a blood meal three times a week. Pupae were incubated at 24.1 ± 0.1 °C and 78.8 ± 3.7% relative humidity (RH) and adults emerged under the same conditions. The above conditions will, henceforth, be referred to as standard laboratory rearing conditions. The emerged flies were collected using the self-stocking of production cage system [49].

### The irradiation process and conditions

Tsetse pupae were irradiated in air at the IPCL using a Gamma cell 220 ^60^Co irradiator (MDS Nordion Ltd., Ottawa, ON, Canada). The dose rate was measured by alanine dosimetry as 2.144 Gy sec^−1^ on 2015-03-03 with an expanded uncertainty (*k* = 2) of 3.2%. The radiation field was mapped using Gafchromic HD-V2 film and the dose uniformity ratio in the volume used for the experiments was < 1.1. The irradiated group was placed in a plastic Petri dish at the centre of a polycarbonate jar (2200 mL) and sandwiched between two phase change material packs (ClimSel™ C7) that kept the temperature below 10 °C during irradiation of the 29-day-old pupae at 110 Gy [47]. The same process was followed for the 22-day-old pupae, but with phase change material to maintain ambient temperatures (ClimSel™ C21, Climator Sweden AB). The non-irradiated flies (0 Gy) were held in the same temperature conditions as the irradiated flies during the irradiation process at both ages [18].

### Packaging and transportation process and conditions

**Figure S1.**
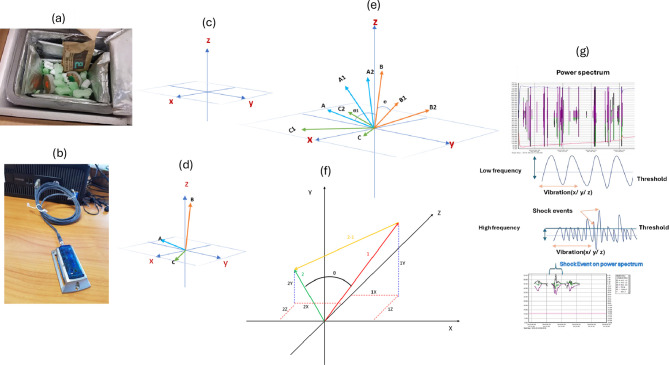
Diagrams and images of materials used for shock evaluation. (a) Image of the MSR 165 data logger used to measure vibration and shock during transportation, (b) Image showing packaging of pupae with S8 (chilled) and humidity packs before transportation of the 29-day-old group in the insulated box, (c) Output of the power spectrum as generated by the MSR 165 data logger software and illustrations of peaks of shock events at high and low frequency, (d, e, f) Illustration of the Cartesian plane showing the axis of movement (x, y, z) of the pupae and package during transportation following the right-hand rule. The length of vectors A, B and C denote the magnitude (scalar) of the vectors, while the angles determine the direction of the vector.

The impact of irradiation and transportation on the quality of sterile *Gmm* adults emerging from pupae that were irradiated at 22 days or 29 days was evaluated during two different periods. Single day collections of *Gmm* pupae (*n = *800–1000) placed in plastic dishes (diameter 5.5 cm, height 1.5 cm) and subsequently maintained under normal rearing conditions were used for each replicate and age. Each batch of 22- or 29-day-old pupae was divided into four experimental groups (*n* ≥ 100): Irradiated and transported (Shipped – 110  Gy), Irradiated but not transported (Unshipped – 110 Gy), non-irradiated but transported (Shipped – 0 Gy) and the control group, which was neither irradiated nor transported (Unshipped – 0 Gy). All experimental groups (including the control group) were sealed in petri dishes (diameter 5.5 cm × height 1.5 cm) containing saw dust to immobilize the pupae during transportation. The two 110 Gy groups of pupae were irradiated as described above. During irradiation, the non-irradiated but transported (Shipped – 0 Gy) and the non-irradiated and non-transported treatments (Unshipped – 0 Gy) of the 29-day-old groups of pupae were also held under chilled conditions (10 °C), like the irradiated flies, while the corresponding 22-day-old groups of pupae were held under ambient temperature in the rearing room. Afterwards, the pupae were placed in vacuum insulated panel transport boxes (AcuTemp, cSafe Global, Monroe, OH, USA) containing polystyrene chips and phase change material (either ClimSel™ C21 for 22-day-old pupae or C7 packs for 29-day-old pupae, Climator Sweden AB). [51]. The non-transported groups were maintained under similar temperature conditions until the end of the transportation period. Humidity packs (Humidipak^®^, Boveda, Minnetonka, MN, USA) were also placed in the boxes to regulate humidity (Fig. S1a), along with a 3-axis acceleration data logger (MSR 165, MSR Electronics GmbH, Seuzach, Switzerland) (Fig. S1b). This robust mini data logger (Size & weight: 39 × 23 × 72 mm, ca. 69 g) records vibrations and shocks along the three orthogonal axes *x*, *y* and *z* (Fig. S1c) and contains a large memory capable of storing over 2 million measured values (https://www.msr.ch/en/product/datalogger-vibration-shock-acceleration-msr165/). All recordings were made at 1600 readings per second. The logger also recorded environmental conditions including temperature, relative humidity, air pressure and light intensity through inbuilt sensors. The logger was programmed to record temperature and the relative humidity data every 3 s, and acceleration whenever it exceeded 3 *g*, (where *g* is referred to as the acceleration of gravity at the earth’s surface, which equals 9.8 ms^−2^ at sea level). Each box was accompanied by a document indicating the number and age of the transported pupae, and the dose and duration of irradiation, and was dispatched from the IPCL by a courier service to the Institute of Tropical Medicine (ITM) insectary at Antwerp, Belgium, from where it was returned and received at the IPCL after 48 ± 12 h. On arrival of the box at Seibersdorf, all data recorded during transportation were downloaded from the MSR 165 logger with MSR 165 Software version 6.04.00 (https://www.msr.ch/en/support/msr-pc-software-standard/) for analysis and the pupae were used for subsequent quality control tests. Pupae and the flies emerging from the 22- or 29-day-old irradiation and transportation treatments, will hereafter also be referred to as the 22-day-old group (5 replicates) and the 29-day-old group (6 replicates).

### Evaluating the impact of shock and vibrations during transportation on the quality of flies

#### Defining vibration and shock variables

Vibration was defined as all the records generated during the transportation where any one of the *x*, *y* and *z* acceleration vectors exceeded 3 *g*. The resultant acceleration vector was calculated from the three (*x*, *y* and *z*) components (see illustrations in Figs. S1c–S1f). For the analysis, the vibration records were filtered to limit the data to events exceeding the thresholds of 5 *g*, 10 *g*, 15 *g* or 20 *g*, which determined the shock events. Each event consisted of a period of shock during which the acceleration exceeded one of these thresholds, until the acceleration fell back below the threshold, including four records before and after the acceleration rose and fell respectively (further descriptions of the shock variables are in File S1). If the acceleration rose again within 8 records, the event was continued until the value remained below the threshold longer than 8 records, resulting in joined events. For the analysis, the following aspects were calculated for each record or pair of records within each event: the magnitude of the resultant vector (the scalar), the change in magnitude between successive scalars within the event, the magnitude of the vector difference between successive vectors, and the angle between successive vectors (Figs. S1c–S1f). In addition, the length of each event in milliseconds and the total number of shock events in each transport replicate were recorded (File S1). Within each event, the maximum and mean values were calculated for scalar, change in scalar, change in vector magnitude, and angle between successive vectors. Finally, the overall maximum and mean values were calculated for all events within the shipment trial.

#### Shock data processing

Data imported from the data logger were processed through various steps before statistical analysis (File S1). The graphical output of the data from the MSR145 software is shown in Figure S1g. In summary, the data were exported from the data logger into csv files. Using the Microsoft Windows shell, the data in each csv file were separated, placing the acceleration and the environmental variables data in two separate csv files. The acceleration data were then filtered according to the thresholds (>5 *g*, >10 *g*, >15 *g*, >20 *g*), after which the csv files were trimmed to exclude any time durations that had been recorded before or after the transportation period, for each replicate. The resulting files were further processed in Excel to generate the values for each record, for the change scalar, the change vector, the angle and the frequency, as detailed in File S1 (Sections 1–4). The files were further processed using a macro, as described in File S1 (Section 5). This macro generated the summary values for the shock variables in each event and for the complete transportation file. This process was repeated for the data from each irradiation and transportation replicate and each threshold. The files containing the environmental data were also trimmed to only include data within the transportation period, and summaries of the maximum, mean and minimum values were calculated for each transportation replicate. The detailed step-by-step process as described above can be found in File S1. The data from each file were then summarized into one table to which the biological data from each indicator of quality were added: emergence, flight and mating ability, insemination rates and the mean spermathecal value (MSV). The data were then used for statistical analysis with R using R-Studio software. To assess the impact of shock on the insemination rate, the spermathecal fill was dichotomized at ≤ 0.5 as being “not full” and those at ≥ 0.75 being analyzed as “full” spermathecae.

### Preliminary experiment on the impact of irradiation of 22-day-old pupae on induced sterility in males

To determine the optimal radiation dose to sterilize *Gmm* male pupae at the age of 22 days, a range of radiation doses (0, 20, 30, 40, 50, 70, 90, 110 Gy) was used for irradiation in air of both males and females, as it was not possible to separate the sexes at the time of this experiment. Irradiated virgin females aged 2–3 days, from the different irradiation doses, were mated with non-irradiated males aged 5–7 days, while irradiated males aged 5–7 days were mated with 2–3-day-old non-irradiated virgin females in separate netted cubic mating cages (45 × 45 × 45 cm) as apart from the aforementioned reason, this was the first dose response curve for irradiation at early stage and it was important to establish the sterility of males and females. Mating events were observed under standard tsetse rearing conditions from 9:30 to 12:30 h to cover the morning mating activity peak [45]. Mating pairs that formed were immediately collected using small tubes (4 cm diameter × 6 cm high) and kept for 24 h (overnight) for copulation. After separation, the males were removed from each tube and the females retained in the tubes for 60 days post mating time to observe productivity. For each radiation dose and the non-irradiated control, more than 20 individual flies were tested to determine their fecundity and induced sterility. The dose of 110 Gy to obtain at least 95% sterility for *Gmm* in late-stage pupae has previously been used in other studies [19, 23].

### Quality control tests

#### Emergence rate and flight propensity

The emergence rates and flight propensity were determined simultaneously. After transportation, the 22-day-old pupae were returned to colony conditions to wait for their emergence and placed in flight ability cages, for the male and female emergence and flight scoring. The 29-day-old pupae were immediately placed in the flight cages for scoring of the same, as soon as they arrived at the IPCL in Seibersdorf. Briefly, pupae from the four experimental groups from either age group were placed in different petri dishes and placed inside a black polymethyl methacrylate flight tube (8.9 cm diameter, 0.3 cm thick wall, 10 cm high), with its base covered by a petri dish with black porous paper. The walls of the tubes were coated with unscented talcum powder to prevent the emerging flies from crawling out of the tube [25, 52]. Each tube was subsequently placed in a netted cubic cage (45 × 45 × 45 cm) under a light intensity of 1500 lux for observation of emergence and flight propensity. The emergence took place for 2 days and the total number of emerged flies *versus* non-emerged pupae were recorded. The number of adults that escaped from the flight tubes were scored as flyers, while those that were unable to escape the tubes were considered non-flyers. The number of flyers was divided by the total number of adults that emerged, to calculate the flight propensity. The emerged male flies from pupae irradiated at both ages were kept in standard fly holding cages (20 cm diameter and 7 cm height) under standard laboratory rearing conditions for use in the mating test.

#### Mating ability, insemination rate and mean spermathecal value (MSV)

At the age of 6–7 days (after the emergence and flight propensity testing) 30 male flies (flyers and non-flyers) from each of the four experimental groups (from each age group) were mated with 2–3-day-old virgin colony females at the ratio of 1:1, in four different mating cages (45 × 45 × 45 cm). A total of 30 females were released first, and 5 min later, the males were also released into the cage. Mating events were observed under standard tsetse rearing conditions from 9:30 a.m. to 12:30 p.m. to cover the morning mating activity peak [45]. When the mating pairs had formed, they were gently collected in individual mating tubes (6 cm × 3.5 cm), where they were held together for 24 h, after which the males were removed from the tubes. The female flies were immediately dissected in phosphate-buffered saline under a binocular microscope, and the insemination rate (the number of female flies that were inseminated *versus* the ones which were not inseminated) and the contents in each spermathecal pairs were assessed at ×100 magnification using a Carl Zeiss compound microscope [44]. The spermathecal fill was scored to the nearest quarter for each spermatheca as: empty (0), quarter full (0.25), half-full (0.50), three-quarter-full (0.75) and full (1.0) [68]. The amount of sperm transferred was then computed as the mean spermathecal value per spermatheca pair. The male mating ability was calculated according to the proportion of males that had mated against the total number released, from each treatment [45].

#### Survival experiment

The remaining flies in the different groups of pupae (apart from those used in the other quality control tests), were used to assess the survival of irradiated and transported flies of the 22- and 29-day-old groups. After completion of emergence (2 days), a sample of 30 male flies from each of the four experimental groups was randomly selected and placed in production cages (diameter 11 cm × 5 cm) in three technical replicates. This process was repeated twice, resulting in two biological replicates for the flies irradiated at both ages, as well as each treatment. The flies were subsequently maintained under normal rearing conditions. Mortality was recorded 2–3 times a week until all flies had died or were at least 100 days old.

#### Statistical analysis

The statistical analysis was performed using R software [60] with the packages ggplot2 [77] to create graphics (plots), ggfortify [32] to visualize unified plots using ggplot2, lme4 [12] for fitting and analyzing mixed models, gcookbook [17] to generate high quality graphs, nlme [58] for linear and nonlinear mixed effects models, lattice [64] for robust and aesthetic data, MuMin [11] for graphics and data visualization, MASS for data transformation [72], plyr [78] for splitting, manipulating and recombining the split data, dplyr [79] for data manipulation, ranger [81] for fast implementation of random effects for high dimensional data, survival [71] to obtain survival curves, coxme [70] to analyze survival data using fixed and random effects, rcompanion [40] and tidyverse [80] for science data structure, and knitr [83] and RMarkdown [82] to produce an output report of the analysis code/commands (supplementary materials). Generalized Linear Mixed Models (GLMM) fit by maximum likelihood (Laplace Approximation) [12] were used to analyze the emergence rates, flight propensity, mating ability, insemination rates and survival of flies at specified time periods (after 15, 30 and 60 days) during the survival period. Kaplan–Meier Analysis (km) survival curves were used to compare the longevity of flies over the 100-day period. The survival curves were compared between treatments using the coxme model where repetitions were considered as random factors [70]. The figures were generated using the tidyverse package [80]. The GLMM method was also used to build models in the analysis of the vibration and shock data, to determine the relationship of the shock variables and the biological quality of the flies. At least two predictors (shock variables) or more were used as fixed variables to correlate the biological response (emergence, flight propensity, mating or insemination rate) to shock during transportation (File S2). Before the models were built, each biological response was assessed by plotting it against each shock variable. Only shock variables that displayed a linear relationship with the biological quality parameters on a scatter plot (*R* ^2 ^> 0.2) were selected to be included in the models. The predictors (shock variables) were added one to another or individually along with the environmental variables and the irradiation treatment. After modelling the response of each biological quality parameter to different combinations of shock variables, the model with the lowest AICc value was selected as the best model for interpretation of the effect of shock on the biological quality parameter tested.

## Results

### Preliminary experiment on sterility test on 22-day-old pupae

The irradiated male pupae at 22 days of age exhibited 90% sterility at 90 Gy and 100% sterility at 110 Gy at which no pupae were produced. Mating of irradiated females with fertile males did not produce any pupae at any of the tested doses, indicating that even the lowest dose of 20 Gy caused 100% sterility in females irradiated at 22 days of age (Table S1) which was an interesting result as the sterility of females need to be understood, in case of accidental release in the field.

### Temperature and relative humidity during transportation

The minimum, average and maximum temperatures inside the insulated box (from all the replicates) during transportation of pupae from Seibersdorf to Antwerp and back at the age of both 22 and 29 days are shown in Table S2. Temperature was strongly positively correlated with relative humidity during the transportation of the 22-day-old pupae (*R* ^2^ *= *0.9, *t* = 75.182, df *= *1, *p* < 0.001), but was not significantly correlated with RH for transportation of 29-day-old pupae (Table S2, File S2). There was a significant difference in the minimum temperature (*t* = −15.40, *p* < 0.001) between the 22- and 29-day-old pupae, as also evident in the average temperatures (*t* = −7.571, *p* < 0.001), with both being higher for the 22-day group packaging than the 29-day group. However, there were no differences in the maximum temperatures (28.3 (± 1.4) and 25.6 (± 1.5)) of the 22- and 29-day-old groups, respectively (Table S2, File S2). There was also a significant difference in the maximum relative humidity (*t* = 5.302, *p* < 0.001) between the 22 and 29 day-olds as well as the average relative humidity (*t* = 10.733, *p* < 0.001), but no significant difference in the minimum relative humidity (Table S2, File S2).

### Quality control tests: impact of irradiation and transportation on the biological quality of emerging flies

#### Emergence rates

**Figure 1:**
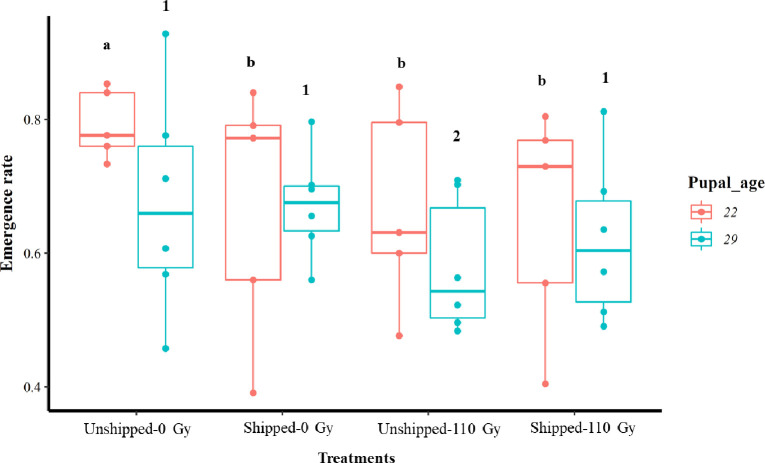
Emergence rate of 22- and 29-day tsetse (*Glossina morsitans morsitans*) pupae groups that were irradiated and transported. Pupae were irradiated and transported at either 22 or 29 days of age from pupation. Comparison of emergence rates between treatment groups at each age. The bars represent the minimum, first quartile, median, third quartile and maximum for each treatment. Bars indicated by the same lower-case letter or number did not differ significantly at the 5% level.

**Figure S2.**
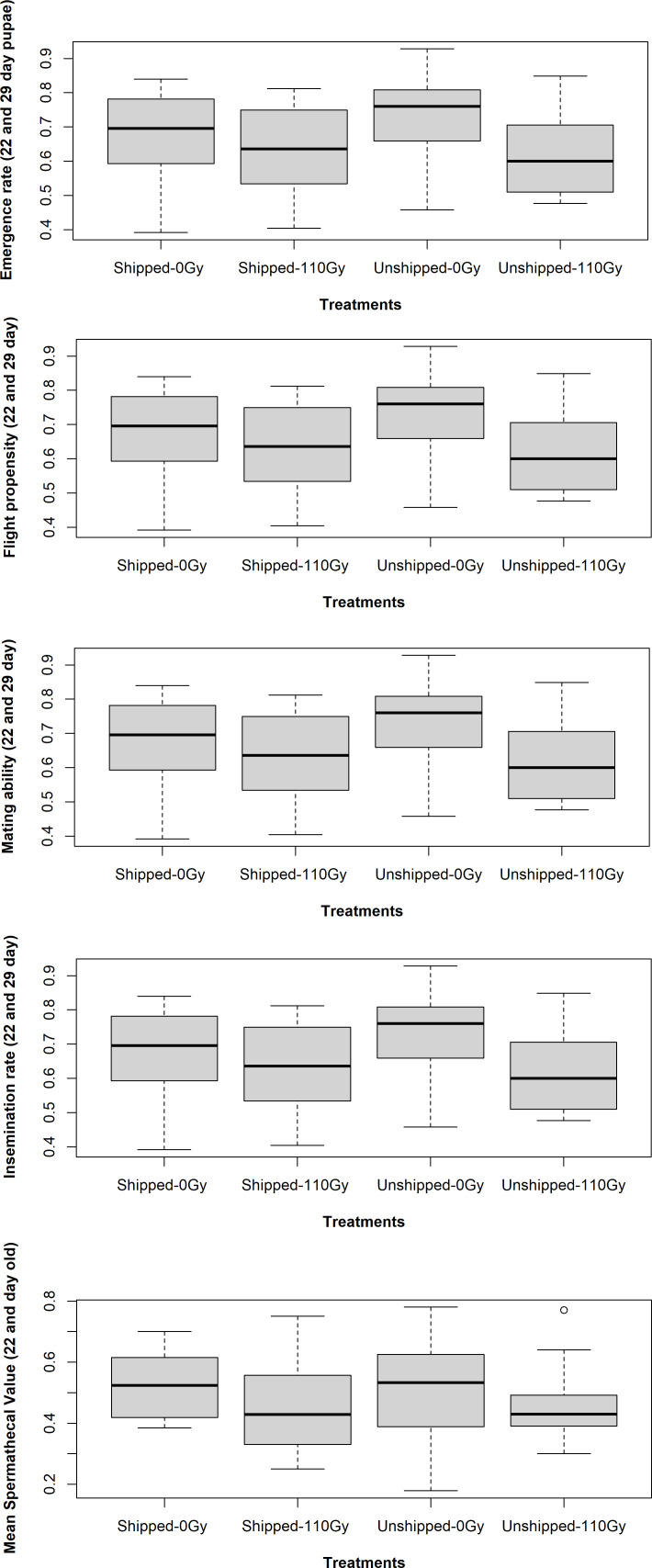
Combined rates of biological parameters following irradiation and transportation of *Glossina morsitans morsitans (Gmm)* pupae at the age of 22 and 29 days (a) Cumulative emergence rates, (b) Cumulative flight propensity, (c) Cumulative mating ability, (d) Cumulative insemination rates, (e) Cumulative mean spermathecal value (MSV) of *Gmm* females inseminated by males that were irradiated and transported as pupae at the age of 22 and 29 days.

**Figure S3.**
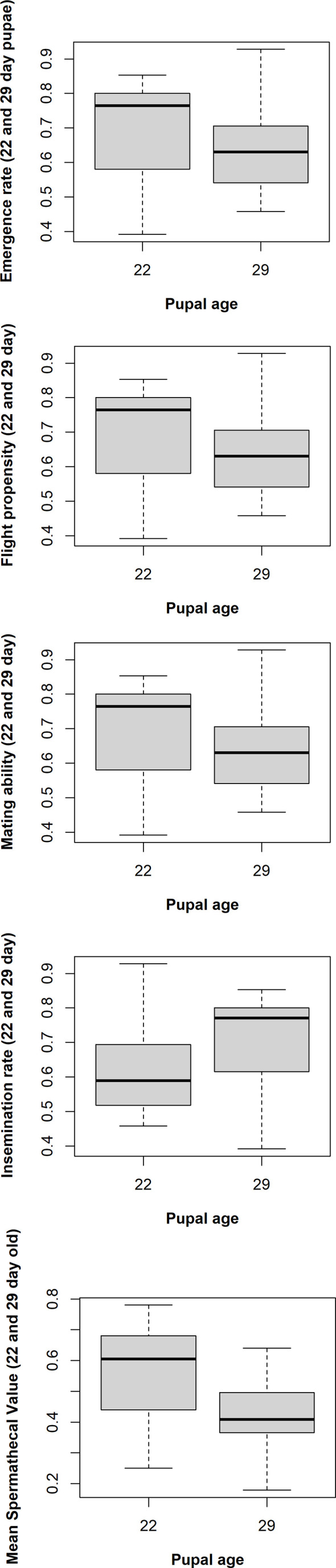
Comparison of biological parameters between *Glossina morsitans morsitans (Gmm)* flies irradiated and transported as pupae at 22 and that of 29 days regardless of treatment. (a) Comparison of emergence rate, (b) Comparison of flight propensity, (c) Comparison of mating ability, (d) Comparative insemination rate, (e) Comparison of the MSV between *Gmm* females inseminated by males transported at the age of 22 days and at 29 days regardless of treatment.

The overall emergence proportions were higher in the 22-day-old group than in the 29-day-old group, regardless of treatment (Table 1, Fig. 1). The emergence rate of the 22-day-old pupae was significantly reduced by irradiation (Unshipped – 110 Gy), transportation (Shipped – 0 Gy), and by their combined effect (Shipped – 110 Gy), relative to the control group (Unshipped – 0 Gy) (Table S3, Fig. 1, File S2). However, the 29-day-old pupae group was negatively affected only by irradiation (Unshipped – 110 Gy), but no significant effect was observed for transportation or their combined effect, relative to the control group (Table S3, Fig. 1, File S2). Collectively, the 22- and 29-day-old pupae were significantly negatively affected by irradiation, transportation and combining irradiation and transportation, relative to the cumulative control groups (Fig. S2a, File S2). The 22-day-old pupae exhibited higher emergence rates compared to the 29-day-old pupae, regardless of treatment (Table S3, Fig. S3a, File S2).

**Table 1:** Summaries of proportions of flies that emerged, took flight, mated and inseminated (with their MSV) (±SE), from each treatment of the irradiated and transported pupae (at 22 and 29 days). The MSV values are means of all the spermathecal values in each treatment. Treatments with asterisks are those that were significant from the controls (Unshipped – 0 Gy).

Treatment	Age	Emergence	Flight propensity	Mating ability	Insemination	Mean spermathecal value (MSV)
Shipped – 110 Gy	22	0.65 ± 0.08***	0.85 ± 0.06***	0.41 ± 0.11***	0.88 ± 0.04**	0.50 ± 0.08
Shipped – 0 Gy	22	0.67 ± 0.08***	0.91 ± 0.05	0.73 ± 0.14**	0.94 ± 0.02*	0.60 ± 0.05
Unshipped – 110 Gy	22	0.67 ± 0.07***	0.95 ± 0.02	0.63 ± 0.12***	0.95 ± 0.02	0.50 ± 0.08
Unshipped – 0 Gy	22	0.79 ± 0.02	0.96 ± 0.02	0.82 ± 0.06	0.99 ± 0.01	0.67 ± 0.03
Average	22	0.70 ± 0.06	0.92 ± 0.03	0.65 ± 0.11	0.94 ± 0.03	0.57 ± 0.06
Shipped – 110 Gy	29	0.62 ± 0.05	0.68 ± 0.09	0.79 ± 0.03	0.91 ± 0.03*	0.41± 0.06
Shipped – 0 Gy	29	0.67 ± 0.03	0.67 ± 0.10	0.77 ± 0.05	0.93 ± 0.03**	0.46 ± 0.03
Unshipped – 110 Gy	29	0.58 ± 0.04**	0.59 ± 0.05***	0.8 ± 0.05	0.95 ± 0.02**	0.43 ± 0.05
Unshipped – 0 Gy	29	0.67 ± 0.07	0.67 ± 0.09	0.79 ± 0.04	0.80 ± 0.03	0.38 ± 0.05
Average	29	0.64 ± 0.05	0.65 ± 0.08	0.79 ± 0.05	0.90 ± 0.03	0.47 ± 0.05
Overall total		0.67 ± 0.06	0.78 ± 0.06	0.72 ± 0.08	0.92 ± 0.03	0.50 ± 0.05

Values that differ significantly from the control (Unshipped – 0 Gy) within each age and treatment are indicated by asterisks (*p < 0.05 < ** *p* < 0.01 *** *p* < 0.001). The statistical test values are given in Table S3 of the supporting information**.**

##### Flight propensity

**Figure 2:**
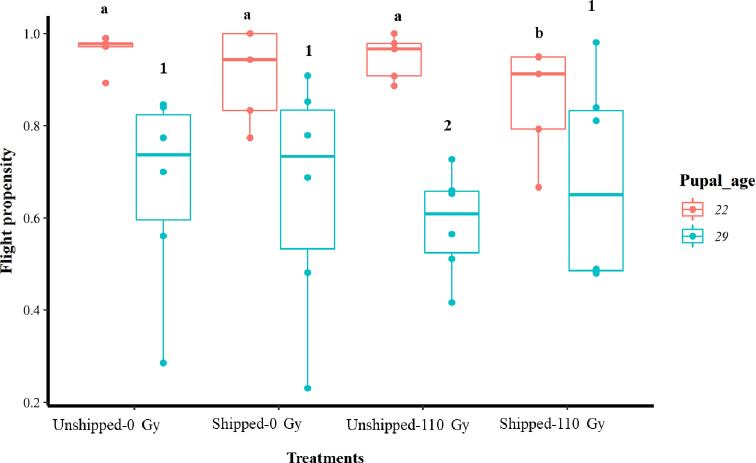
Flight propensity of *Glossina morsitans morsitans* tsetse flies that emerged from treatments of irradiation and transportation of 22- and 29-day-old pupae groups. Pupae were irradiated and transported at either 22 or 29 days of age from pupation. Comparison of flight propensity between treatment groups at each age. The graphs represent the minimum, first quartile, median, third quartile and maximum for each treatment. Bars indicated by the same lower-case letter or number did not differ significantly at the 5% level.

Overall, regardless of the treatment, an average higher proportion of flyers was observed within the 22-day-old group compared to the 29-day-old group (Table 1 and Fig. 2). Combined irradiation and transportation (Shipped – 110 Gy) of pupae at 22 days of age had a significant negative effect on their flight propensity, relative to the control group (Unshipped – 0 Gy) (Table S3, Fig. 2, File S2) while irradiation only (Unshipped – 110 Gy) of the 29-day-old group had a significant negative effect on the flight propensity of emerged flies (Table S3, Fig. 2, File S2). Regardless of age (combining the 22- and 29-day-old pupae), there was a significant negative reduction of flight propensity by irradiation, transportation and the combination of irradiation and transportation, relative to the control (Table S3, Fig. S2b, File S2). Considering the two age groups, there was a significantly higher number of flies that took flight from the 22-day-old pupae group compared to the 29-day-old pupae (Table 2, Fig. S3b, File S2).

**Table 2:** List of best models explaining the impact of shock events on the biological performance indicators.

Biological quality indicator	Best model (cumulative impact of shock/environmental predictors)	Significant predictors in the models
Emergence rate	fm29 <-glmer(cbind(emerged,unemerged) ~ Irradiation + changescalar_mean_15max + Changevector_mean_15max + (1|Replicate), family*=*binomial, data*=*tab)	Irradiation/changescalar_mean _15max/Changevector_mean_15max
Flight propensity	fm29b <- glmer(cbind(in.,out) ~ Age 29 + Temp_mean + Angle_mean_15max + (1|Replicate), family*=*binomial, data*=*tab)	Age (29 days old) /temperature means
Mating ability	fm2 <- glmer(cbind(pairs_formed,unformed_pairs) ~ Irradiation + RH._Max + Temp_mean + changescalar_mean_5max + Changevector_mean_5max + (1|Replicate), family*=*binomial, data*=*tab)	Relative humidity/Temperature means/Changescalar_mean_5max/Changevector_mean_5max _
Insemination rate	fm12 <- glmer(cbind(full,not_full) ~ Irradiation + Temp_mean + Duration.ms._10mean + changescalar_mean_10max+ (1|Replicate), family*=*binomial, data*=*tab)	Temperature means /Duration.ms._10mean/ Changescalar mean_10max
Mean spermathecal value (MSV)	fm28 <- lme(MSV~ Irradiation + Duration.ms._15mean, random=~1|Replicate, data*=*tab)	Duration(ms). _15mean

##### Mating ability

**Figure 3:**
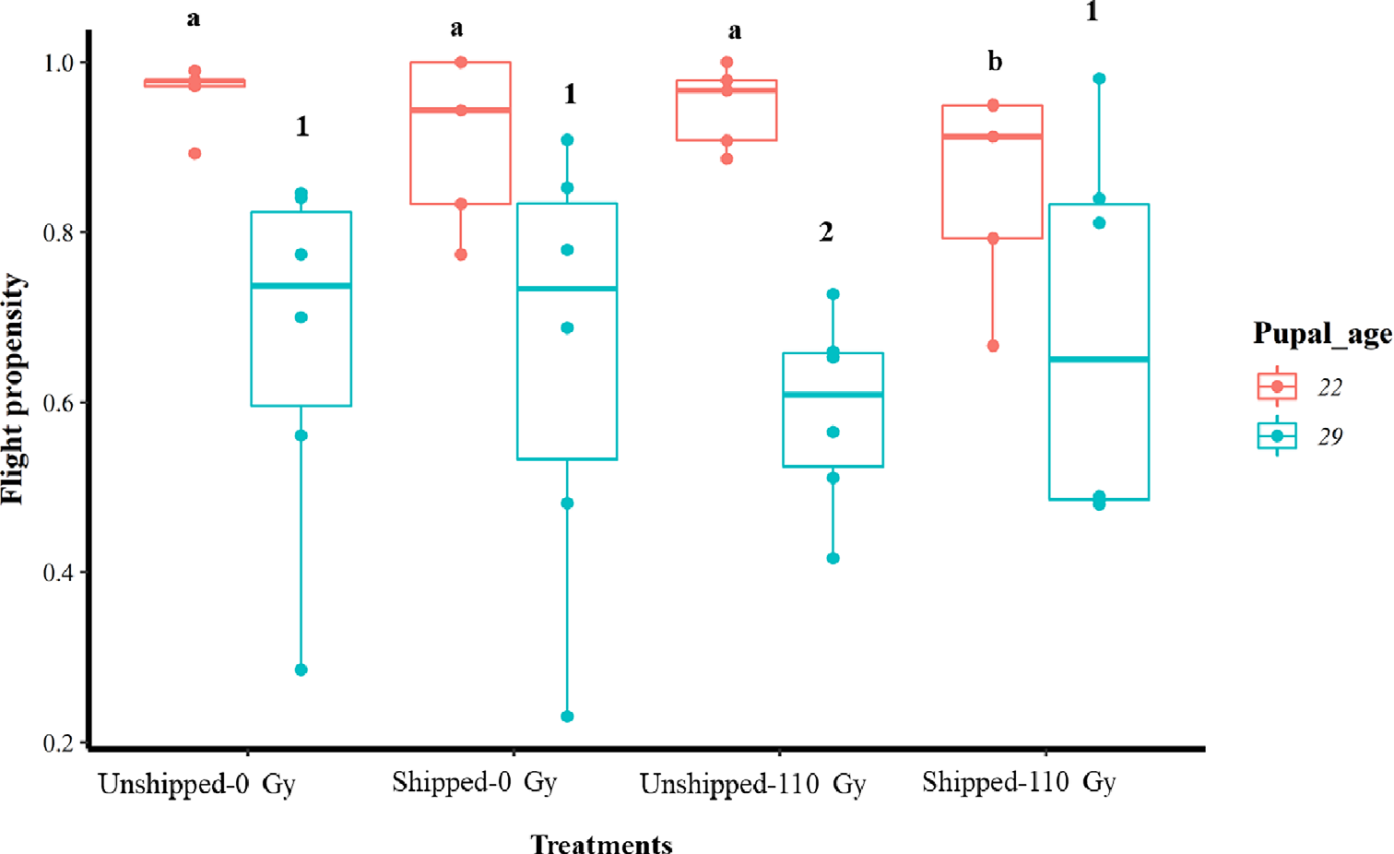
Mating ability of *Glossina morsitans morsitans* tsetse flies that emerged after irradiation and transportation of 22- and 29-day-old pupae groups. Pupae were irradiated and transported at either 22 or 29 days of age from pupation. Comparison of mating ability between treatment groups of each age. The graphs represent the minimum, first quartile, median, third quartile and maximum for each treatment. Bars indicated by the same lower-case letter or number did not differ significantly at the 5% level.

The overall mating proportions of males regardless of the treatment were higher in the 22-day-old group than the 29-day-old pupae (Table 1, Fig. 3). The average mating proportions of the 22 day- and 29-day-old pupae groups are shown in Table 1. The mating ability of flies from the 22-day-old group were significantly reduced by irradiation (Unshipped – 110 Gy), transportation (Shipped – 0 Gy) and by irradiation combined with transportation (Shipped – 110 Gy), compared to control groups (Unshipped – 0 Gy) (Table S3, Fig. 3, File S2). In contrast, there were no significant differences in the mating ability of flies from all the other treatments relative to the control in the 29-day-old group (File S2). Collectively, the mating ability of the 22- and 29-day-old pupae was significantly reduced by irradiation, transportation and the combination of irradiation and transportation (Table S3, Fig. S2c, File S2). Regardless of treatment, the number of mating pairs was significantly higher in the 22-day-old group, compared to the 29-day-old group (Table S3, Fig. S3c, File S2).

##### Insemination rate and the mean spermathecal value (MSV)

**Figure 4:**
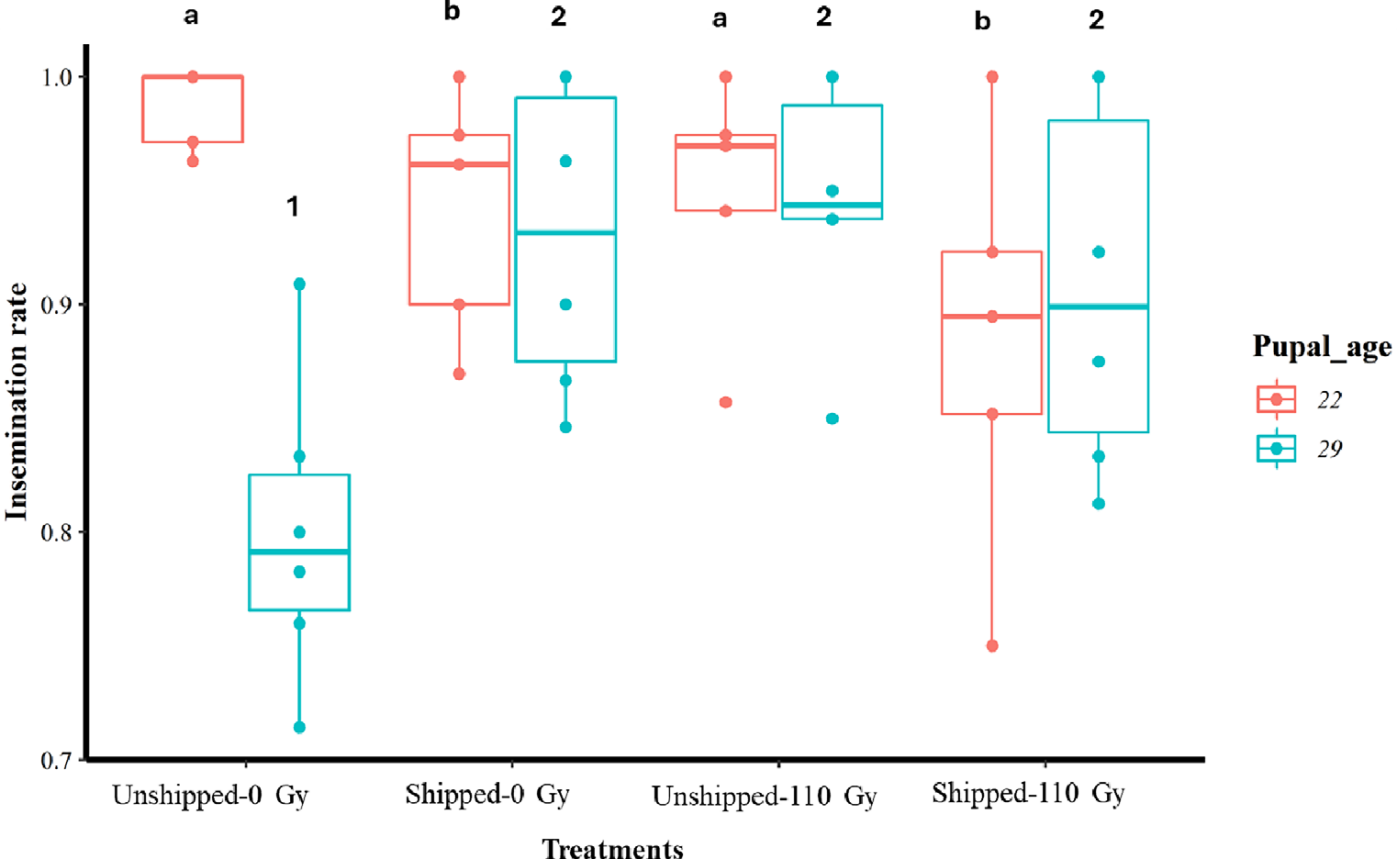
Insemination rate of *Glossina morsitans morsitans* female flies mated with *Glossina morsitans morsitans* tsetse males that emerged from the irradiation and transportation of 22- and 29-day-old pupae groups. Pupae were irradiated and transported at either 22 or 29 days of age from pupation. Comparison of insemination rates between treatment groups at each age. The graphs represent the minimum, first quartile, median, third quartile and maximum for each treatment. Bars indicated by the same lower-case letter or number did not differ significantly at the 5% level.

**Figure 5:**
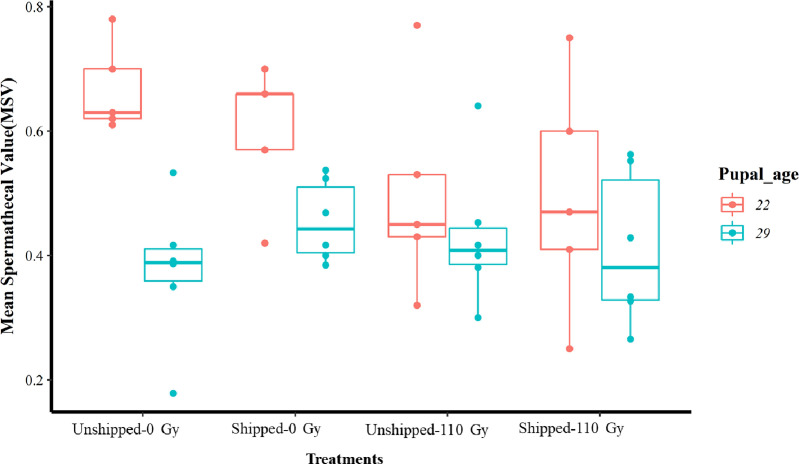
Mean spermathecal value (MSV) of *Glossina morsitans morsitans* tsetse females mated with *Glossina morsitans morsitans* tsetse males that emerged from the irradiation and transportation (relative to the controls), of the 22- and 29-day-old pupae groups. Pupae were irradiated and transported at either 22 or 29 days of age. Comparison of MSV between treatment groups at each age. The graphs represent the minimum, first quartile, median, third quartile and maximum for each treatment. There were no significant differences within the groups.

The average proportions of female flies inseminated together with their MSV from all treatments, for both 22- and 29-day-old pupae, are shown in Table 1. There was a significant reduction in the insemination rate of female flies by males that had only been transported (Shipped – 0 Gy) as well those that had been irradiated and transported (Shipped – 110 Gy), relative to the controls, for the 22-day-old group (Table S3, Fig. 4, File S2). Interestingly, there was a significant increase in the insemination rate of female flies by males from the 29-day-old group, that were irradiated, transported, and both irradiated and transported (Table S3, Fig. 4, File S2). There were no significant differences in the insemination rates of females, by males from all treatments when both age groups were combined (Table S3, Fig. S2d, File S2). However, there were significantly higher insemination rates in the 29-day-old group (Table S3, Fig. S3d, File S2) compared to the 22-day-old group, regardless of treatment. Assessment of the MSV showed that there were no significant differences between treatments, relative to the controls in each age group (Fig. 5

, File S2). Similarly, there were no significant differences between the treatments, relative to the control with the age groups combined (Table S3, Fig. S2e, File S2). Nevertheless, compared to each other and regardless of the treatment, the 22-day-old group had a significantly higher MSV than the 29-day-old group (Table S3, Fig. S3e, File S2).

**Figure 6:**
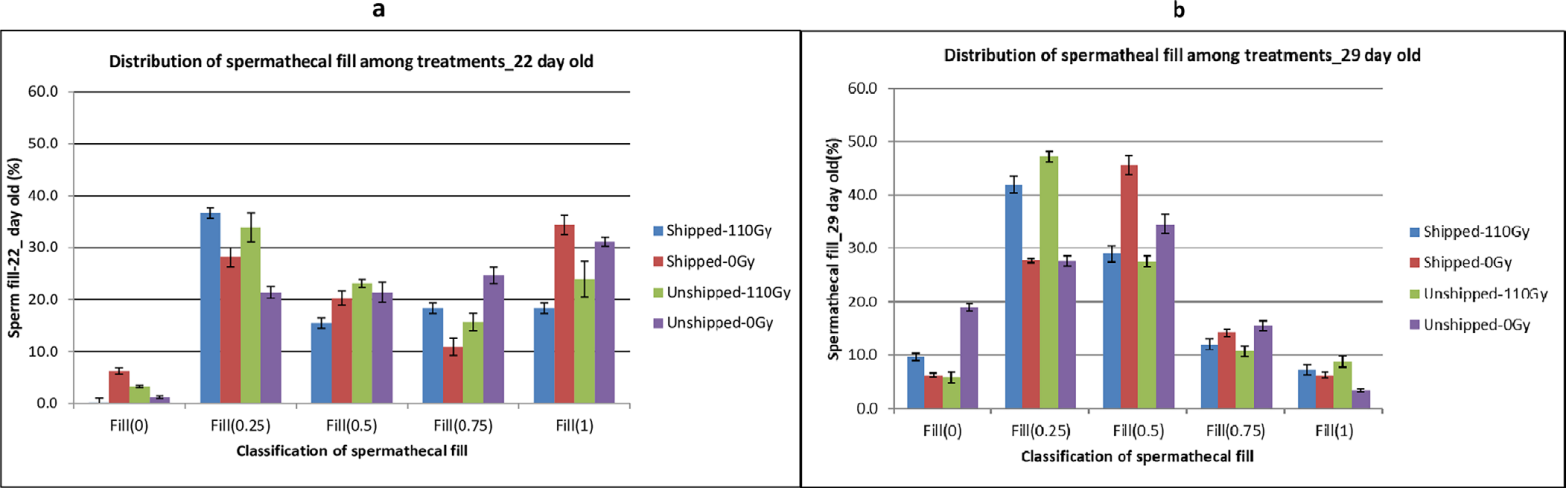
Distribution of spermathecal fill in *Glossina morsitans morsitans* (*Gmm*) females mated with *Gmm* males that emerged from the irradiation and transportation (relative to the controls), of 22- and 29-day-old pupae groups. Comparison of spermathecal fill among treatments of: (a) Females inseminated by *Gmm* male flies that were irradiated and transported as pupae at the age of 22 days; (b) Females inseminated by *Gmm* male flies that were irradiated and transported as pupae at the age of 29 days.

Comparison of differences in spermathecal fill among treatments showed that in the 22-day-old pupae group, there were marginal differences in the spermathecal fill among the four treatment groups (X^2 ^= 7.70, df *= *3, *p* = 0.05, File S2). However, when the treatment groups were considered separately, there were significant differences in spermathecal fill (0, 0.25, 0.5, and 1) within the only irradiated group (Unshipped – 110 Gy) (X^2 ^= 31.19, df *= *4, *p* < 0.001, File S2), the only transported group (Shipped – 0 Gy) (X^2 ^= 34.81, df *= *4, *p* < 0.001, File S2), the transported and irradiated group (Shipped – 110 Gy) (X^2 ^= 13.437, df *= *4, *p* < 0.001, File S2) and the control group (Unshipped – 0 Gy (X^2 ^= 38.53, df *= *4, *p* < 0.05, File S2), the lowest percentages of flies being those that were empty (0) and the highest numbers generally being in those with a quarter fill (0.25). Notably, irradiation and transportation had the lowest percentages of empty spermatheca and the highest in quarter filled spermathecae (Fig. 6a). Likewise, for the 29-day-old pupae, there were no significant differences among the treatment groups, but within the groups, there were significant differences in the distribution of spermathecal fill (0, 0.25, 0.5, and 1) in the only irradiated group (X^2 ^= 61.039, df *= *4, *p* < 0.001, File S2), the only transported group (X^2 ^= 62.821, df *= *4, *p* < 0.001, File S2), the irradiated and transported group (X^2 ^= 55.43, df *= *4, *p* < 0.001, File S2), and the control group (X^2 ^= 32.621, df *= *4, *p* < 0.001, File S2) with the lowest percentages of flies being those that were full (1) and empty (0) and the highest being those that were quarter (0.25) and half filled (0.5) (Fig. 6b). Interestingly, all treatments had higher percentages of full flies compared to the controls.

##### Survival rates

**Figure 7:**
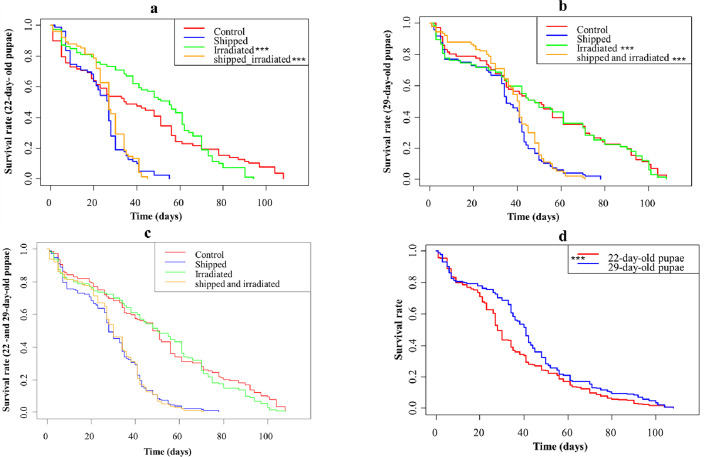
Kaplan–Meir estimator survival plot, based on the events of deaths (in >100 days), of *Glossina morsitans morsitans* males emerging from the irradiation and transportation (relative to the controls) of 22 day and 29 day-old groups. (a) Survival rate (in >100 days) of flies transported as pupae at the age of 22 days; (b) Survival rate (in >100 days) of flies transported as pupae at the age of 29 days; (c) Cumulative survival rate of flies transported as pupae at the age of 22 and 29 days combined; (d) Survival plot showing the survival rate of flies irradiated and transported as pupae at the age of 22 days and 29 days. Values indicated by ^***^ differed significantly (*p* < 0.001) relative to the controls (unshipped – 0 Gy).

The survival over a 100-day period and more showed that males from the irradiated and transported treatment (Shipped – 110 Gy) and the only-transported treatments (Shipped – 0 Gy) of the 22-day-old group survived the shortest time (approximately 45 days and 50 days, respectively), while the control (Unshipped – 0 Gy) and the only-irradiated (Unshipped – 110 Gy) groups survived the longest (approximately 90 days and 110 days, respectively). Notably, increased numbers of only-irradiated flies survived significantly longer, while more irradiated and transported flies survived significantly shorter time periods than the control flies (Table S3, Fig. 7a, File S2). The same trend of a higher number of flies surviving the shortest time duration in the irradiated and transported and the only-transported groups, and the longest time duration in the only-irradiated and control treatments was observed in the 29-day-old group. Increased numbers of flies from the irradiated and transported group also survived a significantly shorter time duration, while the irradiated group survived a significantly longer time duration, compared to the control group (Table S3, Fig. 7b, File S2). Similarly, combining the 22 day-old and 29-day-old groups yielded a survival curve of approximately 74 days and 80 days, respectively for the irradiated plus transported and the only-transported group, while the only-irradiated and the control groups survived the longest periods, of more than 100 days. The only-irradiated group lived significantly longer, while the irradiated and transported group lived significantly shorter relative to the control (Table S3, Fig. 7c, File S2). Overall, regardless of treatment, the 29-day-old group survived for a significantly longer period than the 22-day-old group (Table S3, Fig. 7d, File S2).

**Figure 8:**
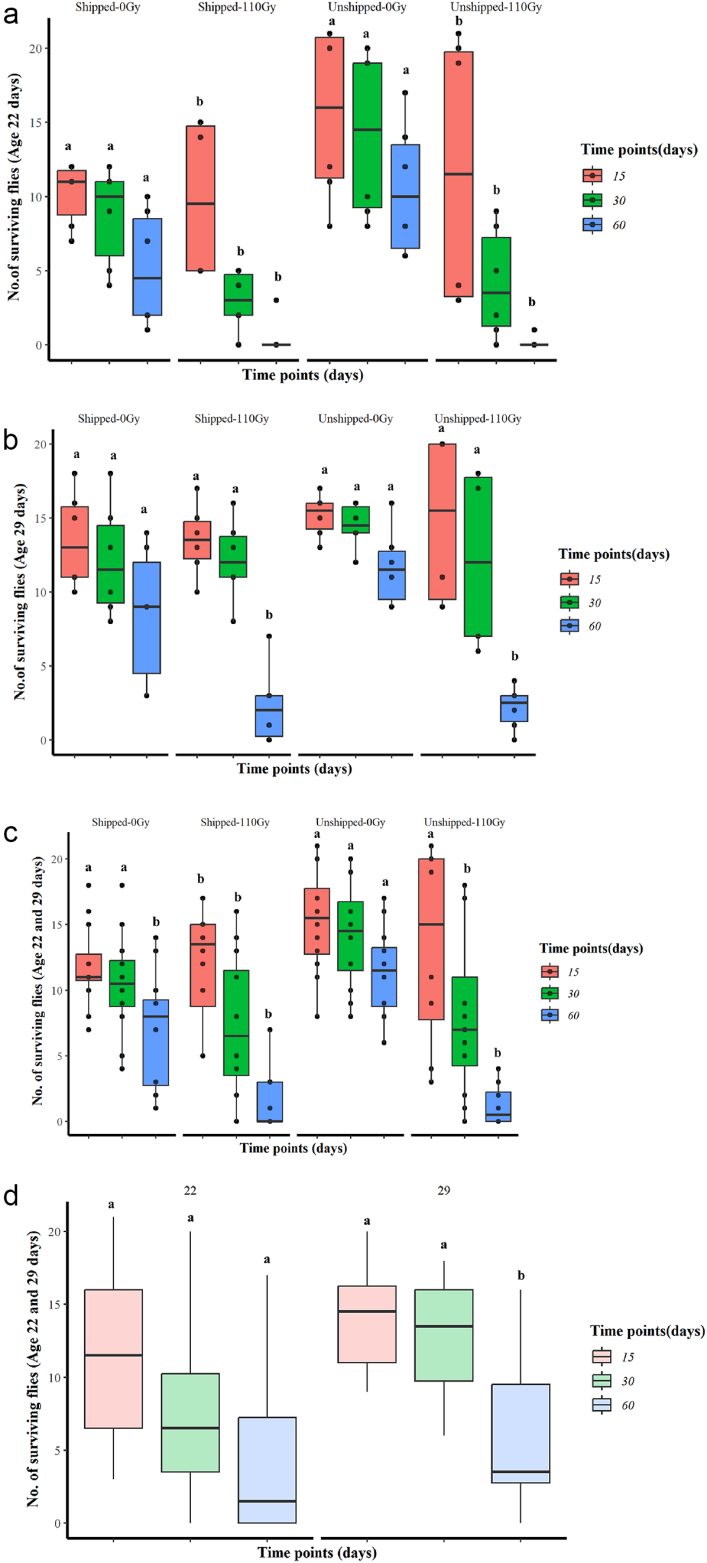
Boxplots based on the binomial analysis of the survival on the 15th, 30th and 60th day, of male flies emerging from the irradiation and transportation (relative to the controls), of 22- and 29-day-old pupae groups. (a) Survival on the 15th, 30th and 60th days of the 22-day-old group; (b) Survival on the 15th, 30th and 60th day of the 29-day-old group; (c) Survival of male flies from the 22- and 29-day-old groups collectively on the 15th, 30th and 60th day; (d) Comparison of survival rate at on the 15th, 30th and 60th day between the 22- and 29-day-old groups regardless of treatment. Values indicated by the same lower-case letter did not differ significantly from the control (Unshipped – 0 Gy).

Analysis of survival of the flies showed that at 15, 30 and 60 days, the number of flies surviving from the 22 day-old group were significantly reduced by irradiation alone (Unshipped – 110 Gy) (*z* = −2.430, *p* < 0.05; *z* = −7.179, *p* < 0.001; *z* = −4.786, *p* < 0.001, respectively, File S2) and by irradiation and transportation (*z* = −2.377, *p* < 0.05; *z* = −7.259, *p* < 0.001; *z* = −5.821, *p* < 0.001, respectively) relative to the control (File S2). Transportation alone had no significant effect on the survival of pupae at the 15th, 30th or 60th day (Fig. 8a, File S2). In contrast, the number of flies surviving from the 29-day-old group was significantly reduced only at 60 days by the only-irradiated (*z* = −6.987, *p* < 0.001) and the combined irradiated and transported (*z* = −7.070, *p* < 0.001) treatments, relative to the control (File S2). Transportation alone did not significantly reduce the survival on the 15th, 30th or 60th day of the 29-day-old group (Fig. 8b, File S2). Collectively, the 22- and 29-day-old group survival was significantly reduced on the 15th, 30th and 60th day (*z* = −2.578, *p* < 0.01; *z* = 6.347, *p* < 0.001; *z* = −9.555, *p* < 0.001, respectively) by transportation alone and combined irradiation and transportation and increased on the 15th day) by combined irradiation and transportation relative to the control (File S2). Also, at 30 and 60 days, the survival was reduced by irradiation alone (*z* = −6.223, *p* < 0.001; *z* = −9.794, *p* < 0.001, respectively), while transportation alone significantly increased the survival at 60 days (*z* = −2.374, *p* < 0.05), relative to the control (Fig. 8c, File S2). There was no significant difference in survival between the 22 day-old and 29-day-old groups on the 15th and 30th day, in contrast to the 60th day where the 29-day-old group had a significantly higher survival rate (*z* = 2.577, *p* < 0.01) than the 22-day-old group, regardless of treatment (Fig. 8d).

### Vibration and shock analysis: impact of vibration and shock during transportation on the biological quality of flies

**Figure 9:**
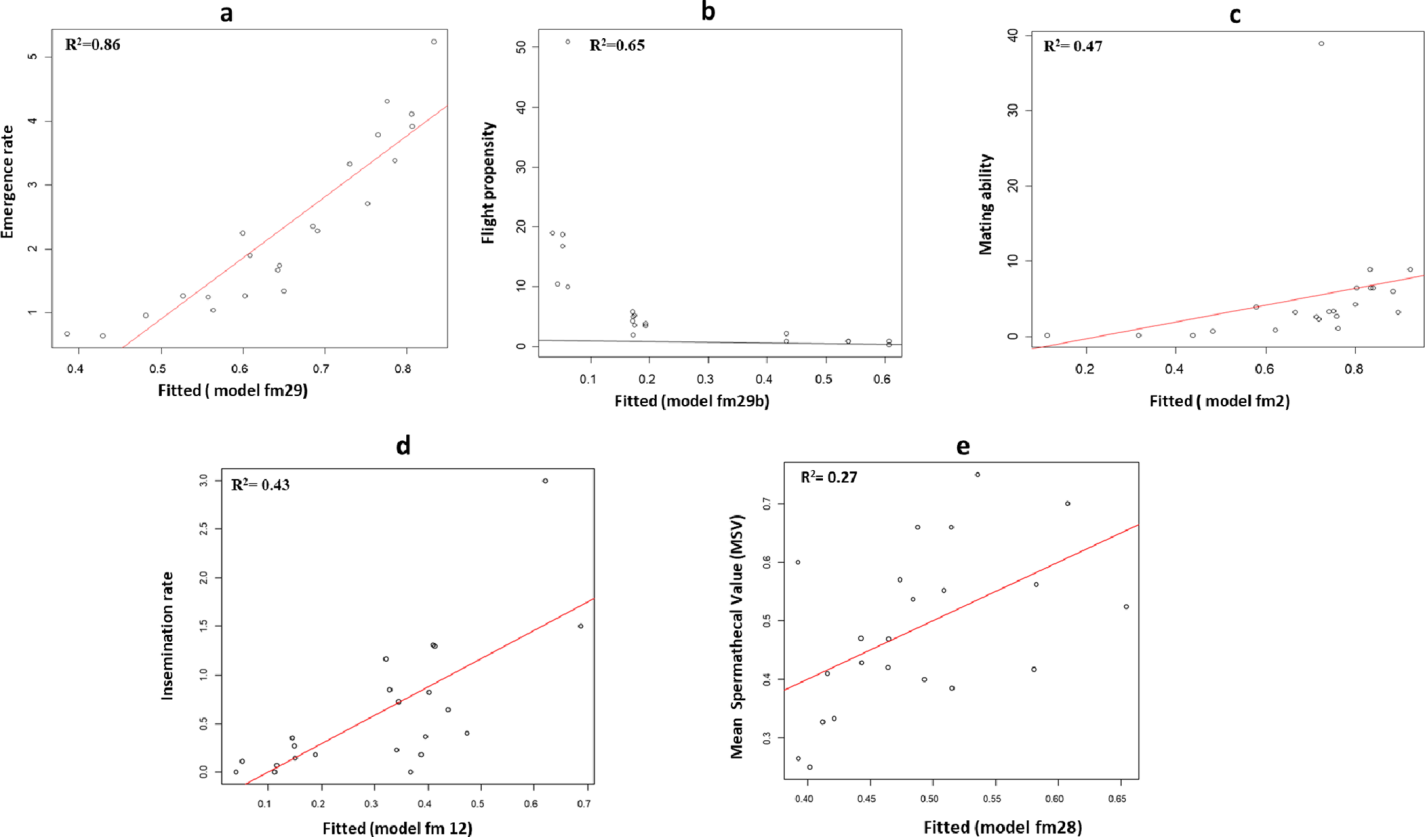
Figures of the fitting of models of shock with biological indicators of the collective 22- and 29-day groups. (a) Effect of shock on the emergence rate; (b) Effect of shock on the flight propensity; (c) Effect of shock on the mating ability; (d) Effect of shock on the insemination rate (full *versus* not full), (e) Effect of shock on the mean spermathecal value (MSV).

**Figure S4.**
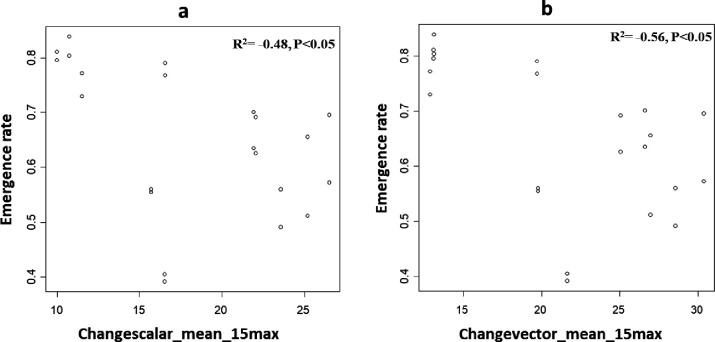
Impact of shock on the cumulative emergence rate of *Glossina morsitans morsitans* tsetse pupae that were transported at the age of 22 and 29 days. (a) Scatter plot showing the relationship between the changescalar_mean_15 max and the emergence rate, (b) Scatter plot showing the negative correlation between the changevector_mean_15 max and the emergence rate. Significant values are indicated by *p* < 0.05.

The impact of vibration and shock on the biological quality indicators of the transported pupae from the 22- and 29-day groups was evaluated and predicted using linear models. Modeling of the shock measurements (also known as characteristics/factors/variables of shock hereafter) against biological quality showed that the best model that explained the impact of shock events on emergence was the additive effect of irradiation and the means of change scalar and change vector (in all events) at the maximum acceleration threshold of 15 *g*, which all significantly contributed to the model (*z* = −2.698, *p* < 0.01; *z* = 7.921, *p* < 0.001; *z* = −9.307, *p* < 0.001, respectively, Table 2, File S2). The model (Fig. 9a) revealed a strong significant positive correlation (*r* = 0.90, *t* = 9.7903, df *= *20, *p* < 0.05) between the predicted and observed emergence rates and explained 86% (*R*^2 ^*= *0.86) of the variation observed in the emergence rate (File S2). In pairwise comparisons, the means of change-scalar and change-vector at the maximum acceleration of 15 *g*, each had a negative correlation with the emergence rates (*r* = −0.48, *t* = −2.4767, df *= *20, *p* < 0.05, and *r* = −0.56, *t* = −3.0538, df *= *20, *p* < 0.05, respectively, Figs. S4b, S4c, File S2).

**Figure S5.**
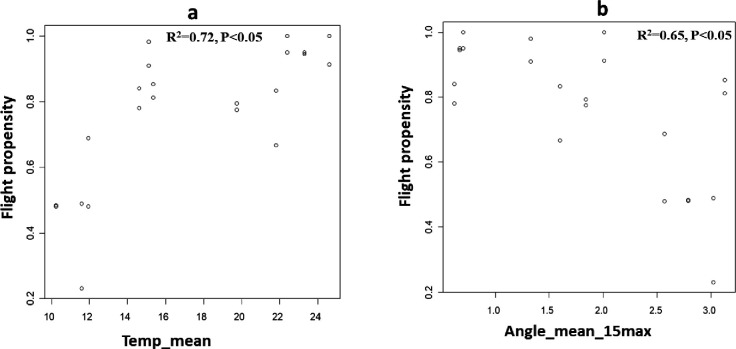
Impact of shock on the cumulative flight propensity of *Glossina morsitans morsitans* tsetse flies emerging from pupae that were transported at the age of 22 and 29 days. (a) Scatter plot of the correlation temperature and flight propensity, (b) Scatter plot of the correlation between the shock variable (Angle_mean_15 max) and the flight propensity. Significant values are indicated by *p* < 0.05.

The results also showed that the best model that explained the impact of shock events on flight propensity was the additive effect of the age and the mean temperature, which significantly contributed to the model (*z* = −2.384, *p* < 0.05; *z* = −3.345, *p* < 0.001, respectively), and also, the mean of the angles in an event at the maximum acceleration threshold of 15 *g* (Table 2, File S2). A fitting of the model (Fig. 9b) showed that there was a strong positive correlation between the predicted and observed flight propensity (*r* = −0.94, *t* = −13.302, df *= *20, *p* < 0.05, File S2) with the model explaining 65% (*R* ^2^ *= *0.65) of the observed variations. The mean temperature significantly increased the flight propensity in a pairwise comparison (*r* = 0.72, *t* = 4.7462, df *= *20, *p* < 0.05, Fig. S5b, File S2), while at the maximum acceleration threshold of 15 *g*, the means of the angles (from all shock events) had a significant negative correlation with flight ability (*r* = −0.65, *t* = −3.9286, df *= *20, *p* < 0.05, Fig. S5c, File S2).

**Figure S6.**
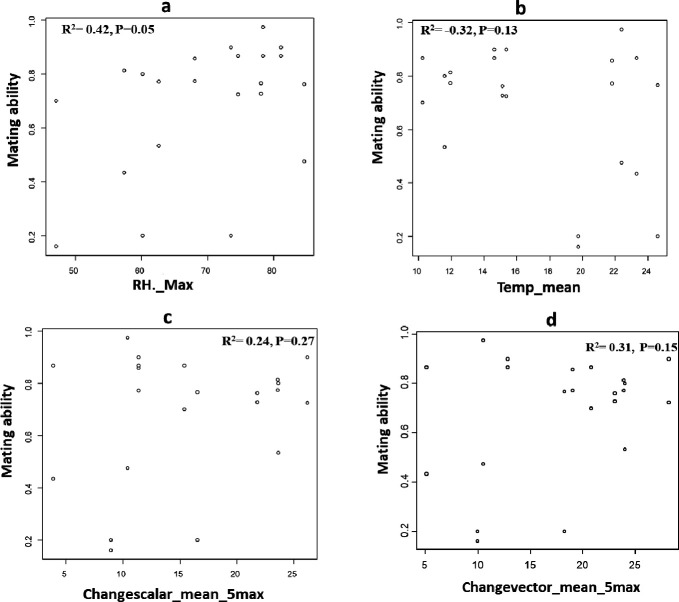
Impact of shock on the cumulative mating ability of *Glossina morsitans morsitans* tsetse flies emerging from pupae that were transported at the age of 22 and 29 days. (a) Scatter plot showing a significant positive correlation of the changescalar_mean_5max on the mating ability, (b) Scatter plot showing a significant positive correlation between the changevector_mean_5max and mating ability, (c) Scatter plot of the correlation between the relative humidity and the mating ability, (d) Scatter plot of the correlation between temperature and mating ability. Significant values are indicated by *p* < 0.05.

Models of the mating ability revealed that the best model describing the impact of shock events on the mating ability was the additive effect of irradiation, the maximum relative humidity, the mean temperature and the means of change scalar and change vector (from all events), at the maximum acceleration threshold of 5 *g* with a significant contribution from all (*z* = −3.897, *p* < 0.001; *z* = 3.042 , *p* < 0.01; *z* = −2.901, *p* < 0 .01; *z* = −4.714, *p* < 0.001; *z* = 4.649, *p* < 0.001, respectively, Table 2, models in File S2). However, the model showed that there was a weak correlation of the observed and predicted mating ability, although the model explained 59% (*R* ^2^ *= *0.59) of the observed variation (Fig. 9c, File S2). In pairwise comparisons, the relative humidity, the means of change_scalar and change_vector at the maximum acceleration of 5 *g* had a positive correlation, while the temperature had a negative correlation with the mating ability (Figs. S6b–S6e, File S2).

**Figure S7.**
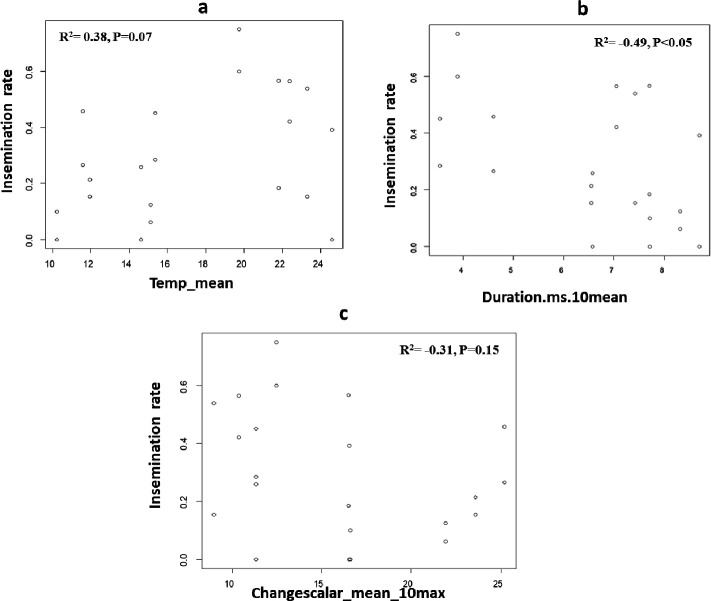
Impact of shock on the cumulative insemination rate of *Glossina morsitans morsitans* tsetse females mated with flies emerging from the pupae that were transported at the age of 22 and 29 days (a) Scatter plot showing the significant positive correlation between temperature and the observed insemination rate, (b) Scatter plot of the correlation between the duration.ms_10mean and the observed insemination rate, (c) Scatter plot of the correlation between the changescalar_mean_10max and the insemination rate. Significant values are indicated by *p* < 0.05.

The results of the insemination rate (full, not full) showed that the best model showing the impact of shock events on insemination rates was the additive effect of irradiation, the mean temperature, the duration at the mean acceleration threshold of 10 *g*, and the means of change of scalar (from all events), at the maximum acceleration threshold of 10 *g*, with all the factors significantly contributing (*z* = −1.259, *p* *=* 0.20810; *z* = 5.535, *p* < 0.001; *z* = −5.109, *p* < 0.001; *z* = 2.614, *p* < 0.01, respectively), to the model (Table 2, File S2). The predicted and observed values of insemination rates were strongly positively correlated (*r* = 0.71, *t* = 4.597, df *= *20, *p* < 0.05), with the model explaining 43% (*R* ^2^ *= *0.43) of the observed variations (Fig. 9d, File S2). In a pairwise comparison, only increased duration of shock events at the mean acceleration threshold of 10 *g* (Duration.ms. 10mean) was strongly negatively correlated (*r* = −0.49, *t* = −2.5455, df *= *20, *p* < 0.05) with the number of inseminated flies (Fig. S7c, File S2).

**Figure S8.**
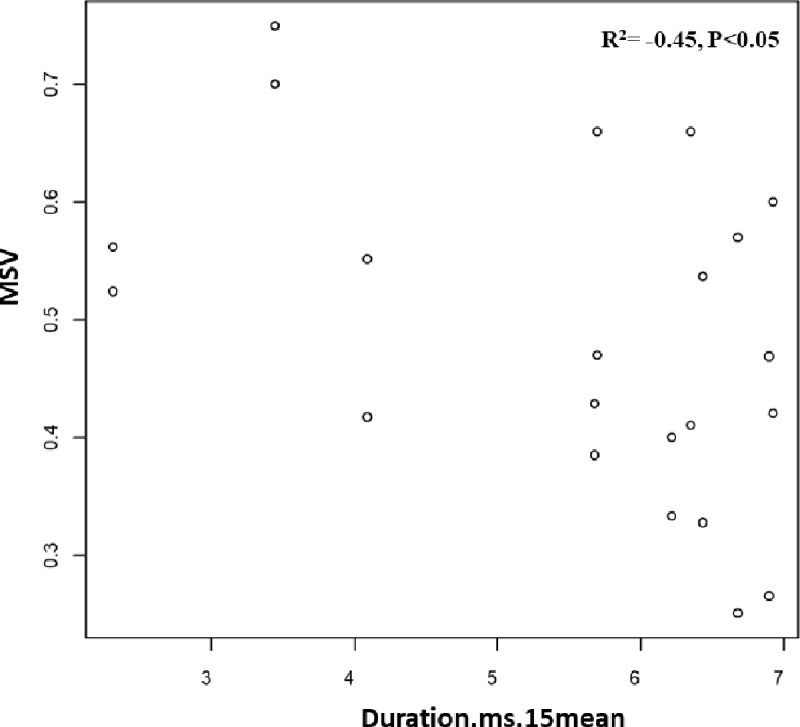
Impact of shock on the cumulative insemination rate of *Glossina morsitans morsitans* (*Gmm)* tsetse females mated with *Gmm* tsetse male flies from pupae transported at the age of 22 and 29 days. Scatter plot of the correlation between the Duration.ms.15mean and the MSV. Significant values are indicated by *p* < 0.05.

In addition to the insemination rate, an analysis of the impact of shock on the mean spermathecal value (MSV) revealed the best model as that with the additive effect of irradiation and duration in shock at the mean acceleration threshold of 15 *g* although these factors did not significantly contribute to the model (Table 2, models in File S2). A fitting of the model showed that there was a significant positive correlation between the observed and predicted values (*r* = 0.52, *t* = 2.754, df *= *20, *p* < 0.05, Fig. 9e, File S2) despite the model only explaining 27% (*R* ^2^ *= *0.27) of the observed variation in the mean spermathecal value of the transported flies. In a pairwise comparison, the duration of the shock events at the mean acceleration threshold of 15 *g* (Duration.ms.15mean) had a significant negative correlation with the MSV (*r* = −0.45, *t* = −2.2615, df *= *20, *p* < 0.05, Fig. S8a, File S2). A summary of the shock factors in models that significantly affected the biological quality of flies are displayed in Table 2.

The above models reveal that in general, the magnitude and direction of shock movements have a significant impact in reduction of the biological quality of the irradiated and transported tsetse flies. Also, the impact was negatively significant due to the synergistic effect of the addition of various shock parameters, even though some of them, such as temperature and humidity, increased the flight and mating ability of the flies, respectively.

## Discussion

This study aimed to optimize and validate a new protocol for the irradiation and transportation of 22-day-old pupae and compare this with the current method of transportation and irradiation of pupae at the age of 29 days, with the objective of improving the method for SIT programs. The impact of irradiation and transportation within age groups relative to their controls, between age groups, as well the impact when both age groups were combined was evaluated. Furthermore, the impact of shock on biological quality indicators during transportation was analyzed.

The sterilization results induced by irradiation treatment for the 22-day-old group are consistent with Langley and Curtis [38], who reported that the effect of irradiation on the reproductive capacity of male flies irradiated at various doses as pupae (at 25–27 days old) and at adult stages was similar, hence similar doses can be used across ages and stages. Their study also showed that pupae that have completed at least two-thirds of their development (e.g., 22 days and above), were less sensitive to detrimental effects of irradiation than earlier stages (8–10 days or 17–19 days) and can therefore withstand radiation for achievement of male sterility without significant mortality. However, this earlier observed lack of significant effect on mortality contradicts our findings where irradiation alone and in combination with transportation significantly reduced the survival rate of the 22-day-old group. Reduced survival has previously been observed after irradiation, where cells undergoing mitosis were more sensitive to irradiation, as the degenerated cells cannot be replaced [59]. Combined irradiation and transportation had a negative impact on all biological indicators except for the MSV. Irradiation alone negatively affected emergence, mating and survival, while transportation affected emergence, mating ability and insemination rates of the 22-day-old group of flies. Overall, the 22-day-old pupae were more negatively affected by irradiation and/or transportation relative to their controls, perhaps due to their young age as compared with 29-day-old pupae. The effect of irradiation may also have been more pronounced as the 22-day-old group had not been chilled, as other studies have suggested that while chilling reduces the quality of the flies compared to no irradiation, it also alleviates the impact of irradiation [46]. Dean and Wortham [19] found that pupae deposited in warmer months were more sensitive to radiation than those deposited in winter, agreeing with observations in our study of the sensitivity of 22-day-old group relative to its controls. It has also been reported in a previous study that sensitivity is age dependent and the earliest age at which pupae should be irradiated is 27 days, as this corresponds to 75 to 80% of pupal life [38].

In our study, the 29-day-old group was mainly affected by irradiation, which had a negative effect on the emergence rates, flight propensity and survival rates, although the longest living flies were also the only-irradiated ones, signaling the hormetic effect of irradiation on survival. According to many studies, irradiation reduces the quality of flies including the flight, survival and insemination rates [6, 29, 42, 53, 65]. Some studies have demonstrated that irradiation noticeably induced changes in the fine structure of the fibrillar flight muscle causing damage to the mitochondria of the flight muscle in flies irradiated with higher doses [5, 13]. Sperm quality and quantity can be altered with increasing radiation doses as irradiation causes nuclear changes in the germ cell preventing the zygote from reaching maturation (dominant lethal mutation). Additionally, sperm transfer and storage is linked to age, size, nutritional status and copula duration [10, 42, 59, 61], which may all have contributed to the observed insemination rates in this study. Combined irradiation and transportation, and transportation alone, also had a negative impact on survival rates over time, while all three treatments positively increased the insemination rates, and the mating ability was not affected by any of the treatments. The impact of long-distance transportation of 29-day-old pupae of *Glossina palpalis gambiensis* has been investigated in a similar study, showing that the percentage of emergence was reduced to 69%, while the flight propensity was even more dramatically reduced to 35%, corroborating our results on the negative impact of transportation [65], as also seen in several other studies [20, 52]. The impact of irradiation alone or transportation alone or their combination may have been reduced by chilling and/or the irradiation (considered as minor stress and sources of oxidative stress), which may have triggered the initial production of reactive oxygen species (ROS), which primed the fly body to produce antioxidants that were later protective against irradiation (in the case of chilling before irradiation) and transportation [15, 24].

The difference in proportions of emergence, flight, mating, insemination and survival rates between the 22- and 29-day-old groups may be attributed to the elimination of the handling steps of chilling, which was the major difference between the two groups. Chilling, as normally involved during the irradiation and transportation (packaging) of the late-stage pupae (29-day-old pupae), has been documented to have adverse effects on emergence, flight propensity, mating competitiveness, insemination and MSV and survival in previous studies [65, 47, 52, 43, 34, 20]. Diallo *et al*. [20] showed that the chilling period during transportation of pupae is a major factor in reducing the quality of flies, with a significant reduction in emergence. Interestingly, irradiation and/or transportation seemed to improve the mating and insemination rate as well as the MSV of the 29-day-old pupae, unlike the 22-day-old pupae, which concurs with studies conducted by Mutika *et al.* [45], who found better mating performance of males that emerged from pupae exposed to low temperature that were subsequently irradiated. Further, as previously mentioned, since irradiation and transportation can be sources of stress (oxidative stress), leading to production of ROS, low levels of irradiation may be advantageous, stimulating better reproductive performance, a phenomenon observed in other studies [2, 24, 62].

For both age groups, over the 100 day-period, transportation alone, and irradiation combined with transportation reduced the duration of survival of flies as compared to their controls. This finding contrasts with a previous study, which found no impact on survival, but there the flies were under starvation stress, unlike in this study where they were blood fed [20]. Irradiated flies from both groups lived the longest, with the 29-day-old irradiated flies living for more than 100 days, agreeing with the studies of Vreysen [73], who also found that *Glossina brevipalpis* flies that were irradiated with a dose between10 and 40 Gy had an increased lifespan. Flies from the 29-day-old group, from all treatments, survived longer (>60 days) than the 22-day-old group (<60 days). Additionally, the analysis of survival after 15, 30 and 60 days of both age groups showed that in the short term (<60 days), irradiation alone (and in combination with transportation) reduced the survival of the flies, but in the long term (>60 days), the impact of transportation (and in combination with irradiation) was more evident as shown by the analysis of the entire duration of the survival experiment. This informs SIT programs, which depend on the short-term survival of the fly, that it may be more important to minimize the impact of irradiation than that of transportation. This was further evident for the 29-day-old group where at 30 and 60 days, the previous irradiation of pupae was seen to decrease the survival rate of the flies.

Notwithstanding the age group, our results generally revealed that shock events together with the environmental conditions during transportation, individually and in combination, had an impact on the biological quality of flies. Emergence, which was the first determinant on the number of flies that would be evaluated, was negatively affected by the change in scalar and vector, revealing that high thresholds (>15 *g*) reduce emergence of transported flies. Other studies have shown that during transportation, vibration frequency and direction can damage the biological quality of the material [27, 28, 36, 48, 52, 65]. Remarkably, our results showed that during transportation, at the acceleration threshold of 5 *g*, a change in magnitude (scalar) or magnitude and direction (vector) had a positive impact on the mating ability. In addition to the previously mentioned increase in insemination rates and MSV in 29-day-old pupae, this effect may be a result of mechanical stimulation which has been observed to increase motility of sperm [63]. Minimal stress has been found to prime better immune responses in insects [2], which can be compared with the low mechanical stimulation and the resulting improved reproductive capacity in our study. A similar example is also in the signaling molecules (during low amplitude vibration) in human bones that play different roles in bone adaptation to high frequency loading [9]. However, as the threshold increased to 10 *g* and 15 *g* with changes in scalar and vector, a negative impact was observed in the insemination and emergence rates, respectively. Negative effects on spermatogenesis have been studied in humans and vibrations lead to oligospermia, azoospermia and reduction in sperm volume and motility [55]. Above 15 *g*, the angle (which determines the change vector), caused a decrease in flight propensity. This implies that while mild stress may be beneficial, increased stress is harmful. Increasing RH enhanced the mating ability of the flies, a result that could be attributed to the 29-day-old group, which had an average RH of 73.8 ± 1.1% during transportation, similar to the RH identified for optimum performance in mass rearing [7, 49]. The duration of shock events >10 *g* and >15 *g* affected the insemination rate and the MSV, respectively suggesting that the length of time in shock was detrimental at these thresholds. Surprisingly, at the acceleration threshold of 20 *g*, there was no evident impact on biological quality, perhaps due to the low number of shock events of this magnitude. This implies that it is not only the magnitude of the acceleration that matters, but also the number of events at that magnitude (or perhaps total duration of the events), in reducing biological quality. While the mechanisms by which vibration and shock events in our study affect emergence, flight, mating and insemination rates may not be clear, it is evident that vibration and shock events reduce the biological quality of the flies, especially the changes in vibration and shock magnitude and direction. The models of the effect of shock on the biological parameters indicate the projected impact of shock variables and the effect of their synergy, despite the non-significance of some of these predictors (shock variables) on their own.

In general, the results show that irradiation and transportation of pupae at an early age improves the performance of flies as there were higher proportions of flies emerging from the 22-day-old group. However, the conditions to which both early and late-stage pupae are subjected before emergence have a great impact on their biological quality and thus, it is necessary to minimize the effect of these processes and improve handling of the flies used in SIT programs for maximum benefits and results. Despite the limitation of our study, which was carried out at two distinct periods, given that more flies (in numbers/quantity) are able to emerge, take flight, mate, inseminate and survive from the transportation of younger pupae, we argue that transportation of 22-day-old pupae should be considered in SIT programs. Furthermore, with the elimination of chilling, the expenses and urgency associated with the transportation of older pupae (>29 days, which are ready to emerge), especially in the case of delayed delivery of packages to destination countries, will no longer be of consequence as there will be sufficient time for handling the flies before emergence.

## Conclusion

To our knowledge, this is the first study to investigate the relative impact of early age irradiation and transportation on emergence, flight propensity, mating ability, insemination and survival, in tsetse species and specifically *G. morsitans morsitans*. It is also the first detailed analysis of the impact of vibration/shock on tsetse flies, where we break down the vibration and shock characteristics and analyze their impact on biological quality. As hypothesized, we demonstrate that irradiation of pupae at an early age (22 days) reduces the negative impact on the quality of sterile males as it eliminates the adverse impact of chilling. As SIT requires large numbers of flies to be released to overflood the wild population, the elimination of chilling, which results in the higher proportions of emerged flies as revealed by this study, will cascade downwards to higher proportions of flight, mating and the MSV, considerably reducing the costs of SIT. We hope that our results will be informative in making decisions regarding the irradiation and transportation of pupae at an early age and on the use of the NIRPSS, i.e., if the pupae can be irradiated and transported immediately after separation at the early stage (at least 22 days), or separated to wait for maturity (at least 29 days) before irradiation and transportation for the improvement of SIT in tsetse suppression and eradication efforts. It will also be prudent to limit shock during transportation by improving the packaging of pupae to minimize the differences in scalars and vectors during transportation as such movement reduces the quality of emerging flies. Other methods such as the use of hypoxia to reduce the production of reactive oxygen species (ROS) during irradiation and transportation may be explored to maintain or improve the quality of the flies. Future studies may further evaluate the effect of shock and use this information to further reduce the negative impact of irradiation and transportation on the 22- day- old pupae that has been demonstrated in this study.

## Data Availability

Materials described in the paper, including all relevant raw data, are available under reference [84] at https://doi.org/10.7910/DVN/DQ3HG3.

## Supplementary material

The supplementary material of this article is available at https://www.parasite-journal.org/10.1051/parasite/2024068/olm.

File S1.
Shock data processing instructions. A file describing the steps followed to process the raw vibration data from the MSR 165 data logger resulting in a final output of only shock data above the threshold values of 5 *g*, 10 *g*, 15 *g* and 20 *g*. The data were further processed using macros to make summaries of statistics (maximum, means, minimum) of shock variables from the processed shock.
File S2.
Output of the R-markdown code file of all the analyses performed using the R and R-studio software.
File S3.
Codes of all the analyses performed in R and R-studio summarized in R-markdown.
