# Supplementary material for: Rearing of Glossina morsitans morsitans tsetse flies for the sterile insect technique: evaluating the impact of irradiation and transportation during early and late-stage pupal development on the quality of emerging adults
Source: Parasite. 2024 Nov 21;31:73. doi: 10.1051/parasite/2024068 (PMC11583636; doi:10.1051/parasite/2024068)
Supplement: Supplementary file 1 — Shock data processing instructions. A file describing the steps followed to process the raw vibration data from the MSR 165 data logger resulting in a final output of only shock data above the threshold values of 5 g, 10 g, 15 g and 20 g. The data were further processed using macros to make summaries of statistics (maximum, means, minimum) of shock variables from the processed shock. [file parasite-31-73-s1.pdf]

Shock data processing

The conventions used below are as follows:

- The `Courier font` indicates things you see in the terminal window or must type into the terminal window.
- Items between angle brackets <> indicate a key to be pressed, e.g. `<Entre>` means press the Enter key.
- *Italics* indicate where you have to supply information, e.g. *`filename`* means you must enter the name of the file to be used.

1. Export msr data to csv

a) Using the MSR V6.04.00 software, open the `msr2csv` program.

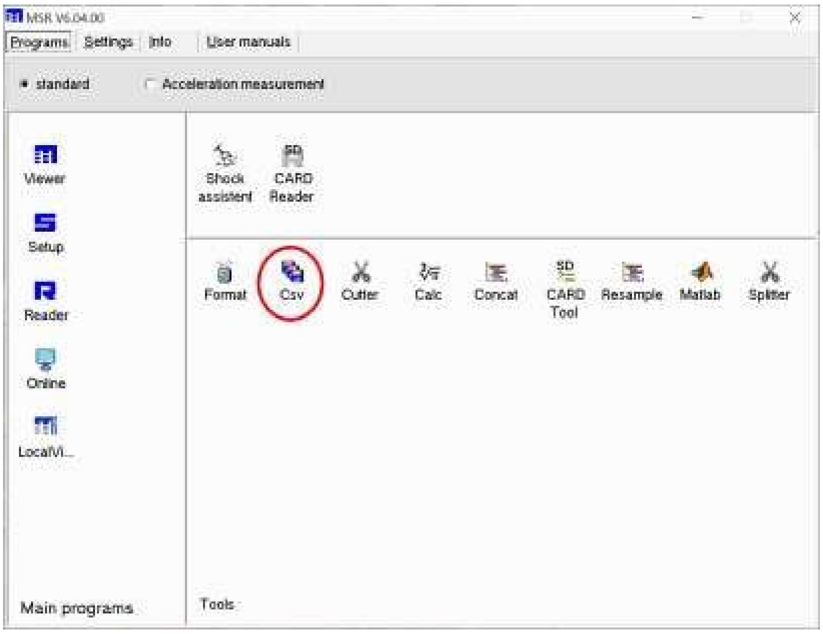

b) Select **`Number format`** `0.000` and **`Column for date/time`** `3 columns date/time/ms`

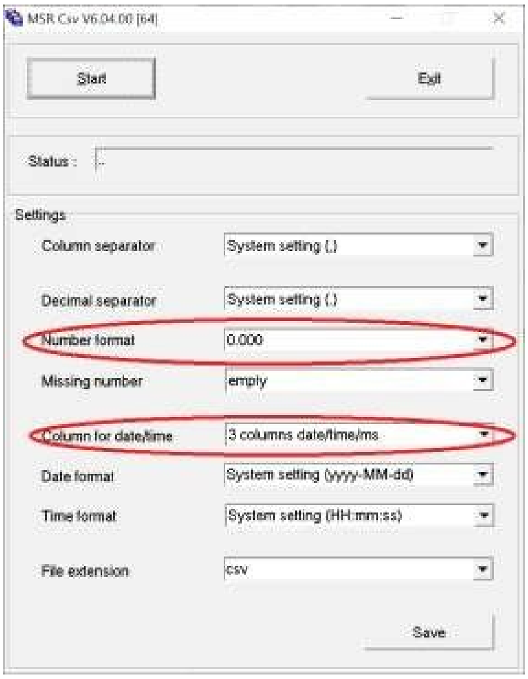

c) Click Start and select the first MSR file to export. Repeat for each MSR file

d) Save the resultant .csv files in a convenient folder in the root of the C:\ drive (e.g. C:\temp, or if this already exists C:\temp\MSR).

2. Separate the data in acceleration and temperature/humidity

[A copy of the terminal window appears below.]

a) Copy the files separate.bat, filter.bat, filter2.exe and sed.exe to this new folder.

b) Open a terminal window by pressing the Windows key followed by cmd and <Enter>.

c) Change to the folder in which the files are stored by typing cd C:\temp\MSR (or cd C:\temp) and press <Enter>. The command line prompt changes to C:\temp\MSR to show in which folder you are located.

d) You can check the files in the folder by typing dir then <Enter>. You can add wild cards to the dir command to see only certain files, e.g. dir MSR* will show only files starting MSR.

e) Type separate *filename* <Enter>. The *filename* is **WITHOUT** the .csv at the end.

f) This routine produces two files, one of the temperature data ending _temp.csv and one of the acceleration data ending _acc.csv.

3. Filter the data

a) Type filter *acceleration_filename threshold before after* <Entre>. Again, this *filename* is WITHOUT the .csv at the end. Threshold is the value to be exceeded for the record to be included in the output file; before is the number of records before the threshold is exceeded that are also output to the file; after is the number of lines after the value falls back below threshold output to the file. Together this constitutes an event.

b) This produces two files. The first file will be produced with the ending _threshold_before_after.csv.

c) One blank line is placed after each event.

d) This output file has the fields Date, Time, ms, X, Y, Z, scalar. There are no header labels, these need to be added in Excel. Scalar is the magnitude of the vector, calculated as sqrt(x^2+y^2+z^2).

e) A second file with the ending _threshold_before_after_count.txt is a simple text file with just two lines, the total number of lines in the csv file and the number of events. **Note** that the first number is the number of records **PLUS** the number of events as there is one blank line for each event.

4. Post processing

a) Open the final csv file in Excel.

b) Save the file in xlsx format.

c) Add a blank row at the top and enter the headings:

Date	Time	ms	X	Y	Z	Scalar	-	Change scalar	Change vector	Angle	Hz

d) Note that there is one blank column between Scalar and Change scalar

e) In the column with the blank heading, starting in row 2 insert 1, 2, etc. down the column to the last entry in the file. This is only used to help copy formulae in the sheet.

f) In the column `Change scalar`, in row 3, enter the formula

g) =IF(OR(G3="",G2=""),"",ABS(G3-G2))

This is the simple change in the magnitude of the two successive scalars without taking account of the change in angle.

h) In the column `Change vector` in row 3 enter the formula

=IF(OR(G2=0,G3=0),"",SQRT((D2-D3)^2+(E2-E3)^2+(F2-F3)^2))

This is the magnitude of the resultant vector between the two vectors taking into account the angle change.

i) In the column `Angle` enter the formula:

=IF(OR(G2=0,G3=0),"",SQRT((D2-D3)^2+(E2-E3)^2+(F2-F3)^2))

This is the angle between the two successive vectors. This allows us to calculate the frequency of the vibration.

j) In the column `Hz` enter the formula:

=IF(K3="","",K3/(2*PI())*1600)

This is an approximation to the vibration frequency in Hz. As the recorder takes 1600 measurements per second it cannot measure frequencies above 800 Hz.

k) Select the range I3:L3 and copy down the complete sheet by placing the mouse on the small black block at the bottom right corner and double clicking.

5. Running the Macros

a) Save the csv file into an Excel Macro-Enabled Workbook(.xlsm)

b) To the next column M add the value of the acceleration threshold from which the data in the file is derived

c) On the macro enabled(.xlsm), select the view menu on the to select the Macros button. In the dialogue box, enter the name "length" (without quotes) in the top left box and click on '**Create**'.

d) When the VBA editor opens, delete everything and paste the macro (**S2_File.txt**) and run .

The cells from in row one from N to AA will automatically fill in with the following headers:

Event, Length, Length-, Duration(ms), scalar_max, scalar_mean, change_scalar_max, change_scalar_mean, Change_vector_max, Change_vector_mean, Angle_max, Angle_mean, Hz_max, Hz_mean,

"**Event**" is an occasion when the acceleration exceeded the threshold set in the analysis, e.g. 20 g.

"**Length**" is the number of times the acceleration exceeds the threshold in the analysis(no. of rows)

"**Length**-" is the number of times the acceleration exceeds the threshold in the analysis(no. of rows) excluding 'before' which is the number of records (in this case '**4**') before the threshold

value is exceeded that are also output to the file, and ‘after’ being the number of records(in this case ‘**4**’) after the value falls back below threshold as shown in the output to the file
“**Duration(ms)** ” is how long an event lasts in milliseconds
“**scalar_max**” is the maximum value of a scalar within an event
“**scalar_mean**” is the mean value of a scalar within an event
“**change_scalar_max**” is the maximum value of change scalar within an event
“**change_scalar_mean**” is the mean value of a change scalar within an event
“**Change_vector_max**” is the maximum value of change vector within an event
“**Change_vector_mean**” is the mean value of change vector within an event
“**Angle_max**” is the maximum value of an angle within an event
“**Angle_mean**” is the mean value of an angle within an event
“**Hz_max**” is the maximum value of the frequency of an event
“**Hz_mean**” is the mean value of the frequency of an event

e) Keep the first workbook open and run the macro in the next workbooks without having to create it again. You may also repeat the same for the other workbooks.

   The values of each header will be generated automatically and you may proceed with general summaries and statistics.

Command Prompt

```
Microsoft Windows [Version 10.0.18362.836]
(c) 2019 Microsoft Corporation. All rights reserved.

C:\Users\Andrew>cd C:\temp\MSR

C:\temp\MSR>dir
 Volume in drive C is Boot
 Volume Serial Number is EA5D-A3FD

 Directory of C:\temp\MSR

2020-05-24  17:37    <DIR>          .
2020-05-24  17:37    <DIR>          ..
2020-05-24  17:34             1,044 filter.bat
2020-05-23  21:36            57,849 filter2.exe
2020-05-22  13:39        59,949,905 MSR451012_170510_184903.csv
2020-05-24  17:08         6,570,475 MSR451012_170510_184903_acc_4_8_16.xlsx
2020-05-22  17:36        10,063,896 MSR451012_170517_100000.csv
2020-05-22  17:26        22,073,252 MSR451012_170524_094900.csv
2020-05-22  17:14         4,584,474 MSR451012_180620_125000.csv
2020-05-22  17:15        17,314,867 MSR451012_180627_144500.csv
2005-02-11  02:14            98,816 sed.exe
2020-05-24  12:59               433 separate.bat
              10 File(s)    120,715,011 bytes
               2 Dir(s)  180,760,043,520 bytes free

C:\temp\MSR>separate MSR451012_170510_184903
C:\temp\MSR>filter MSR451012_170510_184903_acc 4 4 8
Threshold = 4.000000 Before = 4 After = 8
C:\temp\MSR>dir
 Volume in drive C is Boot
 Volume Serial Number is EA5D-A3FD

 Directory of C:\temp\MSR

2020-05-24  17:42    <DIR>          .
2020-05-24  17:42    <DIR>          ..
2020-05-24  17:34             1,044 filter.bat
2020-05-23  21:36            57,849 filter2.exe
2020-05-22  13:39        59,949,905 MSR451012_170510_184903.csv
2020-05-24  17:39        53,973,172 MSR451012_170510_184903_acc.csv
2020-05-24  17:42         1,437,812 MSR451012_170510_184903_acc_4_4_8.csv
2020-05-24  17:42                64 MSR451012_170510_184903_acc_4_4_8_count.txt
2020-05-24  17:08         6,570,475 MSR451012_170510_184903_acc_4_8_16.xlsx
2020-05-24  17:39           115,638 MSR451012_170510_184903_temp.csv
2020-05-22  17:36        10,063,896 MSR451012_170517_100000.csv
2020-05-22  17:26        22,073,252 MSR451012_170524_094900.csv
2020-05-22  17:14         4,584,474 MSR451012_180620_125000.csv
2020-05-22  17:15        17,314,867 MSR451012_180627_144500.csv
2005-02-11  02:14            98,816 sed.exe
2020-05-24  12:59               433 separate.bat
              14 File(s)    176,241,697 bytes
               2 Dir(s)  180,684,349,440 bytes free

C:\temp\MSR>
```
