# Supplementary material for: Rearing of Glossina morsitans morsitans tsetse flies for the sterile insect technique: evaluating the impact of irradiation and transportation during early and late-stage pupal development on the quality of emerging adults
Source: Parasite. 2024 Nov 21;31:73. doi: 10.1051/parasite/2024068 (PMC11583636; doi:10.1051/parasite/2024068)
Supplement: Supplementary file 2 — Output of the R-markdown code file of all the analyses performed using the R and R-studio software. [file parasite-31-73-s2.pdf]

F— title: “All analysis_Impact of irradiation and transportation on G. morsitans morsitans” author: “Mirieri et al.,” date: “12-06-2024” output: word_document —

Load library

```
library(datasets)
library(gcookbook)
library(ggplot2)
library(plyr)
library(dplyr)

##
## Attaching package: 'dplyr'

## The following objects are masked from 'package:plyr':
##
##     arrange, count, desc, failwith, id, mutate, rename, summarise,
##     summarize

## The following objects are masked from 'package:stats':
##
##     filter, lag

## The following objects are masked from 'package:base':
##
##     intersect, setdiff, setequal, union

library(lattice)
library(MASS)

##
## Attaching package: 'MASS'

## The following object is masked from 'package:dplyr':
##
##     select

library(rcompanion)
library(survival)
library(ranger)
library(ggfortify)
library(rmarkdown)
library(knitr)
library(coxme)

## Warning: package 'coxme' was built under R version 4.3.2

## Loading required package: bdsmatrix

##
## Attaching package: 'bdsmatrix'
```

```
## The following object is masked from 'package:base':
##
##     backsolve
```

```
## Graphics
library(lattice)
library(tidyverse)
```

```
## ── Attaching core tidyverse packages ──────────────────────── tidyverse
2.0.0 ──
## ✔ forcats   1.0.0     ✔ stringr   1.5.0
## ✔ lubridate 1.9.2     ✔ tibble    3.2.1
## ✔ purrr     1.0.1     ✔ tidyr     1.3.0
## ✔ readr     2.1.4
```

```
## ── Conflicts ──────────────────────────────────────────
tidyverse_conflicts() ──
## ✖ dplyr::arrange()   masks plyr::arrange()
## ✖ purrr::compact()   masks plyr::compact()
## ✖ dplyr::count()     masks plyr::count()
## ✖ dplyr::desc()      masks plyr::desc()
## ✖ dplyr::failwith()  masks plyr::failwith()
## ✖ dplyr::filter()    masks stats::filter()
## ✖ dplyr::id()        masks plyr::id()
## ✖ dplyr::lag()       masks stats::lag()
## ✖ dplyr::mutate()    masks plyr::mutate()
## ✖ dplyr::rename()    masks plyr::rename()
## ✖ MASS::select()     masks dplyr::select()
## ✖ dplyr::summarise() masks plyr::summarise()
## ✖ dplyr::summarize() masks plyr::summarize()
## ℹ Use the conflicted package (<http://conflicted.r-lib.org/>) to force all
conflicts to become errors
```

```
library(gapminder)
```

```
## Warning: package 'gapminder' was built under R version 4.3.2
```

```
library(FSA)
```

```
## ## FSA v0.9.4. See citation('FSA') if used in publication.
## ## Run fishR() for related website and fishR('IFAR') for related book.
##
## Attaching package: 'FSA'
##
## The following object is masked from 'package:plyr':
##
##     mapvalues
```

```
library(stats)
library(RCA)
```

```
## Loading required package: igraph
##
## Attaching package: 'igraph'
##
## The following objects are masked from 'package:lubridate':
##
##     %--%, union
##
## The following objects are masked from 'package:purrr':
##
##     compose, simplify
##
## The following object is masked from 'package:tidyr':
##
##     crossing
##
## The following object is masked from 'package:tibble':
##
##     as_data_frame
##
## The following objects are masked from 'package:dplyr':
##
##     as_data_frame, groups, union
##
## The following objects are masked from 'package:stats':
##
##     decompose, spectrum
##
## The following object is masked from 'package:base':
##
##     union
##
## Loading required package: gplots
## Registered S3 method overwritten by 'gplots':
##   method         from
##   reorder.factor DescTools
##
## Attaching package: 'gplots'
##
## The following object is masked from 'package:stats':
##
##     lowess
```

```
#library(broom)
library(sp)
```

```
## Warning: package 'sp' was built under R version 4.3.2
```

```
library(ggpubr)
```

```
## Registered S3 methods overwritten by 'car':
##   method       from
##   hist.boot    FSA
##   confint.boot FSA
##
## Attaching package: 'ggpubr'
##
## The following object is masked from 'package:plyr':
##
##     mutate
```

```
library(AICcmodavg)
```

```
## Warning: package 'AICcmodavg' was built under R version 4.3.2
```

```
##
## Attaching package: 'AICcmodavg'
##
## The following object is masked from 'package:ranger':
##
##     importance
```

```
library(car)
```

```
## Loading required package: carData
##
## Attaching package: 'car'
##
## The following object is masked from 'package:FSA':
##
##     bootCase
##
## The following object is masked from 'package:purrr':
##
##     some
##
## The following object is masked from 'package:dplyr':
##
##     recode
```

```
library(ggthemes)
## Mixed generalized linear models
library(lme4)
```

```
## Loading required package: Matrix
##
## Attaching package: 'Matrix'
##
## The following objects are masked from 'package:tidyr':
##
##     expand, pack, unpack
```

```
##
##
## Attaching package: 'lme4'
##
## The following object is masked from 'package:AICcmodavg':
##
##     checkConv
```

```
library(MuMIn)
```

```
## Registered S3 methods overwritten by 'MuMIn':
##   method        from
##   formula.coxme coxme
##   logLik.coxme  coxme
##   logLik.lmekin coxme
##
## Attaching package: 'MuMIn'
##
## The following objects are masked from 'package:AICcmodavg':
##
##     AICc, DIC, importance
##
## The following object is masked from 'package:ranger':
##
##     importance
```

```
library(nlme)
```

```
##
## Attaching package: 'nlme'
##
## The following object is masked from 'package:lme4':
##
##     lmList
##
## The following object is masked from 'package:dplyr':
##
##     collapse
```

```
library(survminer)
```

```
## Warning: package 'survminer' was built under R version 4.3.2
```

```
##
## Attaching package: 'survminer'
##
## The following object is masked from 'package:survival':
##
##     myeloma
```

Working directory

ANALYSIS OF THE IMPACT OF IRRADIATION AND TRASPORATION(22-day old only/29-day old only)

MAIN FIGURES (MANUSCRIPT FIGURES)

Figure 1:Emergence rate(22 and 29 days)-differences between ages within each treatment

```
tab1= read.csv("Figure 1.csv")
head(tab1)
```

```
##   Replicate Pupal_age      Treatments emerged unemerged shipped irradiated
## 1        R1        29  Shipped-110Gy      81        36       1          1
## 2        R1        29    Shipped-0Gy     102        61       1          0
## 3        R1        29 Unshipped-110Gy     85        36       0          1
## 4        R1        29   Unshipped-0Gy     116        47       0          0
## 5        R2        29  Shipped-110Gy      85        81       1          1
## 6        R2        29    Shipped-0Gy     120        63       1          0
##   chilled   Treatment_age         Treatment
## 1       1  Ship-110Gy _22  4_Shipped-110Gy
## 2       1    Ship-0Gy_22      2_Shipped-0Gy
## 3       1 Unship-110Gy_22 3_Unshipped-110Gy
## 4       1   Unship-0Gy_22    1_Unshipped-0Gy
## 5       1  Ship-110Gy _22  4_Shipped-110Gy
## 6       1    Ship-0Gy_22      2_Shipped-0Gy
```

```
Emerg_rate<- tab1$emerged / (tab1$unemerged+ tab1$emerged)
tab1$Pupal_age<- as.factor(tab1$Pupal_age)


Figure_1<-ggplot(tab1, aes(x=factor(Treatments),y=Emerg_rate,
colour=Pupal_age)) +
  geom_boxplot(position=position_dodge(0.8))+
  geom_jitter(position=position_dodge(0.8))


Figure_1
```

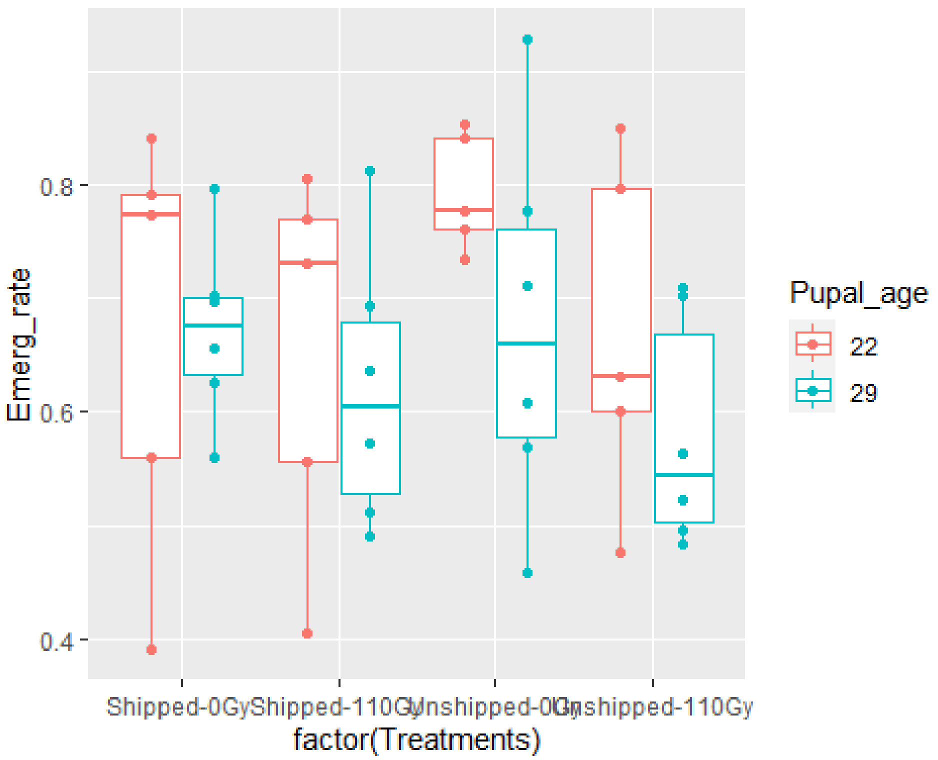


```
tiff("Figure 1.tiff", width = 7, height = 4, units = 'in',compression =
'lzw',res = 300)
plot(Figure_1+theme_tufte() + theme(axis.line = element_line(size = 1, colour
= "black")) + theme(legend.title = element_text(face = "bold")) +
theme(legend.text = element_text(face = "italic"))) + theme(axis.text.x =
element_text(colour = "black")) + theme(axis.text.y = element_text(colour =
"black")) + xlab(expression(bold("Treatments"))) +
ylab(expression(bold("Emergence rate")))

## Warning: The `size` argument of `element_line()` is deprecated as of
ggplot2 3.4.0.
## ℹ Please use the `linewidth` argument instead.
## This warning is displayed once every 8 hours.
## Call `lifecycle::last_lifecycle_warnings()` to see where this warning was
## generated.

dev.off()

## png
##   2

#### Significance of treatments


##22 days

Age22_fig1 <- subset(tab1, Pupal_age== "22")
```

```
head(Age22_fig1)

##    Replicate Pupal_age      Treatments emerged unemerged shipped
irradiated
## 25        R1        22  Shipped-110Gy     125       100       1
1
## 26        R1        22    Shipped-0Gy     126        99       1
0
## 27        R1        22 Unshipped-110Gy     135        90       0
1
## 28        R1        22   Unshipped-0Gy     171        54       0
0
## 29        R2        22  Shipped-110Gy      91       134       1
1
## 30        R2        22    Shipped-0Gy      88       137       1
0
##    chilled   Treatment_age          Treatment
## 25       0   Ship-110Gy_29  4_Shipped-110Gy
## 26       0    Ship-0Gy _29     2_Shipped-0Gy
## 27       0 Unship-110Gy-29 3_Unshipped-110Gy
## 28       0   Unship-0Gy-29   1_Unshipped-0Gy
## 29       0   Ship-110Gy_29  4_Shipped-110Gy
## 30       0    Ship-0Gy _29     2_Shipped-0Gy

Age22_fig1$rate <- Age22_fig1$emerged/ (Age22_fig1$unemerged+
Age22_fig1$emerged)
fm1_22 <- glmer(cbind(emerged, unemerged) ~ Treatment +(1|Replicate), family
= binomial, data =Age22_fig1)
summary(fm1_22)

## Generalized linear mixed model fit by maximum likelihood (Laplace
##   Approximation) [glmerMod]
##  Family: binomial  ( logit )
## Formula: cbind(emerged, unemerged) ~ Treatment + (1 | Replicate)
##    Data: Age22_fig1
##
##      AIC      BIC   logLik deviance df.resid
##    254.9    259.8   -122.4    244.9       15
##
## Scaled residuals:
##    Min     1Q Median     3Q    Max
## -5.924 -1.041 -0.136  1.623  3.763
##
## Random effects:
##  Groups    Name        Variance Std.Dev.
##  Replicate (Intercept) 0.2946   0.5428
## Number of obs: 20, groups:  Replicate, 5
##
```

```
## Fixed effects:
##                            Estimate Std. Error z value Pr(>|z|)
## (Intercept)                 1.42483    0.25424   5.604 2.09e-08 ***
## Treatment2_Shipped-0Gy     -0.65674    0.09923  -6.618 3.63e-11 ***
## Treatment3_Unshipped-110Gy -0.67367    0.09913  -6.796 1.08e-11 ***
## Treatment4_Shipped-110Gy   -0.74468    0.09873  -7.542 4.61e-14 ***
## ---
## Signif. codes:  0 '***' 0.001 '**' 0.01 '*' 0.05 '.' 0.1 ' ' 1
##
## Correlation of Fixed Effects:
##             (Intr) T2_S-0 T3_U-1
## Trtmn2_S-0G -0.223
## Trt3_U-110G -0.224  0.570
## Trt4_S-110G -0.225  0.573  0.573
```

```
##29 days

Age29_fig1 <- subset(tab1, Pupal_age== "29")

head(Age29_fig1 )
```

```
##   Replicate Pupal_age      Treatments emerged unemerged shipped irradiated
## 1        R1        29   Shipped-110Gy      81        36       1          1
## 2        R1        29     Shipped-0Gy     102        61       1          0
## 3        R1        29 Unshipped-110Gy      85        36       0          1
## 4        R1        29   Unshipped-0Gy     116        47       0          0
## 5        R2        29   Shipped-110Gy      85        81       1          1
## 6        R2        29     Shipped-0Gy     120        63       1          0
##   chilled   Treatment_age          Treatment
## 1       1  Ship-110Gy _22  4_Shipped-110Gy
## 2       1    Ship-0Gy_22      2_Shipped-0Gy
## 3       1 Unship-110Gy_22 3_Unshipped-110Gy
## 4       1   Unship-0Gy_22    1_Unshipped-0Gy
## 5       1  Ship-110Gy _22  4_Shipped-110Gy
## 6       1    Ship-0Gy_22      2_Shipped-0Gy
```

```
Age29_fig1 $rate <- Age29_fig1 $emerged/ (Age29_fig1 $unemerged+ Age29_fig1
$emerged)
fm1_29 <- glmer(cbind(emerged, unemerged) ~ Treatment +(1|Replicate), family
= binomial, data = Age29_fig1 )
summary(fm1_29)
```

```
## Generalized linear mixed model fit by maximum likelihood (Laplace
##   Approximation) [glmerMod]
##  Family: binomial  ( logit )
## Formula: cbind(emerged, unemerged) ~ Treatment + (1 | Replicate)
##    Data: Age29_fig1
##
##      AIC      BIC   logLik deviance df.resid
##    227.9    233.8   -109.0    217.9       19
##
```

```
## Scaled residuals: 
##     Min      1Q  Median      3Q     Max 
## -2.9669 -0.4804 -0.0765  0.8728  4.7710 
## 
## Random effects:
##  Groups    Name        Variance Std.Dev.
##  Replicate (Intercept) 0.1474   0.3839  
## Number of obs: 24, groups:  Replicate, 6
## 
## Fixed effects:
##                            Estimate Std. Error z value Pr(>|z|)    
## (Intercept)                 0.65708    0.17274   3.804 0.000142 ***
## Treatment2_Shipped-0Gy      0.11449    0.09964   1.149 0.250553    
## Treatment3_Unshipped-110Gy -0.30747    0.10153  -3.028 0.002458 ** 
## Treatment4_Shipped-110Gy   -0.16945    0.10157  -1.668 0.095250 .  
## ---
## Signif. codes:  0 '***' 0.001 '**' 0.01 '*' 0.05 '.' 0.1 ' ' 1
## 
## Correlation of Fixed Effects:
##             (Intr) T2_S-0 T3_U-1
## Trtmn2_S-0G -0.298              
## Trt3_U-110G -0.296  0.510       
## Trt4_S-110G -0.294  0.510  0.502
```

Figure 2:Flight propensity(22 and 29 days)-differences between ages within each treatment

```
##"flight propensity combined data
tab2= read.csv("Figure 2.csv")
head(tab2)

##   Replicate Pupal_age      Treatments out in. shipped irradiated chilled
## 1        R1        29  Shipped-110Gy  31  33       1          1       1
## 2        R1        29    Shipped-0Gy  26  28       1          0       1
## 3        R1        29 Unshipped-110Gy  30  42       0          1       1
## 4        R1        29   Unshipped-0Gy  21   9       0          0       1
## 5        R2        29  Shipped-110Gy  23  24       1          1       1
## 6        R2        29    Shipped-0Gy   9  30       1          0       1
##     Treatment_age         Treatment
## 1  Ship-110Gy _22  4_Shipped-110Gy
## 2    Ship-0Gy_22      2_Shipped-0Gy
## 3 Unship-110Gy_22 3_Unshipped-110Gy
## 4   Unship-0Gy_22    1_Unshipped-0Gy
## 5  Ship-110Gy _22  4_Shipped-110Gy
## 6    Ship-0Gy_22      2_Shipped-0Gy

Flight_rate<- tab2$out / (tab2$in.+ tab2$out)
tab2$Pupal_age<- as.factor(tab2$Pupal_age)

Figure_2<-ggplot(tab2, aes(x=Treatments,y=Flight_rate, colour=Pupal_age)) +
  geom_boxplot(position=position_dodge(0.8))+
```

```
  geom_jitter(position=position_dodge(0.8))
Figure_2
```

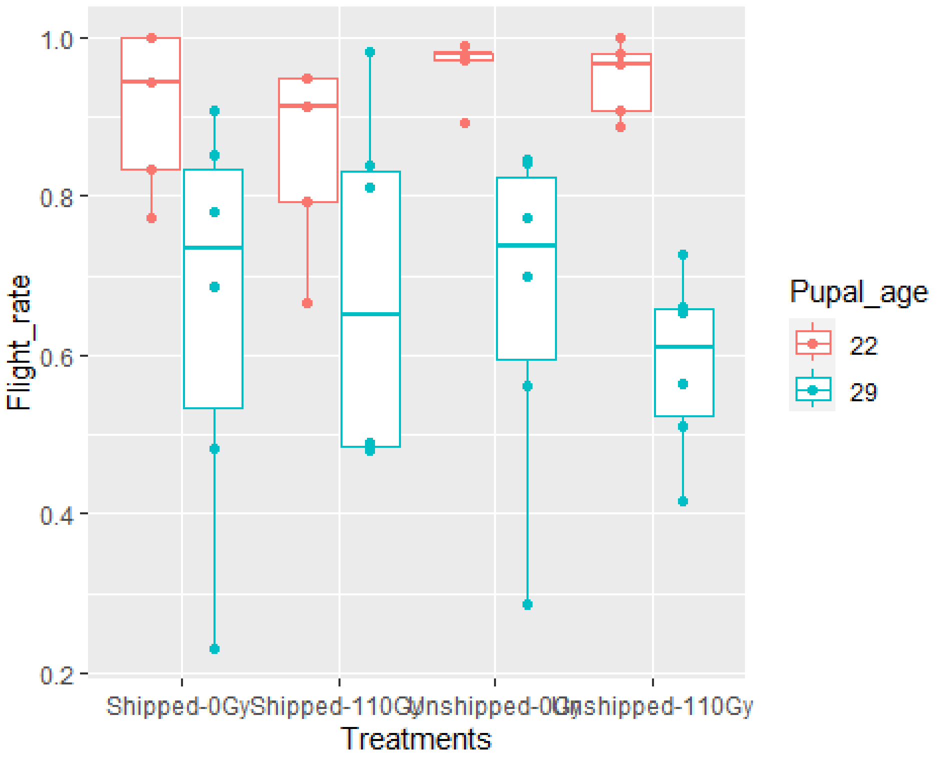


```
tiff("Figure 2.tiff", width = 7, height = 4, units = 'in',compression =
'lzw',res = 300)
plot(Figure_2+theme_tufte() + theme(axis.line = element_line(size = 1, colour
= "black")) + theme(legend.title = element_text(face = "bold")) +
theme(legend.text = element_text(face = "italic"))) + theme(axis.text.x =
element_text(colour = "black")) + theme(axis.text.y = element_text(colour =
"black")) + xlab(expression(bold("Treatments"))) +
ylab(expression(bold("Flight propensity")))
dev.off()

## png
##   2

###### Significance of treatments
##22 days


Age22_fig2 <- subset(tab2, Pupal_age== "22")

head(Age22_fig2 )

##    Replicate Pupal_age      Treatments out in. shipped irradiated chilled
## 25        R1        22   Shipped-110Gy  23   6       1          1       0
## 26        R1        22     Shipped-0Gy  24   7       1          0       0
```

```
## 27        R1        22 Unshipped-110Gy  79   0       0           1       0
## 28        R1        22   Unshipped-0Gy 104   3       0           0       0
## 29        R2        22  Shipped-110Gy    2   1       1           1       0
## 30        R2        22    Shipped-0Gy    5   1       1           0       0
##      Treatment_age         Treatment
## 25   Ship-110Gy_29  4_Shipped-110Gy
## 26    Ship-0Gy _29     2_Shipped-0Gy
## 27 Unship-110Gy-29 3_Unshipped-110Gy
## 28   Unship-0Gy-29   1_Unshipped-0Gy
## 29   Ship-110Gy_29  4_Shipped-110Gy
## 30    Ship-0Gy _29     2_Shipped-0Gy
```

```
Age22_fig2 $rate <- Age22_fig2 $out / (Age22_fig2 $in. + Age22_fig2 $out)
fm2_22 <- glmer(cbind(out,in.) ~ Treatment +(1|Replicate), family = binomial,
data = Age22_fig2 )
summary(fm2_22)
```

```
## Generalized linear mixed model fit by maximum likelihood (Laplace
##   Approximation) [glmerMod]
##  Family: binomial  ( logit )
## Formula: cbind(out, in.) ~ Treatment + (1 | Replicate)
##    Data: Age22_fig2
##
##      AIC      BIC   logLik deviance df.resid
##    113.9    118.8    -51.9    103.9       15
##
## Scaled residuals:
##     Min      1Q  Median      3Q     Max
## -3.2180 -0.7369  0.0110  0.5943  2.2968
##
## Random effects:
##  Groups    Name        Variance Std.Dev.
##  Replicate (Intercept) 0.4382   0.6619
## Number of obs: 20, groups:  Replicate, 5
##
## Fixed effects:
##                            Estimate Std. Error z value Pr(>|z|)
## (Intercept)                  3.5427     0.4064   8.718  < 2e-16 ***
## Treatment2_Shipped-0Gy      -0.6960     0.4068  -1.711 0.087072 .
## Treatment3_Unshipped-110Gy  -0.5125     0.3555  -1.442 0.149386
## Treatment4_Shipped-110Gy    -1.3569     0.3710  -3.657 0.000255 ***
## ---
## Signif. codes:  0 '***' 0.001 '**' 0.01 '*' 0.05 '.' 0.1 ' ' 1
##
## Correlation of Fixed Effects:
##             (Intr) T2_S-0 T3_U-1
## Trtmn2_S-0G -0.482
## Trt3_U-110G -0.509  0.509
## Trt4_S-110G -0.532  0.576  0.559
```

```
##29 days

Age29_fig2 <- subset(tab2, Pupal_age== "29")

head(Age29_fig2)

##   Replicate Pupal_age      Treatments out in. shipped irradiated chilled
## 1        R1        29  Shipped-110Gy  31  33       1          1       1
## 2        R1        29    Shipped-0Gy  26  28       1          0       1
## 3        R1        29 Unshipped-110Gy  30  42       0          1       1
## 4        R1        29   Unshipped-0Gy  21   9       0          0       1
## 5        R2        29  Shipped-110Gy  23  24       1          1       1
## 6        R2        29    Shipped-0Gy   9  30       1          0       1
##     Treatment_age         Treatment
## 1  Ship-110Gy _22  4_Shipped-110Gy
## 2    Ship-0Gy_22      2_Shipped-0Gy
## 3 Unship-110Gy_22 3_Unshipped-110Gy
## 4   Unship-0Gy_22    1_Unshipped-0Gy
## 5  Ship-110Gy _22  4_Shipped-110Gy
## 6    Ship-0Gy_22      2_Shipped-0Gy

Age29_fig2$rate <- Age29_fig2$out / (Age29_fig2$in. + Age29_fig2$out)
fm2_29 <- glmer(cbind(out,in.) ~ Treatment +(1|Replicate), family = binomial,
data = Age29_fig2)
summary(fm2_29)

## Generalized linear mixed model fit by maximum likelihood (Laplace
##   Approximation) [glmerMod]
##  Family: binomial  ( logit )
## Formula: cbind(out, in.) ~ Treatment + (1 | Replicate)
##    Data: Age29_fig2
##
##      AIC      BIC   logLik deviance df.resid
##    174.6    180.5    -82.3    164.6       19
##
## Scaled residuals:
##     Min      1Q  Median      3Q     Max
## -2.8970 -0.6356  0.1513  1.1538  2.4765
##
## Random effects:
##  Groups    Name        Variance Std.Dev.
##  Replicate (Intercept) 0.5157   0.7181
## Number of obs: 24, groups:  Replicate, 6
##
## Fixed effects:
##                           Estimate Std. Error z value Pr(>|z|)
## (Intercept)                0.94483    0.33275   2.839 0.004519 **
## Treatment2_Shipped-0Gy    -0.26291    0.20865  -1.260 0.207654
## Treatment3_Unshipped-110Gy -0.71771    0.20333  -3.530 0.000416 ***
## Treatment4_Shipped-110Gy   -0.06067    0.20619  -0.294 0.768581
```

```
## ---
## Signif. codes:  0 '***' 0.001 '**' 0.01 '*' 0.05 '.' 0.1 ' ' 1
##
## Correlation of Fixed Effects:
##              (Intr) T2_S-0 T3_U-1
## Trtmn2_S-0G -0.349
## Trt3_U-110G -0.356  0.576
## Trt4_S-110G -0.355  0.569  0.582
```

Figure 3: Mating ability(22 and 29 days)-differences between ages within each treatment

```
#####################-combined data###Figure 3

tab3= read.csv("Figure 3.csv")
head(tab3)

##   Replicate Pupal_age      Treatments unformed_pairs pairs_formed
intial_pairs
## 1        R1        29  Shipped-110Gy              4           26
30
## 2        R1        29    Shipped-0Gy              9           21
30
## 3        R1        29 Unshipped-110Gy             4           26
30
## 4        R1        29   Unshipped-0Gy             5           25
30
## 5        R2        29  Shipped-110Gy              6           24
30
## 6        R2        29    Shipped-0Gy             14           16
30
##   shipped irradiated chilled   Treatment_age         Treatment
## 1       1          1       1  Ship-110Gy _22  4_Shipped-110Gy
## 2       1          0       1    Ship-0Gy_22      2_Shipped-0Gy
## 3       0          1       1 Unship-110Gy_22 3_Unshipped-110Gy
## 4       0          0       1   Unship-0Gy_22   1_Unshipped-0Gy
## 5       1          1       1  Ship-110Gy _22  4_Shipped-110Gy
## 6       1          0       1    Ship-0Gy_22      2_Shipped-0Gy

mating_prop<- tab3$pairs_formed  / (tab3$unformed_pairs+ tab3$pairs_formed )
tab3$Pupal_age<- as.factor(tab3$Pupal_age)

Figure_3<-ggplot(tab3, aes(x=Treatments,y=mating_prop, colour=Pupal_age)) +
  geom_boxplot(position=position_dodge(0.8))+
  geom_jitter(position=position_dodge(0.8))
Figure_3
```

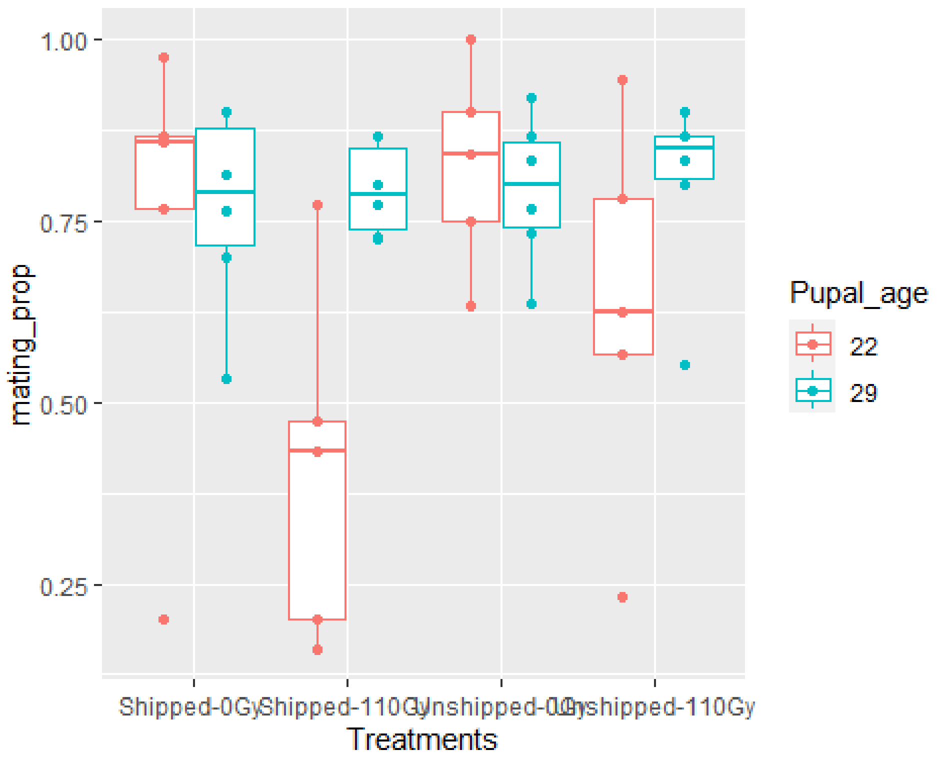


```
tiff("Figure 3.tiff", width = 7, height = 4, units = 'in',compression =
'lzw', res = 300)
plot(Figure_3+theme_tufte() + theme(axis.line = element_line(size = 1, colour
= "black")) + theme(legend.title = element_text(face = "bold")) +
theme(legend.text = element_text(face = "italic"))) + theme(axis.text.x =
element_text(colour = "black")) + theme(axis.text.y = element_text(colour =
"black")) + xlab(expression(bold("Treatments"))) +
ylab(expression(bold("Mating ability")))
dev.off()

## png
##   2

#### Significance of treatments

##22 days


Age22_fig3 <- subset(tab3, Pupal_age== "22")

head(Age22_fig3 )

##    Replicate Pupal_age      Treatments unformed_pairs pairs_formed
intial_pairs
## 25        R1        22   Shipped-110Gy             42            8
50
## 26        R1        22     Shipped-0Gy             40           10
```

```
50
## 27         R1         22 Unshipped-110Gy             11            39
50
## 28         R1         22   Unshipped-0Gy              8            42
50
## 29         R2         22  Shipped-110Gy               8            27
35
## 30         R2         22    Shipped-0Gy               5            30
35
##    shipped irradiated chilled   Treatment_age         Treatment
## 25       1          1       0   Ship-110Gy_29  4_Shipped-110Gy
## 26       1          0       0    Ship-0Gy _29     2_Shipped-0Gy
## 27       0          1       0 Unship-110Gy-29 3_Unshipped-110Gy
## 28       0          0       0   Unship-0Gy-29   1_Unshipped-0Gy
## 29       1          1       0   Ship-110Gy_29  4_Shipped-110Gy
## 30       1          0       0    Ship-0Gy _29     2_Shipped-0Gy
```

```
mating_prop <- Age22_fig3 $pairs_formed/ (Age22_fig3 $unformed_pairs+
Age22_fig3 $pairs_formed)
fm3_22<- glmer(cbind(pairs_formed, unformed_pairs) ~ Treatment
+(1|Replicate), family = binomial, data = Age22_fig3 )
summary(fm3_22)
```

```
## Generalized linear mixed model fit by maximum likelihood (Laplace
##   Approximation) [glmerMod]
##  Family: binomial  ( logit )
## Formula: cbind(pairs_formed, unformed_pairs) ~ Treatment + (1 | Replicate)
##    Data: Age22_fig3
##
##      AIC      BIC   logLik deviance df.resid
##    203.6    208.6    -96.8    193.6       15
##
## Scaled residuals:
##     Min      1Q  Median      3Q     Max
## -4.9052 -1.3221  0.4441  1.7504  3.9890
##
## Random effects:
##  Groups    Name        Variance Std.Dev.
##  Replicate (Intercept) 0.6739   0.8209
## Number of obs: 20, groups:  Replicate, 5
##
## Fixed effects:
##                            Estimate Std. Error z value Pr(>|z|)
## (Intercept)                  1.7983     0.4214   4.268 1.97e-05 ***
## Treatment2_Shipped-0Gy      -0.8152     0.2606  -3.128  0.00176 **
## Treatment3_Unshipped-110Gy  -1.0056     0.2583  -3.894 9.87e-05 ***
## Treatment4_Shipped-110Gy    -2.2063     0.2660  -8.296  < 2e-16 ***
## ---
## Signif. codes:  0 '***' 0.001 '**' 0.01 '*' 0.05 '.' 0.1 ' ' 1
##
```

```
## Correlation of Fixed Effects:
##              (Intr) T2_S-0 T3_U-1
## Trtmn2_S-0G -0.369
## Trt3_U-110G -0.374  0.596
## Trt4_S-110G -0.380  0.585  0.594
```

```
#29 days

Age29_fig3 <- subset(tab3, Pupal_age== "29")


head(Age29_fig3 )
```

```
##   Replicate Pupal_age      Treatments unformed_pairs pairs_formed
intial_pairs
## 1        R1        29  Shipped-110Gy              4           26
30
## 2        R1        29    Shipped-0Gy              9           21
30
## 3        R1        29 Unshipped-110Gy             4           26
30
## 4        R1        29   Unshipped-0Gy             5           25
30
## 5        R2        29  Shipped-110Gy              6           24
30
## 6        R2        29    Shipped-0Gy             14           16
30
##   shipped irradiated chilled   Treatment_age         Treatment
## 1       1          1       1  Ship-110Gy _22  4_Shipped-110Gy
## 2       1          0       1    Ship-0Gy_22      2_Shipped-0Gy
## 3       0          1       1 Unship-110Gy_22 3_Unshipped-110Gy
## 4       0          0       1   Unship-0Gy_22   1_Unshipped-0Gy
## 5       1          1       1  Ship-110Gy _22  4_Shipped-110Gy
## 6       1          0       1    Ship-0Gy_22      2_Shipped-0Gy
```

```
mating_prop <- Age29_fig3 $pairs_formed/ (Age29_fig3 $unformed_pairs+
Age29_fig3 $pairs_formed)
fm3_29 <- glmer(cbind(pairs_formed, unformed_pairs) ~ Treatment
+(1|Replicate), family = binomial, data = Age29_fig3 )
summary(fm3_29)
```

```
## Generalized linear mixed model fit by maximum likelihood (Laplace
##   Approximation) [glmerMod]
##  Family: binomial  ( logit )
## Formula: cbind(pairs_formed, unformed_pairs) ~ Treatment + (1 | Replicate)
##    Data: Age29_fig3
##
##      AIC      BIC   logLik deviance df.resid
##    118.9    124.8    -54.5    108.9       19
##
```

```
## Scaled residuals:
##     Min      1Q  Median      3Q     Max
## -1.8233 -1.0027  0.1686  0.7302  1.6753
##
## Random effects:
##  Groups    Name        Variance Std.Dev.
##  Replicate (Intercept) 0.1002   0.3165
## Number of obs: 24, groups:  Replicate, 6
##
## Fixed effects:
##                            Estimate Std. Error z value Pr(>|z|)
## (Intercept)                 1.46505    0.24386   6.008 1.88e-09 ***
## Treatment2_Shipped-0Gy     -0.26109    0.27905  -0.936    0.349
## Treatment3_Unshipped-110Gy -0.10218    0.28613  -0.357    0.721
## Treatment4_Shipped-110Gy   -0.06225    0.28394  -0.219    0.826
## ---
## Signif. codes:  0 '***' 0.001 '**' 0.01 '*' 0.05 '.' 0.1 ' ' 1
##
## Correlation of Fixed Effects:
##             (Intr) T2_S-0 T3_U-1
## Trtmn2_S-0G -0.618
## Trt3_U-110G -0.600  0.524
## Trt4_S-110G -0.609  0.530  0.515

###### Significance of treatments
```

Figure 4: Insemination rate(22 and 29 days)-differences between ages within each treatment

```
tab4= read.csv("Figure 4.csv")
head(tab4)

##   Replicate Pupal_age      Treatments Inseminated Empty  MSV shipped
irradiated
## 1        R1        22  Shipped-110Gy           8     0 0.75       1
1
## 2        R1        22    Shipped-0Gy          10     0 0.70       1
0
## 3        R1        22 Unshipped-110Gy         38     1 0.77       0
1
## 4        R1        22   Unshipped-0Gy         42     0 0.63       0
0
## 5        R2        22  Shipped-110Gy          23     4 0.41       1
1
## 6        R2        22    Shipped-0Gy          27     3 0.66       1
0
##   chilled   Treatment_age         Treatment
## 1       0  Ship-110Gy _22  4_Shipped-110Gy
## 2       0    Ship-0Gy_22      2_Shipped-0Gy
## 3       0 Unship-110Gy_22 3_Unshipped-110Gy
## 4       0   Unship-0Gy_22   1_Unshipped-0Gy
```

```
## 5       0  Ship-110Gy _22  4_Shipped-110Gy
## 6       0    Ship-0Gy_22      2_Shipped-0Gy

Insemination_rate<- tab4$Inseminated / (tab4$Empty+ tab4$Inseminated)
tab4$Pupal_age<- as.factor(tab4$Pupal_age)

Figure_4<-ggplot(tab4, aes(x=Treatments,y=Insemination_rate,
colour=Pupal_age)) +
  geom_boxplot(position=position_dodge(0.8))+
  geom_jitter(position=position_dodge(0.8))
Figure_4
```

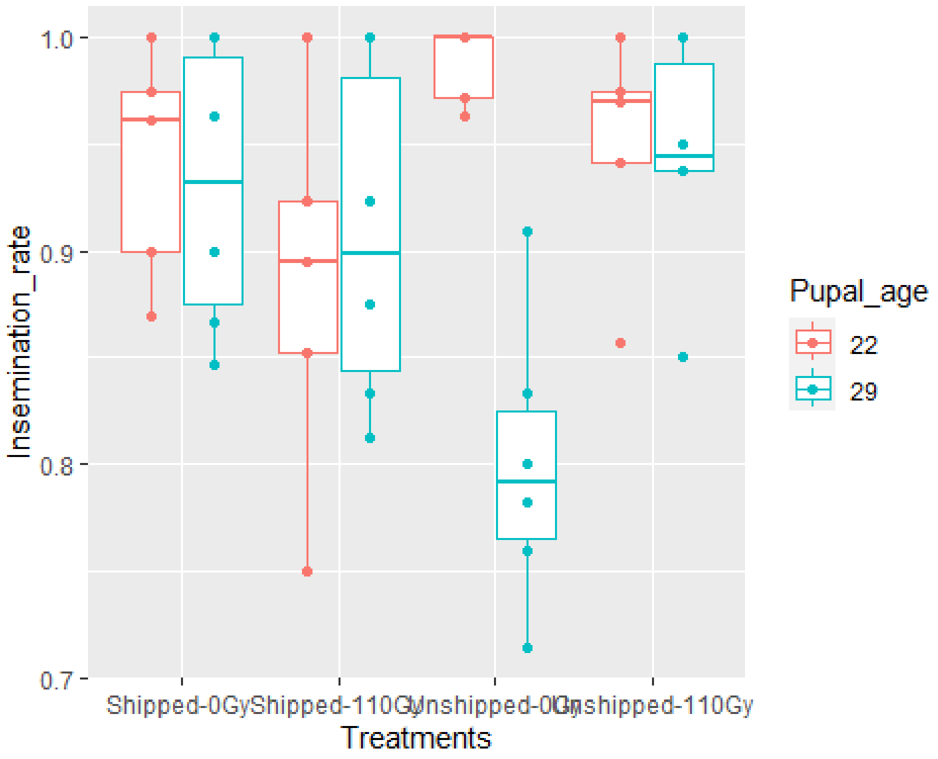


```
tiff("Figure 4.tiff", width = 7, height = 4, units = 'in',compression =
'lzw', res = 300)
plot(Figure_4+theme_tufte() + theme(axis.line = element_line(size = 1, colour
= "black")) + theme(legend.title = element_text(face = "bold")) +
theme(legend.text = element_text(face = "italic"))) + theme(axis.text.x =
element_text(colour = "black")) + theme(axis.text.y = element_text(colour =
"black")) + xlab(expression(bold("Treatments"))) +
ylab(expression(bold("Insemination rate")))
dev.off()

## png
##   2

#### Significance of treatments
```

```r
##22 days

Age22_fig4 <- subset(tab4, Pupal_age== "22")

head(Age22_fig4)

##   Replicate Pupal_age      Treatments Inseminated Empty  MSV shipped
irradiated
## 1        R1        22  Shipped-110Gy           8     0 0.75       1
1
## 2        R1        22    Shipped-0Gy          10     0 0.70       1
0
## 3        R1        22 Unshipped-110Gy         38     1 0.77       0
1
## 4        R1        22   Unshipped-0Gy         42     0 0.63       0
0
## 5        R2        22  Shipped-110Gy          23     4 0.41       1
1
## 6        R2        22    Shipped-0Gy          27     3 0.66       1
0
##   chilled   Treatment_age         Treatment
## 1       0  Ship-110Gy _22  4_Shipped-110Gy
## 2       0    Ship-0Gy_22      2_Shipped-0Gy
## 3       0 Unship-110Gy_22 3_Unshipped-110Gy
## 4       0   Unship-0Gy_22   1_Unshipped-0Gy
## 5       0  Ship-110Gy _22  4_Shipped-110Gy
## 6       0    Ship-0Gy_22      2_Shipped-0Gy

Insemination_rate<- Age22_fig4$Inseminated / (Age22_fig4$Empty+
Age22_fig4$Inseminated)
fm4_22 <- glmer(cbind(Inseminated,Empty) ~ Treatment +(1|Replicate), family =
binomial, data = Age22_fig4)
summary(fm4_22 )

## Generalized linear mixed model fit by maximum likelihood (Laplace
##   Approximation) [glmerMod]
##  Family: binomial  ( logit )
## Formula: cbind(Inseminated, Empty) ~ Treatment + (1 | Replicate)
##    Data: Age22_fig4
##
##      AIC      BIC   logLik deviance df.resid
##     54.7     59.7    -22.3     44.7       15
##
## Scaled residuals:
##     Min      1Q  Median      3Q     Max
## -1.0488 -0.5894  0.1182  0.5314  0.8374
##
## Random effects:
##  Groups    Name        Variance Std.Dev.
##  Replicate (Intercept) 0.2214   0.4706
```

```
## Number of obs: 20, groups:  Replicate, 6
##
## Fixed effects:
##                             Estimate Std. Error z value Pr(>|z|)
## (Intercept)                   4.4288     0.7572   5.849 4.94e-09 ***
## Treatment2_Shipped-0Gy       -1.6106     0.8093  -1.990  0.04658 *
## Treatment3_Unshipped-110Gy  -1.0402     0.8890  -1.170  0.24197
## Treatment4_Shipped-110Gy    -2.2996     0.8206  -2.802  0.00507 **
## ---
## Signif. codes:  0 '***' 0.001 '**' 0.01 '*' 0.05 '.' 0.1 ' ' 1
##
## Correlation of Fixed Effects:
##             (Intr) T2_S-0 T3_U-1
## Trtmn2_S-0G -0.835
## Trt3_U-110G -0.782  0.714
## Trt4_S-110G -0.839  0.782  0.722
```

```
##29 days

Age29_fig4  <- subset(tab4, Pupal_age== "29")

head(Age29_fig4 )
```

```
##    Replicate Pupal_age      Treatments Inseminated Empty       MSV shipped
## 21        R1        29  Shipped-110Gy          21     3 0.3333333       1
## 22        R1        29    Shipped-0Gy          18     2 0.4000000       0
## 23        R1        29 Unshipped-110Gy          15     1 0.6406250       0
## 24        R1        29   Unshipped-0Gy          19     6 0.3500000       1
## 25        R2        29  Shipped-110Gy          20     4 0.5520833       1
## 26        R2        29    Shipped-0Gy          13     2 0.4166667       0
##    irradiated chilled   Treatment_age         Treatment
## 21          0       1   Ship-110Gy_29  4_Shipped-110Gy
## 22          1       1    Ship-0Gy _29     2_Shipped-0Gy
## 23          0       1 Unship-110Gy-29 3_Unshipped-110Gy
## 24          1       1   Unship-0Gy-29   1_Unshipped-0Gy
## 25          0       1   Ship-110Gy_29  4_Shipped-110Gy
## 26          1       1    Ship-0Gy _29     2_Shipped-0Gy
```

```
Insemination_rate<- Age29_fig4 $Inseminated / (Age29_fig4 $Empty+ Age29_fig4
$Inseminated)
fm4_29 <- glmer(cbind(Inseminated,Empty) ~ Treatment +(1|Replicate), family =
binomial, data = Age29_fig4 )
```

```
## boundary (singular) fit: see help('isSingular')
```

```
summary(fm4_29)
```

```
## Generalized linear mixed model fit by maximum likelihood (Laplace
##   Approximation) [glmerMod]
##  Family: binomial  ( logit )
## Formula: cbind(Inseminated, Empty) ~ Treatment + (1 | Replicate)
```

```
##    Data: Age29_fig4
##
##      AIC      BIC   logLik deviance df.resid
##     82.5     88.4    -36.3     72.5       19
##
## Scaled residuals:
##     Min      1Q  Median      3Q     Max
## -1.7329 -0.6594 -0.0625  0.8207  1.4639
##
## Random effects:
##  Groups    Name        Variance  Std.Dev.
##  Replicate (Intercept) 9.631e-16 3.103e-08
## Number of obs: 24, groups:  Replicate, 7
##
## Fixed effects:
##                              Estimate Std. Error z value Pr(>|z|)
## (Intercept)                    1.4523     0.2368   6.132 8.69e-10 ***
## Treatment2_Shipped-0Gy         1.2558     0.4566   2.750  0.00595 **
## Treatment3_Unshipped-110Gy     1.3203     0.4829   2.734  0.00625 **
## Treatment4_Shipped-110Gy       0.7813     0.3852   2.029  0.04250 *
## ---
## Signif. codes:  0 '***' 0.001 '**' 0.01 '*' 0.05 '.' 0.1 ' ' 1
##
## Correlation of Fixed Effects:
##             (Intr) T2_S-0 T3_U-1
## Trtmn2_S-0G -0.519
## Trt3_U-110G -0.490  0.254
## Trt4_S-110G -0.615  0.319  0.302
## optimizer (Nelder_Mead) convergence code: 0 (OK)
## boundary (singular) fit: see help('isSingular')

###### Significance of treatments
```

Figure 5:Mean spermathecal value(22 and 29 days) differences between ages within each treatment

```
tab5= read.csv("Figure 5.csv")
head(tab5)
```

```
##   Replicate Pupal_age pairs_no      Treatments       MSV shipped
irradiated
## 1        R1        29       26   Shipped-110Gy  0.3333333       1
1
## 2        R1        29       21     Shipped-0Gy  0.4000000       1
0
## 3        R1        29       26 Unshipped-110Gy 0.6406250       0
1
## 4        R1        29       25   Unshipped-0Gy 0.3500000       0
0
## 5        R2        29       24   Shipped-110Gy  0.5520833       1
1
```

```
## 6        R2        29       16    Shipped-0Gy  0.4166667       1
0
##   chilled   Treatment_age         Treatment
## 1       1  Ship-110Gy _22  4_Shipped-110Gy
## 2       1    Ship-0Gy_22      2_Shipped-0Gy
## 3       1 Unship-110Gy_22 3_Unshipped-110Gy
## 4       1   Unship-0Gy_22    1_Unshipped-0Gy
## 5       1  Ship-110Gy _22  4_Shipped-110Gy
## 6       1    Ship-0Gy_22      2_Shipped-0Gy
```

```
tab5$Pupal_age<- as.factor(tab5$Pupal_age)

Figure_5<-ggplot(tab5, aes(x=Treatments,y=MSV, colour=Pupal_age)) +
  geom_boxplot(position=position_dodge(0.8))+
  geom_jitter(position=position_dodge(0.8))
Figure_5
```

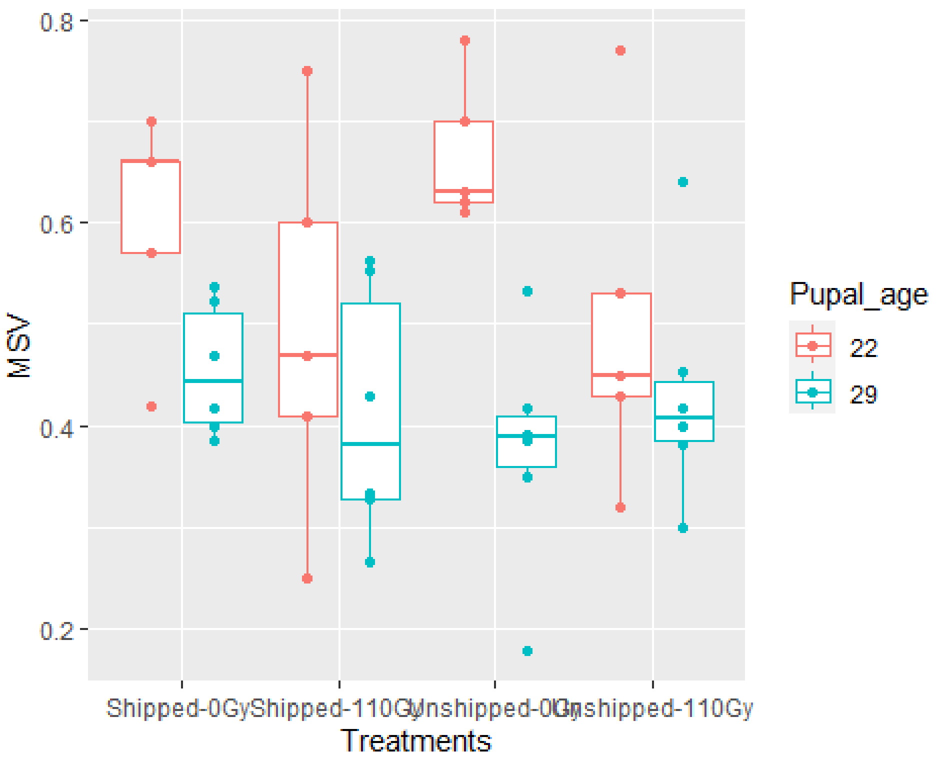


```
tiff("Figure 5.tiff", width = 7, height = 4, units = 'in',compression =
'lzw', res = 300)
plot(Figure_5 +theme_tufte() + theme(axis.line = element_line(size = 1,
colour = "black")) + theme(legend.title = element_text(face = "bold")) +
theme(legend.text = element_text(face = "italic"))) + theme(axis.text.x =
element_text(colour = "black")) + theme(axis.text.y = element_text(colour =
"black")) + xlab(expression(bold("Treatments"))) + ylab(expression(bold("Mean
Spermathecal Value (MSV)")))
dev.off()
```

```
## png
##   2
```

Significance of treatments

22 days

```
Age22_fig5 <- subset(tab5, Pupal_age== "22")

head(Age22_fig5)

##    Replicate Pupal_age pairs_no      Treatments  MSV shipped irradiated
chilled
## 25        R1        22        8  Shipped-110Gy  0.75       1          1
0
## 26        R1        22       10    Shipped-0Gy  0.70       1          0
0
## 27        R1        22       39 Unshipped-110Gy 0.77       0          1
0
## 28        R1        22       42   Unshipped-0Gy 0.63       0          0
0
## 29        R2        22       27  Shipped-110Gy  0.41       1          1
0
## 30        R2        22       30    Shipped-0Gy  0.66       1          0
0
##      Treatment_age         Treatment
## 25   Ship-110Gy_29   4_Shipped-110Gy
## 26    Ship-0Gy _29     2_Shipped-0Gy
## 27 Unship-110Gy-29 3_Unshipped-110Gy
## 28   Unship-0Gy-29   1_Unshipped-0Gy
## 29   Ship-110Gy_29   4_Shipped-110Gy
## 30    Ship-0Gy _29     2_Shipped-0Gy

fm5_22<- lme(MSV ~ Treatment, random=~1|Replicate,data = Age22_fig5)
summary(fm5_22)

## Linear mixed-effects model fit by REML
##   Data: Age22_fig5
##          AIC      BIC   logLik
##   -0.5870663 4.048466 6.293533
##
## Random effects:
##  Formula: ~1 | Replicate
##         (Intercept) Residual
## StdDev:   0.0845309 0.114734
##
## Fixed effects:  MSV ~ Treatment
##                                Value  Std.Error DF   t-value p-value
## (Intercept)                0.6608483 0.06260163 11 10.556407  0.0000
```

```
## Treatment2_Shipped-0Gy     -0.0504640 0.07478648 11 -0.674775  0.5138
## Treatment3_Unshipped-110Gy -0.1524640 0.07478648 11 -2.038657  0.0663
## Treatment4_Shipped-110Gy   -0.1564640 0.07478648 11 -2.092143  0.0604
##  Correlation:
##                            (Intr) T2_S-0 T3_U-1
## Treatment2_Shipped-0Gy     -0.591
## Treatment3_Unshipped-110Gy -0.591  0.529
## Treatment4_Shipped-110Gy   -0.591  0.529  0.529
##
## Standardized Within-Group Residuals:
##          Min           Q1          Med           Q3          Max
## -1.420514088 -0.461751338  0.005852858  0.495319900  1.435806112
##
## Number of Observations: 20
## Number of Groups: 6
```

```
##29days

Age29_fig5  <- subset(tab5, Pupal_age== "29")

head(Age29_fig5)
```

```
##   Replicate Pupal_age pairs_no      Treatments       MSV shipped
irradiated
## 1        R1        29       26  Shipped-110Gy  0.3333333       1
1
## 2        R1        29       21    Shipped-0Gy  0.4000000       1
0
## 3        R1        29       26 Unshipped-110Gy 0.6406250       0
1
## 4        R1        29       25   Unshipped-0Gy 0.3500000       0
0
## 5        R2        29       24  Shipped-110Gy  0.5520833       1
1
## 6        R2        29       16    Shipped-0Gy  0.4166667       1
0
##   chilled   Treatment_age         Treatment
## 1       1  Ship-110Gy _22  4_Shipped-110Gy
## 2       1    Ship-0Gy_22      2_Shipped-0Gy
## 3       1 Unship-110Gy_22 3_Unshipped-110Gy
## 4       1   Unship-0Gy_22   1_Unshipped-0Gy
## 5       1  Ship-110Gy _22  4_Shipped-110Gy
## 6       1    Ship-0Gy_22      2_Shipped-0Gy
```

```
fmS26<- lme(MSV ~ Treatments, random=~1|Replicate,data = Age29_fig5)
summary(fmS26)
```

```
## Linear mixed-effects model fit by REML
##   Data: Age29_fig5
##         AIC       BIC   logLik
##   -13.49243 -7.518039 12.74622
```

```
##
## Random effects:
##  Formula: ~1 | Replicate
##         (Intercept)  Residual
## StdDev:  0.02768739 0.1036417
##
## Fixed effects:  MSV ~ Treatments
##                                Value  Std.Error DF   t-value p-value
## (Intercept)                0.4551464 0.04379535 15 10.392575   0.000
## TreatmentsShipped-110Gy   -0.0436404 0.05983757 15 -0.729315   0.477
## TreatmentsUnshipped-0Gy   -0.0791065 0.05983757 15 -1.322021   0.206
## TreatmentsUnshipped-110Gy -0.0232516 0.05983757 15 -0.388579   0.703
##  Correlation:
##                           (Intr) TS-110 TrU-0G
## TreatmentsShipped-110Gy   -0.683
## TreatmentsUnshipped-0Gy   -0.683  0.500
## TreatmentsUnshipped-110Gy -0.683  0.500  0.500
##
## Standardized Within-Group Residuals:
##         Min          Q1         Med          Q3         Max
## -1.76272348 -0.54309737  0.03434292  0.41863725  1.98751241
##
## Number of Observations: 24
## Number of Groups: 6
```

Figure 6a:Spermathecal fill distribution- 22 days-Chi square values

```
#first,full dataset
tab <- read.csv("spermfill_22.csv", sep=",")
summary(tab)

##   Treatments              X0           X0.25            X0.5
##  Length:4           Min.   :2.0   Min.   :26.00   Min.   :11.00
##  Class :character   1st Qu.:3.5   1st Qu.:31.25   1st Qu.:22.25
##  Mode  :character   Median :6.0   Median :34.50   Median :27.00
##                     Mean   :5.5   Mean   :34.00   Mean   :24.50
##                     3rd Qu.:8.0   3rd Qu.:37.25   3rd Qu.:29.25
##                     Max.   :8.0   Max.   :41.00   Max.   :33.00
##      X0.75              X1
##  Min.   :13.00   Min.   :13.0
##  1st Qu.:13.75   1st Qu.:25.0
##  Median :16.50   Median :36.5
##  Mean   :21.00   Mean   :33.5
##  3rd Qu.:23.75   3rd Qu.:45.0
##  Max.   :38.00   Max.   :48.0

head(tab)

##        Treatments X0 X0.25 X0.5 X0.75 X1
## 1  Shipped-110Gy   8    26   11    13 13
## 2    Shipped-0Gy   8    36   26    14 44
```

```
## 3 Unshipped-110Gy  4    41   28    19 29
## 4   Unshipped-0Gy  2    33   33    38 48
```

```r
chisq.test(tab[,5:6])
```

```
##
##  Pearson's Chi-squared test
##
## data:  tab[, 5:6]
## X-squared = 7.7006, df = 3, p-value = 0.05262
```

```r
tab <- read.csv("spermfill_22.csv", sep=",")
summary(tab)
```

```
##   Treatments              X0          X0.25            X0.5
##  Length:4           Min.   :2.0   Min.   :26.00   Min.   :11.00
##  Class :character   1st Qu.:3.5   1st Qu.:31.25   1st Qu.:22.25
##  Mode  :character   Median :6.0   Median :34.50   Median :27.00
##                     Mean   :5.5   Mean   :34.00   Mean   :24.50
##                     3rd Qu.:8.0   3rd Qu.:37.25   3rd Qu.:29.25
##                     Max.   :8.0   Max.   :41.00   Max.   :33.00
##      X0.75            X1
##  Min.   :13.00   Min.   :13.0
##  1st Qu.:13.75   1st Qu.:25.0
##  Median :16.50   Median :36.5
##  Mean   :21.00   Mean   :33.5
##  3rd Qu.:23.75   3rd Qu.:45.0
##  Max.   :38.00   Max.   :48.0
```

```r
head(tab)
```

```
##        Treatments X0 X0.25 X0.5 X0.75 X1
## 1  Shipped-110Gy   8    26   11    13 13
## 2    Shipped-0Gy   8    36   26    14 44
## 3 Unshipped-110Gy  4    41   28    19 29
## 4   Unshipped-0Gy  2    33   33    38 48
```

```r
chisq.test(tab[,2:6])
```

```
## Warning in chisq.test(tab[, 2:6]): Chi-squared approximation may be
incorrect

##
##  Pearson's Chi-squared test
##
## data:  tab[, 2:6]
## X-squared = 31.717, df = 12, p-value = 0.00153
```

```r
tab<- read.csv("spermfill_22_ship_110Gy.csv", sep=",")
summary(tab)
```

```
##   Treatments              X0         X0.25         X0.5         X0.75   
##  Length:1           Min.   :8   Min.   :26   Min.   :11   Min.   :13  
##  Class :character   1st Qu.:8   1st Qu.:26   1st Qu.:11   1st Qu.:13  
##  Mode  :character   Median :8   Median :26   Median :11   Median :13  
##                     Mean   :8   Mean   :26   Mean   :11   Mean   :13  
##                     3rd Qu.:8   3rd Qu.:26   3rd Qu.:11   3rd Qu.:13  
##                     Max.   :8   Max.   :26   Max.   :11   Max.   :13  
##        X1    
##  Min.   :13  
##  1st Qu.:13  
##  Median :13  
##  Mean   :13  
##  3rd Qu.:13  
##  Max.   :13
```

```
head(tab)
```

```
##       Treatments X0 X0.25 X0.5 X0.75 X1
## 1 Shipped-110Gy   8    26   11    13 13
```

```
chisq.test(tab[,2:6])
```

```
## 
##  Chi-squared test for given probabilities
## 
## data:  tab[, 2:6]
## X-squared = 13.437, df = 4, p-value = 0.009328
```

```
tab <- read.csv("spermfill_22_Shipped-0Gy .csv", sep=",")
summary(tab)
```

```
##   Treatments              X0         X0.25         X0.5         X0.75   
##  Length:1           Min.   :8   Min.   :36   Min.   :26   Min.   :14  
##  Class :character   1st Qu.:8   1st Qu.:36   1st Qu.:26   1st Qu.:14  
##  Mode  :character   Median :8   Median :36   Median :26   Median :14  
##                     Mean   :8   Mean   :36   Mean   :26   Mean   :14  
##                     3rd Qu.:8   3rd Qu.:36   3rd Qu.:26   3rd Qu.:14  
##                     Max.   :8   Max.   :36   Max.   :26   Max.   :14  
##        X1      Grand.total 
##  Min.   :44   Min.   :128  
##  1st Qu.:44   1st Qu.:128  
##  Median :44   Median :128  
##  Mean   :44   Mean   :128  
##  3rd Qu.:44   3rd Qu.:128  
##  Max.   :44   Max.   :128
```

```
head(tab)
```

```
##     Treatments X0 X0.25 X0.5 X0.75 X1 Grand.total
## 1 Shipped-0Gy   8    36   26    14 44         128
```

```
chisq.test(tab[,2:6])
```

```
##
##  Chi-squared test for given probabilities
##
## data:  tab[, 2:6]
## X-squared = 34.812, df = 4, p-value = 5.076e-07
```

```
tab <- read.csv("spermfill_22_Unshipped-0Gy.csv", sep=",")
summary(tab)
```

```
##   Treatments              X0          X0.25          X0.5          X0.75
##  Length:1           Min.   :2   Min.   :33   Min.   :33   Min.   :38
##  Class :character   1st Qu.:2   1st Qu.:33   1st Qu.:33   1st Qu.:38
##  Mode  :character   Median :2   Median :33   Median :33   Median :38
##                     Mean   :2   Mean   :33   Mean   :33   Mean   :38
##                     3rd Qu.:2   3rd Qu.:33   3rd Qu.:33   3rd Qu.:38
##                     Max.   :2   Max.   :33   Max.   :33   Max.   :38
##        X1       Grand.total
##  Min.   :48   Min.   :154
##  1st Qu.:48   1st Qu.:154
##  Median :48   Median :154
##  Mean   :48   Mean   :154
##  3rd Qu.:48   3rd Qu.:154
##  Max.   :48   Max.   :154
```

```
head(tab)
```

```
##      Treatments X0 X0.25 X0.5 X0.75 X1 Grand.total
## 1 Unshipped-0Gy  2    33   33    38 48         154
```

```
chisq.test(tab[,2:6])
```

```
##
##  Chi-squared test for given probabilities
##
## data:  tab[, 2:6]
## X-squared = 38.532, df = 4, p-value = 8.701e-08
```

```
tab <- read.csv("spermfill_22_Unshipped-110Gy.csv", sep=",")
summary(tab)
```

```
##   Treatments              X0          X0.25          X0.5          X0.75
##  Length:1           Min.   :4   Min.   :41   Min.   :28   Min.   :19
##  Class :character   1st Qu.:4   1st Qu.:41   1st Qu.:28   1st Qu.:19
##  Mode  :character   Median :4   Median :41   Median :28   Median :19
##                     Mean   :4   Mean   :41   Mean   :28   Mean   :19
##                     3rd Qu.:4   3rd Qu.:41   3rd Qu.:28   3rd Qu.:19
##                     Max.   :4   Max.   :41   Max.   :28   Max.   :19
##        X1
##  Min.   :29
##  1st Qu.:29
##  Median :29
##  Mean   :29
```

```
##  3rd Qu.:29
##  Max.   :29

head(tab)

##         Treatments X0 X0.25 X0.5 X0.75 X1
## 1 Unshipped-110Gy  4    41   28    19 29

chisq.test(tab[,2:6])

##
##  Chi-squared test for given probabilities
##
## data:  tab[, 2:6]
## X-squared = 31.19, df = 4, p-value = 2.8e-06
```

Figure 6b: Spermathecal fill distribution- Chi square values

```
#first,full dataset
tab <- read.csv("Spermfill_29.csv", sep=",")
summary(tab)

##   Treatment                X0            X0.25            X0.5
##  Length:4           Min.   : 6.00   Min.   :31.00   Min.   :28.00
##  Class :character   1st Qu.: 6.75   1st Qu.:31.75   1st Qu.:34.00
##  Mode  :character   Median : 9.50   Median :40.00   Median :38.00
##                     Mean   :11.75   Mean   :40.75   Mean   :38.75
##                     3rd Qu.:14.50   3rd Qu.:49.00   3rd Qu.:42.75
##                     Max.   :22.00   Max.   :52.00   Max.   :51.00
##      X0.75            X1         Grand.total
##  Min.   :11.0   Min.   :4.00   Min.   :102.0
##  1st Qu.:14.0   1st Qu.:6.25   1st Qu.:109.5
##  Median :15.5   Median :8.00   Median :114.0
##  Mean   :15.0   Mean   :7.25   Mean   :113.5
##  3rd Qu.:16.5   3rd Qu.:9.00   3rd Qu.:118.0
##  Max.   :18.0   Max.   :9.00   Max.   :124.0

head(tab)

##          Treatment X0 X0.25 X0.5 X0.75 X1 Grand.total
## 1  Shipped-110Gy  12    52   36    15  9         124
## 2    Shipped-0Gy   7    31   51    16  7         112
## 3 Unshipped-110Gy  6    48   28    11  9         102
## 4   Unshipped-0Gy 22    32   40    18  4         116

chisq.test(tab[,5:6])

##
##  Pearson's Chi-squared test
##
## data:  tab[, 5:6]
## X-squared = 3.7933, df = 3, p-value = 0.2847
```

```
tab <- read.csv("Spermfill_29_Ship-0Gy.csv", sep=",")
summary(tab)

##   Treatment               X0        X0.25         X0.5        X0.75
##  Length:1           Min.   :7   Min.   :31   Min.   :51   Min.   :16
##  Class :character   1st Qu.:7   1st Qu.:31   1st Qu.:51   1st Qu.:16
##  Mode  :character   Median :7   Median :31   Median :51   Median :16
##                     Mean   :7   Mean   :31   Mean   :51   Mean   :16
##                     3rd Qu.:7   3rd Qu.:31   3rd Qu.:51   3rd Qu.:16
##                     Max.   :7   Max.   :31   Max.   :51   Max.   :16
##        X1      Grand.total
##  Min.   :7   Min.   :112
##  1st Qu.:7   1st Qu.:112
##  Median :7   Median :112
##  Mean   :7   Mean   :112
##  3rd Qu.:7   3rd Qu.:112
##  Max.   :7   Max.   :112

head(tab)

##      Treatment X0 X0.25 X0.5 X0.75 X1 Grand.total
## 1 Shipped-0Gy   7    31   51    16  7         112

chisq.test(tab[,2:6])

##
##  Chi-squared test for given probabilities
##
## data:  tab[, 2:6]
## X-squared = 62.821, df = 4, p-value = 7.399e-13

tab <- read.csv("Spermfill_29_Ship-110Gy.csv", sep=",")
summary(tab)

##   Treatment               X0         X0.25          X0.5         X0.75
##  Length:1           Min.   :12   Min.   :52   Min.   :36   Min.   :15
##  Class :character   1st Qu.:12   1st Qu.:52   1st Qu.:36   1st Qu.:15
##  Mode  :character   Median :12   Median :52   Median :36   Median :15
##                     Mean   :12   Mean   :52   Mean   :36   Mean   :15
##                     3rd Qu.:12   3rd Qu.:52   3rd Qu.:36   3rd Qu.:15
##                     Max.   :12   Max.   :52   Max.   :36   Max.   :15
##        X1      Grand.total
##  Min.   :9   Min.   :124
##  1st Qu.:9   1st Qu.:124
##  Median :9   Median :124
##  Mean   :9   Mean   :124
##  3rd Qu.:9   3rd Qu.:124
##  Max.   :9   Max.   :124

head(tab)
```

```
##        Treatment X0 X0.25 X0.5 X0.75 X1 Grand.total
## 1 Shipped-110Gy  12    52   36    15  9         124
```

```
chisq.test(tab[,2:6])
```

```
##
##  Chi-squared test for given probabilities
##
## data:  tab[, 2:6]
## X-squared = 55.435, df = 4, p-value = 2.633e-11
```

```
tab <- read.csv("Spermfill_29_Unship-110Gy.csv", sep=",")
summary(tab)
```

```
##   Treatment                X0         X0.25         X0.5         X0.75
##  Length:1           Min.   :6   Min.   :48   Min.   :28   Min.   :11
##  Class :character   1st Qu.:6   1st Qu.:48   1st Qu.:28   1st Qu.:11
##  Mode  :character   Median :6   Median :48   Median :28   Median :11
##                     Mean   :6   Mean   :48   Mean   :28   Mean   :11
##                     3rd Qu.:6   3rd Qu.:48   3rd Qu.:28   3rd Qu.:11
##                     Max.   :6   Max.   :48   Max.   :28   Max.   :11
##        X1     Grand.total
##  Min.   :9   Min.   :102
##  1st Qu.:9   1st Qu.:102
##  Median :9   Median :102
##  Mean   :9   Mean   :102
##  3rd Qu.:9   3rd Qu.:102
##  Max.   :9   Max.   :102
```

```
head(tab)
```

```
##          Treatment X0 X0.25 X0.5 X0.75 X1 Grand.total
## 1 Unshipped-110Gy  6    48   28    11  9         102
```

```
chisq.test(tab[,2:6])
```

```
##
##  Chi-squared test for given probabilities
##
## data:  tab[, 2:6]
## X-squared = 61.039, df = 4, p-value = 1.754e-12
```

```
tab <- read.csv("Spermfill_29_Unshipped-0Gy.csv", sep=",")
summary(tab)
```

```
##   Treatment                X0          X0.25          X0.5          X0.75
##  Length:1           Min.   :22   Min.   :32   Min.   :40   Min.   :18
##  Class :character   1st Qu.:22   1st Qu.:32   1st Qu.:40   1st Qu.:18
##  Mode  :character   Median :22   Median :32   Median :40   Median :18
##                     Mean   :22   Mean   :32   Mean   :40   Mean   :18
##                     3rd Qu.:22   3rd Qu.:32   3rd Qu.:40   3rd Qu.:18
##                     Max.   :22   Max.   :32   Max.   :40   Max.   :18
```

```
##        X1     Grand.total
##  Min.   :4   Min.   :116
##  1st Qu.:4   1st Qu.:116
##  Median :4   Median :116
##  Mean   :4   Mean   :116
##  3rd Qu.:4   3rd Qu.:116
##  Max.   :4   Max.   :116
```

```
head(tab)
```

```
##       Treatment X0 X0.25 X0.5 X0.75 X1 Grand.total
## 1 Unshipped-0Gy 22    32   40    18  4         116
```

```
chisq.test(tab[,2:6])
```

```
## 
##  Chi-squared test for given probabilities
## 
## data:  tab[, 2:6]
## X-squared = 32.621, df = 4, p-value = 1.428e-06
```

Kaplan Meier Analysis (km)on the impact of treatments on the duration of survival of blood fed flies(trends)

Figure 7a and 7b

```
##Figure 7a:Comparing difference of trends between treatments(22 days old )

data_2=read.csv("Figure 7a.csv")

#data_2
str(data_2)
```

```
## 'data.frame':    311 obs. of  7 variables:
##  $ day       : int  30 41 41 48 62 80 80 90 90 90 ...
##  $ Pupal_age : int  22 22 22 22 22 22 22 22 22 22 ...
##  $ Replicate : int  1 1 1 1 1 1 1 1 1 1 ...
##  $ Cohort    : int  1 1 1 1 1 1 1 1 1 1 ...
##  $ Treatments: chr  "Unshipped-0Gy" "Unshipped-0Gy" "Unshipped-0Gy" 
"Unshipped-0Gy" ...
##  $ status    : int  1 1 1 1 1 1 1 1 1 1 ...
##  $ Treatment : chr  "1_Unshipped-0Gy" "1_Unshipped-0Gy" "1_Unshipped-0Gy" 
"1_Unshipped-0Gy" ...
```

```
head(data_2)
```

```
##   day Pupal_age Replicate Cohort    Treatments status       Treatment
## 1  30        22         1      1 Unshipped-0Gy      1 1_Unshipped-0Gy
## 2  41        22         1      1 Unshipped-0Gy      1 1_Unshipped-0Gy
## 3  41        22         1      1 Unshipped-0Gy      1 1_Unshipped-0Gy
## 4  48        22         1      1 Unshipped-0Gy      1 1_Unshipped-0Gy
```

```
## 5  62         22          1       1 Unshipped-0Gy      1 1_Unshipped-0Gy
## 6  80         22          1       1 Unshipped-0Gy      1 1_Unshipped-0Gy

data_2=na.omit(data_2)
names(data_2)

## [1] "day"          "Pupal_age"  "Replicate"  "Cohort"      "Treatments"
## [6] "status"      "Treatment"

km <- with(data_2, Surv(day, status))
head(km,115)

##   [1]  30  41  41  48  62  80  80  90  90  90   1  34  34  35  58  58  66
87
##  [19] 104 104  14  16  20  20  21  27  27  27  27  27  30  30  34  34  35
35
##  [37]  38  41  41  41  41  22  23  23  23  27  28  28  30  30  30  34  36
36
##  [55]  41  41   1  30  34  34  38  38  45  64  70  58  70  78  83  97  97
104
##  [73] 108  16  23  23  23  27  27  27  27  27  28  28  30  30  34  34  36
41
##  [91]  41  42  42  45   2  20  20  27  27  27  27  27  28  28  28  30  30
41
## [109]  41  20  21  38  38  50  55

km_trt_fit <- survfit(Surv(day, status) ~ Treatment, data=data_2)
plot(survfit(Surv(day,status)~Treatments,data=data_2), xlab = "Time(days)",
ylab = "Survival rate (22 day-old)", col=c('red','blue','green','orange'),
lwd=2, xlim =c(0, 115))
legend('topright', text.font =, cex=1, c("Control","Shipped",
"Irradiated","shipped_irradiated"), col=c('red','blue', 'green','orange'),
lty = 1, lwd=2, box.lty = 1)
```

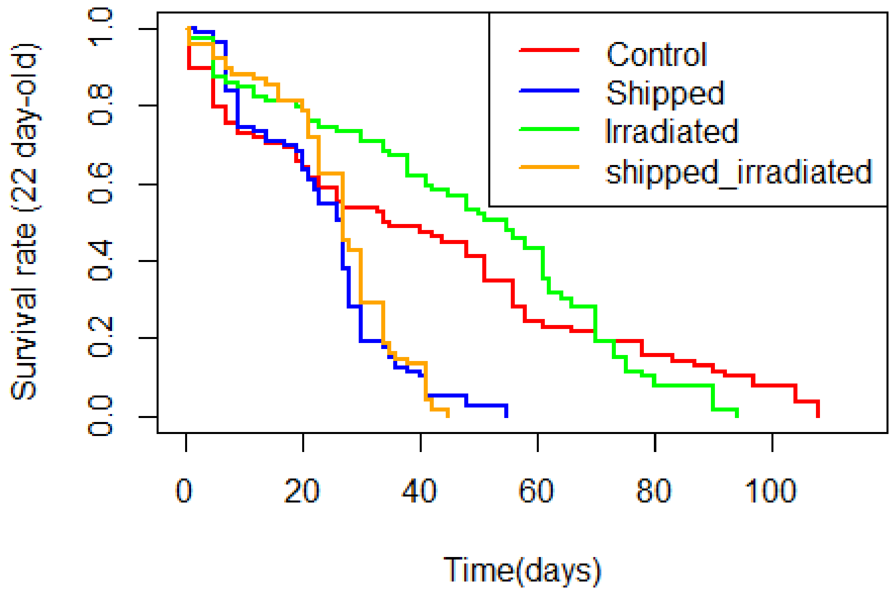


```
## survival: median Kaplan-Meier estimator
survfit(Surv(day,status)~Treatment,data=data_2)

## Call: survfit(formula = Surv(day, status) ~ Treatment, data = data_2)
##
##                                 n events median 0.95LCL 0.95UCL
## Treatment=1_Unshipped-0Gy    79     79   55.0      41      61
## Treatment=2_Shipped-0Gy      78     78   34.5      23      51
## Treatment=3_Unshipped-110Gy 75     75   27.0      27      30
## Treatment=4_Shipped-110Gy    79     79   27.0      22      28

#data_2$Treatment <- relevel(data_2$Treatment, ref = "Unshipped-0Gy")
cox.surv <- coxph(Surv(day, status) ~ Treatment, data = data_2, method
="exact")
summary(cox.surv)

## Call:
## coxph(formula = Surv(day, status) ~ Treatment, data = data_2,
##     method = "exact")
##
##   n= 311, number of events= 311
##
##                               coef exp(coef) se(coef)     z Pr(>|z|)
## Treatment2_Shipped-0Gy     0.05615   1.05776  0.17289 0.325    0.745
## Treatment3_Unshipped-110Gy 1.21040   3.35482  0.19234 6.293 3.11e-10 ***
## Treatment4_Shipped-110Gy   1.34570   3.84087  0.18984 7.089 1.36e-12 ***
## ---
```

```
## Signif. codes:  0 '***' 0.001 '**' 0.01 '*' 0.05 '.' 0.1 ' ' 1
##
##                              exp(coef) exp(-coef) lower .95 upper .95
## Treatment2_Shipped-0Gy           1.058     0.9454    0.7537     1.484
## Treatment3_Unshipped-110Gy       3.355     0.2981    2.3012     4.891
## Treatment4_Shipped-110Gy         3.841     0.2604    2.6475     5.572
##
## Concordance= 0.611  (se = 0.018 )
## Likelihood ratio test= 74.6  on 3 df,   p=4e-16
## Wald test            = 69.2  on 3 df,   p=6e-15
## Score (logrank) test = 75.34  on 3 df,   p=3e-16
```

Figure 7b:Comparing difference of trends between treatments(29 day old)

```
data_3=read.csv("Figure 7b.csv")

#data_3
str(data_3)

## 'data.frame':    324 obs. of  7 variables:
##  $ day       : int  3 3 27 36 50 55 80 90 94 94 ...
##  $ Pupal_age : int  29 29 29 29 29 29 29 29 29 29 ...
##  $ Replicate : int  1 1 1 1 1 1 1 1 1 1 ...
##  $ Cohort    : int  1 1 1 1 1 1 1 1 1 1 ...
##  $ Treatments: chr  "Unshipped-0Gy" "Unshipped-0Gy" "Unshipped-0Gy"
"Unshipped-0Gy" ...
##  $ status    : int  1 1 1 1 1 1 1 1 1 1 ...
##  $ Treatment : chr  "1_Unshipped-0Gy" "1_Unshipped-0Gy" "1_Unshipped-0Gy"
"1_Unshipped-0Gy" ...

head(data_3)

##   day Pupal_age Replicate Cohort    Treatments status       Treatment
## 1   3        29         1      1 Unshipped-0Gy      1 1_Unshipped-0Gy
## 2   3        29         1      1 Unshipped-0Gy      1 1_Unshipped-0Gy
## 3  27        29         1      1 Unshipped-0Gy      1 1_Unshipped-0Gy
## 4  36        29         1      1 Unshipped-0Gy      1 1_Unshipped-0Gy
## 5  50        29         1      1 Unshipped-0Gy      1 1_Unshipped-0Gy
## 6  55        29         1      1 Unshipped-0Gy      1 1_Unshipped-0Gy

data_3=na.omit(data_3)
names(data_3)

## [1] "day"        "Pupal_age"  "Replicate"  "Cohort"     "Treatments"
## [6] "status"     "Treatment"

km <- with(data_3, Surv(day, status))
head(km,115)

##   [1]   3   3  27  36  50  55  80  90  94  94  97  97 100 108   7  35  36
45
##  [19]  52  71  73  80  92  92  94 100 104 104 104 108 108   3   3   8  21
```

```
30
## [37]  34  34  36  36  37  38  41  41  41  45  45  48  50  50  50  55  71
2
## [55]   3   6   6   6  28  34  37  41  41  41  42  43  48  48  50  50  52
55
## [73]  71   3   3   3   6   6   7  30  42  71  71  71  87 100 100 101   6
6
## [91]   6  30  34  34  71  87  90  94 101 101   1   8   8  20  27  34  34
36
## [109]  38  41  41  42  42  42  42
```

```
km_trt_fit <- survfit(Surv(day, status) ~ Treatment, data=data_3)
plot(survfit(Surv(day,status)~Treatments,data=data_3), xlab = "Time(days)",
ylab = "Survival rate (29 day-old)", col=c('red','blue','green','orange'),
lwd=2, xlim =c(0, 115))
legend('topright', text.font = , cex=1, c("Control","Shipped",
"Irradiated","shipped and irradiated"), col=c('red','blue',
'green','orange'), lty = 1, lwd=2, box.lty = 1)
```

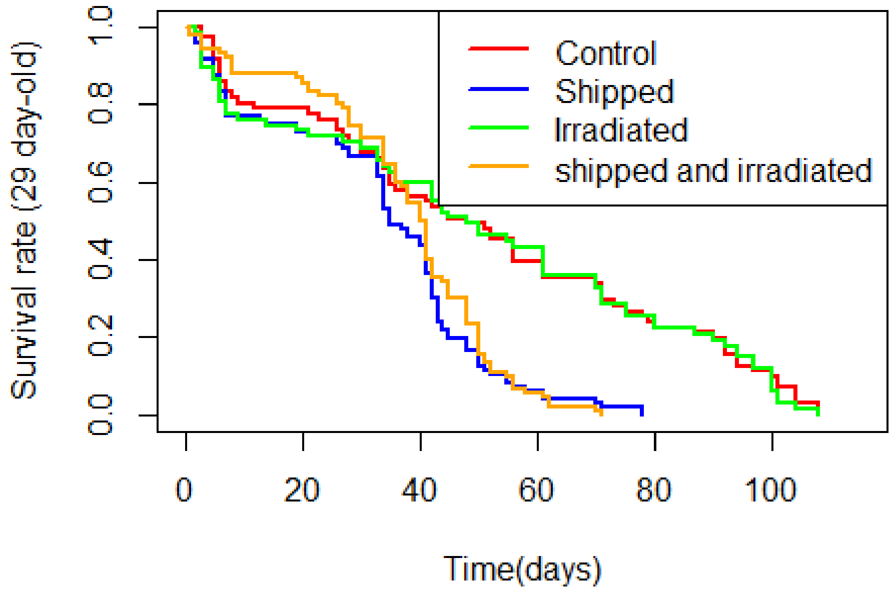


```
### survival: median Kaplan-Meier estimator
survfit(Surv(day,status)~Treatment,data=data_3)

## Call: survfit(formula = Surv(day, status) ~ Treatment, data = data_3)
##
##                              n events median 0.95LCL 0.95UCL
## Treatment=1_Unshipped-0Gy   67     67   48.0      36      61
## Treatment=2_Shipped-0Gy     71     71   48.0      35      61
```

```
## Treatment=3_Unshipped-110Gy 90      90   40.5       37       42
## Treatment=4_Shipped-110Gy   96      96   35.0       34       41
```

```
cox.surv <- coxph(Surv(day, status) ~ Treatment, data = data_3, method
="exact")
summary(cox.surv)
```

```
## Call:
## coxph(formula = Surv(day, status) ~ Treatment, data = data_3,
##     method = "exact")
##
##   n= 324, number of events= 324
##
##                                coef exp(coef) se(coef)      z Pr(>|z|)
## Treatment2_Shipped-0Gy     -0.04147   0.95937  0.18030 -0.230    0.818
## Treatment3_Unshipped-110Gy  0.83723   2.30995  0.18326  4.569 4.91e-06 ***
## Treatment4_Shipped-110Gy    0.97833   2.66001  0.18092  5.407 6.39e-08 ***
## ---
## Signif. codes:  0 '***' 0.001 '**' 0.01 '*' 0.05 '.' 0.1 ' ' 1
##
##                            exp(coef) exp(-coef) lower .95 upper .95
## Treatment2_Shipped-0Gy        0.9594     1.0423    0.6738     1.366
## Treatment3_Unshipped-110Gy    2.3099     0.4329    1.6129     3.308
## Treatment4_Shipped-110Gy      2.6600     0.3759    1.8659     3.792
##
## Concordance= 0.576  (se = 0.019 )
## Likelihood ratio test= 49.65  on 3 df,   p=1e-10
## Wald test            = 46.46  on 3 df,   p=5e-10
## Score (logrank) test = 48.83  on 3 df,   p=1e-10
```

Figure 7c and 7d: Survival trends- Kaplan Meier Analysis (km)

```
##Figure 7c: Comparing difference of trends between treatments(combined data)

data_1=read.csv("Figure 7c.csv")

#data_1
str(data_1)
```

```
## 'data.frame':    635 obs. of  7 variables:
##  $ day       : int  30 41 41 48 62 80 80 90 90 90 ...
##  $ Pupal_age : int  22 22 22 22 22 22 22 22 22 22 ...
##  $ Replicate : int  1 1 1 1 1 1 1 1 1 1 ...
##  $ Cohort    : int  1 1 1 1 1 1 1 1 1 1 ...
##  $ Treatments: chr  "Unshipped-0Gy" "Unshipped-0Gy" "Unshipped-0Gy"
"Unshipped-0Gy" ...
##  $ status    : int  1 1 1 1 1 1 1 1 1 1 ...
##  $ Treatment : chr  "1_Unshipped-0Gy" "1_Unshipped-0Gy" "1_Unshipped-0Gy"
"1_Unshipped-0Gy" ...
```

```
head(data_1)
```

```
##   day Pupal_age Replicate Cohort    Treatments status       Treatment
## 1  30        22         1      1 Unshipped-0Gy      1 1_Unshipped-0Gy
## 2  41        22         1      1 Unshipped-0Gy      1 1_Unshipped-0Gy
## 3  41        22         1      1 Unshipped-0Gy      1 1_Unshipped-0Gy
## 4  48        22         1      1 Unshipped-0Gy      1 1_Unshipped-0Gy
## 5  62        22         1      1 Unshipped-0Gy      1 1_Unshipped-0Gy
## 6  80        22         1      1 Unshipped-0Gy      1 1_Unshipped-0Gy

data_1=na.omit(data_1)
names(data_1)

## [1] "day"          "Pupal_age"   "Replicate"   "Cohort"      "Treatments"
## [6] "status"       "Treatment"

#Kaplan Meier Analysis (km)
km <- with(data_1, Surv(day, status))
head(km,115)

##   [1]  30  41  41  48  62  80  80  90  90  90   1  34  34  35  58  58  66
87
##  [19] 104 104  14  16  20  20  21  27  27  27  27  27  30  30  34  34  35
35
##  [37]  38  41  41  41  41  22  23  23  23  27  28  28  30  30  30  34  36
36
##  [55]  41  41   1  30  34  34  38  38  45  64  70  58  70  78  83  97  97
104
##  [73] 108  16  23  23  23  27  27  27  27  27  28  28  30  30  34  34  36
41
##  [91]  41  42  42  45   2  20  20  27  27  27  27  27  28  28  28  30  30
41
## [109]  41  20  21  38  38  50  55

km_trt_fit <- survfit(Surv(day, status) ~ Treatments, data=data_1)
plot(survfit(Surv(day,status)~Treatments,data=data_1), xlab = "Time(days)",
ylab = "Survival rate(22 and 29 day-old)",
col=c('red','blue','green','orange'), lwd=2, xlim =c(0, 115))
legend('topright', text.font = , cex=1, c("Control","Shipped",
"Irradiated","shipped and irradiated"), col=c('red','blue',
'green','orange'), lty = 1, lwd=2, box.lty = 1)
```

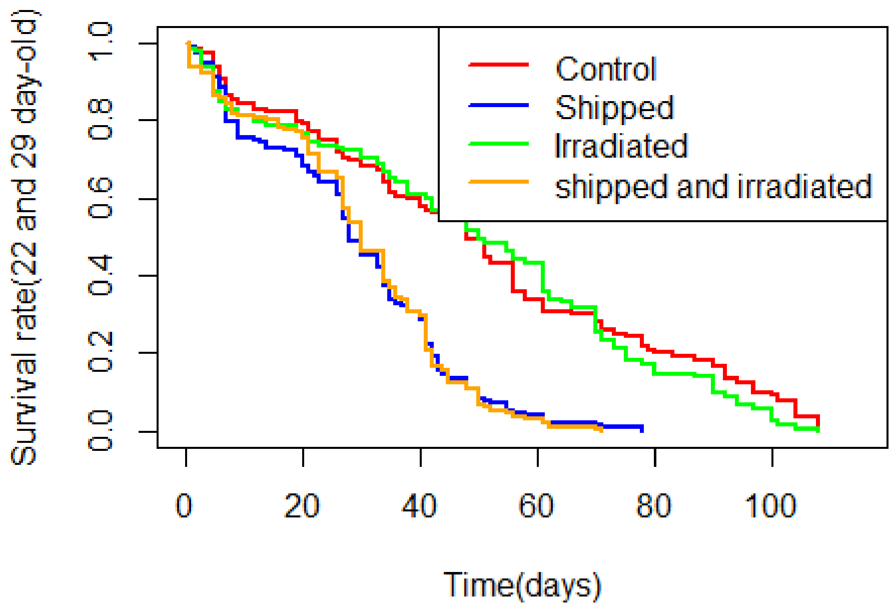


```
### survival: median Kaplan-Meier estimator
survfit(Surv(day,status)~Treatment,data=data_1)

## Call: survfit(formula = Surv(day, status) ~ Treatment, data = data_1)
##
##                                n events median 0.95LCL 0.95UCL
## Treatment=1_Unshipped-0Gy    144    144     50      42      61
## Treatment=2_Shipped-0Gy      139    139     48      40      56
## Treatment=3_Unshipped-110Gy 175    175     30      28      34
## Treatment=4_Shipped-110Gy    177    177     28      27      34

cox.surv <- coxph(Surv(day, status) ~ Treatment, data = data_1, method
="exact")
summary(cox.surv)

## Call:
## coxph(formula = Surv(day, status) ~ Treatment, data = data_1,
##     method = "exact")
##
##   n= 635, number of events= 635
##
##                                coef exp(coef) se(coef)      z Pr(>|z|)
## Treatment2_Shipped-0Gy     -0.1269    0.8808   0.1261 -1.006    0.314
## Treatment3_Unshipped-110Gy  1.0686    2.9114   0.1276  8.373   <2e-16 ***
## Treatment4_Shipped-110Gy    1.0664    2.9050   0.1264  8.436   <2e-16 ***
## ---
## Signif. codes:  0 '***' 0.001 '**' 0.01 '*' 0.05 '.' 0.1 ' ' 1
```

```
##
##                                exp(coef) exp(-coef) lower .95 upper .95
## Treatment2_Shipped-0Gy            0.8808     1.1353    0.6879     1.128
## Treatment3_Unshipped-110Gy        2.9114     0.3435    2.2671     3.739
## Treatment4_Shipped-110Gy          2.9050     0.3442    2.2675     3.722
##
## Concordance= 0.598  (se = 0.013 )
## Likelihood ratio test= 141.3  on 3 df,   p=<2e-16
## Wald test            = 131.5  on 3 df,   p=<2e-16
## Score (logrank) test = 140.9  on 3 df,   p=<2e-16
```

```
####Figure 7d: Comparing difference of trends between ages(combined data)


data_1=read.csv("Figure 7d.csv")
km <- with(data_1, Surv(day, status))
head(km,115)
```

```
##   [1]  30  41  41  48  62  80  80  90  90  90   1  34  34  35  58  58  66
87
##  [19] 104 104  14  16  20  20  21  27  27  27  27  27  30  30  34  34  35
35
##  [37]  38  41  41  41  41  22  23  23  23  27  28  28  30  30  30  34  36
36
##  [55]  41  41   1  30  34  34  38  38  45  64  70  58  70  78  83  97  97
104
##  [73] 108  16  23  23  23  27  27  27  27  27  28  28  30  30  34  34  36
41
##  [91]  41  42  42  45   2  20  20  27  27  27  27  27  28  28  28  30  30
41
## [109]  41  20  21  38  38  50  55
```

```
km_trt_fit <- survfit(Surv(day, status) ~ Pupal_age, data=data_1)
plot(survfit(Surv(day,status)~Pupal_age,data=data_1), xlab = "Time (days)",
ylab = "Survival rate", col=c('red','blue'), lwd=2, xlim =c(0, 115))
legend('topright', text.font = , cex=1, c("22 day-old pupae","29 day-old
pupae"), col=c('red','blue'), lty = 1, lwd=2, box.lty = 1)
```

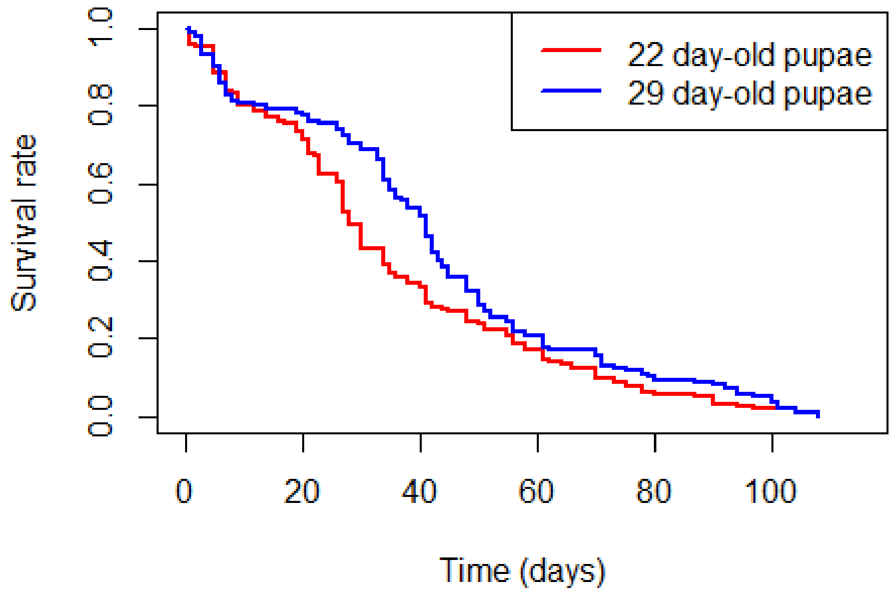


```
### survival: median Kaplan-Meier estimator
survfit(Surv(day,status)~Pupal_age,data=data_1)

## Call: survfit(formula = Surv(day, status) ~ Pupal_age, data = data_1)
##
##                   n events median 0.95LCL 0.95UCL
## Pupal_age=22 311    311     28      27      30
## Pupal_age=29 324    324     41      38      42

cox.surv <- coxph(Surv(day, status) ~ Pupal_age, data = data_1, method
="exact")
summary(cox.surv)

## Call:
## coxph(formula = Surv(day, status) ~ Pupal_age, data = data_1,
##     method = "exact")
##
##   n= 635, number of events= 635
##
##                coef exp(coef) se(coef)      z Pr(>|z|)
## Pupal_age -0.03882   0.96193  0.01187 -3.271  0.00107 **
## ---
## Signif. codes:  0 '***' 0.001 '**' 0.01 '*' 0.05 '.' 0.1 ' ' 1
##
##           exp(coef) exp(-coef) lower .95 upper .95
## Pupal_age    0.9619       1.04    0.9398    0.9846
##
```

```
## Concordance= 0.546  (se = 0.012 )
## Likelihood ratio test= 10.67  on 1 df,   p=0.001
## Wald test            = 10.7  on 1 df,   p=0.001
## Score (logrank) test = 10.76  on 1 df,   p=0.001
```

Fig 8:Survival at point in time-figures (Binomial method) Figure 8a:survival at different time points (15,30,60 days) for each treatment (22 days)

```
data_8a<- read.csv("Figure 8a.csv")
head(data_8a)

##        Treatments Pupal_age cohort rep  n surv_flies Surv_cutdays
## 1   Unshipped-0Gy        22      1   1 21         21       surv15
## 2     Shipped-0Gy        22      1   1 13         12       surv15
## 3 Unshipped-110Gy        22      1   1 21         20       surv15
## 4   Shipped-110Gy        22      1   1 15         15       surv15
## 5   Unshipped-0Gy        22      1   2 21         20       surv15
## 6     Shipped-0Gy        22      1   2 11         11       surv15
##           Treatment  X Time_point
## 1   1_Unshipped-0Gy NA         15
## 2     2_Shipped-0Gy NA         15
## 3 3_Unshipped-110Gy NA         15
## 4   4_Shipped-110Gy NA         15
## 5   1_Unshipped-0Gy NA         15
## 6     2_Shipped-0Gy NA         15

data_8a$Time_point<- as.factor(data_8a$Time_point)

Figure_8a<-ggplot(data_8a,aes(x=Time_point,y=surv_flies,fill=Time_point
))+geom_boxplot(position=position_dodge(0.8))+
  geom_jitter(position=position_dodge(0.8))+
  geom_boxplot(alpha=0.3) +
  labs(fill = "Time points(days)") +
  facet_wrap(~Treatments,ncol = 4) +
  theme_bw(base_size = 16)


Figure_8a
```

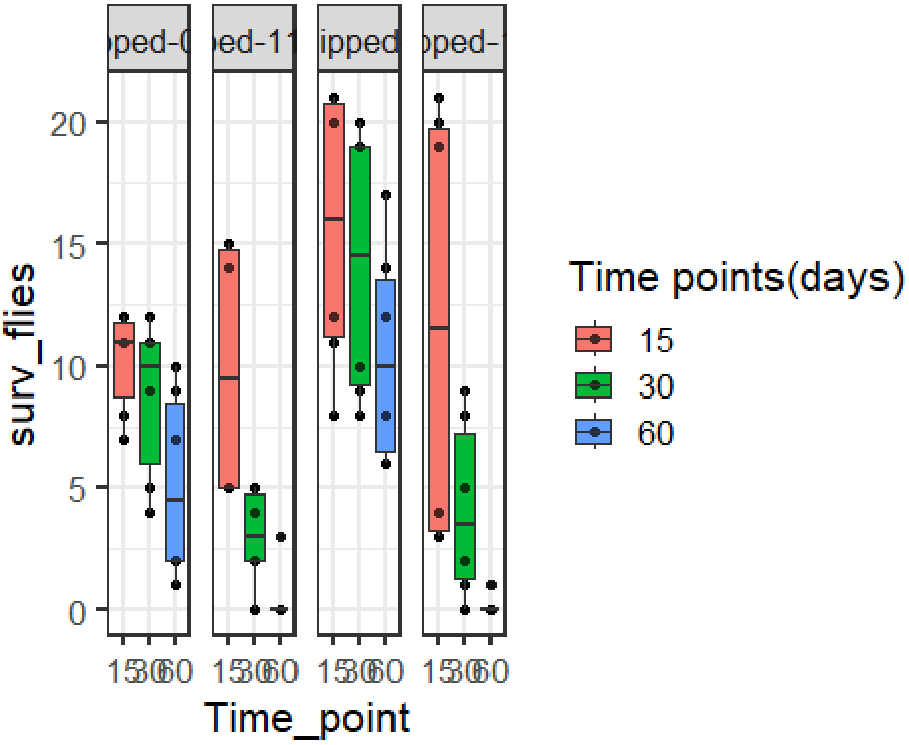


```
tiff("Figure 8a.tiff", width = 7, height = 4, units = 'in', compression =
'lzw',res = 300)
plot(Figure_8a+theme_tufte() + theme(axis.line = element_line(size = 1,
colour = "black")) + theme(legend.title = element_text(face = "bold")) +
theme(legend.text = element_text(face = "italic"))) + theme(axis.text.x =
element_blank()) + xlab(expression(bold("Time points (days)"))) +
ylab(expression(bold("No.of surviving flies (Age 22 days)")))
dev.off()

## png
##   2
```

Figure 8b:survival at different time points (15,30,60 days) for each treatment(29 days)

```
data_8b<- read.csv("Figure 8b.csv")
head(data_8b)

##        Treatments Pupal_age cohort rep  n surv_flies Surv_cutdays
## 1   Unshipped-0Gy        29      1   1 19         17       surv15
## 2     Shipped-0Gy        29      1   1 19         18       surv15
## 3 Unshipped-110Gy        29      1   1 23         20       surv15
## 4   Shipped-110Gy        29      1   1 20         15       surv15
## 5   Unshipped-0Gy        29      1   2 19         13       surv15
## 6     Shipped-0Gy        29      1   2 19         16       surv15
##           Treatment  X Time_point
## 1   1_Unshipped-0Gy NA         15
## 2     2_Shipped-0Gy NA         15
```

```
## 3 3_Unshipped-110Gy NA         15
## 4  4_Shipped-110Gy  NA         15
## 5   1_Unshipped-0Gy NA         15
## 6     2_Shipped-0Gy NA         15

data_8b$Time_point<- as.factor(data_8b$Time_point)

Figure_8b<-ggplot(data_8b,aes(x=Time_point,y=surv_flies,fill=Time_point
))+geom_boxplot(position=position_dodge(0.8))+
  geom_jitter(position=position_dodge(0.8))+
  geom_boxplot(alpha=0.3) +
  labs(fill = "Time points(days)") +
  facet_wrap(~Treatments,ncol = 4) +
  theme_bw(base_size = 16)

Figure_8b
```

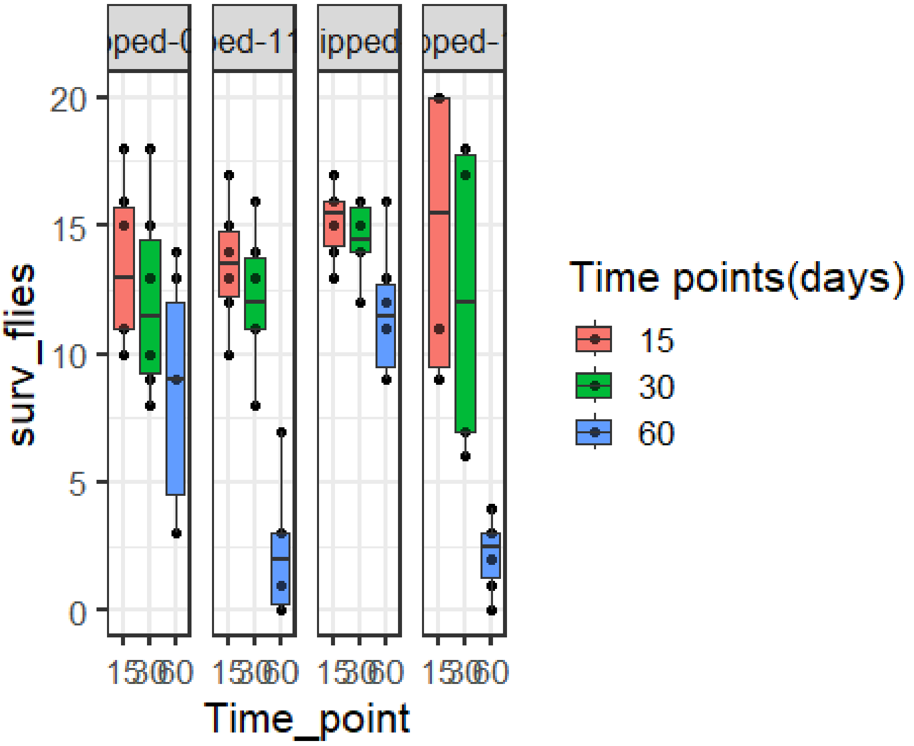


```
tiff("Figure 8b.tiff", width = 7, height = 4, units = 'in',compression =
'lzw', res = 300)
plot(Figure_8b+theme_tufte() + theme(axis.line = element_line(size = 1,
colour = "black")) + theme(legend.title = element_text(face = "bold")) +
theme(legend.text = element_text(face = "italic")) + theme(axis.text.x =
element_blank()) + xlab(expression(bold("Time points (days)"))) +
ylab(expression(bold("No.of surviving flies (Age 29 days) ")))
dev.off()
```

```
## png
##   2
```

Figure 8c:survival at different time points (15,30,60 days) for each treatment (Combined data(22 and 29 days))

```
data_8c<- read.csv("Figure 8c.csv")
head(data_8c)

##        Treatments Pupal_age cohort rep  n Surv_cutdays surv_flies
## 1   Unshipped-0Gy        22      1   1 21      surv_15         21
## 2     Shipped-0Gy        22      1   1 13      surv_15         12
## 3 Unshipped-110Gy        22      1   1 21      surv_15         20
## 4   Shipped-110Gy        22      1   1 15      surv_15         15
## 5   Unshipped-0Gy        22      1   2 21      surv_15         20
## 6     Shipped-0Gy        22      1   2 11      surv_15         11
##           Treatment  X Time_point
## 1   1_Unshipped-0Gy NA         15
## 2     2_Shipped-0Gy NA         15
## 3 3_Unshipped-110Gy NA         15
## 4   4_Shipped-110Gy NA         15
## 5   1_Unshipped-0Gy NA         15
## 6     2_Shipped-0Gy NA         15

data_8c$Time_point<- as.factor(data_8c$Time_point)


Figure_8c<-ggplot(data_8c,aes(x=Time_point,y=surv_flies,fill=Time_point
))+geom_boxplot(position=position_dodge(0.8))+
  geom_jitter(position=position_dodge(0.8))+
  geom_boxplot(alpha=0.3) +
  labs(fill = "Time points(days)") +
  facet_wrap(~Treatments,ncol = 4) +
  theme_bw(base_size = 16)

Figure_8c
```

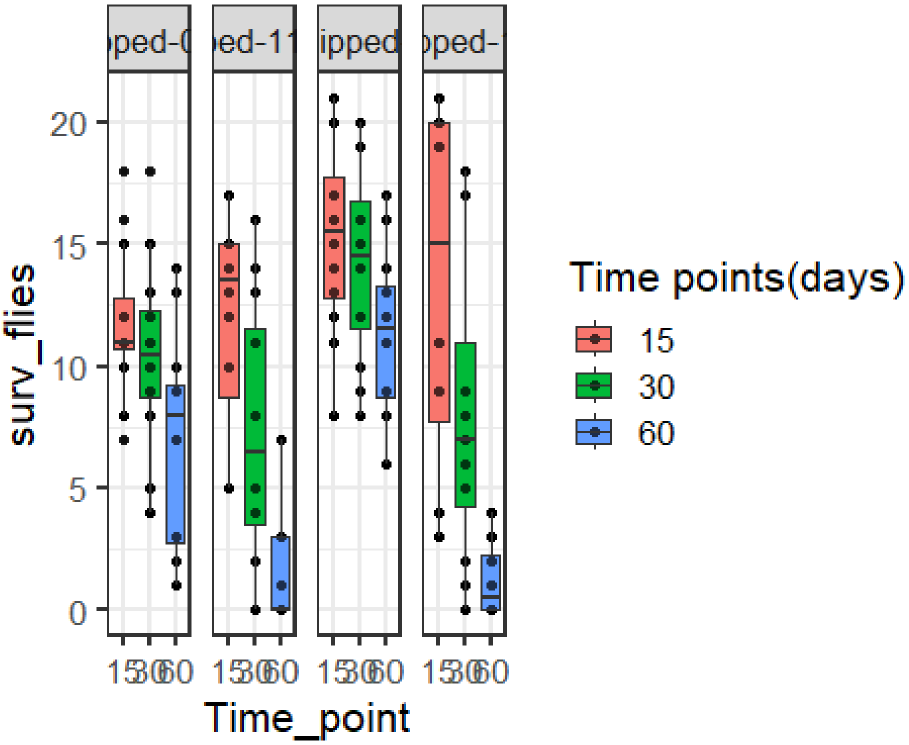


```
tiff("Figure 8c.tiff", width = 7, height = 4, units = 'in', res = 300)
plot(Figure_8c+theme_tufte() + theme(axis.line = element_line(size = 1,
colour = "black")) + theme(legend.title = element_text(face = "bold")) +
theme(legend.text = element_text(face = "italic"))) + theme(axis.text.x =
element_blank()) + xlab(expression(bold("Time points (days)"))) +
ylab(expression(bold("No. of surviving flies (Age 22 and 29 days)")))
dev.off()

## png
##   2
```

Figure 8d: survival at different time points- differences between Pupal_age -Regardless of treatment

```
data_8d<- read.csv("Figure 8d.csv")
head(data_8d)

##        Treatments Pupal_age cohort rep  n Surv_cutdays surv_flies
## 1   Unshipped-0Gy        22      1   1 21      surv_15         21
## 2     Shipped-0Gy        22      1   1 13      surv_15         12
## 3 Unshipped-110Gy        22      1   1 21      surv_15         20
## 4   Shipped-110Gy        22      1   1 15      surv_15         15
## 5   Unshipped-0Gy        22      1   2 21      surv_15         20
## 6     Shipped-0Gy        22      1   2 11      surv_15         11
##           Treatment  X Time_point
## 1   1_Unshipped-0Gy NA         15
## 2     2_Shipped-0Gy NA         15
```

```
## 3 3_Unshipped-110Gy NA         15
## 4  4_Shipped-110Gy  NA         15
## 5   1_Unshipped-0Gy NA         15
## 6     2_Shipped-0Gy NA         15

data_8d$Pupal_age<- as.factor(data_8d$Pupal_age)
data_8d$Time_point<- as.factor(data_8d$Time_point)

Figure_8d<-ggplot(data_8d,aes(x=Time_point,y=surv_flies,fill=Time_point))+
  geom_boxplot(alpha=0.3) +
  labs(fill = "Time points(days)") +
  facet_wrap(~Pupal_age) +theme_bw(base_size = 16)

Figure_8d
```

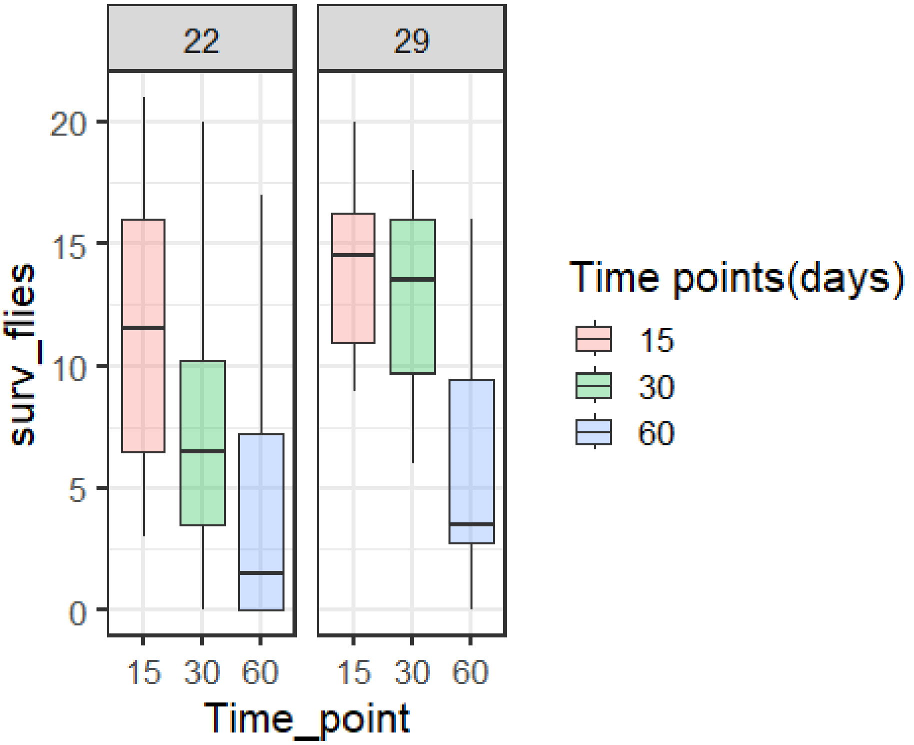


```
tiff("Figure 8d.tiff", width = 7, height = 4, units = 'in', compression =
'lzw',res = 300)
plot(Figure_8d+theme_tufte() + theme(axis.line = element_line(size = 1,
colour = "black")) + theme(legend.title = element_text(face = "bold")) +
theme(legend.text = element_text(face = "italic"))) + theme(axis.text.x =
element_blank()) + xlab(expression(bold("Time points (days)"))) +
ylab(expression(bold("No. of surviving flies (Age 22 and 29 days) ")))
dev.off()

## png
##   2
```

Figure 8: Survival at point in time(####Significant Values)-Binomial method

Figure 8a:survival point in time -22 days

```
data_4<- read.csv("Figure 8a_22.csv")
head(data_4)

##         Treatments cohort rep  n surv15 surv30 surv60         Treatment
## 1    Unshipped-0Gy      1   1 21     21     20     17    1_Unshipped-0Gy
## 2      Shipped-0Gy      1   1 13     12     12      7      2_Shipped-0Gy
## 3 Unshipped-110Gy      1   1 21     20      9      0 3_Unshipped-110Gy
## 4   Shipped-110Gy      1   1 15     15      5      0   4_Shipped-110Gy
## 5    Unshipped-0Gy      1   2 21     20     19     14    1_Unshipped-0Gy
## 6      Shipped-0Gy      1   2 11     11     11     10      2_Shipped-0Gy

boxplot(data_4$surv15 ~ data_4$Treatments, ylab = "Survival rate after 15
days_22")
```

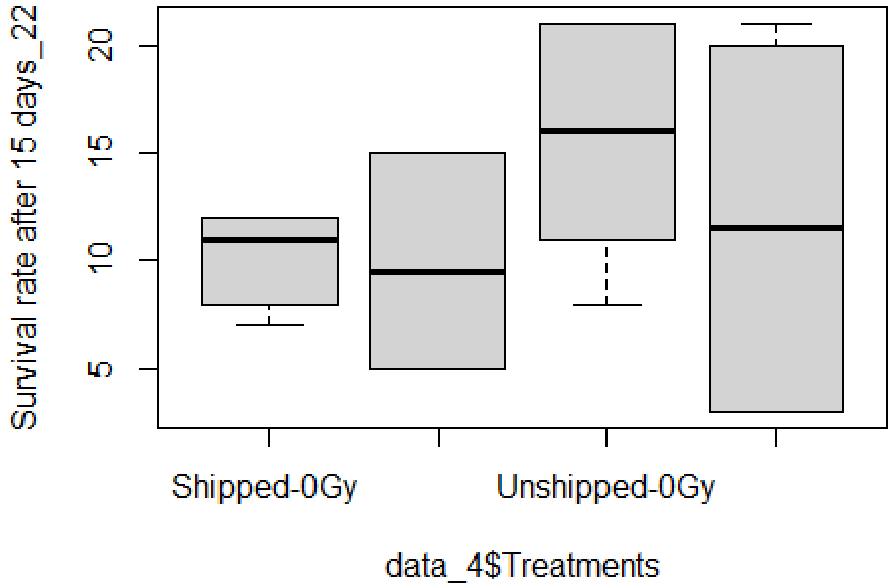


```
boxplot(data_4$surv30 ~ data_4$Treatments, ylab = "Survival rate after
30days_22")
```

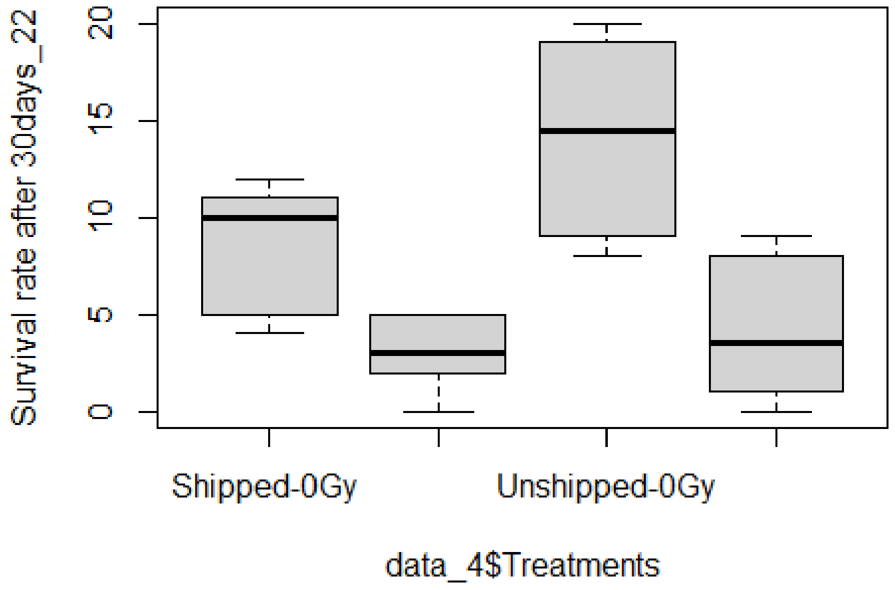


```
boxplot(data_4$surv60 ~ data_4$Treatments, ylab = "Survival rate after 60
days_22")
```

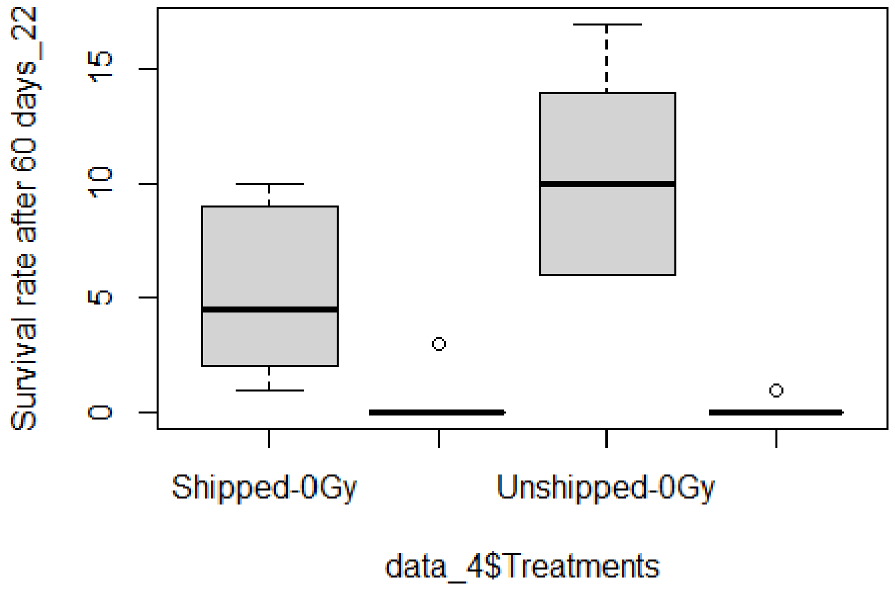

```r
## survival: binomial model survival rate 15 days(difference in treatments)
fm4_1 <- glmer(cbind(surv15,n - surv15) ~ Treatment +(1|rep), family =
binomial, data = data_4)

## boundary (singular) fit: see help('isSingular')

summary(fm4_1)

## Generalized linear mixed model fit by maximum likelihood (Laplace
##   Approximation) [glmerMod]
##  Family: binomial  ( logit )
## Formula: cbind(surv15, n - surv15) ~ Treatment + (1 | rep)
##    Data: data_4
##
##      AIC      BIC   logLik deviance df.resid
##    184.8    190.7    -87.4    174.8       19
##
## Scaled residuals:
##     Min      1Q  Median      3Q     Max
## -3.6708 -2.3353  0.5123  1.8412  2.8460
##
## Random effects:
##  Groups Name        Variance Std.Dev.
##  rep    (Intercept) 0        0
## Number of obs: 24, groups:  rep, 3
##
## Fixed effects:
##                           Estimate Std. Error z value Pr(>|z|)
## (Intercept)                 1.8245     0.2782   6.557 5.48e-11 ***
## Treatment2_Shipped-0Gy      0.0891     0.4527   0.197   0.8440
## Treatment3_Unshipped-110Gy  -0.8719     0.3588  -2.430   0.0151 *
## Treatment4_Shipped-110Gy    -0.8825     0.3713  -2.377   0.0175 *
## ---
## Signif. codes:  0 '***' 0.001 '**' 0.01 '*' 0.05 '.' 0.1 ' ' 1
##
## Correlation of Fixed Effects:
##             (Intr) T2_S-0 T3_U-1
## Trtmn2_S-0G -0.615
## Trt3_U-110G -0.775  0.477
## Trt4_S-110G -0.749  0.461  0.581
## optimizer (Nelder_Mead) convergence code: 0 (OK)
## boundary (singular) fit: see help('isSingular')

## survival: binomial model survival rate 30 days(difference in treatments)
fm4_2 <- glmer(cbind(surv30,n - surv30) ~ Treatment +(1|rep), family =
binomial, data = data_4)
summary(fm4_2)

## Generalized linear mixed model fit by maximum likelihood (Laplace
##   Approximation) [glmerMod]
##  Family: binomial  ( logit )
```

```
## Formula: cbind(surv30, n - surv30) ~ Treatment + (1 | rep)
##    Data: data_4
##
##      AIC      BIC   logLik deviance df.resid
##    129.6    135.5    -59.8    119.6       19
##
## Scaled residuals:
##     Min      1Q  Median      3Q     Max
## -2.6211 -1.5933  0.5059  1.2100  1.8077
##
## Random effects:
##  Groups Name        Variance Std.Dev.
##  rep    (Intercept) 0.08564  0.2926
## Number of obs: 24, groups:  rep, 3
##
## Fixed effects:
##                            Estimate Std. Error z value Pr(>|z|)
## (Intercept)                  1.3315     0.2919   4.562 5.07e-06 ***
## Treatment2_Shipped-0Gy      -0.2531     0.3636  -0.696    0.486
## Treatment3_Unshipped-110Gy  -2.4121     0.3360  -7.179 7.00e-13 ***
## Treatment4_Shipped-110Gy    -2.6201     0.3610  -7.259 3.90e-13 ***
## ---
## Signif. codes:  0 '***' 0.001 '**' 0.01 '*' 0.05 '.' 0.1 ' ' 1
##
## Correlation of Fixed Effects:
##             (Intr) T2_S-0 T3_U-1
## Trtmn2_S-0G -0.530
## Trt3_U-110G -0.583  0.461
## Trt4_S-110G -0.543  0.429  0.479
```

```
## survival: binomial model survival rate 60 days
fm4_3<- glmer(cbind(surv60,n - surv60) ~ Treatment +(1|rep), family =
binomial, data = data_4)
```

```
## boundary (singular) fit: see help('isSingular')
```

```
summary(fm4_3)
```

```
## Generalized linear mixed model fit by maximum likelihood (Laplace
##   Approximation) [glmerMod]
##  Family: binomial  ( logit )
## Formula: cbind(surv60, n - surv60) ~ Treatment + (1 | rep)
##    Data: data_4
##
##      AIC      BIC   logLik deviance df.resid
##    104.9    110.8    -47.5     94.9       19
##
## Scaled residuals:
##     Min      1Q  Median      3Q     Max
## -2.3500 -0.7547 -0.4677  0.7141  3.9378
##
```

```
## Random effects:
##  Groups Name        Variance Std.Dev.
##  rep    (Intercept) 0        0
## Number of obs: 24, groups:  rep, 3
##
## Fixed effects:
##                            Estimate Std. Error z value Pr(>|z|)
## (Intercept)                  0.3365     0.1952   1.724   0.0847 .
## Treatment2_Shipped-0Gy      -0.5660     0.3098  -1.827   0.0677 .
## Treatment3_Unshipped-110Gy  -4.9008     1.0240  -4.786 1.70e-06 ***
## Treatment4_Shipped-110Gy    -3.6073     0.6197  -5.821 5.86e-09 ***
## ---
## Signif. codes:  0 '***' 0.001 '**' 0.01 '*' 0.05 '.' 0.1 ' ' 1
##
## Correlation of Fixed Effects:
##             (Intr) T2_S-0 T3_U-1
## Trtmn2_S-0G -0.630
## Trt3_U-110G -0.191  0.120
## Trt4_S-110G -0.315  0.198  0.060
## optimizer (Nelder_Mead) convergence code: 0 (OK)
## boundary (singular) fit: see help('isSingular')
```

Figure 8a:survival point in time -29 days

```
data_5<- read.csv("Figure 8a_29.csv")
head(data_5)
```

```
##        Treatments cohort rep  n surv15 surv30 surv60         Treatment
## 1   Unshipped-0Gy      1   1 19     17     16     13   1_Unshipped-0Gy
## 2     Shipped-0Gy      1   1 19     18     18     14     2_Shipped-0Gy
## 3 Unshipped-110Gy      1   1 23     20     17      1 3_Unshipped-110Gy
## 4   Shipped-110Gy      1   1 20     15     14      1   4_Shipped-110Gy
## 5   Unshipped-0Gy      1   2 19     13     12     11   1_Unshipped-0Gy
## 6     Shipped-0Gy      1   2 19     16     15     13     2_Shipped-0Gy
```

```
boxplot(data_5$surv15/data_5$n ~ data_5$Treatments, ylab = "Survival rate
after 15 days_29")
```

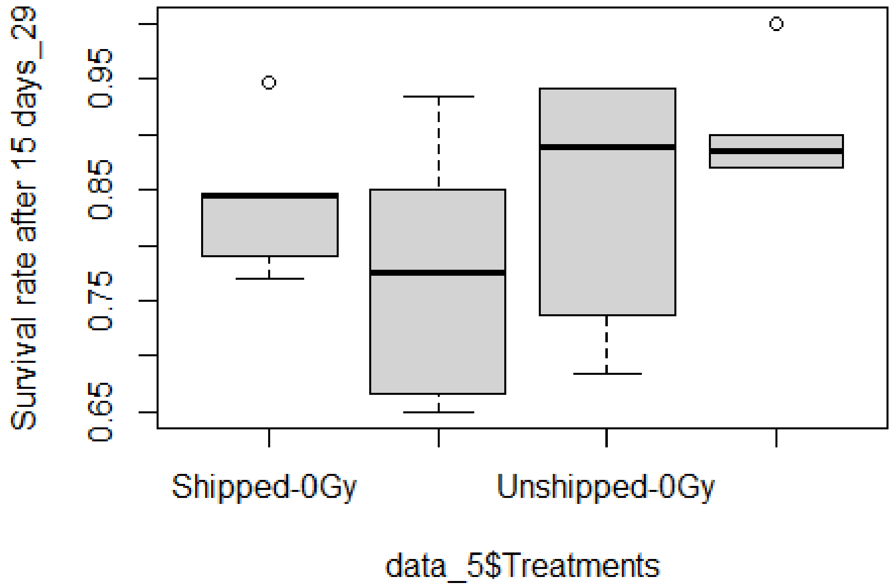


```
boxplot(data_5$surv30/data_5$n ~ data_5$Treatments, ylab = "Survival rate
after 30 days_29")
```

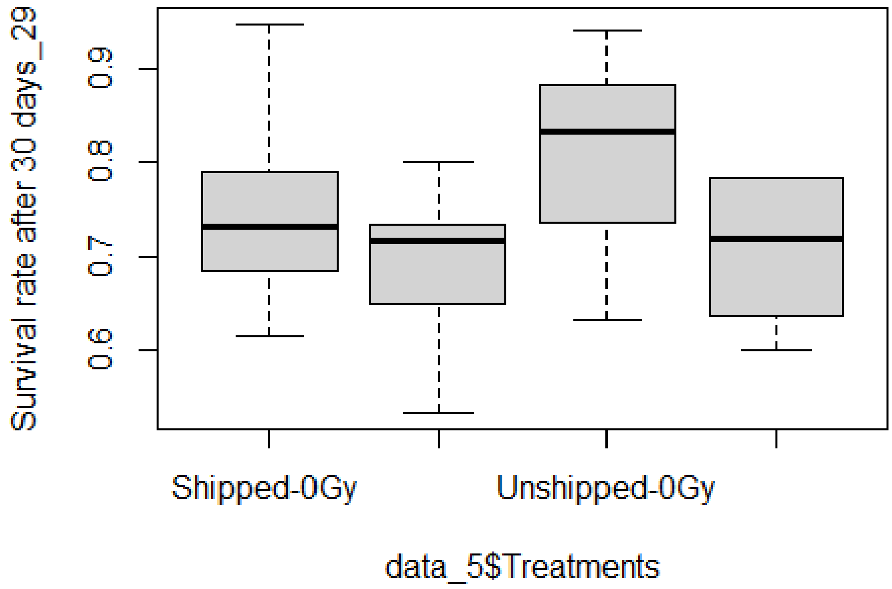

```r
boxplot(data_5$surv60/data_5$n ~ data_5$Treatments, ylab = "Survival rate after 60 days_29")
```

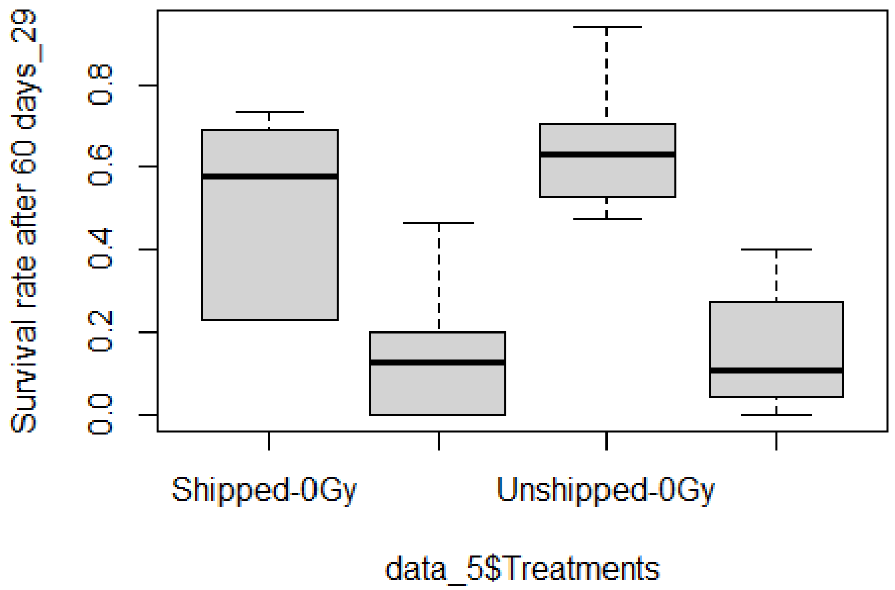


```r
### survival: binomial model survival rate 15 days (difference in treatments)
fm5_1 <- glmer(cbind(surv15,n - surv15) ~ Treatment +(1|rep), family = binomial, data = data_5)

## boundary (singular) fit: see help('isSingular')

summary(fm5_1)

## Generalized linear mixed model fit by maximum likelihood (Laplace
##   Approximation) [glmerMod]
##  Family: binomial  ( logit )
## Formula: cbind(surv15, n - surv15) ~ Treatment + (1 | rep)
##    Data: data_5
##
##      AIC      BIC   logLik deviance df.resid
##     90.3     96.2    -40.1     80.3       19
##
## Scaled residuals:
##      Min       1Q   Median       3Q      Max
## -1.89566 -0.39781  0.02387  0.67728  1.49330
##
## Random effects:
##  Groups Name        Variance Std.Dev.
##  rep    (Intercept) 0        0
```

```
## Number of obs: 24, groups:  rep, 3
##
## Fixed effects:
##                           Estimate Std. Error z value Pr(>|z|)
## (Intercept)               1.677646   0.264221   6.349 2.16e-10 ***
## Treatment2_Shipped-0Gy    0.008753   0.385778   0.023    0.982
## Treatment3_Unshipped-110Gy 0.413095   0.414678   0.996    0.319
## Treatment4_Shipped-110Gy  -0.461251   0.351888  -1.311    0.190
## ---
## Signif. codes:  0 '***' 0.001 '**' 0.01 '*' 0.05 '.' 0.1 ' ' 1
##
## Correlation of Fixed Effects:
##             (Intr) T2_S-0 T3_U-1
## Trtmn2_S-0G -0.685
## Trt3_U-110G -0.637  0.436
## Trt4_S-110G -0.751  0.514  0.478
## optimizer (Nelder_Mead) convergence code: 0 (OK)
## boundary (singular) fit: see help('isSingular')
```

```
### survival: binomial model survival rate 30 days(difference in treatments)
fm5_2 <- glmer(cbind(surv30,n - surv30) ~ Treatment +(1|rep), family =
binomial, data = data_5)
```

```
## boundary (singular) fit: see help('isSingular')
```

```
summary(fm5_2)
```

```
## Generalized linear mixed model fit by maximum likelihood (Laplace
##   Approximation) [glmerMod]
##  Family: binomial  ( logit )
## Formula: cbind(surv30, n - surv30) ~ Treatment + (1 | rep)
##    Data: data_5
##
##      AIC      BIC   logLik deviance df.resid
##    100.0    105.9    -45.0     90.0       19
##
## Scaled residuals:
##      Min       1Q   Median       3Q      Max
## -1.91612 -0.71383  0.08654  0.44398  1.90920
##
## Random effects:
##  Groups Name        Variance Std.Dev.
##  rep    (Intercept) 0        0
## Number of obs: 24, groups:  rep, 3
##
## Fixed effects:
##                          Estimate Std. Error z value Pr(>|z|)
## (Intercept)                1.4214     0.2431   5.846 5.03e-09 ***
## Treatment2_Shipped-0Gy    -0.2664     0.3410  -0.781   0.4346
## Treatment3_Unshipped-110Gy -0.4268     0.3314  -1.288   0.1979
## Treatment4_Shipped-110Gy  -0.5967     0.3226  -1.850   0.0644 .
```

```
## ---
## Signif. codes:  0 '***' 0.001 '**' 0.01 '*' 0.05 '.' 0.1 ' ' 1
##
## Correlation of Fixed Effects:
##             (Intr) T2_S-0 T3_U-1
## Trtmn2_S-0G -0.713
## Trt3_U-110G -0.734  0.523
## Trt4_S-110G -0.754  0.537  0.553
## optimizer (Nelder_Mead) convergence code: 0 (OK)
## boundary (singular) fit: see help('isSingular')
```

```
### survival: binomial model survival rate 60 days(difference in treatments)
fm5_3 <- glmer(cbind(surv60,n - surv60) ~ Treatment +(1|rep), family =
binomial, data = data_5)
summary(fm5_3)
```

```
## Generalized linear mixed model fit by maximum likelihood (Laplace
##   Approximation) [glmerMod]
##  Family: binomial  ( logit )
## Formula: cbind(surv60, n - surv60) ~ Treatment + (1 | rep)
##    Data: data_5
##
##      AIC      BIC   logLik deviance df.resid
##    134.5    140.4    -62.2    124.5       19
##
## Scaled residuals:
##     Min      1Q  Median      3Q     Max
## -2.1852 -1.2276 -0.2319  1.2050  3.8221
##
## Random effects:
##  Groups Name        Variance Std.Dev.
##  rep    (Intercept) 0.001363 0.03692
## Number of obs: 24, groups:  rep, 3
##
## Fixed effects:
##                            Estimate Std. Error z value Pr(>|z|)
## (Intercept)                  0.6111     0.2027   3.015  0.00257 **
## Treatment2_Shipped-0Gy      -0.4859     0.2872  -1.692  0.09066 .
## Treatment3_Unshipped-110Gy  -2.5125     0.3596  -6.987 2.81e-12 ***
## Treatment4_Shipped-110Gy    -2.4834     0.3512  -7.070 1.55e-12 ***
## ---
## Signif. codes:  0 '***' 0.001 '**' 0.01 '*' 0.05 '.' 0.1 ' ' 1
##
## Correlation of Fixed Effects:
##             (Intr) T2_S-0 T3_U-1
## Trtmn2_S-0G -0.698
## Trt3_U-110G -0.558  0.394
## Trt4_S-110G -0.572  0.403  0.324
```

Survival at point in time-Combined data(22 and 29 days) -Figure 8c and 8d
###significance

```
data_4<- read.csv("Figure 8c_8d_significance.csv")
str(data_4)

## 'data.frame':    48 obs. of  9 variables:
##  $ Treatments: chr  "Unshipped-0Gy" "Shipped-0Gy " "Unshipped-110Gy"
"Shipped-110Gy " ...
##  $ Pupal_age : int  22 22 22 22 22 22 22 22 22 22 ...
##  $ cohort    : int  1 1 1 1 1 1 1 1 1 1 ...
##  $ rep       : int  1 1 1 1 2 2 2 2 3 3 ...
##  $ n         : int  21 13 21 15 21 11 21 15 21 12 ...
##  $ surv15    : int  21 12 20 15 20 11 21 14 21 11 ...
##  $ surv30    : int  20 12 9 5 19 11 8 2 19 9 ...
##  $ surv60    : int  17 7 0 0 14 10 0 0 12 9 ...
##  $ Treatment : chr  "1_Unshipped-0Gy" "2_Shipped-0Gy" "3_Unshipped-110Gy"
"4_Shipped-110Gy " ...

summary(data_4)

##   Treatments          Pupal_age        cohort         rep           n
##  Length:48          Min.   :22.0   Min.   :1.0   Min.   :1   Min.   :10.00
##  Class :character   1st Qu.:22.0   1st Qu.:1.0   1st Qu.:1   1st Qu.:12.00
##  Mode  :character   Median :25.5   Median :1.5   Median :2   Median :15.00
##                     Mean   :25.5   Mean   :1.5   Mean   :2   Mean   :15.96
##                     3rd Qu.:29.0   3rd Qu.:2.0   3rd Qu.:3   3rd Qu.:19.25
##                     Max.   :29.0   Max.   :2.0   Max.   :3   Max.   :23.00
##      surv15          surv30          surv60         Treatment
##  Min.   : 3.00   Min.   : 0.00   Min.   : 0.000   Length:48
##  1st Qu.:10.00   1st Qu.: 5.75   1st Qu.: 0.000   Class :character
##  Median :13.00   Median :10.00   Median : 3.000   Mode  :character
##  Mean   :13.02   Mean   :10.12   Mean   : 5.125
##  3rd Qu.:16.25   3rd Qu.:14.25   3rd Qu.: 9.000
##  Max.   :21.00   Max.   :20.00   Max.   :17.000

head(data_4)

##        Treatments Pupal_age cohort rep  n surv15 surv30 surv60
## 1   Unshipped-0Gy        22      1   1 21     21     20     17
## 2     Shipped-0Gy        22      1   1 13     12     12      7
## 3 Unshipped-110Gy        22      1   1 21     20      9      0
## 4   Shipped-110Gy        22      1   1 15     15      5      0
## 5   Unshipped-0Gy        22      1   2 21     20     19     14
## 6     Shipped-0Gy        22      1   2 11     11     11     10
##           Treatment
## 1   1_Unshipped-0Gy
## 2     2_Shipped-0Gy
## 3 3_Unshipped-110Gy
## 4  4_Shipped-110Gy
```

```
## 5   1_Unshipped-0Gy
## 6     2_Shipped-0Gy

#### Individual boxplots for Figure 8c and 8d

Survival_15days<-data_4$surv15

boxplot(Survival_15days ~ data_4$Treatment, ylab = "Survival rate after 15
days (combined data)")
```

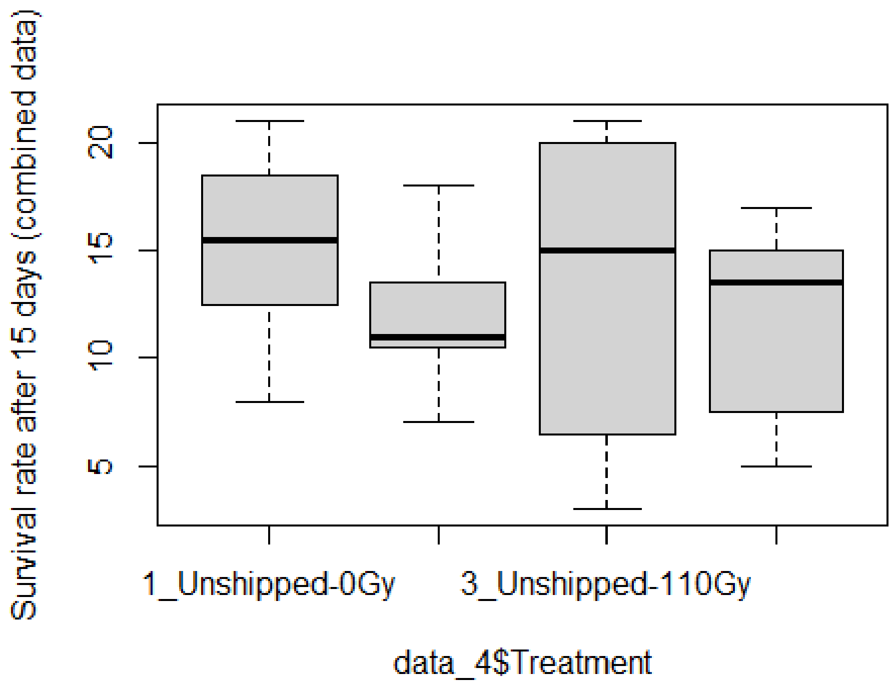


```
boxplot(Survival_15days ~ data_4$Pupal_age, ylab = "Survival rate after 15
days")
```

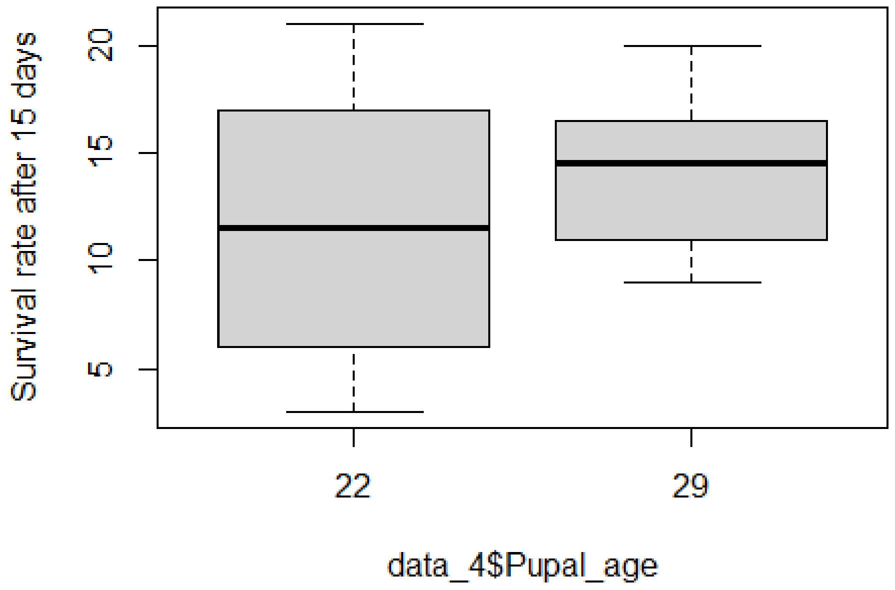


```
#30
boxplot(data_4$surv30/data_4$n ~ data_4$Treatment, ylab = "Survival rate
after 30 days (combined data)")
```

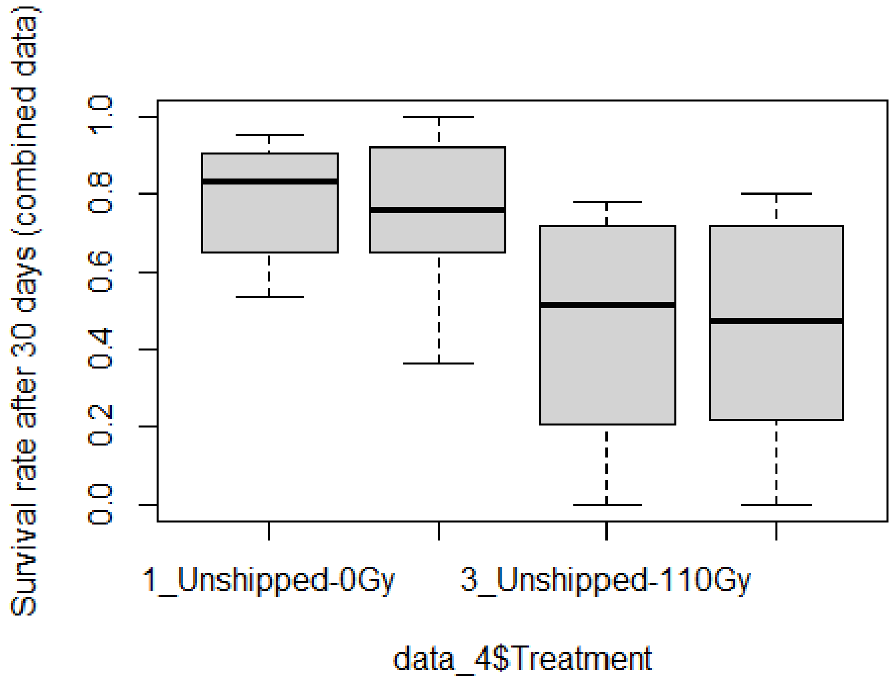

```r
boxplot(data_4$surv30/data_4$n ~ data_4$Pupal_age, ylab = "Survival rate
after 30 days")
```

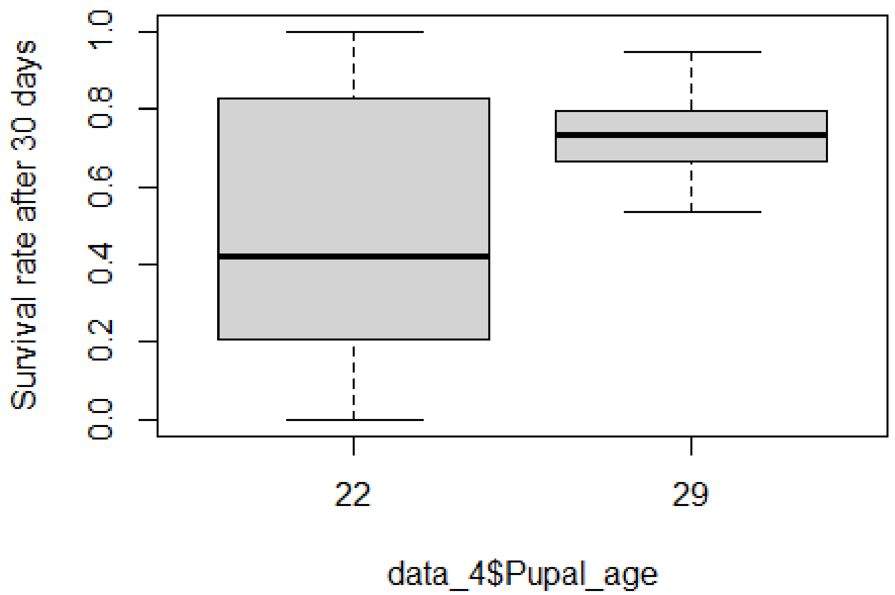


```r
#60
boxplot(data_4$surv60/data_4$n ~ data_4$Treatment, ylab = "Survival rate
after 60 days(combined data)")
```

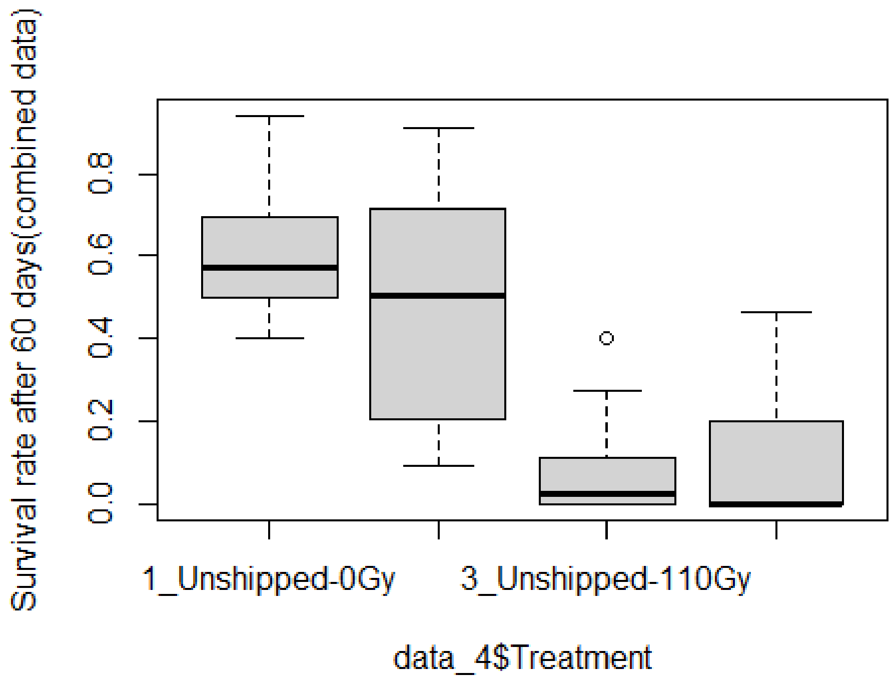


```
boxplot(data_4$surv60/data_4$n ~ data_4$Pupal_age, ylab = "Survival rate
after 60 days")
```

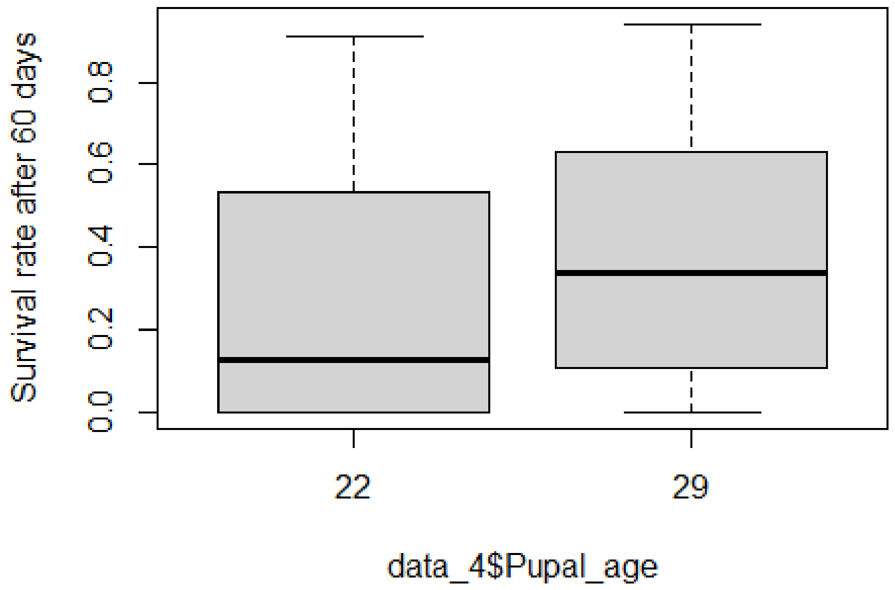

```
#### significance (boxplots above) for Figure 8c and 8d

########################## survival: binomial model survival rate 15 days


###Treatments
fm4_1 <- glmer(cbind(surv15,n - surv15) ~ Treatment +(1|rep), family =
binomial, data = data_4)
summary(fm4_1)

## Generalized linear mixed model fit by maximum likelihood (Laplace
##   Approximation) [glmerMod]
##  Family: binomial  ( logit )
## Formula: cbind(surv15, n - surv15) ~ Treatment + (1 | rep)
##    Data: data_4
##
##      AIC      BIC   logLik deviance df.resid
##    275.2    284.5   -132.6    265.2       43
##
## Scaled residuals:
##     Min      1Q  Median      3Q     Max
## -4.9552 -0.9727  0.6103  1.2290  2.3145
##
## Random effects:
##  Groups Name        Variance Std.Dev.
##  rep    (Intercept) 0.008649 0.093
## Number of obs: 48, groups:  rep, 3
##
## Fixed effects:
##                             Estimate Std. Error z value Pr(>|z|)
## (Intercept)                  1.75224    0.19922   8.796  < 2e-16 ***
## Treatment2_Shipped-0Gy       0.02848    0.29233   0.097  0.92240
## Treatment3_Unshipped-110Gy -0.31804    0.26335  -1.208  0.22716
## Treatment4_Shipped-110Gy   -0.65825    0.25529  -2.578  0.00993 **
## ---
## Signif. codes:  0 '***' 0.001 '**' 0.01 '*' 0.05 '.' 0.1 ' ' 1
##
## Correlation of Fixed Effects:
##             (Intr) T2_S-0 T3_U-1
## Trtmn2_S-0G -0.630
## Trt3_U-110G -0.700  0.477
## Trt4_S-110G -0.722  0.492  0.546

###Pupal age

fm4_1 <- glmer(cbind(surv15,n - surv15) ~ Pupal_age +(1|rep), family =
binomial, data = data_4)
summary(fm4_1)
```

```
## Generalized linear mixed model fit by maximum likelihood (Laplace
##   Approximation) [glmerMod]
##  Family: binomial  ( logit )
## Formula: cbind(surv15, n - surv15) ~ Pupal_age + (1 | rep)
##    Data: data_4
##
##      AIC      BIC   logLik deviance df.resid
##    277.9    283.5   -135.9    271.9       45
##
## Scaled residuals:
##     Min      1Q  Median      3Q     Max
## -4.7004 -1.0567  0.3759  1.2843  2.4175
##
## Random effects:
##  Groups Name        Variance Std.Dev.
##  rep    (Intercept) 0.008696 0.09325
## Number of obs: 48, groups:  rep, 3
##
## Fixed effects:
##             Estimate Std. Error z value Pr(>|z|)
## (Intercept)  0.43507    0.68723   0.633    0.527
## Pupal_age    0.04132    0.02670   1.547    0.122
##
## Correlation of Fixed Effects:
##           (Intr)
## Pupal_age -0.988
```

```
########################### survival: binomial model survival rate 30 days

###Treatments
fm4_2 <- glmer(cbind(surv30,n - surv30) ~ Treatment +(1|rep), family =
binomial, data = data_4)
summary(fm4_2)
```

```
## Generalized linear mixed model fit by maximum likelihood (Laplace
##   Approximation) [glmerMod]
##  Family: binomial  ( logit )
## Formula: cbind(surv30, n - surv30) ~ Treatment + (1 | rep)
##    Data: data_4
##
##      AIC      BIC   logLik deviance df.resid
##    310.6    320.0   -150.3    300.6       43
##
## Scaled residuals:
##     Min      1Q  Median      3Q     Max
## -3.2935 -1.5704  0.1795  1.3858  3.0483
##
## Random effects:
##  Groups Name        Variance Std.Dev.
##  rep    (Intercept) 0.01745  0.1321
```

```
## Number of obs: 48, groups:  rep, 3
##
## Fixed effects:
##                             Estimate Std. Error z value Pr(>|z|)
## (Intercept)                   1.3684     0.1857   7.369 1.72e-13 ***
## Treatment2_Shipped-0Gy       -0.2498     0.2472  -1.011    0.312
## Treatment3_Unshipped-110Gy  -1.3785     0.2215  -6.223 4.87e-10 ***
## Treatment4_Shipped-110Gy    -1.4215     0.2240  -6.347 2.20e-10 ***
## ---
## Signif. codes:  0 '***' 0.001 '**' 0.01 '*' 0.05 '.' 0.1 ' ' 1
##
## Correlation of Fixed Effects:
##               (Intr) T2_S-0 T3_U-1
## Trtmn2_S-0G -0.624
## Trt3_U-110G -0.697  0.523
## Trt4_S-110G -0.689  0.517  0.578
```

```
###Pupal age

fm4_2 <- glmer(cbind(surv30,n - surv30) ~ Pupal_age +(1|rep), family =
binomial, data = data_4)
summary(fm4_1)
```

```
## Generalized linear mixed model fit by maximum likelihood (Laplace
##   Approximation) [glmerMod]
##  Family: binomial  ( logit )
## Formula: cbind(surv15, n - surv15) ~ Pupal_age + (1 | rep)
##    Data: data_4
##
##      AIC      BIC   logLik deviance df.resid
##    277.9    283.5   -135.9    271.9       45
##
## Scaled residuals:
##     Min      1Q  Median      3Q     Max
## -4.7004 -1.0567  0.3759  1.2843  2.4175
##
## Random effects:
##  Groups Name        Variance Std.Dev.
##  rep    (Intercept) 0.008696 0.09325
## Number of obs: 48, groups:  rep, 3
##
## Fixed effects:
##             Estimate Std. Error z value Pr(>|z|)
## (Intercept)  0.43507    0.68723   0.633    0.527
## Pupal_age    0.04132    0.02670   1.547    0.122
##
## Correlation of Fixed Effects:
##           (Intr)
## Pupal_age -0.988
```

```r
########################## survival: binomial model survival rate 60 days

###Treatments
fm4_3<- glmer(cbind(surv60,n - surv60) ~ Treatment +(1|rep), family =
binomial, data = data_4)
summary(fm4_3)
```

```
## Generalized linear mixed model fit by maximum likelihood (Laplace
##   Approximation) [glmerMod]
##  Family: binomial  ( logit )
## Formula: cbind(surv60, n - surv60) ~ Treatment + (1 | rep)
##    Data: data_4
##
##      AIC      BIC   logLik deviance df.resid
##    249.9    259.2   -119.9    239.9       43
##
## Scaled residuals:
##     Min      1Q  Median      3Q     Max
## -2.7316 -1.2366 -0.5862  1.4032  5.2381
##
## Random effects:
##  Groups Name        Variance Std.Dev.
##  rep    (Intercept) 0.006682 0.08175
## Number of obs: 48, groups:  rep, 3
##
## Fixed effects:
##                            Estimate Std. Error z value Pr(>|z|)
## (Intercept)                  0.4723     0.1478   3.196  0.00139 **
## Treatment2_Shipped-0Gy      -0.4966     0.2092  -2.374  0.01758 *
## Treatment3_Unshipped-110Gy  -3.0455     0.3110  -9.794  < 2e-16 ***
## Treatment4_Shipped-110Gy    -2.7773     0.2907  -9.555  < 2e-16 ***
## ---
## Signif. codes:  0 '***' 0.001 '**' 0.01 '*' 0.05 '.' 0.1 ' ' 1
##
## Correlation of Fixed Effects:
##             (Intr) T2_S-0 T3_U-1
## Trtmn2_S-0G -0.634
## Trt3_U-110G -0.427  0.302
## Trt4_S-110G -0.457  0.323  0.219
```

```r
###Pupal age

fm4_3<- glmer(cbind(surv60,n - surv60) ~ Pupal_age +(1|rep), family =
binomial, data = data_4)
```

```
## boundary (singular) fit: see help('isSingular')
```

```r
summary(fm4_3)
```

```
## Generalized linear mixed model fit by maximum likelihood (Laplace
##   Approximation) [glmerMod]
```

```
##  Family: binomial  ( logit )
## Formula: cbind(surv60, n - surv60) ~ Pupal_age + (1 | rep)
##    Data: data_4
## 
##      AIC      BIC   logLik deviance df.resid 
##    468.1    473.8   -231.1    462.1       45 
## 
## Scaled residuals: 
##    Min     1Q Median     3Q    Max 
## -3.368 -2.328 -0.763  2.161  5.494 
## 
## Random effects:
##  Groups Name        Variance Std.Dev.
##  rep    (Intercept) 4e-14    2e-07   
## Number of obs: 48, groups:  rep, 3
## 
## Fixed effects:
##              Estimate Std. Error z value Pr(>|z|)    
## (Intercept) -2.24331    0.58820  -3.814 0.000137 ***
## Pupal_age    0.05779    0.02243   2.577 0.009978 ** 
## ---
## Signif. codes:  0 '***' 0.001 '**' 0.01 '*' 0.05 '.' 0.1 ' ' 1
## 
## Correlation of Fixed Effects:
##           (Intr)
## Pupal_age -0.991
## optimizer (Nelder_Mead) convergence code: 0 (OK)
## boundary (singular) fit: see help('isSingular')
```

MODELS ON THE IMPACT OF SHOCK ON THE EMERGENCE RATE OF 22 AND 29 DAY OLD PUPAE (COMBINED DATA)

Figure 9a

```
tab <- read.csv("Figure 9a.csv")
head(tab)
```

```
##     Date Replicate Age     Treatments emerged unemerged shipped
Irradiation
## 1 170510        R1  22 Shipped-110Gy      125       100     yes
yes
## 2 170510        R1  22   Shipped-0Gy      126        99     yes
no
## 3 170517        R2  22 Shipped-110Gy       91       134     yes
yes
## 4 170517        R2  22   Shipped-0Gy       88       137     yes
no
## 5 170524        R3  22 Shipped-110Gy      173        64     yes
yes
## 6 170524        R3  22   Shipped-0Gy      183        54     yes
no
```

```
##   Events._5max Duration.ms._5max scalar_max_5max scalar_mean_5max
## 1          909            48.750          27.374         21.43900
## 2          909            48.750          27.374         21.43900
## 3          708            87.500          27.398         16.92723
## 4          708            87.500          27.398         16.92723
## 5         1187            93.125          27.374         15.43705
## 6         1187            93.125          27.374         15.43705
##   changescalar_max_5max changescalar_mean_5max Changevector_max_5max
## 1                23.724                8.94875              27.69156
## 2                23.724                8.94875              27.69156
## 3                24.477               11.37550              35.06987
## 4                24.477               11.37550              35.06987
## 5                16.627                3.83800              33.00443
## 6                16.627                3.83800              33.00443
##   Changevector_mean_5max Angle_max_5max Angle_mean_5max Hz_max_5max
## 1               9.937386       3.002116        2.617132    764.4826
## 2               9.937386       3.002116        2.617132    764.4826
## 3              19.035967       2.969360        1.679485    756.1414
## 4              19.035967       2.969360        1.679485    756.1414
## 5               5.064434       2.105922        1.356174    536.2686
## 6               5.064434       2.105922        1.356174    536.2686
##   Hz_mean_5max Events_10max Duration.ms._10max scalar_max_10max
## 1     666.4471          288             36.875           27.374
## 2     666.4471          288             36.875           27.374
## 3     427.6775          315             50.000           27.398
## 4     427.6775          315             50.000           27.398
## 5     345.3468          238             63.750           27.374
## 6     345.3468          238             63.750           27.374
##   scalar_mean_10max changescalar_max_10max changescalar_mean_10max
## 1           21.5800                 23.724                  12.473
## 2           21.5800                 23.724                  12.473
## 3           20.3750                 24.477                  16.488
## 4           20.3750                 24.477                  16.488
## 5           19.6895                 16.627                   8.965
## 6           19.6895                 16.627                   8.965
##   Changevector_max_10max Changevector_mean_10max Angle_max_10max
## 1               27.69156                15.45774        2.687392
## 2               27.69156                15.45774        2.687392
## 3               35.06987                22.58486        2.969360
## 4               35.06987                22.58486        2.969360
## 5               33.00443                10.65686        2.105922
## 6               33.00443                10.65686        2.105922
##   Angle_mean_10max Hz_max_10max Hz_mean_10max Events_15max
Duration.ms._15max
## 1        1.0470779     684.3388      266.6362          144
30.625
## 2        1.0470779     684.3388      266.6362          144
30.625
## 3        1.6138687     756.1414      410.9683          183
29.375
```

```
## 4       1.6138687     756.1414      410.9683          183
29.375
## 5       0.5851573     536.2686      149.0091          111
41.250
## 6       0.5851573     536.2686      149.0091          111
41.250
##   scalar_max_15max scalar_mean_15max changescalar_max_15max
## 1           27.374          22.42986                23.724
## 2           27.374          22.42986                23.724
## 3           27.398          21.71558                24.477
## 4           27.398          21.71558                24.477
## 5           27.374          22.77170                16.627
## 6           27.374          22.77170                16.627
##   changescalar_mean_15max Changevector_max_15max Changevector_mean_15max
## 1                  15.667               27.69156                19.75723
## 2                  15.667               27.69156                19.75723
## 3                  16.488               35.06987                21.61531
## 4                  16.488               35.06987                21.61531
## 5                  11.474               33.00443                12.83840
## 6                  11.474               33.00443                12.83840
##   Angle_max_15max Angle_mean_15max Hz_max_15max Hz_mean_15max Events_20max
## 1        2.687392        1.8427590     684.3388      469.2547           57
## 2        2.687392        1.8427590     684.3388      469.2547           57
## 3        2.969360        1.6010884     756.1414      407.7138           75
## 4        2.969360        1.6010884     756.1414      407.7138           75
## 5        2.105922        0.6709018     536.2686      170.8438           42
## 6        2.105922        0.6709018     536.2686      170.8438           42
##   Duration.ms._20max scalar_max_20max scalar_mean_20max
changescalar_max_20max
## 1             17.500           27.374          26.80175
23.724
## 2             17.500           27.374          26.80175
23.724
## 3             20.625           27.398          26.38567
24.477
## 4             20.625           27.398          26.38567
24.477
## 5             20.625           27.374          25.17900
13.480
## 6             20.625           27.374          25.17900
13.480
##   changescalar_mean_20max Changevector_max_20max Changevector_mean_20max
## 1                 23.7240               27.69156                27.69156
## 2                 23.7240               27.69156                27.69156
## 3                 13.7095               35.06987                29.65156
## 4                 13.7095               35.06987                29.65156
## 5                  6.6090               33.00443                11.19072
## 6                  6.6090               33.00443                11.19072
##   Angle_max_20max Angle_mean_20max Hz_max_20max Hz_mean_20max Events_5mean
## 1        2.687392         2.687392     684.3388      684.3388          909
```

```
## 2       2.687392         2.687392     684.3388      684.3388           909
## 3       2.433775         1.792941     619.7557      456.5688           708
## 4       2.433775         1.792941     619.7557      456.5688           708
## 5       1.715913         0.489164     436.9538      124.5646          1187
## 6       1.715913         0.489164     436.9538      124.5646          1187
##   Duration.ms._5mean scalar_max_5mean scalar_mean_5mean
changescalar_max_5mean
## 1           5.215209         9.516648          7.473747
3.755884
## 2           5.215209         9.516648          7.473747
3.755884
## 3          11.785840        10.551797          7.565272
2.816095
## 4          11.785840        10.551797          7.565272
2.816095
## 5           7.348884         8.022198          6.506753
1.632164
## 6           7.348884         8.022198          6.506753
1.632164
##   changescalar_mean_5mean Changevector_max_5mean Changevector_mean_5mean
## 1               1.6630629               4.501180                2.219564
## 2               1.6630629               4.501180                2.219564
## 3               1.0076953               3.691769                1.638802
## 4               1.0076953               3.691769                1.638802
## 5               0.6804169               2.293485                1.194539
## 6               0.6804169               2.293485                1.194539
##   Angle_max_5mean Angle_mean_5mean Hz_max_5mean Hz_mean_5mean
Events._mean10
## 1       0.4296757        0.1755708    109.41603      44.70874
288
## 2       0.4296757        0.1755708    109.41603      44.70874
288
## 3       0.3715939        0.1414327     94.62562      36.01553
315
## 4       0.3715939        0.1414327     94.62562      36.01553
315
## 5       0.2539263        0.1310499     64.66179      33.37158
238
## 6       0.2539263        0.1310499     64.66179      33.37158
238
##   Duration.ms._10mean scalar_max_10mean scalar_mean_10mean
## 1            3.888889          15.49537           13.49979
## 2            3.888889          15.49537           13.49979
## 3            7.706349          16.38244           13.65682
## 4            7.706349          16.38244           13.65682
## 5            7.415966          15.56483           13.15894
## 6            7.415966          15.56483           13.15894
##   changescalar_max_10mean changescalar_mean_10mean Changevector_max_10mean
## 1                6.557340                 3.249119                8.029862
## 2                6.557340                 3.249119                8.029862
```

```
## 3               4.928854                  1.971893                6.505797
## 4               4.928854                  1.971893                6.505797
## 5               4.004025                  1.664277                5.891075
## 6               4.004025                  1.664277                5.891075
##   Changevector_mean_10mean Angle_max_10mean Angle_mean_10mean
Hz_max_10mean
## 1                 4.538349        0.4719538         0.2224823
120.1821
## 2                 4.538349        0.4719538         0.2224823
120.1821
## 3                 3.339135        0.4047630         0.1751144
103.0721
## 4                 3.339135        0.4047630         0.1751144
103.0721
## 5                 2.909783        0.3492638         0.1583697
88.9393
## 6                 2.909783        0.3492638         0.1583697
88.9393
##   Hz_mean_10mean Events_15mean Duration.ms._15mean scalar_max_15mean
## 1       56.65466           144            3.441840          19.26462
## 2       56.65466           144            3.441840          19.26462
## 3       44.59251           183            6.342213          19.93954
## 4       44.59251           183            6.342213          19.93954
## 5       40.32851           111            6.920045          19.71768
## 6       40.32851           111            6.920045          19.71768
##   scalar_mean_15mean changescalar_max_15mean changescalar_mean_15mean
## 1           17.94158                8.195410                 4.209463
## 2           17.94158                8.195410                 4.209463
## 3           17.79343                6.271208                 2.424063
## 4           17.79343                6.271208                 2.424063
## 5           17.60073                5.291784                 2.055466
## 6           17.60073                5.291784                 2.055466
##   Changevector_max_15mean Changevector_mean_15mean Angle_max_15mean
## 1               10.145769                 6.194357        0.4732660
## 2               10.145769                 6.194357        0.4732660
## 3                8.773143                 4.485467        0.4584332
## 4                8.773143                 4.485467        0.4584332
## 5                8.644509                 4.178605        0.4437400
## 6                8.644509                 4.178605        0.4437400
##   Angle_mean_15mean Hz_max_15mean Hz_mean_15mean Events_20mean
## 1         0.2644405      120.5162       67.33922            57
## 2         0.2644405      120.5162       67.33922            57
## 3         0.2042285      116.7390       52.00635            75
## 4         0.2042285      116.7390       52.00635            75
## 5         0.1960600      112.9975       49.92626            42
## 6         0.1960600      112.9975       49.92626            42
##   Duration.ms._20mean scalar_max_20mean scalar_mean_20mean
## 1            2.587719          22.76440           22.04857
## 2            2.587719          22.76440           22.04857
## 3            5.250000          23.71752           22.29175
```

```
## 4           5.250000         23.71752          22.29175
## 5           5.997024         24.28693          22.58697
## 6           5.997024         24.28693          22.58697
##   changescalar_max_20mean changescalar_mean_20mean Changevector_max_20mean
## 1               7.502070                 3.960703                11.35123
## 2               7.502070                 3.960703                11.35123
## 3               6.190293                 2.676377                11.36833
## 4               6.190293                 2.676377                11.36833
## 5               5.996190                 2.445063                11.97291
## 6               5.996190                 2.445063                11.97291
##   Changevector_mean_20mean Angle_max_20mean Angle_mean_20mean
Hz_max_20mean
## 1                6.922532        0.5201012         0.3140733
132.4427
## 2                6.922532        0.5201012         0.3140733
132.4427
## 3                5.877764        0.5214004         0.2548023
132.7735
## 4                5.877764        0.5214004         0.2548023
132.7735
## 5                5.476046        0.5391966         0.2201107
137.3053
## 6                5.476046        0.5391966         0.2201107
137.3053
##   Hz_mean_20mean RH._Max Temp_Max RH._mean Temp_mean
## 1       79.97812   47.08    23.50    41.69     19.73
## 2       79.97812   60.14    27.50    41.69     19.73
## 3       64.88487   62.61    29.75    53.55     21.79
## 4       64.88487   68.10    32.00    53.55     21.79
## 5       56.05073   57.37    28.69    53.25     23.29
## 6       56.05073   74.59    19.25    53.25     23.29
```

```
str(tab)
```

```
## 'data.frame':    22 obs. of  108 variables:
##  $ Date                    : int  170510 170510 170517 170517 170524
170524 170531 170531 170607 170607 ...
##  $ Replicate               : chr  "R1" "R1" "R2" "R2" ...
##  $ Age                     : int  22 22 22 22 22 22 22 22 22 22 ...
##  $ Treatments              : chr  "Shipped-110Gy " "Shipped-0Gy "
"Shipped-110Gy " "Shipped-0Gy " ...
##  $ emerged                 : int  125 126 91 88 173 183 173 178 181 189
...
##  $ unemerged               : int  100 99 134 137 64 54 52 47 44 36 ...
##  $ shipped                 : chr  "yes" "yes" "yes" "yes" ...
##  $ Irradiation             : chr  "yes" "no" "yes" "no" ...
##  $ Events._5max            : int  909 909 708 708 1187 1187 625 625 606
606 ...
##  $ Duration.ms._5max       : num  48.8 48.8 87.5 87.5 93.1 ...
##  $ scalar_max_5max         : num  27.4 27.4 27.4 27.4 27.4 ...
```

```
##  $ scalar_mean_5max        : num  21.4 21.4 16.9 16.9 15.4 ...
##  $ changescalar_max_5max   : num  23.7 23.7 24.5 24.5 16.6 ...
##  $ changescalar_mean_5max  : num  8.95 8.95 11.38 11.38 3.84 ...
##  $ Changevector_max_5max   : num  27.7 27.7 35.1 35.1 33 ...
##  $ Changevector_mean_5max  : num  9.94 9.94 19.04 19.04 5.06 ...
##  $ Angle_max_5max          : num  3 3 2.97 2.97 2.11 ...
##  $ Angle_mean_5max         : num  2.62 2.62 1.68 1.68 1.36 ...
##  $ Hz_max_5max             : num  764 764 756 756 536 ...
##  $ Hz_mean_5max            : num  666 666 428 428 345 ...
##  $ Events_10max            : int  288 288 315 315 238 238 261 261 219 219
...
##  $ Duration.ms._10max      : num  36.9 36.9 50 50 63.8 ...
##  $ scalar_max_10max        : num  27.4 27.4 27.4 27.4 27.4 ...
##  $ scalar_mean_10max       : num  21.6 21.6 20.4 20.4 19.7 ...
##  $ changescalar_max_10max  : num  23.7 23.7 24.5 24.5 16.6 ...
##  $ changescalar_mean_10max : num  12.47 12.47 16.49 16.49 8.97 ...
##  $ Changevector_max_10max  : num  27.7 27.7 35.1 35.1 33 ...
##  $ Changevector_mean_10max : num  15.5 15.5 22.6 22.6 10.7 ...
##  $ Angle_max_10max         : num  2.69 2.69 2.97 2.97 2.11 ...
##  $ Angle_mean_10max        : num  1.047 1.047 1.614 1.614 0.585 ...
##  $ Hz_max_10max            : num  684 684 756 756 536 ...
##  $ Hz_mean_10max           : num  267 267 411 411 149 ...
##  $ Events_15max            : int  144 144 183 183 111 111 163 163 129 129
...
##  $ Duration.ms._15max      : num  30.6 30.6 29.4 29.4 41.2 ...
##  $ scalar_max_15max        : num  27.4 27.4 27.4 27.4 27.4 ...
##  $ scalar_mean_15max       : num  22.4 22.4 21.7 21.7 22.8 ...
##  $ changescalar_max_15max  : num  23.7 23.7 24.5 24.5 16.6 ...
##  $ changescalar_mean_15max : num  15.7 15.7 16.5 16.5 11.5 ...
##  $ Changevector_max_15max  : num  27.7 27.7 35.1 35.1 33 ...
##  $ Changevector_mean_15max : num  19.8 19.8 21.6 21.6 12.8 ...
##  $ Angle_max_15max         : num  2.69 2.69 2.97 2.97 2.11 ...
##  $ Angle_mean_15max        : num  1.843 1.843 1.601 1.601 0.671 ...
##  $ Hz_max_15max            : num  684 684 756 756 536 ...
##  $ Hz_mean_15max           : num  469 469 408 408 171 ...
##  $ Events_20max            : int  57 57 75 75 42 42 97 97 71 71 ...
##  $ Duration.ms._20max      : num  17.5 17.5 20.6 20.6 20.6 ...
##  $ scalar_max_20max        : num  27.4 27.4 27.4 27.4 27.4 ...
##  $ scalar_mean_20max       : num  26.8 26.8 26.4 26.4 25.2 ...
##  $ changescalar_max_20max  : num  23.7 23.7 24.5 24.5 13.5 ...
##  $ changescalar_mean_20max : num  23.72 23.72 13.71 13.71 6.61 ...
##  $ Changevector_max_20max  : num  27.7 27.7 35.1 35.1 33 ...
##  $ Changevector_mean_20max : num  27.7 27.7 29.7 29.7 11.2 ...
##  $ Angle_max_20max         : num  2.69 2.69 2.43 2.43 1.72 ...
##  $ Angle_mean_20max        : num  2.687 2.687 1.793 1.793 0.489 ...
##  $ Hz_max_20max            : num  684 684 620 620 437 ...
##  $ Hz_mean_20max           : num  684 684 457 457 125 ...
##  $ Events_5mean            : int  909 909 708 708 1187 1187 625 625 606
606 ...
##  $ Duration.ms._5mean      : num  5.22 5.22 11.79 11.79 7.35 ...
```

```
##  $ scalar_max_5mean        : num  9.52 9.52 10.55 10.55 8.02 ...
##  $ scalar_mean_5mean       : num  7.47 7.47 7.57 7.57 6.51 ...
##  $ changescalar_max_5mean  : num  3.76 3.76 2.82 2.82 1.63 ...
##  $ changescalar_mean_5mean : num  1.66 1.66 1.01 1.01 0.68 ...
##  $ Changevector_max_5mean  : num  4.5 4.5 3.69 3.69 2.29 ...
##  $ Changevector_mean_5mean : num  2.22 2.22 1.64 1.64 1.19 ...
##  $ Angle_max_5mean         : num  0.43 0.43 0.372 0.372 0.254 ...
##  $ Angle_mean_5mean        : num  0.176 0.176 0.141 0.141 0.131 ...
##  $ Hz_max_5mean            : num  109.4 109.4 94.6 94.6 64.7 ...
##  $ Hz_mean_5mean           : num  44.7 44.7 36 36 33.4 ...
##  $ Events._mean10          : int  288 288 315 315 238 238 261 261 219 219
...
##  $ Duration.ms._10mean     : num  3.89 3.89 7.71 7.71 7.42 ...
##  $ scalar_max_10mean       : num  15.5 15.5 16.4 16.4 15.6 ...
##  $ scalar_mean_10mean      : num  13.5 13.5 13.7 13.7 13.2 ...
##  $ changescalar_max_10mean : num  6.56 6.56 4.93 4.93 4 ...
##  $ changescalar_mean_10mean: num  3.25 3.25 1.97 1.97 1.66 ...
##  $ Changevector_max_10mean : num  8.03 8.03 6.51 6.51 5.89 ...
##  $ Changevector_mean_10mean: num  4.54 4.54 3.34 3.34 2.91 ...
##  $ Angle_max_10mean        : num  0.472 0.472 0.405 0.405 0.349 ...
##  $ Angle_mean_10mean       : num  0.222 0.222 0.175 0.175 0.158 ...
##  $ Hz_max_10mean           : num  120.2 120.2 103.1 103.1 88.9 ...
##  $ Hz_mean_10mean          : num  56.7 56.7 44.6 44.6 40.3 ...
##  $ Events_15mean           : int  144 144 183 183 111 111 163 163 129 129
...
##  $ Duration.ms._15mean     : num  3.44 3.44 6.34 6.34 6.92 ...
##  $ scalar_max_15mean       : num  19.3 19.3 19.9 19.9 19.7 ...
##  $ scalar_mean_15mean      : num  17.9 17.9 17.8 17.8 17.6 ...
##  $ changescalar_max_15mean : num  8.2 8.2 6.27 6.27 5.29 ...
##  $ changescalar_mean_15mean: num  4.21 4.21 2.42 2.42 2.06 ...
##  $ Changevector_max_15mean : num  10.15 10.15 8.77 8.77 8.64 ...
##  $ Changevector_mean_15mean: num  6.19 6.19 4.49 4.49 4.18 ...
##  $ Angle_max_15mean        : num  0.473 0.473 0.458 0.458 0.444 ...
##  $ Angle_mean_15mean       : num  0.264 0.264 0.204 0.204 0.196 ...
##  $ Hz_max_15mean           : num  121 121 117 117 113 ...
##  $ Hz_mean_15mean          : num  67.3 67.3 52 52 49.9 ...
##  $ Events_20mean           : int  57 57 75 75 42 42 97 97 71 71 ...
##  $ Duration.ms._20mean     : num  2.59 2.59 5.25 5.25 6 ...
##  $ scalar_max_20mean       : num  22.8 22.8 23.7 23.7 24.3 ...
##  $ scalar_mean_20mean      : num  22 22 22.3 22.3 22.6 ...
##  $ changescalar_max_20mean : num  7.5 7.5 6.19 6.19 6 ...
##  $ changescalar_mean_20mean: num  3.96 3.96 2.68 2.68 2.45 ...
##  $ Changevector_max_20mean : num  11.4 11.4 11.4 11.4 12 ...
##   [list output truncated]

tab$pcemerg <- tab$emerged / (tab$emerged+tab$unemerged)

boxplot(tab$pcemerg ~ tab$Treatments, xlab ="Treatments", ylab = "Emergence
rate")
```

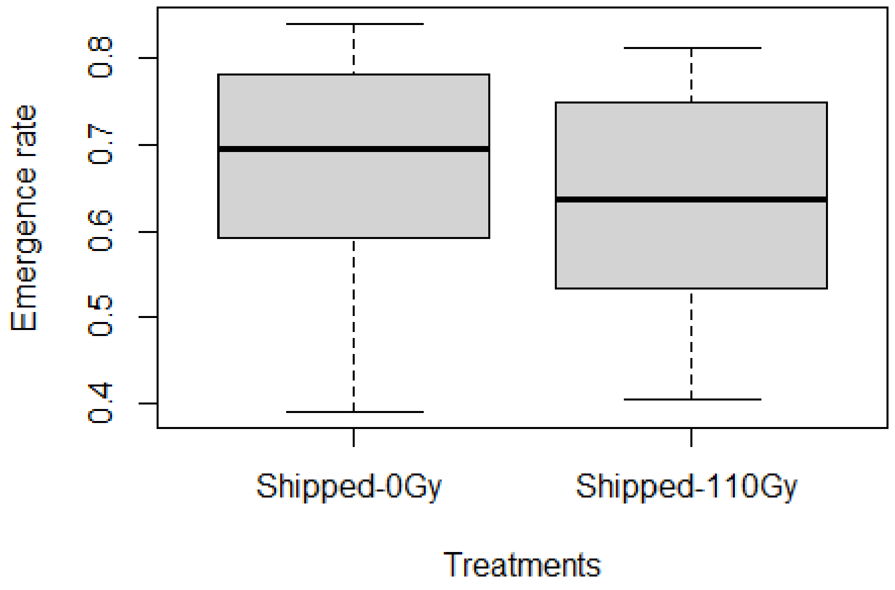


```
plot(tab$pcemerg~tab$RH._Max)#*
```

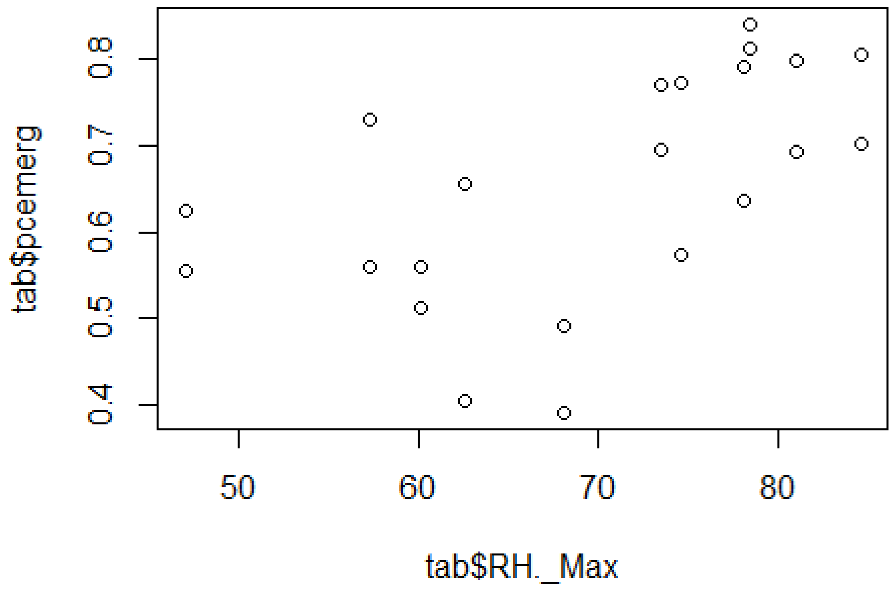


```
plot(tab$pcemerg~tab$RH._mean)
```

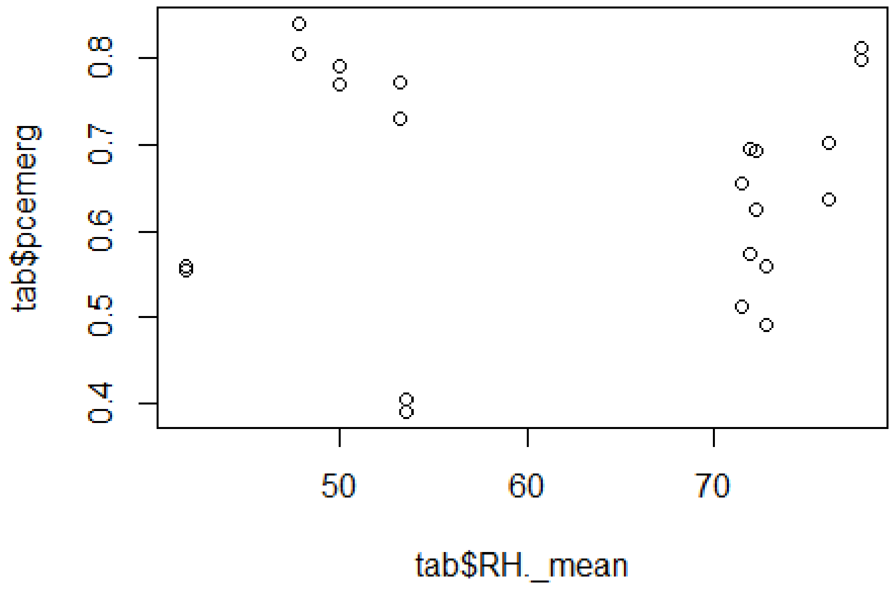


```
plot(tab$pcemerg~tab$Temp_mean)#*
```

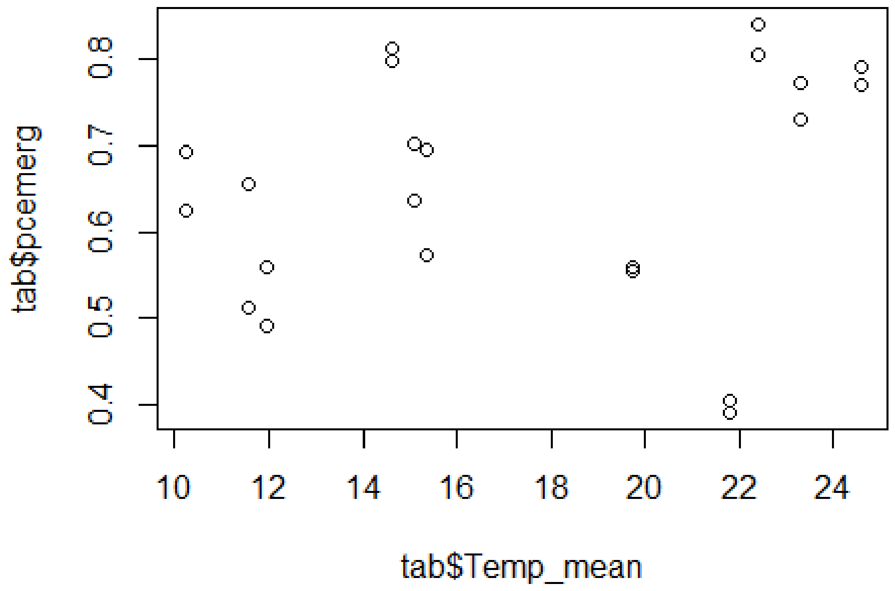


```
plot(tab$pcemerg~tab$Temp_Max)
```

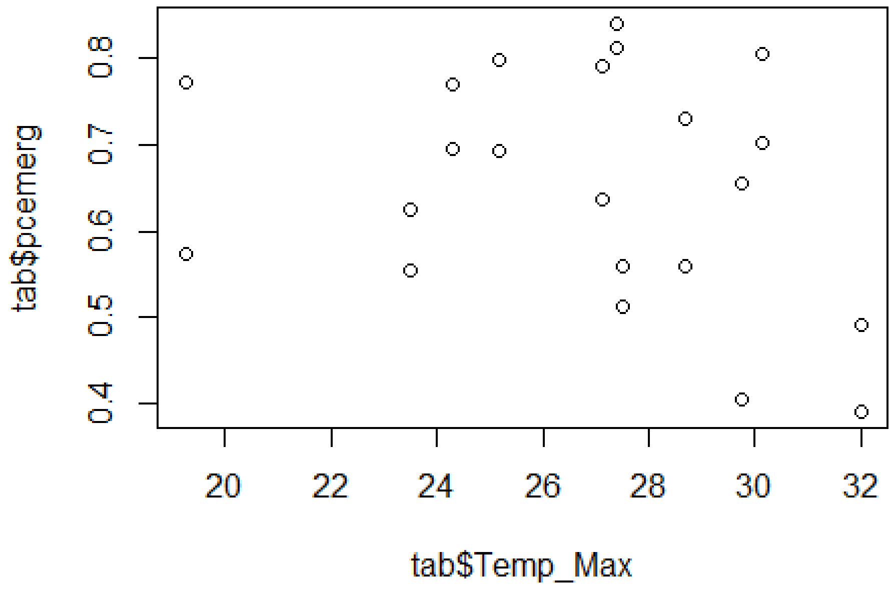


```
#Scatter plots at threshold 5-Max
par(mfrow = c(2,2))
plot(tab$pcemerg~tab$Events._5max)
plot(tab$pcemerg~tab$Duration.ms._5max)
plot(tab$pcemerg~tab$scalar_max_5max)
plot(tab$pcemerg~tab$scalar_mean_5max)
```

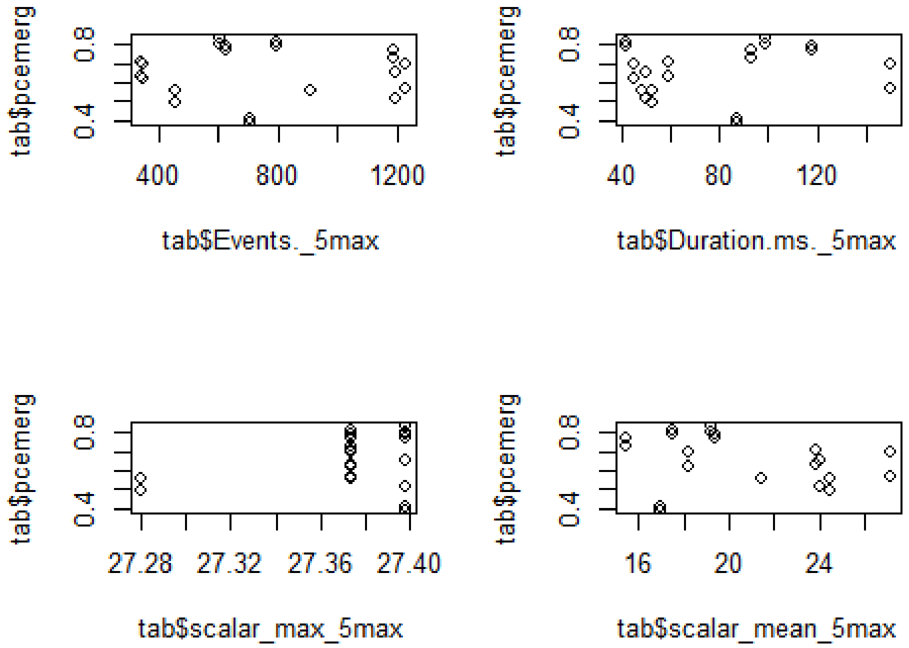


```
plot(tab$pcemerg~tab$changescalar_max_5max)
plot(tab$pcemerg~tab$changescalar_mean_5max)#*
plot(tab$pcemerg~tab$Changevector_max_5max)
plot(tab$pcemerg~tab$Changevector_mean_5max)
```

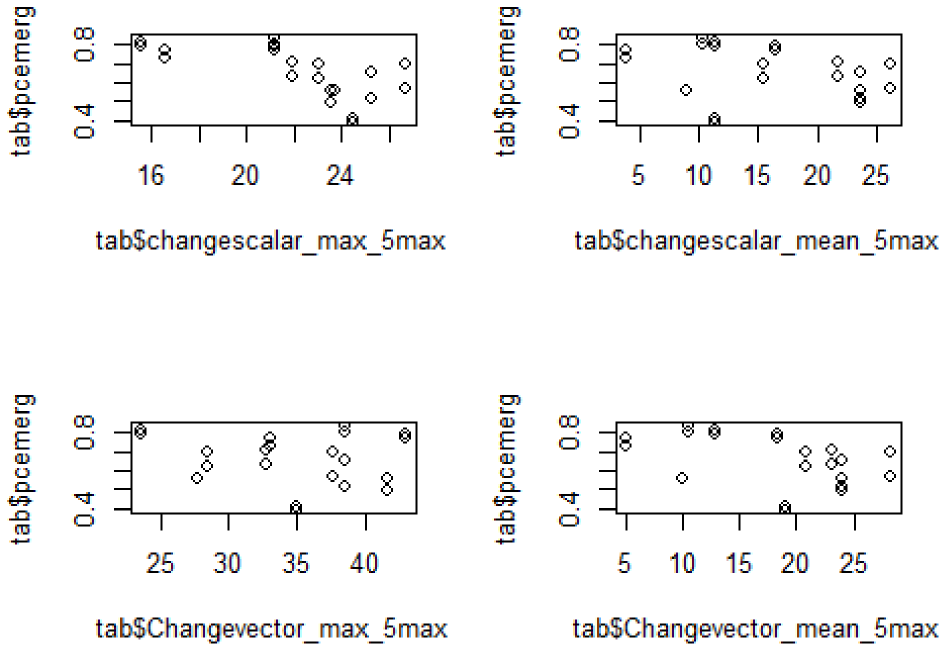


```
plot(tab$pcemerg~tab$Angle_max_5max)
plot(tab$pcemerg~tab$Angle_mean_5max)
plot(tab$pcemerg~tab$Hz_max_5max)
plot(tab$pcemerg~tab$Hz_mean_5max)
```

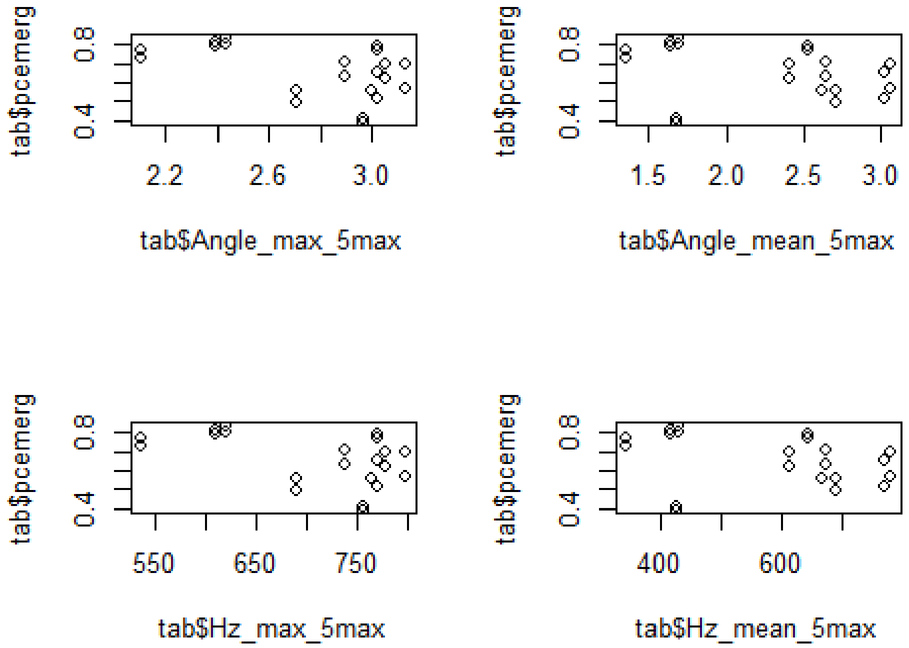

#Scatter plots at threshold 5-Mean

```
par(mfrow = c(2,2))
plot(tab$pcemerg~tab$Duration.ms._5mean)#*
plot(tab$pcemerg~tab$scalar_max_5mean)#*
plot(tab$pcemerg~tab$scalar_mean_5mean)
plot(tab$pcemerg~tab$changescalar_max_5mean)
```

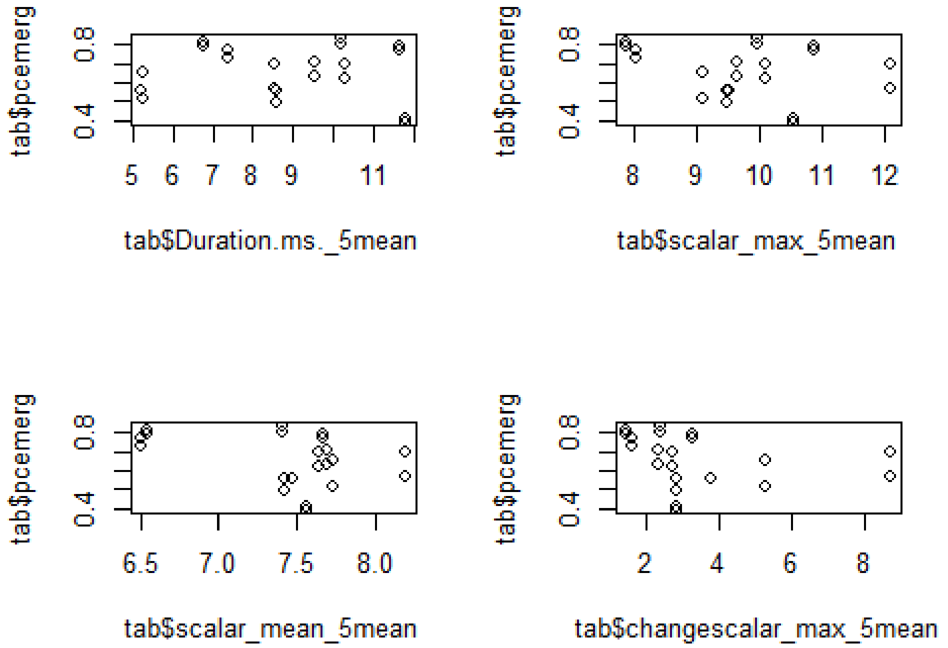


```
plot(tab$pcemerg~tab$changescalar_mean_5mean)
plot(tab$pcemerg~tab$Changevector_max_5mean)
plot(tab$pcemerg~tab$Changevector_mean_5mean)
plot(tab$pcemerg~tab$Angle_max_5mean)
```

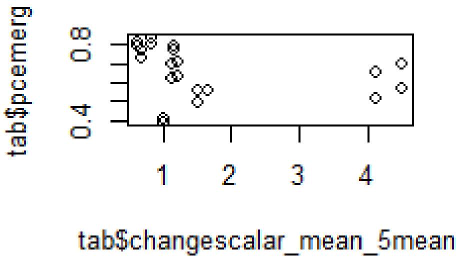

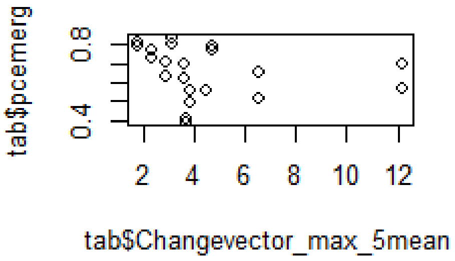

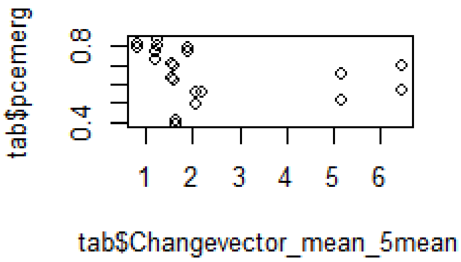

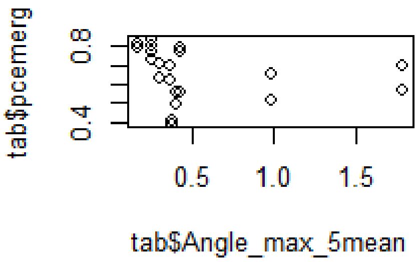


```
plot(tab$pcemerg~tab$Angle_mean_5mean)
plot(tab$pcemerg~tab$Hz_max_5mean)
plot(tab$pcemerg~tab$Hz_mean_5mean)
```

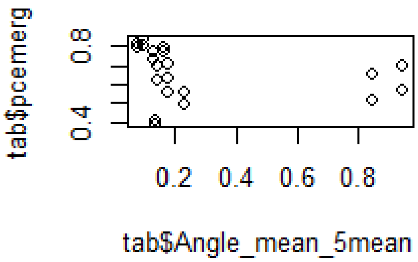

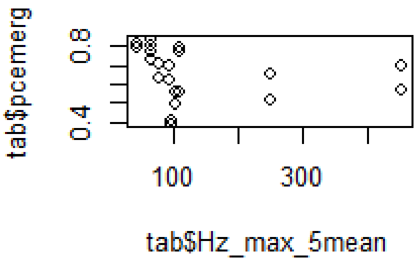

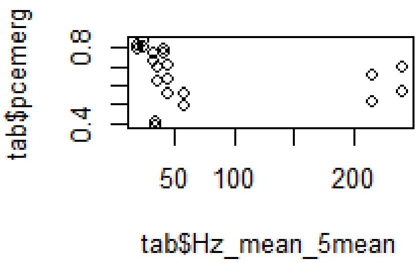

#Scatter plots at threshold 10-Max

```
par(mfrow = c(2,2))
plot(tab$pcemerg~tab$Events._mean10)
plot(tab$pcemerg~tab$Duration.ms._10max)#*
plot(tab$pcemerg~tab$scalar_max_10max)
plot(tab$pcemerg~tab$scalar_mean_10max)
```

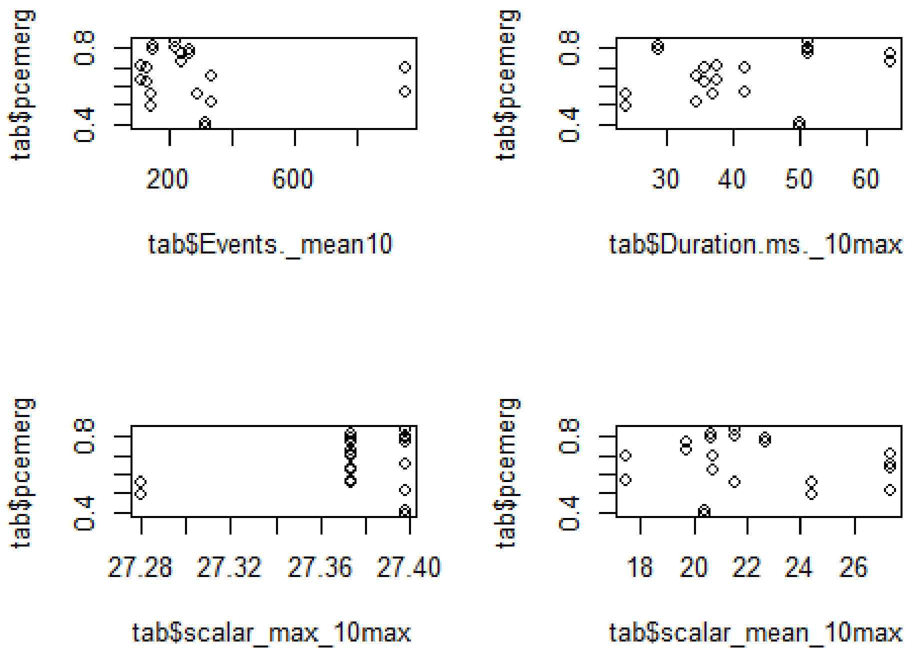


```
plot(tab$pcemerg~tab$changescalar_max_10max)
plot(tab$pcemerg~tab$changescalar_mean_10max)#*
plot(tab$pcemerg~tab$Changevector_max_10max)
plot(tab$pcemerg~tab$Changevector_mean_10max)
```

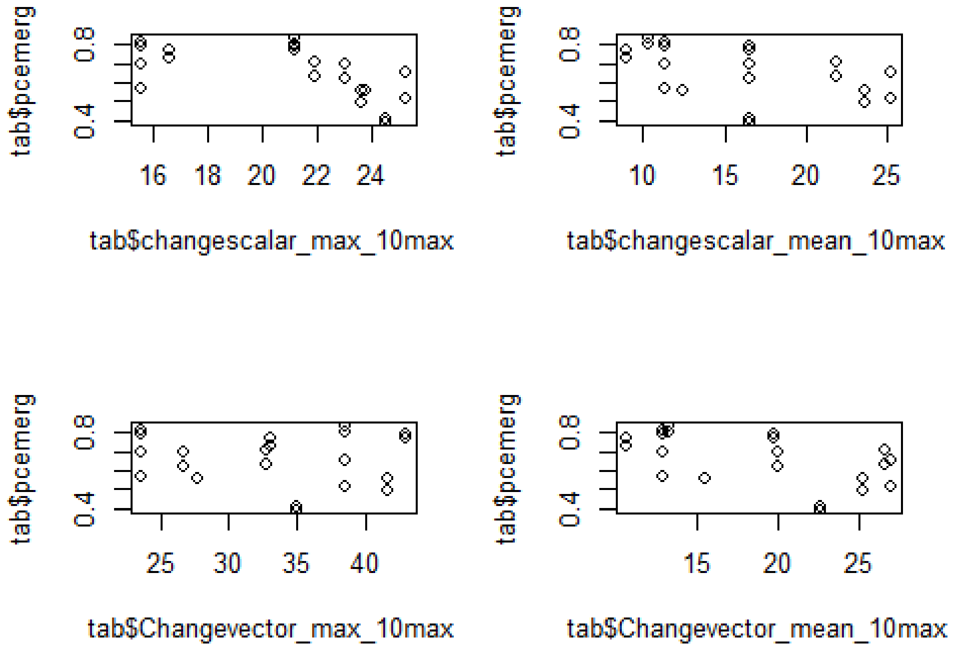


```
plot(tab$pcemerg~tab$Angle_max_10max)
plot(tab$pcemerg~tab$Angle_mean_10max)#*
plot(tab$pcemerg~tab$Hz_max_10max)
plot(tab$pcemerg~tab$Hz_mean_10max)#*
```

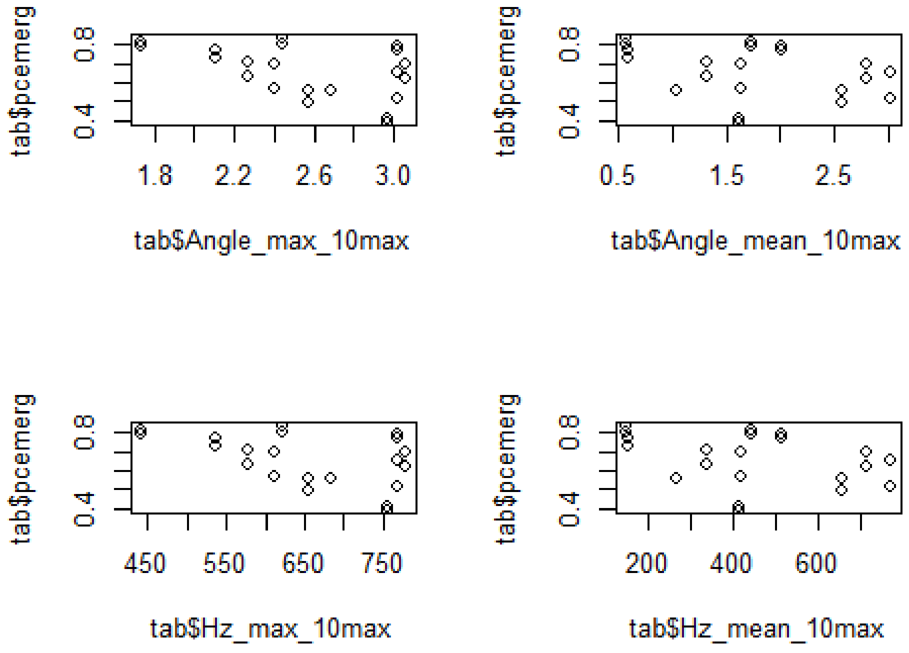

#Scatter plots at threshold 10-Mean

```
par(mfrow = c(2,2))
plot(tab$pcemerg~tab$Duration.ms._10mean)
plot(tab$pcemerg~tab$scalar_max_10mean)
plot(tab$pcemerg~tab$scalar_mean_10mean)#*
plot(tab$pcemerg~tab$changescalar_max_10mean)
```

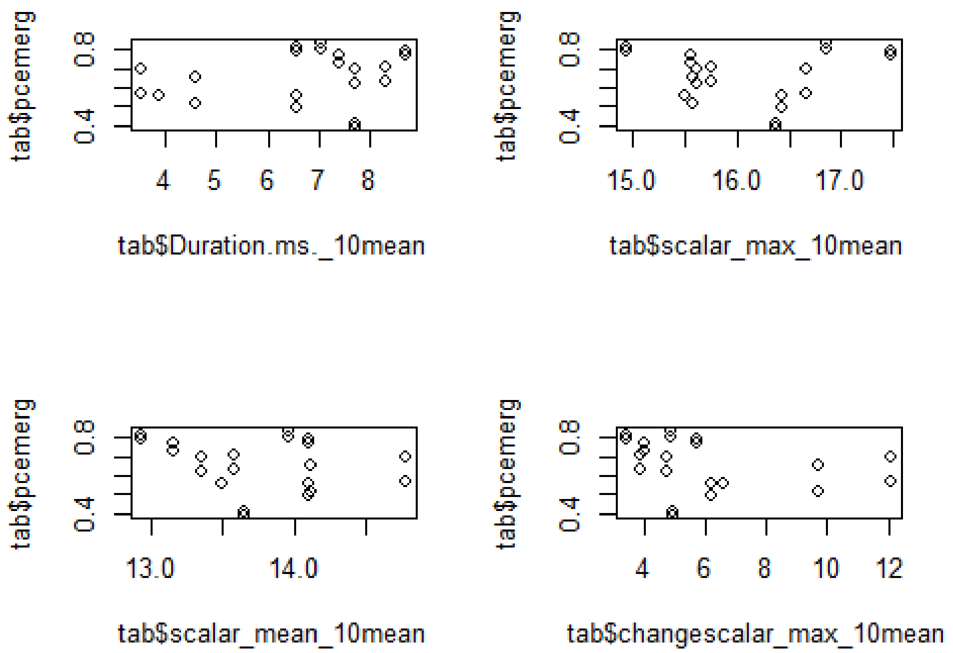


```
plot(tab$pcemerg~tab$changescalar_mean_10mean)
plot(tab$pcemerg~tab$Changevector_max_10mean)
plot(tab$pcemerg~tab$Changevector_mean_10mean)
plot(tab$pcemerg~tab$Angle_max_10mean)
```

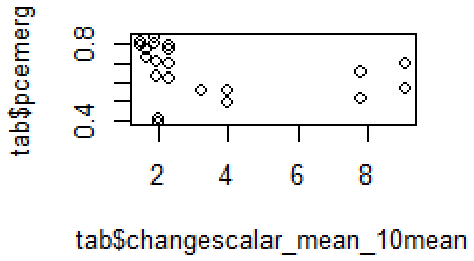

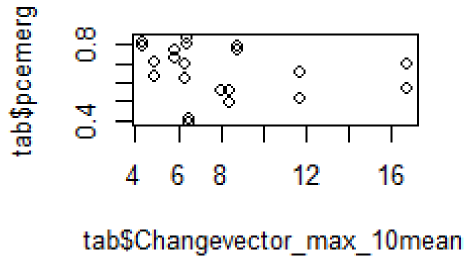

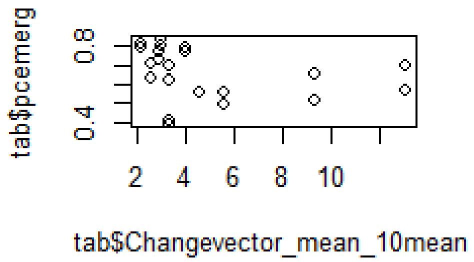

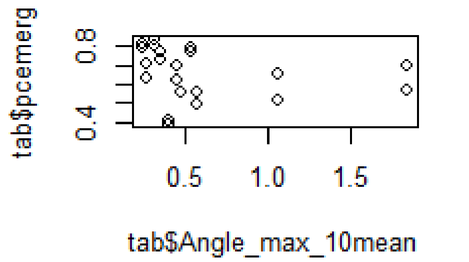


```
plot(tab$pcemerg~tab$Angle_mean_10mean)
plot(tab$pcemerg~tab$Hz_max_10mean)
plot(tab$pcemerg~tab$Hz_mean_10mean)
```

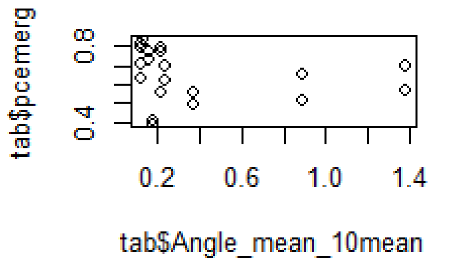

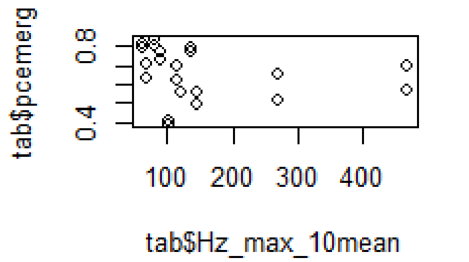

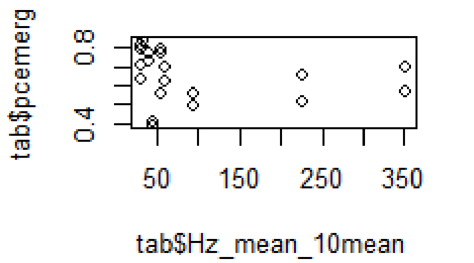

#Scatter plots at threshold 15-Max

```
par(mfrow = c(2,2))
plot(tab$pcemerg~tab$Events_15mean)
plot(tab$pcemerg~tab$Duration.ms._15max)
plot(tab$pcemerg~tab$scalar_max_15max)
plot(tab$pcemerg~tab$scalar_mean_15max)
```

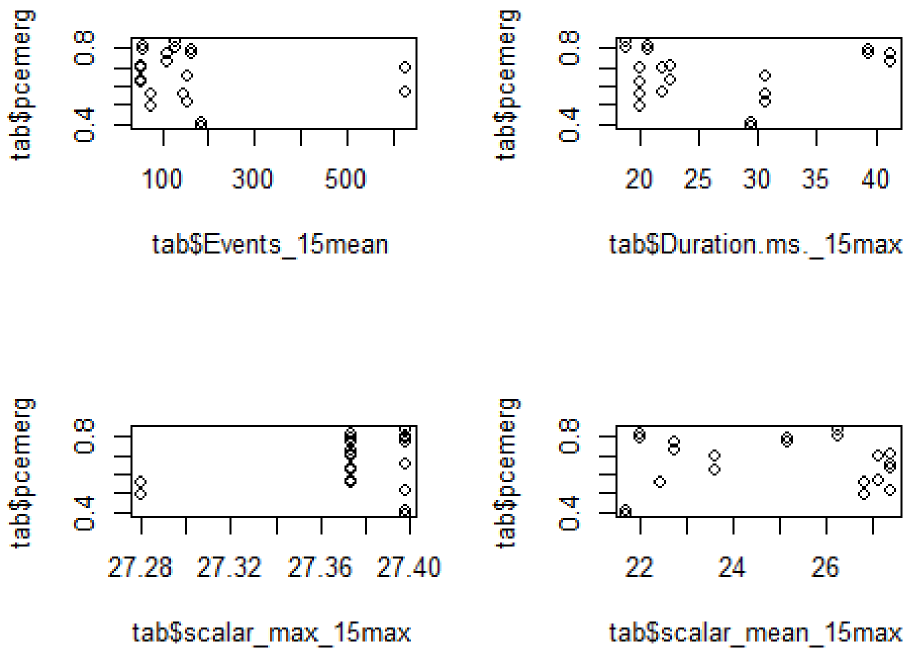


```
plot(tab$pcemerg~tab$changescalar_max_15max)
plot(tab$pcemerg~tab$changescalar_mean_15max)#*
cor.test(tab$pcemerg,tab$changescalar_mean_15max)

##
##  Pearson's product-moment correlation
##
## data:  tab$pcemerg and tab$changescalar_mean_15max
## t = -2.4767, df = 20, p-value = 0.02231
## alternative hypothesis: true correlation is not equal to 0
## 95 percent confidence interval:
##  -0.75240243 -0.07901092
## sample estimates:
##        cor
## -0.4844805

plot(tab$pcemerg~tab$Changevector_max_15max)
plot(tab$pcemerg~tab$Changevector_mean_15max)#
```

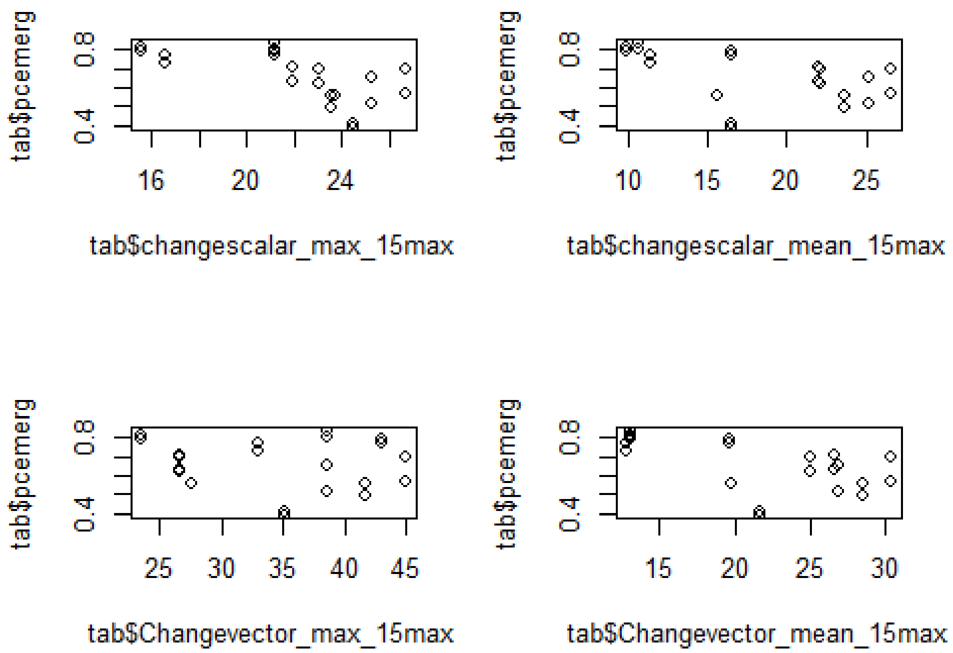


```
cor.test(tab$pcemerg,tab$Changevector_mean_15max)

##
##  Pearson's product-moment correlation
##
## data:  tab$pcemerg and tab$Changevector_mean_15max
## t = -3.0538, df = 20, p-value = 0.006267
## alternative hypothesis: true correlation is not equal to 0
## 95 percent confidence interval:
##  -0.7962240 -0.1867014
## sample estimates:
##        cor
## -0.5639208

plot(tab$pcemerg~tab$Angle_max_15max)
plot(tab$pcemerg~tab$Angle_mean_15max)#*
plot(tab$pcemerg~tab$Hz_max_15max)
plot(tab$pcemerg~tab$Hz_mean_15max)#*
```

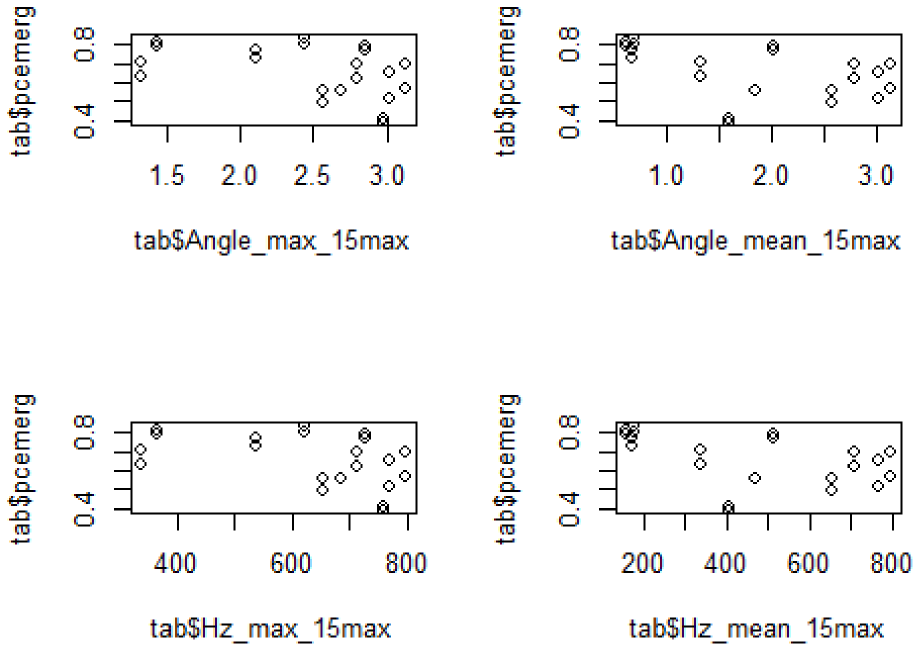

#Scatter plots at threshold 15-Mean

```
par(mfrow = c(2,2))
plot(tab$pcemerg~tab$Duration.ms._15mean)
plot(tab$pcemerg~tab$scalar_max_15mean)
plot(tab$pcemerg~tab$scalar_mean_15mean)#*
plot(tab$pcemerg~tab$changescalar_max_15mean)
```

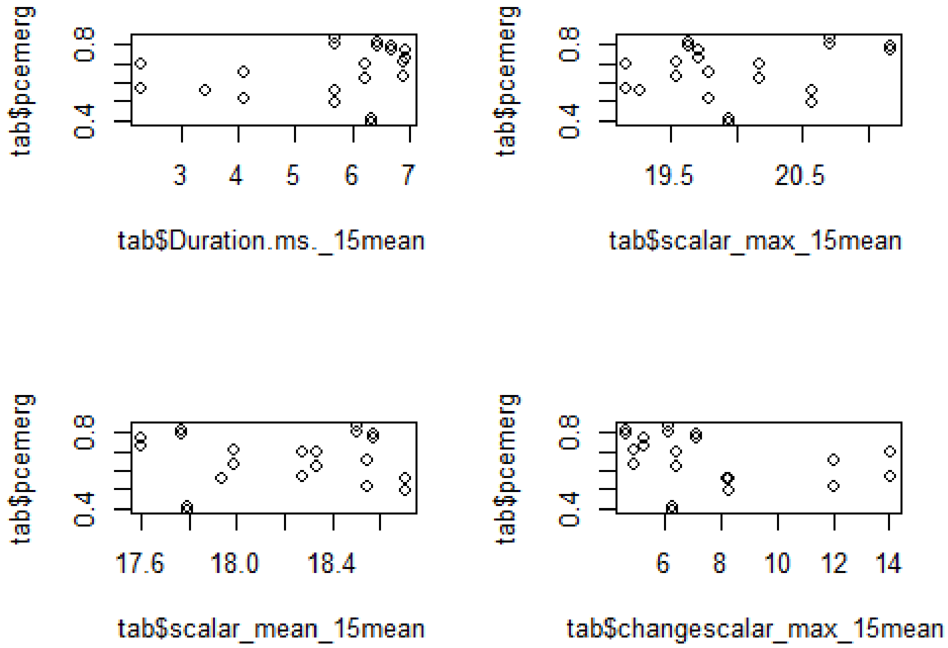


```
plot(tab$pcemerg~tab$changescalar_mean_15mean)#*
plot(tab$pcemerg~tab$Changevector_max_15mean) #*
plot(tab$pcemerg~tab$Changevector_mean_15mean)
plot(tab$pcemerg~tab$Angle_max_15mean)
```

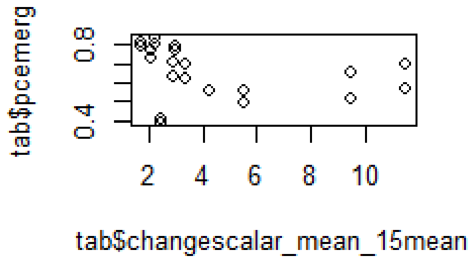

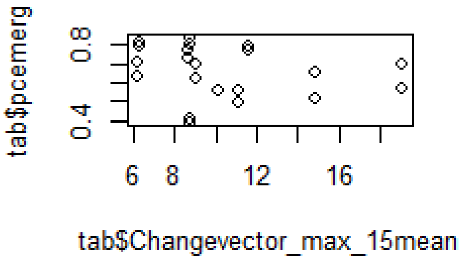

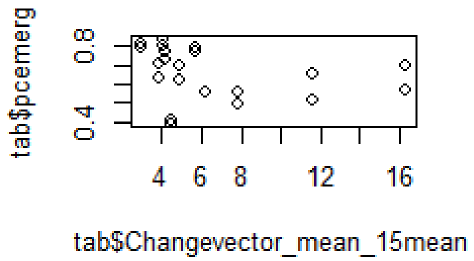

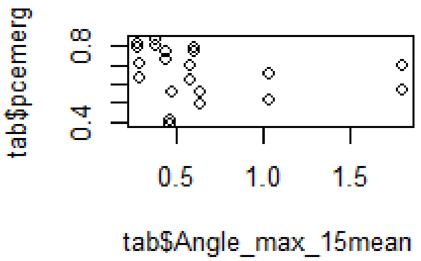


```
plot(tab$pcemerg~tab$Angle_mean_15mean)
plot(tab$pcemerg~tab$Hz_max_15mean)
plot(tab$pcemerg~tab$Hz_mean_15mean)
```

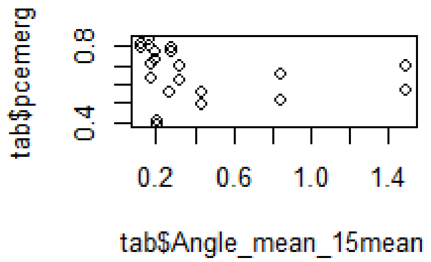

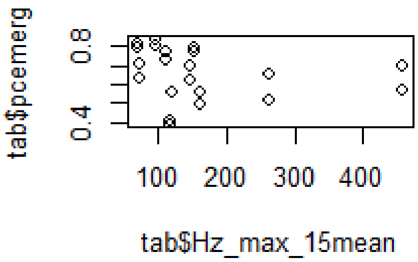

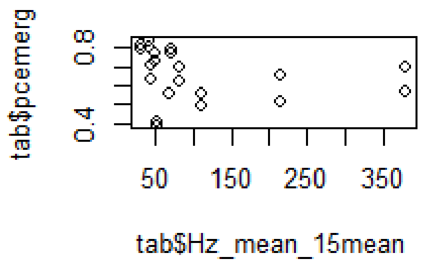

#Scatter plots at threshold 20-Max

```
par(mfrow = c(2,2))
plot(tab$pcemerg~tab$Events_20max)
plot(tab$pcemerg~tab$Duration.ms._20max)
plot(tab$pcemerg~tab$scalar_max_20max)
plot(tab$pcemerg~tab$scalar_mean_20max)#*
```

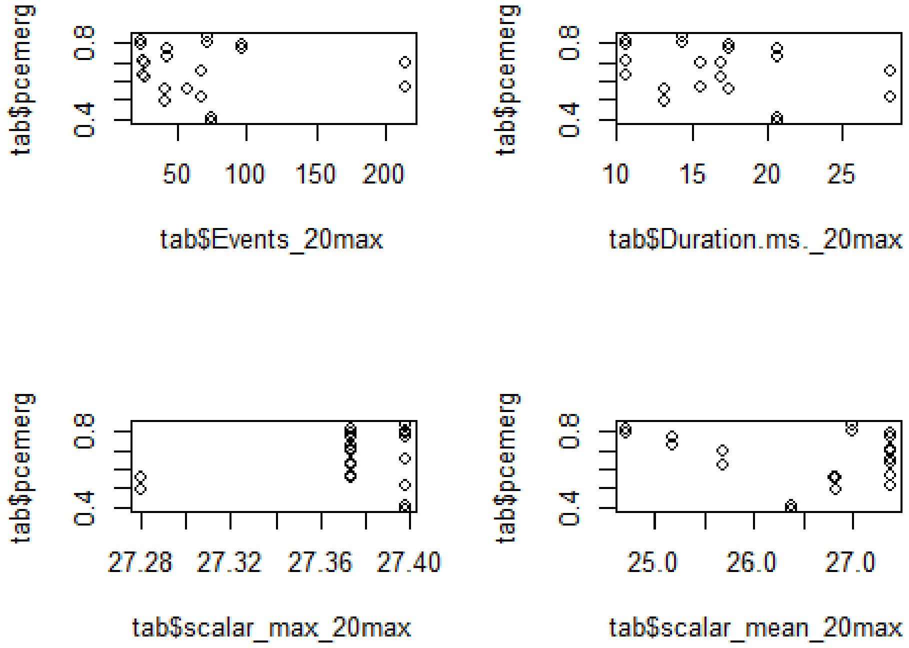


```
plot(tab$pcemerg~tab$changescalar_max_20max)
plot(tab$pcemerg~tab$changescalar_mean_20max)
plot(tab$pcemerg~tab$Changevector_max_20max)
plot(tab$pcemerg~tab$Changevector_mean_20max)
```

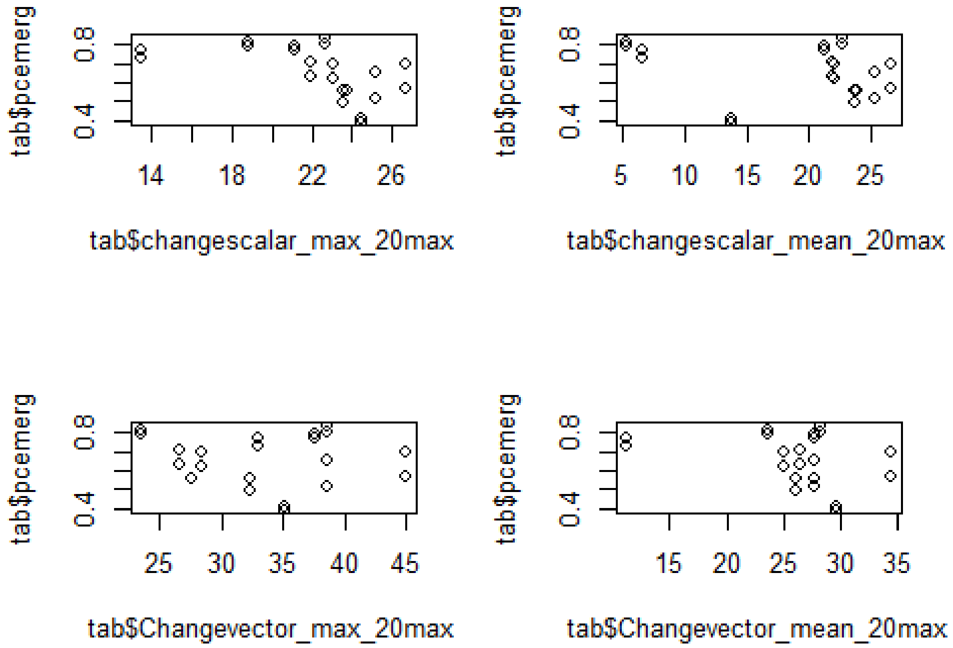


```
plot(tab$pcemerg~tab$Angle_max_20max)
plot(tab$pcemerg~tab$Angle_mean_20max)
plot(tab$pcemerg~tab$Hz_max_20max)
plot(tab$pcemerg~tab$Hz_mean_20max)
```

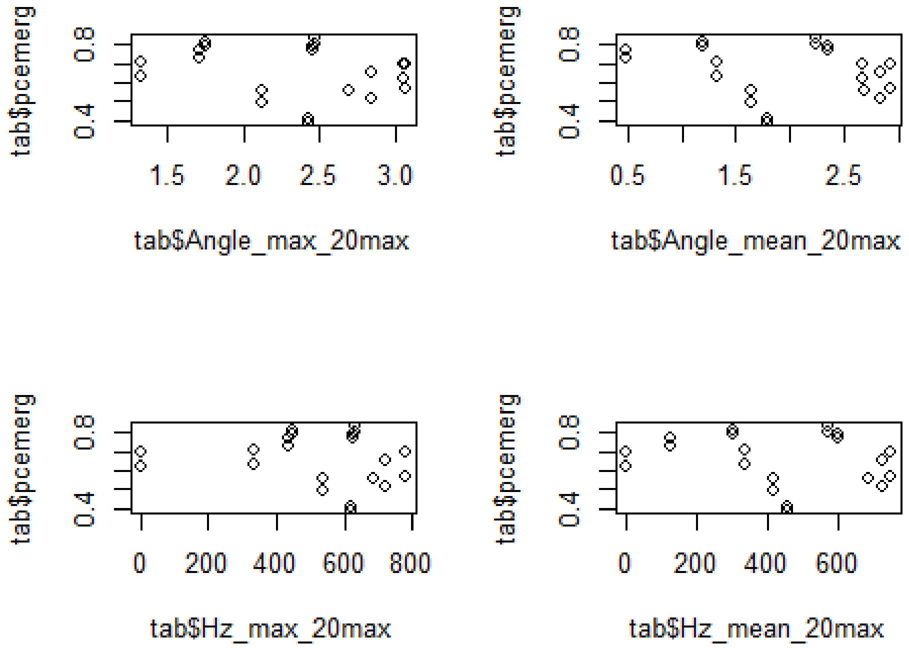

#Scatter plots at threshold 20-Mean

```
par(mfrow = c(2,2))
plot(tab$pcemerg~tab$Duration.ms._20mean)
plot(tab$pcemerg~tab$scalar_max_20mean)
plot(tab$pcemerg~tab$scalar_mean_20mean)
plot(tab$pcemerg~tab$changescalar_max_20mean)
```

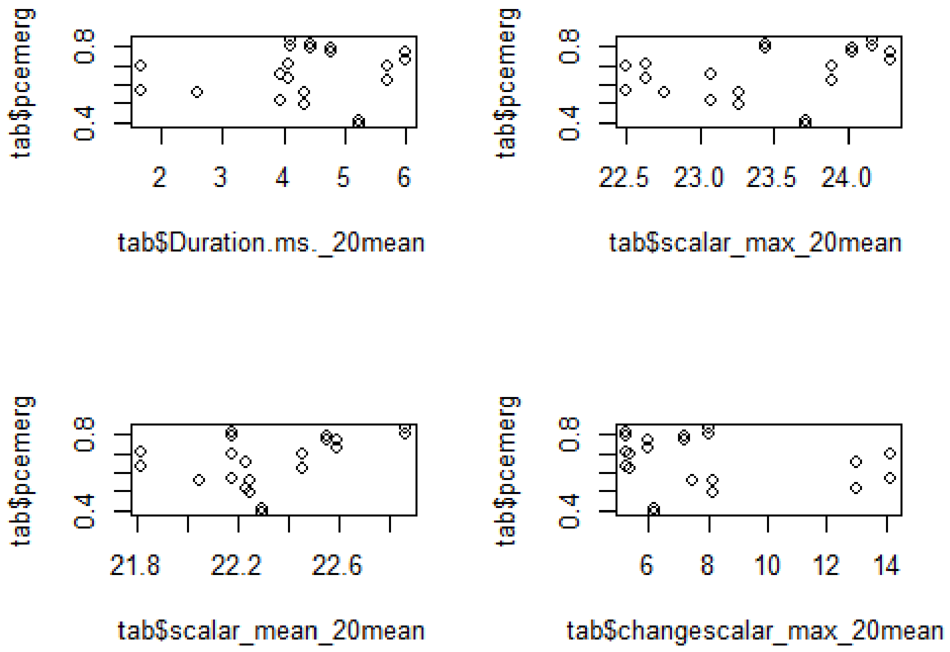


```
plot(tab$pcemerg~tab$changescalar_mean_20mean)#*
plot(tab$pcemerg~tab$Changevector_max_20mean)
plot(tab$pcemerg~tab$Changevector_mean_20mean)
plot(tab$pcemerg~tab$Angle_max_20mean)
```

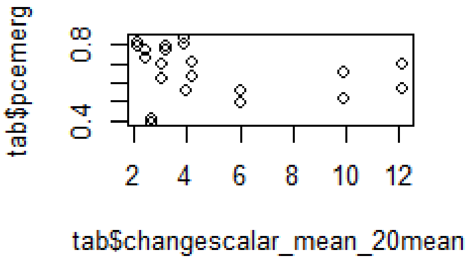

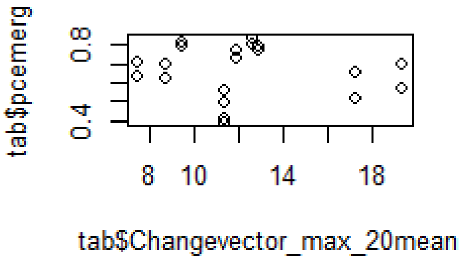

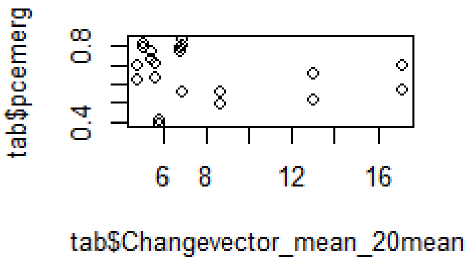

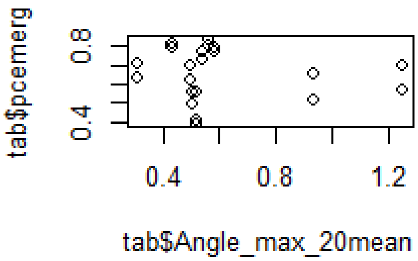


```
plot(tab$pcemerg~tab$Angle_mean_20mean)
plot(tab$pcemerg~tab$Hz_max_20mean)
plot(tab$pcemerg~tab$Hz_mean_20mean)
```

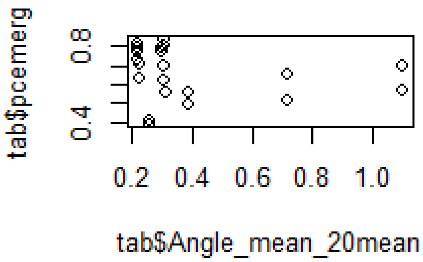

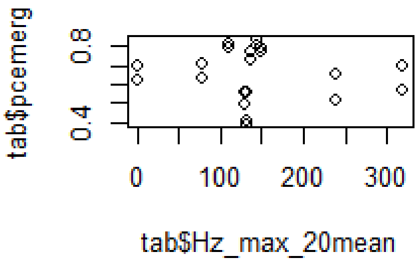

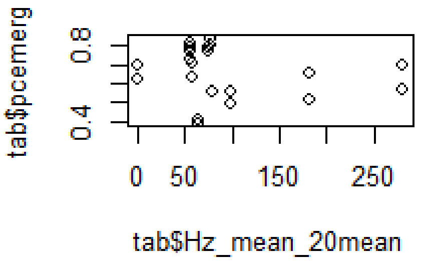

##Models of impact of shock on emergence rate of transported pupae

```
#models threshold 5
fm1 <- glmer(cbind(emerged,unemerged) ~ Irradiation + RH._Max + Temp_mean +
(1|Replicate), family = binomial, data = tab)
fm2 <- glmer(cbind(emerged,unemerged) ~ Irradiation + RH._Max + Temp_mean +
changescalar_mean_5max + scalar_max_5mean + (1|Replicate), family = binomial,
data = tab)
fm3 <- glmer(cbind(emerged,unemerged) ~ Irradiation + RH._Max + Temp_mean +
changescalar_mean_5max + Duration.ms._5mean +(1|Replicate), family =
binomial, data = tab)
fm4 <- glmer(cbind(emerged,unemerged) ~ Irradiation + RH._Max + Temp_mean +
changescalar_mean_5max + (1|Replicate), family = binomial, data = tab)
fm5 <- glmer(cbind(emerged,unemerged) ~ Irradiation + RH._Max + Temp_mean +
scalar_max_5mean + (1|Replicate), family = binomial, data = tab)
fm6 <- glmer(cbind(emerged,unemerged) ~ Irradiation + Temp_mean +
changescalar_mean_5max + scalar_max_5mean + (1|Replicate), family = binomial,
data = tab)
fm7 <- glmer(cbind(emerged,unemerged) ~ Irradiation + RH._Max + Temp_mean +
changescalar_mean_5max + scalar_max_5mean + (1|Replicate), family = binomial,
data = tab)
fm9 <- glmer(cbind(emerged,unemerged) ~ Irradiation + Temp_mean +
changescalar_mean_5max +  (1|Replicate), family = binomial, data = tab)
fm10 <- glmer(cbind(emerged,unemerged) ~ Irradiation + RH._Max + Temp_mean +
changescalar_mean_5max + Duration.ms._5mean +(1|Replicate), family =
binomial, data = tab)
fm11 <- glmer(cbind(emerged,unemerged) ~ Irradiation + RH._Max + Temp_mean +
(1|Replicate), family = binomial, data = tab)
fm12 <- glmer(cbind(emerged,unemerged) ~ Irradiation +Temp_mean + Age
+(1|Replicate), family = binomial, data = tab)
fm13 <- glmer(cbind(emerged,unemerged) ~ Irradiation +RH._Max + Age
+(1|Replicate), family = binomial, data = tab)

AICc(fm1,fm2,fm3,fm4,fm5,fm6,fm7,fm9,fm10,fm11,fm12,fm13)

##      df     AICc
## fm1   5 279.1278
## fm2   7 210.0473
## fm3   7 242.4495
## fm4   6 250.4973
## fm5   6 222.9305
## fm6   6 207.1041
## fm7   7 210.0473
## fm9   5 247.0403
## fm10  7 242.4495
## fm11  5 279.1278
## fm12  5 283.2114
## fm13  5 279.0186

summary(fm6)
```

```
## Generalized linear mixed model fit by maximum likelihood (Laplace
##   Approximation) [glmerMod]
##  Family: binomial  ( logit )
## Formula:
## cbind(emerged, unemerged) ~ Irradiation + Temp_mean +
changescalar_mean_5max +
##     scalar_max_5mean + (1 | Replicate)
##    Data: tab
##
##      AIC      BIC   logLik deviance df.resid
##    201.5    208.1    -94.8    189.5       16
##
## Scaled residuals:
##     Min      1Q  Median      3Q     Max
## -2.4604 -0.7569  0.1291  0.8611  2.0722
##
## Random effects:
##  Groups    Name        Variance Std.Dev.
##  Replicate (Intercept) 0.9111   0.9545
## Number of obs: 22, groups:  Replicate, 6
##
## Fixed effects:
##                         Estimate Std. Error z value Pr(>|z|)
## (Intercept)              7.99460    0.98899   8.084 6.29e-16 ***
## Irradiationyes          -0.18515    0.06827  -2.712  0.00669 **
## Temp_mean               -0.10359    0.02511  -4.126 3.69e-05 ***
## changescalar_mean_5max -0.08900    0.01937  -4.594 4.35e-06 ***
## scalar_max_5mean        -0.41860    0.06381  -6.561 5.36e-11 ***
## ---
## Signif. codes:  0 '***' 0.001 '**' 0.01 '*' 0.05 '.' 0.1 ' ' 1
##
## Correlation of Fixed Effects:
##             (Intr) Irrdtn Tmp_mn chn__5
## Irradiatnys -0.029
## Temp_mean   -0.694 -0.027
## chngsclr__5 -0.634 -0.019  0.954
## sclr_mx_5mn -0.562  0.020 -0.045 -0.132
```

```
fm11a <- glmer(cbind(emerged,unemerged) ~ Irradiation + Temp_mean +
Duration.ms._10max + changescalar_mean_10max+Angle_mean_10max+ (1|Replicate),
family = binomial, data = tab)
fm12b <- glmer(cbind(emerged,unemerged) ~ Irradiation + Temp_mean
+changescalar_mean_10max+ scalar_mean_10mean +(1|Replicate), family =
binomial, data = tab)
fm13c <- glmer(cbind(emerged,unemerged) ~ Irradiation + Temp_mean +
Duration.ms._10max + changescalar_mean_10max+ (1|Replicate), family =
binomial, data = tab)
fm14 <- glmer(cbind(emerged,unemerged) ~ Irradiation + Duration.ms._10max +
scalar_mean_10mean +(1|Replicate), family = binomial, data = tab)
fm15 <- glmer(cbind(emerged,unemerged) ~ Irradiation +
```

```
changescalar_mean_10max+ scalar_mean_10mean +(1|Replicate), family =
binomial, data = tab)
fm16 <- glmer(cbind(emerged,unemerged) ~ Irradiation + Temp_mean +
Duration.ms._10max + Angle_mean_10max+ (1|Replicate), family = binomial, data
= tab)
fm17 <- glmer(cbind(emerged,unemerged) ~ Irradiation +  scalar_mean_10mean
+Angle_mean_10max+  (1|Replicate), family = binomial, data = tab)
fm18 <- glmer(cbind(emerged,unemerged) ~ Irradiation + Age +
changescalar_mean_10max+ scalar_mean_10mean +(1|Replicate), family =
binomial, data = tab)
fm19 <- glmer(cbind(emerged,unemerged) ~ Irradiation + Duration.ms._10max +
Age + (1|Replicate), family = binomial, data = tab)
fm14a <- glmer(cbind(emerged,unemerged) ~ Irradiation + Duration.ms._10max +
(1|Replicate), family = binomial, data = tab)
fm14b <- glmer(cbind(emerged,unemerged) ~ Irradiation + Duration.ms._10max +
changescalar_mean_10max+ Age +(1|Replicate), family = binomial, data = tab)
fm14c <- glmer(cbind(emerged,unemerged) ~ Irradiation +
changescalar_mean_10max+ (1|Replicate), family = binomial, data = tab)


AICc(fm1,fm2,fm3,fm4,fm5,fm6,fm7,fm9,fm10,fm11,fm12,fm13,fm11a,fm12b,fm13c,fm
14,fm14a,fm14b,fm14c,fm15,fm16,fm17,fm18,fm19)

##       df     AICc
## fm1    5 279.1278
## fm2    7 210.0473
## fm3    7 242.4495
## fm4    6 250.4973
## fm5    6 222.9305
## fm6    6 207.1041
## fm7    7 210.0473
## fm9    5 247.0403
## fm10   7 242.4495
## fm11   5 279.1278
## fm12   5 283.2114
## fm13   5 279.0186
## fm11a  7 233.0312
## fm12b  6 273.4480
## fm13c  6 239.9159
## fm14   5 259.0828
## fm14a  4 255.7054
## fm14b  6 244.4702
## fm14c  4 280.4184
## fm15   5 270.3035
## fm16   6 228.9594
## fm17   5 269.0142
## fm18   6 274.0456
## fm19   5 253.5503

summary(fm6)
```

```
## Generalized linear mixed model fit by maximum likelihood (Laplace
##   Approximation) [glmerMod]
##  Family: binomial  ( logit )
## Formula:
## cbind(emerged, unemerged) ~ Irradiation + Temp_mean +
changescalar_mean_5max +
##     scalar_max_5mean + (1 | Replicate)
##    Data: tab
##
##      AIC      BIC   logLik deviance df.resid
##    201.5    208.1    -94.8    189.5       16
##
## Scaled residuals:
##     Min      1Q  Median      3Q     Max
## -2.4604 -0.7569  0.1291  0.8611  2.0722
##
## Random effects:
##  Groups    Name        Variance Std.Dev.
##  Replicate (Intercept) 0.9111   0.9545
## Number of obs: 22, groups:  Replicate, 6
##
## Fixed effects:
##                        Estimate Std. Error z value Pr(>|z|)
## (Intercept)             7.99460    0.98899   8.084 6.29e-16 ***
## Irradiationyes         -0.18515    0.06827  -2.712  0.00669 **
## Temp_mean              -0.10359    0.02511  -4.126 3.69e-05 ***
## changescalar_mean_5max -0.08900    0.01937  -4.594 4.35e-06 ***
## scalar_max_5mean       -0.41860    0.06381  -6.561 5.36e-11 ***
## ---
## Signif. codes:  0 '***' 0.001 '**' 0.01 '*' 0.05 '.' 0.1 ' ' 1
##
## Correlation of Fixed Effects:
##             (Intr) Irrdtn Tmp_mn chn__5
## Irradiatnys -0.029
## Temp_mean   -0.694 -0.027
## chngsclr__5 -0.634 -0.019  0.954
## sclr_mx_5mn -0.562  0.020 -0.045 -0.132
```

```
fm21 <- glmer(cbind(emerged,unemerged) ~ Irradiation + Temp_mean +
changescalar_mean_15max + Angle_mean_15max+
changescalar_mean_15mean+Changevector_max_15mean+ (1|Replicate), family =
binomial, data = tab)
fm22 <- glmer(cbind(emerged,unemerged) ~ Irradiation + Temp_mean +
changescalar_mean_15max + Changevector_mean_15max+ Angle_mean_15max+
(1|Replicate), family = binomial, data = tab)
fm23 <- glmer(cbind(emerged,unemerged) ~ Irradiation +
changescalar_mean_15max + Changevector_mean_15max+ Angle_mean_15max+
(1|Replicate), family = binomial, data = tab)
fm24 <- glmer(cbind(emerged,unemerged) ~ Irradiation + Temp_mean
+changescalar_mean_15max + changescalar_mean_15mean+  (1|Replicate), family =
```

```
binomial, data = tab)
fm25 <- glmer(cbind(emerged,unemerged) ~ Irradiation +
changescalar_mean_15max + Age+ Angle_mean_15max+ (1|Replicate), family =
binomial, data = tab)
fm26 <- glmer(cbind(emerged,unemerged) ~ Irradiation +
changescalar_mean_15max + scalar_mean_15mean + (1|Replicate), family =
binomial, data = tab)
fm27 <- glmer(cbind(emerged,unemerged) ~ Irradiation +
Changevector_mean_15max+Angle_mean_15max+scalar_mean_15mean
+Changevector_max_15mean+ (1|Replicate), family = binomial, data = tab)
fm28 <- glmer(cbind(emerged,unemerged) ~ Irradiation +
Changevector_mean_15max+(1|Replicate), family = binomial, data = tab)
fm29 <- glmer(cbind(emerged,unemerged) ~ Irradiation +
changescalar_mean_15max + Changevector_mean_15max+ (1|Replicate), family =
binomial, data = tab)
fm30 <- glmer(cbind(emerged,unemerged) ~ Irradiation + scalar_mean_15mean +
Age +(1|Replicate), family = binomial, data = tab)
fm21a <- glmer(cbind(emerged,unemerged) ~ Irradiation + Temp_mean +
Changevector_mean_15max+ Angle_mean_15max+changescalar_mean_15mean+
(1|Replicate), family = binomial, data = tab)
fm21b <- glmer(cbind(emerged,unemerged) ~ Irradiation + Temp_mean +
changescalar_mean_15max + scalar_mean_15mean +Changevector_max_15mean+
(1|Replicate), family = binomial, data = tab)
fm21c <- glmer(cbind(emerged,unemerged) ~ Irradiation + Temp_mean +
changescalar_mean_15max + Angle_mean_15max+ (1|Replicate), family = binomial,
data = tab)
fm21d <- glmer(cbind(emerged,unemerged) ~ Irradiation + Temp_mean
+changescalar_mean_15mean+Changevector_max_15mean+ (1|Replicate), family =
binomial, data = tab)
fm21e <- glmer(cbind(emerged,unemerged) ~ Irradiation + Temp_mean
+changescalar_mean_15mean+Changevector_max_15mean+ Age +(1|Replicate), family
= binomial, data = tab)
fm27a <- glmer(cbind(emerged,unemerged) ~ Irradiation + scalar_mean_15mean
+Changevector_max_15mean+ (1|Replicate), family = binomial, data = tab)
fm27b <- glmer(cbind(emerged,unemerged) ~ Irradiation +
Changevector_mean_15max+Angle_mean_15max+scalar_mean_15mean + (1|Replicate),
family = binomial, data = tab)
fm29a <- glmer(cbind(emerged,unemerged) ~ Irradiation +
Changevector_mean_15max+ (1|Replicate), family = binomial, data = tab)



AICc(fm1,fm2,fm3,fm4,fm5,fm6,fm7,fm9,fm10,fm11,fm12,fm13,fm11a,fm12b,fm13c,fm
14,fm14a,fm14b,fm14c,fm15,fm16,fm17,fm18,fm19,fm21,fm22,fm23,fm24,fm25,fm26,f
m27,fm28,fm29,fm29a,fm30,fm21a,fm21b,fm21c,fm21d,fm21e,fm27a,fm27b)

##        df     AICc
## fm1     5 279.1278
## fm2     7 210.0473
## fm3     7 242.4495
```

```
## fm4    6 250.4973
## fm5    6 222.9305
## fm6    6 207.1041
## fm7    7 210.0473
## fm9    5 247.0403
## fm10   7 242.4495
## fm11   5 279.1278
## fm12   5 283.2114
## fm13   5 279.0186
## fm11a  7 233.0312
## fm12b  6 273.4480
## fm13c  6 239.9159
## fm14   5 259.0828
## fm14a  4 255.7054
## fm14b  6 244.4702
## fm14c  4 280.4184
## fm15   5 270.3035
## fm16   6 228.9594
## fm17   5 269.0142
## fm18   6 274.0456
## fm19   5 253.5503
## fm21   8 209.8333
## fm22   7 184.6451
## fm23   6 182.1976
## fm24   6 223.7014
## fm25   6 216.1388
## fm26   5 253.4476
## fm27   7 196.9255
## fm28   4 239.6031
## fm29   5 179.1671
## fm29a  4 239.6031
## fm30   5 277.3387
## fm21a  7 192.7703
## fm21b  7 229.4084
## fm21c  6 225.6124
## fm21d  6 270.0999
## fm21e  7 266.0208
## fm27a  5 289.3100
## fm27b  6 201.8145
```

```
summary(fm29)
```

```
## Generalized linear mixed model fit by maximum likelihood (Laplace
##   Approximation) [glmerMod]
##  Family: binomial  ( logit )
## Formula: cbind(emerged, unemerged) ~ Irradiation + changescalar_mean_15max
+
##     Changevector_mean_15max + (1 | Replicate)
##    Data: tab
##
```

```
##      AIC      BIC   logLik deviance df.resid
##    175.4    180.9    -82.7    165.4       17
##
## Scaled residuals:
##      Min       1Q   Median       3Q      Max
## -2.18179 -0.47932  0.07369  0.79921  2.05772
##
## Random effects:
##  Groups    Name        Variance Std.Dev.
##  Replicate (Intercept) 0.1613   0.4016
## Number of obs: 22, groups:  Replicate, 6
##
## Fixed effects:
##                        Estimate Std. Error z value Pr(>|z|)
## (Intercept)             2.05351    0.22312   9.203  < 2e-16 ***
## Irradiationyes         -0.18444    0.06835  -2.698  0.00697 **
## changescalar_mean_15max  0.24576    0.03103   7.921 2.36e-15 ***
## Changevector_mean_15max -0.26529    0.02850  -9.307  < 2e-16 ***
## ---
## Signif. codes:  0 '***' 0.001 '**' 0.01 '*' 0.05 '.' 0.1 ' ' 1
##
## Correlation of Fixed Effects:
##             (Intr) Irrdtn ch__15
## Irradiatnys -0.170
## chngscl__15  0.167  0.009
## Chngvct__15 -0.305 -0.001 -0.973
```

```
fm31 <- glmer(cbind(emerged,unemerged) ~ Irradiation + Temp_mean +
scalar_mean_20max + changescalar_mean_20mean+ (1|Replicate), family =
binomial, data = tab)
fm32 <- glmer(cbind(emerged,unemerged) ~ Irradiation + Temp_mean +
changescalar_mean_20mean+ (1|Replicate), family = binomial, data = tab)
fm31a <- glmer(cbind(emerged,unemerged) ~ Irradiation + Temp_mean +
scalar_mean_20max + (1|Replicate), family = binomial, data = tab)

AICc(fm1,fm2,fm3,fm4,fm5,fm6,fm7,fm9,fm10,fm11,fm12,fm13,fm11a,fm12b,fm13c,fm
14,fm14a,fm14b,fm14c,fm15,fm16,fm17,fm18,fm19,fm21,fm22,fm23,fm24,fm25,fm26,f
m27,fm28,fm29,fm29a,fm30,fm21a,fm21b,fm21c,fm21d,fm21e,fm27a,fm27b,fm31,fm31a
,fm32)
```

```
##        df     AICc
## fm1     5 279.1278
## fm2     7 210.0473
## fm3     7 242.4495
## fm4     6 250.4973
## fm5     6 222.9305
## fm6     6 207.1041
## fm7     7 210.0473
## fm9     5 247.0403
## fm10    7 242.4495
```

```
## fm11   5 279.1278
## fm12   5 283.2114
## fm13   5 279.0186
## fm11a  7 233.0312
## fm12b  6 273.4480
## fm13c  6 239.9159
## fm14   5 259.0828
## fm14a  4 255.7054
## fm14b  6 244.4702
## fm14c  4 280.4184
## fm15   5 270.3035
## fm16   6 228.9594
## fm17   5 269.0142
## fm18   6 274.0456
## fm19   5 253.5503
## fm21   8 209.8333
## fm22   7 184.6451
## fm23   6 182.1976
## fm24   6 223.7014
## fm25   6 216.1388
## fm26   5 253.4476
## fm27   7 196.9255
## fm28   4 239.6031
## fm29   5 179.1671
## fm29a  4 239.6031
## fm30   5 277.3387
## fm21a  7 192.7703
## fm21b  7 229.4084
## fm21c  6 225.6124
## fm21d  6 270.0999
## fm21e  7 266.0208
## fm27a  5 289.3100
## fm27b  6 201.8145
## fm31   6 274.7091
## fm31a  5 272.3238
## fm32   5 283.2503
```

```
summary(fm29)
```

```
## Generalized linear mixed model fit by maximum likelihood (Laplace
##   Approximation) [glmerMod]
##  Family: binomial  ( logit )
## Formula: cbind(emerged, unemerged) ~ Irradiation + changescalar_mean_15max
+
##     Changevector_mean_15max + (1 | Replicate)
##    Data: tab
##
##      AIC      BIC   logLik deviance df.resid
##    175.4    180.9    -82.7    165.4       17
##
```

```
## Scaled residuals:
##      Min       1Q   Median       3Q      Max
## -2.18179 -0.47932  0.07369  0.79921  2.05772
##
## Random effects:
##  Groups    Name        Variance Std.Dev.
##  Replicate (Intercept) 0.1613   0.4016
## Number of obs: 22, groups:  Replicate, 6
##
## Fixed effects:
##                            Estimate Std. Error z value Pr(>|z|)
## (Intercept)                 2.05351    0.22312   9.203  < 2e-16 ***
## Irradiationyes             -0.18444    0.06835  -2.698  0.00697 **
## changescalar_mean_15max     0.24576    0.03103   7.921 2.36e-15 ***
## Changevector_mean_15max    -0.26529    0.02850  -9.307  < 2e-16 ***
## ---
## Signif. codes:  0 '***' 0.001 '**' 0.01 '*' 0.05 '.' 0.1 ' ' 1
##
## Correlation of Fixed Effects:
##             (Intr) Irrdtn ch__15
## Irradiatnys -0.170
## chngscl__15  0.167  0.009
## Chngvct__15 -0.305 -0.001 -0.973
```

```
#combination of thresholds
fm41 <- glmer(cbind(emerged,unemerged) ~ Irradiation + Temp_mean +
Duration.ms._10max + scalar_max_5mean +
Changevector_mean_15max+(1|Replicate), family = binomial, data = tab)
fm42 <- glmer(cbind(emerged,unemerged) ~ Irradiation + scalar_max_5mean +
Changevector_mean_15max+ (1|Replicate), family = binomial, data = tab)
fm41a <- glmer(cbind(emerged,unemerged) ~ Irradiation + Temp_mean +
Duration.ms._10max + changescalar_mean_5max + (1|Replicate), family =
binomial, data = tab)
fm41b <- glmer(cbind(emerged,unemerged) ~ Irradiation + Temp_mean +
Duration.ms._10max + scalar_max_5mean + (1|Replicate), family = binomial,
data = tab)


AICc(fm1,fm2,fm3,fm4,fm5,fm6,fm7,fm9,fm10,fm11,fm12,fm13,fm11a,fm12b,fm13c,fm
14,fm14a,fm14b,fm14c,fm15,fm16,fm17,fm18,fm19,fm21,fm22,fm23,fm24,fm25,fm26,f
m27,fm28,fm29,fm29a,fm30,fm21a,fm21b,fm21c,fm21d,fm21e,fm27a,fm27b,fm31,fm31a
,fm32,fm41,fm42,fm41a,fm41b)
```

```
##       df     AICc
## fm1    5 279.1278
## fm2    7 210.0473
## fm3    7 242.4495
## fm4    6 250.4973
## fm5    6 222.9305
## fm6    6 207.1041
```

```
## fm7    7 210.0473
## fm9    5 247.0403
## fm10   7 242.4495
## fm11   5 279.1278
## fm12   5 283.2114
## fm13   5 279.0186
## fm11a  7 233.0312
## fm12b  6 273.4480
## fm13c  6 239.9159
## fm14   5 259.0828
## fm14a  4 255.7054
## fm14b  6 244.4702
## fm14c  4 280.4184
## fm15   5 270.3035
## fm16   6 228.9594
## fm17   5 269.0142
## fm18   6 274.0456
## fm19   5 253.5503
## fm21   8 209.8333
## fm22   7 184.6451
## fm23   6 182.1976
## fm24   6 223.7014
## fm25   6 216.1388
## fm26   5 253.4476
## fm27   7 196.9255
## fm28   4 239.6031
## fm29   5 179.1671
## fm29a  4 239.6031
## fm30   5 277.3387
## fm21a  7 192.7703
## fm21b  7 229.4084
## fm21c  6 225.6124
## fm21d  6 270.0999
## fm21e  7 266.0208
## fm27a  5 289.3100
## fm27b  6 201.8145
## fm31   6 274.7091
## fm31a  5 272.3238
## fm32   5 283.2503
## fm41   7 187.7135
## fm42   5 213.5166
## fm41a  6 249.9178
## fm41b  6 211.0242
```

```
summary(fm29)
```

```
## Generalized linear mixed model fit by maximum likelihood (Laplace
##   Approximation) [glmerMod]
##  Family: binomial  ( logit )
## Formula: cbind(emerged, unemerged) ~ Irradiation + changescalar_mean_15max
```

```
+
##     Changevector_mean_15max + (1 | Replicate)
##    Data: tab
##
##      AIC      BIC   logLik deviance df.resid
##    175.4    180.9    -82.7    165.4       17
##
## Scaled residuals:
##      Min       1Q   Median       3Q      Max
## -2.18179 -0.47932  0.07369  0.79921  2.05772
##
## Random effects:
##  Groups    Name        Variance Std.Dev.
##  Replicate (Intercept) 0.1613   0.4016
## Number of obs: 22, groups:  Replicate, 6
##
## Fixed effects:
##                           Estimate Std. Error z value Pr(>|z|)
## (Intercept)                2.05351    0.22312   9.203  < 2e-16 ***
## Irradiationyes            -0.18444    0.06835  -2.698  0.00697 **
## changescalar_mean_15max  0.24576    0.03103   7.921 2.36e-15 ***
## Changevector_mean_15max -0.26529    0.02850  -9.307  < 2e-16 ***
## ---
## Signif. codes:  0 '***' 0.001 '**' 0.01 '*' 0.05 '.' 0.1 ' ' 1
##
## Correlation of Fixed Effects:
##             (Intr) Irrdtn ch__15
## Irradiatnys -0.170
## chngscl__15  0.167  0.009
## Chngvct__15 -0.305 -0.001 -0.973

#the best model remains as fm29 (s12 fig)

##Fig9a
head(tab)

##     Date Replicate Age     Treatments emerged unemerged shipped
Irradiation
## 1 170510        R1  22 Shipped-110Gy      125       100     yes
yes
## 2 170510        R1  22   Shipped-0Gy      126        99     yes
no
## 3 170517        R2  22 Shipped-110Gy       91       134     yes
yes
## 4 170517        R2  22   Shipped-0Gy       88       137     yes
no
## 5 170524        R3  22 Shipped-110Gy      173        64     yes
yes
## 6 170524        R3  22   Shipped-0Gy      183        54     yes
no
```

```
##   Events._5max Duration.ms._5max scalar_max_5max scalar_mean_5max
## 1          909            48.750          27.374         21.43900
## 2          909            48.750          27.374         21.43900
## 3          708            87.500          27.398         16.92723
## 4          708            87.500          27.398         16.92723
## 5         1187            93.125          27.374         15.43705
## 6         1187            93.125          27.374         15.43705
##   changescalar_max_5max changescalar_mean_5max Changevector_max_5max
## 1                23.724                8.94875              27.69156
## 2                23.724                8.94875              27.69156
## 3                24.477               11.37550              35.06987
## 4                24.477               11.37550              35.06987
## 5                16.627                3.83800              33.00443
## 6                16.627                3.83800              33.00443
##   Changevector_mean_5max Angle_max_5max Angle_mean_5max Hz_max_5max
## 1               9.937386       3.002116        2.617132    764.4826
## 2               9.937386       3.002116        2.617132    764.4826
## 3              19.035967       2.969360        1.679485    756.1414
## 4              19.035967       2.969360        1.679485    756.1414
## 5               5.064434       2.105922        1.356174    536.2686
## 6               5.064434       2.105922        1.356174    536.2686
##   Hz_mean_5max Events_10max Duration.ms._10max scalar_max_10max
## 1     666.4471          288             36.875           27.374
## 2     666.4471          288             36.875           27.374
## 3     427.6775          315             50.000           27.398
## 4     427.6775          315             50.000           27.398
## 5     345.3468          238             63.750           27.374
## 6     345.3468          238             63.750           27.374
##   scalar_mean_10max changescalar_max_10max changescalar_mean_10max
## 1           21.5800                 23.724                  12.473
## 2           21.5800                 23.724                  12.473
## 3           20.3750                 24.477                  16.488
## 4           20.3750                 24.477                  16.488
## 5           19.6895                 16.627                   8.965
## 6           19.6895                 16.627                   8.965
##   Changevector_max_10max Changevector_mean_10max Angle_max_10max
## 1               27.69156                15.45774        2.687392
## 2               27.69156                15.45774        2.687392
## 3               35.06987                22.58486        2.969360
## 4               35.06987                22.58486        2.969360
## 5               33.00443                10.65686        2.105922
## 6               33.00443                10.65686        2.105922
##   Angle_mean_10max Hz_max_10max Hz_mean_10max Events_15max
Duration.ms._15max
## 1        1.0470779     684.3388      266.6362          144
30.625
## 2        1.0470779     684.3388      266.6362          144
30.625
## 3        1.6138687     756.1414      410.9683          183
29.375
```

```
## 4        1.6138687     756.1414      410.9683          183
29.375
## 5        0.5851573     536.2686      149.0091          111
41.250
## 6        0.5851573     536.2686      149.0091          111
41.250
##   scalar_max_15max scalar_mean_15max changescalar_max_15max
## 1           27.374          22.42986                 23.724
## 2           27.374          22.42986                 23.724
## 3           27.398          21.71558                 24.477
## 4           27.398          21.71558                 24.477
## 5           27.374          22.77170                 16.627
## 6           27.374          22.77170                 16.627
##   changescalar_mean_15max Changevector_max_15max Changevector_mean_15max
## 1                  15.667               27.69156                19.75723
## 2                  15.667               27.69156                19.75723
## 3                  16.488               35.06987                21.61531
## 4                  16.488               35.06987                21.61531
## 5                  11.474               33.00443                12.83840
## 6                  11.474               33.00443                12.83840
##   Angle_max_15max Angle_mean_15max Hz_max_15max Hz_mean_15max Events_20max
## 1        2.687392        1.8427590     684.3388      469.2547           57
## 2        2.687392        1.8427590     684.3388      469.2547           57
## 3        2.969360        1.6010884     756.1414      407.7138           75
## 4        2.969360        1.6010884     756.1414      407.7138           75
## 5        2.105922        0.6709018     536.2686      170.8438           42
## 6        2.105922        0.6709018     536.2686      170.8438           42
##   Duration.ms._20max scalar_max_20max scalar_mean_20max
changescalar_max_20max
## 1             17.500           27.374          26.80175
23.724
## 2             17.500           27.374          26.80175
23.724
## 3             20.625           27.398          26.38567
24.477
## 4             20.625           27.398          26.38567
24.477
## 5             20.625           27.374          25.17900
13.480
## 6             20.625           27.374          25.17900
13.480
##   changescalar_mean_20max Changevector_max_20max Changevector_mean_20max
## 1                 23.7240               27.69156                27.69156
## 2                 23.7240               27.69156                27.69156
## 3                 13.7095               35.06987                29.65156
## 4                 13.7095               35.06987                29.65156
## 5                  6.6090               33.00443                11.19072
## 6                  6.6090               33.00443                11.19072
##   Angle_max_20max Angle_mean_20max Hz_max_20max Hz_mean_20max Events_5mean
## 1        2.687392         2.687392     684.3388      684.3388          909
```

```
## 2       2.687392         2.687392     684.3388      684.3388           909
## 3       2.433775         1.792941     619.7557      456.5688           708
## 4       2.433775         1.792941     619.7557      456.5688           708
## 5       1.715913         0.489164     436.9538      124.5646          1187
## 6       1.715913         0.489164     436.9538      124.5646          1187
##   Duration.ms._5mean scalar_max_5mean scalar_mean_5mean
changescalar_max_5mean
## 1           5.215209         9.516648          7.473747
3.755884
## 2           5.215209         9.516648          7.473747
3.755884
## 3          11.785840        10.551797          7.565272
2.816095
## 4          11.785840        10.551797          7.565272
2.816095
## 5           7.348884         8.022198          6.506753
1.632164
## 6           7.348884         8.022198          6.506753
1.632164
##   changescalar_mean_5mean Changevector_max_5mean Changevector_mean_5mean
## 1               1.6630629               4.501180                2.219564
## 2               1.6630629               4.501180                2.219564
## 3               1.0076953               3.691769                1.638802
## 4               1.0076953               3.691769                1.638802
## 5               0.6804169               2.293485                1.194539
## 6               0.6804169               2.293485                1.194539
##   Angle_max_5mean Angle_mean_5mean Hz_max_5mean Hz_mean_5mean
Events._mean10
## 1       0.4296757        0.1755708    109.41603      44.70874
288
## 2       0.4296757        0.1755708    109.41603      44.70874
288
## 3       0.3715939        0.1414327     94.62562      36.01553
315
## 4       0.3715939        0.1414327     94.62562      36.01553
315
## 5       0.2539263        0.1310499     64.66179      33.37158
238
## 6       0.2539263        0.1310499     64.66179      33.37158
238
##   Duration.ms._10mean scalar_max_10mean scalar_mean_10mean
## 1            3.888889          15.49537           13.49979
## 2            3.888889          15.49537           13.49979
## 3            7.706349          16.38244           13.65682
## 4            7.706349          16.38244           13.65682
## 5            7.415966          15.56483           13.15894
## 6            7.415966          15.56483           13.15894
##   changescalar_max_10mean changescalar_mean_10mean Changevector_max_10mean
## 1                6.557340                 3.249119                8.029862
## 2                6.557340                 3.249119                8.029862
```

```
## 3                4.928854                  1.971893                  6.505797
## 4                4.928854                  1.971893                  6.505797
## 5                4.004025                  1.664277                  5.891075
## 6                4.004025                  1.664277                  5.891075
##   Changevector_mean_10mean Angle_max_10mean Angle_mean_10mean
Hz_max_10mean
## 1                 4.538349        0.4719538         0.2224823
120.1821
## 2                 4.538349        0.4719538         0.2224823
120.1821
## 3                 3.339135        0.4047630         0.1751144
103.0721
## 4                 3.339135        0.4047630         0.1751144
103.0721
## 5                 2.909783        0.3492638         0.1583697
88.9393
## 6                 2.909783        0.3492638         0.1583697
88.9393
##   Hz_mean_10mean Events_15mean Duration.ms._15mean scalar_max_15mean
## 1       56.65466           144            3.441840          19.26462
## 2       56.65466           144            3.441840          19.26462
## 3       44.59251           183            6.342213          19.93954
## 4       44.59251           183            6.342213          19.93954
## 5       40.32851           111            6.920045          19.71768
## 6       40.32851           111            6.920045          19.71768
##   scalar_mean_15mean changescalar_max_15mean changescalar_mean_15mean
## 1           17.94158                8.195410                 4.209463
## 2           17.94158                8.195410                 4.209463
## 3           17.79343                6.271208                 2.424063
## 4           17.79343                6.271208                 2.424063
## 5           17.60073                5.291784                 2.055466
## 6           17.60073                5.291784                 2.055466
##   Changevector_max_15mean Changevector_mean_15mean Angle_max_15mean
## 1               10.145769                 6.194357        0.4732660
## 2               10.145769                 6.194357        0.4732660
## 3                8.773143                 4.485467        0.4584332
## 4                8.773143                 4.485467        0.4584332
## 5                8.644509                 4.178605        0.4437400
## 6                8.644509                 4.178605        0.4437400
##   Angle_mean_15mean Hz_max_15mean Hz_mean_15mean Events_20mean
## 1         0.2644405      120.5162       67.33922            57
## 2         0.2644405      120.5162       67.33922            57
## 3         0.2042285      116.7390       52.00635            75
## 4         0.2042285      116.7390       52.00635            75
## 5         0.1960600      112.9975       49.92626            42
## 6         0.1960600      112.9975       49.92626            42
##   Duration.ms._20mean scalar_max_20mean scalar_mean_20mean
## 1            2.587719          22.76440           22.04857
## 2            2.587719          22.76440           22.04857
## 3            5.250000          23.71752           22.29175
```

```
## 4           5.250000         23.71752           22.29175
## 5           5.997024         24.28693           22.58697
## 6           5.997024         24.28693           22.58697
##   changescalar_max_20mean changescalar_mean_20mean Changevector_max_20mean
## 1                7.502070                 3.960703                11.35123
## 2                7.502070                 3.960703                11.35123
## 3                6.190293                 2.676377                11.36833
## 4                6.190293                 2.676377                11.36833
## 5                5.996190                 2.445063                11.97291
## 6                5.996190                 2.445063                11.97291
##   Changevector_mean_20mean Angle_max_20mean Angle_mean_20mean
Hz_max_20mean
## 1                 6.922532        0.5201012         0.3140733
132.4427
## 2                 6.922532        0.5201012         0.3140733
132.4427
## 3                 5.877764        0.5214004         0.2548023
132.7735
## 4                 5.877764        0.5214004         0.2548023
132.7735
## 5                 5.476046        0.5391966         0.2201107
137.3053
## 6                 5.476046        0.5391966         0.2201107
137.3053
##   Hz_mean_20mean RH._Max Temp_Max RH._mean Temp_mean   pcemerg
## 1       79.97812   47.08    23.50    41.69     19.73 0.5555556
## 2       79.97812   60.14    27.50    41.69     19.73 0.5600000
## 3       64.88487   62.61    29.75    53.55     21.79 0.4044444
## 4       64.88487   68.10    32.00    53.55     21.79 0.3911111
## 5       56.05073   57.37    28.69    53.25     23.29 0.7299578
## 6       56.05073   74.59    19.25    53.25     23.29 0.7721519
```

```
str(tab)
```

```
## 'data.frame':    22 obs. of  109 variables:
##  $ Date                    : int  170510 170510 170517 170517 170524
170524 170531 170531 170607 170607 ...
##  $ Replicate               : chr  "R1" "R1" "R2" "R2" ...
##  $ Age                     : int  22 22 22 22 22 22 22 22 22 22 ...
##  $ Treatments              : chr  "Shipped-110Gy " "Shipped-0Gy "
"Shipped-110Gy " "Shipped-0Gy " ...
##  $ emerged                 : int  125 126 91 88 173 183 173 178 181 189
...
##  $ unemerged               : int  100 99 134 137 64 54 52 47 44 36 ...
##  $ shipped                 : chr  "yes" "yes" "yes" "yes" ...
##  $ Irradiation             : chr  "yes" "no" "yes" "no" ...
##  $ Events._5max            : int  909 909 708 708 1187 1187 625 625 606
606 ...
##  $ Duration.ms._5max       : num  48.8 48.8 87.5 87.5 93.1 ...
##  $ scalar_max_5max         : num  27.4 27.4 27.4 27.4 27.4 ...
```

```
##  $ scalar_mean_5max        : num  21.4 21.4 16.9 16.9 15.4 ...
##  $ changescalar_max_5max   : num  23.7 23.7 24.5 24.5 16.6 ...
##  $ changescalar_mean_5max  : num  8.95 8.95 11.38 11.38 3.84 ...
##  $ Changevector_max_5max   : num  27.7 27.7 35.1 35.1 33 ...
##  $ Changevector_mean_5max  : num  9.94 9.94 19.04 19.04 5.06 ...
##  $ Angle_max_5max          : num  3 3 2.97 2.97 2.11 ...
##  $ Angle_mean_5max         : num  2.62 2.62 1.68 1.68 1.36 ...
##  $ Hz_max_5max             : num  764 764 756 756 536 ...
##  $ Hz_mean_5max            : num  666 666 428 428 345 ...
##  $ Events_10max            : int  288 288 315 315 238 238 261 261 219 219
...
##  $ Duration.ms._10max      : num  36.9 36.9 50 50 63.8 ...
##  $ scalar_max_10max        : num  27.4 27.4 27.4 27.4 27.4 ...
##  $ scalar_mean_10max       : num  21.6 21.6 20.4 20.4 19.7 ...
##  $ changescalar_max_10max  : num  23.7 23.7 24.5 24.5 16.6 ...
##  $ changescalar_mean_10max : num  12.47 12.47 16.49 16.49 8.97 ...
##  $ Changevector_max_10max  : num  27.7 27.7 35.1 35.1 33 ...
##  $ Changevector_mean_10max : num  15.5 15.5 22.6 22.6 10.7 ...
##  $ Angle_max_10max         : num  2.69 2.69 2.97 2.97 2.11 ...
##  $ Angle_mean_10max        : num  1.047 1.047 1.614 1.614 0.585 ...
##  $ Hz_max_10max            : num  684 684 756 756 536 ...
##  $ Hz_mean_10max           : num  267 267 411 411 149 ...
##  $ Events_15max            : int  144 144 183 183 111 111 163 163 129 129
...
##  $ Duration.ms._15max      : num  30.6 30.6 29.4 29.4 41.2 ...
##  $ scalar_max_15max        : num  27.4 27.4 27.4 27.4 27.4 ...
##  $ scalar_mean_15max       : num  22.4 22.4 21.7 21.7 22.8 ...
##  $ changescalar_max_15max  : num  23.7 23.7 24.5 24.5 16.6 ...
##  $ changescalar_mean_15max : num  15.7 15.7 16.5 16.5 11.5 ...
##  $ Changevector_max_15max  : num  27.7 27.7 35.1 35.1 33 ...
##  $ Changevector_mean_15max : num  19.8 19.8 21.6 21.6 12.8 ...
##  $ Angle_max_15max         : num  2.69 2.69 2.97 2.97 2.11 ...
##  $ Angle_mean_15max        : num  1.843 1.843 1.601 1.601 0.671 ...
##  $ Hz_max_15max            : num  684 684 756 756 536 ...
##  $ Hz_mean_15max           : num  469 469 408 408 171 ...
##  $ Events_20max            : int  57 57 75 75 42 42 97 97 71 71 ...
##  $ Duration.ms._20max      : num  17.5 17.5 20.6 20.6 20.6 ...
##  $ scalar_max_20max        : num  27.4 27.4 27.4 27.4 27.4 ...
##  $ scalar_mean_20max       : num  26.8 26.8 26.4 26.4 25.2 ...
##  $ changescalar_max_20max  : num  23.7 23.7 24.5 24.5 13.5 ...
##  $ changescalar_mean_20max : num  23.72 23.72 13.71 13.71 6.61 ...
##  $ Changevector_max_20max  : num  27.7 27.7 35.1 35.1 33 ...
##  $ Changevector_mean_20max : num  27.7 27.7 29.7 29.7 11.2 ...
##  $ Angle_max_20max         : num  2.69 2.69 2.43 2.43 1.72 ...
##  $ Angle_mean_20max        : num  2.687 2.687 1.793 1.793 0.489 ...
##  $ Hz_max_20max            : num  684 684 620 620 437 ...
##  $ Hz_mean_20max           : num  684 684 457 457 125 ...
##  $ Events_5mean            : int  909 909 708 708 1187 1187 625 625 606
606 ...
##  $ Duration.ms._5mean      : num  5.22 5.22 11.79 11.79 7.35 ...
```

```
##  $ scalar_max_5mean        : num  9.52 9.52 10.55 10.55 8.02 ...
##  $ scalar_mean_5mean       : num  7.47 7.47 7.57 7.57 6.51 ...
##  $ changescalar_max_5mean  : num  3.76 3.76 2.82 2.82 1.63 ...
##  $ changescalar_mean_5mean : num  1.66 1.66 1.01 1.01 0.68 ...
##  $ Changevector_max_5mean  : num  4.5 4.5 3.69 3.69 2.29 ...
##  $ Changevector_mean_5mean : num  2.22 2.22 1.64 1.64 1.19 ...
##  $ Angle_max_5mean         : num  0.43 0.43 0.372 0.372 0.254 ...
##  $ Angle_mean_5mean        : num  0.176 0.176 0.141 0.141 0.131 ...
##  $ Hz_max_5mean            : num  109.4 109.4 94.6 94.6 64.7 ...
##  $ Hz_mean_5mean           : num  44.7 44.7 36 36 33.4 ...
##  $ Events._mean10          : int  288 288 315 315 238 238 261 261 219 219
...
##  $ Duration.ms._10mean     : num  3.89 3.89 7.71 7.71 7.42 ...
##  $ scalar_max_10mean       : num  15.5 15.5 16.4 16.4 15.6 ...
##  $ scalar_mean_10mean      : num  13.5 13.5 13.7 13.7 13.2 ...
##  $ changescalar_max_10mean : num  6.56 6.56 4.93 4.93 4 ...
##  $ changescalar_mean_10mean: num  3.25 3.25 1.97 1.97 1.66 ...
##  $ Changevector_max_10mean : num  8.03 8.03 6.51 6.51 5.89 ...
##  $ Changevector_mean_10mean: num  4.54 4.54 3.34 3.34 2.91 ...
##  $ Angle_max_10mean        : num  0.472 0.472 0.405 0.405 0.349 ...
##  $ Angle_mean_10mean       : num  0.222 0.222 0.175 0.175 0.158 ...
##  $ Hz_max_10mean           : num  120.2 120.2 103.1 103.1 88.9 ...
##  $ Hz_mean_10mean          : num  56.7 56.7 44.6 44.6 40.3 ...
##  $ Events_15mean           : int  144 144 183 183 111 111 163 163 129 129
...
##  $ Duration.ms._15mean     : num  3.44 3.44 6.34 6.34 6.92 ...
##  $ scalar_max_15mean       : num  19.3 19.3 19.9 19.9 19.7 ...
##  $ scalar_mean_15mean      : num  17.9 17.9 17.8 17.8 17.6 ...
##  $ changescalar_max_15mean : num  8.2 8.2 6.27 6.27 5.29 ...
##  $ changescalar_mean_15mean: num  4.21 4.21 2.42 2.42 2.06 ...
##  $ Changevector_max_15mean : num  10.15 10.15 8.77 8.77 8.64 ...
##  $ Changevector_mean_15mean: num  6.19 6.19 4.49 4.49 4.18 ...
##  $ Angle_max_15mean        : num  0.473 0.473 0.458 0.458 0.444 ...
##  $ Angle_mean_15mean       : num  0.264 0.264 0.204 0.204 0.196 ...
##  $ Hz_max_15mean           : num  121 121 117 117 113 ...
##  $ Hz_mean_15mean          : num  67.3 67.3 52 52 49.9 ...
##  $ Events_20mean           : int  57 57 75 75 42 42 97 97 71 71 ...
##  $ Duration.ms._20mean     : num  2.59 2.59 5.25 5.25 6 ...
##  $ scalar_max_20mean       : num  22.8 22.8 23.7 23.7 24.3 ...
##  $ scalar_mean_20mean      : num  22 22 22.3 22.3 22.6 ...
##  $ changescalar_max_20mean : num  7.5 7.5 6.19 6.19 6 ...
##  $ changescalar_mean_20mean: num  3.96 3.96 2.68 2.68 2.45 ...
##  $ Changevector_max_20mean : num  11.4 11.4 11.4 11.4 12 ...
##   [list output truncated]
```

```
summary(fm29)
```

```
## Generalized linear mixed model fit by maximum likelihood (Laplace
##   Approximation) [glmerMod]
##  Family: binomial  ( logit )
```

```
## Formula: cbind(emerged, unemerged) ~ Irradiation + changescalar_mean_15max
+
##     Changevector_mean_15max + (1 | Replicate)
##    Data: tab
##
##      AIC      BIC   logLik deviance df.resid
##    175.4    180.9    -82.7    165.4       17
##
## Scaled residuals:
##      Min       1Q   Median       3Q      Max
## -2.18179 -0.47932  0.07369  0.79921  2.05772
##
## Random effects:
##  Groups    Name        Variance Std.Dev.
##  Replicate (Intercept) 0.1613   0.4016
## Number of obs: 22, groups:  Replicate, 6
##
## Fixed effects:
##                         Estimate Std. Error z value Pr(>|z|)
## (Intercept)              2.05351    0.22312   9.203  < 2e-16 ***
## Irradiationyes          -0.18444    0.06835  -2.698  0.00697 **
## changescalar_mean_15max  0.24576    0.03103   7.921 2.36e-15 ***
## Changevector_mean_15max -0.26529    0.02850  -9.307  < 2e-16 ***
## ---
## Signif. codes:  0 '***' 0.001 '**' 0.01 '*' 0.05 '.' 0.1 ' ' 1
##
## Correlation of Fixed Effects:
##             (Intr) Irrdtn ch__15
## Irradiatnys -0.170
## chngscl__15  0.167  0.009
## Chngvct__15 -0.305 -0.001 -0.973

plot((emerged / unemerged) ~ fitted(fm29), data = tab)
abline(lm((emerged / unemerged) ~ fitted(fm29), data = tab), col = "red")
```

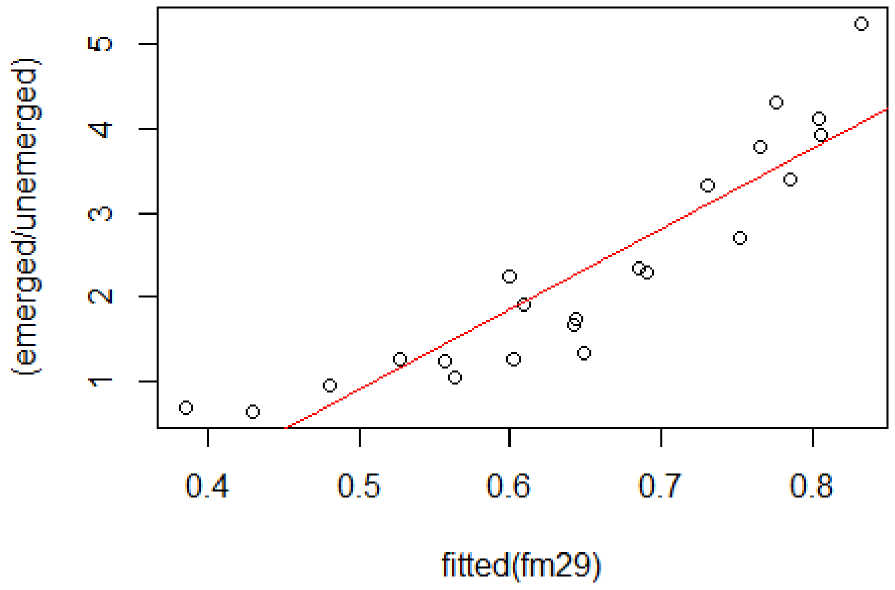


```
cor.test(tab$emerged/tab$unemerged,fitted(fm29))

##
##  Pearson's product-moment correlation
##
## data:  tab$emerged/tab$unemerged and fitted(fm29)
## t = 9.7903, df = 20, p-value = 4.513e-09
## alternative hypothesis: true correlation is not equal to 0
## 95 percent confidence interval:
##  0.7915327 0.9622044
## sample estimates:
##       cor
## 0.9095942

summary(lm(fitted(fm29)~(tab$emerged/tab$unemerged)))$r.squared

## [1] 0.8620339

#####Correlation of the factors in the best model(fm29)
cor.test(tab$changescalar_mean_15max,tab$Changevector_mean_15max)

##
##  Pearson's product-moment correlation
##
## data:  tab$changescalar_mean_15max and tab$Changevector_mean_15max
## t = 23.683, df = 20, p-value = 4.192e-16
## alternative hypothesis: true correlation is not equal to 0
## 95 percent confidence interval:
```

```
##  0.9578497 0.9928978
## sample estimates:
##       cor
## 0.9826338
```

####Conclusion: significantly correlated. Settled on Changevector_mean_15max

MODELS ON THE IMPACT OF SHOCK ON THE flight propensity OF 22 AND 29 DAY OLD PUPAE (COMBINED DATA)

Figure 9b:Flight propensity

```
tab <- read.csv("Figure 9b.csv")
head(tab)
```

```
##     Date Replicate Age     Treatments out in. shipped Irradiation
Events._5max
## 1 170510        R1  22 Shipped-110Gy   23   6     yes         yes
909
## 2 170510        R1  22   Shipped-0Gy   24   7     yes          no
909
## 3 170517        R2  22 Shipped-110Gy    2   1     yes         yes
708
## 4 170517        R2  22   Shipped-0Gy    5   1     yes          no
708
## 5 170524        R3  22 Shipped-110Gy   75   4     yes         yes
1187
## 6 170524        R3  22   Shipped-0Gy   84   5     yes          no
1187
##   Duration.ms._5max scalar_max_5max scalar_mean_5max changescalar_max_5max
## 1            48.750          27.374         21.43900                23.724
## 2            48.750          27.374         21.43900                23.724
## 3            87.500          27.398         16.92723                24.477
## 4            87.500          27.398         16.92723                24.477
## 5            93.125          27.374         15.43705                16.627
## 6            93.125          27.374         15.43705                16.627
##   changescalar_mean_5max Changevector_max_5max Changevector_mean_5max
## 1                8.94875              27.69156               9.937386
## 2                8.94875              27.69156               9.937386
## 3               11.37550              35.06987              19.035967
## 4               11.37550              35.06987              19.035967
## 5                3.83800              33.00443               5.064434
## 6                3.83800              33.00443               5.064434
##   Angle_max_5max Angle_mean_5max Hz_max_5max Hz_mean_5max Events_10max
## 1       3.002116        2.617132    764.4826     666.4471          288
## 2       3.002116        2.617132    764.4826     666.4471          288
## 3       2.969360        1.679485    756.1414     427.6775          315
## 4       2.969360        1.679485    756.1414     427.6775          315
## 5       2.105922        1.356174    536.2686     345.3468          238
## 6       2.105922        1.356174    536.2686     345.3468          238
##   Duration.ms._10max scalar_max_10max scalar_mean_10max
```

```
changescalar_max_10max
## 1             36.875           27.374           21.5800
23.724
## 2             36.875           27.374           21.5800
23.724
## 3             50.000           27.398           20.3750
24.477
## 4             50.000           27.398           20.3750
24.477
## 5             63.750           27.374           19.6895
16.627
## 6             63.750           27.374           19.6895
16.627
##   changescalar_mean_10max Changevector_max_10max Changevector_mean_10max
## 1                  12.473               27.69156                15.45774
## 2                  12.473               27.69156                15.45774
## 3                  16.488               35.06987                22.58486
## 4                  16.488               35.06987                22.58486
## 5                   8.965               33.00443                10.65686
## 6                   8.965               33.00443                10.65686
##   Angle_max_10max Angle_mean_10max Hz_max_10max Hz_mean_10max Events_15max
## 1        2.687392        1.0470779     684.3388      266.6362          144
## 2        2.687392        1.0470779     684.3388      266.6362          144
## 3        2.969360        1.6138687     756.1414      410.9683          183
## 4        2.969360        1.6138687     756.1414      410.9683          183
## 5        2.105922        0.5851573     536.2686      149.0091          111
## 6        2.105922        0.5851573     536.2686      149.0091          111
##   Duration.ms._15max scalar_max_15max scalar_mean_15max
changescalar_max_15max
## 1             30.625           27.374          22.42986
23.724
## 2             30.625           27.374          22.42986
23.724
## 3             29.375           27.398          21.71558
24.477
## 4             29.375           27.398          21.71558
24.477
## 5             41.250           27.374          22.77170
16.627
## 6             41.250           27.374          22.77170
16.627
##   changescalar_mean_15max Changevector_max_15max Changevector_mean_15max
## 1                  15.667               27.69156                19.75723
## 2                  15.667               27.69156                19.75723
## 3                  16.488               35.06987                21.61531
## 4                  16.488               35.06987                21.61531
## 5                  11.474               33.00443                12.83840
## 6                  11.474               33.00443                12.83840
##   Angle_max_15max Angle_mean_15max Hz_max_15max Hz_mean_15max Events_20max
## 1        2.687392        1.8427590     684.3388      469.2547           57
```

```
## 2       2.687392       1.8427590     684.3388      469.2547            57
## 3       2.969360       1.6010884     756.1414      407.7138            75
## 4       2.969360       1.6010884     756.1414      407.7138            75
## 5       2.105922       0.6709018     536.2686      170.8438            42
## 6       2.105922       0.6709018     536.2686      170.8438            42
##   Duration.ms._20max scalar_max_20max scalar_mean_20max
changescalar_max_20max
## 1             17.500           27.374          26.80175
23.724
## 2             17.500           27.374          26.80175
23.724
## 3             20.625           27.398          26.38567
24.477
## 4             20.625           27.398          26.38567
24.477
## 5             20.625           27.374          25.17900
13.480
## 6             20.625           27.374          25.17900
13.480
##   changescalar_mean_20max Changevector_max_20max Changevector_mean_20max
## 1                 23.7240               27.69156                27.69156
## 2                 23.7240               27.69156                27.69156
## 3                 13.7095               35.06987                29.65156
## 4                 13.7095               35.06987                29.65156
## 5                  6.6090               33.00443                11.19072
## 6                  6.6090               33.00443                11.19072
##   Angle_max_20max Angle_mean_20max Hz_max_20max Hz_mean_20max Events_5mean
## 1        2.687392         2.687392     684.3388      684.3388          909
## 2        2.687392         2.687392     684.3388      684.3388          909
## 3        2.433775         1.792941     619.7557      456.5688          708
## 4        2.433775         1.792941     619.7557      456.5688          708
## 5        1.715913         0.489164     436.9538      124.5646         1187
## 6        1.715913         0.489164     436.9538      124.5646         1187
##   Duration.ms._5mean scalar_max_5mean scalar_mean_5mean
changescalar_max_5mean
## 1           5.215209         9.516648          7.473747
3.755884
## 2           5.215209         9.516648          7.473747
3.755884
## 3          11.785840        10.551797          7.565272
2.816095
## 4          11.785840        10.551797          7.565272
2.816095
## 5           7.348884         8.022198          6.506753
1.632164
## 6           7.348884         8.022198          6.506753
1.632164
##   changescalar_mean_5mean Changevector_max_5mean Changevector_mean_5mean
## 1               1.6630629               4.501180                2.219564
## 2               1.6630629               4.501180                2.219564
```

```
## 3              1.0076953              3.691769               1.638802
## 4              1.0076953              3.691769               1.638802
## 5              0.6804169              2.293485               1.194539
## 6              0.6804169              2.293485               1.194539
##   Angle_max_5mean Angle_mean_5mean Hz_max_5mean Hz_mean_5mean
Events._10mean
## 1       0.4296757        0.1755708    109.41603      44.70874
288
## 2       0.4296757        0.1755708    109.41603      44.70874
288
## 3       0.3715939        0.1414327     94.62562      36.01553
315
## 4       0.3715939        0.1414327     94.62562      36.01553
315
## 5       0.2539263        0.1310499     64.66179      33.37158
238
## 6       0.2539263        0.1310499     64.66179      33.37158
238
##   Duration.ms._10mean scalar_max_10mean scalar_mean_10mean
## 1            3.888889          15.49537           13.49979
## 2            3.888889          15.49537           13.49979
## 3            7.706349          16.38244           13.65682
## 4            7.706349          16.38244           13.65682
## 5            7.415966          15.56483           13.15894
## 6            7.415966          15.56483           13.15894
##   changescalar_max_10mean changescalar_mean_10mean Changevector_max_10mean
## 1                6.557340                 3.249119                8.029862
## 2                6.557340                 3.249119                8.029862
## 3                4.928854                 1.971893                6.505797
## 4                4.928854                 1.971893                6.505797
## 5                4.004025                 1.664277                5.891075
## 6                4.004025                 1.664277                5.891075
##   Changevector_mean_10mean Angle_max_10mean Angle_mean_10mean
Hz_max_10mean
## 1                 4.538349        0.4719538         0.2224823
120.1821
## 2                 4.538349        0.4719538         0.2224823
120.1821
## 3                 3.339135        0.4047630         0.1751144
103.0721
## 4                 3.339135        0.4047630         0.1751144
103.0721
## 5                 2.909783        0.3492638         0.1583697
88.9393
## 6                 2.909783        0.3492638         0.1583697
88.9393
##   Hz_mean_10mean Events_15mean Duration.ms._15mean scalar_max_15mean
## 1       56.65466           144            3.441840          19.26462
## 2       56.65466           144            3.441840          19.26462
## 3       44.59251           183            6.342213          19.93954
```

```
## 4       44.59251          183             6.342213          19.93954
## 5       40.32851          111             6.920045          19.71768
## 6       40.32851          111             6.920045          19.71768
##   scalar_mean_15mean changescalar_max_15mean changescalar_mean_15mean
## 1           17.94158                8.195410                 4.209463
## 2           17.94158                8.195410                 4.209463
## 3           17.79343                6.271208                 2.424063
## 4           17.79343                6.271208                 2.424063
## 5           17.60073                5.291784                 2.055466
## 6           17.60073                5.291784                 2.055466
##   Changevector_max_15mean Changevector_mean_15mean Angle_max_15mean
## 1               10.145769                 6.194357        0.4732660
## 2               10.145769                 6.194357        0.4732660
## 3                8.773143                 4.485467        0.4584332
## 4                8.773143                 4.485467        0.4584332
## 5                8.644509                 4.178605        0.4437400
## 6                8.644509                 4.178605        0.4437400
##   Angle_mean_15mean Hz_max_15mean Hz_mean_15mean Events_20mean
## 1         0.2644405      120.5162       67.33922            57
## 2         0.2644405      120.5162       67.33922            57
## 3         0.2042285      116.7390       52.00635            75
## 4         0.2042285      116.7390       52.00635            75
## 5         0.1960600      112.9975       49.92626            42
## 6         0.1960600      112.9975       49.92626            42
##   Duration.ms._20mean scalar_max_20mean scalar_mean_20mean
## 1            2.587719          22.76440           22.04857
## 2            2.587719          22.76440           22.04857
## 3            5.250000          23.71752           22.29175
## 4            5.250000          23.71752           22.29175
## 5            5.997024          24.28693           22.58697
## 6            5.997024          24.28693           22.58697
##   changescalar_max_20mean changescalar_mean_20mean Changevector_max_20mean
## 1                7.502070                 3.960703                11.35123
## 2                7.502070                 3.960703                11.35123
## 3                6.190293                 2.676377                11.36833
## 4                6.190293                 2.676377                11.36833
## 5                5.996190                 2.445063                11.97291
## 6                5.996190                 2.445063                11.97291
##   Changevector_mean_20mean Angle_max_20mean Angle_mean_20mean
Hz_max_20mean
## 1                 6.922532        0.5201012         0.3140733
132.4427
## 2                 6.922532        0.5201012         0.3140733
132.4427
## 3                 5.877764        0.5214004         0.2548023
132.7735
## 4                 5.877764        0.5214004         0.2548023
132.7735
## 5                 5.476046        0.5391966         0.2201107
137.3053
```

```
## 6                 5.476046        0.5391966         0.2201107
137.3053
##   Hz_mean_20mean RH._Max Temp_Max RH._mean Temp_mean
## 1       79.97812   47.08    23.50    41.69     19.73
## 2       79.97812   60.14    27.50    41.69     19.73
## 3       64.88487   62.61    29.75    53.55     21.79
## 4       64.88487   68.10    32.00    53.55     21.79
## 5       56.05073   57.37    28.69    53.25     23.29
## 6       56.05073   74.59    19.25    53.25     23.29
```

```
str(tab)
```

```
## 'data.frame':    22 obs. of  108 variables:
##  $ Date                    : int  170510 170510 170517 170517 170524
170524 170531 170531 170607 170607 ...
##  $ Replicate               : chr  "R1" "R1" "R2" "R2" ...
##  $ Age                     : int  22 22 22 22 22 22 22 22 22 22 ...
##  $ Treatments              : chr  "Shipped-110Gy " "Shipped-0Gy "
"Shipped-110Gy " "Shipped-0Gy " ...
##  $ out                     : int  23 24 2 5 75 84 73 75 76 95 ...
##  $ in.                     : int  6 7 1 1 4 5 7 0 4 0 ...
##  $ shipped                 : chr  "yes" "yes" "yes" "yes" ...
##  $ Irradiation             : chr  "yes" "no" "yes" "no" ...
##  $ Events._5max            : int  909 909 708 708 1187 1187 625 625 606
606 ...
##  $ Duration.ms._5max       : num  48.8 48.8 87.5 87.5 93.1 ...
##  $ scalar_max_5max         : num  27.4 27.4 27.4 27.4 27.4 ...
##  $ scalar_mean_5max        : num  21.4 21.4 16.9 16.9 15.4 ...
##  $ changescalar_max_5max   : num  23.7 23.7 24.5 24.5 16.6 ...
##  $ changescalar_mean_5max  : num  8.95 8.95 11.38 11.38 3.84 ...
##  $ Changevector_max_5max   : num  27.7 27.7 35.1 35.1 33 ...
##  $ Changevector_mean_5max  : num  9.94 9.94 19.04 19.04 5.06 ...
##  $ Angle_max_5max          : num  3 3 2.97 2.97 2.11 ...
##  $ Angle_mean_5max         : num  2.62 2.62 1.68 1.68 1.36 ...
##  $ Hz_max_5max             : num  764 764 756 756 536 ...
##  $ Hz_mean_5max            : num  666 666 428 428 345 ...
##  $ Events_10max            : int  288 288 315 315 238 238 261 261 219 219
...
##  $ Duration.ms._10max      : num  36.9 36.9 50 50 63.8 ...
##  $ scalar_max_10max        : num  27.4 27.4 27.4 27.4 27.4 ...
##  $ scalar_mean_10max       : num  21.6 21.6 20.4 20.4 19.7 ...
##  $ changescalar_max_10max  : num  23.7 23.7 24.5 24.5 16.6 ...
##  $ changescalar_mean_10max : num  12.47 12.47 16.49 16.49 8.97 ...
##  $ Changevector_max_10max  : num  27.7 27.7 35.1 35.1 33 ...
##  $ Changevector_mean_10max : num  15.5 15.5 22.6 22.6 10.7 ...
##  $ Angle_max_10max         : num  2.69 2.69 2.97 2.97 2.11 ...
##  $ Angle_mean_10max        : num  1.047 1.047 1.614 1.614 0.585 ...
##  $ Hz_max_10max            : num  684 684 756 756 536 ...
##  $ Hz_mean_10max           : num  267 267 411 411 149 ...
##  $ Events_15max            : int  144 144 183 183 111 111 163 163 129 129
```

```
...
##  $ Duration.ms._15max      : num  30.6 30.6 29.4 29.4 41.2 ...
##  $ scalar_max_15max        : num  27.4 27.4 27.4 27.4 27.4 ...
##  $ scalar_mean_15max       : num  22.4 22.4 21.7 21.7 22.8 ...
##  $ changescalar_max_15max  : num  23.7 23.7 24.5 24.5 16.6 ...
##  $ changescalar_mean_15max : num  15.7 15.7 16.5 16.5 11.5 ...
##  $ Changevector_max_15max  : num  27.7 27.7 35.1 35.1 33 ...
##  $ Changevector_mean_15max : num  19.8 19.8 21.6 21.6 12.8 ...
##  $ Angle_max_15max         : num  2.69 2.69 2.97 2.97 2.11 ...
##  $ Angle_mean_15max        : num  1.843 1.843 1.601 1.601 0.671 ...
##  $ Hz_max_15max            : num  684 684 756 756 536 ...
##  $ Hz_mean_15max           : num  469 469 408 408 171 ...
##  $ Events_20max            : int  57 57 75 75 42 42 97 97 71 71 ...
##  $ Duration.ms._20max      : num  17.5 17.5 20.6 20.6 20.6 ...
##  $ scalar_max_20max        : num  27.4 27.4 27.4 27.4 27.4 ...
##  $ scalar_mean_20max       : num  26.8 26.8 26.4 26.4 25.2 ...
##  $ changescalar_max_20max  : num  23.7 23.7 24.5 24.5 13.5 ...
##  $ changescalar_mean_20max : num  23.72 23.72 13.71 13.71 6.61 ...
##  $ Changevector_max_20max  : num  27.7 27.7 35.1 35.1 33 ...
##  $ Changevector_mean_20max : num  27.7 27.7 29.7 29.7 11.2 ...
##  $ Angle_max_20max         : num  2.69 2.69 2.43 2.43 1.72 ...
##  $ Angle_mean_20max        : num  2.687 2.687 1.793 1.793 0.489 ...
##  $ Hz_max_20max            : num  684 684 620 620 437 ...
##  $ Hz_mean_20max           : num  684 684 457 457 125 ...
##  $ Events_5mean            : int  909 909 708 708 1187 1187 625 625 606
606 ...
##  $ Duration.ms._5mean      : num  5.22 5.22 11.79 11.79 7.35 ...
##  $ scalar_max_5mean        : num  9.52 9.52 10.55 10.55 8.02 ...
##  $ scalar_mean_5mean       : num  7.47 7.47 7.57 7.57 6.51 ...
##  $ changescalar_max_5mean  : num  3.76 3.76 2.82 2.82 1.63 ...
##  $ changescalar_mean_5mean : num  1.66 1.66 1.01 1.01 0.68 ...
##  $ Changevector_max_5mean  : num  4.5 4.5 3.69 3.69 2.29 ...
##  $ Changevector_mean_5mean : num  2.22 2.22 1.64 1.64 1.19 ...
##  $ Angle_max_5mean         : num  0.43 0.43 0.372 0.372 0.254 ...
##  $ Angle_mean_5mean        : num  0.176 0.176 0.141 0.141 0.131 ...
##  $ Hz_max_5mean            : num  109.4 109.4 94.6 94.6 64.7 ...
##  $ Hz_mean_5mean           : num  44.7 44.7 36 36 33.4 ...
##  $ Events._10mean          : int  288 288 315 315 238 238 261 261 219 219
...
##  $ Duration.ms._10mean     : num  3.89 3.89 7.71 7.71 7.42 ...
##  $ scalar_max_10mean       : num  15.5 15.5 16.4 16.4 15.6 ...
##  $ scalar_mean_10mean      : num  13.5 13.5 13.7 13.7 13.2 ...
##  $ changescalar_max_10mean : num  6.56 6.56 4.93 4.93 4 ...
##  $ changescalar_mean_10mean: num  3.25 3.25 1.97 1.97 1.66 ...
##  $ Changevector_max_10mean : num  8.03 8.03 6.51 6.51 5.89 ...
##  $ Changevector_mean_10mean: num  4.54 4.54 3.34 3.34 2.91 ...
##  $ Angle_max_10mean        : num  0.472 0.472 0.405 0.405 0.349 ...
##  $ Angle_mean_10mean       : num  0.222 0.222 0.175 0.175 0.158 ...
##  $ Hz_max_10mean           : num  120.2 120.2 103.1 103.1 88.9 ...
##  $ Hz_mean_10mean          : num  56.7 56.7 44.6 44.6 40.3 ...
```

```
##  $ Events_15mean           : int  144 144 183 183 111 111 163 163 129 129
...
##  $ Duration.ms._15mean     : num  3.44 3.44 6.34 6.34 6.92 ...
##  $ scalar_max_15mean       : num  19.3 19.3 19.9 19.9 19.7 ...
##  $ scalar_mean_15mean      : num  17.9 17.9 17.8 17.8 17.6 ...
##  $ changescalar_max_15mean : num  8.2 8.2 6.27 6.27 5.29 ...
##  $ changescalar_mean_15mean: num  4.21 4.21 2.42 2.42 2.06 ...
##  $ Changevector_max_15mean : num  10.15 10.15 8.77 8.77 8.64 ...
##  $ Changevector_mean_15mean: num  6.19 6.19 4.49 4.49 4.18 ...
##  $ Angle_max_15mean        : num  0.473 0.473 0.458 0.458 0.444 ...
##  $ Angle_mean_15mean       : num  0.264 0.264 0.204 0.204 0.196 ...
##  $ Hz_max_15mean           : num  121 121 117 117 113 ...
##  $ Hz_mean_15mean          : num  67.3 67.3 52 52 49.9 ...
##  $ Events_20mean           : int  57 57 75 75 42 42 97 97 71 71 ...
##  $ Duration.ms._20mean     : num  2.59 2.59 5.25 5.25 6 ...
##  $ scalar_max_20mean       : num  22.8 22.8 23.7 23.7 24.3 ...
##  $ scalar_mean_20mean      : num  22 22 22.3 22.3 22.6 ...
##  $ changescalar_max_20mean : num  7.5 7.5 6.19 6.19 6 ...
##  $ changescalar_mean_20mean: num  3.96 3.96 2.68 2.68 2.45 ...
##  $ Changevector_max_20mean : num  11.4 11.4 11.4 11.4 12 ...
##   [list output truncated]

tab$pcflight <- tab$out / (tab$out+tab$in.)
boxplot(tab$pcflight~ tab$Treatments, xlab ="Treatments", ylab = "Flight
propensity")
```

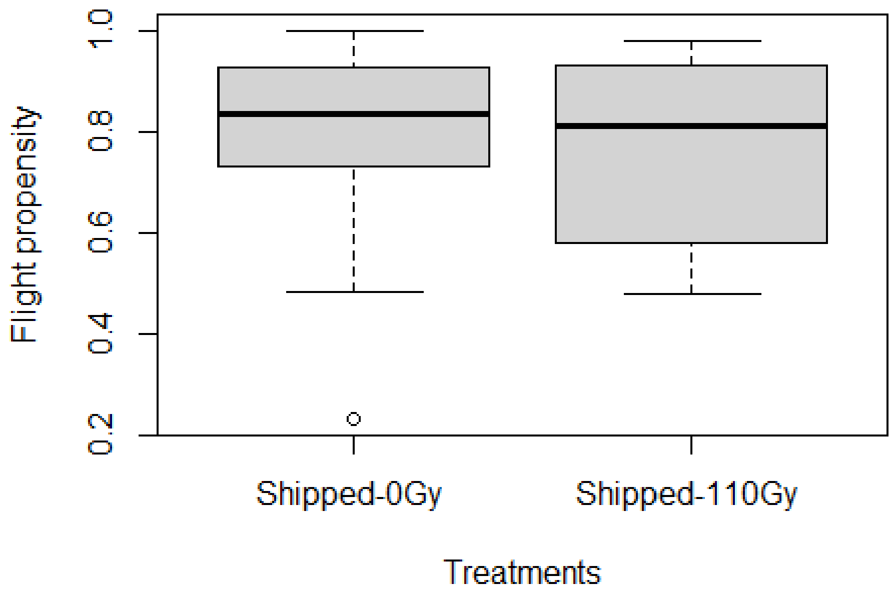


```
plot(tab$pcflight~tab$RH._Max)#*
```

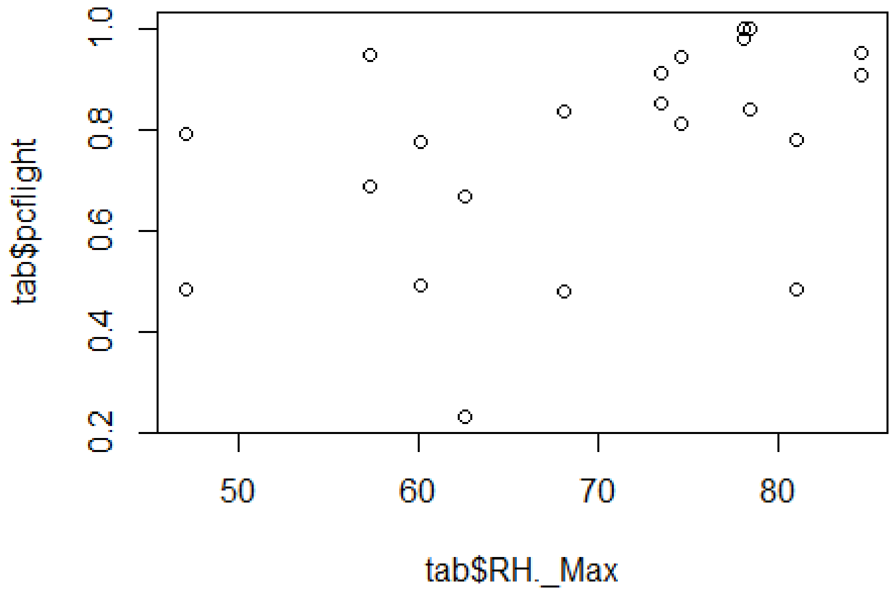


```
plot(tab$pcflight~tab$RH._mean)
```

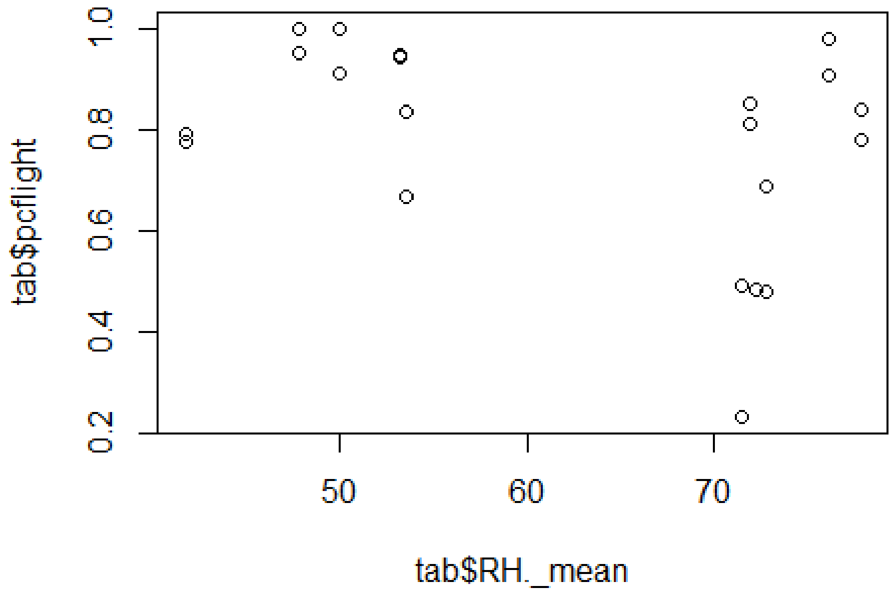


```
plot(tab$pcflight~tab$Temp_mean)#*
```

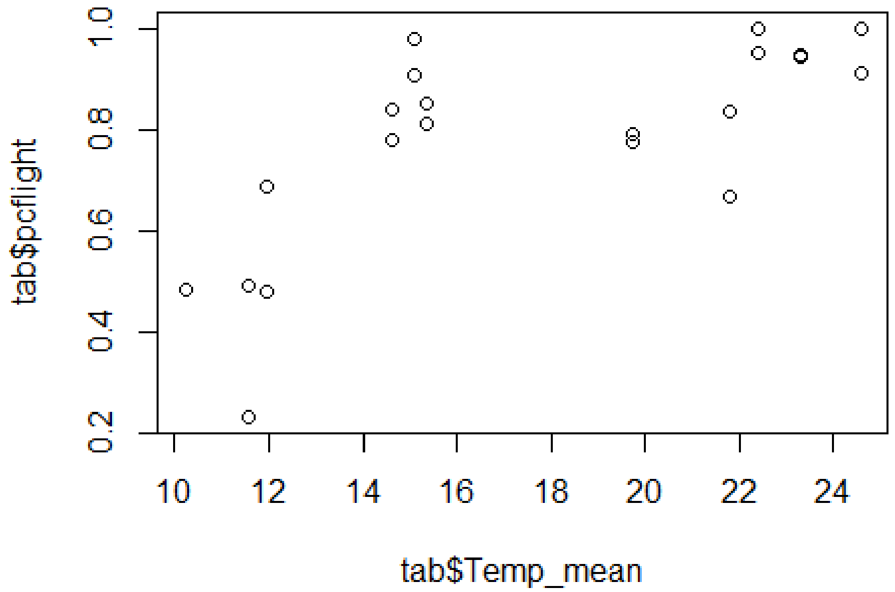


```
cor.test(tab$pcflight,tab$Temp_mean)

##
##  Pearson's product-moment correlation
##
## data:  tab$pcflight and tab$Temp_mean
## t = 4.7462, df = 20, p-value = 0.0001235
## alternative hypothesis: true correlation is not equal to 0
## 95 percent confidence interval:
##  0.4417532 0.8795327
## sample estimates:
##       cor
## 0.7278094

plot(tab$pcflight~tab$Temp_Max)
```

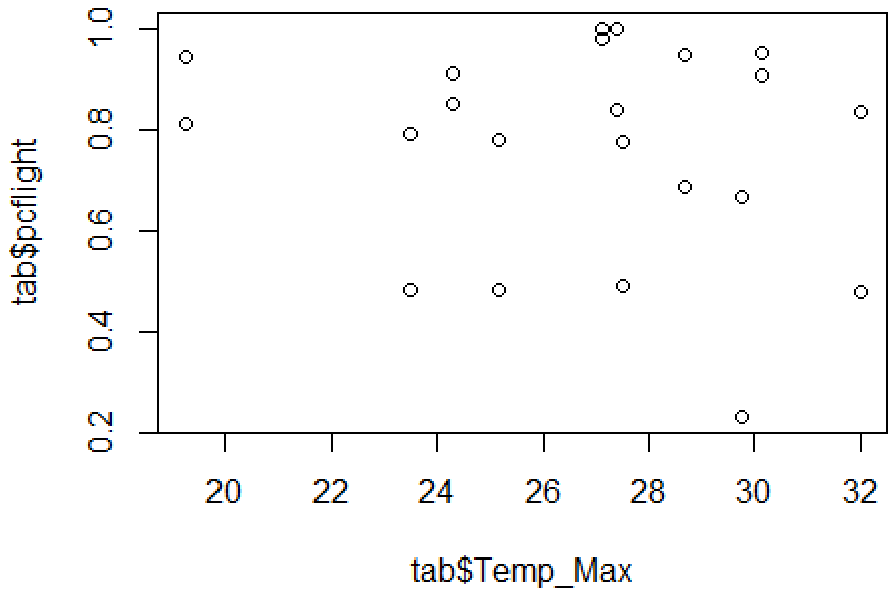


```
par(mfrow = c(2,2))
plot(tab$pcflight~tab$Events._5max)
```

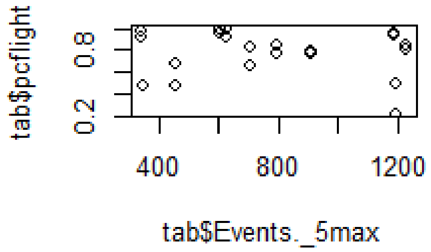

#Scatter plots at threshold 5-Max

```
par(mfrow = c(2,2))
plot(tab$pcflight~tab$Duration.ms._5max)#*
plot(tab$pcflight~tab$scalar_max_5max)
plot(tab$pcflight~tab$scalar_mean_5max)#*
plot(tab$pcflight~tab$changescalar_max_5max)
```

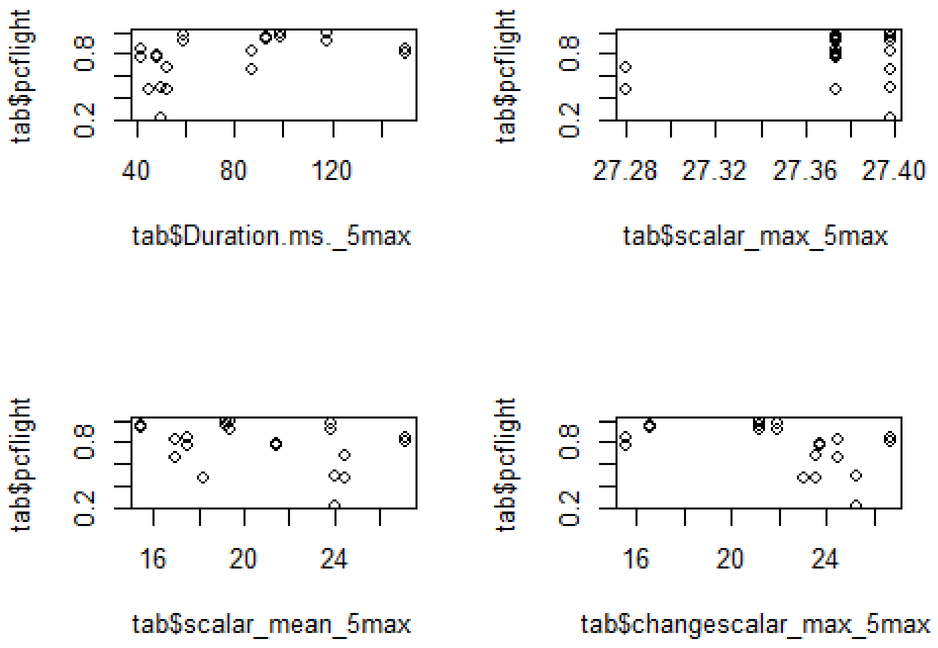


```
plot(tab$pcflight~tab$changescalar_mean_5max)*#
plot(tab$pcflight~tab$Changevector_max_5max)

## integer(0)

plot(tab$pcflight~tab$Changevector_mean_5max)
plot(tab$pcflight~tab$Angle_max_5max)#*
```

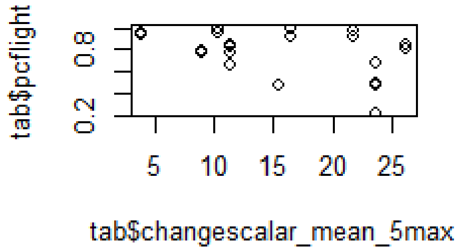

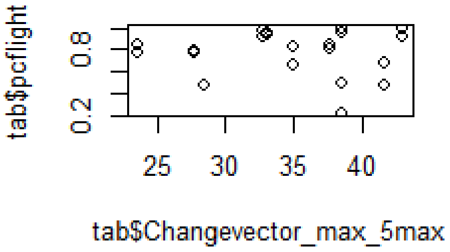

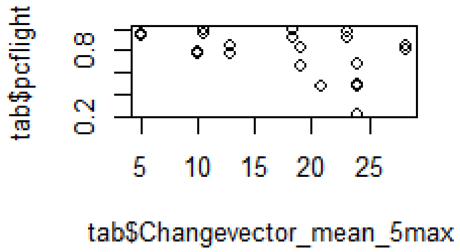

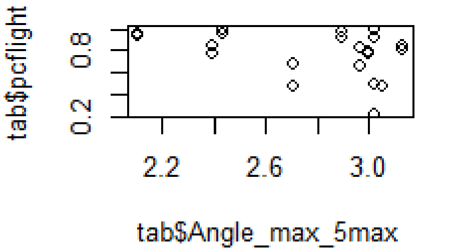


```
plot(tab$pcflight~tab$Angle_mean_5max)
plot(tab$pcflight~tab$Hz_max_5max)#*
plot(tab$pcflight~tab$Hz_mean_5max)
```

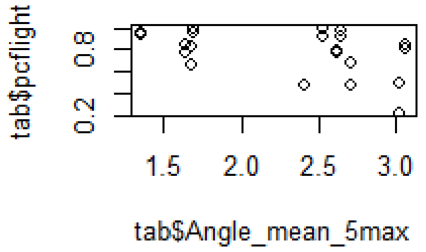

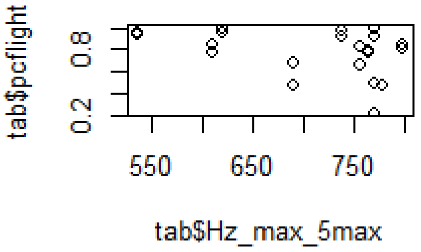

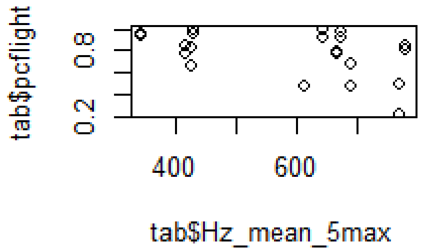

#Scatter plots at threshold 5-Mean

```
par(mfrow = c(2,2))
plot(tab$pcflight~tab$Duration.ms._5mean)#*
plot(tab$pcflight~tab$scalar_max_5mean)
plot(tab$pcflight~tab$scalar_mean_5mean)
plot(tab$pcflight~tab$changescalar_max_5mean)
```

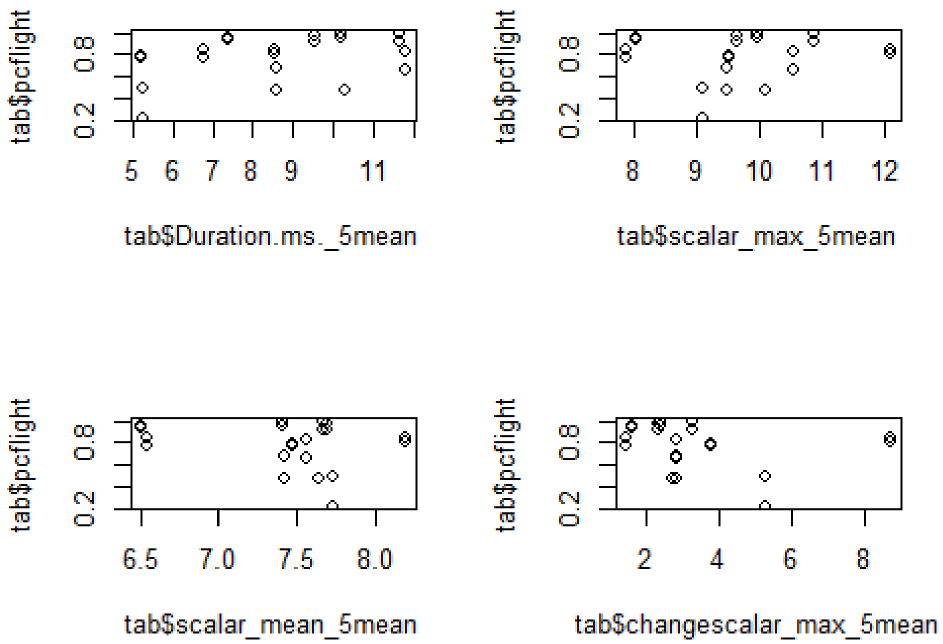


```
plot(tab$pcflight~tab$changescalar_mean_5mean)
plot(tab$pcflight~tab$Changevector_max_5mean)
plot(tab$pcflight~tab$Changevector_mean_5mean)
plot(tab$pcflight~tab$Angle_max_5mean)
```

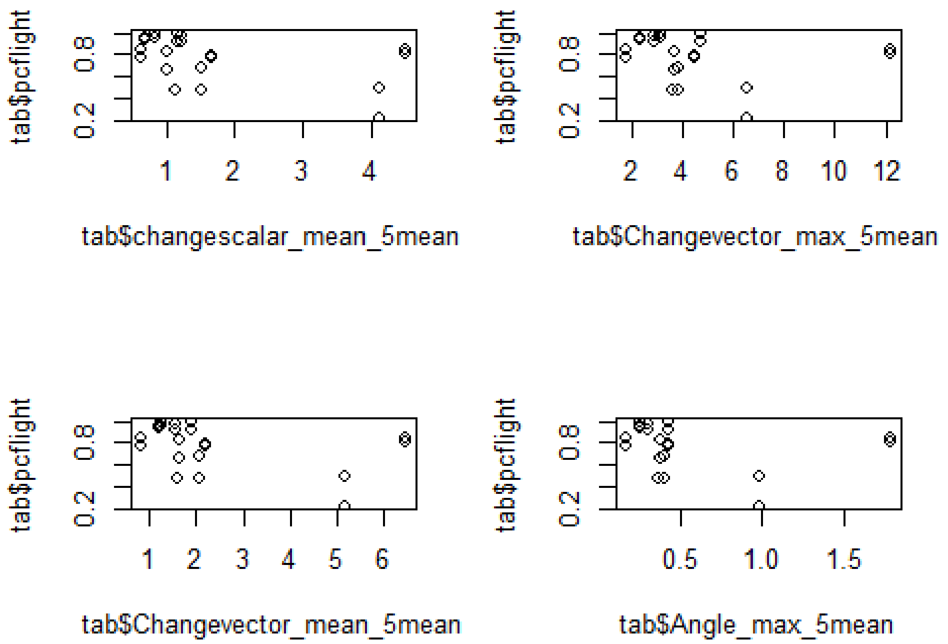


```
plot(tab$pcflight~tab$Angle_mean_5mean)
plot(tab$pcflight~tab$Hz_max_5mean)
plot(tab$pcflight~tab$Hz_mean_5mean)
```

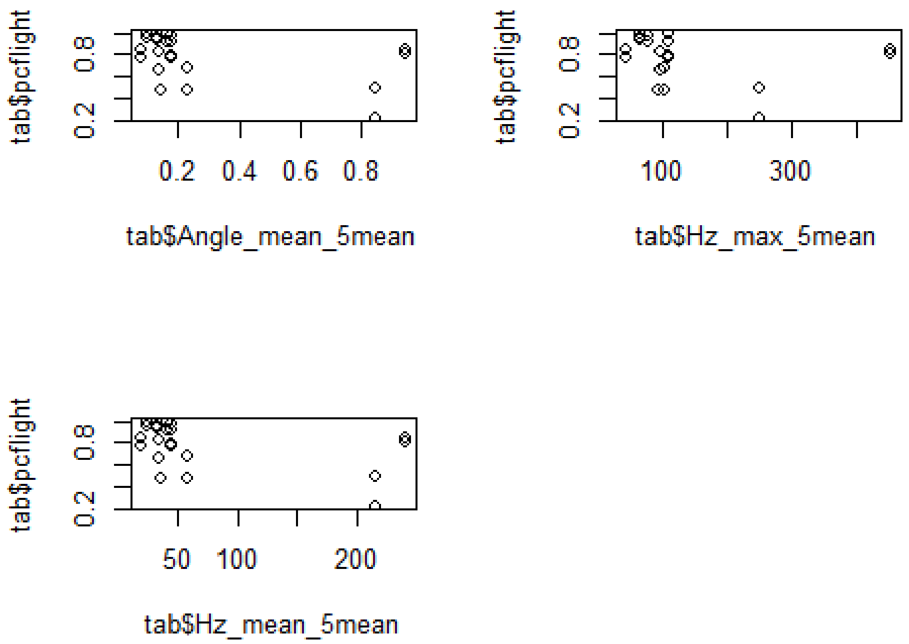

#Scatter plots at threshold 10-Max

```
par(mfrow = c(2,2))
plot(tab$pcflight~tab$Events_10max)
plot(tab$pcflight~tab$Duration.ms._10max)
plot(tab$pcflight~tab$scalar_max_10max)
plot(tab$pcflight~tab$scalar_mean_10max)#*
```

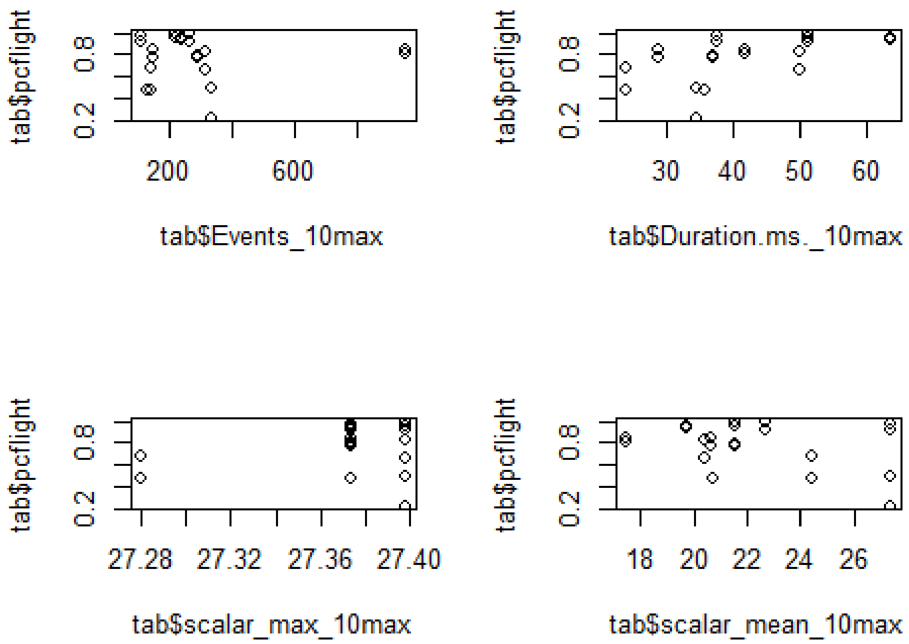


```
plot(tab$pcflight~tab$changescalar_max_10max)
plot(tab$pcflight~tab$changescalar_mean_10max)
plot(tab$pcflight~tab$Changevector_max_10max)
plot(tab$pcflight~tab$Changevector_mean_10max)#*
```

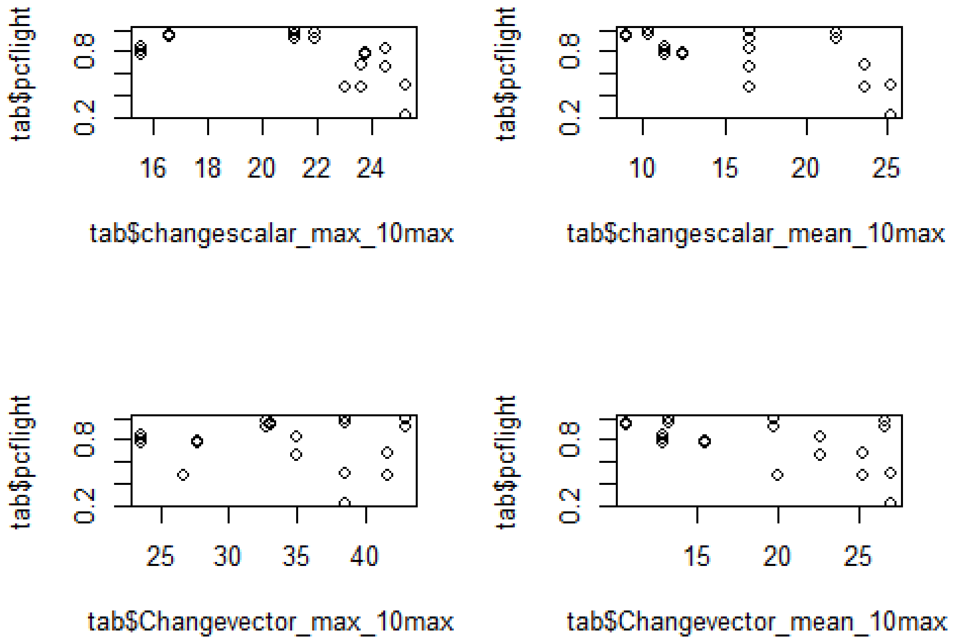


```r
plot(tab$pcflight~tab$Angle_max_10max)
plot(tab$pcflight~tab$Angle_mean_10max)
plot(tab$pcflight~tab$Hz_max_10max)
plot(tab$pcflight~tab$Hz_mean_10max)#*
```

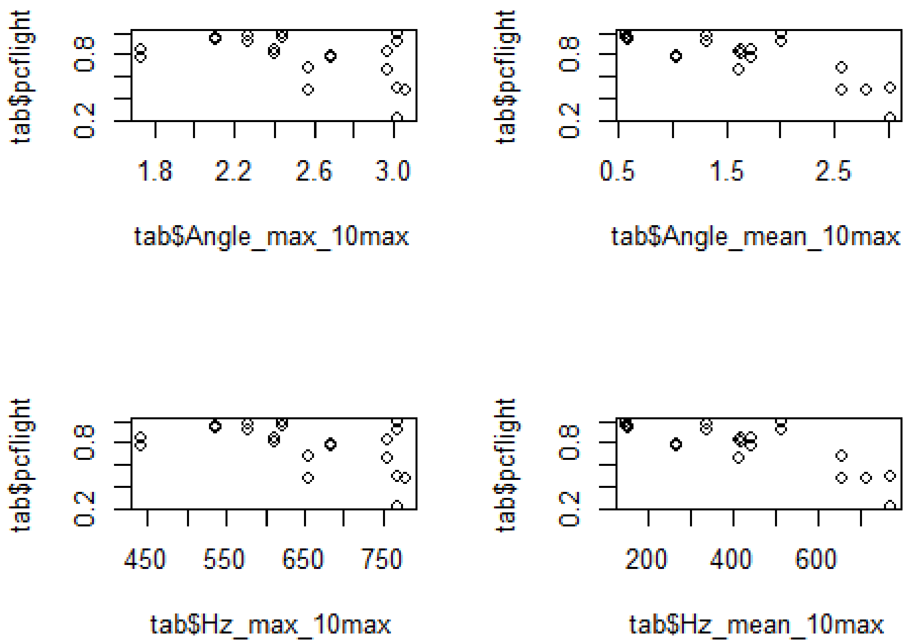

#Scatter plots at threshold 10-Mean

```
par(mfrow = c(2,2))
plot(tab$pcflight~tab$Duration.ms._10mean)#*
plot(tab$pcflight~tab$scalar_max_10mean)#*
plot(tab$pcflight~tab$scalar_mean_10mean)
plot(tab$pcflight~tab$changescalar_max_10mean)
```

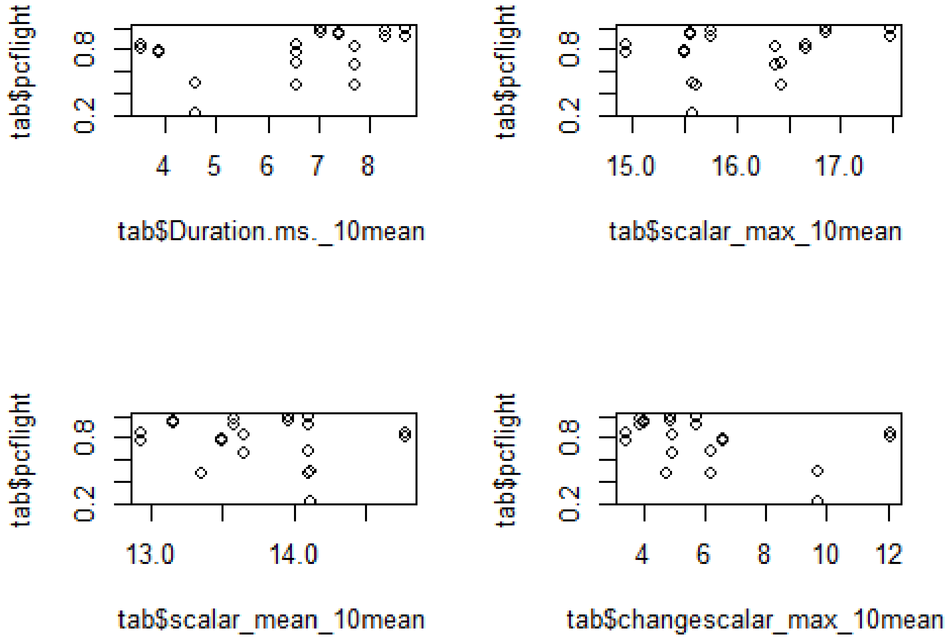


```
plot(tab$pcflight~tab$changescalar_mean_10mean)#*
plot(tab$pcflight~tab$Changevector_max_10mean)
plot(tab$pcflight~tab$Changevector_mean_10mean)#*
plot(tab$pcflight~tab$Angle_max_10mean)
```

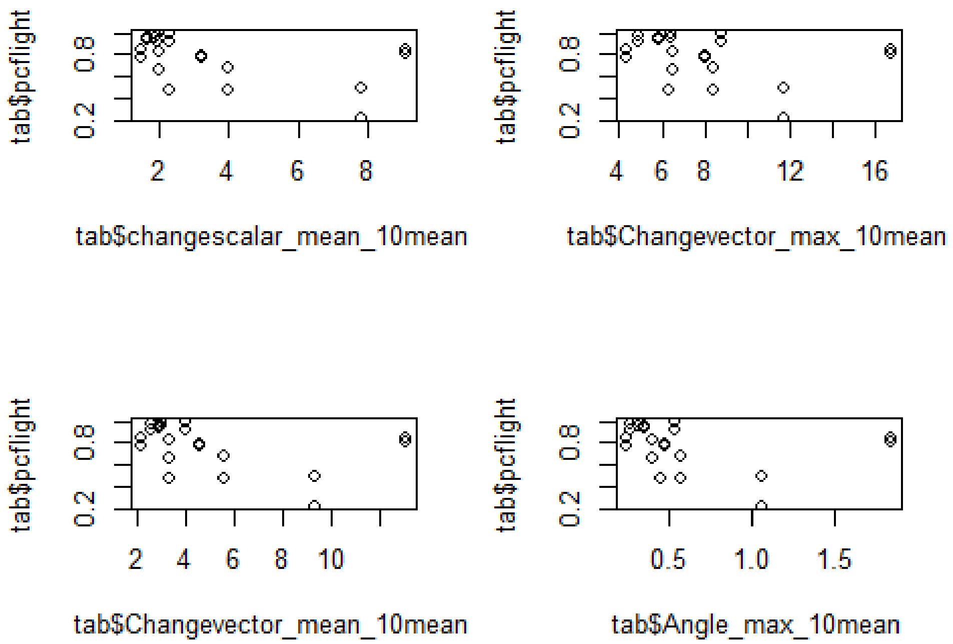


```
plot(tab$pcflight~tab$Angle_mean_10mean)
plot(tab$pcflight~tab$Hz_max_10mean)
plot(tab$pcflight~tab$Hz_mean_10mean)
```

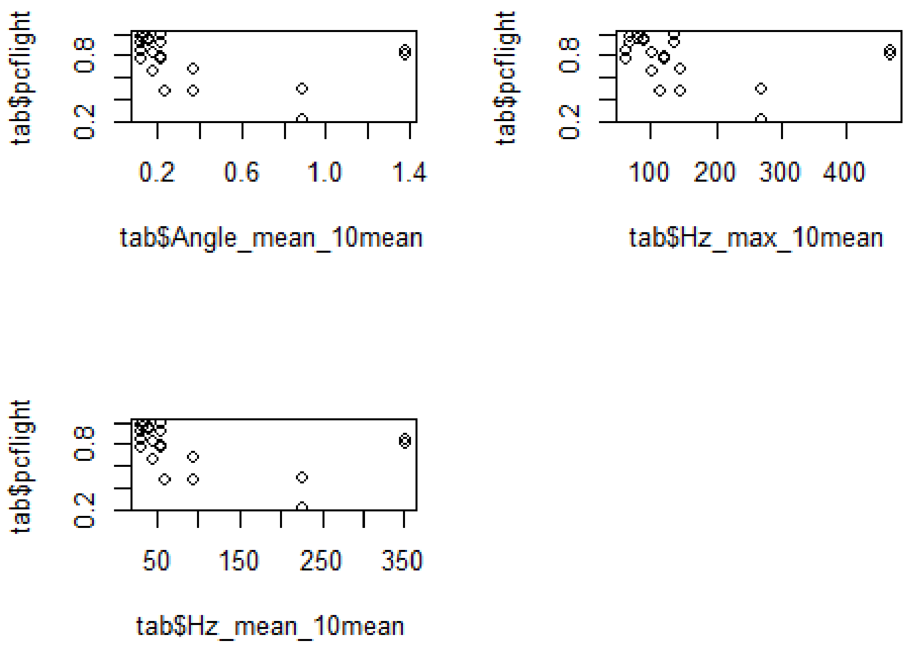

#Scatter plots at threshold 15-Max

```
par(mfrow = c(2,2))
plot(tab$pcflight~tab$Events_15max)
plot(tab$pcflight~tab$Duration.ms._15max)
plot(tab$pcflight~tab$scalar_max_15max)
plot(tab$pcflight~tab$scalar_mean_15max)
```

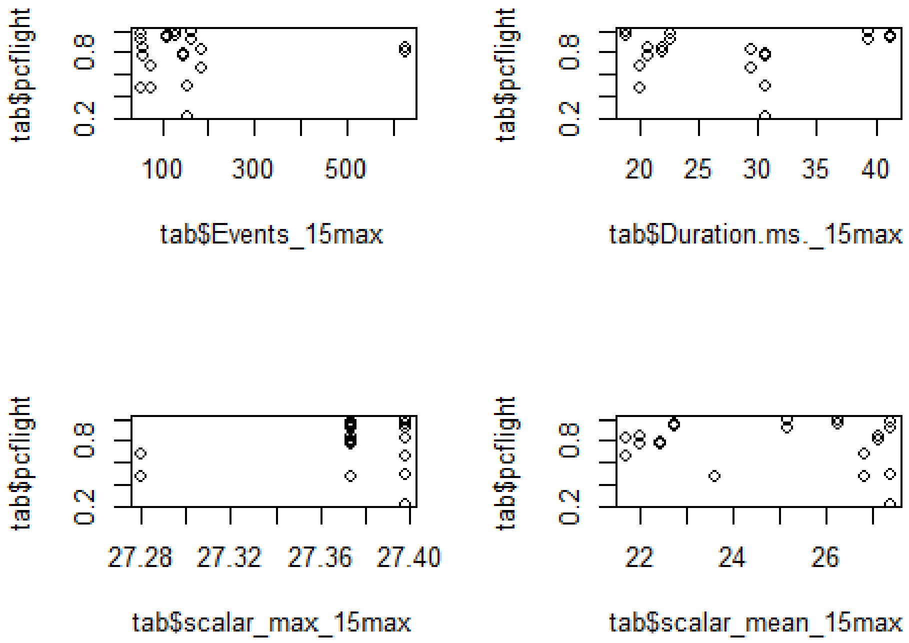


```
plot(tab$pcflight~tab$changescalar_max_15max)
plot(tab$pcflight~tab$changescalar_mean_15max)#*
plot(tab$pcflight~tab$Changevector_max_15max)
plot(tab$pcflight~tab$Changevector_mean_15max)#*
```

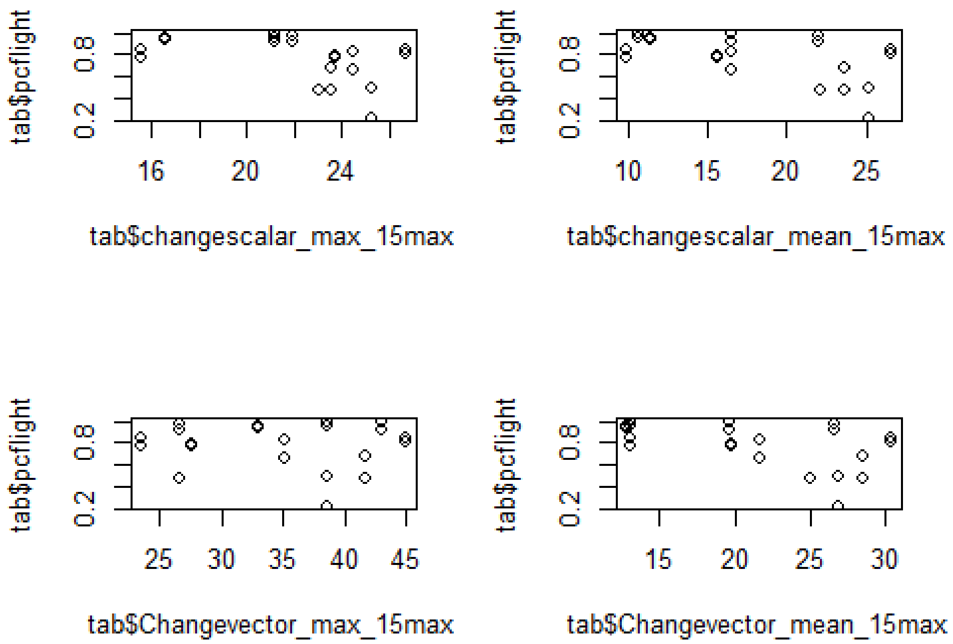


```
plot(tab$pcflight~tab$Angle_max_15max)
plot(tab$pcflight~tab$Angle_mean_15max)#*
cor.test(tab$pcflight,tab$Angle_mean_15max)

##
##  Pearson's product-moment correlation
##
## data:  tab$pcflight and tab$Angle_mean_15max
## t = -3.9286, df = 20, p-value = 0.0008313
## alternative hypothesis: true correlation is not equal to 0
## 95 percent confidence interval:
##  -0.8461441 -0.3302650
## sample estimates:
##        cor
## -0.6599767

plot(tab$pcflight~tab$Hz_max_15max)
plot(tab$pcflight~tab$Hz_mean_15max)#*
```

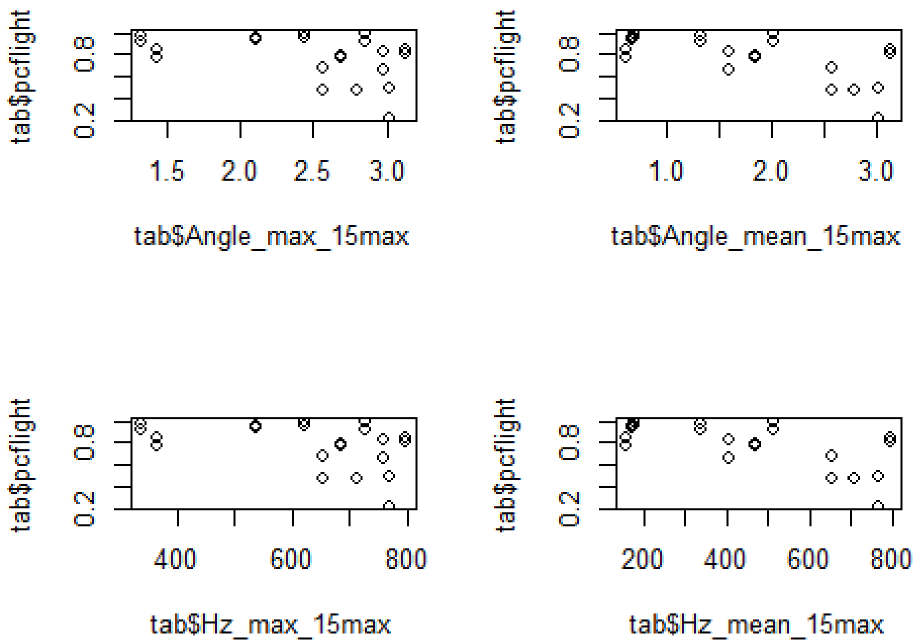

#Scatter plots at threshold 15-Max-mean

```
par(mfrow = c(2,2))

plot(tab$pcflight~tab$Duration.ms._15mean)#*
plot(tab$pcflight~tab$scalar_max_15mean)
plot(tab$pcflight~tab$scalar_mean_15mean)#*
plot(tab$pcflight~tab$changescalar_max_15mean)
```

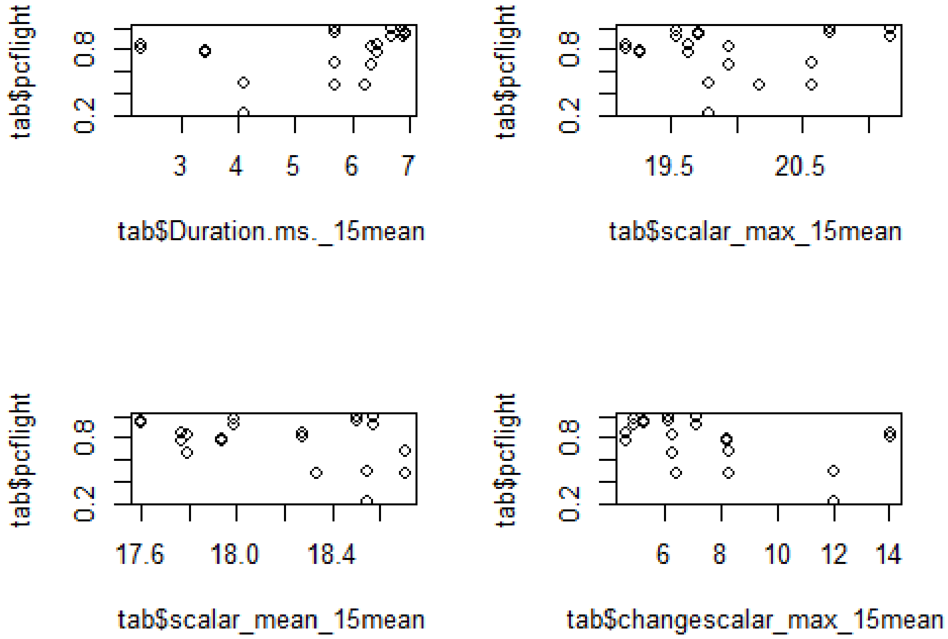


```
plot(tab$pcflight~tab$changescalar_mean_15mean)#*
plot(tab$pcflight~tab$Changevector_max_15mean)
plot(tab$pcflight~tab$Changevector_mean_15mean)#*
plot(tab$pcflight~tab$Angle_max_15mean)
```

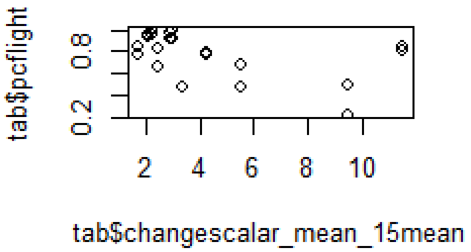

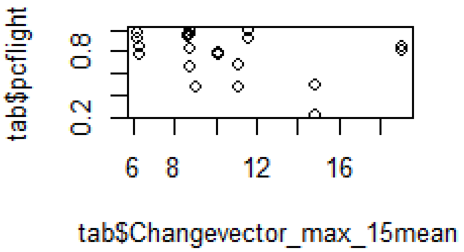

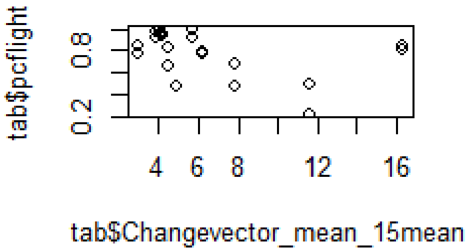

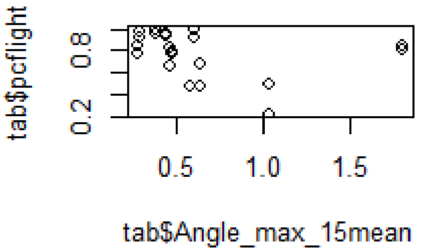


```
plot(tab$pcflight~tab$Angle_mean_15mean)
plot(tab$pcflight~tab$Hz_max_15mean)
plot(tab$pcflight~tab$Hz_mean_15mean)
```

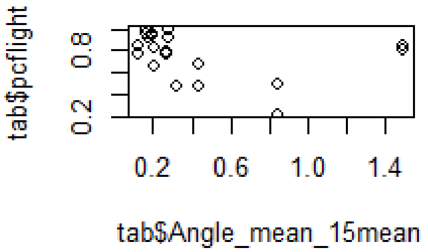

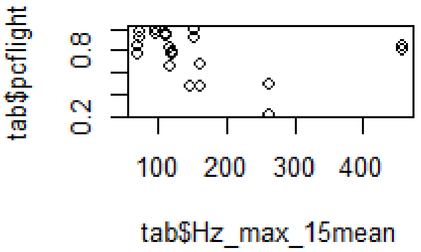

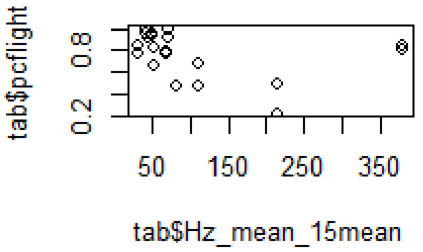

#Scatter plots at threshold 20-Max

```
par(mfrow = c(2,2))

plot(tab$pcflight~tab$Events_20max)
plot(tab$pcflight~tab$Duration.ms._20max)#*
plot(tab$pcflight~tab$scalar_max_20max)
plot(tab$pcflight~tab$scalar_mean_20max)
```

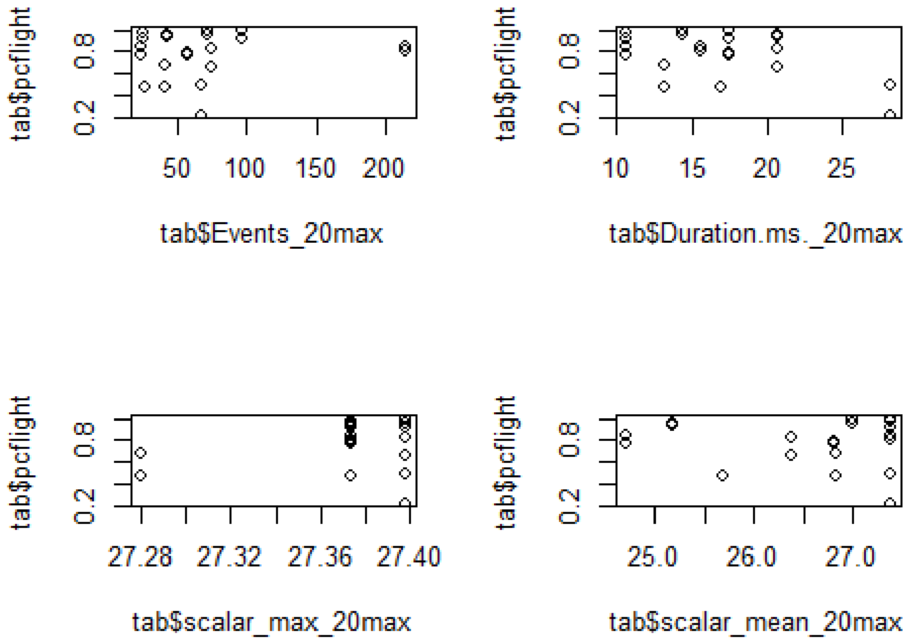


```
plot(tab$pcflight~tab$changescalar_max_20max)#*
plot(tab$pcflight~tab$changescalar_mean_20max)
plot(tab$pcflight~tab$Changevector_max_20max)
plot(tab$pcflight~tab$Changevector_mean_20max)
```

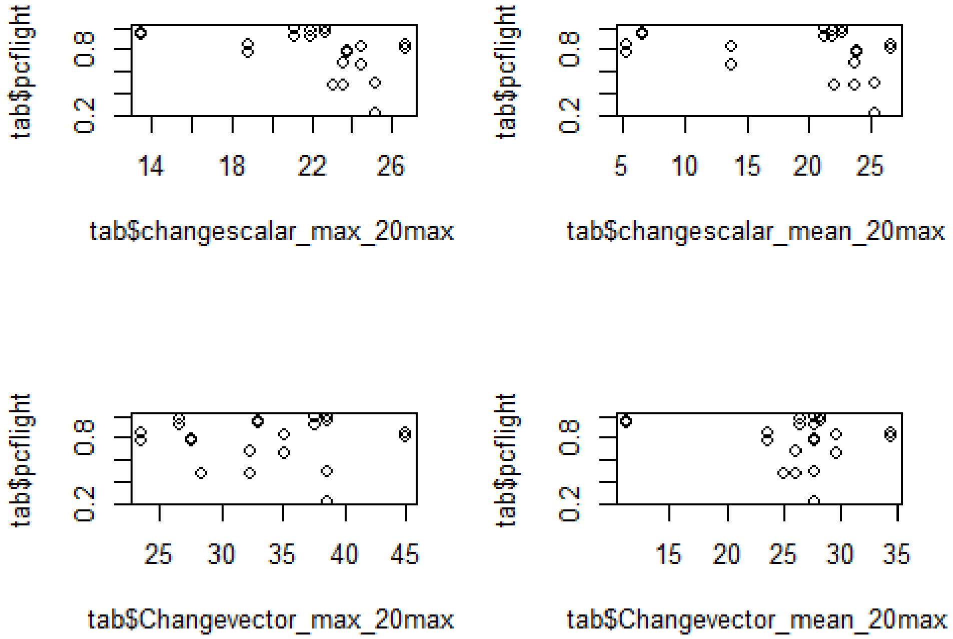


```
plot(tab$pcflight~tab$Angle_max_20max)
plot(tab$pcflight~tab$Angle_mean_20max)#*
plot(tab$pcflight~tab$Hz_max_20max)
plot(tab$pcflight~tab$Hz_mean_20max)
```

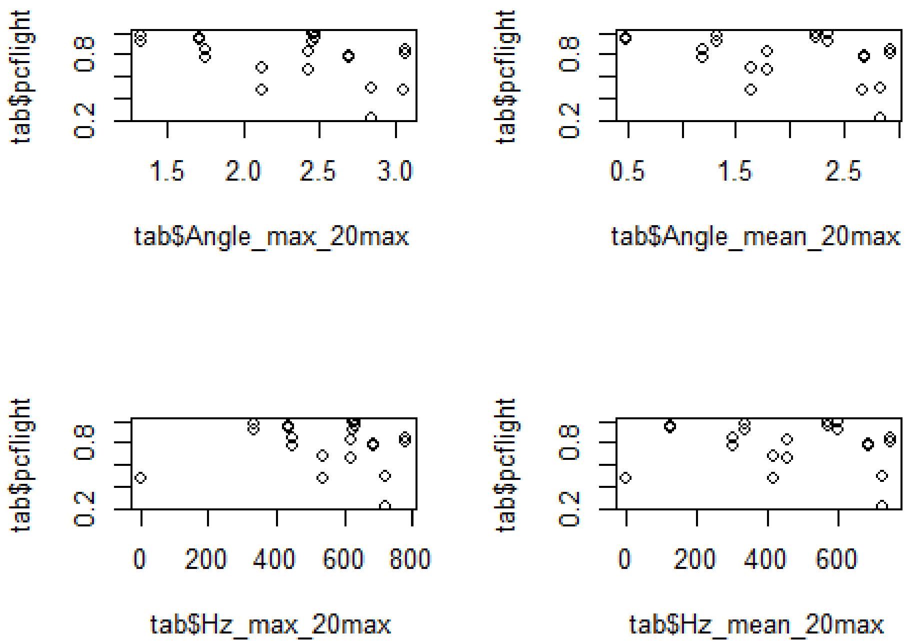

#Scatter plots at threshold 20-Mean

```
par(mfrow = c(2,2))

plot(tab$pcflight~tab$Duration.ms._20mean)
plot(tab$pcflight~tab$scalar_max_20mean)
plot(tab$pcflight~tab$scalar_mean_20mean)
plot(tab$pcflight~tab$changescalar_max_20mean)#*
```

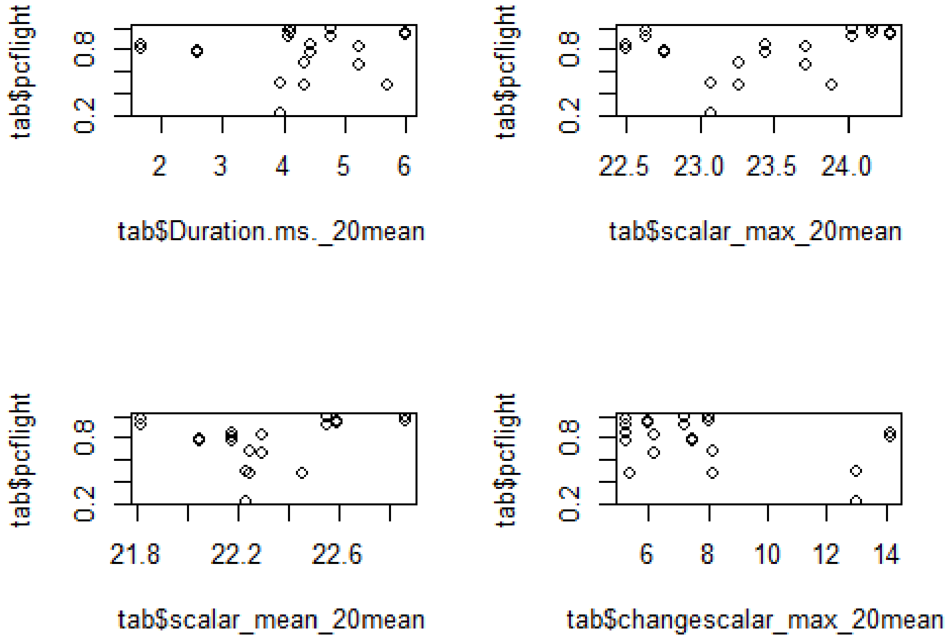


```
plot(tab$pcflight~tab$changescalar_mean_20mean)
plot(tab$pcflight~tab$Changevector_max_20mean) #*
plot(tab$pcflight~tab$Changevector_mean_20mean)
plot(tab$pcflight~tab$Angle_max_20mean)
```

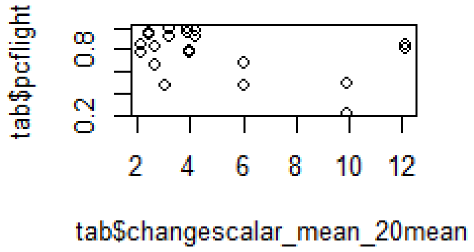

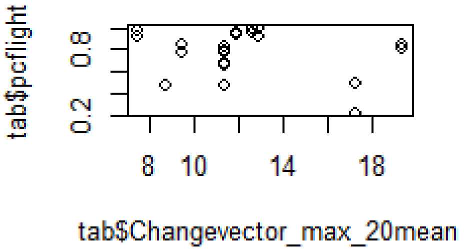

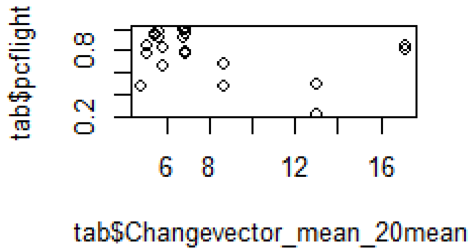

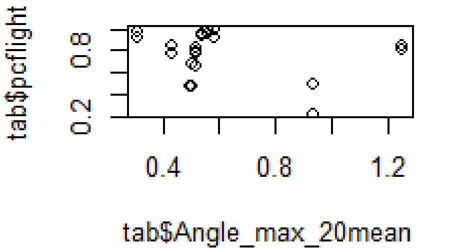


```
plot(tab$pcflight~tab$Angle_mean_20mean)#*
plot(tab$pcflight~tab$Hz_max_20mean)
plot(tab$pcflight~tab$Hz_mean_20mean)
```

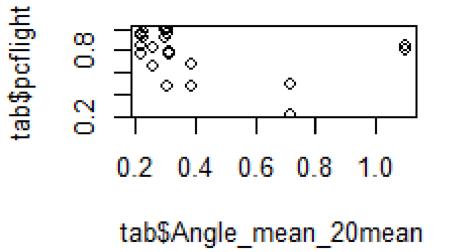

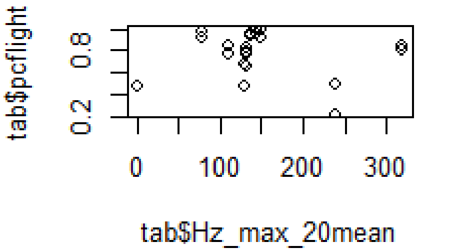

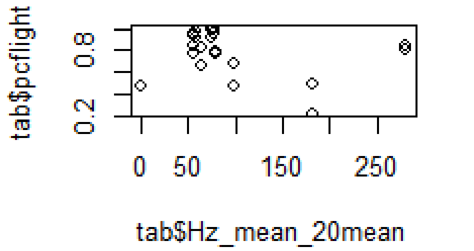

#Models of impact of transportation on the flight propensity

```
factor(tab$Age)

##  [1] 22 22 22 22 22 22 22 22 22 22 29 29 29 29 29 29 29 29 29 29 29 29
## Levels: 22 29

#####Caroline
#models threshold 5
fm1 <- glmer(cbind(out,in.) ~ Irradiation + RH._Max + Temp_mean +
(1|Replicate), family = binomial, data = tab)
fm2 <- glmer(cbind(out,in.) ~ Irradiation + RH._Max + Temp_mean +
changescalar_mean_5max + Changevector_mean_5max + (1|Replicate), family =
binomial, data = tab)
fm3 <- glmer(cbind(out,in.) ~ Irradiation + RH._Max + Temp_mean +
changescalar_mean_5max + Duration.ms._5mean +(1|Replicate), family =
binomial, data = tab)
fm4 <- glmer(cbind(out,in.) ~ Irradiation + RH._Max + Temp_mean +
changescalar_mean_5max +scalar_mean_5max + (1|Replicate), family = binomial,
data = tab)
fm5 <- glmer(cbind(out,in.) ~ Irradiation + RH._Max + Temp_mean +
changescalar_mean_5max + Duration.ms._5mean + Changevector_mean_5max +
(1|Replicate), family = binomial, data = tab)
fm6 <- glmer(cbind(out,in.) ~ Irradiation + RH._Max + Temp_mean +
changescalar_mean_5max + Angle_mean_5max + Duration.ms._5mean +
(1|Replicate), family = binomial, data = tab)
fm7 <- glmer(cbind(out,in.) ~ Irradiation + RH._Max + Temp_mean +
changescalar_mean_5max + Changevector_mean_5max +Duration.ms._5mean
+(1|Replicate), family = binomial, data = tab)
fm8 <- glmer(cbind(out,in.) ~ Irradiation + Temp_mean +
changescalar_mean_5max + Duration.ms._5max +Angle_mean_5max + (1|Replicate),
family = binomial, data = tab)
fm1a<- glmer(cbind(out,in.) ~ Irradiation + RH._Max + Temp_mean +
Duration.ms._5max +scalar_mean_5max + (1|Replicate), family = binomial, data
= tab)
fm1b <- glmer(cbind(out,in.) ~ Irradiation + RH._Max + Temp_mean +
scalar_mean_5max +changescalar_mean_5mean
+Changevector_mean_5max+(1|Replicate), family = binomial, data = tab)
fm1c <- glmer(cbind(out,in.) ~ Irradiation + RH._Max + Temp_mean +
scalar_mean_5max +changescalar_mean_5max +Changevector_mean_5mean +
(1|Replicate), family = binomial, data = tab)
fm1d <- glmer(cbind(out,in.) ~ Irradiation + RH._Max + Temp_mean +
scalar_mean_5max +changescalar_mean_5mean +Changevector_mean_5mean
+(1|Replicate), family = binomial, data = tab)
fm1e <- glmer(cbind(out,in.) ~ Irradiation + RH._Max + Temp_mean +
scalar_mean_5max +(1|Replicate), family = binomial, data = tab)
fm1f <- glmer(cbind(out,in.) ~ Irradiation + RH._Max + Temp_mean +
Angle_mean_5max +(1|Replicate), family = binomial, data = tab)

AICc(fm1,fm2,fm3,fm4,fm5,fm6,fm7,fm8,fm1a,fm1b,fm1c,fm1d,fm1e,fm1f)
```

```
##      df     AICc
## fm1   5 134.4313
## fm2   7 141.9350
## fm3   7 141.9404
## fm4   7 142.1797
## fm5   8 146.9868
## fm6   8 145.1096
## fm7   8 146.9868
## fm8   7 139.7695
## fm1a  7 140.9110
## fm1b  8 147.2285
## fm1c  8 147.2077
## fm1d  8 146.3989
## fm1e  6 137.8047
## fm1f  6 136.9130
```

```
summary(fm1)
```

```
## Generalized linear mixed model fit by maximum likelihood (Laplace
##   Approximation) [glmerMod]
##  Family: binomial  ( logit )
## Formula: cbind(out, in.) ~ Irradiation + RH._Max + Temp_mean + (1 |
Replicate)
##    Data: tab
##
##      AIC      BIC   logLik deviance df.resid
##    130.7    136.1    -60.3    120.7       17
##
## Scaled residuals:
##      Min       1Q   Median       3Q      Max
## -2.25506 -0.59387  0.09192  0.67108  1.70826
##
## Random effects:
##  Groups    Name        Variance Std.Dev.
##  Replicate (Intercept) 0.5234   0.7235
## Number of obs: 22, groups:  Replicate, 6
##
## Fixed effects:
##                   Estimate Std. Error z value Pr(>|z|)
## (Intercept)    -1.787084   0.893352  -2.000   0.0455 *
## Irradiationyes -0.019138   0.183743  -0.104   0.9170
## RH._Max         0.003914   0.010218   0.383   0.7017
## Temp_mean       0.184540   0.023423   7.879 3.31e-15 ***
## ---
## Signif. codes:  0 '***' 0.001 '**' 0.01 '*' 0.05 '.' 0.1 ' ' 1
##
## Correlation of Fixed Effects:
##             (Intr) Irrdtn RH._Mx
## Irradiatnys  0.114
```

```
## RH._Max      -0.837 -0.278
## Temp_mean    -0.517  0.014  0.133

##models for threshold 10

fm11 <- glmer(cbind(out,in.) ~ Irradiation + Temp_mean + Duration.ms._10max +
changescalar_mean_10max+Angle_mean_10max+ (1|Replicate), family = binomial,
data = tab)
fm12 <- glmer(cbind(out,in.) ~ Irradiation + Temp_mean + Duration.ms._10max +
changescalar_mean_10max+ Changevector_mean_10mean + (1|Replicate), family =
binomial, data = tab)
fm13 <- glmer(cbind(out,in.) ~ Irradiation + Temp_mean + Duration.ms._10max +
changescalar_mean_10max+ Changevector_mean_10max + (1|Replicate), family =
binomial, data = tab)
fm14 <- glmer(cbind(out,in.) ~ Irradiation + Duration.ms._10max +
changescalar_mean_10max+Changevector_mean_10max + (1|Replicate), family =
binomial, data = tab)
fm15 <- glmer(cbind(out,in.) ~ Irradiation + Temp_mean +Duration.ms._10mean +
changescalar_mean_10max+ (1|Replicate), family = binomial, data = tab)
fm11a <- glmer(cbind(out,in.) ~ Irradiation + Temp_mean + Duration.ms._10max
+ Changevector_mean_10mean +(1|Replicate), family = binomial, data = tab)
fm11b <- glmer(cbind(out,in.) ~ Irradiation + Temp_mean + Duration.ms._10mean
+ changescalar_mean_10max+scalar_max_10mean +(1|Replicate), family =
binomial, data = tab)
fm11c <- glmer(cbind(out,in.) ~ Irradiation + Temp_mean + Duration.ms._10mean
+ Changevector_mean_10mean +scalar_max_10mean +(1|Replicate), family =
binomial, data = tab)
fm11d <- glmer(cbind(out,in.) ~ Irradiation + Temp_mean +Duration.ms._10mean
+Changevector_mean_10mean + (1|Replicate), family = binomial, data = tab)
fm12a <- glmer(cbind(in.,out) ~ Temp_mean + Angle_mean_10max+ (1|Replicate),
family = binomial, data = tab)
fm12b <- glmer(cbind(in.,out) ~ Temp_mean + Duration.ms._10max+
(1|Replicate), family = binomial, data = tab)
fm12c <- glmer(cbind(in.,out) ~ Temp_mean + Angle_mean_10max+
Duration.ms._10max+(1|Replicate), family = binomial, data = tab)

AICc(fm1,fm2,fm3,fm4,fm5,fm6,fm7,fm8,fm1a,fm1b,fm1c,fm1d,fm1e,fm1f,fm12,fm13,
fm14,fm15,fm11a,fm11b,fm11c,fm11d,fm12a,fm12b,fm12c)

##         df     AICc
## fm1      5 134.4313
## fm2      7 141.9350
## fm3      7 141.9404
## fm4      7 142.1797
## fm5      8 146.9868
## fm6      8 145.1096
## fm7      8 146.9868
## fm8      7 139.7695
## fm1a     7 140.9110
## fm1b     8 147.2285
```

```
## fm1c   8 147.2077
## fm1d   8 146.3989
## fm1e   6 137.8047
## fm1f   6 136.9130
## fm12   7 140.0575
## fm13   7 139.7889
## fm14   6 151.6717
## fm15   6 135.4559
## fm11a  6 136.0822
## fm11b  7 139.6579
## fm11c  7 140.0867
## fm11d  6 137.2959
## fm12a  4 129.7124
## fm12b  4 128.9808
## fm12c  5 131.5295
```

```
summary(fm12b)
```

```
## Generalized linear mixed model fit by maximum likelihood (Laplace
##   Approximation) [glmerMod]
##  Family: binomial  ( logit )
## Formula: cbind(in., out) ~ Temp_mean + Duration.ms._10max + (1 |
Replicate)
##    Data: tab
##
##      AIC      BIC   logLik deviance df.resid
##    126.6    131.0    -59.3    118.6       18
##
## Scaled residuals:
##      Min       1Q   Median       3Q      Max
## -1.82448 -0.64343  0.04714  0.39654  2.04419
##
## Random effects:
##  Groups    Name        Variance Std.Dev.
##  Replicate (Intercept) 0.6306   0.7941
## Number of obs: 22, groups:  Replicate, 6
##
## Fixed effects:
##                    Estimate Std. Error z value Pr(>|z|)
## (Intercept)         1.80141    0.53515   3.366 0.000762 ***
## Temp_mean          -0.14364    0.03451  -4.163 3.14e-05 ***
## Duration.ms._10max -0.02380    0.01607  -1.482 0.138459
## ---
## Signif. codes:  0 '***' 0.001 '**' 0.01 '*' 0.05 '.' 0.1 ' ' 1
##
## Correlation of Fixed Effects:
##             (Intr) Tmp_mn
## Temp_mean   -0.137
## Drtn.ms._10 -0.403 -0.748
```

```r
#

##threshold 15
fm21 <- glmer(cbind(out,in.) ~ Irradiation + Temp_mean +
changescalar_mean_15max + scalar_mean_15mean + Duration.ms._15mean
+(1|Replicate), family = binomial, data = tab)
fm22 <- glmer(cbind(out,in.) ~ Irradiation + Temp_mean +
changescalar_mean_15max + Changevector_mean_15mean +(1|Replicate), family =
binomial, data = tab)
fm23 <- glmer(cbind(out,in.) ~ Irradiation + changescalar_mean_15max +
Changevector_mean_15max+scalar_mean_15mean + (1|Replicate), family =
binomial, data = tab)
fm24 <- glmer(cbind(out,in.) ~ Irradiation + changescalar_mean_15max +
Changevector_mean_15max+ Angle_mean_15max+Duration.ms._15mean +(1|Replicate),
family = binomial, data = tab)
fm25 <- glmer(cbind(out,in.) ~ Irradiation + changescalar_mean_15max +
Changevector_mean_15max+ Angle_mean_15max+ (1|Replicate), family = binomial,
data = tab)
fm26 <- glmer(cbind(out,in.) ~ Irradiation + changescalar_mean_15max +
Changevector_mean_15max+scalar_mean_15mean + Duration.ms._15mean
+(1|Replicate), family = binomial, data = tab)
fm27 <- glmer(cbind(out,in.) ~ Irradiation +
Changevector_mean_15max+scalar_mean_15mean + (1|Replicate), family =
binomial, data = tab)
fm28 <- glmer(cbind(out,in.) ~ Irradiation + Changevector_mean_15max+
changescalar_mean_15mean +(1|Replicate), family = binomial, data = tab)
fm29 <- glmer(cbind(out,in.) ~ Irradiation + changescalar_mean_15max +
Changevector_mean_15max+(1|Replicate), family = binomial, data = tab)
fm30 <- glmer(cbind(out,in.) ~ Irradiation +Temp_mean +Angle_mean_15max +
Changevector_mean_15mean +(1|Replicate), family = binomial, data = tab)
fm30a <-glmer(cbind(out,in.) ~ Irradiation +Temp_mean + Duration.ms._15mean
+changescalar_mean_15mean + Changevector_mean_15mean +(1|Replicate), family =
binomial, data = tab)
fm29a<- glmer(cbind(out,in.) ~ Irradiation + changescalar_mean_15max +
Changevector_mean_15max+ Duration.ms._15mean +scalar_mean_15mean
+(1|Replicate), family = binomial, data = tab)
fm29b <- glmer(cbind(in.,out) ~ Age +Temp_mean+ Angle_mean_15max+
(1|Replicate), family = binomial, data = tab)
fm29c <- glmer(cbind(in.,out) ~ Temp_mean + Angle_mean_15max+ (1|Replicate),
family = binomial, data = tab)
fm29d <- glmer(cbind(in.,out) ~ Age + Angle_mean_15max+ (1|Replicate), family
= binomial, data = tab)

AICc(fm1,fm2,fm3,fm4,fm5,fm6,fm7,fm8,fm1a,fm1b,fm1c,fm1d,fm1e,fm1f,fm13,fm14,
fm15,fm11a,fm11b,fm11c,fm11d,fm12a,fm12b,fm12c,fm21,fm22,fm23,fm24,fm25,fm26,
fm27,fm28,fm29,fm30,fm30a,fm29a,fm29b,fm29c,fm29d)

##        df     AICc
## fm1     5 134.4313
## fm2     7 141.9350
```

```
## fm3    7 141.9404
## fm4    7 142.1797
## fm5    8 146.9868
## fm6    8 145.1096
## fm7    8 146.9868
## fm8    7 139.7695
## fm1a   7 140.9110
## fm1b   8 147.2285
## fm1c   8 147.2077
## fm1d   8 146.3989
## fm1e   6 137.8047
## fm1f   6 136.9130
## fm13   7 139.7889
## fm14   6 151.6717
## fm15   6 135.4559
## fm11a  6 136.0822
## fm11b  7 139.6579
## fm11c  7 140.0867
## fm11d  6 137.2959
## fm12a  4 129.7124
## fm12b  4 128.9808
## fm12c  5 131.5295
## fm21   7 139.7218
## fm22   6 138.0995
## fm23   6 140.4174
## fm24   7 142.8149
## fm25   6 138.5834
## fm26   7 144.7207
## fm27   5 138.9408
## fm28   5 143.7079
## fm29   5 140.7846
## fm30   6 135.2286
## fm30a  7 141.2747
## fm29a  7 144.7207
## fm29b  5 127.1254
## fm29c  4 128.1561
## fm29d  4 130.2264
```

```
summary(fm29b)
```

```
## Generalized linear mixed model fit by maximum likelihood (Laplace
##   Approximation) [glmerMod]
##  Family: binomial  ( logit )
## Formula: cbind(in., out) ~ Age + Temp_mean + Angle_mean_15max + (1 |
Replicate)
##    Data: tab
##
##      AIC      BIC   logLik deviance df.resid
##    123.4    128.8    -56.7    113.4       17
##
```

```
## Scaled residuals:
##      Min       1Q   Median       3Q      Max
## -1.84397 -0.45397  0.04892  0.73451  2.07606
##
## Random effects:
##  Groups    Name        Variance Std.Dev.
##  Replicate (Intercept) 0.2272   0.4767
## Number of obs: 22, groups:  Replicate, 6
##
## Fixed effects:
##                  Estimate Std. Error z value Pr(>|z|)
## (Intercept)       13.8621     6.0125   2.306 0.021137 *
## Age               -0.3545     0.1487  -2.384 0.017143 *
## Temp_mean         -0.3967     0.1186  -3.345 0.000823 ***
## Angle_mean_15max   0.3097     0.2603   1.190 0.234142
## ---
## Signif. codes:  0 '***' 0.001 '**' 0.01 '*' 0.05 '.' 0.1 ' ' 1
##
## Correlation of Fixed Effects:
##             (Intr) Age    Tmp_mn
## Age         -0.984
## Temp_mean   -0.984  0.943
## Angl_mn_15m -0.432  0.278  0.523
```

```
##Threshold 20

fm31 <- glmer(cbind(out,in.) ~ Irradiation + Temp_mean + Duration.ms._20max +
changescalar_mean_20max + Angle_mean_20max + (1|Replicate), family =
binomial, data = tab)
fm32 <- glmer(cbind(out,in.) ~ Irradiation + Temp_mean +
changescalar_mean_20mean+ Angle_mean_20mean +(1|Replicate), family =
binomial, data = tab)
fm31a <- glmer(cbind(out,in.) ~ Irradiation + Temp_mean + Duration.ms._20max
+ changescalar_mean_20max + Angle_mean_20mean + (1|Replicate), family =
binomial, data = tab)
fm32a <- glmer(cbind(out,in.) ~ Irradiation + Temp_mean +
changescalar_mean_20max +Angle_mean_20max +(1|Replicate), family = binomial,
data = tab)

AICc(fm1,fm2,fm3,fm4,fm5,fm6,fm7,fm8,fm1a,fm1b,fm1c,fm1d,fm1e,fm1f,fm13,fm14,
fm15,fm11a,fm11b,fm11c,fm11d,fm12a,fm12b,fm12c,fm21,fm22,fm23,fm24,fm25,fm26,
fm27,fm28,fm29,fm30,fm30a,fm29a,fm29b,fm29c,fm29d,fm31,fm31a,fm32,fm32a)
```

```
##       df     AICc
## fm1    5 134.4313
## fm2    7 141.9350
## fm3    7 141.9404
## fm4    7 142.1797
## fm5    8 146.9868
## fm6    8 145.1096
```

```
## fm7    8 146.9868
## fm8    7 139.7695
## fm1a   7 140.9110
## fm1b   8 147.2285
## fm1c   8 147.2077
## fm1d   8 146.3989
## fm1e   6 137.8047
## fm1f   6 136.9130
## fm13   7 139.7889
## fm14   6 151.6717
## fm15   6 135.4559
## fm11a  6 136.0822
## fm11b  7 139.6579
## fm11c  7 140.0867
## fm11d  6 137.2959
## fm12a  4 129.7124
## fm12b  4 128.9808
## fm12c  5 131.5295
## fm21   7 139.7218
## fm22   6 138.0995
## fm23   6 140.4174
## fm24   7 142.8149
## fm25   6 138.5834
## fm26   7 144.7207
## fm27   5 138.9408
## fm28   5 143.7079
## fm29   5 140.7846
## fm30   6 135.2286
## fm30a  7 141.2747
## fm29a  7 144.7207
## fm29b  5 127.1254
## fm29c  4 128.1561
## fm29d  4 130.2264
## fm31   7 138.8322
## fm31a  7 138.9801
## fm32   6 137.0564
## fm32a  6 135.4027
```

```
summary(fm29b)
```

```
## Generalized linear mixed model fit by maximum likelihood (Laplace
##   Approximation) [glmerMod]
##  Family: binomial  ( logit )
## Formula: cbind(in., out) ~ Age + Temp_mean + Angle_mean_15max + (1 |
Replicate)
##    Data: tab
## 
##      AIC      BIC   logLik deviance df.resid
##    123.4    128.8    -56.7    113.4       17
## 
```

```
## Scaled residuals: 
##      Min       1Q   Median       3Q      Max 
## -1.84397 -0.45397  0.04892  0.73451  2.07606 
## 
## Random effects:
##  Groups    Name        Variance Std.Dev.
##  Replicate (Intercept) 0.2272   0.4767  
## Number of obs: 22, groups:  Replicate, 6
## 
## Fixed effects:
##                  Estimate Std. Error z value Pr(>|z|)    
## (Intercept)       13.8621     6.0125   2.306 0.021137 *  
## Age               -0.3545     0.1487  -2.384 0.017143 *  
## Temp_mean         -0.3967     0.1186  -3.345 0.000823 ***
## Angle_mean_15max   0.3097     0.2603   1.190 0.234142    
## ---
## Signif. codes:  0 '***' 0.001 '**' 0.01 '*' 0.05 '.' 0.1 ' ' 1
## 
## Correlation of Fixed Effects:
##             (Intr) Age    Tmp_mn
## Age         -0.984              
## Temp_mean   -0.984  0.943       
## Angl_mn_15m -0.432  0.278  0.523

#combination of thresholds
fm41 <- glmer(cbind(out,in.) ~ Irradiation + Temp_mean + RH._Max + 
changescalar_mean_10max + Angle_mean_15max + Changevector_mean_15mean 
+(1|Replicate), family = binomial, data = tab)
fm42 <- glmer(cbind(out,in.) ~ Irradiation +Temp_mean + RH._Max + 
changescalar_mean_20max +Angle_mean_20max + Duration.ms._10mean 
+(1|Replicate), family = binomial, data = tab)

AICc(fm1,fm2,fm3,fm4,fm5,fm6,fm7,fm8,fm13,fm14,fm15,fm11a,fm11b,fm11c,fm11d,f
m21,fm22,fm23,fm24,fm25,fm26,fm27,fm28,fm29,fm30,fm30a,fm29a,fm29b,fm29c,fm29
d,fm31,fm32,fm31a,fm32a,fm41,fm42)

##       df     AICc
## fm1    5 134.4313
## fm2    7 141.9350
## fm3    7 141.9404
## fm4    7 142.1797
## fm5    8 146.9868
## fm6    8 145.1096
## fm7    8 146.9868
## fm8    7 139.7695
## fm13   7 139.7889
## fm14   6 151.6717
## fm15   6 135.4559
## fm11a  6 136.0822
## fm11b  7 139.6579
```

```
## fm11c  7 140.0867
## fm11d  6 137.2959
## fm21   7 139.7218
## fm22   6 138.0995
## fm23   6 140.4174
## fm24   7 142.8149
## fm25   6 138.5834
## fm26   7 144.7207
## fm27   5 138.9408
## fm28   5 143.7079
## fm29   5 140.7846
## fm30   6 135.2286
## fm30a  7 141.2747
## fm29a  7 144.7207
## fm29b  5 127.1254
## fm29c  4 128.1561
## fm29d  4 130.2264
## fm31   7 138.8322
## fm32   6 137.0564
## fm31a  7 138.9801
## fm32a  6 135.4027
## fm41   8 144.4872
## fm42   8 144.8514

#the best model remains as fm29b

##Figure 9b

head(tab)

##     Date Replicate Age     Treatments out in. shipped Irradiation
Events._5max
## 1 170510        R1  22 Shipped-110Gy   23   6     yes         yes
909
## 2 170510        R1  22   Shipped-0Gy   24   7     yes          no
909
## 3 170517        R2  22 Shipped-110Gy    2   1     yes         yes
708
## 4 170517        R2  22   Shipped-0Gy    5   1     yes          no
708
## 5 170524        R3  22 Shipped-110Gy   75   4     yes         yes
1187
## 6 170524        R3  22   Shipped-0Gy   84   5     yes          no
1187
##   Duration.ms._5max scalar_max_5max scalar_mean_5max changescalar_max_5max
## 1            48.750          27.374         21.43900                23.724
## 2            48.750          27.374         21.43900                23.724
## 3            87.500          27.398         16.92723                24.477
## 4            87.500          27.398         16.92723                24.477
## 5            93.125          27.374         15.43705                16.627
```

```
## 6            93.125          27.374         15.43705                16.627
##   changescalar_mean_5max Changevector_max_5max Changevector_mean_5max
## 1                8.94875              27.69156               9.937386
## 2                8.94875              27.69156               9.937386
## 3               11.37550              35.06987              19.035967
## 4               11.37550              35.06987              19.035967
## 5                3.83800              33.00443               5.064434
## 6                3.83800              33.00443               5.064434
##   Angle_max_5max Angle_mean_5max Hz_max_5max Hz_mean_5max Events_10max
## 1       3.002116        2.617132    764.4826     666.4471          288
## 2       3.002116        2.617132    764.4826     666.4471          288
## 3       2.969360        1.679485    756.1414     427.6775          315
## 4       2.969360        1.679485    756.1414     427.6775          315
## 5       2.105922        1.356174    536.2686     345.3468          238
## 6       2.105922        1.356174    536.2686     345.3468          238
##   Duration.ms._10max scalar_max_10max scalar_mean_10max
changescalar_max_10max
## 1             36.875           27.374           21.5800
23.724
## 2             36.875           27.374           21.5800
23.724
## 3             50.000           27.398           20.3750
24.477
## 4             50.000           27.398           20.3750
24.477
## 5             63.750           27.374           19.6895
16.627
## 6             63.750           27.374           19.6895
16.627
##   changescalar_mean_10max Changevector_max_10max Changevector_mean_10max
## 1                  12.473               27.69156                15.45774
## 2                  12.473               27.69156                15.45774
## 3                  16.488               35.06987                22.58486
## 4                  16.488               35.06987                22.58486
## 5                   8.965               33.00443                10.65686
## 6                   8.965               33.00443                10.65686
##   Angle_max_10max Angle_mean_10max Hz_max_10max Hz_mean_10max Events_15max
## 1        2.687392        1.0470779     684.3388      266.6362          144
## 2        2.687392        1.0470779     684.3388      266.6362          144
## 3        2.969360        1.6138687     756.1414      410.9683          183
## 4        2.969360        1.6138687     756.1414      410.9683          183
## 5        2.105922        0.5851573     536.2686      149.0091          111
## 6        2.105922        0.5851573     536.2686      149.0091          111
##   Duration.ms._15max scalar_max_15max scalar_mean_15max
changescalar_max_15max
## 1             30.625           27.374          22.42986
23.724
## 2             30.625           27.374          22.42986
23.724
## 3             29.375           27.398          21.71558
```

```
24.477
## 4            29.375           27.398          21.71558
24.477
## 5            41.250           27.374          22.77170
16.627
## 6            41.250           27.374          22.77170
16.627
##   changescalar_mean_15max Changevector_max_15max Changevector_mean_15max
## 1                  15.667               27.69156                19.75723
## 2                  15.667               27.69156                19.75723
## 3                  16.488               35.06987                21.61531
## 4                  16.488               35.06987                21.61531
## 5                  11.474               33.00443                12.83840
## 6                  11.474               33.00443                12.83840
##   Angle_max_15max Angle_mean_15max Hz_max_15max Hz_mean_15max Events_20max
## 1        2.687392        1.8427590     684.3388      469.2547           57
## 2        2.687392        1.8427590     684.3388      469.2547           57
## 3        2.969360        1.6010884     756.1414      407.7138           75
## 4        2.969360        1.6010884     756.1414      407.7138           75
## 5        2.105922        0.6709018     536.2686      170.8438           42
## 6        2.105922        0.6709018     536.2686      170.8438           42
##   Duration.ms._20max scalar_max_20max scalar_mean_20max
changescalar_max_20max
## 1            17.500           27.374          26.80175
23.724
## 2            17.500           27.374          26.80175
23.724
## 3            20.625           27.398          26.38567
24.477
## 4            20.625           27.398          26.38567
24.477
## 5            20.625           27.374          25.17900
13.480
## 6            20.625           27.374          25.17900
13.480
##   changescalar_mean_20max Changevector_max_20max Changevector_mean_20max
## 1                 23.7240               27.69156                27.69156
## 2                 23.7240               27.69156                27.69156
## 3                 13.7095               35.06987                29.65156
## 4                 13.7095               35.06987                29.65156
## 5                  6.6090               33.00443                11.19072
## 6                  6.6090               33.00443                11.19072
##   Angle_max_20max Angle_mean_20max Hz_max_20max Hz_mean_20max Events_5mean
## 1        2.687392         2.687392     684.3388      684.3388          909
## 2        2.687392         2.687392     684.3388      684.3388          909
## 3        2.433775         1.792941     619.7557      456.5688          708
## 4        2.433775         1.792941     619.7557      456.5688          708
## 5        1.715913         0.489164     436.9538      124.5646         1187
## 6        1.715913         0.489164     436.9538      124.5646         1187
##   Duration.ms._5mean scalar_max_5mean scalar_mean_5mean
```

```
changescalar_max_5mean
## 1          5.215209         9.516648          7.473747
3.755884
## 2          5.215209         9.516648          7.473747
3.755884
## 3         11.785840        10.551797          7.565272
2.816095
## 4         11.785840        10.551797          7.565272
2.816095
## 5          7.348884         8.022198          6.506753
1.632164
## 6          7.348884         8.022198          6.506753
1.632164
##   changescalar_mean_5mean Changevector_max_5mean Changevector_mean_5mean
## 1               1.6630629               4.501180                2.219564
## 2               1.6630629               4.501180                2.219564
## 3               1.0076953               3.691769                1.638802
## 4               1.0076953               3.691769                1.638802
## 5               0.6804169               2.293485                1.194539
## 6               0.6804169               2.293485                1.194539
##   Angle_max_5mean Angle_mean_5mean Hz_max_5mean Hz_mean_5mean
Events._10mean
## 1       0.4296757        0.1755708    109.41603      44.70874
288
## 2       0.4296757        0.1755708    109.41603      44.70874
288
## 3       0.3715939        0.1414327     94.62562      36.01553
315
## 4       0.3715939        0.1414327     94.62562      36.01553
315
## 5       0.2539263        0.1310499     64.66179      33.37158
238
## 6       0.2539263        0.1310499     64.66179      33.37158
238
##   Duration.ms._10mean scalar_max_10mean scalar_mean_10mean
## 1            3.888889          15.49537           13.49979
## 2            3.888889          15.49537           13.49979
## 3            7.706349          16.38244           13.65682
## 4            7.706349          16.38244           13.65682
## 5            7.415966          15.56483           13.15894
## 6            7.415966          15.56483           13.15894
##   changescalar_max_10mean changescalar_mean_10mean Changevector_max_10mean
## 1                6.557340                 3.249119                8.029862
## 2                6.557340                 3.249119                8.029862
## 3                4.928854                 1.971893                6.505797
## 4                4.928854                 1.971893                6.505797
## 5                4.004025                 1.664277                5.891075
## 6                4.004025                 1.664277                5.891075
##   Changevector_mean_10mean Angle_max_10mean Angle_mean_10mean
Hz_max_10mean
```

```
## 1                 4.538349       0.4719538         0.2224823
120.1821
## 2                 4.538349       0.4719538         0.2224823
120.1821
## 3                 3.339135       0.4047630         0.1751144
103.0721
## 4                 3.339135       0.4047630         0.1751144
103.0721
## 5                 2.909783       0.3492638         0.1583697
88.9393
## 6                 2.909783       0.3492638         0.1583697
88.9393
##   Hz_mean_10mean Events_15mean Duration.ms._15mean scalar_max_15mean
## 1       56.65466           144            3.441840          19.26462
## 2       56.65466           144            3.441840          19.26462
## 3       44.59251           183            6.342213          19.93954
## 4       44.59251           183            6.342213          19.93954
## 5       40.32851           111            6.920045          19.71768
## 6       40.32851           111            6.920045          19.71768
##   scalar_mean_15mean changescalar_max_15mean changescalar_mean_15mean
## 1           17.94158                8.195410                 4.209463
## 2           17.94158                8.195410                 4.209463
## 3           17.79343                6.271208                 2.424063
## 4           17.79343                6.271208                 2.424063
## 5           17.60073                5.291784                 2.055466
## 6           17.60073                5.291784                 2.055466
##   Changevector_max_15mean Changevector_mean_15mean Angle_max_15mean
## 1               10.145769                 6.194357        0.4732660
## 2               10.145769                 6.194357        0.4732660
## 3                8.773143                 4.485467        0.4584332
## 4                8.773143                 4.485467        0.4584332
## 5                8.644509                 4.178605        0.4437400
## 6                8.644509                 4.178605        0.4437400
##   Angle_mean_15mean Hz_max_15mean Hz_mean_15mean Events_20mean
## 1         0.2644405      120.5162       67.33922            57
## 2         0.2644405      120.5162       67.33922            57
## 3         0.2042285      116.7390       52.00635            75
## 4         0.2042285      116.7390       52.00635            75
## 5         0.1960600      112.9975       49.92626            42
## 6         0.1960600      112.9975       49.92626            42
##   Duration.ms._20mean scalar_max_20mean scalar_mean_20mean
## 1            2.587719          22.76440           22.04857
## 2            2.587719          22.76440           22.04857
## 3            5.250000          23.71752           22.29175
## 4            5.250000          23.71752           22.29175
## 5            5.997024          24.28693           22.58697
## 6            5.997024          24.28693           22.58697
##   changescalar_max_20mean changescalar_mean_20mean Changevector_max_20mean
## 1                7.502070                 3.960703                11.35123
## 2                7.502070                 3.960703                11.35123
```

```
## 3                6.190293                 2.676377                11.36833
## 4                6.190293                 2.676377                11.36833
## 5                5.996190                 2.445063                11.97291
## 6                5.996190                 2.445063                11.97291
##   Changevector_mean_20mean Angle_max_20mean Angle_mean_20mean
Hz_max_20mean
## 1                 6.922532        0.5201012         0.3140733
132.4427
## 2                 6.922532        0.5201012         0.3140733
132.4427
## 3                 5.877764        0.5214004         0.2548023
132.7735
## 4                 5.877764        0.5214004         0.2548023
132.7735
## 5                 5.476046        0.5391966         0.2201107
137.3053
## 6                 5.476046        0.5391966         0.2201107
137.3053
##   Hz_mean_20mean RH._Max Temp_Max RH._mean Temp_mean  pcflight
## 1       79.97812   47.08    23.50    41.69     19.73 0.7931034
## 2       79.97812   60.14    27.50    41.69     19.73 0.7741935
## 3       64.88487   62.61    29.75    53.55     21.79 0.6666667
## 4       64.88487   68.10    32.00    53.55     21.79 0.8333333
## 5       56.05073   57.37    28.69    53.25     23.29 0.9493671
## 6       56.05073   74.59    19.25    53.25     23.29 0.9438202
```

```
str(tab)
```

```
## 'data.frame':    22 obs. of  109 variables:
##  $ Date                     : int  170510 170510 170517 170517 170524
170524 170531 170531 170607 170607 ...
##  $ Replicate                : chr  "R1" "R1" "R2" "R2" ...
##  $ Age                      : int  22 22 22 22 22 22 22 22 22 22 ...
##  $ Treatments               : chr  "Shipped-110Gy " "Shipped-0Gy "
"Shipped-110Gy " "Shipped-0Gy " ...
##  $ out                      : int  23 24 2 5 75 84 73 75 76 95 ...
##  $ in.                      : int  6 7 1 1 4 5 7 0 4 0 ...
##  $ shipped                  : chr  "yes" "yes" "yes" "yes" ...
##  $ Irradiation              : chr  "yes" "no" "yes" "no" ...
##  $ Events._5max             : int  909 909 708 708 1187 1187 625 625 606
606 ...
##  $ Duration.ms._5max        : num  48.8 48.8 87.5 87.5 93.1 ...
##  $ scalar_max_5max          : num  27.4 27.4 27.4 27.4 27.4 ...
##  $ scalar_mean_5max         : num  21.4 21.4 16.9 16.9 15.4 ...
##  $ changescalar_max_5max    : num  23.7 23.7 24.5 24.5 16.6 ...
##  $ changescalar_mean_5max   : num  8.95 8.95 11.38 11.38 3.84 ...
##  $ Changevector_max_5max    : num  27.7 27.7 35.1 35.1 33 ...
##  $ Changevector_mean_5max   : num  9.94 9.94 19.04 19.04 5.06 ...
##  $ Angle_max_5max           : num  3 3 2.97 2.97 2.11 ...
##  $ Angle_mean_5max          : num  2.62 2.62 1.68 1.68 1.36 ...
```

```
##  $ Hz_max_5max            : num  764 764 756 756 536 ...
##  $ Hz_mean_5max           : num  666 666 428 428 345 ...
##  $ Events_10max           : int  288 288 315 315 238 238 261 261 219 219
...
##  $ Duration.ms._10max      : num  36.9 36.9 50 50 63.8 ...
##  $ scalar_max_10max        : num  27.4 27.4 27.4 27.4 27.4 ...
##  $ scalar_mean_10max       : num  21.6 21.6 20.4 20.4 19.7 ...
##  $ changescalar_max_10max  : num  23.7 23.7 24.5 24.5 16.6 ...
##  $ changescalar_mean_10max : num  12.47 12.47 16.49 16.49 8.97 ...
##  $ Changevector_max_10max  : num  27.7 27.7 35.1 35.1 33 ...
##  $ Changevector_mean_10max : num  15.5 15.5 22.6 22.6 10.7 ...
##  $ Angle_max_10max         : num  2.69 2.69 2.97 2.97 2.11 ...
##  $ Angle_mean_10max        : num  1.047 1.047 1.614 1.614 0.585 ...
##  $ Hz_max_10max            : num  684 684 756 756 536 ...
##  $ Hz_mean_10max           : num  267 267 411 411 149 ...
##  $ Events_15max            : int  144 144 183 183 111 111 163 163 129 129
...
##  $ Duration.ms._15max      : num  30.6 30.6 29.4 29.4 41.2 ...
##  $ scalar_max_15max        : num  27.4 27.4 27.4 27.4 27.4 ...
##  $ scalar_mean_15max       : num  22.4 22.4 21.7 21.7 22.8 ...
##  $ changescalar_max_15max  : num  23.7 23.7 24.5 24.5 16.6 ...
##  $ changescalar_mean_15max : num  15.7 15.7 16.5 16.5 11.5 ...
##  $ Changevector_max_15max  : num  27.7 27.7 35.1 35.1 33 ...
##  $ Changevector_mean_15max : num  19.8 19.8 21.6 21.6 12.8 ...
##  $ Angle_max_15max         : num  2.69 2.69 2.97 2.97 2.11 ...
##  $ Angle_mean_15max        : num  1.843 1.843 1.601 1.601 0.671 ...
##  $ Hz_max_15max            : num  684 684 756 756 536 ...
##  $ Hz_mean_15max           : num  469 469 408 408 171 ...
##  $ Events_20max            : int  57 57 75 75 42 42 97 97 71 71 ...
##  $ Duration.ms._20max      : num  17.5 17.5 20.6 20.6 20.6 ...
##  $ scalar_max_20max        : num  27.4 27.4 27.4 27.4 27.4 ...
##  $ scalar_mean_20max       : num  26.8 26.8 26.4 26.4 25.2 ...
##  $ changescalar_max_20max  : num  23.7 23.7 24.5 24.5 13.5 ...
##  $ changescalar_mean_20max : num  23.72 23.72 13.71 13.71 6.61 ...
##  $ Changevector_max_20max  : num  27.7 27.7 35.1 35.1 33 ...
##  $ Changevector_mean_20max : num  27.7 27.7 29.7 29.7 11.2 ...
##  $ Angle_max_20max         : num  2.69 2.69 2.43 2.43 1.72 ...
##  $ Angle_mean_20max        : num  2.687 2.687 1.793 1.793 0.489 ...
##  $ Hz_max_20max            : num  684 684 620 620 437 ...
##  $ Hz_mean_20max           : num  684 684 457 457 125 ...
##  $ Events_5mean            : int  909 909 708 708 1187 1187 625 625 606
606 ...
##  $ Duration.ms._5mean      : num  5.22 5.22 11.79 11.79 7.35 ...
##  $ scalar_max_5mean        : num  9.52 9.52 10.55 10.55 8.02 ...
##  $ scalar_mean_5mean       : num  7.47 7.47 7.57 7.57 6.51 ...
##  $ changescalar_max_5mean  : num  3.76 3.76 2.82 2.82 1.63 ...
##  $ changescalar_mean_5mean : num  1.66 1.66 1.01 1.01 0.68 ...
##  $ Changevector_max_5mean  : num  4.5 4.5 3.69 3.69 2.29 ...
##  $ Changevector_mean_5mean : num  2.22 2.22 1.64 1.64 1.19 ...
##  $ Angle_max_5mean         : num  0.43 0.43 0.372 0.372 0.254 ...
```

```
##  $ Angle_mean_5mean        : num  0.176 0.176 0.141 0.141 0.131 ...
##  $ Hz_max_5mean            : num  109.4 109.4 94.6 94.6 64.7 ...
##  $ Hz_mean_5mean           : num  44.7 44.7 36 36 33.4 ...
##  $ Events._10mean          : int  288 288 315 315 238 238 261 261 219 219
...
##  $ Duration.ms._10mean     : num  3.89 3.89 7.71 7.71 7.42 ...
##  $ scalar_max_10mean       : num  15.5 15.5 16.4 16.4 15.6 ...
##  $ scalar_mean_10mean      : num  13.5 13.5 13.7 13.7 13.2 ...
##  $ changescalar_max_10mean : num  6.56 6.56 4.93 4.93 4 ...
##  $ changescalar_mean_10mean: num  3.25 3.25 1.97 1.97 1.66 ...
##  $ Changevector_max_10mean : num  8.03 8.03 6.51 6.51 5.89 ...
##  $ Changevector_mean_10mean: num  4.54 4.54 3.34 3.34 2.91 ...
##  $ Angle_max_10mean        : num  0.472 0.472 0.405 0.405 0.349 ...
##  $ Angle_mean_10mean       : num  0.222 0.222 0.175 0.175 0.158 ...
##  $ Hz_max_10mean           : num  120.2 120.2 103.1 103.1 88.9 ...
##  $ Hz_mean_10mean          : num  56.7 56.7 44.6 44.6 40.3 ...
##  $ Events_15mean           : int  144 144 183 183 111 111 163 163 129 129
...
##  $ Duration.ms._15mean     : num  3.44 3.44 6.34 6.34 6.92 ...
##  $ scalar_max_15mean       : num  19.3 19.3 19.9 19.9 19.7 ...
##  $ scalar_mean_15mean      : num  17.9 17.9 17.8 17.8 17.6 ...
##  $ changescalar_max_15mean : num  8.2 8.2 6.27 6.27 5.29 ...
##  $ changescalar_mean_15mean: num  4.21 4.21 2.42 2.42 2.06 ...
##  $ Changevector_max_15mean : num  10.15 10.15 8.77 8.77 8.64 ...
##  $ Changevector_mean_15mean: num  6.19 6.19 4.49 4.49 4.18 ...
##  $ Angle_max_15mean        : num  0.473 0.473 0.458 0.458 0.444 ...
##  $ Angle_mean_15mean       : num  0.264 0.264 0.204 0.204 0.196 ...
##  $ Hz_max_15mean           : num  121 121 117 117 113 ...
##  $ Hz_mean_15mean          : num  67.3 67.3 52 52 49.9 ...
##  $ Events_20mean           : int  57 57 75 75 42 42 97 97 71 71 ...
##  $ Duration.ms._20mean     : num  2.59 2.59 5.25 5.25 6 ...
##  $ scalar_max_20mean       : num  22.8 22.8 23.7 23.7 24.3 ...
##  $ scalar_mean_20mean      : num  22 22 22.3 22.3 22.6 ...
##  $ changescalar_max_20mean : num  7.5 7.5 6.19 6.19 6 ...
##  $ changescalar_mean_20mean: num  3.96 3.96 2.68 2.68 2.45 ...
##  $ Changevector_max_20mean : num  11.4 11.4 11.4 11.4 12 ...
##   [list output truncated]
```

```
summary(fm29b)
```

```
## Generalized linear mixed model fit by maximum likelihood (Laplace
##   Approximation) [glmerMod]
##  Family: binomial  ( logit )
## Formula: cbind(in., out) ~ Age + Temp_mean + Angle_mean_15max + (1 |
Replicate)
##    Data: tab
##
##      AIC      BIC   logLik deviance df.resid
##    123.4    128.8    -56.7    113.4       17
##
```

```
## Scaled residuals:
##      Min       1Q   Median       3Q      Max
## -1.84397 -0.45397  0.04892  0.73451  2.07606
##
## Random effects:
##  Groups    Name        Variance Std.Dev.
##  Replicate (Intercept) 0.2272   0.4767
## Number of obs: 22, groups:  Replicate, 6
##
## Fixed effects:
##                   Estimate Std. Error z value Pr(>|z|)
## (Intercept)        13.8621     6.0125   2.306 0.021137 *
## Age                -0.3545     0.1487  -2.384 0.017143 *
## Temp_mean          -0.3967     0.1186  -3.345 0.000823 ***
## Angle_mean_15max    0.3097     0.2603   1.190 0.234142
## ---
## Signif. codes:  0 '***' 0.001 '**' 0.01 '*' 0.05 '.' 0.1 ' ' 1
##
## Correlation of Fixed Effects:
##              (Intr) Age    Tmp_mn
## Age          -0.984
## Temp_mean    -0.984  0.943
## Angl_mn_15m -0.432  0.278  0.523

plot((out / in.) ~ fitted(fm29b), data = tab)
FIT <- lm(tab$pcflight ~ fitted(fm29b))
abline(reg = FIT)
```

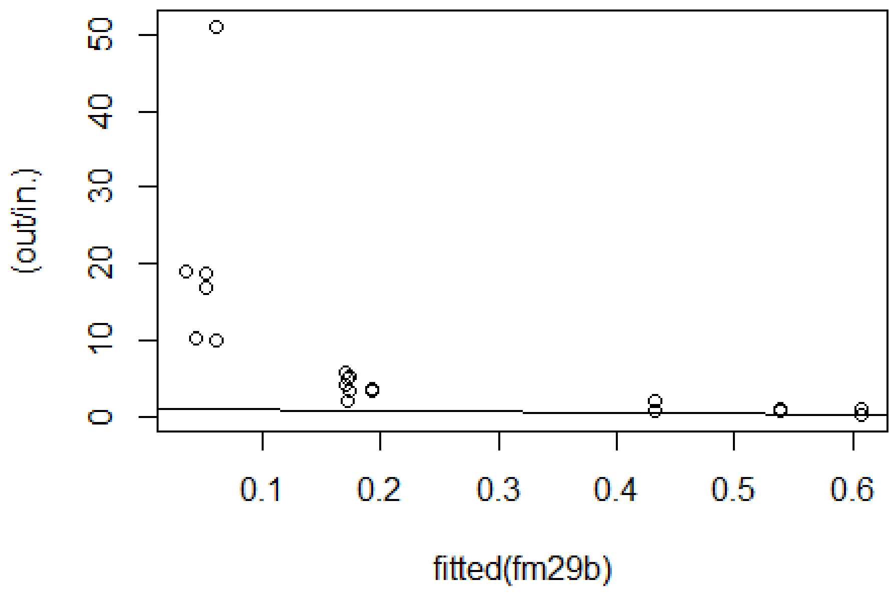

```
cor.test(tab$pcflight,fitted(fm29b))

##
##  Pearson's product-moment correlation
##
## data:  tab$pcflight and fitted(fm29b)
## t = -13.302, df = 20, p-value = 2.158e-11
## alternative hypothesis: true correlation is not equal to 0
## 95 percent confidence interval:
##  -0.9784548 -0.8765474
## sample estimates:
##        cor
## -0.9478636

summary(lm(fitted(fm29b)~(tab$out/tab$in.)))$r.squared

## [1] 0.6577943

#any(is.na(tab))

## Independent variables not correlated
```

MODELS ON THE IMPACT OF SHOCK ON THE Mating abilityOF 22 AND 29 DAY OLD PUPAE (COMBINED DATA)

Figure 9c: Mating ability

```
tab <- read.csv("Figure 9c.csv")
head(tab)

##     Date Replicate Age     Treatments unformed_pairs pairs_formed shipped
## 1 170510        R1  22 Shipped-110Gy             42            8     yes
## 2 170510        R1  22   Shipped-0Gy             40           10      no
## 3 170517        R2  22 Shipped-110Gy              8           27     yes
## 4 170517        R2  22   Shipped-0Gy              5           30      no
## 5 170524        R3  22 Shipped-110Gy             17           13     yes
## 6 170524        R3  22   Shipped-0Gy              4           26      no
##   Irradiation Events._5max Duration.ms._5max scalar_max_5max
scalar_mean_5max
## 1         yes          909            48.750          27.374
21.43900
## 2          no          909            48.750          27.374
21.43900
## 3         yes          708            87.500          27.398
16.92723
## 4          no          708            87.500          27.398
16.92723
## 5         yes         1187            93.125          27.374
15.43705
## 6          no         1187            93.125          27.374
15.43705
```

```
##   changescalar_max_5max changescalar_mean_5max Changevector_max_5max
## 1                23.724                8.94875              27.69156
## 2                23.724                8.94875              27.69156
## 3                24.477               11.37550              35.06987
## 4                24.477               11.37550              35.06987
## 5                16.627                3.83800              33.00443
## 6                16.627                3.83800              33.00443
##   Changevector_mean_5max Angle_max_5max Angle_mean_5max Hz_max_5max
## 1               9.937386       3.002116        2.617132    764.4826
## 2               9.937386       3.002116        2.617132    764.4826
## 3              19.035967       2.969360        1.679485    756.1414
## 4              19.035967       2.969360        1.679485    756.1414
## 5               5.064434       2.105922        1.356174    536.2686
## 6               5.064434       2.105922        1.356174    536.2686
##   Hz_mean_5max Events_10max Duration.ms._10max scalar_max_10max
## 1     666.4471          288             36.875           27.374
## 2     666.4471          288             36.875           27.374
## 3     427.6775          315             50.000           27.398
## 4     427.6775          315             50.000           27.398
## 5     345.3468          238             63.750           27.374
## 6     345.3468          238             63.750           27.374
##   scalar_mean_10max changescalar_max_10max changescalar_mean_10max
## 1           21.5800                 23.724                  12.473
## 2           21.5800                 23.724                  12.473
## 3           20.3750                 24.477                  16.488
## 4           20.3750                 24.477                  16.488
## 5           19.6895                 16.627                   8.965
## 6           19.6895                 16.627                   8.965
##   Changevector_max_10max Changevector_mean_10max Angle_max_10max
## 1               27.69156                15.45774        2.687392
## 2               27.69156                15.45774        2.687392
## 3               35.06987                22.58486        2.969360
## 4               35.06987                22.58486        2.969360
## 5               33.00443                10.65686        2.105922
## 6               33.00443                10.65686        2.105922
##   Angle_mean_10max Hz_max_10max Hz_mean_10max Events_15max
Duration.ms._15max
## 1        1.0470779     684.3388      266.6362          144
30.625
## 2        1.0470779     684.3388      266.6362          144
30.625
## 3        1.6138687     756.1414      410.9683          183
29.375
## 4        1.6138687     756.1414      410.9683          183
29.375
## 5        0.5851573     536.2686      149.0091          111
41.250
## 6        0.5851573     536.2686      149.0091          111
41.250
##   scalar_max_15max scalar_mean_15max changescalar_max_15max
```

```
## 1          27.374         22.42986                  23.724
## 2          27.374         22.42986                  23.724
## 3          27.398         21.71558                  24.477
## 4          27.398         21.71558                  24.477
## 5          27.374         22.77170                  16.627
## 6          27.374         22.77170                  16.627
##   changescalar_mean_15max Changevector_max_15max Changevector_mean_15max
## 1                  15.667               27.69156                19.75723
## 2                  15.667               27.69156                19.75723
## 3                  16.488               35.06987                21.61531
## 4                  16.488               35.06987                21.61531
## 5                  11.474               33.00443                12.83840
## 6                  11.474               33.00443                12.83840
##   Angle_max_15max Angle_mean_15max Hz_max_15max Hz_mean_15max Events_20max
## 1        2.687392        1.8427590     684.3388      469.2547           57
## 2        2.687392        1.8427590     684.3388      469.2547           57
## 3        2.969360        1.6010884     756.1414      407.7138           75
## 4        2.969360        1.6010884     756.1414      407.7138           75
## 5        2.105922        0.6709018     536.2686      170.8438           42
## 6        2.105922        0.6709018     536.2686      170.8438           42
##   Duration.ms._20max scalar_max_20max scalar_mean_20max
changescalar_max_20max
## 1             17.500           27.374          26.80175
23.724
## 2             17.500           27.374          26.80175
23.724
## 3             20.625           27.398          26.38567
24.477
## 4             20.625           27.398          26.38567
24.477
## 5             20.625           27.374          25.17900
13.480
## 6             20.625           27.374          25.17900
13.480
##   changescalar_mean_20max Changevector_max_20max Changevector_mean_20max
## 1                 23.7240               27.69156                27.69156
## 2                 23.7240               27.69156                27.69156
## 3                 13.7095               35.06987                29.65156
## 4                 13.7095               35.06987                29.65156
## 5                  6.6090               33.00443                11.19072
## 6                  6.6090               33.00443                11.19072
##   Angle_max_20max Angle_mean_20max Hz_max_20max Hz_mean_20max Events_5mean
## 1        2.687392         2.687392     684.3388      684.3388          909
## 2        2.687392         2.687392     684.3388      684.3388          909
## 3        2.433775         1.792941     619.7557      456.5688          708
## 4        2.433775         1.792941     619.7557      456.5688          708
## 5        1.715913         0.489164     436.9538      124.5646         1187
## 6        1.715913         0.489164     436.9538      124.5646         1187
##   Duration.ms._5mean scalar_max_5mean scalar_mean_5mean
changescalar_max_5mean
```

```
## 1          5.215209        9.516648         7.473747
3.755884
## 2          5.215209        9.516648         7.473747
3.755884
## 3         11.785840       10.551797         7.565272
2.816095
## 4         11.785840       10.551797         7.565272
2.816095
## 5          7.348884        8.022198         6.506753
1.632164
## 6          7.348884        8.022198         6.506753
1.632164
##   changescalar_mean_5mean Changevector_max_5mean Changevector_mean_5mean
## 1               1.6630629               4.501180                2.219564
## 2               1.6630629               4.501180                2.219564
## 3               1.0076953               3.691769                1.638802
## 4               1.0076953               3.691769                1.638802
## 5               0.6804169               2.293485                1.194539
## 6               0.6804169               2.293485                1.194539
##   Angle_max_5mean Angle_mean_5mean Hz_max_5mean Hz_mean_5mean
Events._10mean
## 1       0.4296757        0.1755708    109.41603      44.70874
288
## 2       0.4296757        0.1755708    109.41603      44.70874
288
## 3       0.3715939        0.1414327     94.62562      36.01553
315
## 4       0.3715939        0.1414327     94.62562      36.01553
315
## 5       0.2539263        0.1310499     64.66179      33.37158
238
## 6       0.2539263        0.1310499     64.66179      33.37158
238
##   Duration.ms._10mean scalar_max_10mean scalar_mean_10mean
## 1            3.888889          15.49537           13.49979
## 2            3.888889          15.49537           13.49979
## 3            7.706349          16.38244           13.65682
## 4            7.706349          16.38244           13.65682
## 5            7.415966          15.56483           13.15894
## 6            7.415966          15.56483           13.15894
##   changescalar_max_10mean changescalar_mean_10mean Changevector_max_10mean
## 1                6.557340                 3.249119                8.029862
## 2                6.557340                 3.249119                8.029862
## 3                4.928854                 1.971893                6.505797
## 4                4.928854                 1.971893                6.505797
## 5                4.004025                 1.664277                5.891075
## 6                4.004025                 1.664277                5.891075
##   Changevector_mean_10mean Angle_max_10mean Angle_mean_10mean
Hz_max_10mean
## 1                 4.538349        0.4719538         0.2224823
```

```
120.1821
## 2              4.538349       0.4719538         0.2224823
120.1821
## 3              3.339135       0.4047630         0.1751144
103.0721
## 4              3.339135       0.4047630         0.1751144
103.0721
## 5              2.909783       0.3492638         0.1583697
88.9393
## 6              2.909783       0.3492638         0.1583697
88.9393
##   Hz_mean_10mean Events_15mean Duration.ms._15mean scalar_max_15mean
## 1       56.65466           144            3.441840          19.26462
## 2       56.65466           144            3.441840          19.26462
## 3       44.59251           183            6.342213          19.93954
## 4       44.59251           183            6.342213          19.93954
## 5       40.32851           111            6.920045          19.71768
## 6       40.32851           111            6.920045          19.71768
##   scalar_mean_15mean changescalar_max_15mean changescalar_mean_15mean
## 1           17.94158                8.195410                 4.209463
## 2           17.94158                8.195410                 4.209463
## 3           17.79343                6.271208                 2.424063
## 4           17.79343                6.271208                 2.424063
## 5           17.60073                5.291784                 2.055466
## 6           17.60073                5.291784                 2.055466
##   Changevector_max_15mean Changevector_mean_15mean Angle_max_15mean
## 1               10.145769                 6.194357        0.4732660
## 2               10.145769                 6.194357        0.4732660
## 3                8.773143                 4.485467        0.4584332
## 4                8.773143                 4.485467        0.4584332
## 5                8.644509                 4.178605        0.4437400
## 6                8.644509                 4.178605        0.4437400
##   Angle_mean_15mean Hz_max_15mean Hz_mean_15mean Events_20mean
## 1         0.2644405      120.5162       67.33922            57
## 2         0.2644405      120.5162       67.33922            57
## 3         0.2042285      116.7390       52.00635            75
## 4         0.2042285      116.7390       52.00635            75
## 5         0.1960600      112.9975       49.92626            42
## 6         0.1960600      112.9975       49.92626            42
##   Duration.ms._20mean scalar_max_20mean scalar_mean_20mean
## 1            2.587719          22.76440           22.04857
## 2            2.587719          22.76440           22.04857
## 3            5.250000          23.71752           22.29175
## 4            5.250000          23.71752           22.29175
## 5            5.997024          24.28693           22.58697
## 6            5.997024          24.28693           22.58697
##   changescalar_max_20mean changescalar_mean_20mean Changevector_max_20mean
## 1                7.502070                 3.960703                11.35123
## 2                7.502070                 3.960703                11.35123
## 3                6.190293                 2.676377                11.36833
```

```
## 4               6.190293                 2.676377                11.36833
## 5               5.996190                 2.445063                11.97291
## 6               5.996190                 2.445063                11.97291
##   Changevector_mean_20mean Angle_max_20mean Angle_mean_20mean
Hz_max_20mean
## 1                6.922532        0.5201012         0.3140733
132.4427
## 2                6.922532        0.5201012         0.3140733
132.4427
## 3                5.877764        0.5214004         0.2548023
132.7735
## 4                5.877764        0.5214004         0.2548023
132.7735
## 5                5.476046        0.5391966         0.2201107
137.3053
## 6                5.476046        0.5391966         0.2201107
137.3053
##   Hz_mean_20mean RH._Max Temp_Max RH._mean Temp_mean
## 1       79.97812   47.08    23.50    41.69     19.73
## 2       79.97812   60.14    27.50    41.69     19.73
## 3       64.88487   62.61    29.75    53.55     21.79
## 4       64.88487   68.10    32.00    53.55     21.79
## 5       56.05073   57.37    28.69    53.25     23.29
## 6       56.05073   74.59    19.25    53.25     23.29
```

```
summary(tab)
```

```
##       Date         Replicate              Age         Treatments
##  Min.   :100801   Length:22          Min.   :22.00   Length:22
##  1st Qu.:170519   Class :character   1st Qu.:22.00   Class :character
##  Median :170607   Mode  :character   Median :29.00   Mode  :character
##  Mean   :168808                      Mean   :25.82
##  3rd Qu.:180690                      3rd Qu.:29.00
##  Max.   :180725                      Max.   :29.00
##  unformed_pairs   pairs_formed     shipped          Irradiation
##  Min.   : 1.00   Min.   : 4.00   Length:22          Length:22
##  1st Qu.: 4.00   1st Qu.:16.00   Class :character   Class :character
##  Median : 6.00   Median :21.00   Mode  :character   Mode  :character
##  Mean   :10.50   Mean   :20.41
##  3rd Qu.:12.75   3rd Qu.:26.00
##  Max.   :42.00   Max.   :39.00
##   Events._5max    Duration.ms._5max scalar_max_5max scalar_mean_5max
##  Min.   : 341.0   Min.   : 41.88    Min.   :27.28   Min.   :15.44
##  1st Qu.: 495.0   1st Qu.: 49.06    1st Qu.:27.37   1st Qu.:17.64
##  Median : 708.0   Median : 59.38    Median :27.37   Median :19.35
##  Mean   : 763.6   Mean   : 76.76    Mean   :27.37   Mean   :20.67
##  3rd Qu.:1117.5   3rd Qu.: 97.34    3rd Qu.:27.40   3rd Qu.:23.95
##  Max.   :1228.0   Max.   :150.00    Max.   :27.40   Max.   :27.13
##  changescalar_max_5max changescalar_mean_5max Changevector_max_5max
##  Min.   :15.58         Min.   : 3.838         Min.   :23.56
```

```
##  1st Qu.:21.16         1st Qu.:10.623         1st Qu.:29.49
##  Median :23.03         Median :15.391         Median :35.07
##  Mean   :22.10         Mean   :15.724         Mean   :34.55
##  3rd Qu.:24.29         3rd Qu.:23.124         3rd Qu.:38.57
##  Max.   :26.65         Max.   :26.175         Max.   :43.01
##  Changevector_mean_5max Angle_max_5max  Angle_mean_5max  Hz_max_5max
##  Min.   : 5.064         Min.   :2.106   Min.   :1.356   Min.   :536.3
##  1st Qu.:11.063         1st Qu.:2.505   1st Qu.:1.682   1st Qu.:637.8
##  Median :19.036         Median :2.969   Median :2.527   Median :756.1
##  Mean   :17.786         Mean   :2.795   Mean   :2.304   Mean   :711.8
##  3rd Qu.:23.722         3rd Qu.:3.023   3rd Qu.:2.689   3rd Qu.:769.9
##  Max.   :28.208         Max.   :3.132   Max.   :3.057   Max.   :797.5
##   Hz_mean_5max    Events_10max   Duration.ms._10max scalar_max_10max
##  Min.   :345.3   Min.   :109.0   Min.   :23.75      Min.   :27.28
##  1st Qu.:428.4   1st Qu.:144.0   1st Qu.:34.69      1st Qu.:27.37
##  Median :643.4   Median :238.0   Median :37.50      Median :27.37
##  Mean   :586.8   Mean   :285.4   Mean   :41.36      Mean   :27.37
##  3rd Qu.:684.9   3rd Qu.:308.2   3rd Qu.:50.94      3rd Qu.:27.40
##  Max.   :778.4   Max.   :956.0   Max.   :63.75      Max.   :27.40
##  scalar_mean_10max changescalar_max_10max changescalar_mean_10max
##  Min.   :17.45     Min.   :15.58          Min.   : 8.965
##  1st Qu.:20.45     1st Qu.:17.76          1st Qu.:11.337
##  Median :21.55     Median :21.91          Median :16.488
##  Mean   :22.17     Mean   :21.09          Mean   :15.891
##  3rd Qu.:23.99     3rd Qu.:23.69          3rd Qu.:20.581
##  Max.   :27.37     Max.   :25.20          Max.   :25.203
##  Changevector_max_10max Changevector_mean_10max Angle_max_10max
##  Min.   :23.56          Min.   :10.66           Min.   :1.734
##  1st Qu.:26.93          1st Qu.:12.98           1st Qu.:2.303
##  Median :33.00          Median :19.71           Median :2.570
##  Mean   :33.10          Mean   :18.75           Mean   :2.570
##  3rd Qu.:38.57          3rd Qu.:24.61           3rd Qu.:3.007
##  Max.   :43.01          Max.   :26.96           Max.   :3.057
##  Angle_mean_10max  Hz_max_10max   Hz_mean_10max    Events_15max
##  Min.   :0.5721   Min.   :441.5   Min.   :145.7   Min.   : 54.00
##  1st Qu.:1.1168   1st Qu.:586.4   1st Qu.:284.4   1st Qu.: 62.75
##  Median :1.6457   Median :654.4   Median :419.1   Median :129.00
##  Mean   :1.7194   Mean   :654.4   Mean   :437.8   Mean   :159.45
##  3rd Qu.:2.4297   3rd Qu.:765.7   3rd Qu.:618.7   3rd Qu.:161.00
##  Max.   :3.0198   Max.   :778.5   Max.   :769.0   Max.   :627.00
##  Duration.ms._15max scalar_max_15max scalar_mean_15max
changescalar_max_15max
##  Min.   :18.75      Min.   :27.28    Min.   :21.72     Min.   :15.58
##  1st Qu.:20.16      1st Qu.:27.37    1st Qu.:22.52     1st Qu.:21.16
##  Median :22.50      Median :27.37    Median :25.17     Median :23.03
##  Mean   :26.82      Mean   :27.37    Mean   :24.79     Mean   :22.10
##  3rd Qu.:30.62      3rd Qu.:27.40    3rd Qu.:27.06     3rd Qu.:24.29
##  Max.   :41.25      Max.   :27.40    Max.   :27.37     Max.   :26.65
##  changescalar_mean_15max Changevector_max_15max Changevector_mean_15max
##  Min.   : 9.943          Min.   :23.56          Min.   :12.84
```

```
##  1st Qu.:12.522         1st Qu.:26.93          1st Qu.:14.73  
##  Median :16.540         Median :35.07          Median :21.62  
##  Mean   :18.188         Mean   :34.48          Mean   :21.59  
##  3rd Qu.:23.193         3rd Qu.:40.88          3rd Qu.:26.86  
##  Max.   :26.513         Max.   :44.89          Max.   :30.34  
##  Angle_max_15max Angle_mean_15max  Hz_max_15max   Hz_mean_15max  
##  Min.   :1.326   Min.   :0.6202   Min.   :337.7   Min.   :157.9  
##  1st Qu.:2.189   1st Qu.:0.8599   1st Qu.:557.4   1st Qu.:219.0  
##  Median :2.687   Median :1.8428   Median :684.3   Median :469.3  
##  Mean   :2.484   Mean   :1.8436   Mean   :632.5   Mean   :469.5  
##  3rd Qu.:2.942   3rd Qu.:2.7354   3rd Qu.:749.1   3rd Qu.:696.6  
##  Max.   :3.125   Max.   :3.1249   Max.   :795.7   Max.   :795.7  
##   Events_20max   Duration.ms._20max scalar_max_20max scalar_mean_20max
##  Min.   : 23.0   Min.   :10.62      Min.   :27.28    Min.   :24.72    
##  1st Qu.: 29.5   1st Qu.:13.44      1st Qu.:27.37    1st Qu.:25.87    
##  Median : 57.0   Median :16.88      Median :27.37    Median :26.83    
##  Mean   : 67.0   Mean   :16.88      Mean   :27.37    Mean   :26.55    
##  3rd Qu.: 74.0   3rd Qu.:19.84      3rd Qu.:27.40    3rd Qu.:27.37    
##  Max.   :215.0   Max.   :28.12      Max.   :27.40    Max.   :27.37    
##  changescalar_max_20max changescalar_mean_20max Changevector_max_20max
##  Min.   :13.48          Min.   : 5.401          Min.   :23.56         
##  1st Qu.:21.34          1st Qu.:15.570          1st Qu.:27.87         
##  Median :23.03          Median :22.061          Median :33.00         
##  Mean   :22.23          Mean   :19.313          Mean   :33.31         
##  3rd Qu.:24.29          3rd Qu.:23.686          3rd Qu.:38.32         
##  Max.   :26.65          Max.   :26.513          Max.   :44.89         
##  Changevector_mean_20max Angle_max_20max Angle_mean_20max  Hz_max_20max   
##  Min.   :11.19           Min.   :1.326   Min.   :0.4892   Min.   :  3.057  
##  1st Qu.:25.33           1st Qu.:1.844   1st Qu.:1.4039   1st Qu.:439.219  
##  Median :27.68           Median :2.457   Median :2.2315   Median :619.756  
##  Mean   :26.20           Mean   :2.357   Mean   :2.0118   Mean   :529.617  
##  3rd Qu.:28.17           3rd Qu.:2.800   3rd Qu.:2.6834   3rd Qu.:670.696  
##  Max.   :34.48           Max.   :3.062   Max.   :2.9219   Max.   :779.845  
##  Hz_mean_20max      Events_5mean    Duration.ms._5mean scalar_max_5mean
##  Min.   :  2.671   Min.   : 341.0   Min.   : 5.215     Min.   : 7.866  
##  1st Qu.:311.298   1st Qu.: 495.0   1st Qu.: 6.909     1st Qu.: 9.178  
##  Median :456.569   Median : 708.0   Median : 8.597     Median : 9.648  
##  Mean   :450.696   Mean   : 763.6   Mean   : 8.653     Mean   : 9.743  
##  3rd Qu.:662.651   3rd Qu.:1117.5   3rd Qu.:10.250     3rd Qu.:10.434  
##  Max.   :744.045   Max.   :1228.0   Max.   :11.786     Max.   :12.092  
##  scalar_mean_5mean changescalar_max_5mean changescalar_mean_5mean
##  Min.   :6.507     Min.   :1.442          Min.   :0.6227         
##  1st Qu.:7.410     1st Qu.:2.339          1st Qu.:0.8770         
##  Median :7.565     Median :2.816          Median :1.1437         
##  Mean   :7.438     Mean   :3.378          Mean   :1.6777         
##  3rd Qu.:7.682     3rd Qu.:3.632          3rd Qu.:1.6224         
##  Max.   :8.188     Max.   :8.696          Max.   :4.5129         
##  Changevector_max_5mean Changevector_mean_5mean Angle_max_5mean 
##  Min.   : 1.767         Min.   :0.8316          Min.   :0.1659  
##  1st Qu.: 2.939         1st Qu.:1.3240          1st Qu.:0.2672  
```

```
##  Median : 3.692        Median :1.6388         Median :0.3716
##  Mean   : 4.463        Mean   :2.3524         Mean   :0.5221
##  3rd Qu.: 4.665        3rd Qu.:2.1882         3rd Qu.:0.4295
##  Max.   :12.182        Max.   :6.4512         Max.   :1.7833
##  Angle_mean_5mean   Hz_max_5mean    Hz_mean_5mean    Events._10mean
##  Min.   :0.07985   Min.   : 42.24   Min.   : 20.33   Min.   :109.0
##  1st Qu.:0.13365   1st Qu.: 68.03   1st Qu.: 34.03   1st Qu.:144.0
##  Median :0.16791   Median : 94.63   Median : 42.76   Median :238.0
##  Mean   :0.28474   Mean   :132.96   Mean   : 72.51   Mean   :285.4
##  3rd Qu.:0.21880   3rd Qu.:109.37   3rd Qu.: 55.72   3rd Qu.:308.2
##  Max.   :0.94024   Max.   :454.12   Max.   :239.43   Max.   :956.0
##  Duration.ms._10mean scalar_max_10mean scalar_mean_10mean
##  Min.   :3.532       Min.   :14.94     Min.   :12.92
##  1st Qu.:5.091       1st Qu.:15.57     1st Qu.:13.38
##  Median :7.046       Median :15.75     Median :13.66
##  Mean   :6.549       Mean   :16.07     Mean   :13.75
##  3rd Qu.:7.710       3rd Qu.:16.60     3rd Qu.:14.10
##  Max.   :8.695       Max.   :17.47     Max.   :14.79
##  changescalar_max_10mean changescalar_mean_10mean Changevector_max_10mean
##  Min.   : 3.380          Min.   :1.479            Min.   : 4.344
##  1st Qu.: 4.173          1st Qu.:1.897            1st Qu.: 6.000
##  Median : 4.929          Median :2.288            Median : 6.506
##  Mean   : 5.991          Mean   :3.423            Mean   : 8.003
##  3rd Qu.: 6.468          3rd Qu.:3.775            3rd Qu.: 8.712
##  Max.   :12.085          Max.   :9.086            Max.   :16.709
##  Changevector_mean_10mean Angle_max_10mean Angle_mean_10mean Hz_max_10mean
##  Min.   : 2.170           Min.   :0.2465   Min.   :0.1225    Min.   :
62.78
##  1st Qu.: 2.923           1st Qu.:0.3279   1st Qu.:0.1414    1st Qu.:
83.50
##  Median : 3.339           Median :0.4504   Median :0.2215    Median
:114.68
##  Mean   : 4.881           Mean   :0.5915   Mean   :0.3678    Mean
:150.62
##  3rd Qu.: 5.319           3rd Qu.:0.5606   3rd Qu.:0.3422    3rd
Qu.:142.76
##  Max.   :13.024           Max.   :1.8310   Max.   :1.3785    Max.
:466.26
##  Hz_mean_10mean   Events_15mean    Duration.ms._15mean scalar_max_15mean
##  Min.   : 31.20   Min.   : 54.00   Min.   :2.310       Min.   :19.16
##  1st Qu.: 36.02   1st Qu.: 62.75   1st Qu.:4.485       1st Qu.:19.57
##  Median : 56.39   Median :129.00   Median :6.215       Median :19.78
##  Mean   : 93.65   Mean   :159.45   Mean   :5.518       Mean   :19.97
##  3rd Qu.: 87.15   3rd Qu.:161.00   3rd Qu.:6.617       3rd Qu.:20.47
##  Max.   :351.03   Max.   :627.00   Max.   :6.920       Max.   :21.17
##  scalar_mean_15mean changescalar_max_15mean changescalar_mean_15mean
##  Min.   :17.60      Min.   : 4.622          Min.   : 1.704
##  1st Qu.:17.83      1st Qu.: 5.493          1st Qu.: 2.300
##  Median :18.27      Median : 6.393          Median : 2.959
##  Mean   :18.18      Mean   : 7.567          Mean   : 4.400
```

```
##  3rd Qu.:18.53      3rd Qu.: 8.247         3rd Qu.: 5.211
##  Max.   :18.71      Max.   :14.038         Max.   :11.493
##  Changevector_max_15mean Changevector_mean_15mean Angle_max_15mean
##  Min.   : 6.224          Min.   : 2.939           Min.   :0.2772
##  1st Qu.: 8.675          1st Qu.: 4.075           1st Qu.:0.3934
##  Median : 9.016          Median : 4.903           Median :0.4733
##  Mean   :10.393          Mean   : 6.549           Mean   :0.6328
##  3rd Qu.:11.444          3rd Qu.: 7.425           3rd Qu.:0.6286
##  Max.   :19.029          Max.   :16.285           Max.   :1.7973
##  Angle_mean_15mean Hz_max_15mean    Hz_mean_15mean   Events_20mean
##  Min.   :0.1236    Min.   : 70.58   Min.   : 31.48   Min.   : 23.0
##  1st Qu.:0.1790    1st Qu.:100.19   1st Qu.: 45.59   1st Qu.: 29.5
##  Median :0.2644    Median :120.52   Median : 67.34   Median : 57.0
##  Mean   :0.4088    Mean   :161.14   Mean   :104.11   Mean   : 67.0
##  3rd Qu.:0.4098    3rd Qu.:160.08   3rd Qu.:104.37   3rd Qu.: 74.0
##  Max.   :1.4887    Max.   :457.68   Max.   :379.09   Max.   :215.0
##  Duration.ms._20mean scalar_max_20mean scalar_mean_20mean
##  Min.   :1.663       Min.   :22.50     Min.   :21.81
##  1st Qu.:3.989       1st Qu.:22.84     1st Qu.:22.17
##  Median :4.344       Median :23.45     Median :22.25
##  Mean   :4.264       Mean   :23.43     Mean   :22.31
##  3rd Qu.:5.133       3rd Qu.:23.99     3rd Qu.:22.53
##  Max.   :5.997       Max.   :24.29     Max.   :22.86
##  changescalar_max_20mean changescalar_mean_20mean Changevector_max_20mean
##  Min.   : 5.234          Min.   : 2.159           Min.   : 7.482
##  1st Qu.: 5.531          1st Qu.: 2.780           1st Qu.: 9.975
##  Median : 7.212          Median : 3.869           Median :11.368
##  Mean   : 7.824          Mean   : 4.883           Mean   :12.189
##  3rd Qu.: 8.123          3rd Qu.: 5.590           3rd Qu.:12.832
##  Max.   :14.139          Max.   :12.105           Max.   :19.387
##  Changevector_mean_20mean Angle_max_20mean Angle_mean_20mean Hz_max_20mean
##  Min.   : 4.828           Min.   :0.3096   Min.   :0.2178    Min.   :
0.5016
##  1st Qu.: 5.527           1st Qu.:0.4961   1st Qu.:0.2321    1st
Qu.:114.2034
##  Median : 6.843           Median :0.5214   Median :0.3023    Median
:132.7735
##  Mean   : 7.865           Mean   :0.6046   Mean   :0.3945    Mean
:142.5937
##  3rd Qu.: 8.273           3rd Qu.:0.5788   3rd Qu.:0.3688    3rd
Qu.:147.3906
##  Max.   :17.154           Max.   :1.2518   Max.   :1.0993    Max.
:318.7755
##  Hz_mean_20mean       RH._Max         Temp_Max        RH._mean
##  Min.   :  0.3079   Min.   :47.08   Min.   :19.25   Min.   :41.69
##  1st Qu.: 56.3355   1st Qu.:60.76   1st Qu.:24.53   1st Qu.:50.83
##  Median : 75.5923   Median :73.52   Median :27.38   Median :71.53
##  Mean   : 93.4190   Mean   :69.60   Mean   :26.80   Mean   :62.64
##  3rd Qu.: 93.9064   3rd Qu.:78.33   3rd Qu.:29.48   3rd Qu.:72.73
##  Max.   :279.9378   Max.   :84.59   Max.   :32.00   Max.   :77.90
```

```
##    Temp_mean
##  Min.   :10.22
##  1st Qu.:12.61
##  Median :15.36
##  Mean   :17.33
##  3rd Qu.:22.23
##  Max.   :24.58
```

```
str(tab)
```

```
## 'data.frame':    22 obs. of  108 variables:
##  $ Date                     : int  170510 170510 170517 170517 170524
170524 170531 170531 170607 170607 ...
##  $ Replicate                : chr  "R1" "R1" "R2" "R2" ...
##  $ Age                      : int  22 22 22 22 22 22 22 22 22 22 ...
##  $ Treatments               : chr  "Shipped-110Gy " "Shipped-0Gy "
"Shipped-110Gy " "Shipped-0Gy " ...
##  $ unformed_pairs           : int  42 40 8 5 17 4 16 7 21 1 ...
##  $ pairs_formed             : int  8 10 27 30 13 26 4 23 19 39 ...
##  $ shipped                  : chr  "yes" "no" "yes" "no" ...
##  $ Irradiation              : chr  "yes" "no" "yes" "no" ...
##  $ Events._5max             : int  909 909 708 708 1187 1187 625 625 606
606 ...
##  $ Duration.ms._5max        : num  48.8 48.8 87.5 87.5 93.1 ...
##  $ scalar_max_5max          : num  27.4 27.4 27.4 27.4 27.4 ...
##  $ scalar_mean_5max         : num  21.4 21.4 16.9 16.9 15.4 ...
##  $ changescalar_max_5max    : num  23.7 23.7 24.5 24.5 16.6 ...
##  $ changescalar_mean_5max   : num  8.95 8.95 11.38 11.38 3.84 ...
##  $ Changevector_max_5max    : num  27.7 27.7 35.1 35.1 33 ...
##  $ Changevector_mean_5max   : num  9.94 9.94 19.04 19.04 5.06 ...
##  $ Angle_max_5max           : num  3 3 2.97 2.97 2.11 ...
##  $ Angle_mean_5max          : num  2.62 2.62 1.68 1.68 1.36 ...
##  $ Hz_max_5max              : num  764 764 756 756 536 ...
##  $ Hz_mean_5max             : num  666 666 428 428 345 ...
##  $ Events_10max             : int  288 288 315 315 238 238 261 261 219 219
...
##  $ Duration.ms._10max       : num  36.9 36.9 50 50 63.8 ...
##  $ scalar_max_10max         : num  27.4 27.4 27.4 27.4 27.4 ...
##  $ scalar_mean_10max        : num  21.6 21.6 20.4 20.4 19.7 ...
##  $ changescalar_max_10max   : num  23.7 23.7 24.5 24.5 16.6 ...
##  $ changescalar_mean_10max  : num  12.47 12.47 16.49 16.49 8.97 ...
##  $ Changevector_max_10max   : num  27.7 27.7 35.1 35.1 33 ...
##  $ Changevector_mean_10max  : num  15.5 15.5 22.6 22.6 10.7 ...
##  $ Angle_max_10max          : num  2.69 2.69 2.97 2.97 2.11 ...
##  $ Angle_mean_10max         : num  1.047 1.047 1.614 1.614 0.585 ...
##  $ Hz_max_10max             : num  684 684 756 756 536 ...
##  $ Hz_mean_10max            : num  267 267 411 411 149 ...
##  $ Events_15max             : int  144 144 183 183 111 111 163 163 129 129
...
##  $ Duration.ms._15max       : num  30.6 30.6 29.4 29.4 41.2 ...
```

```
##  $ scalar_max_15max        : num  27.4 27.4 27.4 27.4 27.4 ...
##  $ scalar_mean_15max       : num  22.4 22.4 21.7 21.7 22.8 ...
##  $ changescalar_max_15max  : num  23.7 23.7 24.5 24.5 16.6 ...
##  $ changescalar_mean_15max : num  15.7 15.7 16.5 16.5 11.5 ...
##  $ Changevector_max_15max  : num  27.7 27.7 35.1 35.1 33 ...
##  $ Changevector_mean_15max : num  19.8 19.8 21.6 21.6 12.8 ...
##  $ Angle_max_15max         : num  2.69 2.69 2.97 2.97 2.11 ...
##  $ Angle_mean_15max        : num  1.843 1.843 1.601 1.601 0.671 ...
##  $ Hz_max_15max            : num  684 684 756 756 536 ...
##  $ Hz_mean_15max           : num  469 469 408 408 171 ...
##  $ Events_20max            : int  57 57 75 75 42 42 97 97 71 71 ...
##  $ Duration.ms._20max      : num  17.5 17.5 20.6 20.6 20.6 ...
##  $ scalar_max_20max        : num  27.4 27.4 27.4 27.4 27.4 ...
##  $ scalar_mean_20max       : num  26.8 26.8 26.4 26.4 25.2 ...
##  $ changescalar_max_20max  : num  23.7 23.7 24.5 24.5 13.5 ...
##  $ changescalar_mean_20max : num  23.72 23.72 13.71 13.71 6.61 ...
##  $ Changevector_max_20max  : num  27.7 27.7 35.1 35.1 33 ...
##  $ Changevector_mean_20max : num  27.7 27.7 29.7 29.7 11.2 ...
##  $ Angle_max_20max         : num  2.69 2.69 2.43 2.43 1.72 ...
##  $ Angle_mean_20max        : num  2.687 2.687 1.793 1.793 0.489 ...
##  $ Hz_max_20max            : num  684 684 620 620 437 ...
##  $ Hz_mean_20max           : num  684 684 457 457 125 ...
##  $ Events_5mean            : int  909 909 708 708 1187 1187 625 625 606
606 ...
##  $ Duration.ms._5mean      : num  5.22 5.22 11.79 11.79 7.35 ...
##  $ scalar_max_5mean        : num  9.52 9.52 10.55 10.55 8.02 ...
##  $ scalar_mean_5mean       : num  7.47 7.47 7.57 7.57 6.51 ...
##  $ changescalar_max_5mean  : num  3.76 3.76 2.82 2.82 1.63 ...
##  $ changescalar_mean_5mean : num  1.66 1.66 1.01 1.01 0.68 ...
##  $ Changevector_max_5mean  : num  4.5 4.5 3.69 3.69 2.29 ...
##  $ Changevector_mean_5mean : num  2.22 2.22 1.64 1.64 1.19 ...
##  $ Angle_max_5mean         : num  0.43 0.43 0.372 0.372 0.254 ...
##  $ Angle_mean_5mean        : num  0.176 0.176 0.141 0.141 0.131 ...
##  $ Hz_max_5mean            : num  109.4 109.4 94.6 94.6 64.7 ...
##  $ Hz_mean_5mean           : num  44.7 44.7 36 36 33.4 ...
##  $ Events._10mean          : int  288 288 315 315 238 238 261 261 219 219
...
##  $ Duration.ms._10mean     : num  3.89 3.89 7.71 7.71 7.42 ...
##  $ scalar_max_10mean       : num  15.5 15.5 16.4 16.4 15.6 ...
##  $ scalar_mean_10mean      : num  13.5 13.5 13.7 13.7 13.2 ...
##  $ changescalar_max_10mean : num  6.56 6.56 4.93 4.93 4 ...
##  $ changescalar_mean_10mean: num  3.25 3.25 1.97 1.97 1.66 ...
##  $ Changevector_max_10mean : num  8.03 8.03 6.51 6.51 5.89 ...
##  $ Changevector_mean_10mean: num  4.54 4.54 3.34 3.34 2.91 ...
##  $ Angle_max_10mean        : num  0.472 0.472 0.405 0.405 0.349 ...
##  $ Angle_mean_10mean       : num  0.222 0.222 0.175 0.175 0.158 ...
##  $ Hz_max_10mean           : num  120.2 120.2 103.1 103.1 88.9 ...
##  $ Hz_mean_10mean          : num  56.7 56.7 44.6 44.6 40.3 ...
##  $ Events_15mean           : int  144 144 183 183 111 111 163 163 129 129
...
```

```
## $ Duration.ms._15mean     : num  3.44 3.44 6.34 6.34 6.92 ...
## $ scalar_max_15mean       : num  19.3 19.3 19.9 19.9 19.7 ...
## $ scalar_mean_15mean      : num  17.9 17.9 17.8 17.8 17.6 ...
## $ changescalar_max_15mean : num  8.2 8.2 6.27 6.27 5.29 ...
## $ changescalar_mean_15mean: num  4.21 4.21 2.42 2.42 2.06 ...
## $ Changevector_max_15mean : num  10.15 10.15 8.77 8.77 8.64 ...
## $ Changevector_mean_15mean: num  6.19 6.19 4.49 4.49 4.18 ...
## $ Angle_max_15mean        : num  0.473 0.473 0.458 0.458 0.444 ...
## $ Angle_mean_15mean       : num  0.264 0.264 0.204 0.204 0.196 ...
## $ Hz_max_15mean           : num  121 121 117 117 113 ...
## $ Hz_mean_15mean          : num  67.3 67.3 52 52 49.9 ...
## $ Events_20mean           : int  57 57 75 75 42 42 97 97 71 71 ...
## $ Duration.ms._20mean     : num  2.59 2.59 5.25 5.25 6 ...
## $ scalar_max_20mean       : num  22.8 22.8 23.7 23.7 24.3 ...
## $ scalar_mean_20mean      : num  22 22 22.3 22.3 22.6 ...
## $ changescalar_max_20mean : num  7.5 7.5 6.19 6.19 6 ...
## $ changescalar_mean_20mean: num  3.96 3.96 2.68 2.68 2.45 ...
## $ Changevector_max_20mean : num  11.4 11.4 11.4 11.4 12 ...
##   [list output truncated]
```

```
tab$pcmating <- tab$pairs_formed / (tab$pairs_formed+tab$unformed_pairs)
boxplot(tab$pcmating ~ tab$Treatments, xlab ="Treatments", ylab = "mating
ability")
```

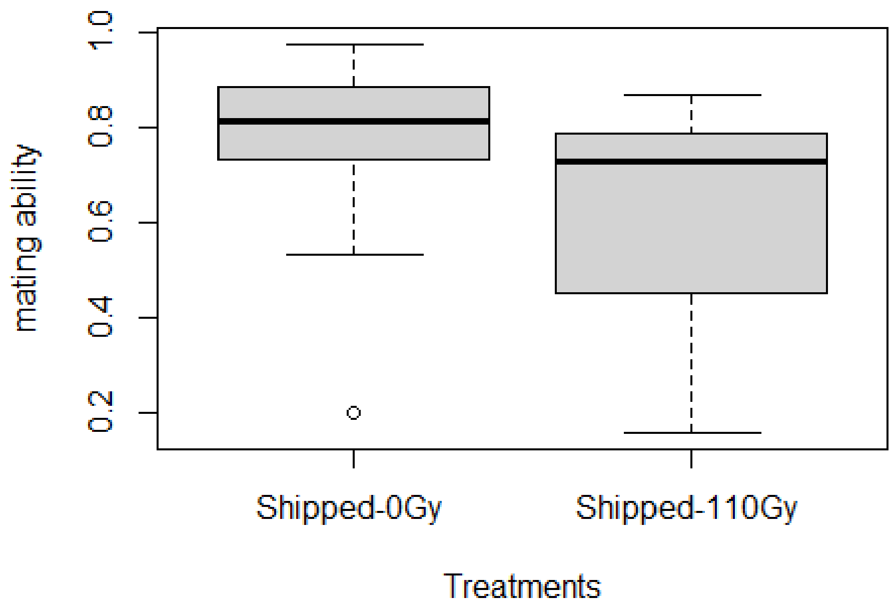


```
#####SCATTER PLOTS
plot(tab$pcmating~tab$RH._Max)#*
```

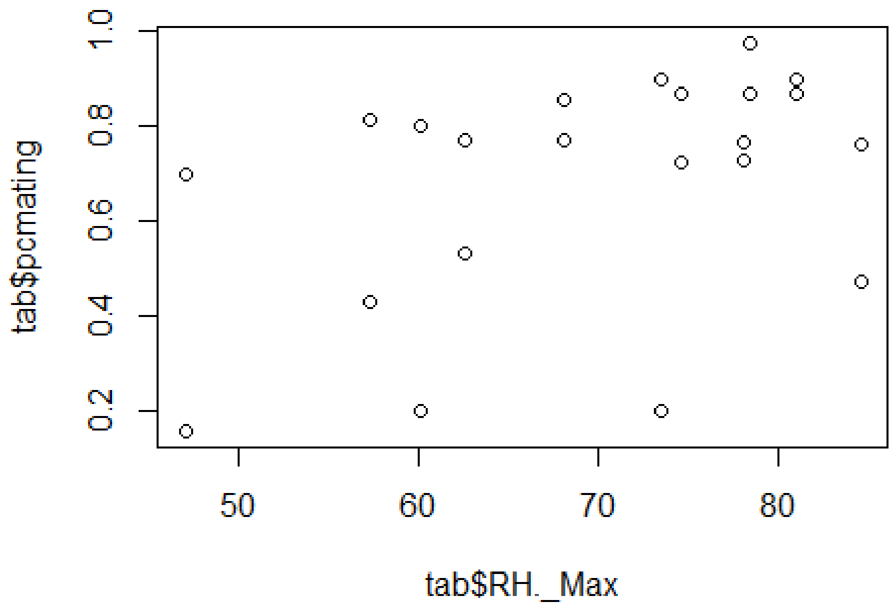


```
cor.test(tab$pcmating,tab$RH._Max)

##
##  Pearson's product-moment correlation
##
## data:  tab$pcmating and tab$RH._Max
## t = 2.0813, df = 20, p-value = 0.05047
## alternative hypothesis: true correlation is not equal to 0
## 95 percent confidence interval:
##  0.0003907999 0.7161439459
## sample estimates:
##       cor
## 0.4219297

plot(tab$pcmating~tab$RH._mean)
```

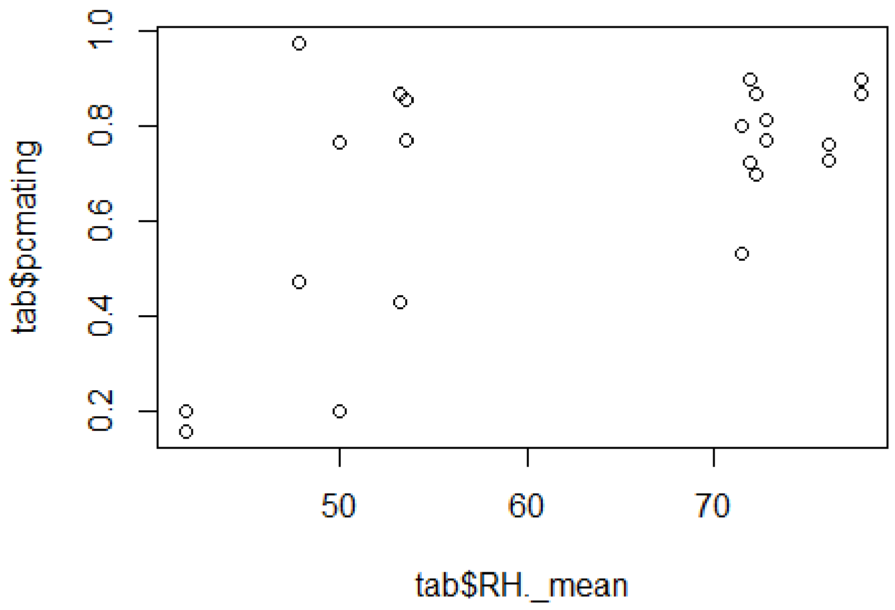


```
plot(tab$pcmating~tab$Temp_mean)#*
```

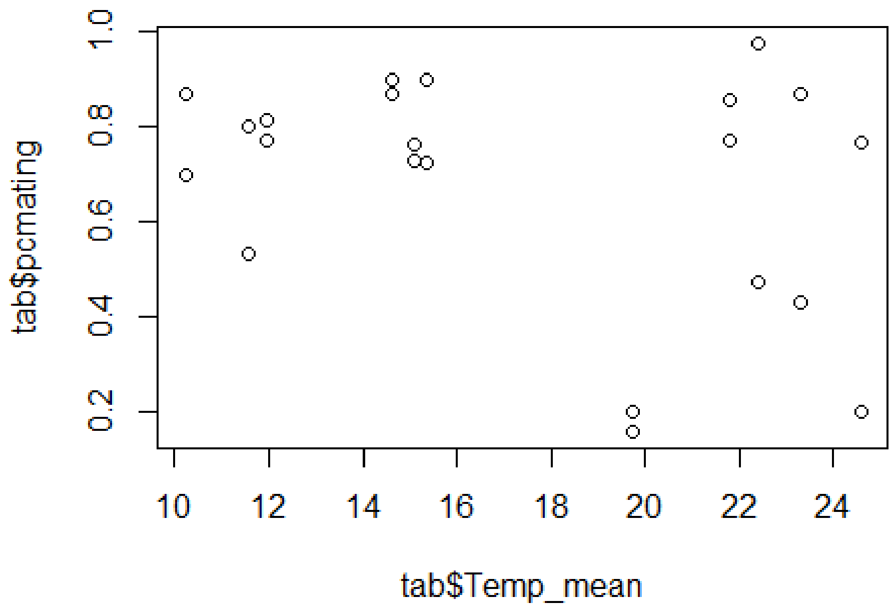


```
cor.test(tab$pcmating,tab$Temp_mean)
```

```
##
##  Pearson's product-moment correlation
##
## data:  tab$pcmating and tab$Temp_mean
## t = -1.5396, df = 20, p-value = 0.1393
## alternative hypothesis: true correlation is not equal to 0
## 95 percent confidence interval:
##  -0.6569590  0.1113844
## sample estimates:
##        cor
## -0.3255102

plot(tab$pcmating~tab$Temp_Max)
```

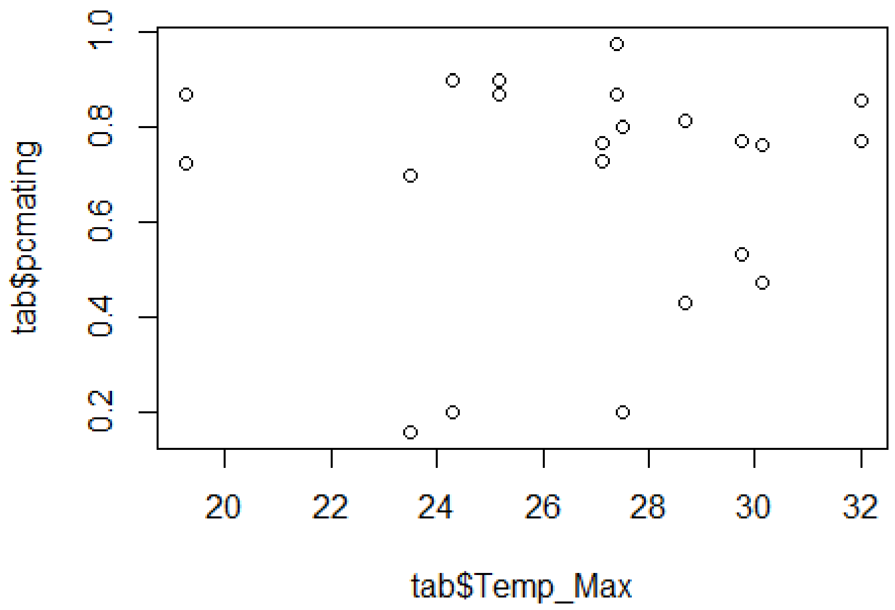


```
#Scatter plots at threshold 5-Max
par(mfrow = c(2,2))
plot(tab$pcmating~tab$Events._5max)
plot(tab$pcmating~tab$Duration.ms._5max)
plot(tab$pcmating~tab$scalar_max_5max)
plot(tab$pcmating~tab$scalar_mean_5max)
```

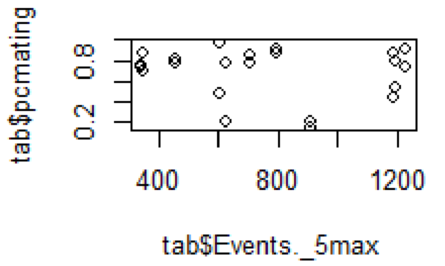

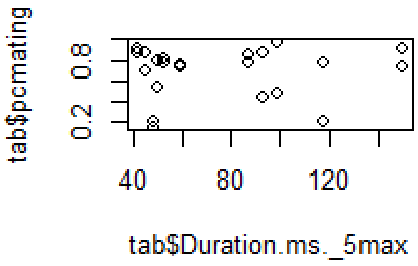

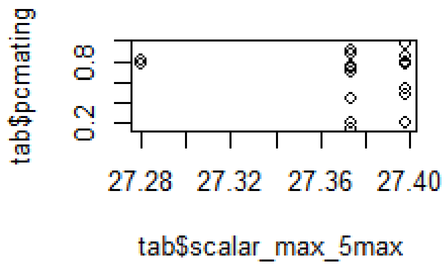

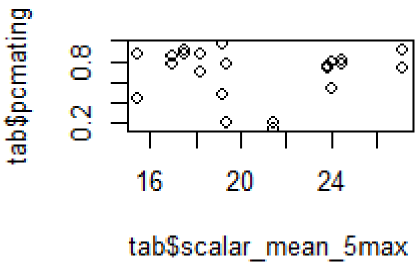


```
plot(tab$pcmating~tab$changescalar_max_5max)
plot(tab$pcmating~tab$changescalar_mean_5max)#*
cor.test(tab$pcmating,tab$changescalar_mean_5max)

##
##  Pearson's product-moment correlation
##
## data:  tab$pcmating and tab$changescalar_mean_5max
## t = 1.1194, df = 20, p-value = 0.2762
## alternative hypothesis: true correlation is not equal to 0
## 95 percent confidence interval:
##  -0.1991838  0.6027217
## sample estimates:
##       cor
## 0.2428157

plot(tab$pcmating~tab$Changevector_max_5max)
plot(tab$pcmating~tab$Changevector_mean_5max)#*
```

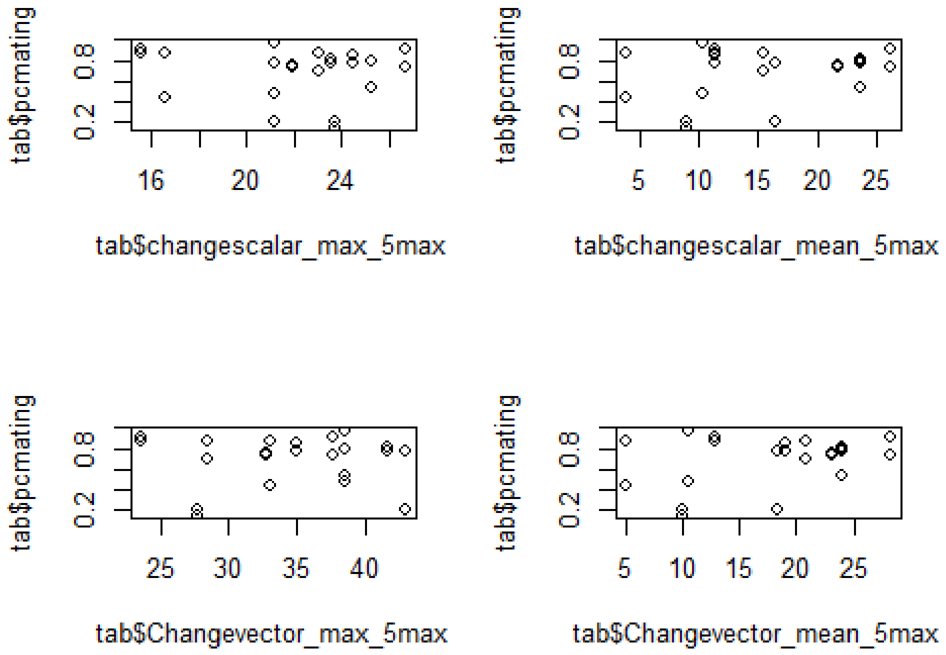


```
cor.test(tab$pcmating,tab$Changevector_mean_5max)

##
##  Pearson's product-moment correlation
##
## data:  tab$pcmating and tab$Changevector_mean_5max
## t = 1.4834, df = 20, p-value = 0.1536
## alternative hypothesis: true correlation is not equal to 0
## 95 percent confidence interval:
##  -0.1231227  0.6501408
## sample estimates:
##       cor
## 0.3148284

plot(tab$pcmating~tab$Angle_max_5max)
plot(tab$pcmating~tab$Angle_mean_5max)
plot(tab$pcmating~tab$Hz_max_5max)
plot(tab$pcmating~tab$Hz_mean_5max)
```

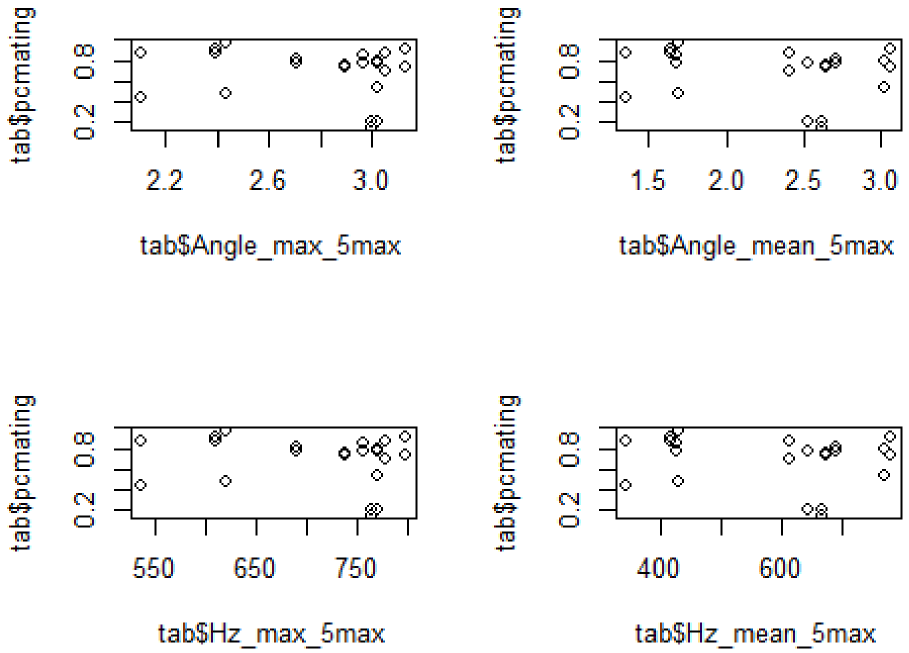

#Scatter plots at threshold 5-Mean

```
par(mfrow = c(2,2))
plot(tab$pcmating~tab$Duration.ms._5mean)#*
plot(tab$pcmating~tab$scalar_max_5mean)
plot(tab$pcmating~tab$scalar_mean_5mean)
plot(tab$pcmating~tab$changescalar_max_5mean)
```

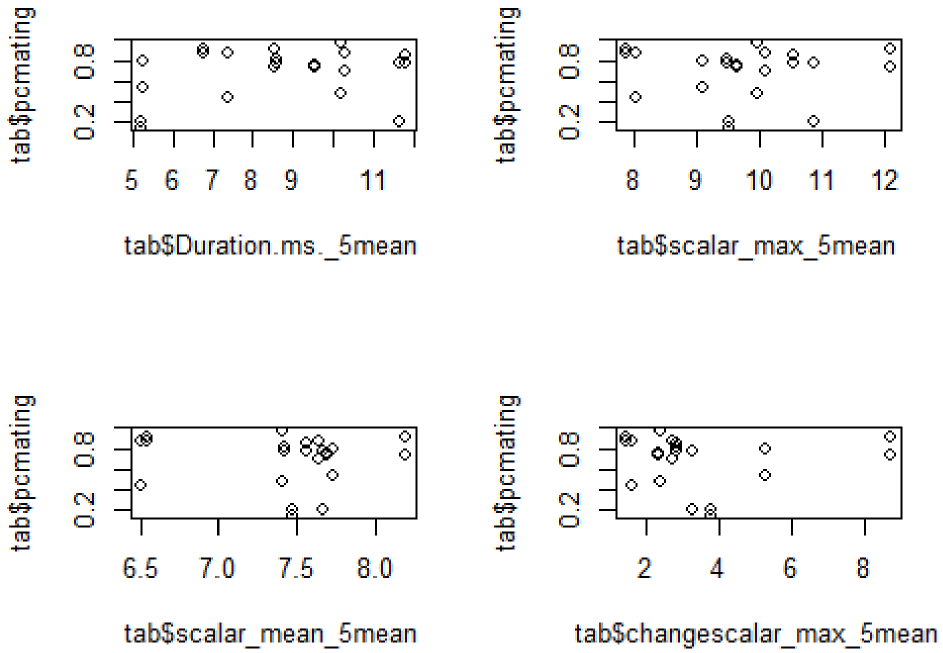


```
plot(tab$pcmating~tab$changescalar_mean_5mean)
plot(tab$pcmating~tab$Changevector_max_5mean)
plot(tab$pcmating~tab$Changevector_mean_5mean)
plot(tab$pcmating~tab$Angle_max_5mean)
```

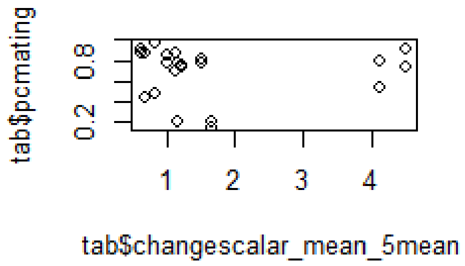

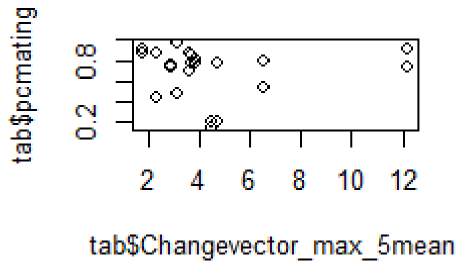

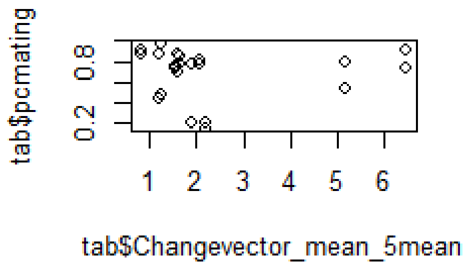

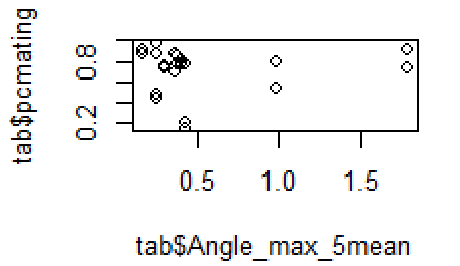


```
plot(tab$pcmating~tab$Angle_mean_5mean)
plot(tab$pcmating~tab$Hz_max_5mean)
plot(tab$pcmating~tab$Hz_mean_5mean)
```

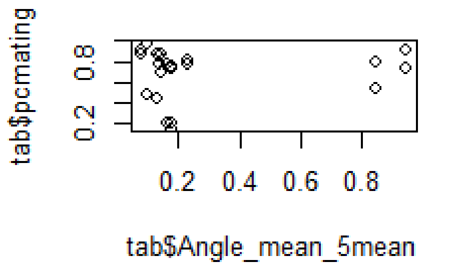

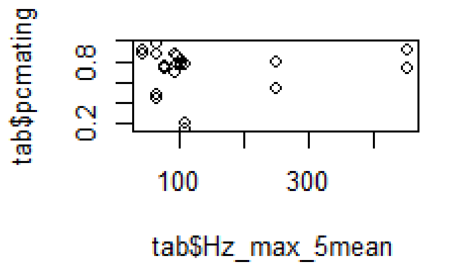

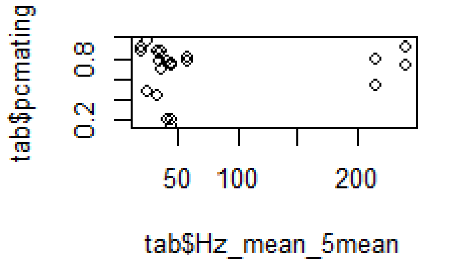

#Scatter plots at threshold 10-Max

```
par(mfrow = c(2,2))
plot(tab$pcmating~tab$Events_10max)#*
plot(tab$pcmating~tab$Duration.ms._10max)
plot(tab$pcmating~tab$scalar_max_10max)
plot(tab$pcmating~tab$scalar_mean_10max)
```

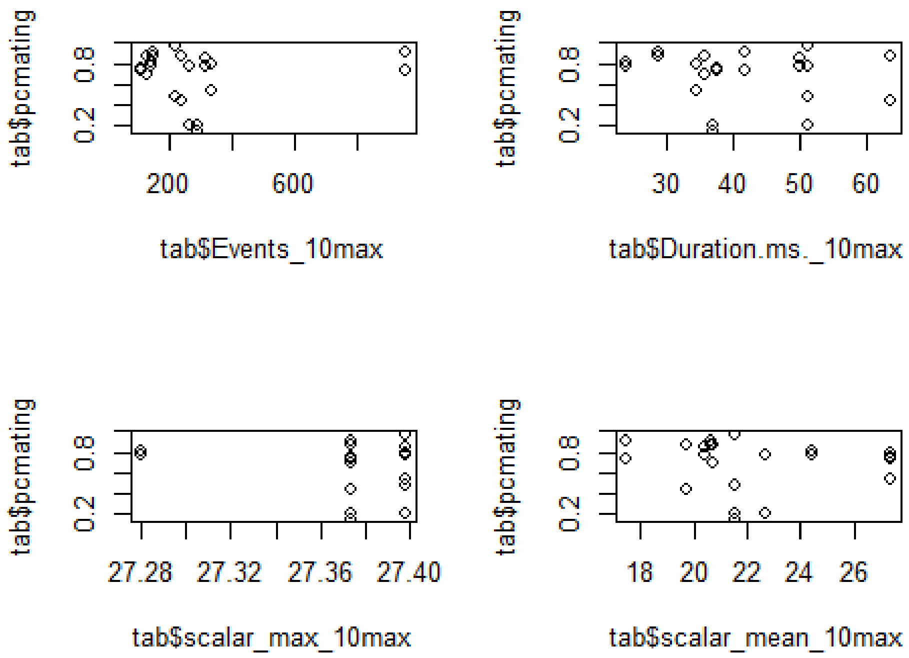


```
plot(tab$pcmating~tab$changescalar_max_10max)#*
plot(tab$pcmating~tab$changescalar_mean_10max)
plot(tab$pcmating~tab$Changevector_max_10max)
plot(tab$pcmating~tab$Changevector_mean_10max)
```

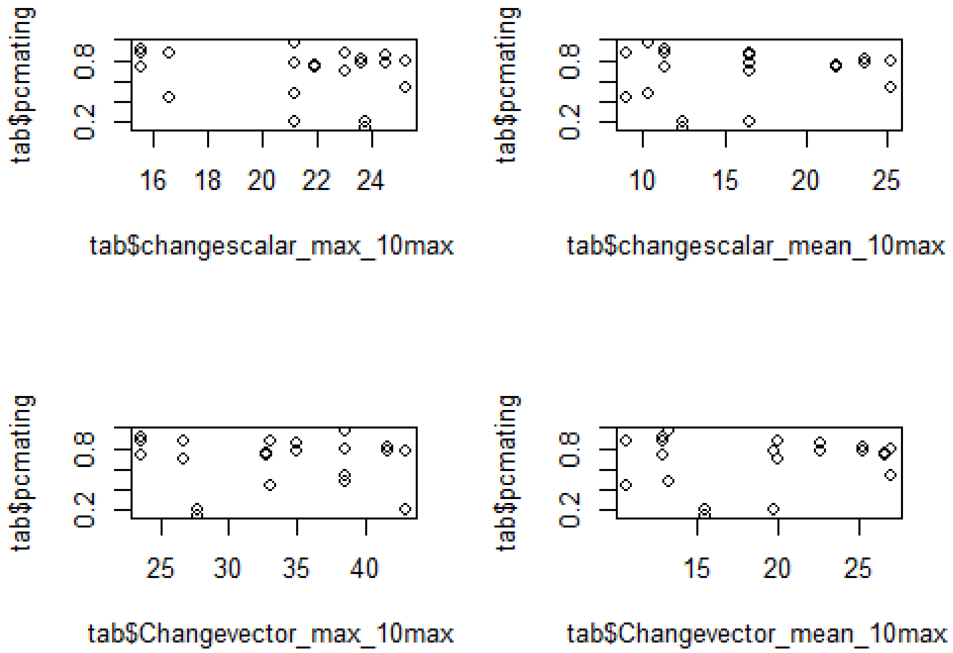


```
plot(tab$pcmating~tab$Angle_max_10max)#*
plot(tab$pcmating~tab$Angle_mean_10max)
plot(tab$pcmating~tab$Hz_max_10max)
plot(tab$pcmating~tab$Hz_mean_10max)
```

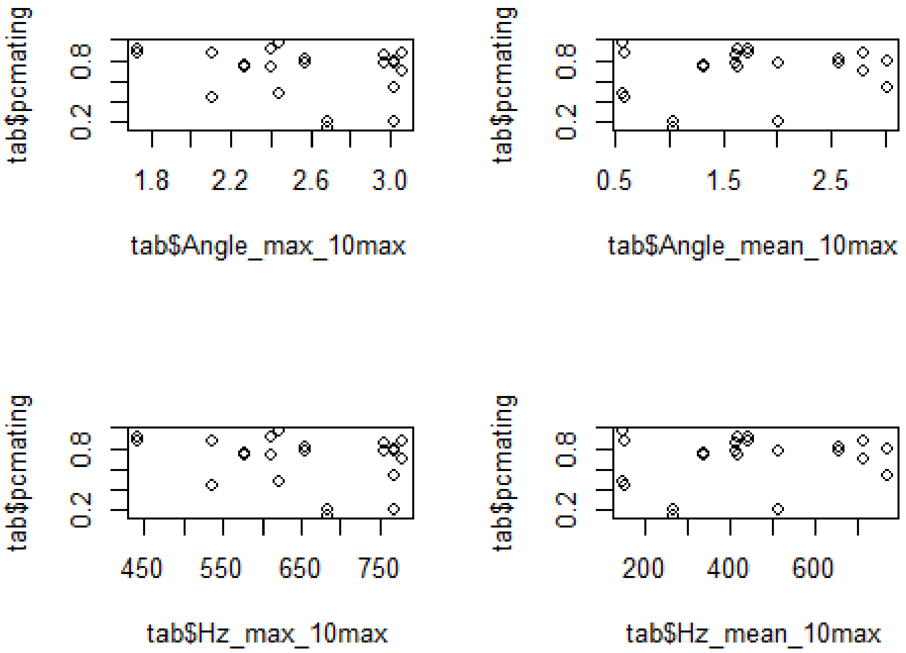

#Scatter plots at threshold 10-Mean

```
par(mfrow = c(2,2))
plot(tab$pcmating~tab$Duration.ms._10mean)
plot(tab$pcmating~tab$scalar_max_10mean)
plot(tab$pcmating~tab$scalar_mean_10mean)
plot(tab$pcmating~tab$changescalar_max_10mean)
```

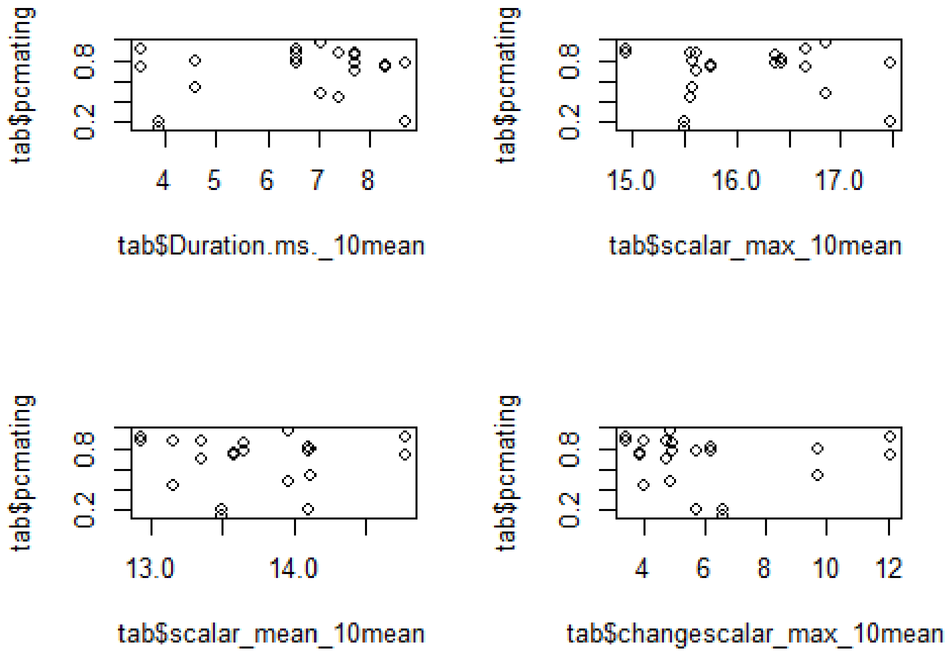


```
plot(tab$pcmating~tab$changescalar_mean_10mean)
plot(tab$pcmating~tab$Changevector_max_10mean)
plot(tab$pcmating~tab$Changevector_mean_10mean)
plot(tab$pcmating~tab$Angle_max_10mean)
```

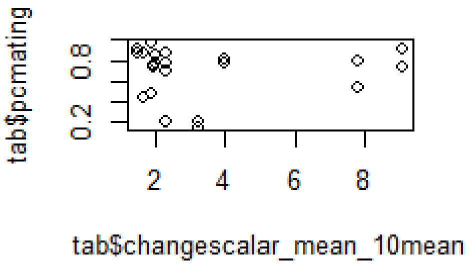

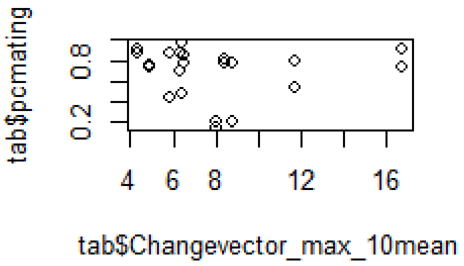

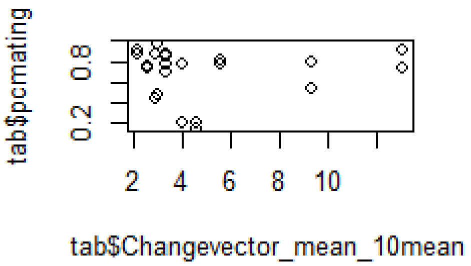

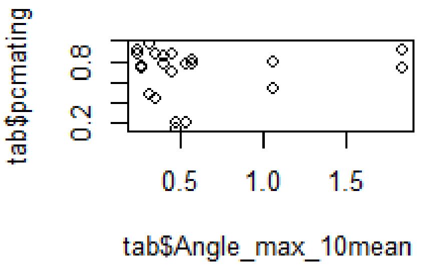


```
plot(tab$pcmating~tab$Angle_mean_10mean)
plot(tab$pcmating~tab$Hz_max_10mean)
plot(tab$pcmating~tab$Hz_mean_10mean)
```

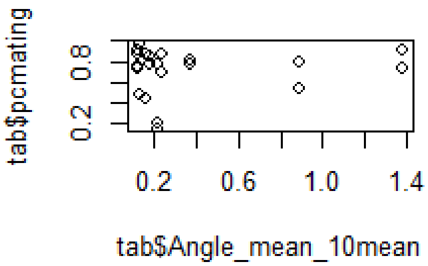

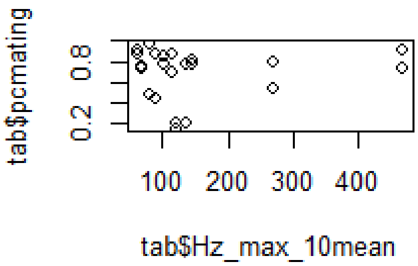

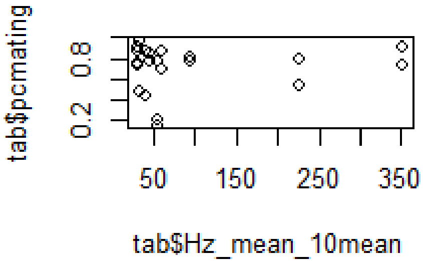

#Scatter plots at threshold 15-Max

```
par(mfrow = c(2,2))
plot(tab$pcmating~tab$Events_15max)
plot(tab$pcmating~tab$Duration.ms._15max)#*
plot(tab$pcmating~tab$scalar_max_15max)
plot(tab$pcmating~tab$scalar_mean_15max)
```

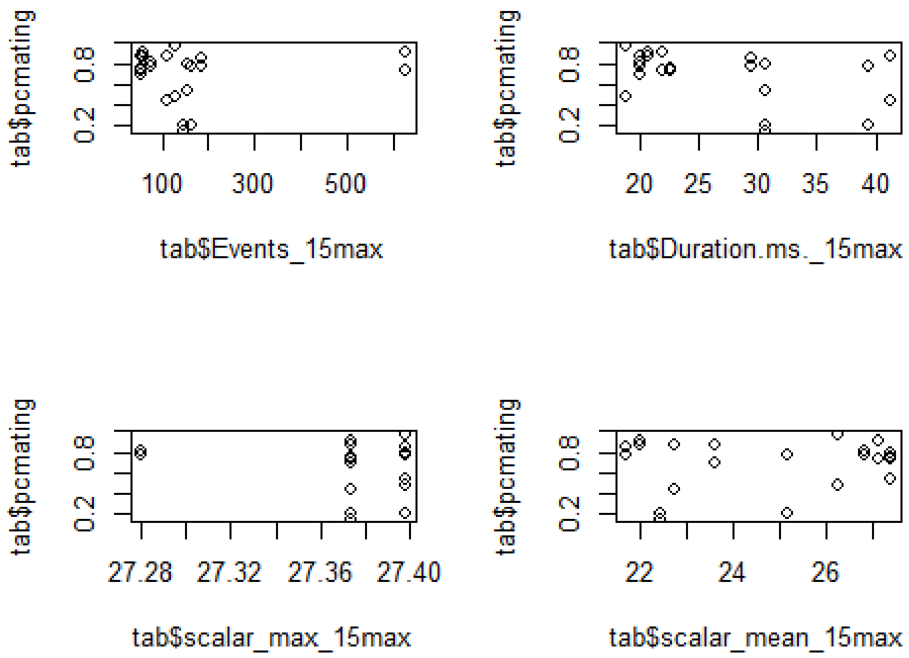


```
plot(tab$pcmating~tab$changescalar_max_15max)#*
plot(tab$pcmating~tab$changescalar_mean_15max)
plot(tab$pcmating~tab$Changevector_max_15max)
plot(tab$pcmating~tab$Changevector_mean_15max)
```

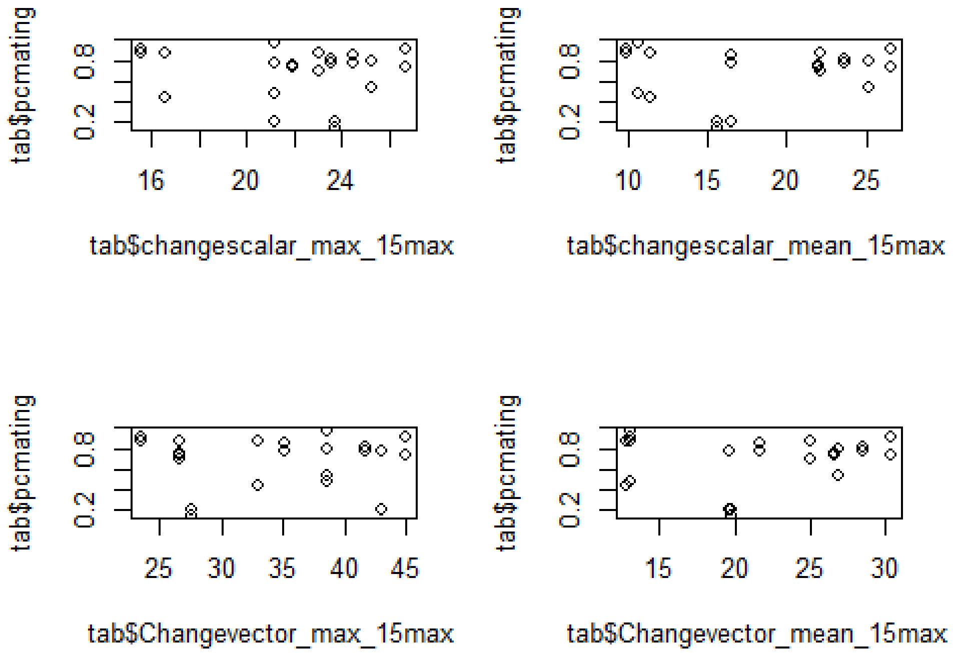


```
plot(tab$pcmating~tab$Angle_max_15max)#*
plot(tab$pcmating~tab$Angle_mean_15max)
plot(tab$pcmating~tab$Hz_max_15max)#*
plot(tab$pcmating~tab$Hz_mean_15max)
```

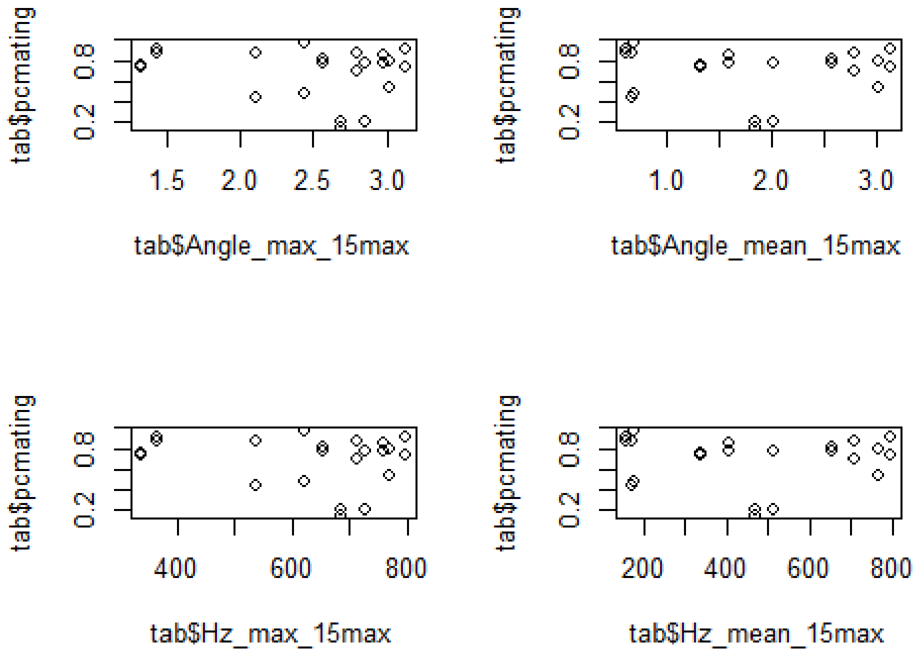

#Scatter plots at threshold 15-Mean

```
par(mfrow = c(2,2))
plot(tab$pcmating~tab$Duration.ms._15mean)#*
plot(tab$pcmating~tab$scalar_max_15mean)
plot(tab$pcmating~tab$scalar_mean_15mean)
plot(tab$pcmating~tab$changescalar_max_15mean)#*
```

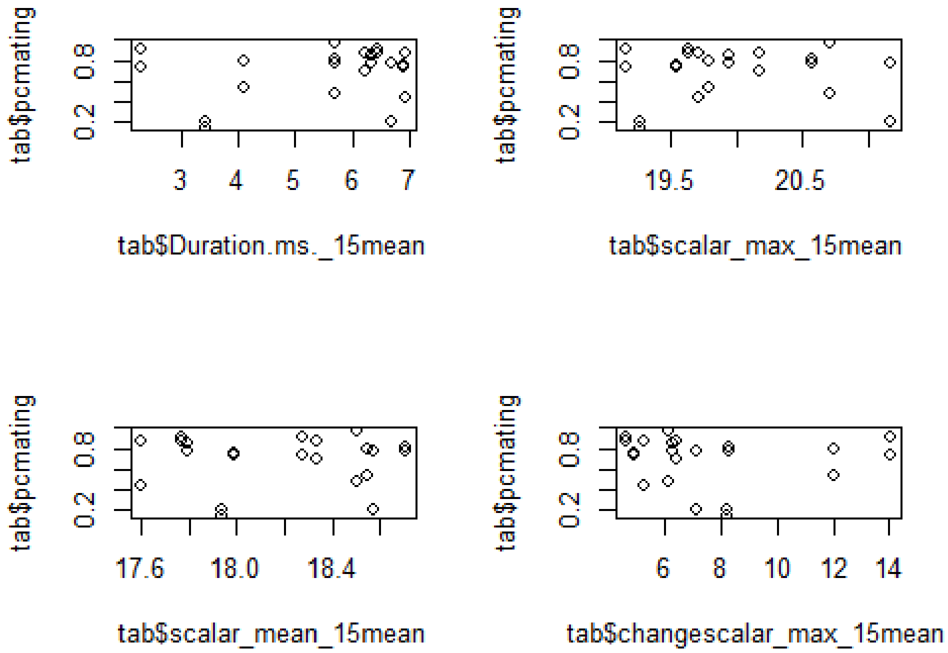


```
plot(tab$pcmating~tab$changescalar_mean_15mean)
plot(tab$pcmating~tab$Changevector_max_15mean)
plot(tab$pcmating~tab$Changevector_mean_15mean)
plot(tab$pcmating~tab$Angle_max_15mean)
```

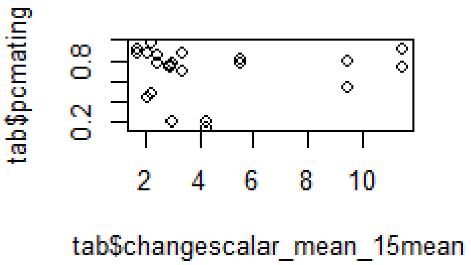

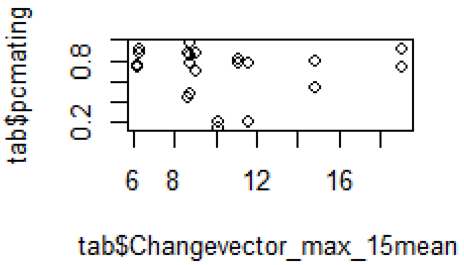

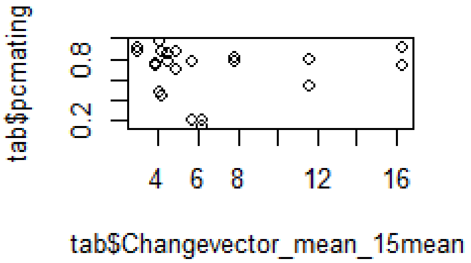

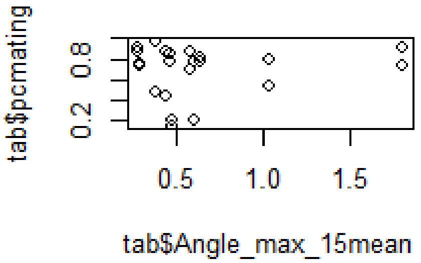


```
plot(tab$pcmating~tab$Angle_mean_15mean)
plot(tab$pcmating~tab$Hz_max_15mean)
plot(tab$pcmating~tab$Hz_mean_15mean)
```

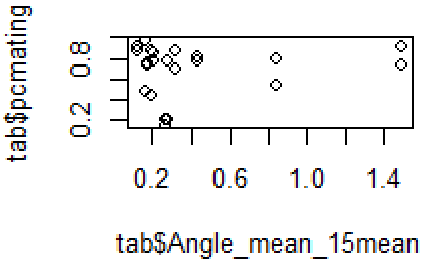

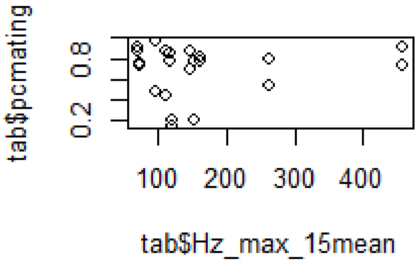

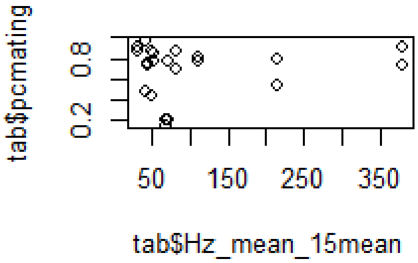

#Scatter plots at threshold 20-Max

```
par(mfrow = c(2,2))
plot(tab$pcmating~tab$Events_20max)
plot(tab$pcmating~tab$Duration.ms._20max)
plot(tab$pcmating~tab$scalar_max_20max)
plot(tab$pcmating~tab$scalar_mean_20max)
```

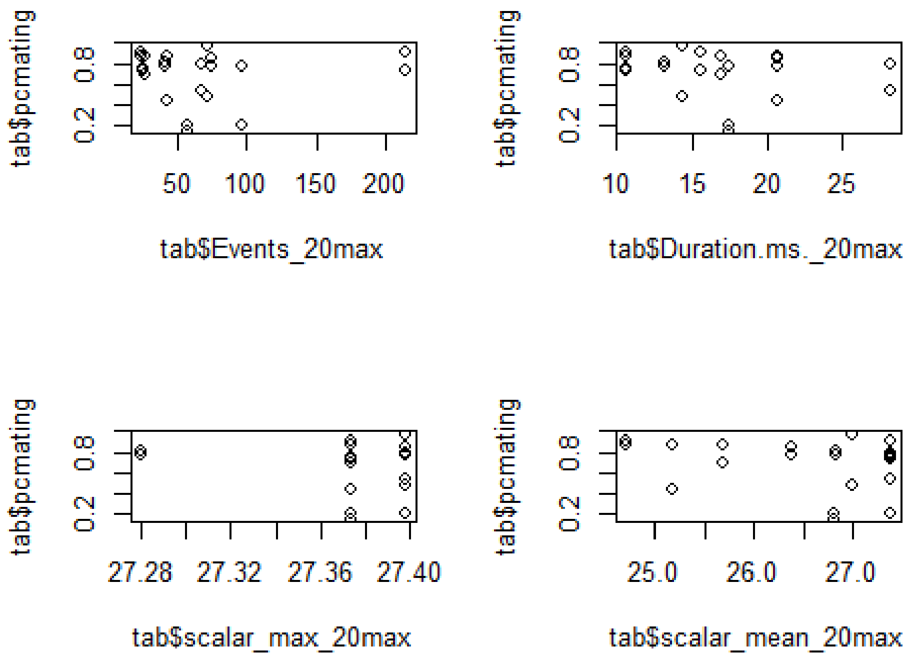


```
plot(tab$pcmating~tab$changescalar_max_20max)
plot(tab$pcmating~tab$changescalar_mean_20max)
plot(tab$pcmating~tab$Changevector_max_20max) #*
plot(tab$pcmating~tab$Changevector_mean_20max)
```

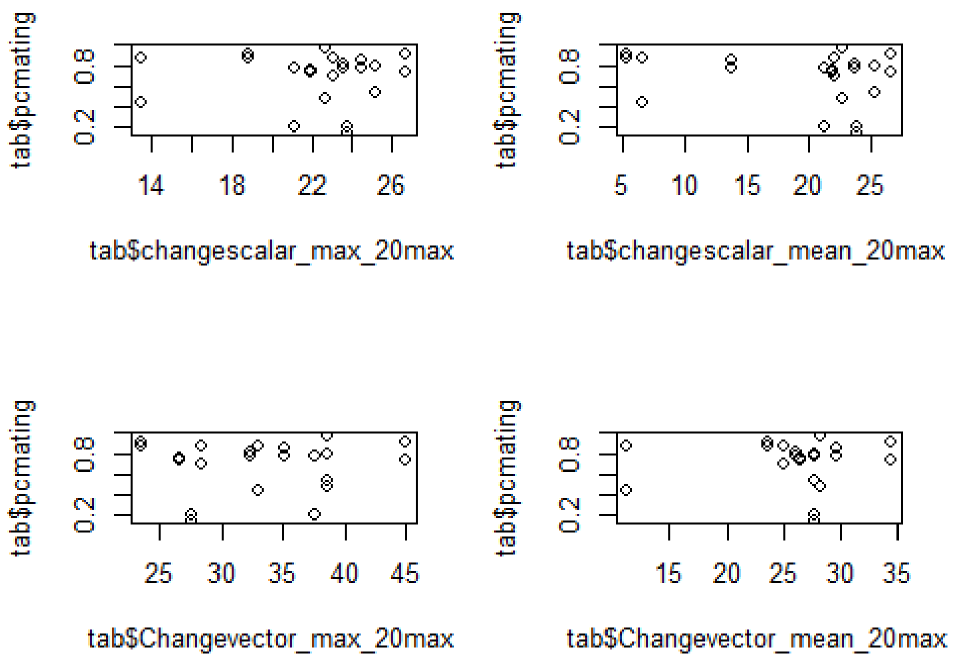


```
plot(tab$pcmating~tab$Angle_max_20max)
plot(tab$pcmating~tab$Angle_mean_20max)#*
plot(tab$pcmating~tab$Hz_max_20max)#*
plot(tab$pcmating~tab$Hz_mean_20max)#*
```

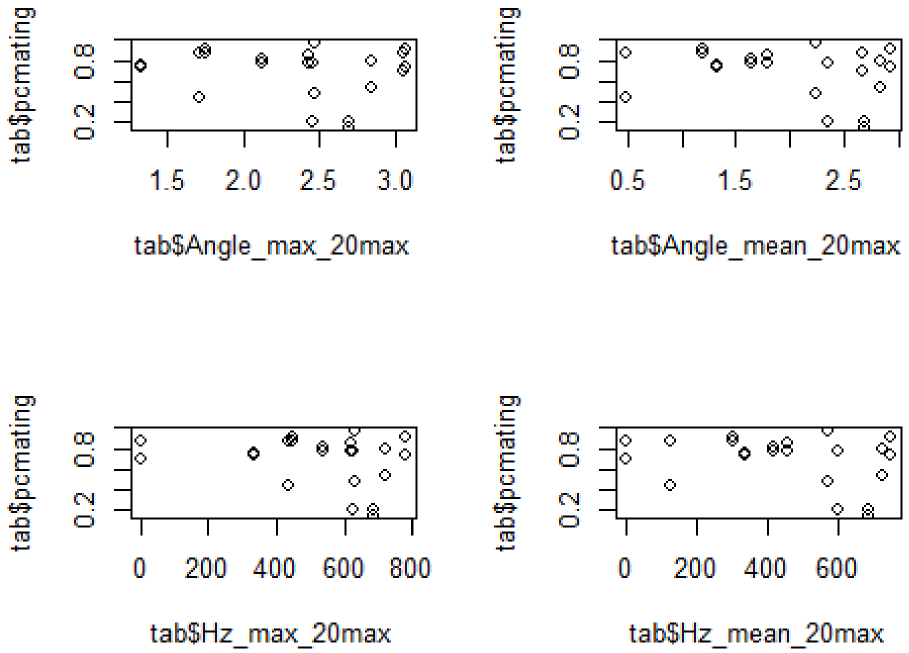

#Scatter plots at threshold 20-Mean

```
par(mfrow = c(2,2))
plot(tab$pcmating~tab$Duration.ms._20mean)
plot(tab$pcmating~tab$scalar_max_20mean)#*
plot(tab$pcmating~tab$scalar_mean_20mean)
plot(tab$pcmating~tab$changescalar_max_20mean)
```

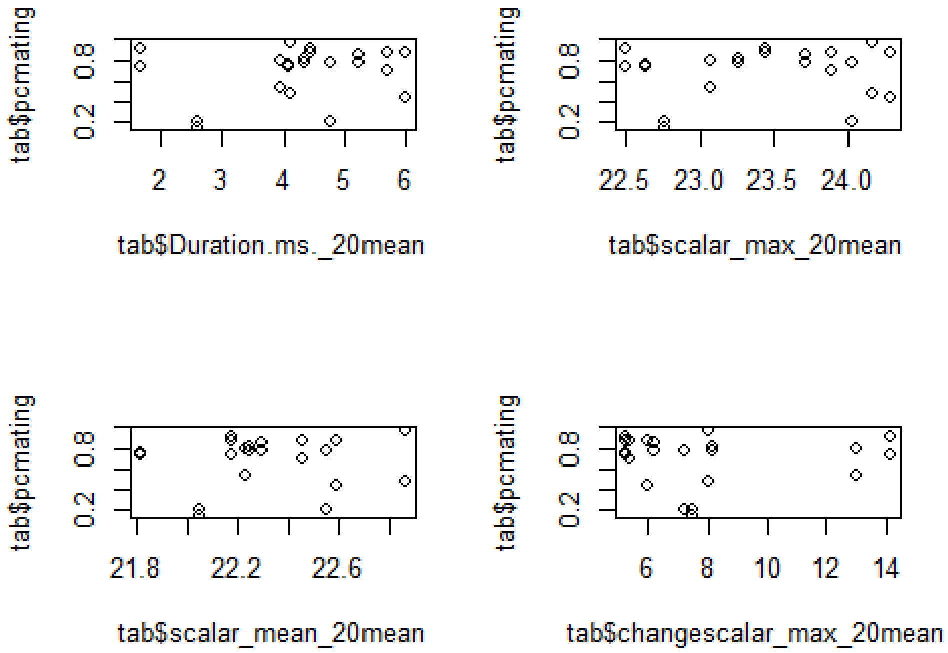


```
plot(tab$pcmating~tab$changescalar_mean_20mean)
plot(tab$pcmating~tab$Changevector_max_20mean)
plot(tab$pcmating~tab$Changevector_mean_20mean)
plot(tab$pcmating~tab$Angle_max_20mean)
```

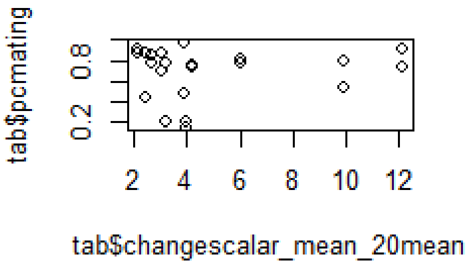

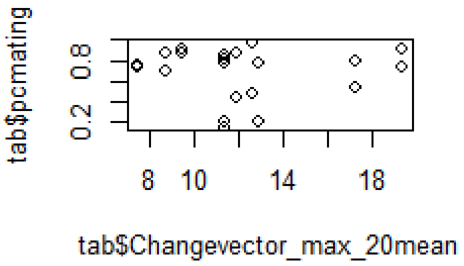

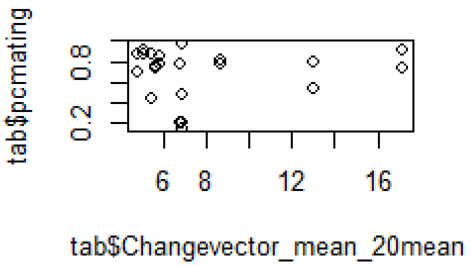

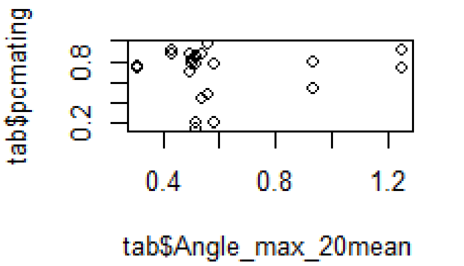


```
plot(tab$pcmating~tab$Angle_mean_20mean)
plot(tab$pcmating~tab$Hz_max_20mean)
plot(tab$pcmating~tab$Hz_mean_20mean)
```

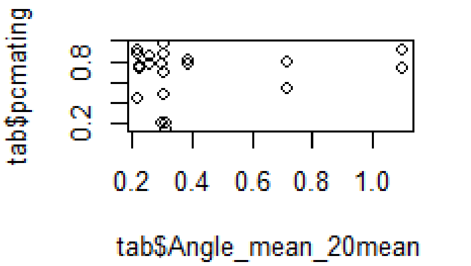

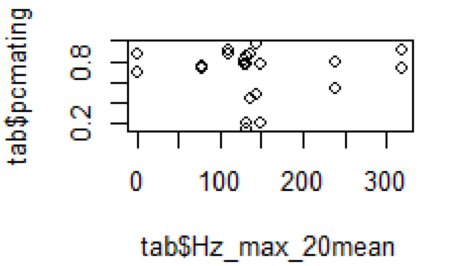

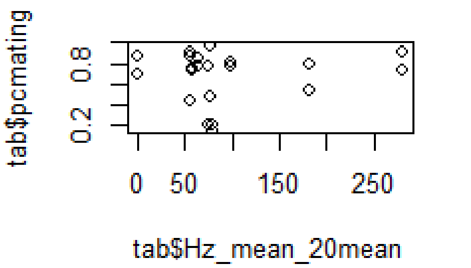

```r
tab$Age <- as.factor(tab$Age)



#***************************************************************************
************************************************************************

#####Caroline
#models threshold 5
fm1 <- glmer(cbind(pairs_formed,unformed_pairs) ~ Irradiation + RH._Max +
Temp_mean + (1|Replicate), family = binomial, data = tab)
fm2 <- glmer(cbind(pairs_formed,unformed_pairs) ~ Irradiation + RH._Max +
Temp_mean + changescalar_mean_5max + Changevector_mean_5max + (1|Replicate),
family = binomial, data = tab)
fm3 <- glmer(cbind(pairs_formed,unformed_pairs) ~ Irradiation + RH._Max +
Temp_mean + changescalar_mean_5max + Duration.ms._5mean +(1|Replicate),
family = binomial, data = tab)
fm4 <- glmer(cbind(pairs_formed,unformed_pairs) ~ Irradiation + RH._Max +
Temp_mean + changescalar_mean_5max + (1|Replicate), family = binomial, data =
tab)
fm5 <- glmer(cbind(pairs_formed,unformed_pairs) ~ Irradiation + RH._Max +
Temp_mean + changescalar_mean_5max + Duration.ms._5mean +
Changevector_mean_5max + (1|Replicate), family = binomial, data = tab)
fm6 <- glmer(cbind(pairs_formed,unformed_pairs) ~ Irradiation + RH._Max +
Temp_mean  + Duration.ms._5mean + (1|Replicate), family = binomial, data =
tab)
fm7 <- glmer(cbind(pairs_formed,unformed_pairs) ~ Irradiation + RH._Max +
Temp_mean + changescalar_mean_5max + Changevector_mean_5max
+Duration.ms._5mean +(1|Replicate), family = binomial, data = tab)
fm8 <- glmer(cbind(pairs_formed,unformed_pairs) ~ Irradiation + Temp_mean +
changescalar_mean_5max + Duration.ms._5max +(1|Replicate), family = binomial,
data = tab)
fm1a<- glmer(cbind(pairs_formed,unformed_pairs) ~ Irradiation + RH._Max +
Temp_mean + Duration.ms._5mean + (1|Replicate), family = binomial, data =
tab)
fm1b <- glmer(cbind(pairs_formed,unformed_pairs) ~ Irradiation + RH._Max +
(1|Replicate), family = binomial, data = tab)
fm2a<- glmer(cbind(pairs_formed,unformed_pairs) ~ Irradiation +
Changevector_mean_5max + Age +(1|Replicate), family = binomial, data = tab)
fm1c <- glmer(cbind(pairs_formed,unformed_pairs) ~ Irradiation + RH._Max +
Temp_mean + Age+(1|Replicate), family = binomial, data = tab)
fm1d <- glmer(cbind(pairs_formed,unformed_pairs) ~ Irradiation + Age +
RH._Max + (1|Replicate), family = binomial, data = tab)
fm1e <- glmer(cbind(pairs_formed,unformed_pairs) ~ Irradiation + Age +
Temp_mean + (1|Replicate), family = binomial, data = tab)
AICc(fm1,fm2,fm3,fm4,fm5,fm6,fm7,fm8,fm1a,fm1b,fm1c,fm1d,fm1e,fm2a)

##       df     AICc
## fm1    5 188.2524
## fm2    7 167.4287
```

```
## fm3   7 168.7920
## fm4   6 185.6606
## fm5   8 171.4418
## fm6   6 169.0118
## fm7   8 171.4418
## fm8   6 188.8255
## fm1a  6 169.0118
## fm1b  4 210.5869
## fm1c  6 190.4239
## fm1d  5 186.6885
## fm1e  5 204.5465
## fm2a  5 204.9368
```

```
summary(fm2)
```

```
## Generalized linear mixed model fit by maximum likelihood (Laplace
##   Approximation) [glmerMod]
##  Family: binomial  ( logit )
## Formula: cbind(pairs_formed, unformed_pairs) ~ Irradiation + RH._Max +
##     Temp_mean + changescalar_mean_5max + Changevector_mean_5max +
##     (1 | Replicate)
##    Data: tab
##
##      AIC      BIC   logLik deviance df.resid
##    159.4    167.1    -72.7    145.4       15
##
## Scaled residuals:
##     Min      1Q  Median      3Q     Max
## -2.9313 -0.5176  0.1348  0.7859  3.5493
##
## Random effects:
##  Groups    Name        Variance Std.Dev.
##  Replicate (Intercept) 0.4348   0.6594
## Number of obs: 22, groups:  Replicate, 6
##
## Fixed effects:
##                         Estimate Std. Error z value Pr(>|z|)
## (Intercept)              1.32718    2.02066   0.657  0.51130
## Irradiationyes          -0.73659    0.18901  -3.897 9.73e-05 ***
## RH._Max                  0.04282    0.01408   3.042  0.00235 **
## Temp_mean               -0.16014    0.05521  -2.901  0.00372 **
## changescalar_mean_5max -0.27899    0.05918  -4.714 2.43e-06 ***
## Changevector_mean_5max  0.23337    0.05020   4.649 3.34e-06 ***
## ---
## Signif. codes:  0 '***' 0.001 '**' 0.01 '*' 0.05 '.' 0.1 ' ' 1
##
## Correlation of Fixed Effects:
##             (Intr) Irrdtn RH._Mx Tmp_mn chn__5
## Irradiatnys -0.125
## RH._Max     -0.590  0.061
```

```
## Temp_mean    -0.853  0.068  0.132
## chngsclr__5 -0.529  0.094  0.216  0.565
## Chngvctr__5 -0.105 -0.072 -0.140  0.136 -0.697

##models for threshold 10

fm11 <- glmer(cbind(pairs_formed,unformed_pairs) ~ Irradiation + RH._Max +
Temp_mean + Duration.ms._10max + changescalar_max_10max+Angle_max_10max+
(1|Replicate), family = binomial, data = tab)
fm12 <- glmer(cbind(pairs_formed,unformed_pairs) ~ Irradiation + RH._Max +
Temp_mean + Duration.ms._10max + changescalar_max_10max+ (1|Replicate),
family = binomial, data = tab)
fm13 <- glmer(cbind(pairs_formed,unformed_pairs) ~ Irradiation + Temp_mean
+RH._Max + Duration.ms._10mean +  Changevector_mean_10mean +
changescalar_max_10max+(1|Replicate), family = binomial, data = tab)
fm14 <- glmer(cbind(pairs_formed,unformed_pairs) ~ Irradiation + RH._Max
+Duration.ms._10max + changescalar_mean_10max+Changevector_mean_10mean +
(1|Replicate), family = binomial, data = tab)
fm15 <- glmer(cbind(pairs_formed,unformed_pairs) ~ Irradiation + Temp_mean
+Duration.ms._10mean + changescalar_mean_10max+ (1|Replicate), family =
binomial, data = tab)
fm11a <- glmer(cbind(pairs_formed,unformed_pairs) ~ Irradiation + RH._Max
+Temp_mean + Duration.ms._10max + Changevector_mean_10mean
+changescalar_max_10max+(1|Replicate), family = binomial, data = tab)
fm11b <- glmer(cbind(pairs_formed,unformed_pairs) ~ Irradiation + RH._Max +
Temp_mean + Duration.ms._10max + Changevector_max_10mean + Angle_max_10max+
(1|Replicate), family = binomial, data = tab)
fm11c <- glmer(cbind(pairs_formed,unformed_pairs) ~ Irradiation + RH._Max +
Temp_mean + Duration.ms._10mean + Changevector_mean_10mean +(1|Replicate),
family = binomial, data = tab)
fm11d <- glmer(cbind(pairs_formed,unformed_pairs) ~ Irradiation + Temp_mean
+Duration.ms._10mean +Changevector_mean_10mean + Angle_max_10max+
(1|Replicate), family = binomial, data = tab)

AICc(fm1,fm2,fm3,fm4,fm5,fm6,fm7,fm8,fm1a,fm1b,fm1c,fm1d,fm1e,fm2a,fm11,fm12,
fm13,fm14,fm15,fm11a,fm11b,fm11c,fm11d)

##        df     AICc
## fm1     5 188.2524
## fm2     7 167.4287
## fm3     7 168.7920
## fm4     6 185.6606
## fm5     8 171.4418
## fm6     6 169.0118
## fm7     8 171.4418
## fm8     6 188.8255
## fm1a    6 169.0118
## fm1b    4 210.5869
## fm1c    6 190.4239
## fm1d    5 186.6885
```

```
## fm1e   5 204.5465
## fm2a   5 204.9368
## fm11   8 188.6461
## fm12   7 185.4325
## fm13   8 179.6289
## fm14   7 218.0183
## fm15   6 170.2412
## fm11a  8 185.5155
## fm11b  8 187.6725
## fm11c  7 182.7774
## fm11d  7 182.9574
```

```
summary(fm2)
```

```
## Generalized linear mixed model fit by maximum likelihood (Laplace
##   Approximation) [glmerMod]
##  Family: binomial  ( logit )
## Formula: cbind(pairs_formed, unformed_pairs) ~ Irradiation + RH._Max +
##     Temp_mean + changescalar_mean_5max + Changevector_mean_5max +
##     (1 | Replicate)
##    Data: tab
##
##      AIC      BIC   logLik deviance df.resid
##    159.4    167.1    -72.7    145.4       15
##
## Scaled residuals:
##     Min      1Q  Median      3Q     Max
## -2.9313 -0.5176  0.1348  0.7859  3.5493
##
## Random effects:
##  Groups    Name        Variance Std.Dev.
##  Replicate (Intercept) 0.4348   0.6594
## Number of obs: 22, groups:  Replicate, 6
##
## Fixed effects:
##                         Estimate Std. Error z value Pr(>|z|)
## (Intercept)              1.32718    2.02066   0.657  0.51130
## Irradiationyes          -0.73659    0.18901  -3.897 9.73e-05 ***
## RH._Max                  0.04282    0.01408   3.042  0.00235 **
## Temp_mean               -0.16014    0.05521  -2.901  0.00372 **
## changescalar_mean_5max -0.27899    0.05918  -4.714 2.43e-06 ***
## Changevector_mean_5max  0.23337    0.05020   4.649 3.34e-06 ***
## ---
## Signif. codes:  0 '***' 0.001 '**' 0.01 '*' 0.05 '.' 0.1 ' ' 1
##
## Correlation of Fixed Effects:
##             (Intr) Irrdtn RH._Mx Tmp_mn chn__5
## Irradiatnys -0.125
## RH._Max     -0.590  0.061
## Temp_mean   -0.853  0.068  0.132
```

```
## chngsclr__5 -0.529  0.094  0.216  0.565
## Chngvctr__5 -0.105 -0.072 -0.140  0.136 -0.697

#

##threshold 15
fm21 <- glmer(cbind(pairs_formed,unformed_pairs) ~ Irradiation + Temp_mean +
changescalar_mean_15max + Duration.ms._15mean + Age+(1|Replicate), family =
binomial, data = tab)
fm22 <- glmer(cbind(pairs_formed,unformed_pairs) ~ Irradiation + Temp_mean +
changescalar_mean_15max +Duration.ms._15max +(1|Replicate), family =
binomial, data = tab)
fm23 <- glmer(cbind(pairs_formed,unformed_pairs) ~ Irradiation +
changescalar_mean_15max + Age +(1|Replicate), family = binomial, data = tab)
fm24 <- glmer(cbind(pairs_formed,unformed_pairs) ~ Irradiation +
changescalar_max_15mean  + Angle_max_15max+
Angle_mean_15max+Duration.ms._15mean +(1|Replicate), family = binomial, data
= tab)
fm25 <- glmer(cbind(pairs_formed,unformed_pairs) ~ Irradiation +
changescalar_mean_15max + Angle_mean_15max+ (1|Replicate), family = binomial,
data = tab)
fm26 <- glmer(cbind(pairs_formed,unformed_pairs) ~ Irradiation +
changescalar_mean_15max + Duration.ms._15mean +(1|Replicate), family =
binomial, data = tab)
fm27 <- glmer(cbind(pairs_formed,unformed_pairs) ~ Irradiation +
changescalar_max_15mean  + Age + (1|Replicate), family = binomial, data =
tab)
fm28 <- glmer(cbind(pairs_formed,unformed_pairs) ~ Irradiation + Temp_mean +
changescalar_mean_15mean +(1|Replicate), family = binomial, data = tab)
fm29 <- glmer(cbind(pairs_formed,unformed_pairs) ~ Irradiation +
changescalar_mean_15max + Changevector_mean_15max+ (1|Replicate), family =
binomial, data = tab)
fm30 <- glmer(cbind(pairs_formed,unformed_pairs) ~ Irradiation +Temp_mean +
Duration.ms._15max + Age+(1|Replicate), family = binomial, data = tab)
fm30a <- glmer(cbind(pairs_formed,unformed_pairs) ~ Irradiation +Temp_mean +
Duration.ms._15max +changescalar_mean_15mean + Angle_mean_15max+
(1|Replicate), family = binomial, data = tab)
fm29a<- glmer(cbind(pairs_formed,unformed_pairs) ~ Irradiation +
changescalar_max_15mean + Duration.ms._15mean +(1|Replicate), family =
binomial, data = tab)

AICc(fm1,fm2,fm3,fm4,fm5,fm6,fm7,fm8,fm1a,fm1b,fm1c,fm1d,fm1e,fm2a,fm11,fm12,
fm13,fm14,fm15,fm11a,fm11b,fm11c,fm11d,fm21,fm22,fm23,fm24,fm25,fm26,fm27,fm2
8,fm29,fm29a,fm30,fm30a)

##       df     AICc
## fm1    5 188.2524
## fm2    7 167.4287
## fm3    7 168.7920
## fm4    6 185.6606
```

```
## fm5    8 171.4418
## fm6    6 169.0118
## fm7    8 171.4418
## fm8    6 188.8255
## fm1a   6 169.0118
## fm1b   4 210.5869
## fm1c   6 190.4239
## fm1d   5 186.6885
## fm1e   5 204.5465
## fm2a   5 204.9368
## fm11   8 188.6461
## fm12   7 185.4325
## fm13   8 179.6289
## fm14   7 218.0183
## fm15   6 170.2412
## fm11a  8 185.5155
## fm11b  8 187.6725
## fm11c  7 182.7774
## fm11d  7 182.9574
## fm21   7 197.1327
## fm22   6 194.7331
## fm23   5 197.8296
## fm24   7 211.4870
## fm25   5 221.7540
## fm26   5 205.6269
## fm27   5 193.1944
## fm28   5 195.2344
## fm29   5 221.8427
## fm29a  5 209.5454
## fm30   6 188.2935
## fm30a  7 184.8849
```

```
summary(fm2)
```

```
## Generalized linear mixed model fit by maximum likelihood (Laplace
##   Approximation) [glmerMod]
##  Family: binomial  ( logit )
## Formula: cbind(pairs_formed, unformed_pairs) ~ Irradiation + RH._Max +
##     Temp_mean + changescalar_mean_5max + Changevector_mean_5max +
##     (1 | Replicate)
##    Data: tab
##
##      AIC      BIC   logLik deviance df.resid
##    159.4    167.1    -72.7    145.4       15
##
## Scaled residuals:
##     Min      1Q  Median      3Q     Max
## -2.9313 -0.5176  0.1348  0.7859  3.5493
##
## Random effects:
```

```
##  Groups    Name        Variance Std.Dev.
##  Replicate (Intercept) 0.4348   0.6594  
## Number of obs: 22, groups:  Replicate, 6
## 
## Fixed effects:
##                         Estimate Std. Error z value Pr(>|z|)    
## (Intercept)              1.32718    2.02066   0.657  0.51130    
## Irradiationyes          -0.73659    0.18901  -3.897 9.73e-05 ***
## RH._Max                  0.04282    0.01408   3.042  0.00235 ** 
## Temp_mean               -0.16014    0.05521  -2.901  0.00372 ** 
## changescalar_mean_5max -0.27899    0.05918  -4.714 2.43e-06 ***
## Changevector_mean_5max  0.23337    0.05020   4.649 3.34e-06 ***
## ---
## Signif. codes:  0 '***' 0.001 '**' 0.01 '*' 0.05 '.' 0.1 ' ' 1
## 
## Correlation of Fixed Effects:
##             (Intr) Irrdtn RH._Mx Tmp_mn chn__5
## Irradiatnys -0.125                            
## RH._Max     -0.590  0.061                     
## Temp_mean   -0.853  0.068  0.132              
## chngsclr__5 -0.529  0.094  0.216  0.565       
## Chngvctr__5 -0.105 -0.072 -0.140  0.136 -0.697

##Threshold 20

fm31 <- glmer(cbind(pairs_formed,unformed_pairs) ~ Irradiation + Temp_mean + Angle_mean_20max + scalar_max_20mean +(1|Replicate), family = binomial, data = tab)
fm32 <- glmer(cbind(pairs_formed,unformed_pairs) ~ Irradiation + Temp_mean + Changevector_max_20max + scalar_max_20mean +(1|Replicate), family = binomial, data = tab)
fm31a <- glmer(cbind(pairs_formed,unformed_pairs) ~ Irradiation + Age + Temp_mean +scalar_max_20mean + (1|Replicate), family = binomial, data = tab)
fm32a <- glmer(cbind(pairs_formed,unformed_pairs) ~ Irradiation + Temp_mean + Angle_mean_20max + Changevector_max_20max + (1|Replicate), family = binomial, data = tab)

AICc(fm1,fm2,fm3,fm4,fm5,fm6,fm7,fm8,fm1a,fm1b,fm1c,fm1d,fm1e,fm2a,fm11,fm12,fm13,fm14,fm15,fm11a,fm11b,fm11c,fm11d,fm21,fm22,fm23,fm24,fm25,fm26,fm27,fm28,fm29,fm29a,fm30,fm30a,fm31,fm32,fm31a,fm32a)

##       df     AICc
## fm1    5 188.2524
## fm2    7 167.4287
## fm3    7 168.7920
## fm4    6 185.6606
## fm5    8 171.4418
## fm6    6 169.0118
## fm7    8 171.4418
## fm8    6 188.8255
```

```
## fm1a   6 169.0118
## fm1b   4 210.5869
## fm1c   6 190.4239
## fm1d   5 186.6885
## fm1e   5 204.5465
## fm2a   5 204.9368
## fm11   8 188.6461
## fm12   7 185.4325
## fm13   8 179.6289
## fm14   7 218.0183
## fm15   6 170.2412
## fm11a  8 185.5155
## fm11b  8 187.6725
## fm11c  7 182.7774
## fm11d  7 182.9574
## fm21   7 197.1327
## fm22   6 194.7331
## fm23   5 197.8296
## fm24   7 211.4870
## fm25   5 221.7540
## fm26   5 205.6269
## fm27   5 193.1944
## fm28   5 195.2344
## fm29   5 221.8427
## fm29a  5 209.5454
## fm30   6 188.2935
## fm30a  7 184.8849
## fm31   6 186.9519
## fm32   6 192.9375
## fm31a  6 181.2781
## fm32a  6 188.7082
```

```
summary(fm2)
```

```
## Generalized linear mixed model fit by maximum likelihood (Laplace
##   Approximation) [glmerMod]
##  Family: binomial  ( logit )
## Formula: cbind(pairs_formed, unformed_pairs) ~ Irradiation + RH._Max +
##     Temp_mean + changescalar_mean_5max + Changevector_mean_5max +
##     (1 | Replicate)
##    Data: tab
##
##      AIC      BIC   logLik deviance df.resid
##    159.4    167.1    -72.7    145.4       15
##
## Scaled residuals:
##     Min      1Q  Median      3Q     Max
## -2.9313 -0.5176  0.1348  0.7859  3.5493
##
## Random effects:
```

```
##  Groups    Name        Variance Std.Dev.
##  Replicate (Intercept) 0.4348   0.6594
## Number of obs: 22, groups:  Replicate, 6
##
## Fixed effects:
##                         Estimate Std. Error z value Pr(>|z|)
## (Intercept)              1.32718    2.02066   0.657  0.51130
## Irradiationyes          -0.73659    0.18901  -3.897 9.73e-05 ***
## RH._Max                  0.04282    0.01408   3.042  0.00235 **
## Temp_mean               -0.16014    0.05521  -2.901  0.00372 **
## changescalar_mean_5max -0.27899    0.05918  -4.714 2.43e-06 ***
## Changevector_mean_5max  0.23337    0.05020   4.649 3.34e-06 ***
## ---
## Signif. codes:  0 '***' 0.001 '**' 0.01 '*' 0.05 '.' 0.1 ' ' 1
##
## Correlation of Fixed Effects:
##             (Intr) Irrdtn RH._Mx Tmp_mn chn__5
## Irradiatnys -0.125
## RH._Max     -0.590  0.061
## Temp_mean   -0.853  0.068  0.132
## chngsclr__5 -0.529  0.094  0.216  0.565
## Chngvctr__5 -0.105 -0.072 -0.140  0.136 -0.697
```

```
#combination of thresehold s

fm41 <- glmer(cbind(pairs_formed,unformed_pairs) ~ Irradiation + Temp_mean
+Duration.ms._10mean + changescalar_mean_10max+Changevector_mean_5max + Age
+(1|Replicate), family = binomial, data = tab)
fm42 <- glmer(cbind(pairs_formed,unformed_pairs) ~ Irradiation + RH._Max +
Temp_mean + changescalar_mean_5max + changescalar_mean_10max+ (1|Replicate),
family = binomial, data = tab)
fm41a <- glmer(cbind(pairs_formed,unformed_pairs) ~ Irradiation + Temp_mean
+Duration.ms._10mean + Changevector_mean_5max +(1|Replicate), family =
binomial, data = tab)
fm42a <- glmer(cbind(pairs_formed,unformed_pairs) ~ Irradiation + RH._Max +
Temp_mean + changescalar_mean_5max + changescalar_mean_10max+ (1|Replicate),
family = binomial, data = tab)
fm41b <- glmer(cbind(pairs_formed,unformed_pairs) ~ Irradiation + Temp_mean
+Duration.ms._10mean + changescalar_mean_10max+Changevector_mean_5max
+(1|Replicate), family = binomial, data = tab)

AICc(fm1,fm2,fm3,fm4,fm5,fm6,fm7,fm8,fm1a,fm1b,fm1c,fm1d,fm1e,fm2a,fm11,fm12,
fm13,fm14,fm15,fm11a,fm11b,fm11c,fm11d,fm21,fm22,fm23,fm24,fm25,fm26,fm27,fm2
8,fm29,fm29a,fm30,fm30a,fm31,fm32,fm31a,fm32a,fm41,fm41b,fm42,fm41a,fm42a)
```

```
##       df     AICc
## fm1    5 188.2524
## fm2    7 167.4287
## fm3    7 168.7920
## fm4    6 185.6606
```

```
## fm5    8 171.4418
## fm6    6 169.0118
## fm7    8 171.4418
## fm8    6 188.8255
## fm1a   6 169.0118
## fm1b   4 210.5869
## fm1c   6 190.4239
## fm1d   5 186.6885
## fm1e   5 204.5465
## fm2a   5 204.9368
## fm11   8 188.6461
## fm12   7 185.4325
## fm13   8 179.6289
## fm14   7 218.0183
## fm15   6 170.2412
## fm11a  8 185.5155
## fm11b  8 187.6725
## fm11c  7 182.7774
## fm11d  7 182.9574
## fm21   7 197.1327
## fm22   6 194.7331
## fm23   5 197.8296
## fm24   7 211.4870
## fm25   5 221.7540
## fm26   5 205.6269
## fm27   5 193.1944
## fm28   5 195.2344
## fm29   5 221.8427
## fm29a  5 209.5454
## fm30   6 188.2935
## fm30a  7 184.8849
## fm31   6 186.9519
## fm32   6 192.9375
## fm31a  6 181.2781
## fm32a  6 188.7082
## fm41   8 173.0346
## fm41b  7 168.0689
## fm42   7 187.7495
## fm41a  6 194.5786
## fm42a  7 187.7495

#the best model remains as fm2


##Figure 9c

head(tab)

##     Date Replicate Age     Treatments unformed_pairs pairs_formed shipped
## 1 170510        R1  22 Shipped-110Gy              42            8     yes
```

```
## 2 170510        R1  22   Shipped-0Gy             40           10      no
## 3 170517        R2  22 Shipped-110Gy              8           27     yes
## 4 170517        R2  22   Shipped-0Gy              5           30      no
## 5 170524        R3  22 Shipped-110Gy             17           13     yes
## 6 170524        R3  22   Shipped-0Gy              4           26      no
##   Irradiation Events._5max Duration.ms._5max scalar_max_5max
scalar_mean_5max
## 1         yes          909            48.750          27.374
21.43900
## 2          no          909            48.750          27.374
21.43900
## 3         yes          708            87.500          27.398
16.92723
## 4          no          708            87.500          27.398
16.92723
## 5         yes         1187            93.125          27.374
15.43705
## 6          no         1187            93.125          27.374
15.43705
##   changescalar_max_5max changescalar_mean_5max Changevector_max_5max
## 1                23.724                8.94875              27.69156
## 2                23.724                8.94875              27.69156
## 3                24.477               11.37550              35.06987
## 4                24.477               11.37550              35.06987
## 5                16.627                3.83800              33.00443
## 6                16.627                3.83800              33.00443
##   Changevector_mean_5max Angle_max_5max Angle_mean_5max Hz_max_5max
## 1               9.937386       3.002116        2.617132    764.4826
## 2               9.937386       3.002116        2.617132    764.4826
## 3              19.035967       2.969360        1.679485    756.1414
## 4              19.035967       2.969360        1.679485    756.1414
## 5               5.064434       2.105922        1.356174    536.2686
## 6               5.064434       2.105922        1.356174    536.2686
##   Hz_mean_5max Events_10max Duration.ms._10max scalar_max_10max
## 1     666.4471          288             36.875           27.374
## 2     666.4471          288             36.875           27.374
## 3     427.6775          315             50.000           27.398
## 4     427.6775          315             50.000           27.398
## 5     345.3468          238             63.750           27.374
## 6     345.3468          238             63.750           27.374
##   scalar_mean_10max changescalar_max_10max changescalar_mean_10max
## 1           21.5800                 23.724                  12.473
## 2           21.5800                 23.724                  12.473
## 3           20.3750                 24.477                  16.488
## 4           20.3750                 24.477                  16.488
## 5           19.6895                 16.627                   8.965
## 6           19.6895                 16.627                   8.965
##   Changevector_max_10max Changevector_mean_10max Angle_max_10max
## 1               27.69156                15.45774        2.687392
## 2               27.69156                15.45774        2.687392
```

```
## 3              35.06987                22.58486        2.969360
## 4              35.06987                22.58486        2.969360
## 5              33.00443                10.65686        2.105922
## 6              33.00443                10.65686        2.105922
##   Angle_mean_10max Hz_max_10max Hz_mean_10max Events_15max
Duration.ms._15max
## 1        1.0470779     684.3388      266.6362          144
30.625
## 2        1.0470779     684.3388      266.6362          144
30.625
## 3        1.6138687     756.1414      410.9683          183
29.375
## 4        1.6138687     756.1414      410.9683          183
29.375
## 5        0.5851573     536.2686      149.0091          111
41.250
## 6        0.5851573     536.2686      149.0091          111
41.250
##   scalar_max_15max scalar_mean_15max changescalar_max_15max
## 1           27.374          22.42986                 23.724
## 2           27.374          22.42986                 23.724
## 3           27.398          21.71558                 24.477
## 4           27.398          21.71558                 24.477
## 5           27.374          22.77170                 16.627
## 6           27.374          22.77170                 16.627
##   changescalar_mean_15max Changevector_max_15max Changevector_mean_15max
## 1                  15.667               27.69156                19.75723
## 2                  15.667               27.69156                19.75723
## 3                  16.488               35.06987                21.61531
## 4                  16.488               35.06987                21.61531
## 5                  11.474               33.00443                12.83840
## 6                  11.474               33.00443                12.83840
##   Angle_max_15max Angle_mean_15max Hz_max_15max Hz_mean_15max Events_20max
## 1        2.687392        1.8427590     684.3388      469.2547           57
## 2        2.687392        1.8427590     684.3388      469.2547           57
## 3        2.969360        1.6010884     756.1414      407.7138           75
## 4        2.969360        1.6010884     756.1414      407.7138           75
## 5        2.105922        0.6709018     536.2686      170.8438           42
## 6        2.105922        0.6709018     536.2686      170.8438           42
##   Duration.ms._20max scalar_max_20max scalar_mean_20max
changescalar_max_20max
## 1             17.500           27.374          26.80175
23.724
## 2             17.500           27.374          26.80175
23.724
## 3             20.625           27.398          26.38567
24.477
## 4             20.625           27.398          26.38567
24.477
## 5             20.625           27.374          25.17900
```

```
13.480
## 6             20.625           27.374          25.17900
13.480
##   changescalar_mean_20max Changevector_max_20max Changevector_mean_20max
## 1                 23.7240               27.69156                27.69156
## 2                 23.7240               27.69156                27.69156
## 3                 13.7095               35.06987                29.65156
## 4                 13.7095               35.06987                29.65156
## 5                  6.6090               33.00443                11.19072
## 6                  6.6090               33.00443                11.19072
##   Angle_max_20max Angle_mean_20max Hz_max_20max Hz_mean_20max Events_5mean
## 1        2.687392         2.687392     684.3388      684.3388          909
## 2        2.687392         2.687392     684.3388      684.3388          909
## 3        2.433775         1.792941     619.7557      456.5688          708
## 4        2.433775         1.792941     619.7557      456.5688          708
## 5        1.715913         0.489164     436.9538      124.5646         1187
## 6        1.715913         0.489164     436.9538      124.5646         1187
##   Duration.ms._5mean scalar_max_5mean scalar_mean_5mean
changescalar_max_5mean
## 1           5.215209         9.516648          7.473747
3.755884
## 2           5.215209         9.516648          7.473747
3.755884
## 3          11.785840        10.551797          7.565272
2.816095
## 4          11.785840        10.551797          7.565272
2.816095
## 5           7.348884         8.022198          6.506753
1.632164
## 6           7.348884         8.022198          6.506753
1.632164
##   changescalar_mean_5mean Changevector_max_5mean Changevector_mean_5mean
## 1               1.6630629               4.501180                2.219564
## 2               1.6630629               4.501180                2.219564
## 3               1.0076953               3.691769                1.638802
## 4               1.0076953               3.691769                1.638802
## 5               0.6804169               2.293485                1.194539
## 6               0.6804169               2.293485                1.194539
##   Angle_max_5mean Angle_mean_5mean Hz_max_5mean Hz_mean_5mean
Events._10mean
## 1       0.4296757        0.1755708    109.41603      44.70874
288
## 2       0.4296757        0.1755708    109.41603      44.70874
288
## 3       0.3715939        0.1414327     94.62562      36.01553
315
## 4       0.3715939        0.1414327     94.62562      36.01553
315
## 5       0.2539263        0.1310499     64.66179      33.37158
238
```

```
## 6       0.2539263        0.1310499     64.66179      33.37158
238
##   Duration.ms._10mean scalar_max_10mean scalar_mean_10mean
## 1            3.888889          15.49537           13.49979
## 2            3.888889          15.49537           13.49979
## 3            7.706349          16.38244           13.65682
## 4            7.706349          16.38244           13.65682
## 5            7.415966          15.56483           13.15894
## 6            7.415966          15.56483           13.15894
##   changescalar_max_10mean changescalar_mean_10mean Changevector_max_10mean
## 1                6.557340                 3.249119                8.029862
## 2                6.557340                 3.249119                8.029862
## 3                4.928854                 1.971893                6.505797
## 4                4.928854                 1.971893                6.505797
## 5                4.004025                 1.664277                5.891075
## 6                4.004025                 1.664277                5.891075
##   Changevector_mean_10mean Angle_max_10mean Angle_mean_10mean
Hz_max_10mean
## 1                 4.538349        0.4719538         0.2224823
120.1821
## 2                 4.538349        0.4719538         0.2224823
120.1821
## 3                 3.339135        0.4047630         0.1751144
103.0721
## 4                 3.339135        0.4047630         0.1751144
103.0721
## 5                 2.909783        0.3492638         0.1583697
88.9393
## 6                 2.909783        0.3492638         0.1583697
88.9393
##   Hz_mean_10mean Events_15mean Duration.ms._15mean scalar_max_15mean
## 1       56.65466           144            3.441840          19.26462
## 2       56.65466           144            3.441840          19.26462
## 3       44.59251           183            6.342213          19.93954
## 4       44.59251           183            6.342213          19.93954
## 5       40.32851           111            6.920045          19.71768
## 6       40.32851           111            6.920045          19.71768
##   scalar_mean_15mean changescalar_max_15mean changescalar_mean_15mean
## 1           17.94158                8.195410                 4.209463
## 2           17.94158                8.195410                 4.209463
## 3           17.79343                6.271208                 2.424063
## 4           17.79343                6.271208                 2.424063
## 5           17.60073                5.291784                 2.055466
## 6           17.60073                5.291784                 2.055466
##   Changevector_max_15mean Changevector_mean_15mean Angle_max_15mean
## 1               10.145769                 6.194357        0.4732660
## 2               10.145769                 6.194357        0.4732660
## 3                8.773143                 4.485467        0.4584332
## 4                8.773143                 4.485467        0.4584332
## 5                8.644509                 4.178605        0.4437400
```

```
## 6                8.644509                 4.178605        0.4437400
##   Angle_mean_15mean Hz_max_15mean Hz_mean_15mean Events_20mean
## 1         0.2644405      120.5162       67.33922            57
## 2         0.2644405      120.5162       67.33922            57
## 3         0.2042285      116.7390       52.00635            75
## 4         0.2042285      116.7390       52.00635            75
## 5         0.1960600      112.9975       49.92626            42
## 6         0.1960600      112.9975       49.92626            42
##   Duration.ms._20mean scalar_max_20mean scalar_mean_20mean
## 1            2.587719          22.76440           22.04857
## 2            2.587719          22.76440           22.04857
## 3            5.250000          23.71752           22.29175
## 4            5.250000          23.71752           22.29175
## 5            5.997024          24.28693           22.58697
## 6            5.997024          24.28693           22.58697
##   changescalar_max_20mean changescalar_mean_20mean Changevector_max_20mean
## 1                7.502070                 3.960703                11.35123
## 2                7.502070                 3.960703                11.35123
## 3                6.190293                 2.676377                11.36833
## 4                6.190293                 2.676377                11.36833
## 5                5.996190                 2.445063                11.97291
## 6                5.996190                 2.445063                11.97291
##   Changevector_mean_20mean Angle_max_20mean Angle_mean_20mean
Hz_max_20mean
## 1                 6.922532        0.5201012         0.3140733
132.4427
## 2                 6.922532        0.5201012         0.3140733
132.4427
## 3                 5.877764        0.5214004         0.2548023
132.7735
## 4                 5.877764        0.5214004         0.2548023
132.7735
## 5                 5.476046        0.5391966         0.2201107
137.3053
## 6                 5.476046        0.5391966         0.2201107
137.3053
##   Hz_mean_20mean RH._Max Temp_Max RH._mean Temp_mean  pcmating
## 1       79.97812   47.08    23.50    41.69     19.73 0.1600000
## 2       79.97812   60.14    27.50    41.69     19.73 0.2000000
## 3       64.88487   62.61    29.75    53.55     21.79 0.7714286
## 4       64.88487   68.10    32.00    53.55     21.79 0.8571429
## 5       56.05073   57.37    28.69    53.25     23.29 0.4333333
## 6       56.05073   74.59    19.25    53.25     23.29 0.8666667
```

```
str(tab)
```

```
## 'data.frame':    22 obs. of  109 variables:
##  $ Date                     : int  170510 170510 170517 170517 170524
170524 170531 170531 170607 170607 ...
##  $ Replicate                : chr  "R1" "R1" "R2" "R2" ...
```

```
##  $ Age                       : Factor w/ 2 levels "22","29": 1 1 1 1 1 1 1 1
1 1 ...
##  $ Treatments                : chr  "Shipped-110Gy " "Shipped-0Gy "
"Shipped-110Gy " "Shipped-0Gy " ...
##  $ unformed_pairs            : int  42 40 8 5 17 4 16 7 21 1 ...
##  $ pairs_formed              : int  8 10 27 30 13 26 4 23 19 39 ...
##  $ shipped                   : chr  "yes" "no" "yes" "no" ...
##  $ Irradiation               : chr  "yes" "no" "yes" "no" ...
##  $ Events._5max              : int  909 909 708 708 1187 1187 625 625 606
606 ...
##  $ Duration.ms._5max         : num  48.8 48.8 87.5 87.5 93.1 ...
##  $ scalar_max_5max           : num  27.4 27.4 27.4 27.4 27.4 ...
##  $ scalar_mean_5max          : num  21.4 21.4 16.9 16.9 15.4 ...
##  $ changescalar_max_5max     : num  23.7 23.7 24.5 24.5 16.6 ...
##  $ changescalar_mean_5max    : num  8.95 8.95 11.38 11.38 3.84 ...
##  $ Changevector_max_5max     : num  27.7 27.7 35.1 35.1 33 ...
##  $ Changevector_mean_5max    : num  9.94 9.94 19.04 19.04 5.06 ...
##  $ Angle_max_5max            : num  3 3 2.97 2.97 2.11 ...
##  $ Angle_mean_5max           : num  2.62 2.62 1.68 1.68 1.36 ...
##  $ Hz_max_5max               : num  764 764 756 756 536 ...
##  $ Hz_mean_5max              : num  666 666 428 428 345 ...
##  $ Events_10max              : int  288 288 315 315 238 238 261 261 219 219
...
##  $ Duration.ms._10max        : num  36.9 36.9 50 50 63.8 ...
##  $ scalar_max_10max          : num  27.4 27.4 27.4 27.4 27.4 ...
##  $ scalar_mean_10max         : num  21.6 21.6 20.4 20.4 19.7 ...
##  $ changescalar_max_10max    : num  23.7 23.7 24.5 24.5 16.6 ...
##  $ changescalar_mean_10max   : num  12.47 12.47 16.49 16.49 8.97 ...
##  $ Changevector_max_10max    : num  27.7 27.7 35.1 35.1 33 ...
##  $ Changevector_mean_10max   : num  15.5 15.5 22.6 22.6 10.7 ...
##  $ Angle_max_10max           : num  2.69 2.69 2.97 2.97 2.11 ...
##  $ Angle_mean_10max          : num  1.047 1.047 1.614 1.614 0.585 ...
##  $ Hz_max_10max              : num  684 684 756 756 536 ...
##  $ Hz_mean_10max             : num  267 267 411 411 149 ...
##  $ Events_15max              : int  144 144 183 183 111 111 163 163 129 129
...
##  $ Duration.ms._15max        : num  30.6 30.6 29.4 29.4 41.2 ...
##  $ scalar_max_15max          : num  27.4 27.4 27.4 27.4 27.4 ...
##  $ scalar_mean_15max         : num  22.4 22.4 21.7 21.7 22.8 ...
##  $ changescalar_max_15max    : num  23.7 23.7 24.5 24.5 16.6 ...
##  $ changescalar_mean_15max   : num  15.7 15.7 16.5 16.5 11.5 ...
##  $ Changevector_max_15max    : num  27.7 27.7 35.1 35.1 33 ...
##  $ Changevector_mean_15max   : num  19.8 19.8 21.6 21.6 12.8 ...
##  $ Angle_max_15max           : num  2.69 2.69 2.97 2.97 2.11 ...
##  $ Angle_mean_15max          : num  1.843 1.843 1.601 1.601 0.671 ...
##  $ Hz_max_15max              : num  684 684 756 756 536 ...
##  $ Hz_mean_15max             : num  469 469 408 408 171 ...
##  $ Events_20max              : int  57 57 75 75 42 42 97 97 71 71 ...
##  $ Duration.ms._20max        : num  17.5 17.5 20.6 20.6 20.6 ...
##  $ scalar_max_20max          : num  27.4 27.4 27.4 27.4 27.4 ...
```

```
##  $ scalar_mean_20max       : num  26.8 26.8 26.4 26.4 25.2 ...
##  $ changescalar_max_20max  : num  23.7 23.7 24.5 24.5 13.5 ...
##  $ changescalar_mean_20max : num  23.72 23.72 13.71 13.71 6.61 ...
##  $ Changevector_max_20max  : num  27.7 27.7 35.1 35.1 33 ...
##  $ Changevector_mean_20max : num  27.7 27.7 29.7 29.7 11.2 ...
##  $ Angle_max_20max         : num  2.69 2.69 2.43 2.43 1.72 ...
##  $ Angle_mean_20max        : num  2.687 2.687 1.793 1.793 0.489 ...
##  $ Hz_max_20max            : num  684 684 620 620 437 ...
##  $ Hz_mean_20max           : num  684 684 457 457 125 ...
##  $ Events_5mean            : int  909 909 708 708 1187 1187 625 625 606
606 ...
##  $ Duration.ms._5mean      : num  5.22 5.22 11.79 11.79 7.35 ...
##  $ scalar_max_5mean        : num  9.52 9.52 10.55 10.55 8.02 ...
##  $ scalar_mean_5mean       : num  7.47 7.47 7.57 7.57 6.51 ...
##  $ changescalar_max_5mean  : num  3.76 3.76 2.82 2.82 1.63 ...
##  $ changescalar_mean_5mean : num  1.66 1.66 1.01 1.01 0.68 ...
##  $ Changevector_max_5mean  : num  4.5 4.5 3.69 3.69 2.29 ...
##  $ Changevector_mean_5mean : num  2.22 2.22 1.64 1.64 1.19 ...
##  $ Angle_max_5mean         : num  0.43 0.43 0.372 0.372 0.254 ...
##  $ Angle_mean_5mean        : num  0.176 0.176 0.141 0.141 0.131 ...
##  $ Hz_max_5mean            : num  109.4 109.4 94.6 94.6 64.7 ...
##  $ Hz_mean_5mean           : num  44.7 44.7 36 36 33.4 ...
##  $ Events._10mean          : int  288 288 315 315 238 238 261 261 219 219
...
##  $ Duration.ms._10mean     : num  3.89 3.89 7.71 7.71 7.42 ...
##  $ scalar_max_10mean       : num  15.5 15.5 16.4 16.4 15.6 ...
##  $ scalar_mean_10mean      : num  13.5 13.5 13.7 13.7 13.2 ...
##  $ changescalar_max_10mean : num  6.56 6.56 4.93 4.93 4 ...
##  $ changescalar_mean_10mean: num  3.25 3.25 1.97 1.97 1.66 ...
##  $ Changevector_max_10mean : num  8.03 8.03 6.51 6.51 5.89 ...
##  $ Changevector_mean_10mean: num  4.54 4.54 3.34 3.34 2.91 ...
##  $ Angle_max_10mean        : num  0.472 0.472 0.405 0.405 0.349 ...
##  $ Angle_mean_10mean       : num  0.222 0.222 0.175 0.175 0.158 ...
##  $ Hz_max_10mean           : num  120.2 120.2 103.1 103.1 88.9 ...
##  $ Hz_mean_10mean          : num  56.7 56.7 44.6 44.6 40.3 ...
##  $ Events_15mean           : int  144 144 183 183 111 111 163 163 129 129
...
##  $ Duration.ms._15mean     : num  3.44 3.44 6.34 6.34 6.92 ...
##  $ scalar_max_15mean       : num  19.3 19.3 19.9 19.9 19.7 ...
##  $ scalar_mean_15mean      : num  17.9 17.9 17.8 17.8 17.6 ...
##  $ changescalar_max_15mean : num  8.2 8.2 6.27 6.27 5.29 ...
##  $ changescalar_mean_15mean: num  4.21 4.21 2.42 2.42 2.06 ...
##  $ Changevector_max_15mean : num  10.15 10.15 8.77 8.77 8.64 ...
##  $ Changevector_mean_15mean: num  6.19 6.19 4.49 4.49 4.18 ...
##  $ Angle_max_15mean        : num  0.473 0.473 0.458 0.458 0.444 ...
##  $ Angle_mean_15mean       : num  0.264 0.264 0.204 0.204 0.196 ...
##  $ Hz_max_15mean           : num  121 121 117 117 113 ...
##  $ Hz_mean_15mean          : num  67.3 67.3 52 52 49.9 ...
##  $ Events_20mean           : int  57 57 75 75 42 42 97 97 71 71 ...
##  $ Duration.ms._20mean     : num  2.59 2.59 5.25 5.25 6 ...
```

```
##  $ scalar_max_20mean       : num  22.8 22.8 23.7 23.7 24.3 ...
##  $ scalar_mean_20mean      : num  22 22 22.3 22.3 22.6 ...
##  $ changescalar_max_20mean : num  7.5 7.5 6.19 6.19 6 ...
##  $ changescalar_mean_20mean: num  3.96 3.96 2.68 2.68 2.45 ...
##  $ Changevector_max_20mean : num  11.4 11.4 11.4 11.4 12 ...
##   [list output truncated]
```

```
summary(fm2)
```

```
## Generalized linear mixed model fit by maximum likelihood (Laplace
##   Approximation) [glmerMod]
##  Family: binomial  ( logit )
## Formula: cbind(pairs_formed, unformed_pairs) ~ Irradiation + RH._Max +
##     Temp_mean + changescalar_mean_5max + Changevector_mean_5max +
##     (1 | Replicate)
##    Data: tab
##
##      AIC      BIC   logLik deviance df.resid
##    159.4    167.1    -72.7    145.4       15
##
## Scaled residuals:
##     Min      1Q  Median      3Q     Max
## -2.9313 -0.5176  0.1348  0.7859  3.5493
##
## Random effects:
##  Groups    Name        Variance Std.Dev.
##  Replicate (Intercept) 0.4348   0.6594
## Number of obs: 22, groups:  Replicate, 6
##
## Fixed effects:
##                        Estimate Std. Error z value Pr(>|z|)
## (Intercept)             1.32718    2.02066   0.657  0.51130
## Irradiationyes         -0.73659    0.18901  -3.897 9.73e-05 ***
## RH._Max                 0.04282    0.01408   3.042  0.00235 **
## Temp_mean              -0.16014    0.05521  -2.901  0.00372 **
## changescalar_mean_5max -0.27899    0.05918  -4.714 2.43e-06 ***
## Changevector_mean_5max  0.23337    0.05020   4.649 3.34e-06 ***
## ---
## Signif. codes:  0 '***' 0.001 '**' 0.01 '*' 0.05 '.' 0.1 ' ' 1
##
## Correlation of Fixed Effects:
##             (Intr) Irrdtn RH._Mx Tmp_mn chn__5
## Irradiatnys -0.125
## RH._Max     -0.590  0.061
## Temp_mean   -0.853  0.068  0.132
## chngsclr__5 -0.529  0.094  0.216  0.565
## Chngvctr__5 -0.105 -0.072 -0.140  0.136 -0.697
```

```r
plot((pairs_formed / unformed_pairs) ~ fitted(fm2), data = tab)
abline(lm((pairs_formed / unformed_pairs) ~ fitted(fm2), data = tab), col =
"red")
```

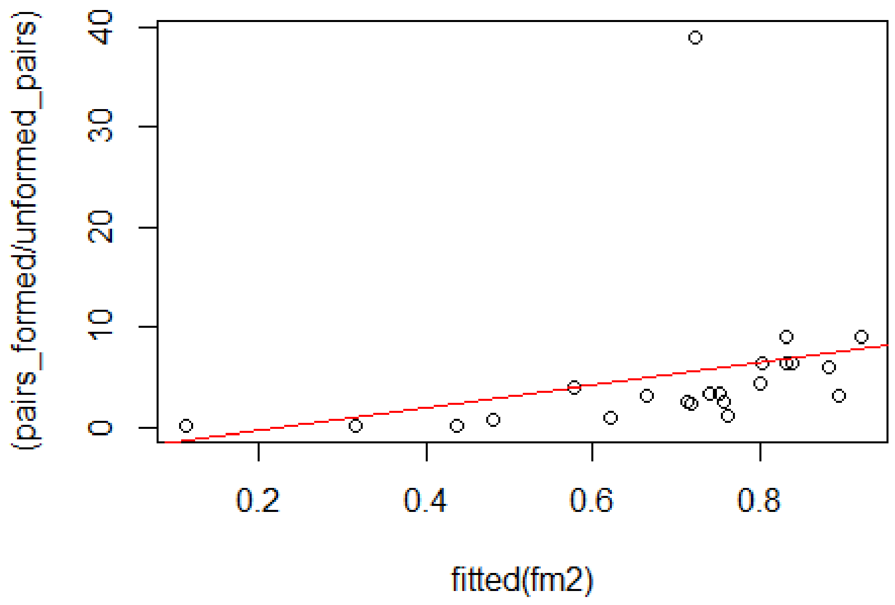


```r
cor.test(tab$pairs_formed/tab$unformed_pairs,fitted(fm2))

##
##  Pearson's product-moment correlation
##
## data:  tab$pairs_formed/tab$unformed_pairs and fitted(fm2)
## t = 1.2977, df = 20, p-value = 0.2091
## alternative hypothesis: true correlation is not equal to 0
## 95 percent confidence interval:
##  -0.1619488  0.6266652
## sample estimates:
##       cor
## 0.2786882

summary(lm(fitted(fm2)~(tab$pairs_formed/tab$unformed_pairs)))$r.squared

## [1] 0.592087

########Correlation of factors from best model(fm12)
cor.test(tab$changescalar_mean_5max,tab$Changevector_mean_5max)

##
##  Pearson's product-moment correlation
##
```

```
## data:  tab$changescalar_mean_5max and tab$Changevector_mean_5max
## t = 13.252, df = 20, p-value = 2.311e-11
## alternative hypothesis: true correlation is not equal to 0
## 95 percent confidence interval:
##  0.8757134 0.9783013
## sample estimates:
##       cor
## 0.9474979

plot(tab$changescalar_mean_5max~tab$Changevector_mean_5max)
```

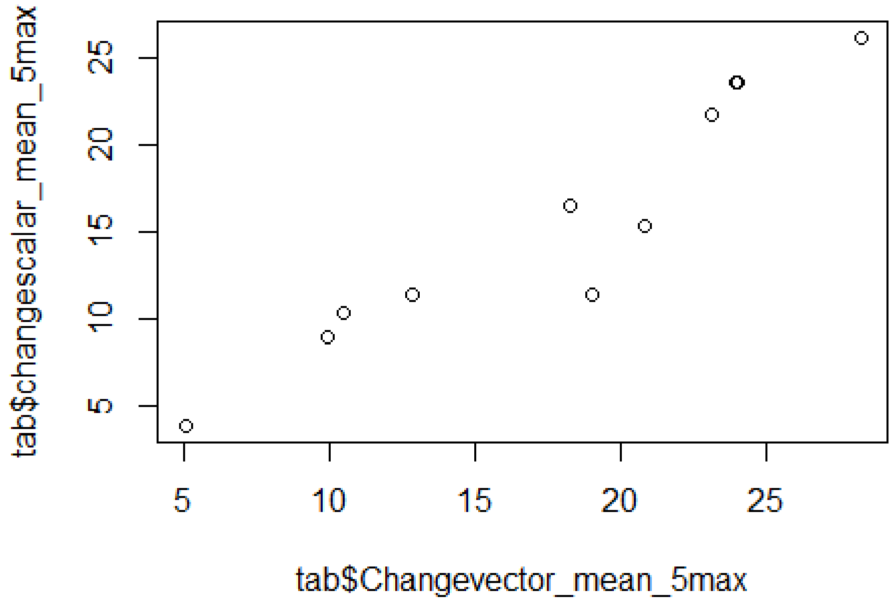


```
## Conclusion: Correlated.One can be dropped


######************************************************************
**********************************************************************
**********
```

THE IMPACT OF SHOCK ON THE INSEMINATION RATE OF 22 AND 29 DAY OLD PUPAE (COMBINED DATA)

Figure 9d:INSEMINATION RATE

```
tab <- read.csv("Figure 9d.csv")
head(tab)

##     Date Replicate Age     Treatments not_full full  MSV MSV2 shipped
## 1 170510        R1  22 Shipped-110Gy        2    6 0.75 1.50     yes
```

```
## 2 170510        R1  22   Shipped-0Gy         4    6 0.70 1.40     yes
## 3 170517        R2  22 Shipped-110Gy       22    5 0.41 0.82     yes
## 4 170517        R2  22   Shipped-0Gy        13   17 0.66 1.32     yes
## 5 170524        R3  22 Shipped-110Gy        6    7 0.60 1.20     yes
## 6 170524        R3  22   Shipped-0Gy        22    4 0.42 0.84     yes
##   Irradiation Events._5max Duration.ms._5max scalar_max_5max
scalar_mean_5max
## 1         yes          909            48.750          27.374
21.43900
## 2          no          909            48.750          27.374
21.43900
## 3         yes          708            87.500          27.398
16.92723
## 4          no          708            87.500          27.398
16.92723
## 5         yes         1187            93.125          27.374
15.43705
## 6          no         1187            93.125          27.374
15.43705
##   changescalar_max_5max changescalar_mean_5max Changevector_max_5max
## 1                23.724                8.94875              27.69156
## 2                23.724                8.94875              27.69156
## 3                24.477               11.37550              35.06987
## 4                24.477               11.37550              35.06987
## 5                16.627                3.83800              33.00443
## 6                16.627                3.83800              33.00443
##   Changevector_mean_5max Angle_max_5max Angle_mean_5max Hz_max_5max
## 1               9.937386       3.002116        2.617132    764.4826
## 2               9.937386       3.002116        2.617132    764.4826
## 3              19.035967       2.969360        1.679485    756.1414
## 4              19.035967       2.969360        1.679485    756.1414
## 5               5.064434       2.105922        1.356174    536.2686
## 6               5.064434       2.105922        1.356174    536.2686
##   Hz_mean_5max Events_10max Duration.ms._10max scalar_max_10max
## 1     666.4471          288             36.875           27.374
## 2     666.4471          288             36.875           27.374
## 3     427.6775          315             50.000           27.398
## 4     427.6775          315             50.000           27.398
## 5     345.3468          238             63.750           27.374
## 6     345.3468          238             63.750           27.374
##   scalar_mean_10max changescalar_max_10max changescalar_mean_10max
## 1           21.5800                 23.724                  12.473
## 2           21.5800                 23.724                  12.473
## 3           20.3750                 24.477                  16.488
## 4           20.3750                 24.477                  16.488
## 5           19.6895                 16.627                   8.965
## 6           19.6895                 16.627                   8.965
##   Changevector_max_10max Changevector_mean_10max Angle_max_10max
## 1               27.69156                15.45774        2.687392
## 2               27.69156                15.45774        2.687392
```

```
## 3               35.06987                22.58486        2.969360
## 4               35.06987                22.58486        2.969360
## 5               33.00443                10.65686        2.105922
## 6               33.00443                10.65686        2.105922
##   Angle_mean_10max Hz_max_10max Hz_mean_10max Events_15max
Duration.ms._15max
## 1        1.0470779     684.3388      266.6362          144
30.625
## 2        1.0470779     684.3388      266.6362          144
30.625
## 3        1.6138687     756.1414      410.9683          183
29.375
## 4        1.6138687     756.1414      410.9683          183
29.375
## 5        0.5851573     536.2686      149.0091          111
41.250
## 6        0.5851573     536.2686      149.0091          111
41.250
##   scalar_max_15max scalar_mean_15max changescalar_max_15max
## 1           27.374          22.42986                 23.724
## 2           27.374          22.42986                 23.724
## 3           27.398          21.71558                 24.477
## 4           27.398          21.71558                 24.477
## 5           27.374          22.77170                 16.627
## 6           27.374          22.77170                 16.627
##   changescalar_mean_15max Changevector_max_15max Changevector_mean_15max
## 1                  15.667               27.69156                19.75723
## 2                  15.667               27.69156                19.75723
## 3                  16.488               35.06987                21.61531
## 4                  16.488               35.06987                21.61531
## 5                  11.474               33.00443                12.83840
## 6                  11.474               33.00443                12.83840
##   Angle_max_15max Angle_mean_15max Hz_max_15max Hz_mean_15max Events_20max
## 1        2.687392        1.8427590     684.3388      469.2547           57
## 2        2.687392        1.8427590     684.3388      469.2547           57
## 3        2.969360        1.6010884     756.1414      407.7138           75
## 4        2.969360        1.6010884     756.1414      407.7138           75
## 5        2.105922        0.6709018     536.2686      170.8438           42
## 6        2.105922        0.6709018     536.2686      170.8438           42
##   Duration.ms._20max scalar_max_20max scalar_mean_20max
changescalar_max_20max
## 1             17.500           27.374          26.80175
23.724
## 2             17.500           27.374          26.80175
23.724
## 3             20.625           27.398          26.38567
24.477
## 4             20.625           27.398          26.38567
24.477
## 5             20.625           27.374          25.17900
```

```
13.480
## 6             20.625           27.374          25.17900
13.480
##   changescalar_mean_20max Changevector_max_20max Changevector_mean_20max
## 1                 23.7240               27.69156                27.69156
## 2                 23.7240               27.69156                27.69156
## 3                 13.7095               35.06987                29.65156
## 4                 13.7095               35.06987                29.65156
## 5                  6.6090               33.00443                11.19072
## 6                  6.6090               33.00443                11.19072
##   Angle_max_20max Angle_mean_20max Hz_max_20max Hz_mean_20max Events_5mean
## 1        2.687392         2.687392     684.3388      684.3388          909
## 2        2.687392         2.687392     684.3388      684.3388          909
## 3        2.433775         1.792941     619.7557      456.5688          708
## 4        2.433775         1.792941     619.7557      456.5688          708
## 5        1.715913         0.489164     436.9538      124.5646         1187
## 6        1.715913         0.489164     436.9538      124.5646         1187
##   Duration.ms._5mean scalar_max_5mean scalar_mean_5mean
changescalar_max_5mean
## 1           5.215209         9.516648          7.473747
3.755884
## 2           5.215209         9.516648          7.473747
3.755884
## 3          11.785840        10.551797          7.565272
2.816095
## 4          11.785840        10.551797          7.565272
2.816095
## 5           7.348884         8.022198          6.506753
1.632164
## 6           7.348884         8.022198          6.506753
1.632164
##   changescalar_mean_5mean Changevector_max_5mean Changevector_mean_5mean
## 1               1.6630629               4.501180                2.219564
## 2               1.6630629               4.501180                2.219564
## 3               1.0076953               3.691769                1.638802
## 4               1.0076953               3.691769                1.638802
## 5               0.6804169               2.293485                1.194539
## 6               0.6804169               2.293485                1.194539
##   Angle_max_5mean Angle_mean_5mean Hz_max_5mean Hz_mean_5mean
Events._10mean
## 1       0.4296757        0.1755708    109.41603      44.70874
288
## 2       0.4296757        0.1755708    109.41603      44.70874
288
## 3       0.3715939        0.1414327     94.62562      36.01553
315
## 4       0.3715939        0.1414327     94.62562      36.01553
315
## 5       0.2539263        0.1310499     64.66179      33.37158
238
```

```
## 6       0.2539263        0.1310499     64.66179      33.37158
238
##   Duration.ms._10mean scalar_max_10mean scalar_mean_10mean
## 1            3.888889          15.49537           13.49979
## 2            3.888889          15.49537           13.49979
## 3            7.706349          16.38244           13.65682
## 4            7.706349          16.38244           13.65682
## 5            7.415966          15.56483           13.15894
## 6            7.415966          15.56483           13.15894
##   changescalar_max_10mean changescalar_mean_10mean Changevector_max_10mean
## 1                6.557340                 3.249119                8.029862
## 2                6.557340                 3.249119                8.029862
## 3                4.928854                 1.971893                6.505797
## 4                4.928854                 1.971893                6.505797
## 5                4.004025                 1.664277                5.891075
## 6                4.004025                 1.664277                5.891075
##   Changevector_mean_10mean Angle_max_10mean Angle_mean_10mean
Hz_max_10mean
## 1                 4.538349        0.4719538         0.2224823
120.1821
## 2                 4.538349        0.4719538         0.2224823
120.1821
## 3                 3.339135        0.4047630         0.1751144
103.0721
## 4                 3.339135        0.4047630         0.1751144
103.0721
## 5                 2.909783        0.3492638         0.1583697
88.9393
## 6                 2.909783        0.3492638         0.1583697
88.9393
##   Hz_mean_10mean Events_15mean Duration.ms._15mean scalar_max_15mean
## 1       56.65466           144            3.441840          19.26462
## 2       56.65466           144            3.441840          19.26462
## 3       44.59251           183            6.342213          19.93954
## 4       44.59251           183            6.342213          19.93954
## 5       40.32851           111            6.920045          19.71768
## 6       40.32851           111            6.920045          19.71768
##   scalar_mean_15mean changesclar_max_15mean changescalar_mean_15mean
## 1           17.94158               8.195410                 4.209463
## 2           17.94158               8.195410                 4.209463
## 3           17.79343               6.271208                 2.424063
## 4           17.79343               6.271208                 2.424063
## 5           17.60073               5.291784                 2.055466
## 6           17.60073               5.291784                 2.055466
##   Changevector_max_15mean Changevector_mean_15mean Angle_max_15mean
## 1               10.145769                 6.194357        0.4732660
## 2               10.145769                 6.194357        0.4732660
## 3                8.773143                 4.485467        0.4584332
## 4                8.773143                 4.485467        0.4584332
## 5                8.644509                 4.178605        0.4437400
```

```
## 6                8.644509                 4.178605        0.4437400
##   Angle_mean_15mean Hz_max_15mean Hz_mean_15mean Events_20mean
## 1         0.2644405      120.5162       67.33922            57
## 2         0.2644405      120.5162       67.33922            57
## 3         0.2042285      116.7390       52.00635            75
## 4         0.2042285      116.7390       52.00635            75
## 5         0.1960600      112.9975       49.92626            42
## 6         0.1960600      112.9975       49.92626            42
##   Duration.ms._20mean scalar_max_20mean scalar_mean_20mean
## 1            2.587719          22.76440           22.04857
## 2            2.587719          22.76440           22.04857
## 3            5.250000          23.71752           22.29175
## 4            5.250000          23.71752           22.29175
## 5            5.997024          24.28693           22.58697
## 6            5.997024          24.28693           22.58697
##   changescalar_max_20mean changescalar_mean_20mean Changevector_max_20mean
## 1                7.502070                 3.960703                11.35123
## 2                7.502070                 3.960703                11.35123
## 3                6.190293                 2.676377                11.36833
## 4                6.190293                 2.676377                11.36833
## 5                5.996190                 2.445063                11.97291
## 6                5.996190                 2.445063                11.97291
##   Changevector_mean_20mean Angle_max_20mean Angle_mean_20mean
Hz_max_20mean
## 1                 6.922532        0.5201012         0.3140733
132.4427
## 2                 6.922532        0.5201012         0.3140733
132.4427
## 3                 5.877764        0.5214004         0.2548023
132.7735
## 4                 5.877764        0.5214004         0.2548023
132.7735
## 5                 5.476046        0.5391966         0.2201107
137.3053
## 6                 5.476046        0.5391966         0.2201107
137.3053
##   Hz_mean_20mean RH._Max Temp_Max RH._mean Temp_mean
## 1       79.97812   47.08    23.50    41.69     19.73
## 2       79.97812   60.14    27.50    41.69     19.73
## 3       64.88487   62.61    29.75    53.55     21.79
## 4       64.88487   68.10    32.00    53.55     21.79
## 5       56.05073   57.37    28.69    53.25     23.29
## 6       56.05073   74.59    19.25    53.25     23.29
```

```
summary(tab)
```

```
##       Date          Replicate              Age          Treatments
##  Min.   :170510   Length:22          Min.   :22.00   Length:22
##  1st Qu.:170526   Class :character   1st Qu.:22.00   Class :character
##  Median :180620   Mode  :character   Median :29.00   Mode  :character
```

```
##  Mean   :176081                      Mean   :25.82
##  3rd Qu.:180716                      3rd Qu.:29.00
##  Max.   :180801                      Max.   :29.00
##     not_full          full             MSV              MSV2
##  Min.   : 2.00   Min.   : 0.000   Min.   :0.2500   Min.   :0.5000
##  1st Qu.:11.00   1st Qu.: 2.000   1st Qu.:0.4025   1st Qu.:0.8050
##  Median :13.50   Median : 5.500   Median :0.4694   Median :0.9387
##  Mean   :13.82   Mean   : 5.955   Mean   :0.4859   Mean   :0.9718
##  3rd Qu.:17.75   3rd Qu.: 7.750   3rd Qu.:0.5681   3rd Qu.:1.1362
##  Max.   :26.00   Max.   :22.000   Max.   :0.7500   Max.   :1.5000
##    shipped          Irradiation         Events._5max    Duration.ms._5max
##  Length:22          Length:22          Min.   : 341.0   Min.   : 41.88
##  Class :character   Class :character   1st Qu.: 495.0   1st Qu.: 49.06
##  Mode  :character   Mode  :character   Median : 708.0   Median : 59.38
##                                        Mean   : 763.6   Mean   : 76.76
##                                        3rd Qu.:1117.5   3rd Qu.: 97.34
##                                        Max.   :1228.0   Max.   :150.00
##  scalar_max_5max scalar_mean_5max changescalar_max_5max
changescalar_mean_5max
##  Min.   :27.28   Min.   :15.44    Min.   :15.58         Min.   : 3.838
##  1st Qu.:27.37   1st Qu.:17.64    1st Qu.:21.16         1st Qu.:10.623
##  Median :27.37   Median :19.35    Median :23.03         Median :15.391
##  Mean   :27.37   Mean   :20.67    Mean   :22.10         Mean   :15.724
##  3rd Qu.:27.40   3rd Qu.:23.95    3rd Qu.:24.29         3rd Qu.:23.124
##  Max.   :27.40   Max.   :27.13    Max.   :26.65         Max.   :26.175
##  Changevector_max_5max Changevector_mean_5max Angle_max_5max
Angle_mean_5max
##  Min.   :23.56         Min.   : 5.064         Min.   :2.106   Min.
:1.356
##  1st Qu.:29.49         1st Qu.:11.063         1st Qu.:2.505   1st
Qu.:1.682
##  Median :35.07         Median :19.036         Median :2.969   Median
:2.527
##  Mean   :34.55         Mean   :17.786         Mean   :2.795   Mean
:2.304
##  3rd Qu.:38.57         3rd Qu.:23.722         3rd Qu.:3.023   3rd
Qu.:2.689
##  Max.   :43.01         Max.   :28.208         Max.   :3.132   Max.
:3.057
##   Hz_max_5max     Hz_mean_5max    Events_10max   Duration.ms._10max
##  Min.   :536.3   Min.   :345.3   Min.   :109.0   Min.   :23.75
##  1st Qu.:637.8   1st Qu.:428.4   1st Qu.:144.0   1st Qu.:34.69
##  Median :756.1   Median :643.4   Median :238.0   Median :37.50
##  Mean   :711.8   Mean   :586.8   Mean   :285.4   Mean   :41.36
##  3rd Qu.:769.9   3rd Qu.:684.9   3rd Qu.:308.2   3rd Qu.:50.94
##  Max.   :797.5   Max.   :778.4   Max.   :956.0   Max.   :63.75
##  scalar_max_10max scalar_mean_10max changescalar_max_10max
##  Min.   :27.28    Min.   :17.45     Min.   :15.58
##  1st Qu.:27.37    1st Qu.:20.45     1st Qu.:17.76
##  Median :27.37    Median :21.55     Median :21.91
```

```
##  Mean   :27.37    Mean   :22.17     Mean   :21.09
##  3rd Qu.:27.40    3rd Qu.:23.99     3rd Qu.:23.69
##  Max.   :27.40    Max.   :27.37     Max.   :25.20
##  changescalar_mean_10max Changevector_max_10max Changevector_mean_10max
##  Min.   : 8.965          Min.   :23.56          Min.   :10.66
##  1st Qu.:11.337          1st Qu.:26.93          1st Qu.:12.98
##  Median :16.488          Median :33.00          Median :19.71
##  Mean   :15.891          Mean   :33.10          Mean   :18.75
##  3rd Qu.:20.581          3rd Qu.:38.57          3rd Qu.:24.61
##  Max.   :25.203          Max.   :43.01          Max.   :26.96
##  Angle_max_10max Angle_mean_10max  Hz_max_10max   Hz_mean_10max
##  Min.   :1.734   Min.   :0.5721   Min.   :441.5   Min.   :145.7
##  1st Qu.:2.303   1st Qu.:1.1168   1st Qu.:586.4   1st Qu.:284.4
##  Median :2.570   Median :1.6457   Median :654.4   Median :419.1
##  Mean   :2.570   Mean   :1.7194   Mean   :654.4   Mean   :437.8
##  3rd Qu.:3.007   3rd Qu.:2.4297   3rd Qu.:765.7   3rd Qu.:618.7
##  Max.   :3.057   Max.   :3.0198   Max.   :778.5   Max.   :769.0
##   Events_15max    Duration.ms._15max scalar_max_15max scalar_mean_15max
##  Min.   : 54.00   Min.   :18.75      Min.   :27.28    Min.   :21.72
##  1st Qu.: 62.75   1st Qu.:20.16      1st Qu.:27.37    1st Qu.:22.52
##  Median :129.00   Median :22.50      Median :27.37    Median :25.17
##  Mean   :159.45   Mean   :26.82      Mean   :27.37    Mean   :24.79
##  3rd Qu.:161.00   3rd Qu.:30.62      3rd Qu.:27.40    3rd Qu.:27.06
##  Max.   :627.00   Max.   :41.25      Max.   :27.40    Max.   :27.37
##  changescalar_max_15max changescalar_mean_15max Changevector_max_15max
##  Min.   :15.58          Min.   : 9.943          Min.   :23.56
##  1st Qu.:21.16          1st Qu.:12.522          1st Qu.:26.93
##  Median :23.03          Median :16.540          Median :35.07
##  Mean   :22.10          Mean   :18.188          Mean   :34.48
##  3rd Qu.:24.29          3rd Qu.:23.193          3rd Qu.:40.88
##  Max.   :26.65          Max.   :26.513          Max.   :44.89
##  Changevector_mean_15max Angle_max_15max Angle_mean_15max  Hz_max_15max
##  Min.   :12.84           Min.   :1.326   Min.   :0.6202   Min.   :337.7
##  1st Qu.:14.73           1st Qu.:2.189   1st Qu.:0.8599   1st Qu.:557.4
##  Median :21.62           Median :2.687   Median :1.8428   Median :684.3
##  Mean   :21.59           Mean   :2.484   Mean   :1.8436   Mean   :632.5
##  3rd Qu.:26.86           3rd Qu.:2.942   3rd Qu.:2.7354   3rd Qu.:749.1
##  Max.   :30.34           Max.   :3.125   Max.   :3.1249   Max.   :795.7
##  Hz_mean_15max    Events_20max   Duration.ms._20max scalar_max_20max
##  Min.   :157.9   Min.   : 23.0   Min.   :10.62      Min.   :27.28
##  1st Qu.:219.0   1st Qu.: 29.5   1st Qu.:13.44      1st Qu.:27.37
##  Median :469.3   Median : 57.0   Median :16.88      Median :27.37
##  Mean   :469.5   Mean   : 67.0   Mean   :16.88      Mean   :27.37
##  3rd Qu.:696.6   3rd Qu.: 74.0   3rd Qu.:19.84      3rd Qu.:27.40
##  Max.   :795.7   Max.   :215.0   Max.   :28.12      Max.   :27.40
##  scalar_mean_20max changescalar_max_20max changescalar_mean_20max
##  Min.   :24.72     Min.   :13.48          Min.   : 5.401
##  1st Qu.:25.87     1st Qu.:21.34          1st Qu.:15.570
##  Median :26.83     Median :23.03          Median :22.061
##  Mean   :26.55     Mean   :22.23          Mean   :19.313
```

```
##  3rd Qu.:27.37     3rd Qu.:24.29          3rd Qu.:23.686
##  Max.   :27.37     Max.   :26.65          Max.   :26.513
##  Changevector_max_20max Changevector_mean_20max Angle_max_20max
##  Min.   :23.56          Min.   :11.19           Min.   :1.326
##  1st Qu.:27.87          1st Qu.:25.33           1st Qu.:1.844
##  Median :33.00          Median :27.68           Median :2.457
##  Mean   :33.31          Mean   :26.20           Mean   :2.357
##  3rd Qu.:38.32          3rd Qu.:28.17           3rd Qu.:2.800
##  Max.   :44.89          Max.   :34.48           Max.   :3.062
##  Angle_mean_20max  Hz_max_20max     Hz_mean_20max      Events_5mean
##  Min.   :0.4892   Min.   :  3.057   Min.   :  2.671   Min.   : 341.0
##  1st Qu.:1.4039   1st Qu.:439.219   1st Qu.:311.298   1st Qu.: 495.0
##  Median :2.2315   Median :619.756   Median :456.569   Median : 708.0
##  Mean   :2.0118   Mean   :529.617   Mean   :450.696   Mean   : 763.6
##  3rd Qu.:2.6834   3rd Qu.:670.696   3rd Qu.:662.651   3rd Qu.:1117.5
##  Max.   :2.9219   Max.   :779.845   Max.   :744.045   Max.   :1228.0
##  Duration.ms._5mean scalar_max_5mean scalar_mean_5mean
changescalar_max_5mean
##  Min.   : 5.215     Min.   : 7.866   Min.   :6.507     Min.   :1.442
##  1st Qu.: 6.909     1st Qu.: 9.178   1st Qu.:7.410     1st Qu.:2.339
##  Median : 8.597     Median : 9.648   Median :7.565     Median :2.816
##  Mean   : 8.653     Mean   : 9.743   Mean   :7.438     Mean   :3.378
##  3rd Qu.:10.250     3rd Qu.:10.434   3rd Qu.:7.682     3rd Qu.:3.632
##  Max.   :11.786     Max.   :12.092   Max.   :8.188     Max.   :8.696
##  changescalar_mean_5mean Changevector_max_5mean Changevector_mean_5mean
##  Min.   :0.6227          Min.   : 1.767         Min.   :0.8316
##  1st Qu.:0.8770          1st Qu.: 2.939         1st Qu.:1.3240
##  Median :1.1437          Median : 3.692         Median :1.6388
##  Mean   :1.6777          Mean   : 4.463         Mean   :2.3524
##  3rd Qu.:1.6224          3rd Qu.: 4.665         3rd Qu.:2.1882
##  Max.   :4.5129          Max.   :12.182         Max.   :6.4512
##  Angle_max_5mean  Angle_mean_5mean   Hz_max_5mean    Hz_mean_5mean
##  Min.   :0.1659   Min.   :0.07985   Min.   : 42.24   Min.   : 20.33
##  1st Qu.:0.2672   1st Qu.:0.13365   1st Qu.: 68.03   1st Qu.: 34.03
##  Median :0.3716   Median :0.16791   Median : 94.63   Median : 42.76
##  Mean   :0.5221   Mean   :0.28474   Mean   :132.96   Mean   : 72.51
##  3rd Qu.:0.4295   3rd Qu.:0.21880   3rd Qu.:109.37   3rd Qu.: 55.72
##  Max.   :1.7833   Max.   :0.94024   Max.   :454.12   Max.   :239.43
##  Events._10mean  Duration.ms._10mean scalar_max_10mean scalar_mean_10mean
##  Min.   :109.0   Min.   :3.532       Min.   :14.94     Min.   :12.92
##  1st Qu.:144.0   1st Qu.:5.091       1st Qu.:15.57     1st Qu.:13.38
##  Median :238.0   Median :7.046       Median :15.75     Median :13.66
##  Mean   :285.4   Mean   :6.549       Mean   :16.07     Mean   :13.75
##  3rd Qu.:308.2   3rd Qu.:7.710       3rd Qu.:16.60     3rd Qu.:14.10
##  Max.   :956.0   Max.   :8.695       Max.   :17.47     Max.   :14.79
##  changescalar_max_10mean changescalar_mean_10mean Changevector_max_10mean
##  Min.   : 3.380          Min.   :1.479            Min.   : 4.344
##  1st Qu.: 4.173          1st Qu.:1.897            1st Qu.: 6.000
##  Median : 4.929          Median :2.288            Median : 6.506
##  Mean   : 5.991          Mean   :3.423            Mean   : 8.003
```

```
##  3rd Qu.: 6.468          3rd Qu.:3.775            3rd Qu.: 8.712
##  Max.   :12.085          Max.   :9.086            Max.   :16.709
##  Changevector_mean_10mean Angle_max_10mean Angle_mean_10mean Hz_max_10mean
##  Min.   : 2.170           Min.   :0.2465   Min.   :0.1225    Min.   :
62.78
##  1st Qu.: 2.923           1st Qu.:0.3279   1st Qu.:0.1414    1st Qu.:
83.50
##  Median : 3.339           Median :0.4504   Median :0.2215    Median
:114.68
##  Mean   : 4.881           Mean   :0.5915   Mean   :0.3678    Mean
:150.62
##  3rd Qu.: 5.319           3rd Qu.:0.5606   3rd Qu.:0.3422    3rd
Qu.:142.76
##  Max.   :13.024           Max.   :1.8310   Max.   :1.3785    Max.
:466.26
##  Hz_mean_10mean   Events_15mean    Duration.ms._15mean scalar_max_15mean
##  Min.   : 31.20   Min.   : 54.00   Min.   :2.310       Min.   :19.16
##  1st Qu.: 36.02   1st Qu.: 62.75   1st Qu.:4.485       1st Qu.:19.57
##  Median : 56.39   Median :129.00   Median :6.215       Median :19.78
##  Mean   : 93.65   Mean   :159.45   Mean   :5.518       Mean   :19.97
##  3rd Qu.: 87.15   3rd Qu.:161.00   3rd Qu.:6.617       3rd Qu.:20.47
##  Max.   :351.03   Max.   :627.00   Max.   :6.920       Max.   :21.17
##  scalar_mean_15mean changescalar_max_15mean changescalar_mean_15mean
##  Min.   :17.60      Min.   : 4.622          Min.   : 1.704
##  1st Qu.:17.83      1st Qu.: 5.493          1st Qu.: 2.300
##  Median :18.27      Median : 6.393          Median : 2.959
##  Mean   :18.18      Mean   : 7.567          Mean   : 4.400
##  3rd Qu.:18.53      3rd Qu.: 8.247          3rd Qu.: 5.211
##  Max.   :18.71      Max.   :14.038          Max.   :11.493
##  Changevector_max_15mean Changevector_mean_15mean Angle_max_15mean
##  Min.   : 6.224          Min.   : 2.939           Min.   :0.2772
##  1st Qu.: 8.675          1st Qu.: 4.075           1st Qu.:0.3934
##  Median : 9.016          Median : 4.903           Median :0.4733
##  Mean   :10.393          Mean   : 6.549           Mean   :0.6328
##  3rd Qu.:11.444          3rd Qu.: 7.425           3rd Qu.:0.6286
##  Max.   :19.029          Max.   :16.285           Max.   :1.7973
##  Angle_mean_15mean Hz_max_15mean    Hz_mean_15mean   Events_20mean
##  Min.   :0.1236    Min.   : 70.58   Min.   : 31.48   Min.   : 23.0
##  1st Qu.:0.1790    1st Qu.:100.19   1st Qu.: 45.59   1st Qu.: 29.5
##  Median :0.2644    Median :120.52   Median : 67.34   Median : 57.0
##  Mean   :0.4088    Mean   :161.14   Mean   :104.11   Mean   : 67.0
##  3rd Qu.:0.4098    3rd Qu.:160.08   3rd Qu.:104.37   3rd Qu.: 74.0
##  Max.   :1.4887    Max.   :457.68   Max.   :379.09   Max.   :215.0
##  Duration.ms._20mean scalar_max_20mean scalar_mean_20mean
##  Min.   :1.663       Min.   :22.50     Min.   :21.81
##  1st Qu.:3.989       1st Qu.:22.84     1st Qu.:22.17
##  Median :4.344       Median :23.45     Median :22.25
##  Mean   :4.264       Mean   :23.43     Mean   :22.31
##  3rd Qu.:5.133       3rd Qu.:23.99     3rd Qu.:22.53
##  Max.   :5.997       Max.   :24.29     Max.   :22.86
```

```
##  changescalar_max_20mean changescalar_mean_20mean Changevector_max_20mean
##  Min.   : 5.234          Min.   : 2.159           Min.   : 7.482
##  1st Qu.: 5.531          1st Qu.: 2.780           1st Qu.: 9.975
##  Median : 7.212          Median : 3.869           Median :11.368
##  Mean   : 7.824          Mean   : 4.883           Mean   :12.189
##  3rd Qu.: 8.123          3rd Qu.: 5.590           3rd Qu.:12.832
##  Max.   :14.139          Max.   :12.105           Max.   :19.387
##  Changevector_mean_20mean Angle_max_20mean Angle_mean_20mean Hz_max_20mean
##  Min.   : 4.828           Min.   :0.3096   Min.   :0.2178    Min.   :
0.5016
##  1st Qu.: 5.527           1st Qu.:0.4961   1st Qu.:0.2321    1st
Qu.:114.2034
##  Median : 6.843           Median :0.5214   Median :0.3023    Median
:132.7735
##  Mean   : 7.865           Mean   :0.6046   Mean   :0.3945    Mean
:142.5937
##  3rd Qu.: 8.273           3rd Qu.:0.5788   3rd Qu.:0.3688    3rd
Qu.:147.3906
##  Max.   :17.154           Max.   :1.2518   Max.   :1.0993    Max.
:318.7755
##  Hz_mean_20mean        RH._Max         Temp_Max        RH._mean
##  Min.   :  0.3079   Min.   :47.08   Min.   :19.25   Min.   :41.69
##  1st Qu.: 56.3355   1st Qu.:60.76   1st Qu.:24.53   1st Qu.:50.83
##  Median : 75.5923   Median :73.52   Median :27.38   Median :71.53
##  Mean   : 93.4190   Mean   :69.60   Mean   :26.80   Mean   :62.64
##  3rd Qu.: 93.9064   3rd Qu.:78.33   3rd Qu.:29.48   3rd Qu.:72.73
##  Max.   :279.9378   Max.   :84.59   Max.   :32.00   Max.   :77.90
##    Temp_mean
##  Min.   :10.22
##  1st Qu.:12.61
##  Median :15.36
##  Mean   :17.33
##  3rd Qu.:22.23
##  Max.   :24.58
```

```
str(tab)
```

```
## 'data.frame':    22 obs. of  110 variables:
##  $ Date                     : int  170510 170510 170517 170517 170524
170524 170531 170531 170607 170607 ...
##  $ Replicate                : chr  "R1" "R1" "R2" "R2" ...
##  $ Age                      : int  22 22 22 22 22 22 22 22 22 22 ...
##  $ Treatments               : chr  "Shipped-110Gy " "Shipped-0Gy "
"Shipped-110Gy " "Shipped-0Gy " ...
##  $ not_full                 : int  2 4 22 13 6 22 4 14 11 17 ...
##  $ full                     : int  6 6 5 17 7 4 0 9 8 22 ...
##  $ MSV                      : num  0.75 0.7 0.41 0.66 0.6 0.42 0.25 0.57
0.47 0.66 ...
##  $ MSV2                     : num  1.5 1.4 0.82 1.32 1.2 0.84 0.5 1.14 0.94
1.32 ...
```

```
##  $ shipped                  : chr  "yes" "yes" "yes" "yes" ...
##  $ Irradiation              : chr  "yes" "no" "yes" "no" ...
##  $ Events._5max             : int  909 909 708 708 1187 1187 625 625 606
606 ...
##  $ Duration.ms._5max        : num  48.8 48.8 87.5 87.5 93.1 ...
##  $ scalar_max_5max          : num  27.4 27.4 27.4 27.4 27.4 ...
##  $ scalar_mean_5max         : num  21.4 21.4 16.9 16.9 15.4 ...
##  $ changescalar_max_5max    : num  23.7 23.7 24.5 24.5 16.6 ...
##  $ changescalar_mean_5max   : num  8.95 8.95 11.38 11.38 3.84 ...
##  $ Changevector_max_5max    : num  27.7 27.7 35.1 35.1 33 ...
##  $ Changevector_mean_5max   : num  9.94 9.94 19.04 19.04 5.06 ...
##  $ Angle_max_5max           : num  3 3 2.97 2.97 2.11 ...
##  $ Angle_mean_5max          : num  2.62 2.62 1.68 1.68 1.36 ...
##  $ Hz_max_5max              : num  764 764 756 756 536 ...
##  $ Hz_mean_5max             : num  666 666 428 428 345 ...
##  $ Events_10max             : int  288 288 315 315 238 238 261 261 219 219
...
##  $ Duration.ms._10max       : num  36.9 36.9 50 50 63.8 ...
##  $ scalar_max_10max         : num  27.4 27.4 27.4 27.4 27.4 ...
##  $ scalar_mean_10max        : num  21.6 21.6 20.4 20.4 19.7 ...
##  $ changescalar_max_10max   : num  23.7 23.7 24.5 24.5 16.6 ...
##  $ changescalar_mean_10max  : num  12.47 12.47 16.49 16.49 8.97 ...
##  $ Changevector_max_10max   : num  27.7 27.7 35.1 35.1 33 ...
##  $ Changevector_mean_10max  : num  15.5 15.5 22.6 22.6 10.7 ...
##  $ Angle_max_10max          : num  2.69 2.69 2.97 2.97 2.11 ...
##  $ Angle_mean_10max         : num  1.047 1.047 1.614 1.614 0.585 ...
##  $ Hz_max_10max             : num  684 684 756 756 536 ...
##  $ Hz_mean_10max            : num  267 267 411 411 149 ...
##  $ Events_15max             : int  144 144 183 183 111 111 163 163 129 129
...
##  $ Duration.ms._15max       : num  30.6 30.6 29.4 29.4 41.2 ...
##  $ scalar_max_15max         : num  27.4 27.4 27.4 27.4 27.4 ...
##  $ scalar_mean_15max        : num  22.4 22.4 21.7 21.7 22.8 ...
##  $ changescalar_max_15max   : num  23.7 23.7 24.5 24.5 16.6 ...
##  $ changescalar_mean_15max  : num  15.7 15.7 16.5 16.5 11.5 ...
##  $ Changevector_max_15max   : num  27.7 27.7 35.1 35.1 33 ...
##  $ Changevector_mean_15max  : num  19.8 19.8 21.6 21.6 12.8 ...
##  $ Angle_max_15max          : num  2.69 2.69 2.97 2.97 2.11 ...
##  $ Angle_mean_15max         : num  1.843 1.843 1.601 1.601 0.671 ...
##  $ Hz_max_15max             : num  684 684 756 756 536 ...
##  $ Hz_mean_15max            : num  469 469 408 408 171 ...
##  $ Events_20max             : int  57 57 75 75 42 42 97 97 71 71 ...
##  $ Duration.ms._20max       : num  17.5 17.5 20.6 20.6 20.6 ...
##  $ scalar_max_20max         : num  27.4 27.4 27.4 27.4 27.4 ...
##  $ scalar_mean_20max        : num  26.8 26.8 26.4 26.4 25.2 ...
##  $ changescalar_max_20max   : num  23.7 23.7 24.5 24.5 13.5 ...
##  $ changescalar_mean_20max  : num  23.72 23.72 13.71 13.71 6.61 ...
##  $ Changevector_max_20max   : num  27.7 27.7 35.1 35.1 33 ...
##  $ Changevector_mean_20max  : num  27.7 27.7 29.7 29.7 11.2 ...
##  $ Angle_max_20max          : num  2.69 2.69 2.43 2.43 1.72 ...
```

```
##  $ Angle_mean_20max        : num  2.687 2.687 1.793 1.793 0.489 ...
##  $ Hz_max_20max            : num  684 684 620 620 437 ...
##  $ Hz_mean_20max           : num  684 684 457 457 125 ...
##  $ Events_5mean            : int  909 909 708 708 1187 1187 625 625 606
606 ...
##  $ Duration.ms._5mean      : num  5.22 5.22 11.79 11.79 7.35 ...
##  $ scalar_max_5mean        : num  9.52 9.52 10.55 10.55 8.02 ...
##  $ scalar_mean_5mean       : num  7.47 7.47 7.57 7.57 6.51 ...
##  $ changescalar_max_5mean  : num  3.76 3.76 2.82 2.82 1.63 ...
##  $ changescalar_mean_5mean : num  1.66 1.66 1.01 1.01 0.68 ...
##  $ Changevector_max_5mean  : num  4.5 4.5 3.69 3.69 2.29 ...
##  $ Changevector_mean_5mean : num  2.22 2.22 1.64 1.64 1.19 ...
##  $ Angle_max_5mean         : num  0.43 0.43 0.372 0.372 0.254 ...
##  $ Angle_mean_5mean        : num  0.176 0.176 0.141 0.141 0.131 ...
##  $ Hz_max_5mean            : num  109.4 109.4 94.6 94.6 64.7 ...
##  $ Hz_mean_5mean           : num  44.7 44.7 36 36 33.4 ...
##  $ Events._10mean          : int  288 288 315 315 238 238 261 261 219 219
...
##  $ Duration.ms._10mean     : num  3.89 3.89 7.71 7.71 7.42 ...
##  $ scalar_max_10mean       : num  15.5 15.5 16.4 16.4 15.6 ...
##  $ scalar_mean_10mean      : num  13.5 13.5 13.7 13.7 13.2 ...
##  $ changescalar_max_10mean : num  6.56 6.56 4.93 4.93 4 ...
##  $ changescalar_mean_10mean: num  3.25 3.25 1.97 1.97 1.66 ...
##  $ Changevector_max_10mean : num  8.03 8.03 6.51 6.51 5.89 ...
##  $ Changevector_mean_10mean: num  4.54 4.54 3.34 3.34 2.91 ...
##  $ Angle_max_10mean        : num  0.472 0.472 0.405 0.405 0.349 ...
##  $ Angle_mean_10mean       : num  0.222 0.222 0.175 0.175 0.158 ...
##  $ Hz_max_10mean           : num  120.2 120.2 103.1 103.1 88.9 ...
##  $ Hz_mean_10mean          : num  56.7 56.7 44.6 44.6 40.3 ...
##  $ Events_15mean           : int  144 144 183 183 111 111 163 163 129 129
...
##  $ Duration.ms._15mean     : num  3.44 3.44 6.34 6.34 6.92 ...
##  $ scalar_max_15mean       : num  19.3 19.3 19.9 19.9 19.7 ...
##  $ scalar_mean_15mean      : num  17.9 17.9 17.8 17.8 17.6 ...
##  $ changescalar_max_15mean : num  8.2 8.2 6.27 6.27 5.29 ...
##  $ changescalar_mean_15mean: num  4.21 4.21 2.42 2.42 2.06 ...
##  $ Changevector_max_15mean : num  10.15 10.15 8.77 8.77 8.64 ...
##  $ Changevector_mean_15mean: num  6.19 6.19 4.49 4.49 4.18 ...
##  $ Angle_max_15mean        : num  0.473 0.473 0.458 0.458 0.444 ...
##  $ Angle_mean_15mean       : num  0.264 0.264 0.204 0.204 0.196 ...
##  $ Hz_max_15mean           : num  121 121 117 117 113 ...
##  $ Hz_mean_15mean          : num  67.3 67.3 52 52 49.9 ...
##  $ Events_20mean           : int  57 57 75 75 42 42 97 97 71 71 ...
##  $ Duration.ms._20mean     : num  2.59 2.59 5.25 5.25 6 ...
##  $ scalar_max_20mean       : num  22.8 22.8 23.7 23.7 24.3 ...
##  $ scalar_mean_20mean      : num  22 22 22.3 22.3 22.6 ...
##  $ changescalar_max_20mean : num  7.5 7.5 6.19 6.19 6 ...
##   [list output truncated]
```

```
tab$pcinsemination <- tab$full / (tab$full+tab$not_full)
boxplot(tab$pcinsemination ~ tab$Treatments, xlab ="Treatments", ylab =
"Insemination rate")
```

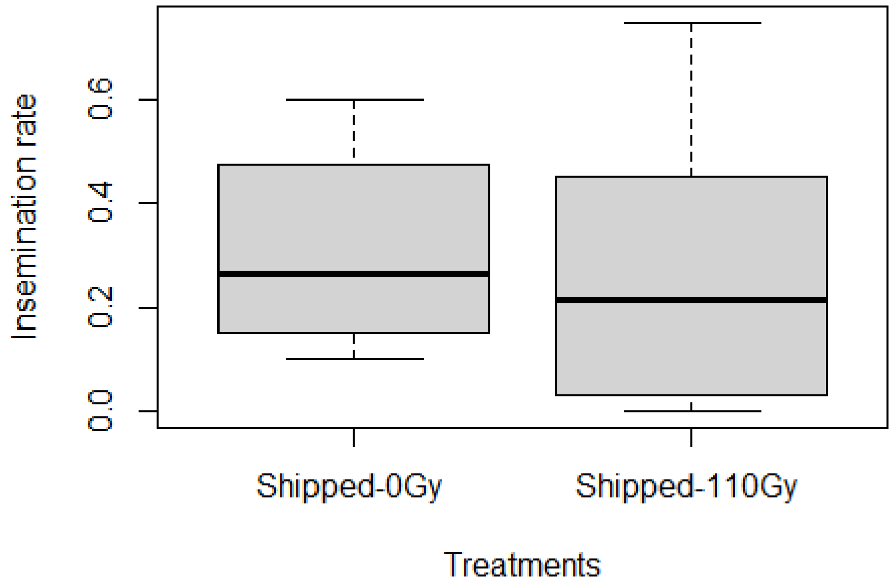


```
#####SCATTER PLOTS

plot(tab$pcinsemination~tab$RH._Max)
```

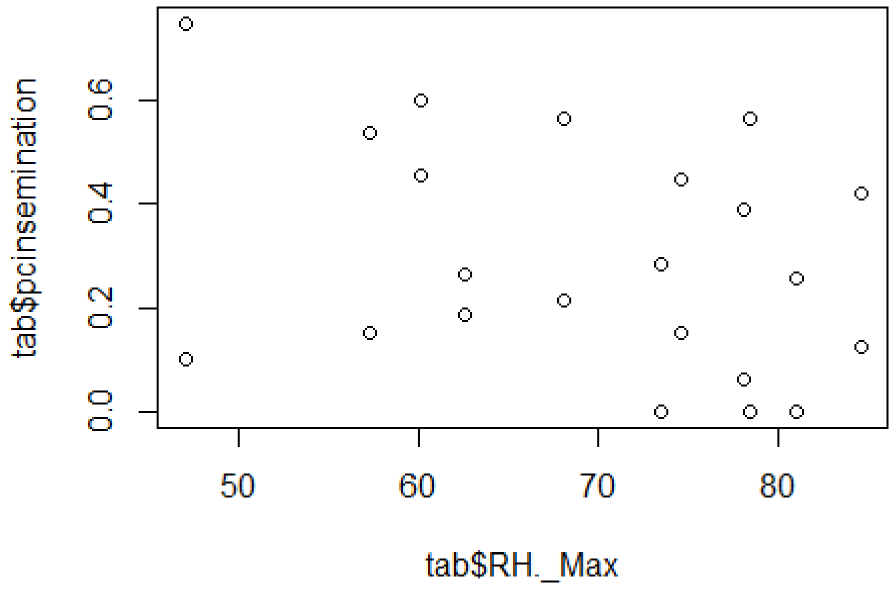


```
plot(tab$pcinsemination~tab$RH._mean)#*
```

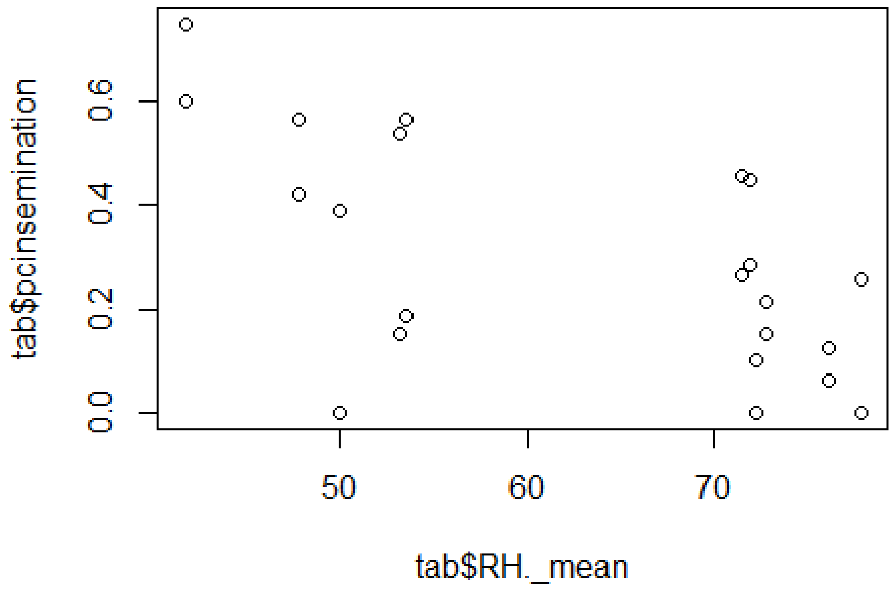


```
plot(tab$pcinsemination~tab$Temp_mean)#*
```

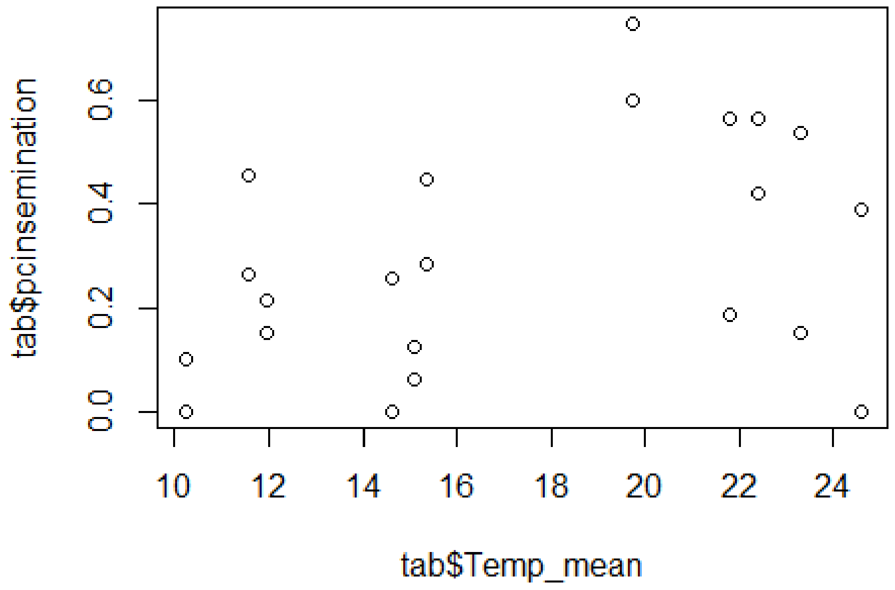


```
cor.test(tab$pcinsemination,tab$Temp_mean)

##
##  Pearson's product-moment correlation
##
## data:  tab$pcinsemination and tab$Temp_mean
## t = 1.8905, df = 20, p-value = 0.07327
## alternative hypothesis: true correlation is not equal to 0
## 95 percent confidence interval:
##  -0.03856667  0.69662194
## sample estimates:
##      cor
## 0.389373

plot(tab$pcinsemination~tab$Temp_Max)
```

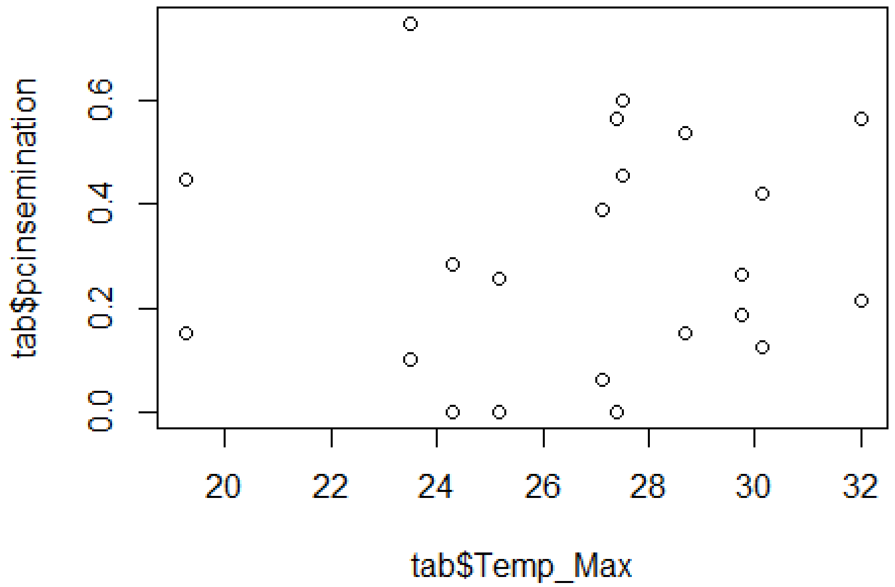


```
par(mfrow = c(2,2))

#Scatter plots at threshold 5-Max
plot(tab$pcinsemination~tab$Events._5max)#*
plot(tab$pcinsemination~tab$Duration.ms._5max)
plot(tab$pcinsemination~tab$scalar_max_5max)
plot(tab$pcinsemination~tab$scalar_mean_5max)
```

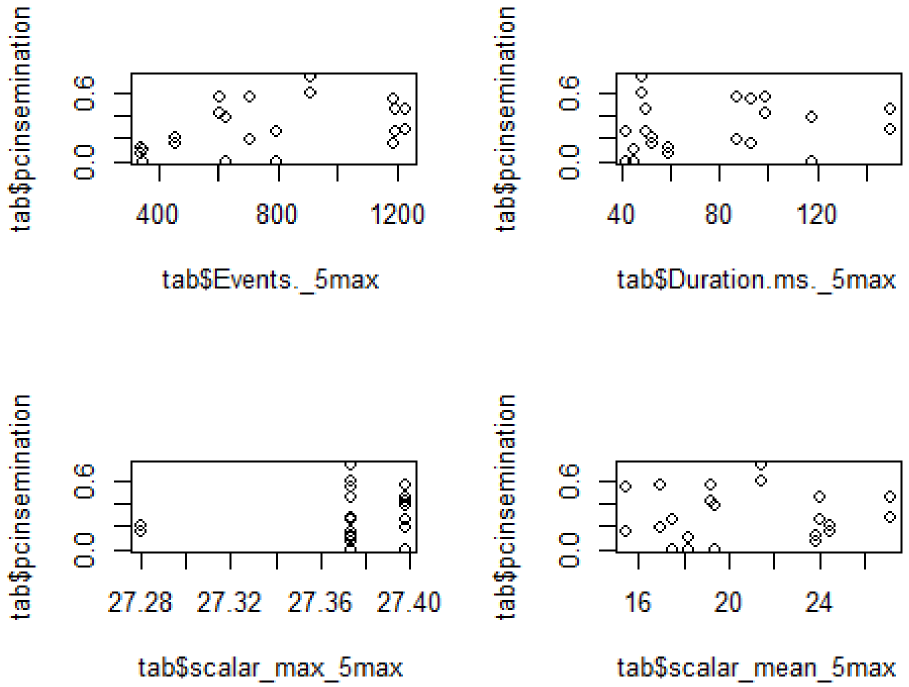


```
plot(tab$pcinsemination~tab$changescalar_max_5max)
plot(tab$pcinsemination~tab$changescalar_mean_5max)#*
plot(tab$pcinsemination~tab$Changevector_max_5max)
plot(tab$pcinsemination~tab$Changevector_mean_5max)#*
```

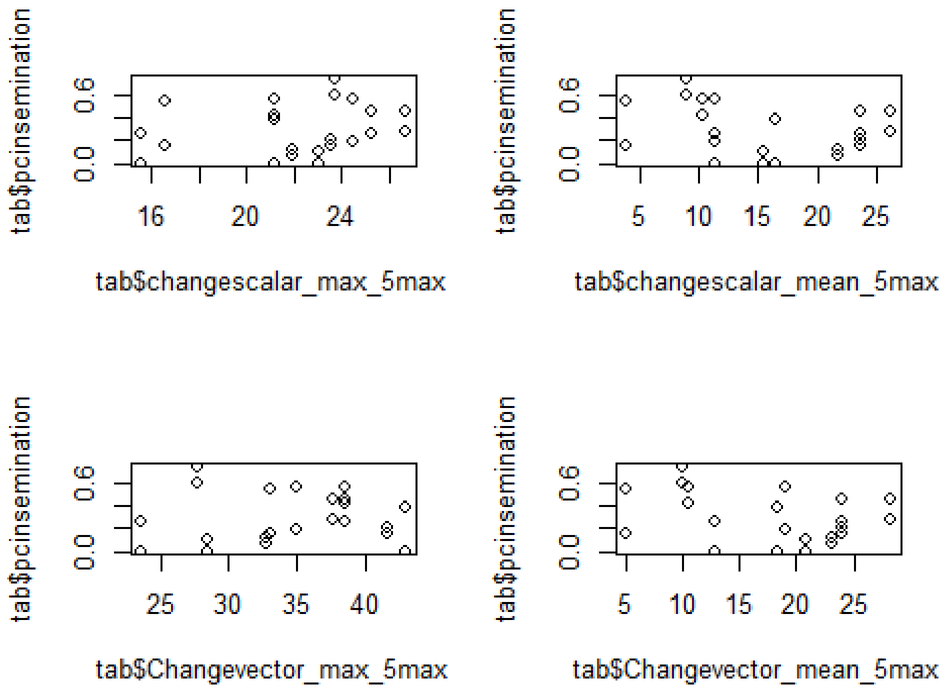


```
plot(tab$pcinsemination~tab$Angle_max_5max)
plot(tab$pcinsemination~tab$Angle_mean_5max)
plot(tab$pcinsemination~tab$Hz_max_5max)
plot(tab$pcinsemination~tab$Hz_mean_5max)
```

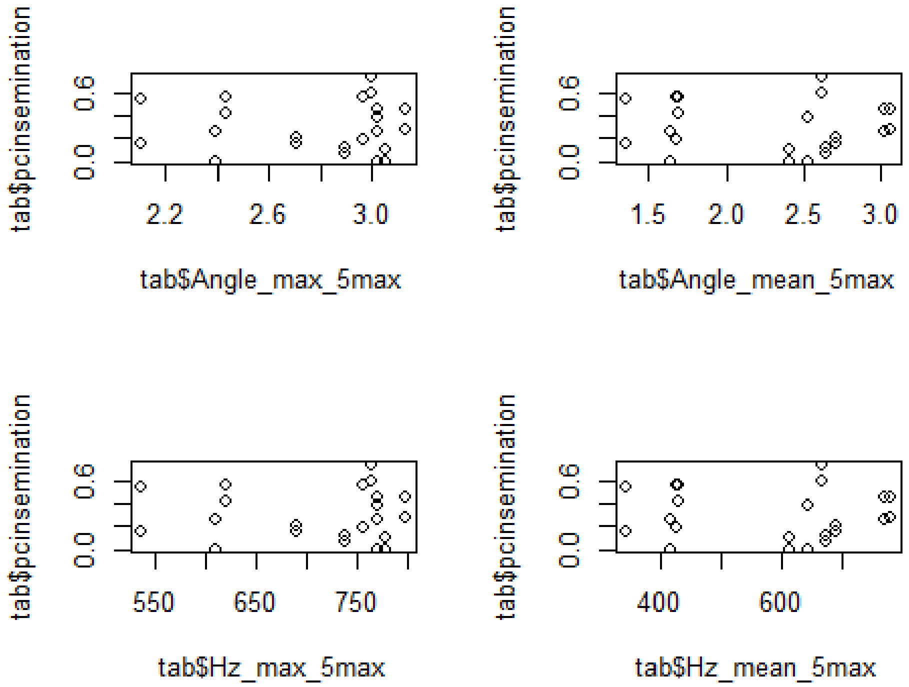

#Scatter plots at threshold 5-Mean

```
par(mfrow = c(2,2))
plot(tab$pcinsemination~tab$Duration.ms._5mean)#*
plot(tab$pcinsemination~tab$scalar_max_5mean)
plot(tab$pcinsemination~tab$scalar_mean_5mean)
plot(tab$pcinsemination~tab$changescalar_max_5mean)
```

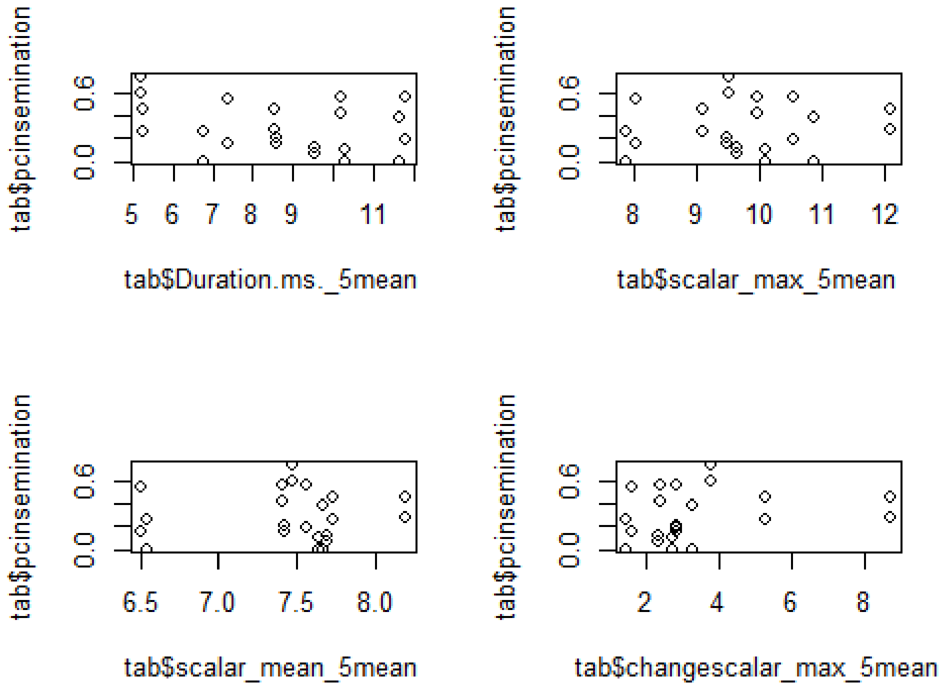


```
plot(tab$pcinsemination~tab$changescalar_mean_5mean)
plot(tab$pcinsemination~tab$Changevector_max_5mean)
plot(tab$pcinsemination~tab$Changevector_mean_5mean)
plot(tab$pcinsemination~tab$Angle_max_5mean)
```

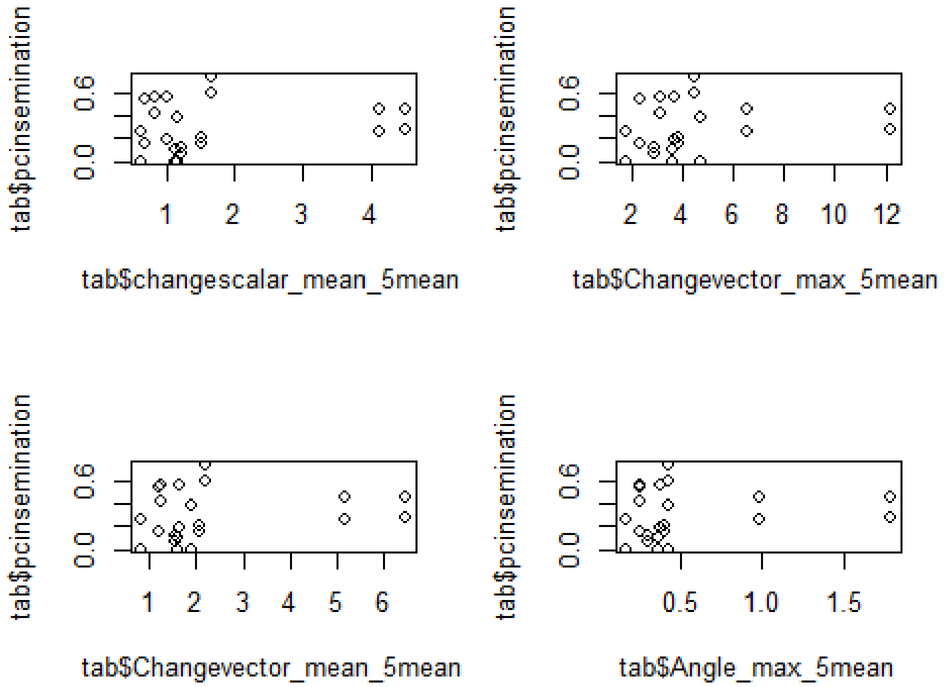


```
plot(tab$pcinsemination~tab$Angle_mean_5mean)
plot(tab$pcinsemination~tab$Hz_max_5mean)
plot(tab$pcinsemination~tab$Hz_mean_5mean)
```

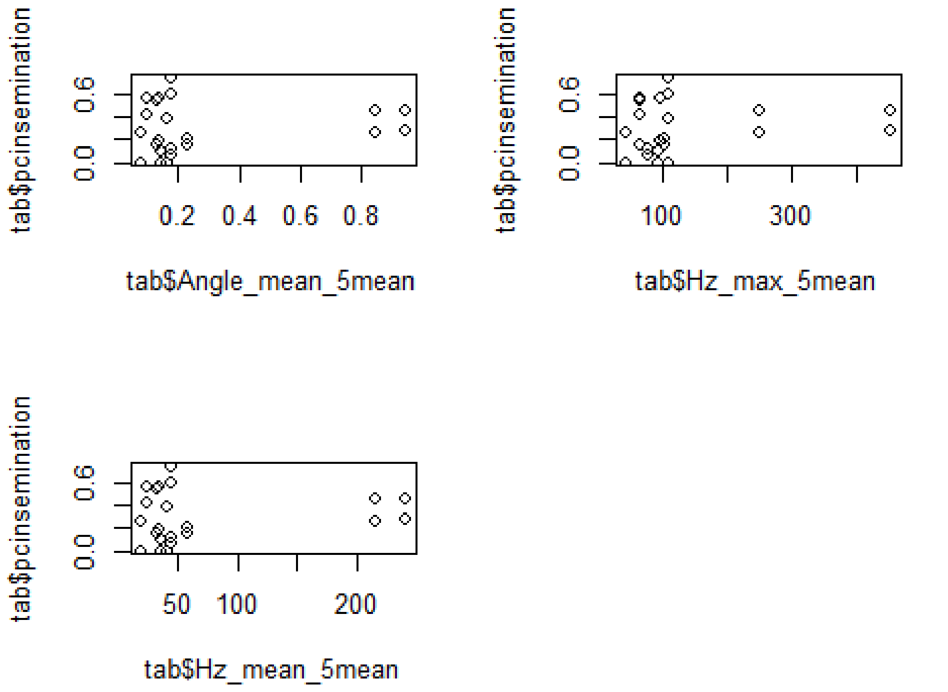

#Scatter plots at threshold 10-Max

```
par(mfrow = c(2,2))
plot(tab$pcinsemination~tab$Duration.ms._10max)#
plot(tab$pcinsemination~tab$Duration.ms._10max)
plot(tab$pcinsemination~tab$scalar_max_10max)
plot(tab$pcinsemination~tab$scalar_mean_10max)#*
```

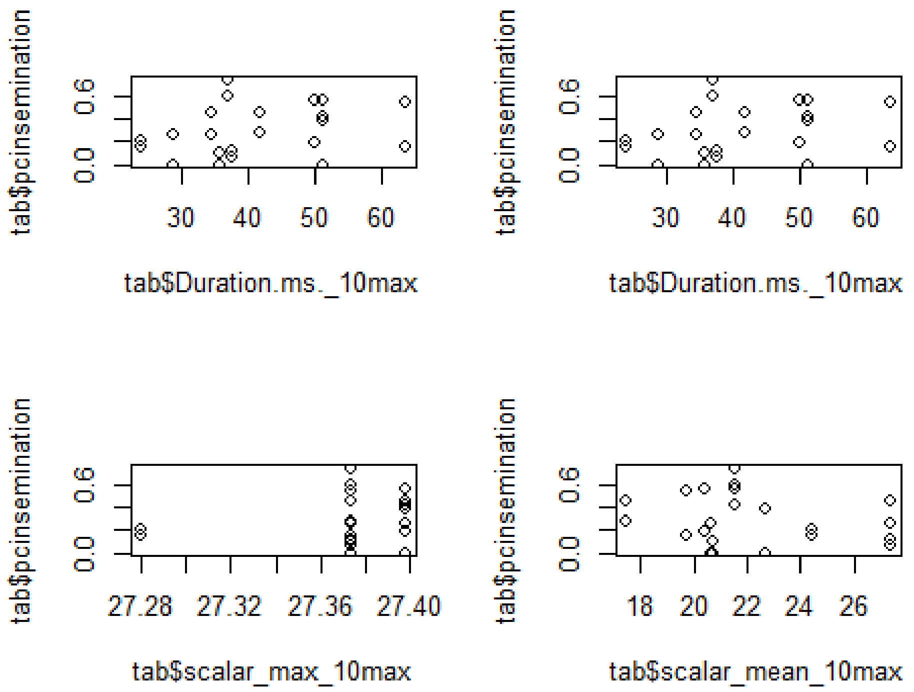


```
plot(tab$pcinsemination~tab$changescalar_max_10max)
plot(tab$pcinsemination~tab$changescalar_mean_10max)#*
cor.test(tab$pcinsemination,tab$changescalar_mean_10max)

##
##  Pearson's product-moment correlation
##
## data:  tab$pcinsemination and tab$changescalar_mean_10max
## t = -1.4609, df = 20, p-value = 0.1596
## alternative hypothesis: true correlation is not equal to 0
## 95 percent confidence interval:
##  -0.647380  0.127815
## sample estimates:
##        cor
## -0.3105271

plot(tab$pcinsemination~tab$Changevector_max_10max)
plot(tab$pcinsemination~tab$Changevector_mean_10max)#*
```

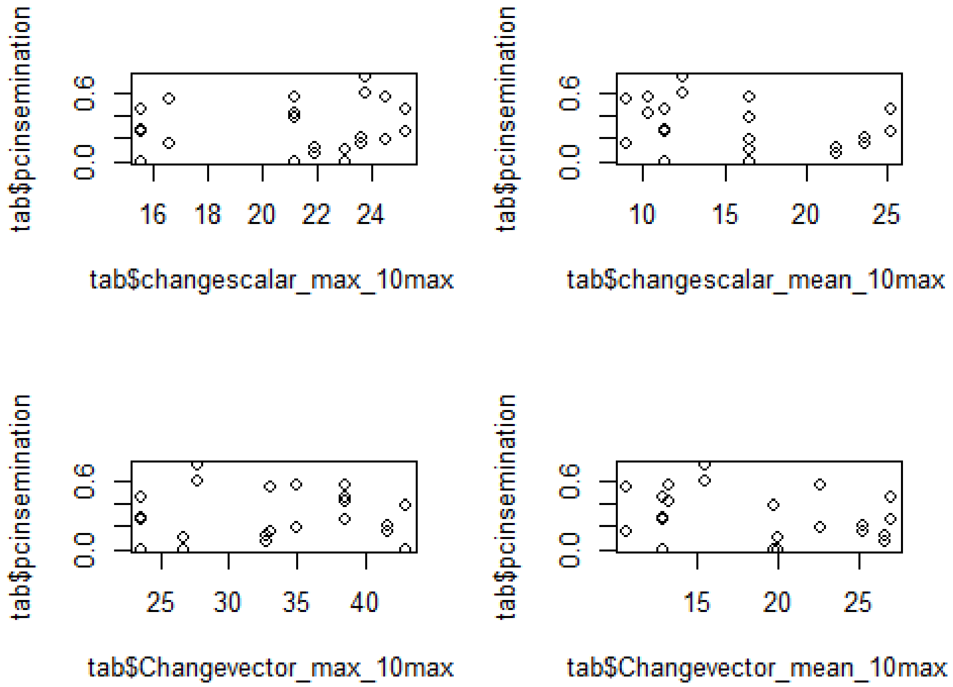


```
plot(tab$pcinsemination~tab$Angle_max_10max)
plot(tab$pcinsemination~tab$Angle_mean_10max)#*
plot(tab$pcinsemination~tab$Hz_max_10max)
plot(tab$pcinsemination~tab$Hz_mean_10max)#*
```

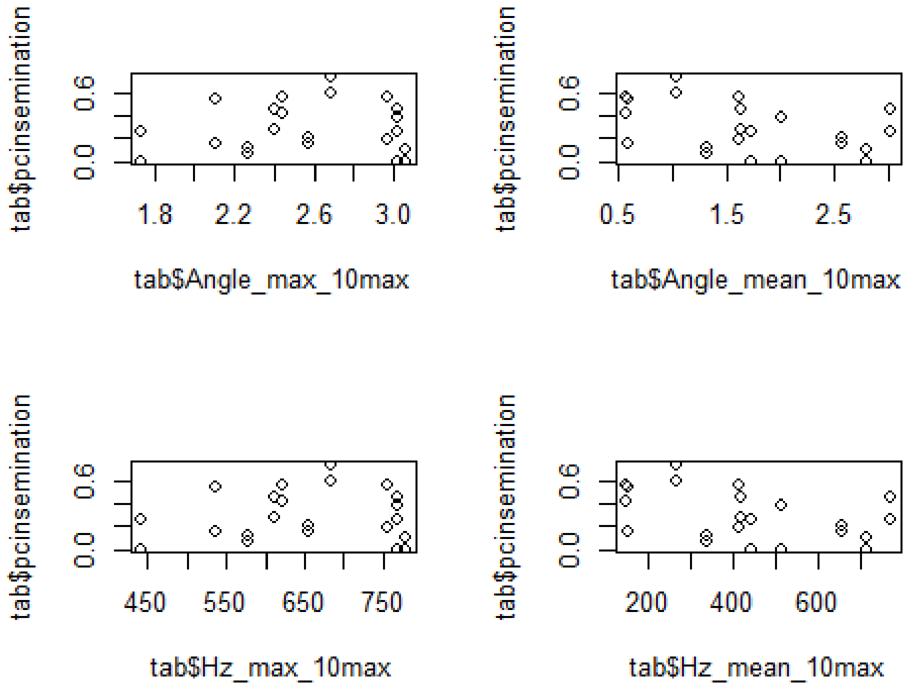

#Scatter plots at threshold 10-Mean

```
par(mfrow = c(2,2))
plot(tab$pcinsemination~tab$Duration.ms._10mean)#*
cor.test(tab$pcinsemination,tab$Duration.ms._10mean)

##
##  Pearson's product-moment correlation
##
## data:  tab$pcinsemination and tab$Duration.ms._10mean
## t = -2.5455, df = 20, p-value = 0.01926
## alternative hypothesis: true correlation is not equal to 0
## 95 percent confidence interval:
##  -0.7581589 -0.0923141
## sample estimates:
##        cor
## -0.4946698

plot(tab$pcinsemination~tab$scalar_max_10mean)
plot(tab$pcinsemination~tab$scalar_mean_10mean)
plot(tab$pcinsemination~tab$changescalar_max_10mean)#
```

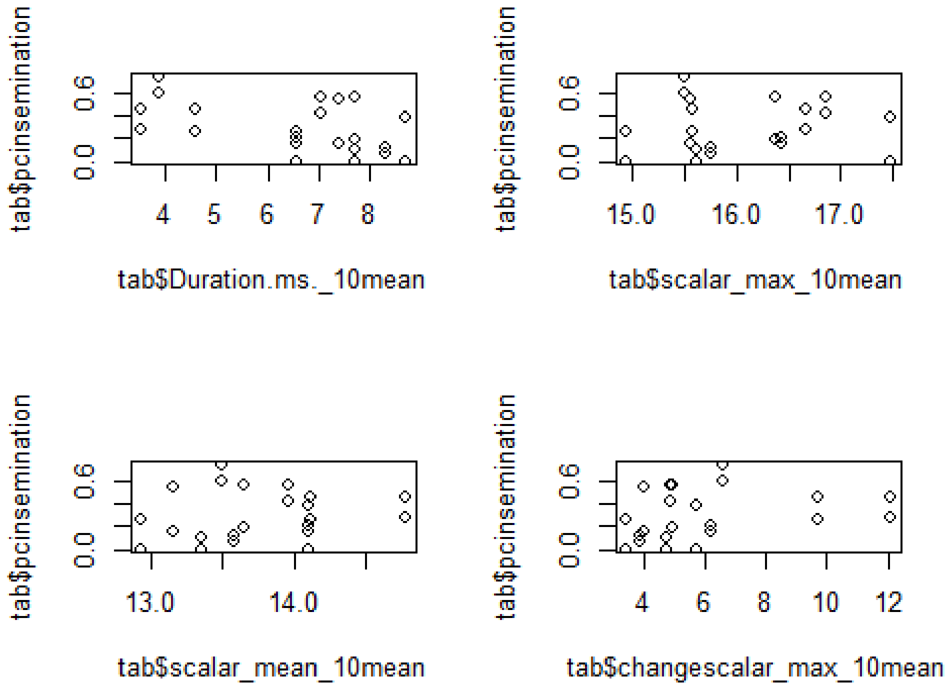


```
plot(tab$pcinsemination~tab$changescalar_mean_10mean)
plot(tab$pcinsemination~tab$Changevector_max_10mean)
plot(tab$pcinsemination~tab$Changevector_mean_10mean)#
plot(tab$pcinsemination~tab$Angle_max_10mean)
```

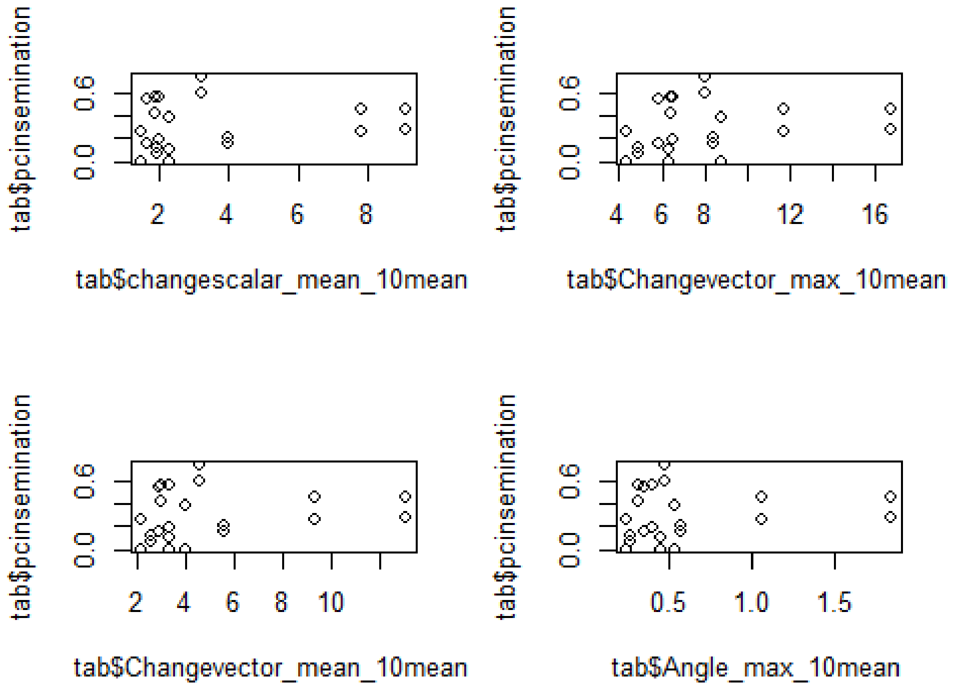


```
plot(tab$pcinsemination~tab$Angle_mean_10mean)
plot(tab$pcinsemination~tab$Hz_max_10mean)
plot(tab$pcinsemination~tab$Hz_mean_10mean)
```

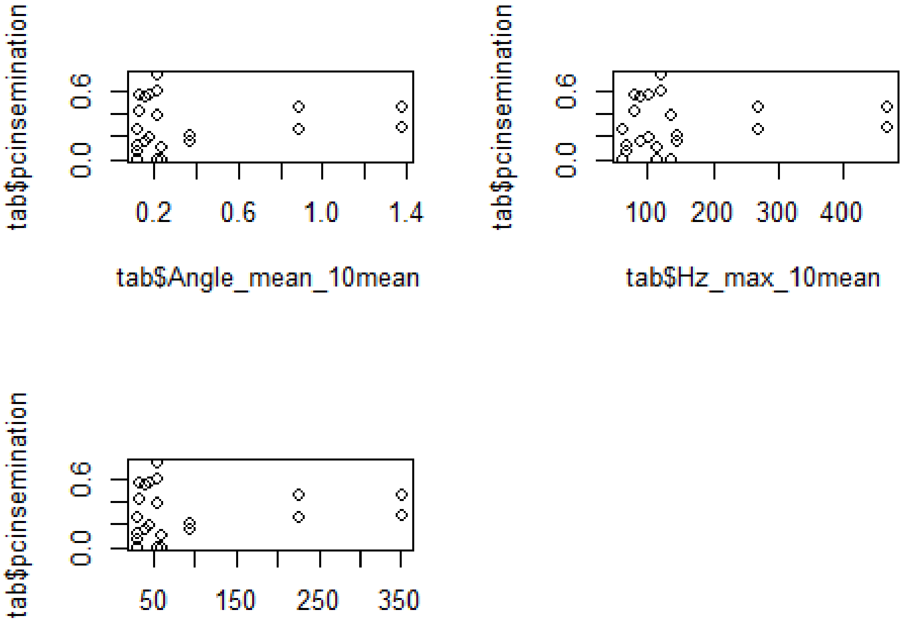

#Scatter plots at threshold 15-Max

```
par(mfrow = c(2,2))
plot(tab$pcinsemination~tab$Events_15max)
plot(tab$pcinsemination~tab$Duration.ms._15max)#*
plot(tab$pcinsemination~tab$scalar_max_15max)
plot(tab$pcinsemination~tab$scalar_mean_15max)
```

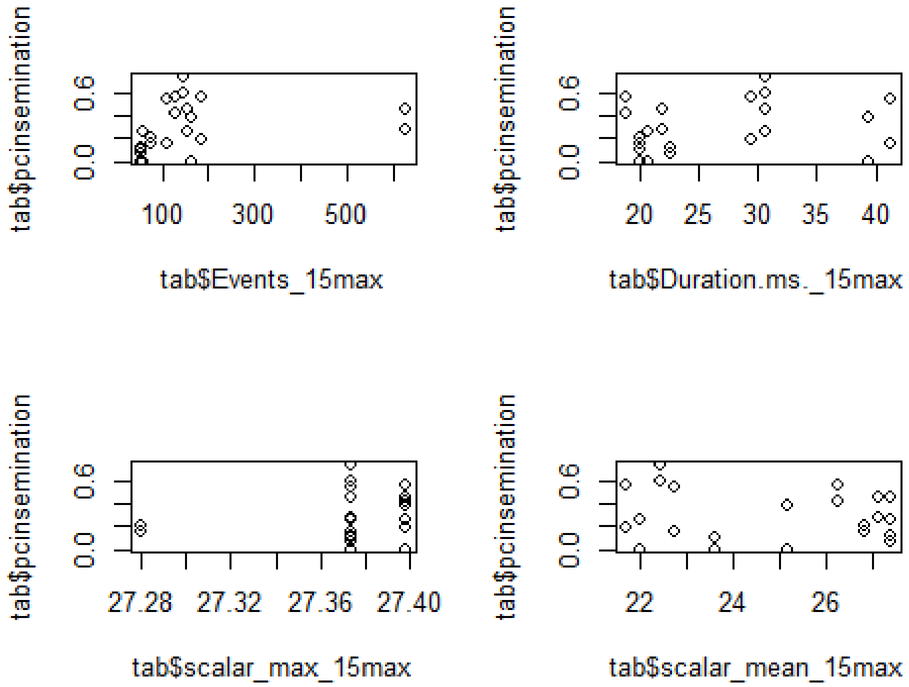


```
plot(tab$pcinsemination~tab$changescalar_max_15max)#*
plot(tab$pcinsemination~tab$changescalar_mean_15max)
plot(tab$pcinsemination~tab$Changevector_max_15max)
plot(tab$pcinsemination~tab$Changevector_mean_15max)
```

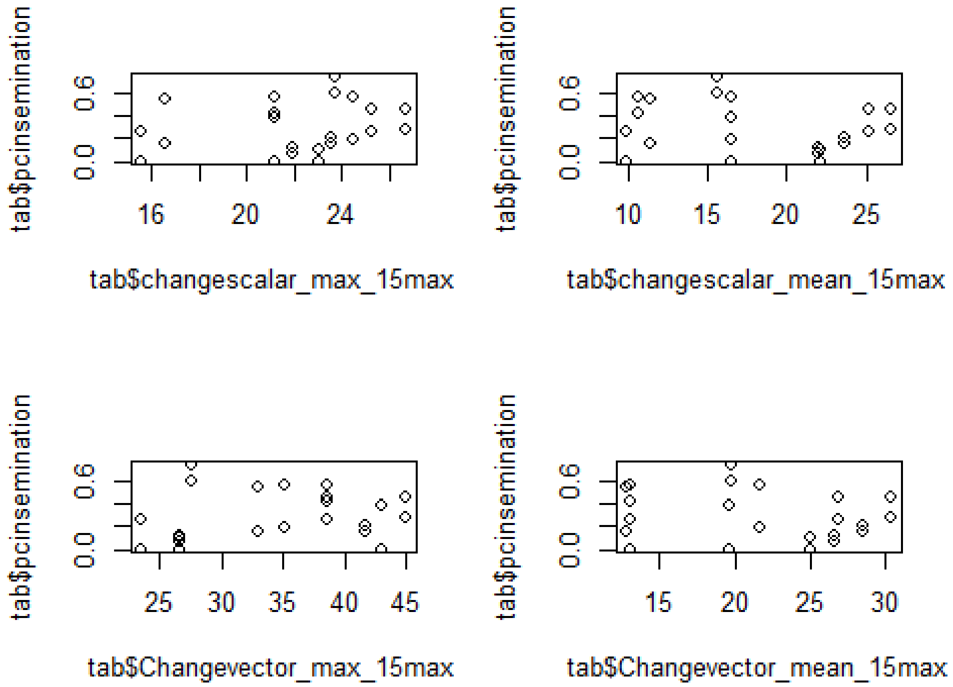


```
plot(tab$pcinsemination~tab$Angle_max_15max)#*
plot(tab$pcinsemination~tab$Angle_mean_15max)
plot(tab$pcinsemination~tab$Hz_max_15max) #*
plot(tab$pcinsemination~tab$Hz_mean_15max)
```

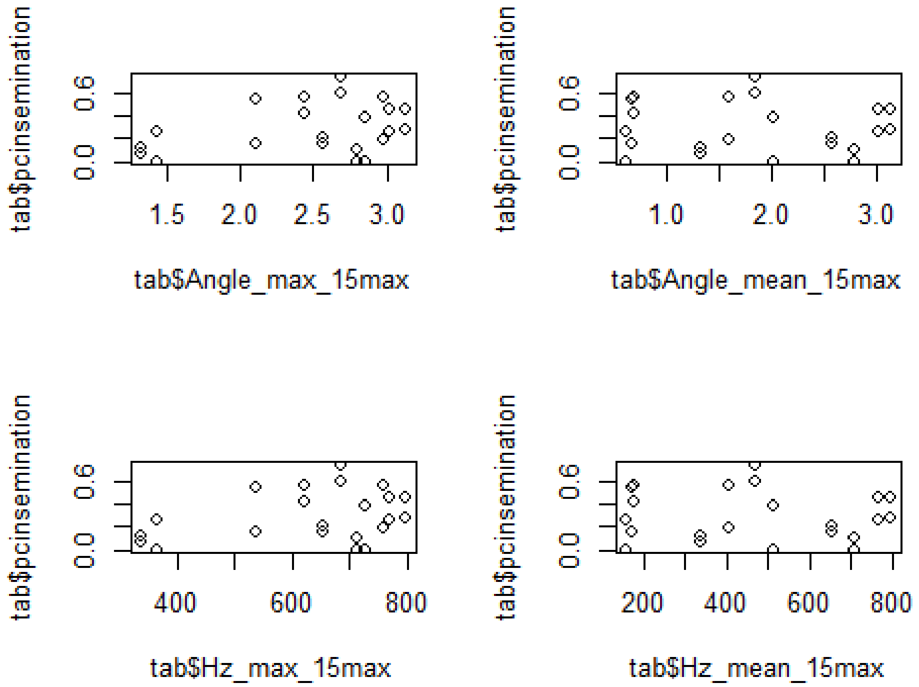

#Scatter plots at threshold 15-Mean

```
par(mfrow = c(2,2))
plot(tab$pcinsemination~tab$Duration.ms._15mean)#*
plot(tab$pcinsemination~tab$scalar_max_15mean)#*
plot(tab$pcinsemination~tab$scalar_mean_15mean)
plot(tab$pcinsemination~tab$changescalar_max_15mean)#*
```

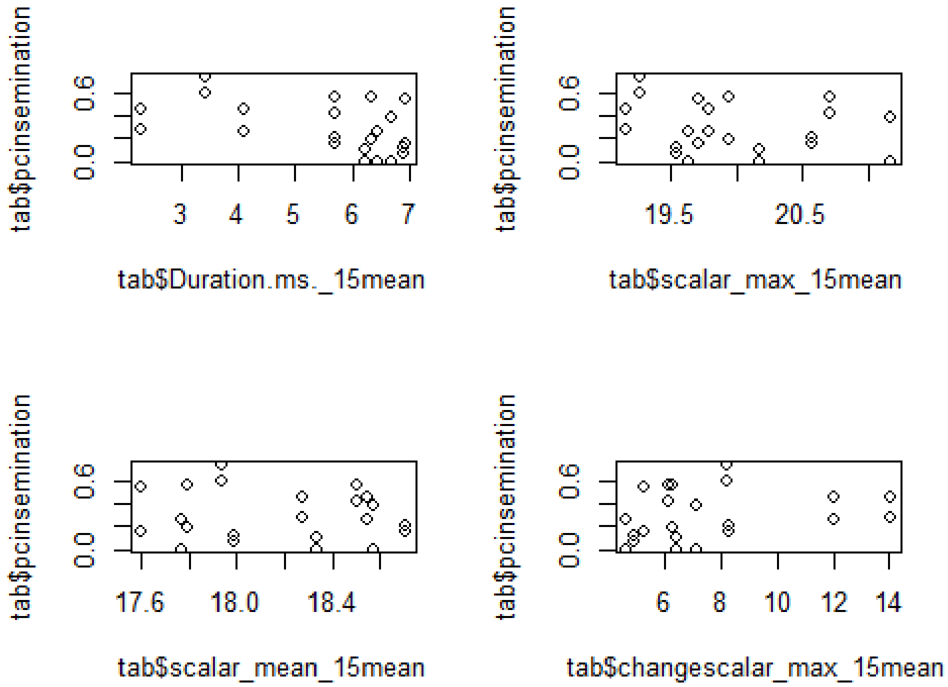


```
plot(tab$pcinsemination~tab$changescalar_mean_15mean)
plot(tab$pcinsemination~tab$Changevector_max_15mean)#*
plot(tab$pcinsemination~tab$Changevector_mean_15mean)
plot(tab$pcinsemination~tab$Angle_max_15mean)
```

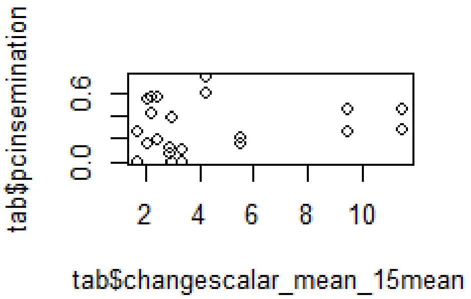

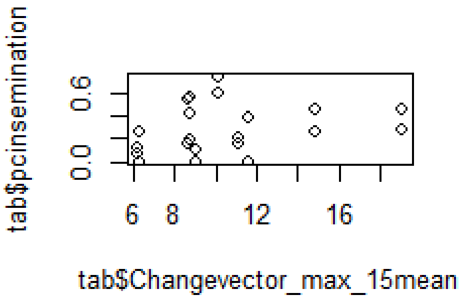

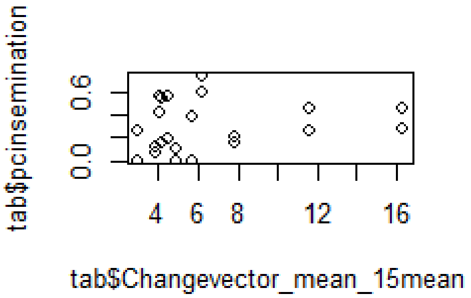

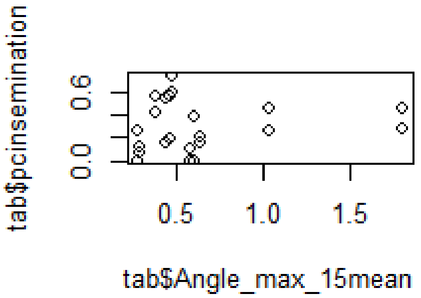


```
plot(tab$pcinsemination~tab$Angle_mean_15mean)
plot(tab$pcinsemination~tab$Hz_max_15mean)
plot(tab$pcinsemination~tab$Hz_mean_15mean)
```

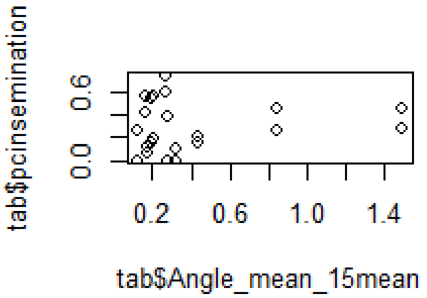

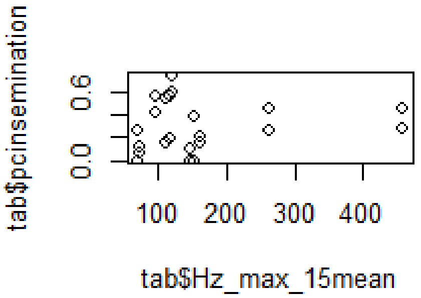

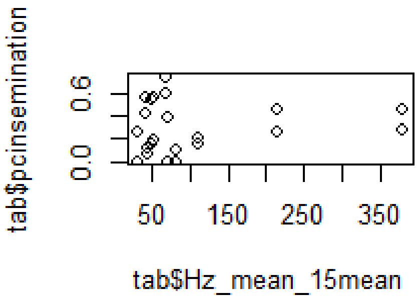

#Scatter plots at threshold 20-Max

```
par(mfrow = c(2,2))
plot(tab$pcinsemination~tab$Events_20max)
plot(tab$pcinsemination~tab$Duration.ms._20max)#*
plot(tab$pcinsemination~tab$scalar_max_20max)
plot(tab$pcinsemination~tab$scalar_mean_20max)
```

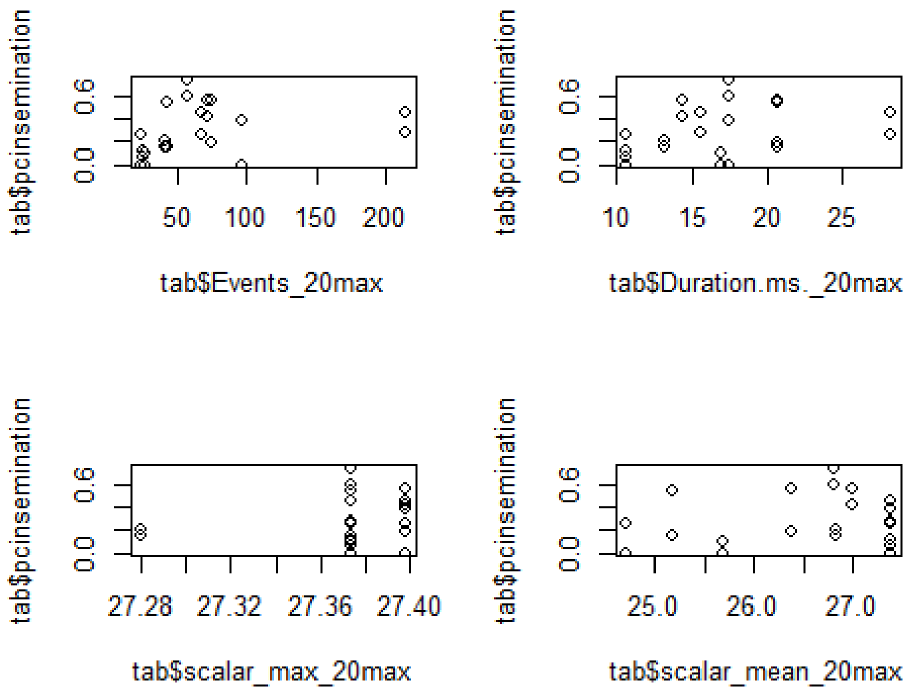


```
plot(tab$pcinsemination~tab$changescalar_max_20max)#*
plot(tab$pcinsemination~tab$changescalar_mean_20max)
plot(tab$pcinsemination~tab$Changevector_max_20max)
plot(tab$pcinsemination~tab$Changevector_mean_20max)
```

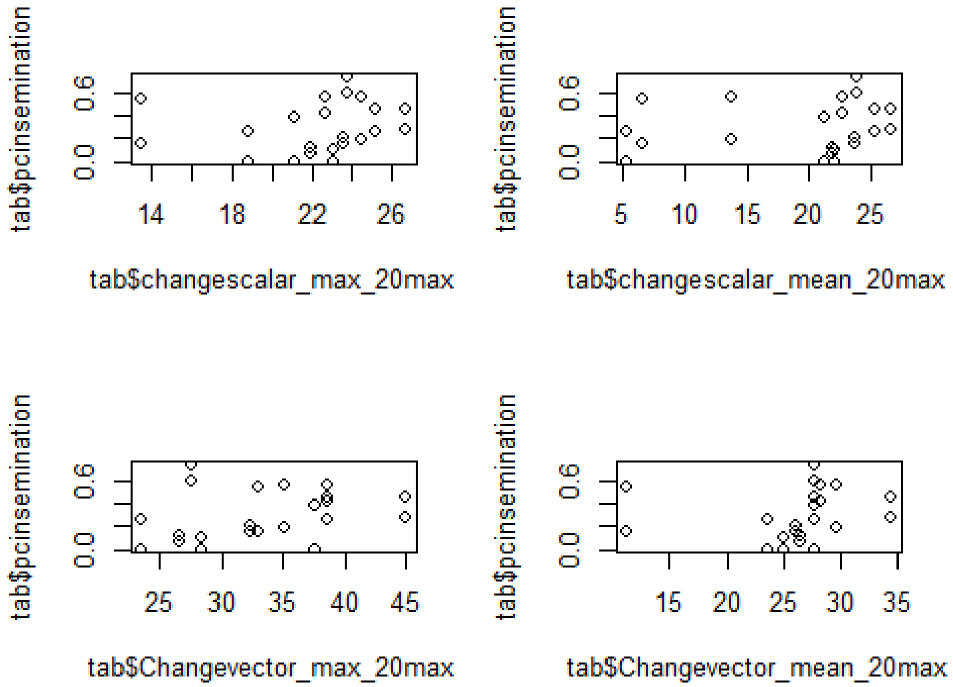


```
plot(tab$pcinsemination~tab$Angle_max_20max)#*
plot(tab$pcinsemination~tab$Angle_mean_20max)#*
plot(tab$pcinsemination~tab$Hz_max_20max)#*
plot(tab$pcinsemination~tab$Hz_mean_20max)#*
```

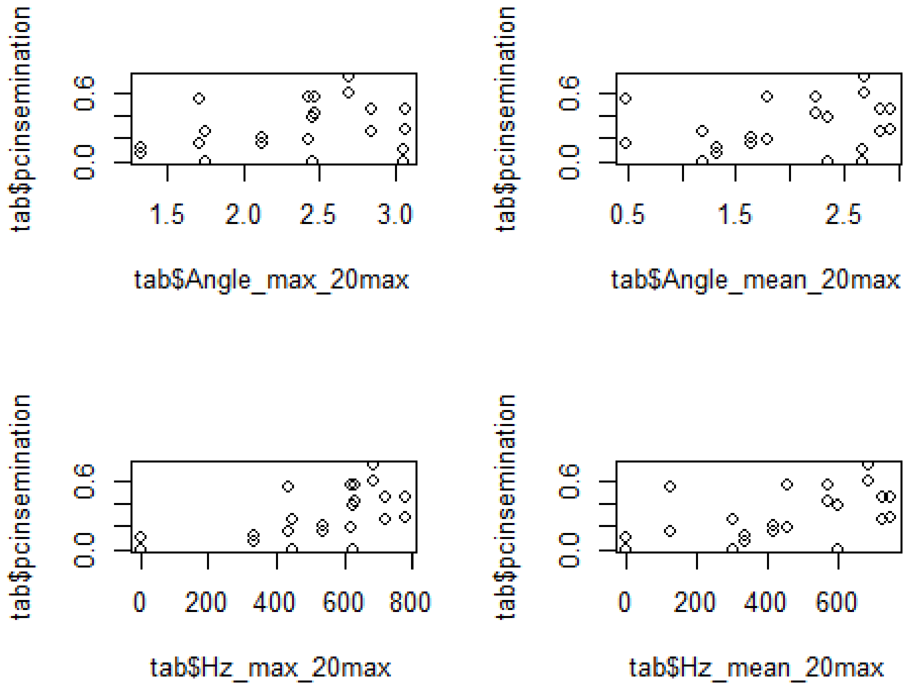

#Scatter plots at threshold 20-Mean

```
par(mfrow = c(2,2))
plot(tab$pcinsemination~tab$Events_20mean)#*
plot(tab$pcinsemination~tab$Duration.ms._20mean)#*
plot(tab$pcinsemination~tab$scalar_max_20mean)
plot(tab$pcinsemination~tab$scalar_mean_20mean)
```

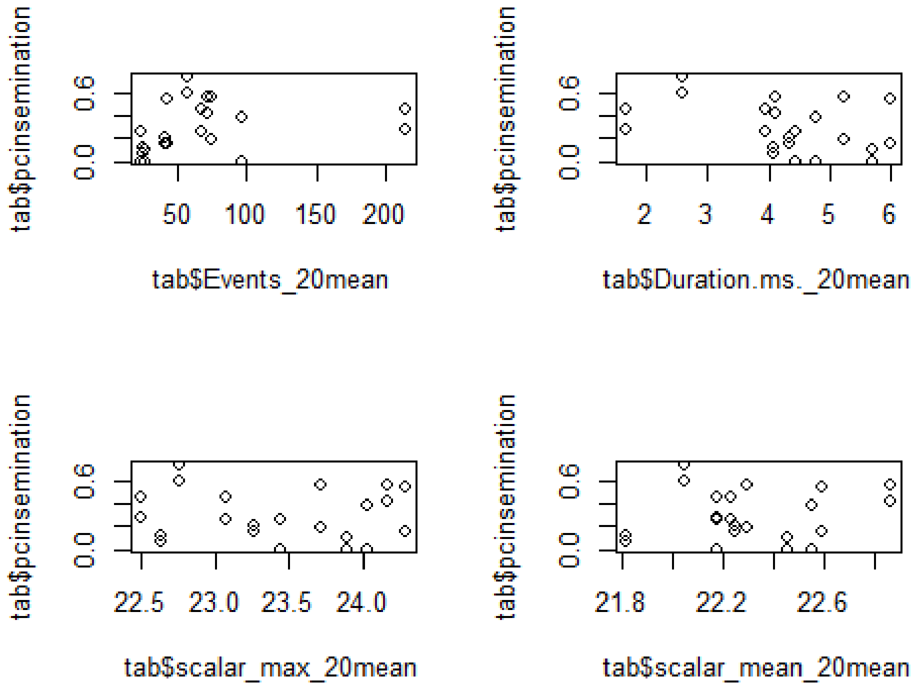


```
plot(tab$pcinsemination~tab$changescalar_max_20mean)
plot(tab$pcinsemination~tab$changescalar_mean_20mean)
plot(tab$pcinsemination~tab$Changevector_max_20mean)#*
plot(tab$pcinsemination~tab$Changevector_mean_20mean)
```

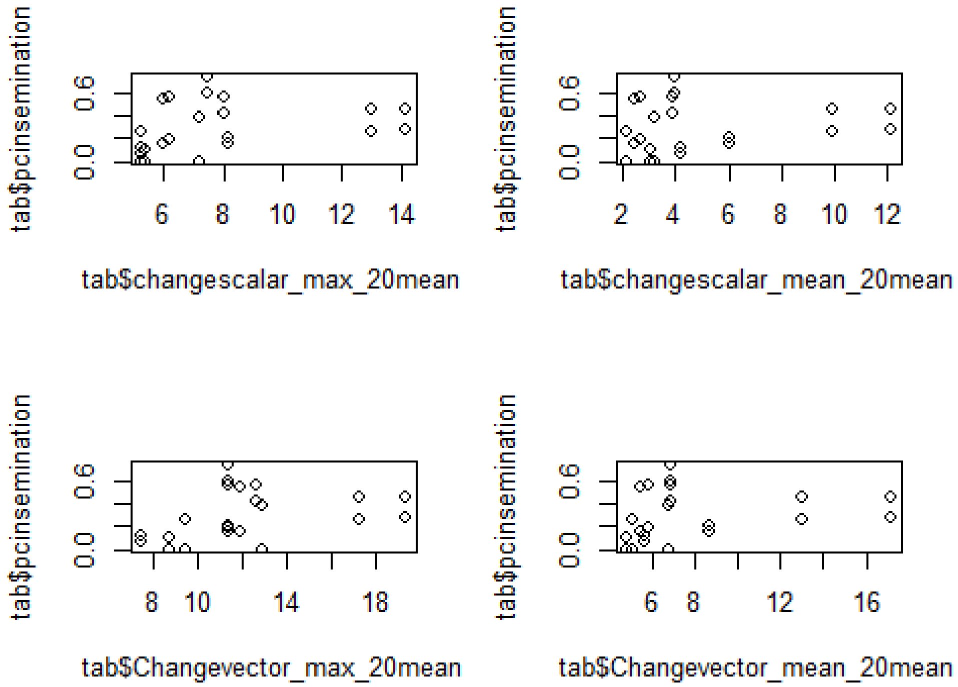


```
plot(tab$pcinsemination~tab$Angle_max_20mean)
plot(tab$pcinsemination~tab$Angle_mean_20mean)
plot(tab$pcinsemination~tab$Hz_max_20mean)#*
plot(tab$pcinsemination~tab$Hz_mean_20mean)
```

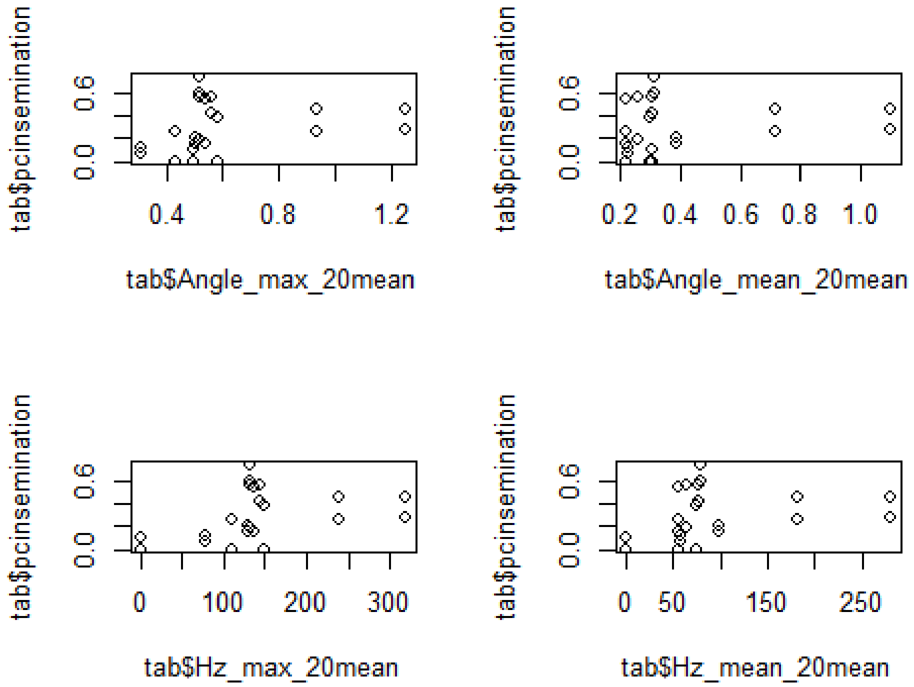

Models of Impact of transportation on the insemination rate

```
tab$Age <- as.factor(tab$Age)


#models threshold 5
fm1 <- glmer(cbind(full,not_full) ~ Irradiation +  RH._Max +Temp_mean +
(1|Replicate), family = binomial, data = tab)
fm2 <- glmer(cbind(full,not_full) ~ Irradiation + RH._Max + Temp_mean +
changescalar_mean_5max + Duration.ms._5mean + (1|Replicate), family =
binomial, data = tab)
fm3 <- glmer(cbind(full,not_full) ~ Irradiation + RH._Max + Age +
Duration.ms._5mean +(1|Replicate), family = binomial, data = tab)
fm4 <- glmer(cbind(full,not_full) ~ Irradiation + RH._Max + Temp_mean +
changescalar_mean_5max + Changevector_max_5max +   (1|Replicate), family =
binomial, data = tab)
fm5 <- glmer(cbind(full,not_full) ~ Irradiation + RH._Max + Temp_mean +
changescalar_mean_5max + Duration.ms._5mean + Changevector_max_5max
+(1|Replicate), family = binomial, data = tab)
fm6 <- glmer(cbind(full,not_full) ~ Irradiation + RH._Max + Temp_mean +
changescalar_mean_5max + (1|Replicate), family = binomial, data = tab)
fm7 <- glmer(cbind(full,not_full) ~ Irradiation + RH._Max + Temp_mean +
Changevector_max_5max +  (1|Replicate), family = binomial, data = tab)
fm8 <- glmer(cbind(full,not_full) ~ Irradiation + Temp_mean +
changescalar_mean_5max + scalar_max_5mean + (1|Replicate), family = binomial,
data = tab)
fm1a <- glmer(cbind(full,not_full) ~ Irradiation + Age+ Temp_mean +
```

```
(1|Replicate), family = binomial, data = tab)
fm1b <- glmer(cbind(full,not_full) ~ Irradiation + Age+ RH._Max +
(1|Replicate), family = binomial, data = tab)

AICc(fm1,fm2,fm3,fm4,fm5,fm6,fm7,fm8,fm1a,fm1b)

##      df     AICc
## fm1   5 142.0019
## fm2   7 134.4797
## fm3   6 134.0154
## fm4   7 144.9677
## fm5   8 138.7653
## fm6   6 140.9644
## fm7   6 141.4632
## fm8   6 150.0441
## fm1a  5 144.9050
## fm1b  5 141.2139

summary(fm3)

## Generalized linear mixed model fit by maximum likelihood (Laplace
##   Approximation) [glmerMod]
##  Family: binomial  ( logit )
## Formula:
## cbind(full, not_full) ~ Irradiation + RH._Max + Age + Duration.ms._5mean +
##     (1 | Replicate)
##    Data: tab
##
##      AIC      BIC   logLik deviance df.resid
##    128.4    135.0    -58.2    116.4       16
##
## Scaled residuals:
##     Min      1Q  Median      3Q     Max
## -2.1383 -1.0707 -0.0861  0.8876  2.4171
##
## Random effects:
##  Groups    Name        Variance Std.Dev.
##  Replicate (Intercept) 0.3672   0.606
## Number of obs: 22, groups:  Replicate, 6
##
## Fixed effects:
##                    Estimate Std. Error z value Pr(>|z|)
## (Intercept)         4.25050    1.62154   2.621  0.00876 **
## Irradiationyes     -0.33327    0.23517  -1.417  0.15644
## RH._Max            -0.03461    0.02027  -1.708  0.08766 .
## Age29              -1.41606    0.28379  -4.990 6.04e-07 ***
## Duration.ms._5mean -0.21754    0.06695  -3.249  0.00116 **
## ---
## Signif. codes:  0 '***' 0.001 '**' 0.01 '*' 0.05 '.' 0.1 ' ' 1
##
```

```
## Correlation of Fixed Effects:
##              (Intr) Irrdtn RH._Mx Age29
## Irradiatnys -0.237
## RH._Max     -0.898  0.175
## Age29       -0.373 -0.037  0.132
## Drtn.ms._5m -0.411  0.086  0.016  0.505
```

```
fm11 <- glmer(cbind(full,not_full) ~ Irradiation +  RH._Max + 
Duration.ms._10max + changescalar_mean_10max+(1|Replicate), family = 
binomial, data = tab)
fm12 <- glmer(cbind(full,not_full) ~ Irradiation + Temp_mean + 
Duration.ms._10mean + changescalar_mean_10max+ (1|Replicate), family = 
binomial, data = tab)
fm13 <- glmer(cbind(full,not_full) ~ Irradiation + Temp_mean + 
Duration.ms._10max + Changevector_mean_10max + 
changescalar_max_10mean+(1|Replicate), family = binomial, data = tab)
fm14 <- glmer(cbind(full,not_full) ~ Irradiation + Duration.ms._10mean + 
changescalar_mean_10max+ Changevector_max_10mean +(1|Replicate), family = 
binomial, data = tab)
fm15 <- glmer(cbind(full,not_full) ~ Irradiation + 
Angle_mean_15max+Duration.ms._10max + changescalar_mean_10max+ (1|Replicate), 
family = binomial, data = tab)
fm11a <- glmer(cbind(full,not_full) ~ Irradiation + Age + RH._Max + 
Duration.ms._10max + changescalar_mean_10max+(1|Replicate), family = 
binomial, data = tab)
fm12a <- glmer(cbind(full,not_full) ~ Irradiation + RH._Max + 
Duration.ms._10mean + changescalar_mean_10max+ (1|Replicate), family = 
binomial, data = tab)
fm11b <- glmer(cbind(full,not_full) ~ Irradiation +  RH._Max + 
Duration.ms._10max + scalar_mean_10max+(1|Replicate), family = binomial, data 
= tab)
fm11c <- glmer(cbind(full,not_full) ~ Irradiation +  RH._Max + 
Duration.ms._10max + changescalar_mean_10max+(1|Replicate), family = 
binomial, data = tab)
fm12b <- glmer(cbind(full,not_full) ~ Irradiation + Temp_mean + 
Duration.ms._10mean + (1|Replicate), family = binomial, data = tab)


AICc(fm1,fm2,fm3,fm4,fm5,fm6,fm7,fm8,fm1a,fm1b,fm11,fm12,fm13,fm14,fm11a,fm11
b,fm11c,fm12a,fm12b)
```

```
##       df     AICc
## fm1    5 142.0019
## fm2    7 134.4797
## fm3    6 134.0154
## fm4    7 144.9677
## fm5    8 138.7653
## fm6    6 140.9644
## fm7    6 141.4632
## fm8    6 150.0441
```

```
## fm1a   5 144.9050
## fm1b   5 141.2139
## fm11   6 150.4921
## fm12   6 119.5203
## fm13   7 131.8931
## fm14   6 147.0193
## fm11a  7 144.6982
## fm11b  6 155.4982
## fm11c  6 150.4921
## fm12a  6 144.0408
## fm12b  5 121.4973
```

```
summary(fm12)
```

```
## Generalized linear mixed model fit by maximum likelihood (Laplace
##   Approximation) [glmerMod]
##  Family: binomial  ( logit )
## Formula:
## cbind(full, not_full) ~ Irradiation + Temp_mean + Duration.ms._10mean +
##     changescalar_mean_10max + (1 | Replicate)
##    Data: tab
##
##      AIC      BIC   logLik deviance df.resid
##    113.9    120.5    -51.0    101.9       16
##
## Scaled residuals:
##     Min      1Q  Median      3Q     Max
## -2.4407 -1.0049 -0.2980  0.9521  1.9152
##
## Random effects:
##  Groups    Name        Variance Std.Dev.
##  Replicate (Intercept) 0.002217 0.04708
## Number of obs: 22, groups:  Replicate, 6
##
## Fixed effects:
##                         Estimate Std. Error z value Pr(>|z|)
## (Intercept)             -2.59622    0.87307  -2.974  0.00294 **
## Irradiationyes          -0.29225    0.23216  -1.259  0.20810
## Temp_mean                0.22260    0.04022   5.535 3.12e-08 ***
## Duration.ms._10mean     -0.50092    0.09805  -5.109 3.24e-07 ***
## changescalar_mean_10max  0.07505    0.02871   2.614  0.00894 **
## ---
## Signif. codes:  0 '***' 0.001 '**' 0.01 '*' 0.05 '.' 0.1 ' ' 1
##
## Correlation of Fixed Effects:
##             (Intr) Irrdtn Tmp_mn D.._10
## Irradiatnys -0.203
## Temp_mean   -0.713  0.054
## Drtn.ms._10  0.035  0.094 -0.662
## chngscl__10 -0.767 -0.039  0.664 -0.399
```

```
#threshold 15
fm21 <- glmer(cbind(full,not_full) ~ Irradiation + Temp_mean +
Angle_max_15max+changescalar_max_15mean+Duration.ms._15max+(1|Replicate),
family = binomial, data = tab)
fm22 <- glmer(cbind(full,not_full) ~ Irradiation + RH._mean +
changescalar_max_15mean+Duration.ms._15mean+ (1|Replicate), family =
binomial, data = tab)
fm23 <- glmer(cbind(full,not_full) ~ Irradiation +
Angle_max_15max+Duration.ms._15max+changescalar_max_15mean+(1|Replicate),
family = binomial, data = tab)
fm24 <- glmer(cbind(full,not_full) ~ Irradiation + changescalar_max_15mean +
Angle_max_15max+Duration.ms._15mean+ (1|Replicate), family = binomial, data =
tab)
fm25 <- glmer(cbind(full,not_full) ~ Irradiation + changescalar_max_15mean +
Angle_max_15max+ scalar_max_10mean+(1|Replicate), family = binomial, data =
tab)
fm26 <- glmer(cbind(full,not_full) ~ Irradiation +
Angle_max_15max+scalar_max_15mean + Duration.ms._15max +(1|Replicate), family
= binomial, data = tab)
fm27 <- glmer(cbind(full,not_full) ~ Irradiation +
Angle_max_15max+Changevector_max_15mean+scalar_max_10mean+ (1|Replicate),
family = binomial, data = tab)
fm28 <- glmer(cbind(full,not_full) ~ Irradiation + Temp_mean +
Changevector_max_15mean+scalar_max_15mean+(1|Replicate), family = binomial,
data = tab)
fm29 <- glmer(cbind(full,not_full) ~ Irradiation + changescalar_mean_15max +
Changevector_mean_15mean+ Age+ (1|Replicate), family = binomial, data = tab)
fm30 <- glmer(cbind(full,not_full) ~ Irradiation + scalar_max_15mean+
Duration.ms._15mean+ Changevector_max_15mean+(1|Replicate), family =
binomial, data = tab)
fm21a <- glmer(cbind(full,not_full) ~ Irradiation + Temp_mean +
Angle_max_15max+changescalar_max_15mean+ (1|Replicate), family = binomial,
data = tab)
fm27a <- glmer(cbind(full,not_full) ~ Irradiation +Temp_mean +
Duration.ms._15mean+ Changevector_max_15mean+ (1|Replicate), family =
binomial, data = tab)
fm21b <- glmer(cbind(full,not_full) ~ Irradiation + Temp_mean +
Angle_max_15max+changescalar_max_15mean+ (1|Replicate), family = binomial,
data = tab)
AICc(fm1,fm2,fm3,fm4,fm5,fm6,fm7,fm8,fm1a,fm1b,fm11,fm12,fm13,fm14,fm11a,fm11
b,fm11c,fm12a,fm12b,fm21,fm22,fm23,fm24,fm25,fm26,fm27,fm28,fm29,fm30,fm21a,f
m27a,fm21b)

##        df     AICc
## fm1     5 142.0019
## fm2     7 134.4797
## fm3     6 134.0154
## fm4     7 144.9677
## fm5     8 138.7653
## fm6     6 140.9644
```

```
## fm7    6 141.4632
## fm8    6 150.0441
## fm1a   5 144.9050
## fm1b   5 141.2139
## fm11   6 150.4921
## fm12   6 119.5203
## fm13   7 131.8931
## fm14   6 147.0193
## fm11a  7 144.6982
## fm11b  6 155.4982
## fm11c  6 150.4921
## fm12a  6 144.0408
## fm12b  5 121.4973
## fm21   7 134.7744
## fm22   6 120.8532
## fm23   6 152.9068
## fm24   6 139.4548
## fm25   6 155.0636
## fm26   6 150.4280
## fm27   6 155.3545
## fm28   6 136.3177
## fm29   6 127.0716
## fm30   6 145.1004
## fm21a  6 131.2901
## fm27a  6 124.8235
## fm21b  6 131.2901
```

```
summary(fm12)
```

```
## Generalized linear mixed model fit by maximum likelihood (Laplace
##   Approximation) [glmerMod]
##  Family: binomial  ( logit )
## Formula:
## cbind(full, not_full) ~ Irradiation + Temp_mean + Duration.ms._10mean +
##     changescalar_mean_10max + (1 | Replicate)
##    Data: tab
##
##      AIC      BIC   logLik deviance df.resid
##    113.9    120.5    -51.0    101.9       16
##
## Scaled residuals:
##     Min      1Q  Median      3Q     Max
## -2.4407 -1.0049 -0.2980  0.9521  1.9152
##
## Random effects:
##  Groups    Name        Variance Std.Dev.
##  Replicate (Intercept) 0.002217 0.04708
## Number of obs: 22, groups:  Replicate, 6
##
## Fixed effects:
```

```
##                           Estimate Std. Error z value Pr(>|z|)
## (Intercept)               -2.59622    0.87307  -2.974  0.00294 **
## Irradiationyes            -0.29225    0.23216  -1.259  0.20810
## Temp_mean                  0.22260    0.04022   5.535 3.12e-08 ***
## Duration.ms._10mean       -0.50092    0.09805  -5.109 3.24e-07 ***
## changescalar_mean_10max  0.07505    0.02871   2.614  0.00894 **
## ---
## Signif. codes:  0 '***' 0.001 '**' 0.01 '*' 0.05 '.' 0.1 ' ' 1
##
## Correlation of Fixed Effects:
##             (Intr) Irrdtn Tmp_mn D.._10
## Irradiatnys -0.203
## Temp_mean   -0.713  0.054
## Drtn.ms._10  0.035  0.094 -0.662
## chngscl__10 -0.767 -0.039  0.664 -0.399

fm31 <- glmer(cbind(full,not_full) ~ Irradiation + Temp_mean
+Changevector_max_20max+(1|Replicate), family = binomial, data = tab)
fm32 <- glmer(cbind(full,not_full) ~ Irradiation + Temp_mean +
Changevector_max_20mean+ Duration.ms._20max +(1|Replicate), family =
binomial, data = tab)
fm31a <- glmer(cbind(full,not_full) ~ Irradiation + RH._mean +
Angle_max_20max +(1|Replicate), family = binomial, data = tab)
fm31b <- glmer(cbind(full,not_full) ~ Irradiation + Temp_mean
+Duration.ms._20max +(1|Replicate), family = binomial, data = tab)
fm32a <- glmer(cbind(full,not_full) ~ Irradiation + Temp_mean +
Changevector_max_20mean+ Duration.ms._20mean +(1|Replicate), family =
binomial, data = tab)
fm31c <- glmer(cbind(full,not_full) ~ Irradiation + Temp_mean
+Changevector_max_20max+Angle_mean_20max +(1|Replicate), family = binomial,
data = tab)

AICc(fm1,fm2,fm3,fm4,fm5,fm6,fm7,fm8,fm1a,fm1b,fm11,fm12,fm13,fm14,fm11a,fm11
b,fm11c,fm12a,fm12b,fm21,fm22,fm23,fm24,fm25,fm26,fm27,fm28,fm29,fm30,fm21a,f
m27a,fm21b,fm31,fm32,fm31a,fm31b,fm32a,fm31c)

##        df     AICc
## fm1     5 142.0019
## fm2     7 134.4797
## fm3     6 134.0154
## fm4     7 144.9677
## fm5     8 138.7653
## fm6     6 140.9644
## fm7     6 141.4632
## fm8     6 150.0441
## fm1a    5 144.9050
## fm1b    5 141.2139
## fm11    6 150.4921
## fm12    6 119.5203
## fm13    7 131.8931
```

```
## fm14   6 147.0193
## fm11a  7 144.6982
## fm11b  6 155.4982
## fm11c  6 150.4921
## fm12a  6 144.0408
## fm12b  5 121.4973
## fm21   7 134.7744
## fm22   6 120.8532
## fm23   6 152.9068
## fm24   6 139.4548
## fm25   6 155.0636
## fm26   6 150.4280
## fm27   6 155.3545
## fm28   6 136.3177
## fm29   6 127.0716
## fm30   6 145.1004
## fm21a  6 131.2901
## fm27a  6 124.8235
## fm21b  6 131.2901
## fm31   5 136.7918
## fm32   6 133.5936
## fm31a  5 131.1846
## fm31b  5 143.5571
## fm32a  6 128.3010
## fm31c  6 134.8639
```

```
summary(fm12)
```

```
## Generalized linear mixed model fit by maximum likelihood (Laplace
##   Approximation) [glmerMod]
##  Family: binomial  ( logit )
## Formula:
## cbind(full, not_full) ~ Irradiation + Temp_mean + Duration.ms._10mean +
##     changescalar_mean_10max + (1 | Replicate)
##    Data: tab
##
##      AIC      BIC   logLik deviance df.resid
##    113.9    120.5    -51.0    101.9       16
##
## Scaled residuals:
##     Min      1Q  Median      3Q     Max
## -2.4407 -1.0049 -0.2980  0.9521  1.9152
##
## Random effects:
##  Groups    Name        Variance Std.Dev.
##  Replicate (Intercept) 0.002217 0.04708
## Number of obs: 22, groups:  Replicate, 6
##
## Fixed effects:
##                          Estimate Std. Error z value Pr(>|z|)
```

```
## (Intercept)              -2.59622    0.87307  -2.974  0.00294 **
## Irradiationyes           -0.29225    0.23216  -1.259  0.20810
## Temp_mean                 0.22260    0.04022   5.535 3.12e-08 ***
## Duration.ms._10mean      -0.50092    0.09805  -5.109 3.24e-07 ***
## changescalar_mean_10max  0.07505    0.02871   2.614  0.00894 **
## ---
## Signif. codes:  0 '***' 0.001 '**' 0.01 '*' 0.05 '.' 0.1 ' ' 1
##
## Correlation of Fixed Effects:
##             (Intr) Irrdtn Tmp_mn D.._10
## Irradiatnys -0.203
## Temp_mean   -0.713  0.054
## Drtn.ms._10  0.035  0.094 -0.662
## chngscl__10 -0.767 -0.039  0.664 -0.399
```

```
#combination of thresholds

fm41 <- glmer(cbind(full,not_full) ~ Irradiation + RH._Max +
changescalar_mean_10max+ Duration.ms._5mean +Angle_max_20max +(1|Replicate),
family = binomial, data = tab)
fm42 <- glmer(cbind(full,not_full) ~ Irradiation + Temp_mean +
Duration.ms._10mean + Angle_max_20max ++ (1|Replicate), family = binomial,
data = tab)
fm43 <- glmer(cbind(full,not_full) ~ Irradiation + RH._mean +
changescalar_max_15mean+Duration.ms._5mean+ Angle_max_20max +(1|Replicate),
family = binomial, data = tab)
fm44 <- glmer(cbind(full,not_full) ~ Irradiation + RH._mean + Angle_max_20max
+(1|Replicate), family = binomial, data = tab)
fm45 <- glmer(cbind(full,not_full) ~ Irradiation + RH._Max + Age +
changescalar_mean_10max+(1|Replicate), family = binomial, data = tab)
fm46 <- glmer(cbind(full,not_full) ~ Irradiation + RH._Max + Age +
changescalar_mean_10max+ Duration.ms._5mean +(1|Replicate), family =
binomial, data = tab)
fm47 <- glmer(cbind(full,not_full) ~ Irradiation + RH._mean +
changescalar_max_15mean+Duration.ms._5mean+ (1|Replicate), family = binomial,
data = tab)
AICc(fm1,fm2,fm3,fm4,fm5,fm6,fm7,fm8,fm1a,fm1b,fm11,fm12,fm13,fm14,fm11a,fm11
b,fm11c,fm12a,fm12b,fm21,fm22,fm23,fm24,fm25,fm26,fm27,fm28,fm29,fm30,fm21a,f
m27a,fm21b,fm31,fm32,fm31a,fm31b,fm32a,fm31c,fm41,fm42,fm43,fm44,fm45,fm46,fm
47)
```

```
##        df     AICc
## fm1     5 142.0019
## fm2     7 134.4797
## fm3     6 134.0154
## fm4     7 144.9677
## fm5     8 138.7653
## fm6     6 140.9644
## fm7     6 141.4632
## fm8     6 150.0441
```

```
## fm1a   5 144.9050
## fm1b   5 141.2139
## fm11   6 150.4921
## fm12   6 119.5203
## fm13   7 131.8931
## fm14   6 147.0193
## fm11a  7 144.6982
## fm11b  6 155.4982
## fm11c  6 150.4921
## fm12a  6 144.0408
## fm12b  5 121.4973
## fm21   7 134.7744
## fm22   6 120.8532
## fm23   6 152.9068
## fm24   6 139.4548
## fm25   6 155.0636
## fm26   6 150.4280
## fm27   6 155.3545
## fm28   6 136.3177
## fm29   6 127.0716
## fm30   6 145.1004
## fm21a  6 131.2901
## fm27a  6 124.8235
## fm21b  6 131.2901
## fm31   5 136.7918
## fm32   6 133.5936
## fm31a  5 131.1846
## fm31b  5 143.5571
## fm32a  6 128.3010
## fm31c  6 134.8639
## fm41   7 149.2284
## fm42   6 123.7183
## fm43   7 125.3293
## fm44   5 131.1846
## fm45   6 143.9741
## fm46   7 137.6587
## fm47   6 121.1931
```

```
summary(fm12)
```

```
## Generalized linear mixed model fit by maximum likelihood (Laplace
##   Approximation) [glmerMod]
##  Family: binomial  ( logit )
## Formula:
## cbind(full, not_full) ~ Irradiation + Temp_mean + Duration.ms._10mean +
##     changescalar_mean_10max + (1 | Replicate)
##    Data: tab
##
##      AIC      BIC   logLik deviance df.resid
##    113.9    120.5    -51.0    101.9       16
```

```
##
## Scaled residuals:
##     Min      1Q  Median      3Q     Max
## -2.4407 -1.0049 -0.2980  0.9521  1.9152
##
## Random effects:
##  Groups    Name        Variance Std.Dev.
##  Replicate (Intercept) 0.002217 0.04708
## Number of obs: 22, groups:  Replicate, 6
##
## Fixed effects:
##                          Estimate Std. Error z value Pr(>|z|)
## (Intercept)              -2.59622    0.87307  -2.974  0.00294 **
## Irradiationyes           -0.29225    0.23216  -1.259  0.20810
## Temp_mean                 0.22260    0.04022   5.535 3.12e-08 ***
## Duration.ms._10mean      -0.50092    0.09805  -5.109 3.24e-07 ***
## changescalar_mean_10max  0.07505    0.02871   2.614  0.00894 **
## ---
## Signif. codes:  0 '***' 0.001 '**' 0.01 '*' 0.05 '.' 0.1 ' ' 1
##
## Correlation of Fixed Effects:
##             (Intr) Irrdtn Tmp_mn D.._10
## Irradiatnys -0.203
## Temp_mean   -0.713  0.054
## Drtn.ms._10  0.035  0.094 -0.662
## chngscl__10 -0.767 -0.039  0.664 -0.399

#the best model remains as fm12


##Figure 9d

head(tab)

##     Date Replicate Age     Treatments not_full full  MSV MSV2 shipped
## 1 170510        R1  22 Shipped-110Gy        2    6 0.75 1.50     yes
## 2 170510        R1  22   Shipped-0Gy        4    6 0.70 1.40     yes
## 3 170517        R2  22 Shipped-110Gy       22    5 0.41 0.82     yes
## 4 170517        R2  22   Shipped-0Gy       13   17 0.66 1.32     yes
## 5 170524        R3  22 Shipped-110Gy        6    7 0.60 1.20     yes
## 6 170524        R3  22   Shipped-0Gy       22    4 0.42 0.84     yes
##   Irradiation Events._5max Duration.ms._5max scalar_max_5max
scalar_mean_5max
## 1         yes          909            48.750          27.374
21.43900
## 2          no          909            48.750          27.374
21.43900
## 3         yes          708            87.500          27.398
16.92723
## 4          no          708            87.500          27.398
```

```
16.92723
## 5         yes         1187            93.125          27.374
15.43705
## 6          no         1187            93.125          27.374
15.43705
##   changescalar_max_5max changescalar_mean_5max Changevector_max_5max
## 1                23.724                8.94875              27.69156
## 2                23.724                8.94875              27.69156
## 3                24.477               11.37550              35.06987
## 4                24.477               11.37550              35.06987
## 5                16.627                3.83800              33.00443
## 6                16.627                3.83800              33.00443
##   Changevector_mean_5max Angle_max_5max Angle_mean_5max Hz_max_5max
## 1               9.937386       3.002116        2.617132    764.4826
## 2               9.937386       3.002116        2.617132    764.4826
## 3              19.035967       2.969360        1.679485    756.1414
## 4              19.035967       2.969360        1.679485    756.1414
## 5               5.064434       2.105922        1.356174    536.2686
## 6               5.064434       2.105922        1.356174    536.2686
##   Hz_mean_5max Events_10max Duration.ms._10max scalar_max_10max
## 1     666.4471          288             36.875           27.374
## 2     666.4471          288             36.875           27.374
## 3     427.6775          315             50.000           27.398
## 4     427.6775          315             50.000           27.398
## 5     345.3468          238             63.750           27.374
## 6     345.3468          238             63.750           27.374
##   scalar_mean_10max changescalar_max_10max changescalar_mean_10max
## 1           21.5800                 23.724                  12.473
## 2           21.5800                 23.724                  12.473
## 3           20.3750                 24.477                  16.488
## 4           20.3750                 24.477                  16.488
## 5           19.6895                 16.627                   8.965
## 6           19.6895                 16.627                   8.965
##   Changevector_max_10max Changevector_mean_10max Angle_max_10max
## 1               27.69156                15.45774        2.687392
## 2               27.69156                15.45774        2.687392
## 3               35.06987                22.58486        2.969360
## 4               35.06987                22.58486        2.969360
## 5               33.00443                10.65686        2.105922
## 6               33.00443                10.65686        2.105922
##   Angle_mean_10max Hz_max_10max Hz_mean_10max Events_15max
Duration.ms._15max
## 1        1.0470779     684.3388      266.6362          144
30.625
## 2        1.0470779     684.3388      266.6362          144
30.625
## 3        1.6138687     756.1414      410.9683          183
29.375
## 4        1.6138687     756.1414      410.9683          183
29.375
```

```
## 5        0.5851573     536.2686      149.0091          111
41.250
## 6        0.5851573     536.2686      149.0091          111
41.250
##   scalar_max_15max scalar_mean_15max changescalar_max_15max
## 1           27.374          22.42986                 23.724
## 2           27.374          22.42986                 23.724
## 3           27.398          21.71558                 24.477
## 4           27.398          21.71558                 24.477
## 5           27.374          22.77170                 16.627
## 6           27.374          22.77170                 16.627
##   changescalar_mean_15max Changevector_max_15max Changevector_mean_15max
## 1                  15.667               27.69156                19.75723
## 2                  15.667               27.69156                19.75723
## 3                  16.488               35.06987                21.61531
## 4                  16.488               35.06987                21.61531
## 5                  11.474               33.00443                12.83840
## 6                  11.474               33.00443                12.83840
##   Angle_max_15max Angle_mean_15max Hz_max_15max Hz_mean_15max Events_20max
## 1        2.687392        1.8427590     684.3388      469.2547           57
## 2        2.687392        1.8427590     684.3388      469.2547           57
## 3        2.969360        1.6010884     756.1414      407.7138           75
## 4        2.969360        1.6010884     756.1414      407.7138           75
## 5        2.105922        0.6709018     536.2686      170.8438           42
## 6        2.105922        0.6709018     536.2686      170.8438           42
##   Duration.ms._20max scalar_max_20max scalar_mean_20max
changescalar_max_20max
## 1             17.500           27.374          26.80175
23.724
## 2             17.500           27.374          26.80175
23.724
## 3             20.625           27.398          26.38567
24.477
## 4             20.625           27.398          26.38567
24.477
## 5             20.625           27.374          25.17900
13.480
## 6             20.625           27.374          25.17900
13.480
##   changescalar_mean_20max Changevector_max_20max Changevector_mean_20max
## 1                 23.7240               27.69156                27.69156
## 2                 23.7240               27.69156                27.69156
## 3                 13.7095               35.06987                29.65156
## 4                 13.7095               35.06987                29.65156
## 5                  6.6090               33.00443                11.19072
## 6                  6.6090               33.00443                11.19072
##   Angle_max_20max Angle_mean_20max Hz_max_20max Hz_mean_20max Events_5mean
## 1        2.687392         2.687392     684.3388      684.3388          909
## 2        2.687392         2.687392     684.3388      684.3388          909
## 3        2.433775         1.792941     619.7557      456.5688          708
```

```
## 4        2.433775         1.792941     619.7557      456.5688          708
## 5        1.715913         0.489164     436.9538      124.5646         1187
## 6        1.715913         0.489164     436.9538      124.5646         1187
##   Duration.ms._5mean scalar_max_5mean scalar_mean_5mean
changescalar_max_5mean
## 1           5.215209         9.516648          7.473747
3.755884
## 2           5.215209         9.516648          7.473747
3.755884
## 3          11.785840        10.551797          7.565272
2.816095
## 4          11.785840        10.551797          7.565272
2.816095
## 5           7.348884         8.022198          6.506753
1.632164
## 6           7.348884         8.022198          6.506753
1.632164
##   changescalar_mean_5mean Changevector_max_5mean Changevector_mean_5mean
## 1               1.6630629               4.501180                2.219564
## 2               1.6630629               4.501180                2.219564
## 3               1.0076953               3.691769                1.638802
## 4               1.0076953               3.691769                1.638802
## 5               0.6804169               2.293485                1.194539
## 6               0.6804169               2.293485                1.194539
##   Angle_max_5mean Angle_mean_5mean Hz_max_5mean Hz_mean_5mean
Events._10mean
## 1       0.4296757        0.1755708    109.41603      44.70874
288
## 2       0.4296757        0.1755708    109.41603      44.70874
288
## 3       0.3715939        0.1414327     94.62562      36.01553
315
## 4       0.3715939        0.1414327     94.62562      36.01553
315
## 5       0.2539263        0.1310499     64.66179      33.37158
238
## 6       0.2539263        0.1310499     64.66179      33.37158
238
##   Duration.ms._10mean scalar_max_10mean scalar_mean_10mean
## 1            3.888889          15.49537           13.49979
## 2            3.888889          15.49537           13.49979
## 3            7.706349          16.38244           13.65682
## 4            7.706349          16.38244           13.65682
## 5            7.415966          15.56483           13.15894
## 6            7.415966          15.56483           13.15894
##   changescalar_max_10mean changescalar_mean_10mean Changevector_max_10mean
## 1                6.557340                 3.249119                8.029862
## 2                6.557340                 3.249119                8.029862
## 3                4.928854                 1.971893                6.505797
## 4                4.928854                 1.971893                6.505797
```

```
## 5                 4.004025                  1.664277                  5.891075
## 6                 4.004025                  1.664277                  5.891075
##   Changevector_mean_10mean Angle_max_10mean Angle_mean_10mean
Hz_max_10mean
## 1                 4.538349        0.4719538         0.2224823
120.1821
## 2                 4.538349        0.4719538         0.2224823
120.1821
## 3                 3.339135        0.4047630         0.1751144
103.0721
## 4                 3.339135        0.4047630         0.1751144
103.0721
## 5                 2.909783        0.3492638         0.1583697
88.9393
## 6                 2.909783        0.3492638         0.1583697
88.9393
##   Hz_mean_10mean Events_15mean Duration.ms._15mean scalar_max_15mean
## 1       56.65466           144            3.441840          19.26462
## 2       56.65466           144            3.441840          19.26462
## 3       44.59251           183            6.342213          19.93954
## 4       44.59251           183            6.342213          19.93954
## 5       40.32851           111            6.920045          19.71768
## 6       40.32851           111            6.920045          19.71768
##   scalar_mean_15mean changescalar_max_15mean changescalar_mean_15mean
## 1           17.94158                8.195410                 4.209463
## 2           17.94158                8.195410                 4.209463
## 3           17.79343                6.271208                 2.424063
## 4           17.79343                6.271208                 2.424063
## 5           17.60073                5.291784                 2.055466
## 6           17.60073                5.291784                 2.055466
##   Changevector_max_15mean Changevector_mean_15mean Angle_max_15mean
## 1               10.145769                 6.194357        0.4732660
## 2               10.145769                 6.194357        0.4732660
## 3                8.773143                 4.485467        0.4584332
## 4                8.773143                 4.485467        0.4584332
## 5                8.644509                 4.178605        0.4437400
## 6                8.644509                 4.178605        0.4437400
##   Angle_mean_15mean Hz_max_15mean Hz_mean_15mean Events_20mean
## 1         0.2644405      120.5162       67.33922            57
## 2         0.2644405      120.5162       67.33922            57
## 3         0.2042285      116.7390       52.00635            75
## 4         0.2042285      116.7390       52.00635            75
## 5         0.1960600      112.9975       49.92626            42
## 6         0.1960600      112.9975       49.92626            42
##   Duration.ms._20mean scalar_max_20mean scalar_mean_20mean
## 1            2.587719          22.76440           22.04857
## 2            2.587719          22.76440           22.04857
## 3            5.250000          23.71752           22.29175
## 4            5.250000          23.71752           22.29175
## 5            5.997024          24.28693           22.58697
```

```
## 6            5.997024          24.28693           22.58697
##   changescalar_max_20mean changescalar_mean_20mean Changevector_max_20mean
## 1                7.502070                 3.960703                11.35123
## 2                7.502070                 3.960703                11.35123
## 3                6.190293                 2.676377                11.36833
## 4                6.190293                 2.676377                11.36833
## 5                5.996190                 2.445063                11.97291
## 6                5.996190                 2.445063                11.97291
##   Changevector_mean_20mean Angle_max_20mean Angle_mean_20mean
Hz_max_20mean
## 1                 6.922532        0.5201012         0.3140733
132.4427
## 2                 6.922532        0.5201012         0.3140733
132.4427
## 3                 5.877764        0.5214004         0.2548023
132.7735
## 4                 5.877764        0.5214004         0.2548023
132.7735
## 5                 5.476046        0.5391966         0.2201107
137.3053
## 6                 5.476046        0.5391966         0.2201107
137.3053
##   Hz_mean_20mean RH._Max Temp_Max RH._mean Temp_mean pcinsemination
## 1       79.97812   47.08    23.50    41.69     19.73      0.7500000
## 2       79.97812   60.14    27.50    41.69     19.73      0.6000000
## 3       64.88487   62.61    29.75    53.55     21.79      0.1851852
## 4       64.88487   68.10    32.00    53.55     21.79      0.5666667
## 5       56.05073   57.37    28.69    53.25     23.29      0.5384615
## 6       56.05073   74.59    19.25    53.25     23.29      0.1538462
```

```
str(tab)
```

```
## 'data.frame':    22 obs. of  111 variables:
##  $ Date                     : int  170510 170510 170517 170517 170524
170524 170531 170531 170607 170607 ...
##  $ Replicate                : chr  "R1" "R1" "R2" "R2" ...
##  $ Age                      : Factor w/ 2 levels "22","29": 1 1 1 1 1 1 1 1
1 1 ...
##  $ Treatments               : chr  "Shipped-110Gy " "Shipped-0Gy "
"Shipped-110Gy " "Shipped-0Gy " ...
##  $ not_full                 : int  2 4 22 13 6 22 4 14 11 17 ...
##  $ full                     : int  6 6 5 17 7 4 0 9 8 22 ...
##  $ MSV                      : num  0.75 0.7 0.41 0.66 0.6 0.42 0.25 0.57
0.47 0.66 ...
##  $ MSV2                     : num  1.5 1.4 0.82 1.32 1.2 0.84 0.5 1.14 0.94
1.32 ...
##  $ shipped                  : chr  "yes" "yes" "yes" "yes" ...
##  $ Irradiation              : chr  "yes" "no" "yes" "no" ...
##  $ Events._5max             : int  909 909 708 708 1187 1187 625 625 606
606 ...
```

```
##  $ Duration.ms._5max       : num  48.8 48.8 87.5 87.5 93.1 ...
##  $ scalar_max_5max         : num  27.4 27.4 27.4 27.4 27.4 ...
##  $ scalar_mean_5max        : num  21.4 21.4 16.9 16.9 15.4 ...
##  $ changescalar_max_5max   : num  23.7 23.7 24.5 24.5 16.6 ...
##  $ changescalar_mean_5max  : num  8.95 8.95 11.38 11.38 3.84 ...
##  $ Changevector_max_5max   : num  27.7 27.7 35.1 35.1 33 ...
##  $ Changevector_mean_5max  : num  9.94 9.94 19.04 19.04 5.06 ...
##  $ Angle_max_5max          : num  3 3 2.97 2.97 2.11 ...
##  $ Angle_mean_5max         : num  2.62 2.62 1.68 1.68 1.36 ...
##  $ Hz_max_5max             : num  764 764 756 756 536 ...
##  $ Hz_mean_5max            : num  666 666 428 428 345 ...
##  $ Events_10max            : int  288 288 315 315 238 238 261 261 219 219
...
##  $ Duration.ms._10max      : num  36.9 36.9 50 50 63.8 ...
##  $ scalar_max_10max        : num  27.4 27.4 27.4 27.4 27.4 ...
##  $ scalar_mean_10max       : num  21.6 21.6 20.4 20.4 19.7 ...
##  $ changescalar_max_10max  : num  23.7 23.7 24.5 24.5 16.6 ...
##  $ changescalar_mean_10max : num  12.47 12.47 16.49 16.49 8.97 ...
##  $ Changevector_max_10max  : num  27.7 27.7 35.1 35.1 33 ...
##  $ Changevector_mean_10max : num  15.5 15.5 22.6 22.6 10.7 ...
##  $ Angle_max_10max         : num  2.69 2.69 2.97 2.97 2.11 ...
##  $ Angle_mean_10max        : num  1.047 1.047 1.614 1.614 0.585 ...
##  $ Hz_max_10max            : num  684 684 756 756 536 ...
##  $ Hz_mean_10max           : num  267 267 411 411 149 ...
##  $ Events_15max            : int  144 144 183 183 111 111 163 163 129 129
...
##  $ Duration.ms._15max      : num  30.6 30.6 29.4 29.4 41.2 ...
##  $ scalar_max_15max        : num  27.4 27.4 27.4 27.4 27.4 ...
##  $ scalar_mean_15max       : num  22.4 22.4 21.7 21.7 22.8 ...
##  $ changescalar_max_15max  : num  23.7 23.7 24.5 24.5 16.6 ...
##  $ changescalar_mean_15max : num  15.7 15.7 16.5 16.5 11.5 ...
##  $ Changevector_max_15max  : num  27.7 27.7 35.1 35.1 33 ...
##  $ Changevector_mean_15max : num  19.8 19.8 21.6 21.6 12.8 ...
##  $ Angle_max_15max         : num  2.69 2.69 2.97 2.97 2.11 ...
##  $ Angle_mean_15max        : num  1.843 1.843 1.601 1.601 0.671 ...
##  $ Hz_max_15max            : num  684 684 756 756 536 ...
##  $ Hz_mean_15max           : num  469 469 408 408 171 ...
##  $ Events_20max            : int  57 57 75 75 42 42 97 97 71 71 ...
##  $ Duration.ms._20max      : num  17.5 17.5 20.6 20.6 20.6 ...
##  $ scalar_max_20max        : num  27.4 27.4 27.4 27.4 27.4 ...
##  $ scalar_mean_20max       : num  26.8 26.8 26.4 26.4 25.2 ...
##  $ changescalar_max_20max  : num  23.7 23.7 24.5 24.5 13.5 ...
##  $ changescalar_mean_20max : num  23.72 23.72 13.71 13.71 6.61 ...
##  $ Changevector_max_20max  : num  27.7 27.7 35.1 35.1 33 ...
##  $ Changevector_mean_20max : num  27.7 27.7 29.7 29.7 11.2 ...
##  $ Angle_max_20max         : num  2.69 2.69 2.43 2.43 1.72 ...
##  $ Angle_mean_20max        : num  2.687 2.687 1.793 1.793 0.489 ...
##  $ Hz_max_20max            : num  684 684 620 620 437 ...
##  $ Hz_mean_20max           : num  684 684 457 457 125 ...
##  $ Events_5mean            : int  909 909 708 708 1187 1187 625 625 606
```

```
606 ...
##  $ Duration.ms._5mean      : num  5.22 5.22 11.79 11.79 7.35 ...
##  $ scalar_max_5mean        : num  9.52 9.52 10.55 10.55 8.02 ...
##  $ scalar_mean_5mean       : num  7.47 7.47 7.57 7.57 6.51 ...
##  $ changescalar_max_5mean  : num  3.76 3.76 2.82 2.82 1.63 ...
##  $ changescalar_mean_5mean : num  1.66 1.66 1.01 1.01 0.68 ...
##  $ Changevector_max_5mean  : num  4.5 4.5 3.69 3.69 2.29 ...
##  $ Changevector_mean_5mean : num  2.22 2.22 1.64 1.64 1.19 ...
##  $ Angle_max_5mean         : num  0.43 0.43 0.372 0.372 0.254 ...
##  $ Angle_mean_5mean        : num  0.176 0.176 0.141 0.141 0.131 ...
##  $ Hz_max_5mean            : num  109.4 109.4 94.6 94.6 64.7 ...
##  $ Hz_mean_5mean           : num  44.7 44.7 36 36 33.4 ...
##  $ Events._10mean          : int  288 288 315 315 238 238 261 261 219 219
...
##  $ Duration.ms._10mean     : num  3.89 3.89 7.71 7.71 7.42 ...
##  $ scalar_max_10mean       : num  15.5 15.5 16.4 16.4 15.6 ...
##  $ scalar_mean_10mean      : num  13.5 13.5 13.7 13.7 13.2 ...
##  $ changescalar_max_10mean : num  6.56 6.56 4.93 4.93 4 ...
##  $ changescalar_mean_10mean: num  3.25 3.25 1.97 1.97 1.66 ...
##  $ Changevector_max_10mean : num  8.03 8.03 6.51 6.51 5.89 ...
##  $ Changevector_mean_10mean: num  4.54 4.54 3.34 3.34 2.91 ...
##  $ Angle_max_10mean        : num  0.472 0.472 0.405 0.405 0.349 ...
##  $ Angle_mean_10mean       : num  0.222 0.222 0.175 0.175 0.158 ...
##  $ Hz_max_10mean           : num  120.2 120.2 103.1 103.1 88.9 ...
##  $ Hz_mean_10mean          : num  56.7 56.7 44.6 44.6 40.3 ...
##  $ Events_15mean           : int  144 144 183 183 111 111 163 163 129 129
...
##  $ Duration.ms._15mean     : num  3.44 3.44 6.34 6.34 6.92 ...
##  $ scalar_max_15mean       : num  19.3 19.3 19.9 19.9 19.7 ...
##  $ scalar_mean_15mean      : num  17.9 17.9 17.8 17.8 17.6 ...
##  $ changescalar_max_15mean : num  8.2 8.2 6.27 6.27 5.29 ...
##  $ changescalar_mean_15mean: num  4.21 4.21 2.42 2.42 2.06 ...
##  $ Changevector_max_15mean : num  10.15 10.15 8.77 8.77 8.64 ...
##  $ Changevector_mean_15mean: num  6.19 6.19 4.49 4.49 4.18 ...
##  $ Angle_max_15mean        : num  0.473 0.473 0.458 0.458 0.444 ...
##  $ Angle_mean_15mean       : num  0.264 0.264 0.204 0.204 0.196 ...
##  $ Hz_max_15mean           : num  121 121 117 117 113 ...
##  $ Hz_mean_15mean          : num  67.3 67.3 52 52 49.9 ...
##  $ Events_20mean           : int  57 57 75 75 42 42 97 97 71 71 ...
##  $ Duration.ms._20mean     : num  2.59 2.59 5.25 5.25 6 ...
##  $ scalar_max_20mean       : num  22.8 22.8 23.7 23.7 24.3 ...
##  $ scalar_mean_20mean      : num  22 22 22.3 22.3 22.6 ...
##  $ changescalar_max_20mean : num  7.5 7.5 6.19 6.19 6 ...
##    [list output truncated]

summary(fm12)

## Generalized linear mixed model fit by maximum likelihood (Laplace
##   Approximation) [glmerMod]
##  Family: binomial  ( logit )
```

```
## Formula:
## cbind(full, not_full) ~ Irradiation + Temp_mean + Duration.ms._10mean +
##     changescalar_mean_10max + (1 | Replicate)
##    Data: tab
##
##      AIC      BIC   logLik deviance df.resid
##    113.9    120.5    -51.0    101.9       16
##
## Scaled residuals:
##     Min      1Q  Median      3Q     Max
## -2.4407 -1.0049 -0.2980  0.9521  1.9152
##
## Random effects:
##  Groups    Name        Variance Std.Dev.
##  Replicate (Intercept) 0.002217 0.04708
## Number of obs: 22, groups:  Replicate, 6
##
## Fixed effects:
##                         Estimate Std. Error z value Pr(>|z|)
## (Intercept)             -2.59622    0.87307  -2.974  0.00294 **
## Irradiationyes          -0.29225    0.23216  -1.259  0.20810
## Temp_mean                0.22260    0.04022   5.535 3.12e-08 ***
## Duration.ms._10mean     -0.50092    0.09805  -5.109 3.24e-07 ***
## changescalar_mean_10max  0.07505    0.02871   2.614  0.00894 **
## ---
## Signif. codes:  0 '***' 0.001 '**' 0.01 '*' 0.05 '.' 0.1 ' ' 1
##
## Correlation of Fixed Effects:
##             (Intr) Irrdtn Tmp_mn D.._10
## Irradiatnys -0.203
## Temp_mean   -0.713  0.054
## Drtn.ms._10  0.035  0.094 -0.662
## chngscl__10 -0.767 -0.039  0.664 -0.399
```

```
plot((full / not_full) ~ fitted(fm12), data = tab)
abline(lm((full / not_full) ~ fitted(fm12), data = tab), col = "red")
```

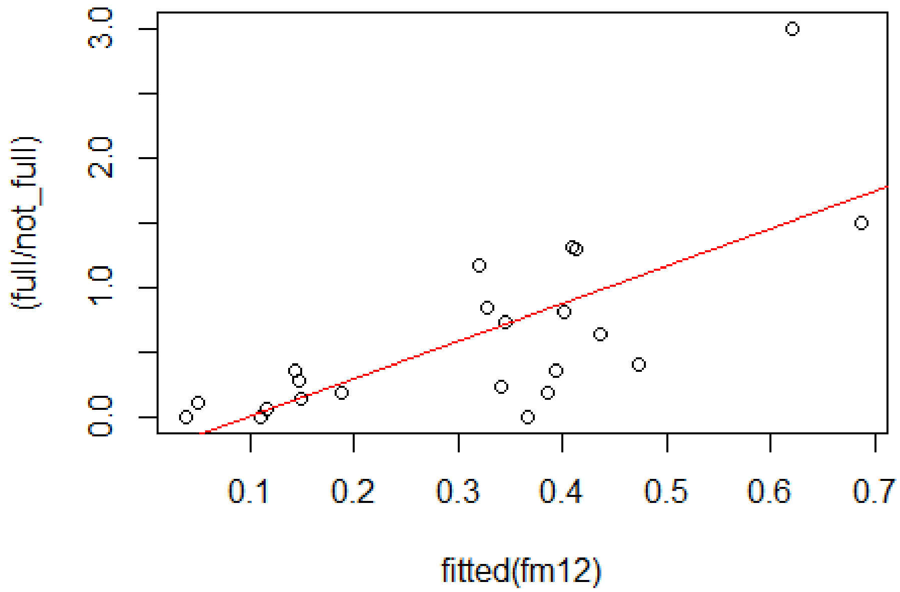


```
cor.test(tab$full/tab$not_full,fitted(fm12))

##
##  Pearson's product-moment correlation
##
## data:  tab$full/tab$not_full and fitted(fm12)
## t = 4.597, df = 20, p-value = 0.0001746
## alternative hypothesis: true correlation is not equal to 0
## 95 percent confidence interval:
##  0.4229951 0.8742013
## sample estimates:
##       cor
## 0.7167751

summary(lm(fitted(fm12)~(tab$full/tab$not_full)))$r.squared

## [1] 0.4327613

##### Independent variables not correlated
```

THE IMPACT OF SHOCK ON THE MEAN SPERMATHECAL VALUE(MSV) OF 22 AND 29 DAY OLD PUPAE(COMBINED DATA) Figure 9e

```
tab <- read.csv("Figure 9e.csv")
head(tab)

##     Date Replicate Age     Treatments not_full full  MSV MSV2 shipped
## 1 170510        R1  22 Shipped-110Gy        2    6 0.75 1.50     yes
```

```
## 2 170510        R1  22   Shipped-0Gy         4    6 0.70 1.40     yes
## 3 170517        R2  22 Shipped-110Gy       22    5 0.41 0.82     yes
## 4 170517        R2  22   Shipped-0Gy       13   17 0.66 1.32     yes
## 5 170524        R3  22 Shipped-110Gy        6    7 0.60 1.20     yes
## 6 170524        R3  22   Shipped-0Gy       22    4 0.42 0.84     yes
##   Irradiation Events._5max Duration.ms._5max scalar_max_5max
scalar_mean_5max
## 1         yes          909            48.750          27.374
21.43900
## 2          no          909            48.750          27.374
21.43900
## 3         yes          708            87.500          27.398
16.92723
## 4          no          708            87.500          27.398
16.92723
## 5         yes         1187            93.125          27.374
15.43705
## 6          no         1187            93.125          27.374
15.43705
##   changescalar_max_5max changescalar_mean_5max Changevector_max_5max
## 1                23.724                8.94875              27.69156
## 2                23.724                8.94875              27.69156
## 3                24.477               11.37550              35.06987
## 4                24.477               11.37550              35.06987
## 5                16.627                3.83800              33.00443
## 6                16.627                3.83800              33.00443
##   Changevector_mean_5max Angle_max_5max Angle_mean_5max Hz_max_5max
## 1               9.937386       3.002116        2.617132    764.4826
## 2               9.937386       3.002116        2.617132    764.4826
## 3              19.035967       2.969360        1.679485    756.1414
## 4              19.035967       2.969360        1.679485    756.1414
## 5               5.064434       2.105922        1.356174    536.2686
## 6               5.064434       2.105922        1.356174    536.2686
##   Hz_mean_5max Events_10max Duration.ms._10max scalar_max_10max
## 1     666.4471          288             36.875           27.374
## 2     666.4471          288             36.875           27.374
## 3     427.6775          315             50.000           27.398
## 4     427.6775          315             50.000           27.398
## 5     345.3468          238             63.750           27.374
## 6     345.3468          238             63.750           27.374
##   scalar_mean_10max changescalar_max_10max changescalar_mean_10max
## 1           21.5800                 23.724                  12.473
## 2           21.5800                 23.724                  12.473
## 3           20.3750                 24.477                  16.488
## 4           20.3750                 24.477                  16.488
## 5           19.6895                 16.627                   8.965
## 6           19.6895                 16.627                   8.965
##   Changevector_max_10max Changevector_mean_10max Angle_max_10max
## 1               27.69156                15.45774        2.687392
## 2               27.69156                15.45774        2.687392
```

```
## 3               35.06987                22.58486        2.969360
## 4               35.06987                22.58486        2.969360
## 5               33.00443                10.65686        2.105922
## 6               33.00443                10.65686        2.105922
##   Angle_mean_10max Hz_max_10max Hz_mean_10max Events_15max
Duration.ms._15max
## 1        1.0470779     684.3388      266.6362          144
30.625
## 2        1.0470779     684.3388      266.6362          144
30.625
## 3        1.6138687     756.1414      410.9683          183
29.375
## 4        1.6138687     756.1414      410.9683          183
29.375
## 5        0.5851573     536.2686      149.0091          111
41.250
## 6        0.5851573     536.2686      149.0091          111
41.250
##   scalar_max_15max scalar_mean_15max changescalar_max_15max
## 1           27.374          22.42986                 23.724
## 2           27.374          22.42986                 23.724
## 3           27.398          21.71558                 24.477
## 4           27.398          21.71558                 24.477
## 5           27.374          22.77170                 16.627
## 6           27.374          22.77170                 16.627
##   changescalar_mean_15max Changevector_max_15max Changevector_mean_15max
## 1                  15.667               27.69156                19.75723
## 2                  15.667               27.69156                19.75723
## 3                  16.488               35.06987                21.61531
## 4                  16.488               35.06987                21.61531
## 5                  11.474               33.00443                12.83840
## 6                  11.474               33.00443                12.83840
##   Angle_max_15max Angle_mean_15max Hz_max_15max Hz_mean_15max Events_20max
## 1        2.687392        1.8427590     684.3388      469.2547           57
## 2        2.687392        1.8427590     684.3388      469.2547           57
## 3        2.969360        1.6010884     756.1414      407.7138           75
## 4        2.969360        1.6010884     756.1414      407.7138           75
## 5        2.105922        0.6709018     536.2686      170.8438           42
## 6        2.105922        0.6709018     536.2686      170.8438           42
##   Duration.ms._20max scalar_max_20max scalar_mean_20max
changescalar_max_20max
## 1             17.500           27.374          26.80175
23.724
## 2             17.500           27.374          26.80175
23.724
## 3             20.625           27.398          26.38567
24.477
## 4             20.625           27.398          26.38567
24.477
## 5             20.625           27.374          25.17900
```

```
13.480
## 6             20.625           27.374          25.17900
13.480
##   changescalar_mean_20max Changevector_max_20max Changevector_mean_20max
## 1                 23.7240               27.69156                27.69156
## 2                 23.7240               27.69156                27.69156
## 3                 13.7095               35.06987                29.65156
## 4                 13.7095               35.06987                29.65156
## 5                  6.6090               33.00443                11.19072
## 6                  6.6090               33.00443                11.19072
##   Angle_max_20max Angle_mean_20max Hz_max_20max Hz_mean_20max Events_5mean
## 1        2.687392         2.687392     684.3388      684.3388          909
## 2        2.687392         2.687392     684.3388      684.3388          909
## 3        2.433775         1.792941     619.7557      456.5688          708
## 4        2.433775         1.792941     619.7557      456.5688          708
## 5        1.715913         0.489164     436.9538      124.5646         1187
## 6        1.715913         0.489164     436.9538      124.5646         1187
##   Duration.ms._5mean scalar_max_5mean scalar_mean_5mean
changescalar_max_5mean
## 1           5.215209         9.516648          7.473747
3.755884
## 2           5.215209         9.516648          7.473747
3.755884
## 3          11.785840        10.551797          7.565272
2.816095
## 4          11.785840        10.551797          7.565272
2.816095
## 5           7.348884         8.022198          6.506753
1.632164
## 6           7.348884         8.022198          6.506753
1.632164
##   changescalar_mean_5mean Changevector_max_5mean Changevector_mean_5mean
## 1               1.6630629               4.501180                2.219564
## 2               1.6630629               4.501180                2.219564
## 3               1.0076953               3.691769                1.638802
## 4               1.0076953               3.691769                1.638802
## 5               0.6804169               2.293485                1.194539
## 6               0.6804169               2.293485                1.194539
##   Angle_max_5mean Angle_mean_5mean Hz_max_5mean Hz_mean_5mean
Events._10mean
## 1       0.4296757        0.1755708    109.41603      44.70874
288
## 2       0.4296757        0.1755708    109.41603      44.70874
288
## 3       0.3715939        0.1414327     94.62562      36.01553
315
## 4       0.3715939        0.1414327     94.62562      36.01553
315
## 5       0.2539263        0.1310499     64.66179      33.37158
238
```

```
## 6       0.2539263        0.1310499     64.66179      33.37158
238
##   Duration.ms._10mean scalar_max_10mean scalar_mean_10mean
## 1            3.888889          15.49537           13.49979
## 2            3.888889          15.49537           13.49979
## 3            7.706349          16.38244           13.65682
## 4            7.706349          16.38244           13.65682
## 5            7.415966          15.56483           13.15894
## 6            7.415966          15.56483           13.15894
##   changescalar_max_10mean changescalar_mean_10mean Changevector_max_10mean
## 1                6.557340                 3.249119                8.029862
## 2                6.557340                 3.249119                8.029862
## 3                4.928854                 1.971893                6.505797
## 4                4.928854                 1.971893                6.505797
## 5                4.004025                 1.664277                5.891075
## 6                4.004025                 1.664277                5.891075
##   Changevector_mean_10mean Angle_max_10mean Angle_mean_10mean
Hz_max_10mean
## 1                 4.538349        0.4719538         0.2224823
120.1821
## 2                 4.538349        0.4719538         0.2224823
120.1821
## 3                 3.339135        0.4047630         0.1751144
103.0721
## 4                 3.339135        0.4047630         0.1751144
103.0721
## 5                 2.909783        0.3492638         0.1583697
88.9393
## 6                 2.909783        0.3492638         0.1583697
88.9393
##   Hz_mean_10mean Events_15mean Duration.ms._15mean scalar_max_15mean
## 1       56.65466           144            3.441840          19.26462
## 2       56.65466           144            3.441840          19.26462
## 3       44.59251           183            6.342213          19.93954
## 4       44.59251           183            6.342213          19.93954
## 5       40.32851           111            6.920045          19.71768
## 6       40.32851           111            6.920045          19.71768
##   scalar_mean_15mean changescalar_max_15mean changescalar_mean_15mean
## 1           17.94158                8.195410                 4.209463
## 2           17.94158                8.195410                 4.209463
## 3           17.79343                6.271208                 2.424063
## 4           17.79343                6.271208                 2.424063
## 5           17.60073                5.291784                 2.055466
## 6           17.60073                5.291784                 2.055466
##   Changevector_max_15mean Changevector_mean_15mean Angle_max_15mean
## 1               10.145769                 6.194357        0.4732660
## 2               10.145769                 6.194357        0.4732660
## 3                8.773143                 4.485467        0.4584332
## 4                8.773143                 4.485467        0.4584332
## 5                8.644509                 4.178605        0.4437400
```

```
## 6                8.644509                 4.178605        0.4437400
##   Angle_mean_15mean Hz_max_15mean Hz_mean_15mean Events_20mean
## 1         0.2644405      120.5162       67.33922            57
## 2         0.2644405      120.5162       67.33922            57
## 3         0.2042285      116.7390       52.00635            75
## 4         0.2042285      116.7390       52.00635            75
## 5         0.1960600      112.9975       49.92626            42
## 6         0.1960600      112.9975       49.92626            42
##   Duration.ms._20mean scalar_max_20mean scalar_mean_20mean
## 1            2.587719          22.76440           22.04857
## 2            2.587719          22.76440           22.04857
## 3            5.250000          23.71752           22.29175
## 4            5.250000          23.71752           22.29175
## 5            5.997024          24.28693           22.58697
## 6            5.997024          24.28693           22.58697
##   changescalar_max_20mean changescalar_mean_20mean Changevector_max_20mean
## 1                7.502070                 3.960703                11.35123
## 2                7.502070                 3.960703                11.35123
## 3                6.190293                 2.676377                11.36833
## 4                6.190293                 2.676377                11.36833
## 5                5.996190                 2.445063                11.97291
## 6                5.996190                 2.445063                11.97291
##   Changevector_mean_20mean Angle_max_20mean Angle_mean_20mean
Hz_max_20mean
## 1                 6.922532        0.5201012         0.3140733
132.4427
## 2                 6.922532        0.5201012         0.3140733
132.4427
## 3                 5.877764        0.5214004         0.2548023
132.7735
## 4                 5.877764        0.5214004         0.2548023
132.7735
## 5                 5.476046        0.5391966         0.2201107
137.3053
## 6                 5.476046        0.5391966         0.2201107
137.3053
##   Hz_mean_20mean RH._Max Temp_Max RH._mean Temp_mean
## 1       79.97812   47.08    23.50    41.69     19.73
## 2       79.97812   60.14    27.50    41.69     19.73
## 3       64.88487   62.61    29.75    53.55     21.79
## 4       64.88487   68.10    32.00    53.55     21.79
## 5       56.05073   57.37    28.69    53.25     23.29
## 6       56.05073   74.59    19.25    53.25     23.29
```

```
summary(tab)
```

```
##       Date         Replicate              Age         Treatments
##  Min.   :170510   Length:22          Min.   :22.00   Length:22
##  1st Qu.:170526   Class :character   1st Qu.:22.00   Class :character
##  Median :180620   Mode  :character   Median :29.00   Mode  :character
```

```
##  Mean   :176081                      Mean   :25.82
##  3rd Qu.:180716                      3rd Qu.:29.00
##  Max.   :180801                      Max.   :29.00
##     not_full          full             MSV              MSV2
##  Min.   : 2.00   Min.   : 0.000   Min.   :0.2500   Min.   :0.5000
##  1st Qu.:11.00   1st Qu.: 2.000   1st Qu.:0.4025   1st Qu.:0.8050
##  Median :13.50   Median : 5.500   Median :0.4694   Median :0.9387
##  Mean   :13.82   Mean   : 5.955   Mean   :0.4859   Mean   :0.9718
##  3rd Qu.:17.75   3rd Qu.: 7.750   3rd Qu.:0.5681   3rd Qu.:1.1362
##  Max.   :26.00   Max.   :22.000   Max.   :0.7500   Max.   :1.5000
##    shipped          Irradiation         Events._5max    Duration.ms._5max
##  Length:22          Length:22          Min.   : 341.0   Min.   : 41.88
##  Class :character   Class :character   1st Qu.: 495.0   1st Qu.: 49.06
##  Mode  :character   Mode  :character   Median : 708.0   Median : 59.38
##                                        Mean   : 763.6   Mean   : 76.76
##                                        3rd Qu.:1117.5   3rd Qu.: 97.34
##                                        Max.   :1228.0   Max.   :150.00
##  scalar_max_5max scalar_mean_5max changescalar_max_5max
changescalar_mean_5max
##  Min.   :27.28   Min.   :15.44    Min.   :15.58         Min.   : 3.838
##  1st Qu.:27.37   1st Qu.:17.64    1st Qu.:21.16         1st Qu.:10.623
##  Median :27.37   Median :19.35    Median :23.03         Median :15.391
##  Mean   :27.37   Mean   :20.67    Mean   :22.10         Mean   :15.724
##  3rd Qu.:27.40   3rd Qu.:23.95    3rd Qu.:24.29         3rd Qu.:23.124
##  Max.   :27.40   Max.   :27.13    Max.   :26.65         Max.   :26.175
##  Changevector_max_5max Changevector_mean_5max Angle_max_5max
Angle_mean_5max
##  Min.   :23.56         Min.   : 5.064         Min.   :2.106   Min.
:1.356
##  1st Qu.:29.49         1st Qu.:11.063         1st Qu.:2.505   1st
Qu.:1.682
##  Median :35.07         Median :19.036         Median :2.969   Median
:2.527
##  Mean   :34.55         Mean   :17.786         Mean   :2.795   Mean
:2.304
##  3rd Qu.:38.57         3rd Qu.:23.722         3rd Qu.:3.023   3rd
Qu.:2.689
##  Max.   :43.01         Max.   :28.208         Max.   :3.132   Max.
:3.057
##   Hz_max_5max     Hz_mean_5max    Events_10max   Duration.ms._10max
##  Min.   :536.3   Min.   :345.3   Min.   :109.0   Min.   :23.75
##  1st Qu.:637.8   1st Qu.:428.4   1st Qu.:144.0   1st Qu.:34.69
##  Median :756.1   Median :643.4   Median :238.0   Median :37.50
##  Mean   :711.8   Mean   :586.8   Mean   :285.4   Mean   :41.36
##  3rd Qu.:769.9   3rd Qu.:684.9   3rd Qu.:308.2   3rd Qu.:50.94
##  Max.   :797.5   Max.   :778.4   Max.   :956.0   Max.   :63.75
##  scalar_max_10max scalar_mean_10max changescalar_max_10max
##  Min.   :27.28    Min.   :17.45     Min.   :15.58
##  1st Qu.:27.37    1st Qu.:20.45     1st Qu.:17.76
##  Median :27.37    Median :21.55     Median :21.91
```

```
##  Mean   :27.37    Mean   :22.17     Mean   :21.09
##  3rd Qu.:27.40    3rd Qu.:23.99     3rd Qu.:23.69
##  Max.   :27.40    Max.   :27.37     Max.   :25.20
##  changescalar_mean_10max Changevector_max_10max Changevector_mean_10max
##  Min.   : 8.965          Min.   :23.56          Min.   :10.66
##  1st Qu.:11.337          1st Qu.:26.93          1st Qu.:12.98
##  Median :16.488          Median :33.00          Median :19.71
##  Mean   :15.891          Mean   :33.10          Mean   :18.75
##  3rd Qu.:20.581          3rd Qu.:38.57          3rd Qu.:24.61
##  Max.   :25.203          Max.   :43.01          Max.   :26.96
##  Angle_max_10max Angle_mean_10max  Hz_max_10max   Hz_mean_10max
##  Min.   :1.734   Min.   :0.5721   Min.   :441.5   Min.   :145.7
##  1st Qu.:2.303   1st Qu.:1.1168   1st Qu.:586.4   1st Qu.:284.4
##  Median :2.570   Median :1.6457   Median :654.4   Median :419.1
##  Mean   :2.570   Mean   :1.7194   Mean   :654.4   Mean   :437.8
##  3rd Qu.:3.007   3rd Qu.:2.4297   3rd Qu.:765.7   3rd Qu.:618.7
##  Max.   :3.057   Max.   :3.0198   Max.   :778.5   Max.   :769.0
##   Events_15max    Duration.ms._15max scalar_max_15max scalar_mean_15max
##  Min.   : 54.00   Min.   :18.75      Min.   :27.28    Min.   :21.72
##  1st Qu.: 62.75   1st Qu.:20.16      1st Qu.:27.37    1st Qu.:22.52
##  Median :129.00   Median :22.50      Median :27.37    Median :25.17
##  Mean   :159.45   Mean   :26.82      Mean   :27.37    Mean   :24.79
##  3rd Qu.:161.00   3rd Qu.:30.62      3rd Qu.:27.40    3rd Qu.:27.06
##  Max.   :627.00   Max.   :41.25      Max.   :27.40    Max.   :27.37
##  changescalar_max_15max changescalar_mean_15max Changevector_max_15max
##  Min.   :15.58          Min.   : 9.943          Min.   :23.56
##  1st Qu.:21.16          1st Qu.:12.522          1st Qu.:26.93
##  Median :23.03          Median :16.540          Median :35.07
##  Mean   :22.10          Mean   :18.188          Mean   :34.48
##  3rd Qu.:24.29          3rd Qu.:23.193          3rd Qu.:40.88
##  Max.   :26.65          Max.   :26.513          Max.   :44.89
##  Changevector_mean_15max Angle_max_15max Angle_mean_15max  Hz_max_15max
##  Min.   :12.84           Min.   :1.326   Min.   :0.6202   Min.   :337.7
##  1st Qu.:14.73           1st Qu.:2.189   1st Qu.:0.8599   1st Qu.:557.4
##  Median :21.62           Median :2.687   Median :1.8428   Median :684.3
##  Mean   :21.59           Mean   :2.484   Mean   :1.8436   Mean   :632.5
##  3rd Qu.:26.86           3rd Qu.:2.942   3rd Qu.:2.7354   3rd Qu.:749.1
##  Max.   :30.34           Max.   :3.125   Max.   :3.1249   Max.   :795.7
##  Hz_mean_15max    Events_20max   Duration.ms._20max scalar_max_20max
##  Min.   :157.9   Min.   : 23.0   Min.   :10.62      Min.   :27.28
##  1st Qu.:219.0   1st Qu.: 29.5   1st Qu.:13.44      1st Qu.:27.37
##  Median :469.3   Median : 57.0   Median :16.88      Median :27.37
##  Mean   :469.5   Mean   : 67.0   Mean   :16.88      Mean   :27.37
##  3rd Qu.:696.6   3rd Qu.: 74.0   3rd Qu.:19.84      3rd Qu.:27.40
##  Max.   :795.7   Max.   :215.0   Max.   :28.12      Max.   :27.40
##  scalar_mean_20max changescalar_max_20max changescalar_mean_20max
##  Min.   :24.72     Min.   :13.48          Min.   : 5.401
##  1st Qu.:25.87     1st Qu.:21.34          1st Qu.:15.570
##  Median :26.83     Median :23.03          Median :22.061
##  Mean   :26.55     Mean   :22.23          Mean   :19.313
```

```
##  3rd Qu.:27.37     3rd Qu.:24.29          3rd Qu.:23.686
##  Max.   :27.37     Max.   :26.65          Max.   :26.513
##  Changevector_max_20max Changevector_mean_20max Angle_max_20max
##  Min.   :23.56          Min.   :11.19           Min.   :1.326
##  1st Qu.:27.87          1st Qu.:25.33           1st Qu.:1.844
##  Median :33.00          Median :27.68           Median :2.457
##  Mean   :33.31          Mean   :26.20           Mean   :2.357
##  3rd Qu.:38.32          3rd Qu.:28.17           3rd Qu.:2.800
##  Max.   :44.89          Max.   :34.48           Max.   :3.062
##  Angle_mean_20max  Hz_max_20max     Hz_mean_20max      Events_5mean
##  Min.   :0.4892   Min.   :  3.057   Min.   :  2.671   Min.   : 341.0
##  1st Qu.:1.4039   1st Qu.:439.219   1st Qu.:311.298   1st Qu.: 495.0
##  Median :2.2315   Median :619.756   Median :456.569   Median : 708.0
##  Mean   :2.0118   Mean   :529.617   Mean   :450.696   Mean   : 763.6
##  3rd Qu.:2.6834   3rd Qu.:670.696   3rd Qu.:662.651   3rd Qu.:1117.5
##  Max.   :2.9219   Max.   :779.845   Max.   :744.045   Max.   :1228.0
##  Duration.ms._5mean scalar_max_5mean scalar_mean_5mean
changescalar_max_5mean
##  Min.   : 5.215     Min.   : 7.866   Min.   :6.507     Min.   :1.442
##  1st Qu.: 6.909     1st Qu.: 9.178   1st Qu.:7.410     1st Qu.:2.339
##  Median : 8.597     Median : 9.648   Median :7.565     Median :2.816
##  Mean   : 8.653     Mean   : 9.743   Mean   :7.438     Mean   :3.378
##  3rd Qu.:10.250     3rd Qu.:10.434   3rd Qu.:7.682     3rd Qu.:3.632
##  Max.   :11.786     Max.   :12.092   Max.   :8.188     Max.   :8.696
##  changescalar_mean_5mean Changevector_max_5mean Changevector_mean_5mean
##  Min.   :0.6227          Min.   : 1.767         Min.   :0.8316
##  1st Qu.:0.8770          1st Qu.: 2.939         1st Qu.:1.3240
##  Median :1.1437          Median : 3.692         Median :1.6388
##  Mean   :1.6777          Mean   : 4.463         Mean   :2.3524
##  3rd Qu.:1.6224          3rd Qu.: 4.665         3rd Qu.:2.1882
##  Max.   :4.5129          Max.   :12.182         Max.   :6.4512
##  Angle_max_5mean  Angle_mean_5mean   Hz_max_5mean    Hz_mean_5mean
##  Min.   :0.1659   Min.   :0.07985   Min.   : 42.24   Min.   : 20.33
##  1st Qu.:0.2672   1st Qu.:0.13365   1st Qu.: 68.03   1st Qu.: 34.03
##  Median :0.3716   Median :0.16791   Median : 94.63   Median : 42.76
##  Mean   :0.5221   Mean   :0.28474   Mean   :132.96   Mean   : 72.51
##  3rd Qu.:0.4295   3rd Qu.:0.21880   3rd Qu.:109.37   3rd Qu.: 55.72
##  Max.   :1.7833   Max.   :0.94024   Max.   :454.12   Max.   :239.43
##  Events._10mean  Duration.ms._10mean scalar_max_10mean scalar_mean_10mean
##  Min.   :109.0   Min.   :3.532       Min.   :14.94     Min.   :12.92
##  1st Qu.:144.0   1st Qu.:5.091       1st Qu.:15.57     1st Qu.:13.38
##  Median :238.0   Median :7.046       Median :15.75     Median :13.66
##  Mean   :285.4   Mean   :6.549       Mean   :16.07     Mean   :13.75
##  3rd Qu.:308.2   3rd Qu.:7.710       3rd Qu.:16.60     3rd Qu.:14.10
##  Max.   :956.0   Max.   :8.695       Max.   :17.47     Max.   :14.79
##  changescalar_max_10mean changescalar_mean_10mean Changevector_max_10mean
##  Min.   : 3.380          Min.   :1.479            Min.   : 4.344
##  1st Qu.: 4.173          1st Qu.:1.897            1st Qu.: 6.000
##  Median : 4.929          Median :2.288            Median : 6.506
##  Mean   : 5.991          Mean   :3.423            Mean   : 8.003
```

```
##  3rd Qu.: 6.468         3rd Qu.:3.775          3rd Qu.: 8.712
##  Max.   :12.085         Max.   :9.086          Max.   :16.709
##  Changevector_mean_10mean Angle_max_10mean Angle_mean_10mean Hz_max_10mean
##  Min.   : 2.170          Min.   :0.2465   Min.   :0.1225    Min.   :
62.78
##  1st Qu.: 2.923          1st Qu.:0.3279   1st Qu.:0.1414    1st Qu.:
83.50
##  Median : 3.339          Median :0.4504   Median :0.2215    Median
:114.68
##  Mean   : 4.881          Mean   :0.5915   Mean   :0.3678    Mean
:150.62
##  3rd Qu.: 5.319          3rd Qu.:0.5606   3rd Qu.:0.3422    3rd
Qu.:142.76
##  Max.   :13.024          Max.   :1.8310   Max.   :1.3785    Max.
:466.26
##  Hz_mean_10mean   Events_15mean    Duration.ms._15mean scalar_max_15mean
##  Min.   : 31.20   Min.   : 54.00   Min.   :2.310       Min.   :19.16
##  1st Qu.: 36.02   1st Qu.: 62.75   1st Qu.:4.485       1st Qu.:19.57
##  Median : 56.39   Median :129.00   Median :6.215       Median :19.78
##  Mean   : 93.65   Mean   :159.45   Mean   :5.518       Mean   :19.97
##  3rd Qu.: 87.15   3rd Qu.:161.00   3rd Qu.:6.617       3rd Qu.:20.47
##  Max.   :351.03   Max.   :627.00   Max.   :6.920       Max.   :21.17
##  scalar_mean_15mean changescalar_max_15mean changescalar_mean_15mean
##  Min.   :17.60      Min.   : 4.622          Min.   : 1.704
##  1st Qu.:17.83      1st Qu.: 5.493          1st Qu.: 2.300
##  Median :18.27      Median : 6.393          Median : 2.959
##  Mean   :18.18      Mean   : 7.567          Mean   : 4.400
##  3rd Qu.:18.53      3rd Qu.: 8.247          3rd Qu.: 5.211
##  Max.   :18.71      Max.   :14.038          Max.   :11.493
##  Changevector_max_15mean Changevector_mean_15mean Angle_max_15mean
##  Min.   : 6.224         Min.   : 2.939           Min.   :0.2772
##  1st Qu.: 8.675         1st Qu.: 4.075           1st Qu.:0.3934
##  Median : 9.016         Median : 4.903           Median :0.4733
##  Mean   :10.393         Mean   : 6.549           Mean   :0.6328
##  3rd Qu.:11.444         3rd Qu.: 7.425           3rd Qu.:0.6286
##  Max.   :19.029         Max.   :16.285           Max.   :1.7973
##  Angle_mean_15mean Hz_max_15mean    Hz_mean_15mean   Events_20mean
##  Min.   :0.1236    Min.   : 70.58   Min.   : 31.48   Min.   : 23.0
##  1st Qu.:0.1790    1st Qu.:100.19   1st Qu.: 45.59   1st Qu.: 29.5
##  Median :0.2644    Median :120.52   Median : 67.34   Median : 57.0
##  Mean   :0.4088    Mean   :161.14   Mean   :104.11   Mean   : 67.0
##  3rd Qu.:0.4098    3rd Qu.:160.08   3rd Qu.:104.37   3rd Qu.: 74.0
##  Max.   :1.4887    Max.   :457.68   Max.   :379.09   Max.   :215.0
##  Duration.ms._20mean scalar_max_20mean scalar_mean_20mean
##  Min.   :1.663       Min.   :22.50     Min.   :21.81
##  1st Qu.:3.989       1st Qu.:22.84     1st Qu.:22.17
##  Median :4.344       Median :23.45     Median :22.25
##  Mean   :4.264       Mean   :23.43     Mean   :22.31
##  3rd Qu.:5.133       3rd Qu.:23.99     3rd Qu.:22.53
##  Max.   :5.997       Max.   :24.29     Max.   :22.86
```

```
##  changescalar_max_20mean changescalar_mean_20mean Changevector_max_20mean
##  Min.   : 5.234          Min.   : 2.159           Min.   : 7.482
##  1st Qu.: 5.531          1st Qu.: 2.780           1st Qu.: 9.975
##  Median : 7.212          Median : 3.869           Median :11.368
##  Mean   : 7.824          Mean   : 4.883           Mean   :12.189
##  3rd Qu.: 8.123          3rd Qu.: 5.590           3rd Qu.:12.832
##  Max.   :14.139          Max.   :12.105           Max.   :19.387
##  Changevector_mean_20mean Angle_max_20mean Angle_mean_20mean Hz_max_20mean
##  Min.   : 4.828           Min.   :0.3096   Min.   :0.2178    Min.   :
0.5016
##  1st Qu.: 5.527           1st Qu.:0.4961   1st Qu.:0.2321    1st
Qu.:114.2034
##  Median : 6.843           Median :0.5214   Median :0.3023    Median
:132.7735
##  Mean   : 7.865           Mean   :0.6046   Mean   :0.3945    Mean
:142.5937
##  3rd Qu.: 8.273           3rd Qu.:0.5788   3rd Qu.:0.3688    3rd
Qu.:147.3906
##  Max.   :17.154           Max.   :1.2518   Max.   :1.0993    Max.
:318.7755
##  Hz_mean_20mean        RH._Max         Temp_Max        RH._mean
##  Min.   :  0.3079   Min.   :47.08   Min.   :19.25   Min.   :41.69
##  1st Qu.: 56.3355   1st Qu.:60.76   1st Qu.:24.53   1st Qu.:50.83
##  Median : 75.5923   Median :73.52   Median :27.38   Median :71.53
##  Mean   : 93.4190   Mean   :69.60   Mean   :26.80   Mean   :62.64
##  3rd Qu.: 93.9064   3rd Qu.:78.33   3rd Qu.:29.48   3rd Qu.:72.73
##  Max.   :279.9378   Max.   :84.59   Max.   :32.00   Max.   :77.90
##    Temp_mean
##  Min.   :10.22
##  1st Qu.:12.61
##  Median :15.36
##  Mean   :17.33
##  3rd Qu.:22.23
##  Max.   :24.58
```

```
str(tab)
```

```
## 'data.frame':    22 obs. of  110 variables:
##  $ Date                    : int  170510 170510 170517 170517 170524
170524 170531 170531 170607 170607 ...
##  $ Replicate               : chr  "R1" "R1" "R2" "R2" ...
##  $ Age                     : int  22 22 22 22 22 22 22 22 22 22 ...
##  $ Treatments              : chr  "Shipped-110Gy " "Shipped-0Gy "
"Shipped-110Gy " "Shipped-0Gy " ...
##  $ not_full                : int  2 4 22 13 6 22 4 14 11 17 ...
##  $ full                    : int  6 6 5 17 7 4 0 9 8 22 ...
##  $ MSV                     : num  0.75 0.7 0.41 0.66 0.6 0.42 0.25 0.57
0.47 0.66 ...
##  $ MSV2                    : num  1.5 1.4 0.82 1.32 1.2 0.84 0.5 1.14 0.94
1.32 ...
```

```
##  $ shipped                  : chr  "yes" "yes" "yes" "yes" ...
##  $ Irradiation              : chr  "yes" "no" "yes" "no" ...
##  $ Events._5max             : int  909 909 708 708 1187 1187 625 625 606
606 ...
##  $ Duration.ms._5max        : num  48.8 48.8 87.5 87.5 93.1 ...
##  $ scalar_max_5max          : num  27.4 27.4 27.4 27.4 27.4 ...
##  $ scalar_mean_5max         : num  21.4 21.4 16.9 16.9 15.4 ...
##  $ changescalar_max_5max    : num  23.7 23.7 24.5 24.5 16.6 ...
##  $ changescalar_mean_5max   : num  8.95 8.95 11.38 11.38 3.84 ...
##  $ Changevector_max_5max    : num  27.7 27.7 35.1 35.1 33 ...
##  $ Changevector_mean_5max   : num  9.94 9.94 19.04 19.04 5.06 ...
##  $ Angle_max_5max           : num  3 3 2.97 2.97 2.11 ...
##  $ Angle_mean_5max          : num  2.62 2.62 1.68 1.68 1.36 ...
##  $ Hz_max_5max              : num  764 764 756 756 536 ...
##  $ Hz_mean_5max             : num  666 666 428 428 345 ...
##  $ Events_10max             : int  288 288 315 315 238 238 261 261 219 219
...
##  $ Duration.ms._10max       : num  36.9 36.9 50 50 63.8 ...
##  $ scalar_max_10max         : num  27.4 27.4 27.4 27.4 27.4 ...
##  $ scalar_mean_10max        : num  21.6 21.6 20.4 20.4 19.7 ...
##  $ changescalar_max_10max   : num  23.7 23.7 24.5 24.5 16.6 ...
##  $ changescalar_mean_10max  : num  12.47 12.47 16.49 16.49 8.97 ...
##  $ Changevector_max_10max   : num  27.7 27.7 35.1 35.1 33 ...
##  $ Changevector_mean_10max  : num  15.5 15.5 22.6 22.6 10.7 ...
##  $ Angle_max_10max          : num  2.69 2.69 2.97 2.97 2.11 ...
##  $ Angle_mean_10max         : num  1.047 1.047 1.614 1.614 0.585 ...
##  $ Hz_max_10max             : num  684 684 756 756 536 ...
##  $ Hz_mean_10max            : num  267 267 411 411 149 ...
##  $ Events_15max             : int  144 144 183 183 111 111 163 163 129 129
...
##  $ Duration.ms._15max       : num  30.6 30.6 29.4 29.4 41.2 ...
##  $ scalar_max_15max         : num  27.4 27.4 27.4 27.4 27.4 ...
##  $ scalar_mean_15max        : num  22.4 22.4 21.7 21.7 22.8 ...
##  $ changescalar_max_15max   : num  23.7 23.7 24.5 24.5 16.6 ...
##  $ changescalar_mean_15max  : num  15.7 15.7 16.5 16.5 11.5 ...
##  $ Changevector_max_15max   : num  27.7 27.7 35.1 35.1 33 ...
##  $ Changevector_mean_15max  : num  19.8 19.8 21.6 21.6 12.8 ...
##  $ Angle_max_15max          : num  2.69 2.69 2.97 2.97 2.11 ...
##  $ Angle_mean_15max         : num  1.843 1.843 1.601 1.601 0.671 ...
##  $ Hz_max_15max             : num  684 684 756 756 536 ...
##  $ Hz_mean_15max            : num  469 469 408 408 171 ...
##  $ Events_20max             : int  57 57 75 75 42 42 97 97 71 71 ...
##  $ Duration.ms._20max       : num  17.5 17.5 20.6 20.6 20.6 ...
##  $ scalar_max_20max         : num  27.4 27.4 27.4 27.4 27.4 ...
##  $ scalar_mean_20max        : num  26.8 26.8 26.4 26.4 25.2 ...
##  $ changescalar_max_20max   : num  23.7 23.7 24.5 24.5 13.5 ...
##  $ changescalar_mean_20max  : num  23.72 23.72 13.71 13.71 6.61 ...
##  $ Changevector_max_20max   : num  27.7 27.7 35.1 35.1 33 ...
##  $ Changevector_mean_20max  : num  27.7 27.7 29.7 29.7 11.2 ...
##  $ Angle_max_20max          : num  2.69 2.69 2.43 2.43 1.72 ...
```

```
##  $ Angle_mean_20max        : num  2.687 2.687 1.793 1.793 0.489 ...
##  $ Hz_max_20max            : num  684 684 620 620 437 ...
##  $ Hz_mean_20max           : num  684 684 457 457 125 ...
##  $ Events_5mean            : int  909 909 708 708 1187 1187 625 625 606
606 ...
##  $ Duration.ms._5mean      : num  5.22 5.22 11.79 11.79 7.35 ...
##  $ scalar_max_5mean        : num  9.52 9.52 10.55 10.55 8.02 ...
##  $ scalar_mean_5mean       : num  7.47 7.47 7.57 7.57 6.51 ...
##  $ changescalar_max_5mean  : num  3.76 3.76 2.82 2.82 1.63 ...
##  $ changescalar_mean_5mean : num  1.66 1.66 1.01 1.01 0.68 ...
##  $ Changevector_max_5mean  : num  4.5 4.5 3.69 3.69 2.29 ...
##  $ Changevector_mean_5mean : num  2.22 2.22 1.64 1.64 1.19 ...
##  $ Angle_max_5mean         : num  0.43 0.43 0.372 0.372 0.254 ...
##  $ Angle_mean_5mean        : num  0.176 0.176 0.141 0.141 0.131 ...
##  $ Hz_max_5mean            : num  109.4 109.4 94.6 94.6 64.7 ...
##  $ Hz_mean_5mean           : num  44.7 44.7 36 36 33.4 ...
##  $ Events._10mean          : int  288 288 315 315 238 238 261 261 219 219
...
##  $ Duration.ms._10mean     : num  3.89 3.89 7.71 7.71 7.42 ...
##  $ scalar_max_10mean       : num  15.5 15.5 16.4 16.4 15.6 ...
##  $ scalar_mean_10mean      : num  13.5 13.5 13.7 13.7 13.2 ...
##  $ changescalar_max_10mean : num  6.56 6.56 4.93 4.93 4 ...
##  $ changescalar_mean_10mean: num  3.25 3.25 1.97 1.97 1.66 ...
##  $ Changevector_max_10mean : num  8.03 8.03 6.51 6.51 5.89 ...
##  $ Changevector_mean_10mean: num  4.54 4.54 3.34 3.34 2.91 ...
##  $ Angle_max_10mean        : num  0.472 0.472 0.405 0.405 0.349 ...
##  $ Angle_mean_10mean       : num  0.222 0.222 0.175 0.175 0.158 ...
##  $ Hz_max_10mean           : num  120.2 120.2 103.1 103.1 88.9 ...
##  $ Hz_mean_10mean          : num  56.7 56.7 44.6 44.6 40.3 ...
##  $ Events_15mean           : int  144 144 183 183 111 111 163 163 129 129
...
##  $ Duration.ms._15mean     : num  3.44 3.44 6.34 6.34 6.92 ...
##  $ scalar_max_15mean       : num  19.3 19.3 19.9 19.9 19.7 ...
##  $ scalar_mean_15mean      : num  17.9 17.9 17.8 17.8 17.6 ...
##  $ changescalar_max_15mean : num  8.2 8.2 6.27 6.27 5.29 ...
##  $ changescalar_mean_15mean: num  4.21 4.21 2.42 2.42 2.06 ...
##  $ Changevector_max_15mean : num  10.15 10.15 8.77 8.77 8.64 ...
##  $ Changevector_mean_15mean: num  6.19 6.19 4.49 4.49 4.18 ...
##  $ Angle_max_15mean        : num  0.473 0.473 0.458 0.458 0.444 ...
##  $ Angle_mean_15mean       : num  0.264 0.264 0.204 0.204 0.196 ...
##  $ Hz_max_15mean           : num  121 121 117 117 113 ...
##  $ Hz_mean_15mean          : num  67.3 67.3 52 52 49.9 ...
##  $ Events_20mean           : int  57 57 75 75 42 42 97 97 71 71 ...
##  $ Duration.ms._20mean     : num  2.59 2.59 5.25 5.25 6 ...
##  $ scalar_max_20mean       : num  22.8 22.8 23.7 23.7 24.3 ...
##  $ scalar_mean_20mean      : num  22 22 22.3 22.3 22.6 ...
##  $ changescalar_max_20mean : num  7.5 7.5 6.19 6.19 6 ...
##   [list output truncated]
```

```
boxplot(tab$MSV ~ tab$Treatments, xlab ="Treatments", ylab = "Mean
Spermathecal Value(MSV)")
```

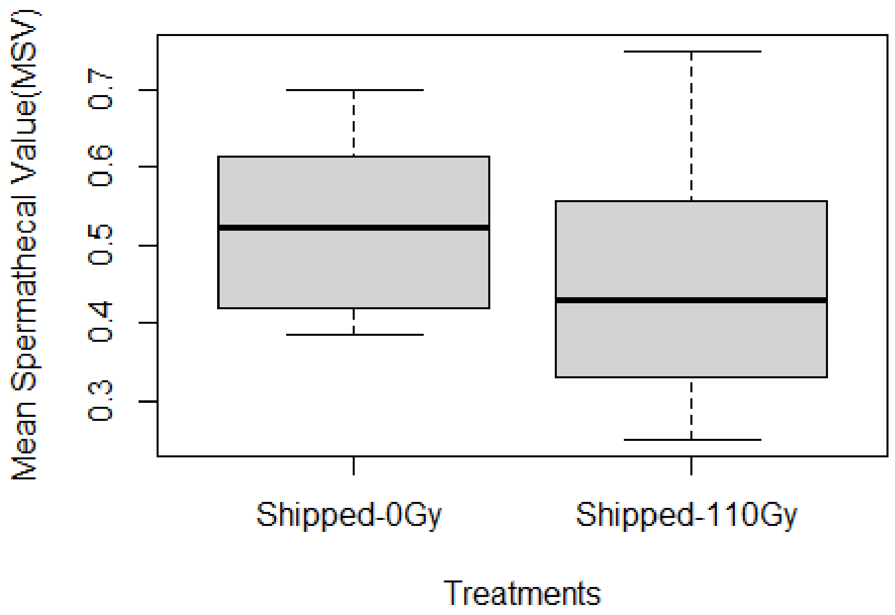


```
na.omit(tab)

##      Date Replicate Age    Treatments not_full full       MSV      MSV2
## 1  170510        R1  22 Shipped-110Gy        2    6 0.7500000 1.5000000
## 2  170510        R1  22   Shipped-0Gy        4    6 0.7000000 1.4000000
## 3  170517        R2  22 Shipped-110Gy       22    5 0.4100000 0.8200000
## 4  170517        R2  22   Shipped-0Gy       13   17 0.6600000 1.3200000
## 5  170524        R3  22 Shipped-110Gy        6    7 0.6000000 1.2000000
## 6  170524        R3  22   Shipped-0Gy       22    4 0.4200000 0.8400000
## 7  170531        R4  22 Shipped-110Gy        4    0 0.2500000 0.5000000
## 8  170531        R4  22   Shipped-0Gy       14    9 0.5700000 1.1400000
## 9  170607        R5  22 Shipped-110Gy       11    8 0.4700000 0.9400000
## 10 170607        R5  22   Shipped-0Gy       17   22 0.6600000 1.3200000
## 11 180620        R1  29 Shipped-110Gy       24    0 0.3333333 0.6666667
## 12 180620        R1  29   Shipped-0Gy       18    2 0.4000000 0.8000000
## 13 180627        R2  29 Shipped-110Gy       13   11 0.5520833 1.1041667
## 14 180627        R2  29   Shipped-0Gy       11    4 0.4166667 0.8333333
## 15 180711        R3  29 Shipped-110Gy       11    3 0.4285714 0.8571429
## 16 180711        R3  29   Shipped-0Gy       11    2 0.3846154 0.7692308
## 17 180717        R4  29 Shipped-110Gy       11    9 0.5625000 1.1250000
## 18 180717        R4  29   Shipped-0Gy       15    6 0.5238095 1.0476190
## 19 180725        R5  29 Shipped-110Gy       15    1 0.2656250 0.5312500
## 20 180725        R5  29   Shipped-0Gy       14    2 0.4687500 0.9375000
## 21 180801        R6  29 Shipped-110Gy       26    0 0.3269231 0.6538462
```

```
## 22 180801        R6  29   Shipped-0Gy        20    7 0.5370370 1.0740741
##    shipped Irradiation Events._5max Duration.ms._5max scalar_max_5max
## 1      yes         yes          909            48.750          27.374
## 2      yes          no          909            48.750          27.374
## 3      yes         yes          708            87.500          27.398
## 4      yes          no          708            87.500          27.398
## 5      yes         yes         1187            93.125          27.374
## 6      yes          no         1187            93.125          27.374
## 7      yes         yes          625           117.500          27.398
## 8      yes          no          625           117.500          27.398
## 9      yes         yes          606            98.750          27.398
## 10     yes          no          606            98.750          27.398
## 11     yes         yes          344            45.000          27.374
## 12     yes          no          344            45.000          27.374
## 13     yes         yes         1197            50.000          27.398
## 14     yes          no         1197            50.000          27.398
## 15     yes         yes          458            52.500          27.280
## 16     yes          no          458            52.500          27.280
## 17     yes         yes         1228           150.000          27.374
## 18     yes          no         1228           150.000          27.374
## 19     yes         yes          341            59.375          27.374
## 20     yes          no          341            59.375          27.374
## 21     yes         yes          797            41.875          27.374
## 22     yes          no          797            41.875          27.374
##    scalar_mean_5max changescalar_max_5max changescalar_mean_5max
## 1          21.43900                23.724                8.94875
## 2          21.43900                23.724                8.94875
## 3          16.92723                24.477               11.37550
## 4          16.92723                24.477               11.37550
## 5          15.43705                16.627                3.83800
## 6          15.43705                16.627                3.83800
## 7          19.34543                21.153               16.54000
## 8          19.34543                21.153               16.54000
## 9          19.15606                21.177               10.38500
## 10         19.15606                21.177               10.38500
## 11         18.22700                23.031               15.39086
## 12         18.22700                23.031               15.39086
## 13         23.98200                25.203               23.62100
## 14         23.98200                25.203               23.62100
## 15         24.43700                23.570               23.57000
## 16         24.43700                23.570               23.57000
## 17         27.13300                26.650               26.17500
## 18         27.13300                26.650               26.17500
## 19         23.85200                21.912               21.78600
## 20         23.85200                21.912               21.78600
## 21         17.44762                15.583               11.33700
## 22         17.44762                15.583               11.33700
##    Changevector_max_5max Changevector_mean_5max Angle_max_5max
Angle_mean_5max
## 1               27.69156               9.937386       3.002116
```

```
2.617132
## 2                27.69156              9.937386        3.002116
2.617132
## 3                35.06987             19.035967        2.969360
1.679485
## 4                35.06987             19.035967        2.969360
1.679485
## 5                33.00443              5.064434        2.105922
1.356174
## 6                33.00443              5.064434        2.105922
1.356174
## 7                43.01068             18.255955        3.019490
2.526598
## 8                43.01068             18.255955        3.019490
2.526598
## 9                38.58406             10.469185        2.437353
1.690084
## 10               38.58406             10.469185        2.437353
1.690084
## 11               28.39364             20.815676        3.057269
2.410337
## 12               28.39364             20.815676        3.057269
2.410337
## 13               38.53399             24.007565        3.024598
3.019751
## 14               38.53399             24.007565        3.024598
3.019751
## 15               41.64894             23.940939        2.706695
2.706695
## 16               41.64894             23.940939        2.706695
2.706695
## 17               37.75116             28.208132        3.131724
3.056608
## 18               37.75116             28.208132        3.131724
3.056608
## 19               32.78848             23.063334        2.895380
2.637554
## 20               32.78848             23.063334        2.895380
2.637554
## 21               23.55997             12.844674        2.397547
1.645732
## 22               23.55997             12.844674        2.397547
1.645732
##    Hz_max_5max Hz_mean_5max Events_10max Duration.ms._10max
scalar_max_10max
## 1     764.4826     666.4471          288             36.875
27.374
## 2     764.4826     666.4471          288             36.875
27.374
## 3     756.1414     427.6775          315             50.000
```

```
27.398
## 4     756.1414     427.6775           315               50.000
27.398
## 5     536.2686     345.3468           238               63.750
27.374
## 6     536.2686     345.3468           238               63.750
27.374
## 7     768.9069     643.3929           261               51.250
27.398
## 8     768.9069     643.3929           261               51.250
27.398
## 9     620.6669     430.3764           219               51.250
27.398
## 10    620.6669     430.3764           219               51.250
27.398
## 11    778.5273     613.7872           127               35.625
27.374
## 12    778.5273     613.7872           127               35.625
27.374
## 13    770.2075     768.9732           336               34.375
27.398
## 14    770.2075     768.9732           336               34.375
27.398
## 15    689.2542     689.2542           143               23.750
27.280
## 16    689.2542     689.2542           143               23.750
27.280
## 17    797.4869     778.3588           956               41.875
27.374
## 18    797.4869     778.3588           956               41.875
27.374
## 19    737.3024     671.6477           109               37.500
27.374
## 20    737.3024     671.6477           109               37.500
27.374
## 21    610.5303     419.0822           147               28.750
27.374
## 22    610.5303     419.0822           147               28.750
27.374
##    scalar_mean_10max changescalar_max_10max changescalar_mean_10max
## 1           21.58000                 23.724                  12.473
## 2           21.58000                 23.724                  12.473
## 3           20.37500                 24.477                  16.488
## 4           20.37500                 24.477                  16.488
## 5           19.68950                 16.627                   8.965
## 6           19.68950                 16.627                   8.965
## 7           22.66617                 21.153                  16.540
## 8           22.66617                 21.153                  16.540
## 9           21.55080                 21.177                  10.385
## 10          21.55080                 21.177                  10.385
```

```
## 11          20.67788                 23.031                   16.586
## 12          20.67788                 23.031                   16.586
## 13          27.37400                 25.203                   25.203
## 14          27.37400                 25.203                   25.203
## 15          24.43700                 23.570                   23.570
## 16          24.43700                 23.570                   23.570
## 17          17.44762                 15.583                   11.337
## 18          17.44762                 15.583                   11.337
## 19          27.37400                 21.912                   21.912
## 20          27.37400                 21.912                   21.912
## 21          20.66608                 15.583                   11.337
## 22          20.66608                 15.583                   11.337
##    Changevector_max_10max Changevector_mean_10max Angle_max_10max
## 1                27.69156                15.45774        2.687392
## 2                27.69156                15.45774        2.687392
## 3                35.06987                22.58486        2.969360
## 4                35.06987                22.58486        2.969360
## 5                33.00443                10.65686        2.105922
## 6                33.00443                10.65686        2.105922
## 7                43.01068                19.71266        3.019490
## 8                43.01068                19.71266        3.019490
## 9                38.58406                13.32574        2.437353
## 10               38.58406                13.32574        2.437353
## 11               26.68027                19.98326        3.057269
## 12               26.68027                19.98326        3.057269
## 13               38.53399                26.95529        3.019751
## 14               38.53399                26.95529        3.019751
## 15               41.64894                25.28215        2.569745
## 16               41.64894                25.28215        2.569745
## 17               23.55997                12.84467        2.397547
## 18               23.55997                12.84467        2.397547
## 19               32.78848                26.58408        2.271090
## 20               32.78848                26.58408        2.271090
## 21               23.55997                12.86923        1.733631
## 22               23.55997                12.86923        1.733631
##    Angle_mean_10max Hz_max_10max Hz_mean_10max Events_15max
Duration.ms._15max
## 1         1.0470779     684.3388      266.6362          144
30.625
## 2         1.0470779     684.3388      266.6362          144
30.625
## 3         1.6138687     756.1414      410.9683          183
29.375
## 4         1.6138687     756.1414      410.9683          183
29.375
## 5         0.5851573     536.2686      149.0091          111
41.250
## 6         0.5851573     536.2686      149.0091          111
41.250
## 7         2.0094655     768.9069      511.7062          163
```

```
39.375
## 8         2.0094655     768.9069      511.7062           163
39.375
## 9         0.5720825     620.6669      145.6796           129
18.750
## 10        0.5720825     620.6669      145.6796           129
18.750
## 11        2.7905896     778.5273      710.6178            54
20.000
## 12        2.7905896     778.5273      710.6178            54
20.000
## 13        3.0197506     768.9732      768.9732           155
30.625
## 14        3.0197506     768.9732      768.9732           155
30.625
## 15        2.5697446     654.3801      654.3801            74
20.000
## 16        2.5697446     654.3801      654.3801            74
20.000
## 17        1.6457318     610.5303      419.0822           627
21.875
## 18        1.6457318     610.5303      419.0822           627
21.875
## 19        1.3260388     578.3283      337.6730            55
22.500
## 20        1.3260388     578.3283      337.6730            55
22.500
## 21        1.7336315     441.4656      441.4656            59
20.625
## 22        1.7336315     441.4656      441.4656            59
20.625
##    scalar_max_15max scalar_mean_15max changescalar_max_15max
## 1            27.374          22.42986                 23.724
## 2            27.374          22.42986                 23.724
## 3            27.398          21.71558                 24.477
## 4            27.398          21.71558                 24.477
## 5            27.374          22.77170                 16.627
## 6            27.374          22.77170                 16.627
## 7            27.398          25.17089                 21.153
## 8            27.398          25.17089                 21.153
## 9            27.398          26.26925                 21.177
## 10           27.398          26.26925                 21.177
## 11           27.374          23.62200                 23.031
## 12           27.374          23.62200                 23.031
## 13           27.398          27.37400                 25.203
## 14           27.398          27.37400                 25.203
## 15           27.280          26.83000                 23.570
## 16           27.280          26.83000                 23.570
## 17           27.374          27.13300                 26.650
## 18           27.374          27.13300                 26.650
```

```
## 19          27.374         27.37400                    21.912
## 20          27.374         27.37400                    21.912
## 21          27.374         22.01064                    15.583
## 22          27.374         22.01064                    15.583
##    changescalar_mean_15max Changevector_max_15max Changevector_mean_15max
## 1                 15.66700               27.69156                19.75723
## 2                 15.66700               27.69156                19.75723
## 3                 16.48800               35.06987                21.61531
## 4                 16.48800               35.06987                21.61531
## 5                 11.47400               33.00443                12.83840
## 6                 11.47400               33.00443                12.83840
## 7                 16.54000               43.01068                19.68153
## 8                 16.54000               43.01068                19.68153
## 9                 10.69533               38.58406                13.08278
## 10                10.69533               38.58406                13.08278
## 11                22.06100               26.68027                25.02888
## 12                22.06100               26.68027                25.02888
## 13                25.20300               38.53399                26.95529
## 14                25.20300               38.53399                26.95529
## 15                23.57000               41.64894                28.52343
## 16                23.57000               41.64894                28.52343
## 17                26.51300               44.88917                30.34401
## 18                26.51300               44.88917                30.34401
## 19                21.91200               26.58408                26.58408
## 20                21.91200               26.58408                26.58408
## 21                 9.94300               23.55997                13.04511
## 22                 9.94300               23.55997                13.04511
##    Angle_max_15max Angle_mean_15max Hz_max_15max Hz_mean_15max
Events_20max
## 1         2.687392        1.8427590     684.3388      469.2547
57
## 2         2.687392        1.8427590     684.3388      469.2547
57
## 3         2.969360        1.6010884     756.1414      407.7138
75
## 4         2.969360        1.6010884     756.1414      407.7138
75
## 5         2.105922        0.6709018     536.2686      170.8438
42
## 6         2.105922        0.6709018     536.2686      170.8438
42
## 7         2.858084        2.0094655     727.8052      511.7062
97
## 8         2.858084        2.0094655     727.8052      511.7062
97
## 9         2.437353        0.7045289     620.6669      179.4068
71
## 10        2.437353        0.7045289     620.6669      179.4068
71
## 11        2.790590        2.7905896     710.6178      710.6178
```

```
26
## 12        2.790590        2.7905896     710.6178      710.6178
26
## 13        3.019751        3.0197506     768.9732      768.9732
67
## 14        3.019751        3.0197506     768.9732      768.9732
67
## 15        2.569745        2.5697446     654.3801      654.3801
40
## 16        2.569745        2.5697446     654.3801      654.3801
40
## 17        3.124861        3.1248615     795.7394      795.7394
215
## 18        3.124861        3.1248615     795.7394      795.7394
215
## 19        1.326039        1.3260388     337.6730      337.6730
24
## 20        1.326039        1.3260388     337.6730      337.6730
24
## 21        1.434879        0.6201624     365.3889      157.9230
23
## 22        1.434879        0.6201624     365.3889      157.9230
23
##    Duration.ms._20max scalar_max_20max scalar_mean_20max
changescalar_max_20max
## 1              17.500           27.374          26.80175
23.724
## 2              17.500           27.374          26.80175
23.724
## 3              20.625           27.398          26.38567
24.477
## 4              20.625           27.398          26.38567
24.477
## 5              20.625           27.374          25.17900
13.480
## 6              20.625           27.374          25.17900
13.480
## 7              17.500           27.398          27.37400
21.153
## 8              17.500           27.398          27.37400
21.153
## 9              14.375           27.398          26.99500
22.590
## 10             14.375           27.398          26.99500
22.590
## 11             16.875           27.374          25.69840
23.031
## 12             16.875           27.374          25.69840
23.031
## 13             28.125           27.398          27.37400
```

```
25.203
## 14              28.125           27.398          27.37400
25.203
## 15              13.125           27.280          26.83000
23.570
## 16              13.125           27.280          26.83000
23.570
## 17              15.625           27.374          27.37400
26.650
## 18              15.625           27.374          27.37400
26.650
## 19              10.625           27.374          27.37400
21.912
## 20              10.625           27.374          27.37400
21.912
## 21              10.625           27.374          24.71600
18.770
## 22              10.625           27.374          24.71600
18.770
##    changescalar_mean_20max Changevector_max_20max Changevector_mean_20max
## 1                  23.7240               27.69156                27.69156
## 2                  23.7240               27.69156                27.69156
## 3                  13.7095               35.06987                29.65156
## 4                  13.7095               35.06987                29.65156
## 5                   6.6090               33.00443                11.19072
## 6                   6.6090               33.00443                11.19072
## 7                  21.1530               37.65988                27.82295
## 8                  21.1530               37.65988                27.82295
## 9                  22.5900               38.58406                28.28571
## 10                 22.5900               38.58406                28.28571
## 11                 22.0610               28.39364                25.02888
## 12                 22.0610               28.39364                25.02888
## 13                 25.2030               38.53399                27.67895
## 14                 25.2030               38.53399                27.67895
## 15                 23.5700               32.39028                26.22228
## 16                 23.5700               32.39028                26.22228
## 17                 26.5130               44.88917                34.48002
## 18                 26.5130               44.88917                34.48002
## 19                 21.9120               26.58408                26.58408
## 20                 21.9120               26.58408                26.58408
## 21                  5.4005               23.55997                23.55997
## 22                  5.4005               23.55997                23.55997
##    Angle_max_20max Angle_mean_20max Hz_max_20max Hz_mean_20max
Events_5mean
## 1         2.687392         2.687392   684.338806    684.338806
909
## 2         2.687392         2.687392   684.338806    684.338806
909
## 3         2.433775         1.792941   619.755737    456.568756
708
```

```
## 4         2.433775        1.792941   619.755737    456.568756
708
## 5         1.715913        0.489164   436.953766    124.564590
1187
## 6         1.715913        0.489164   436.953766    124.564590
1187
## 7         2.456815        2.346721   625.622742    597.587647
625
## 8         2.456815        2.346721   625.622742    597.587647
625
## 9         2.473098        2.231482   629.769348    568.242188
606
## 10        2.473098        2.231482   629.769348    568.242188
606
## 11        3.057269        2.671452     3.057269      2.671452
344
## 12        3.057269        2.671452     3.057269      2.671452
344
## 13        2.837015        2.837015   722.439880    722.439880
1197
## 14        2.837015        2.837015   722.439880    722.439880
1197
## 15        2.121830        1.637631   540.319580    417.019348
458
## 16        2.121830        1.637631   540.319580    417.019348
458
## 17        3.062443        2.921857   779.844849    744.044861
1228
## 18        3.062443        2.921857   779.844849    744.044861
1228
## 19        1.326039        1.326039   337.673035    337.673035
341
## 20        1.326039        1.326039   337.673035    337.673035
341
## 21        1.751492        1.187941   446.013886    302.506744
797
## 22        1.751492        1.187941   446.013886    302.506744
797
##    Duration.ms._5mean scalar_max_5mean scalar_mean_5mean
changescalar_max_5mean
## 1            5.215209         9.516648          7.473747
3.755884
## 2            5.215209         9.516648          7.473747
3.755884
## 3           11.785840        10.551797          7.565272
2.816095
## 4           11.785840        10.551797          7.565272
2.816095
## 5            7.348884         8.022198          6.506753
1.632164
```

```
## 6           7.348884         8.022198          6.506753
1.632164
## 7          11.660000        10.866869          7.661629
3.259443
## 8          11.660000        10.866869          7.661629
3.259443
## 9          10.192863         9.977073          7.407830
2.386498
## 10         10.192863         9.977073          7.407830
2.386498
## 11         10.268895        10.081480          7.639484
2.714073
## 12         10.268895        10.081480          7.639484
2.714073
## 13          5.269946         9.081577          7.721689
5.278800
## 14          5.269946         9.081577          7.721689
5.278800
## 15          8.597162         9.467170          7.418493
2.849013
## 16          8.597162         9.467170          7.418493
2.849013
## 17          8.547435        12.092415          8.187675
8.695595
## 18          8.547435        12.092415          8.187675
8.695595
## 19          9.536290         9.647760          7.688582
2.322557
## 20          9.536290         9.647760          7.688582
2.322557
## 21          6.762861         7.866112          6.543674
1.442472
## 22          6.762861         7.866112          6.543674
1.442472
##    changescalar_mean_5mean Changevector_max_5mean Changevector_mean_5mean
## 1                1.6630629               4.501180               2.2195642
## 2                1.6630629               4.501180               2.2195642
## 3                1.0076953               3.691769               1.6388020
## 4                1.0076953               3.691769               1.6388020
## 5                0.6804169               2.293485               1.1945393
## 6                0.6804169               2.293485               1.1945393
## 7                1.1437375               4.719048               1.8831480
## 8                1.1437375               4.719048               1.8831480
## 9                0.8333807               3.104335               1.2454215
## 10               0.8333807               3.104335               1.2454215
## 11               1.1398769               3.622518               1.5856004
## 12               1.1398769               3.622518               1.5856004
## 13               4.1304387               6.531255               5.1731570
## 14               4.1304387               6.531255               5.1731570
## 15               1.5004447               3.800236               2.0941445
```

```
## 16              1.5004447                3.800236                    2.0941445
## 17              4.5129455               12.182116                    6.4512431
## 18              4.5129455               12.182116                    6.4512431
## 19              1.2198887                2.883656                    1.5596537
## 20              1.2198887                2.883656                    1.5596537
## 21              0.6226729                1.766703                    0.8315615
## 22              0.6226729                1.766703                    0.8315615
##    Angle_max_5mean Angle_mean_5mean Hz_max_5mean Hz_mean_5mean
Events._10mean
## 1        0.4296757       0.17557082    109.41603      44.70874
288
## 2        0.4296757       0.17557082    109.41603      44.70874
288
## 3        0.3715939       0.14143267     94.62562      36.01553
315
## 4        0.3715939       0.14143267     94.62562      36.01553
315
## 5        0.2539263       0.13104989     64.66179      33.37158
238
## 6        0.2539263       0.13104989     64.66179      33.37158
238
## 7        0.4289521       0.16791337    109.23175      42.75879
261
## 8        0.4289521       0.16791337    109.23175      42.75879
261
## 9        0.2459020       0.10166733     62.61844      25.88937
219
## 10       0.2459020       0.10166733     62.61844      25.88937
219
## 11       0.3642217       0.14406242     92.74830      36.68519
127
## 12       0.3642217       0.14406242     92.74830      36.68519
127
## 13       0.9873386       0.84052125    251.42371     214.03698
336
## 14       0.9873386       0.84052125    251.42371     214.03698
336
## 15       0.4058039       0.23266164    103.33711      59.24680
143
## 16       0.4058039       0.23266164    103.33711      59.24680
143
## 17       1.7833433       0.94024345    454.12465     239.43103
956
## 18       1.7833433       0.94024345    454.12465     239.43103
956
## 19       0.3068617       0.17720143     78.14168      45.12397
109
## 20       0.3068617       0.17720143     78.14168      45.12397
109
## 21       0.1658718       0.07985005     42.23891      20.33365
```

```
147
## 22       0.1658718       0.07985005     42.23891      20.33365
147
##    Duration.ms._10mean scalar_max_10mean scalar_mean_10mean
## 1             3.888889          15.49537           13.49979
## 2             3.888889          15.49537           13.49979
## 3             7.706349          16.38244           13.65682
## 4             7.706349          16.38244           13.65682
## 5             7.415966          15.56483           13.15894
## 6             7.415966          15.56483           13.15894
## 7             8.694923          17.47214           14.09932
## 8             8.694923          17.47214           14.09932
## 9             7.046233          16.84753           13.95908
## 10            7.046233          16.84753           13.95908
## 11            7.711614          15.61902           13.34467
## 12            7.711614          15.61902           13.34467
## 13            4.603795          15.58534           14.11735
## 14            4.603795          15.58534           14.11735
## 15            6.551573          16.43141           14.10137
## 16            6.551573          16.43141           14.10137
## 17            3.531642          16.65479           14.78717
## 18            3.531642          16.65479           14.78717
## 19            8.314220          15.75183           13.57757
## 20            8.314220          15.75183           13.57757
## 21            6.568878          14.94327           12.92497
## 22            6.568878          14.94327           12.92497
##    changescalar_max_10mean changescalar_mean_10mean
Changevector_max_10mean
## 1                 6.557340                 3.249119
8.029862
## 2                 6.557340                 3.249119
8.029862
## 3                 4.928854                 1.971893
6.505797
## 4                 4.928854                 1.971893
6.505797
## 5                 4.004025                 1.664277
5.891075
## 6                 4.004025                 1.664277
5.891075
## 7                 5.731820                 2.287741
8.824568
## 8                 5.731820                 2.287741
8.824568
## 9                 4.827333                 1.882122
6.463404
## 10                4.827333                 1.882122
6.463404
## 11                4.678425                 2.304800
6.327268
```

```
## 12               4.678425                 2.304800
6.327268
## 13               9.671390                 7.831686
11.687588
## 14               9.671390                 7.831686
11.687588
## 15               6.199000                 3.950780
8.375599
## 16               6.199000                 3.950780
8.375599
## 17              12.084997                 9.085673
16.708552
## 18              12.084997                 9.085673
16.708552
## 19               3.833505                 1.941137
4.873919
## 20               3.833505                 1.941137
4.873919
## 21               3.380361                 1.478924
4.343664
## 22               3.380361                 1.478924
4.343664
##    Changevector_mean_10mean Angle_max_10mean Angle_mean_10mean
Hz_max_10mean
## 1                  4.538349        0.4719538         0.2224823
120.18206
## 2                  4.538349        0.4719538         0.2224823
120.18206
## 3                  3.339135        0.4047630         0.1751144
103.07205
## 4                  3.339135        0.4047630         0.1751144
103.07205
## 5                  2.909783        0.3492638         0.1583697
88.93930
## 6                  2.909783        0.3492638         0.1583697
88.93930
## 7                  4.023194        0.5316167         0.2214615
135.37507
## 8                  4.023194        0.5316167         0.2214615
135.37507
## 9                  2.961754        0.3207744         0.1358000
81.68452
## 10                 2.961754        0.3207744         0.1358000
81.68452
## 11                 3.297187        0.4503593         0.2418717
114.68307
## 12                 3.297187        0.4503593         0.2418717
114.68307
## 13                 9.298636        1.0612962         0.8879423
270.25685
```

```
## 14                9.298636       1.0612962         0.8879423
270.25685
## 15                5.578557       0.5703053         0.3757078
145.22704
## 16                5.578557       0.5703053         0.3757078
145.22704
## 17               13.023632       1.8310150         1.3784785
466.26415
## 18               13.023632       1.8310150         1.3784785
466.26415
## 19                2.547766       0.2685230         0.1255957
68.37882
## 20                2.547766       0.2685230         0.1255957
68.37882
## 21                2.169658       0.2465197         0.1225393
62.77572
## 22                2.169658       0.2465197         0.1225393
62.77572
##    Hz_mean_10mean Events_15mean Duration.ms._15mean scalar_max_15mean
## 1        56.65466           144            3.441840          19.26462
## 2        56.65466           144            3.441840          19.26462
## 3        44.59251           183            6.342213          19.93954
## 4        44.59251           183            6.342213          19.93954
## 5        40.32851           111            6.920045          19.71768
## 6        40.32851           111            6.920045          19.71768
## 7        56.39470           163            6.679448          21.17141
## 8        56.39470           163            6.679448          21.17141
## 9        34.58118           129            5.692829          20.71912
## 10       34.58118           129            5.692829          20.71912
## 11       61.59211            54            6.215278          20.16783
## 12       61.59211            54            6.215278          20.16783
## 13      226.11266           155            4.088710          19.78399
## 14      226.11266           155            4.088710          19.78399
## 15       95.67319            74            5.675676          20.57669
## 16       95.67319            74            5.675676          20.57669
## 17      351.02666           627            2.309609          19.16135
## 18      351.02666           627            2.309609          19.16135
## 19       31.98268            55            6.897727          19.54340
## 20       31.98268            55            6.897727          19.54340
## 21       31.20437            59            6.430085          19.64119
## 22       31.20437            59            6.430085          19.64119
##    scalar_mean_15mean changescalar_max_15mean changescalar_mean_15mean
## 1            17.94158                8.195410                 4.209463
## 2            17.94158                8.195410                 4.209463
## 3            17.79343                6.271208                 2.424063
## 4            17.79343                6.271208                 2.424063
## 5            17.60073                5.291784                 2.055466
## 6            17.60073                5.291784                 2.055466
## 7            18.56933                7.117798                 2.959305
## 8            18.56933                7.117798                 2.959305
```

```
## 9            18.50218               6.094922                 2.259104
## 10           18.50218               6.094922                 2.259104
## 11           18.33345               6.392648                 3.365325
## 12           18.33345               6.392648                 3.365325
## 13           18.54576              12.053490                 9.476997
## 14           18.54576              12.053490                 9.476997
## 15           18.70649               8.263824                 5.544206
## 16           18.70649               8.263824                 5.544206
## 17           18.27424              14.037563                11.493413
## 18           18.27424              14.037563                11.493413
## 19           17.98809               4.894255                 2.913892
## 20           17.98809               4.894255                 2.913892
## 21           17.77297               4.621576                 1.704082
## 22           17.77297               4.621576                 1.704082
##    Changevector_max_15mean Changevector_mean_15mean Angle_max_15mean
## 1                10.145769                 6.194357        0.4732660
## 2                10.145769                 6.194357        0.4732660
## 3                 8.773143                 4.485467        0.4584332
## 4                 8.773143                 4.485467        0.4584332
## 5                 8.644509                 4.178605        0.4437400
## 6                 8.644509                 4.178605        0.4437400
## 7                11.561665                 5.681387        0.5983693
## 8                11.561665                 5.681387        0.5983693
## 9                 8.764987                 4.040512        0.3766614
## 10                8.764987                 4.040512        0.3766614
## 11                9.016411                 4.903490        0.5774477
## 12                9.016411                 4.903490        0.5774477
## 13               14.789442                11.603298        1.0369326
## 14               14.789442                11.603298        1.0369326
## 15               11.090594                 7.834694        0.6387165
## 16               11.090594                 7.834694        0.6387165
## 17               19.028860                16.285452        1.7973103
## 18               19.028860                16.285452        1.7973103
## 19                6.223851                 3.892721        0.2826364
## 20                6.223851                 3.892721        0.2826364
## 21                6.278427                 2.939477        0.2771535
## 22                6.278427                 2.939477        0.2771535
##    Angle_mean_15mean Hz_max_15mean Hz_mean_15mean Events_20mean
## 1          0.2644405     120.51619       67.33922            57
## 2          0.2644405     120.51619       67.33922            57
## 3          0.2042285     116.73905       52.00635            75
## 4          0.2042285     116.73905       52.00635            75
## 5          0.1960600     112.99747       49.92626            42
## 6          0.1960600     112.99747       49.92626            42
## 7          0.2760395     152.37349       70.29288            97
## 8          0.2760395     152.37349       70.29288            97
## 9          0.1655346      95.91604       42.15305            71
## 10         0.1655346      95.91604       42.15305            71
## 11         0.3209953     147.04584       81.74078            26
## 12         0.3209953     147.04584       81.74078            26
```

```
## 13         0.8448429     264.05270      215.13746             67
## 14         0.8448429     264.05270      215.13746             67
## 15         0.4394608     162.64783      111.90777             40
## 16         0.4394608     162.64783      111.90777             40
## 17         1.4886767     457.68131      379.08842            215
## 18         1.4886767     457.68131      379.08842            215
## 19         0.1733759      71.97276       44.14980             24
## 20         0.1733759      71.97276       44.14980             24
## 21         0.1236359      70.57657       31.48361             23
## 22         0.1236359      70.57657       31.48361             23
##    Duration.ms._20mean scalar_max_20mean scalar_mean_20mean
## 1             2.587719          22.76440           22.04857
## 2             2.587719          22.76440           22.04857
## 3             5.250000          23.71752           22.29175
## 4             5.250000          23.71752           22.29175
## 5             5.997024          24.28693           22.58697
## 6             5.997024          24.28693           22.58697
## 7             4.780928          24.02707           22.55178
## 8             4.780928          24.02707           22.55178
## 9             4.110915          24.17137           22.85893
## 10            4.110915          24.17137           22.85893
## 11            5.697115          23.89650           22.45107
## 12            5.697115          23.89650           22.45107
## 13            3.955224          23.07013           22.22635
## 14            3.955224          23.07013           22.22635
## 15            4.343750          23.26423           22.24845
## 16            4.343750          23.26423           22.24845
## 17            1.662791          22.49881           22.17691
## 18            1.662791          22.49881           22.17691
## 19            4.088542          22.63937           21.81412
## 20            4.088542          22.63937           21.81412
## 21            4.429348          23.44813           22.17297
## 22            4.429348          23.44813           22.17297
##    changescalar_max_20mean changescalar_mean_20mean
Changevector_max_20mean
## 1                 7.502070                 3.960703
11.351230
## 2                 7.502070                 3.960703
11.351230
## 3                 6.190293                 2.676377
11.368331
## 4                 6.190293                 2.676377
11.368331
## 5                 5.996190                 2.445063
11.972913
## 6                 5.996190                 2.445063
11.972913
## 7                 7.212021                 3.252846
12.895357
## 8                 7.212021                 3.252846
```

```
12.895357
## 9                   8.011803                  3.869286
12.641429
## 10                  8.011803                  3.869286
12.641429
## 11                  5.376538                  3.092617
8.757743
## 12                  5.376538                  3.092617
8.757743
## 13                 13.001493                  9.904319
17.353135
## 14                 13.001493                  9.904319
17.353135
## 15                  8.159425                  6.056935
11.347463
## 16                  8.159425                  6.056935
11.347463
## 17                 14.139033                 12.105276
19.387201
## 18                 14.139033                 12.105276
19.387201
## 19                  5.236417                  4.189330
7.482498
## 20                  5.236417                  4.189330
7.482498
## 21                  5.234087                  2.159112
9.517031
## 22                  5.234087                  2.159112
9.517031
##    Changevector_mean_20mean Angle_max_20mean Angle_mean_20mean
Hz_max_20mean
## 1                   6.922532        0.5201012         0.3140733
132.4426733
## 2                   6.922532        0.5201012         0.3140733
132.4426733
## 3                   5.877764        0.5214004         0.2548023
132.7735352
## 4                   5.877764        0.5214004         0.2548023
132.7735352
## 5                   5.476046        0.5391966         0.2201107
137.3052959
## 6                   5.476046        0.5391966         0.2201107
137.3052959
## 7                   6.842624        0.5845279         0.2968501
148.8488099
## 8                   6.842624        0.5845279         0.2968501
148.8488099
## 9                   6.915520        0.5616227         0.3022871
143.0160498
## 10                  6.915520        0.5616227         0.3022871
```

```
143.0160498
## 11              4.827669        0.4931102         0.3048133
0.5015529
## 12              4.827669        0.4931102         0.3048133
0.5015529
## 13             13.021549        0.9347129         0.7174151
238.0226803
## 14             13.021549        0.9347129         0.7174151
238.0226803
## 15              8.723417        0.5050627         0.3870018
128.6131706
## 16              8.723417        0.5050627         0.3870018
128.6131706
## 17             17.154057        1.2518285         1.0993131
318.7755134
## 18             17.154057        1.2518285         1.0993131
318.7755134
## 19              5.678423        0.3095699         0.2245841
78.8313326
## 20              5.678423        0.3095699         0.2245841
78.8313326
## 21              5.071838        0.4296136         0.2177648
109.4002023
## 22              5.071838        0.4296136         0.2177648
109.4002023
##    Hz_mean_20mean RH._Max Temp_Max RH._mean Temp_mean
## 1       79.978117   47.08    23.50    41.69     19.73
## 2       79.978117   60.14    27.50    41.69     19.73
## 3       64.884868   62.61    29.75    53.55     21.79
## 4       64.884868   68.10    32.00    53.55     21.79
## 5       56.050730   57.37    28.69    53.25     23.29
## 6       56.050730   74.59    19.25    53.25     23.29
## 7       75.592258   73.52    24.31    50.02     24.58
## 8       75.592258   78.07    27.13    50.02     24.58
## 9       76.976767   84.59    30.13    47.82     22.38
## 10      76.976767   78.42    27.38    47.82     22.38
## 11       0.307912   81.07    25.19    72.32     10.22
## 12       0.307912   47.08    23.50    72.32     10.22
## 13     182.688259   60.14    27.50    71.53     11.59
## 14     182.688259   62.61    29.75    71.53     11.59
## 15      98.549185   68.10    32.00    72.87     11.94
## 16      98.549185   57.37    28.69    72.87     11.94
## 17     279.937784   74.59    19.25    71.95     15.36
## 18     279.937784   73.52    24.31    71.95     15.36
## 19      57.189869   78.07    27.13    76.17     15.11
## 20      57.189869   84.59    30.13    76.17     15.11
## 21      55.453343   78.42    27.38    77.90     14.61
## 22      55.453343   81.07    25.19    77.90     14.61
```

```
#####SCATTER PLOTS
plot(tab$MSV~tab$RH._Max)#*
```

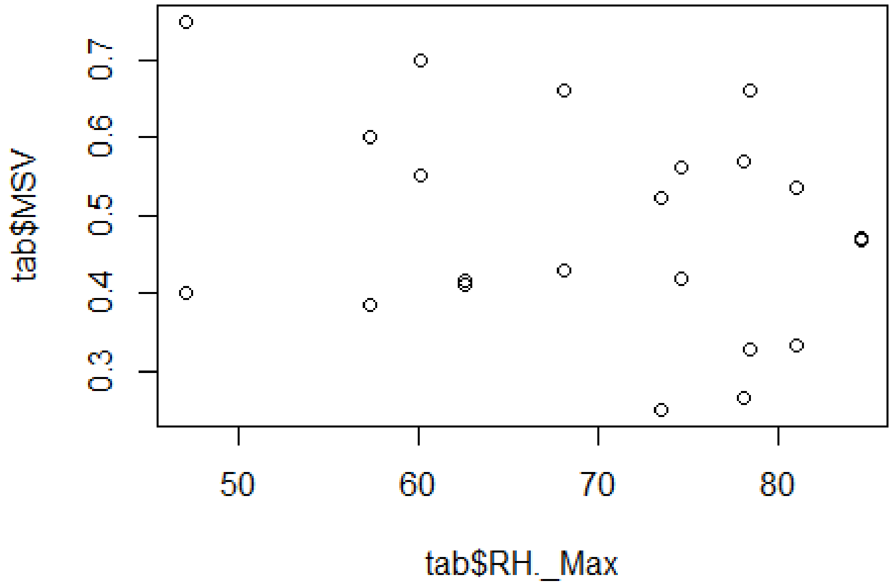


```
plot(tab$MSV~tab$RH._mean)#*
```

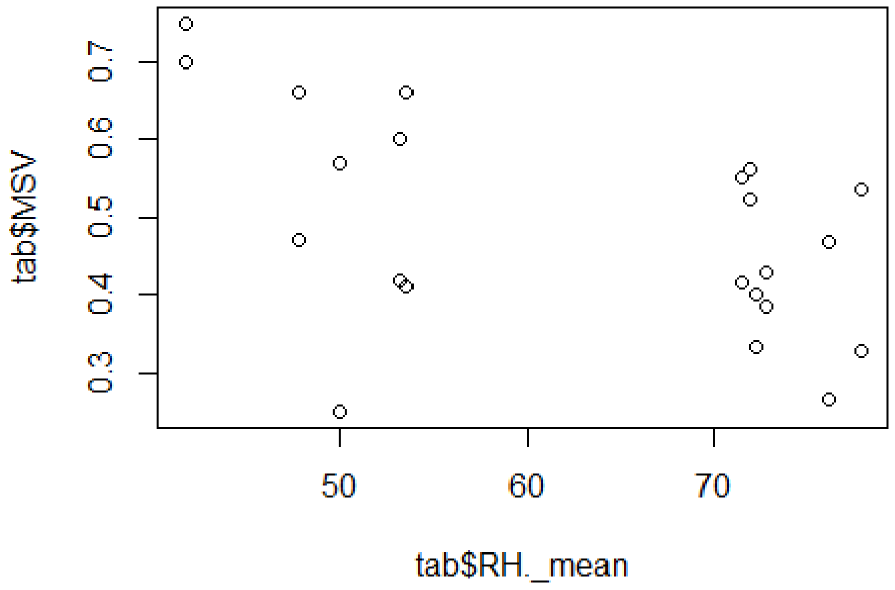


```
plot(tab$MSV~tab$Temp_mean)#*
```

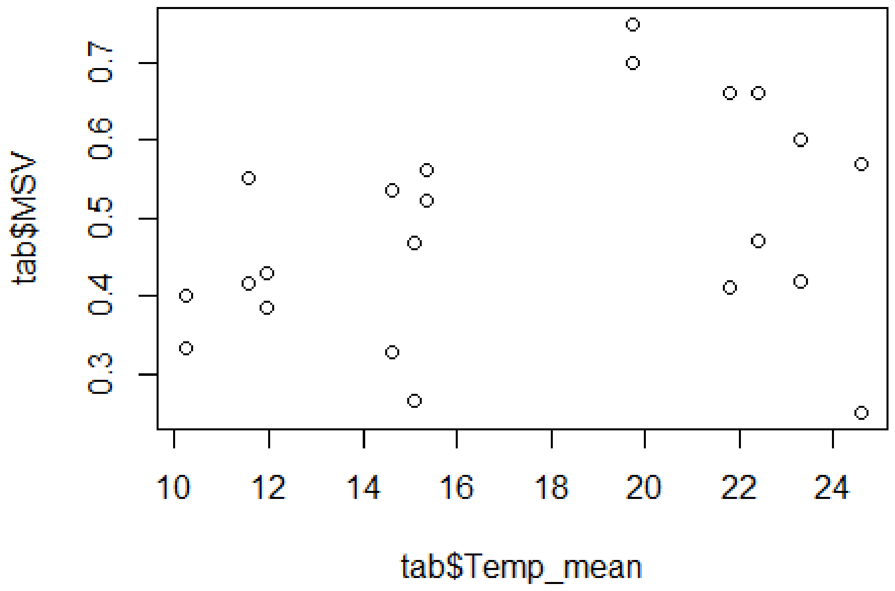


```
plot(tab$MSV~tab$Temp_Max)
```

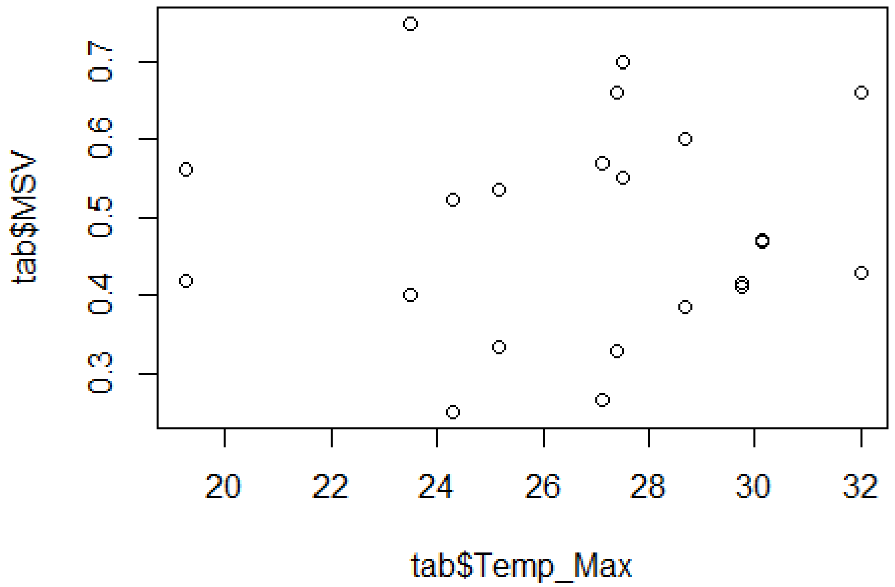


```
#Scatter plots at threshold 5-Max

par(mfrow = c(2,2))
plot(tab$MSV~tab$Events._5max)
plot(tab$MSV~tab$Duration.ms._5max)
plot(tab$MSV~tab$scalar_max_5max)
plot(tab$MSV~tab$scalar_mean_5max)
```

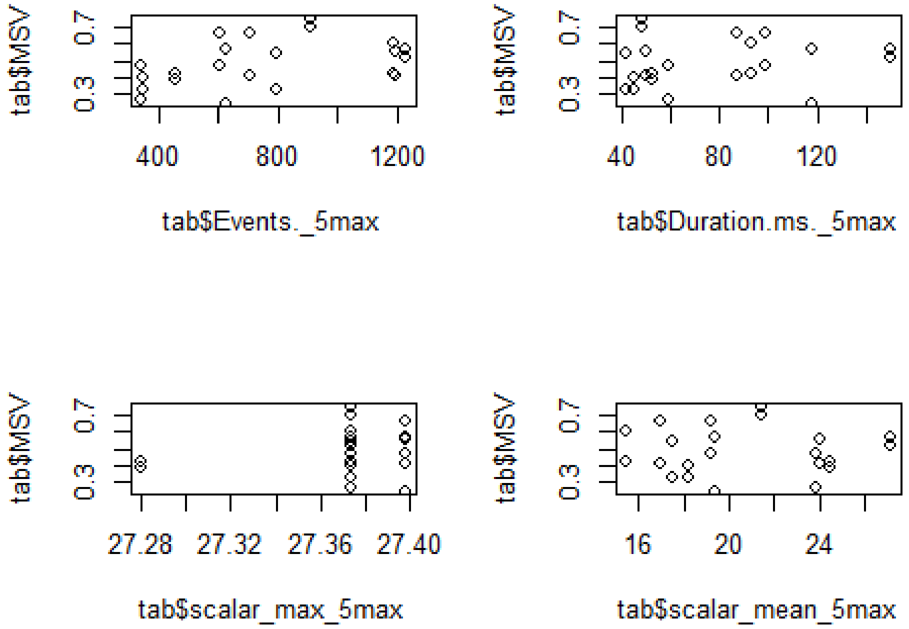


```
plot(tab$MSV~tab$changescalar_max_5max)
plot(tab$MSV~tab$changescalar_mean_5max)#*
plot(tab$MSV~tab$Changevector_max_5max)
plot(tab$MSV~tab$Changevector_mean_5max)#*
```

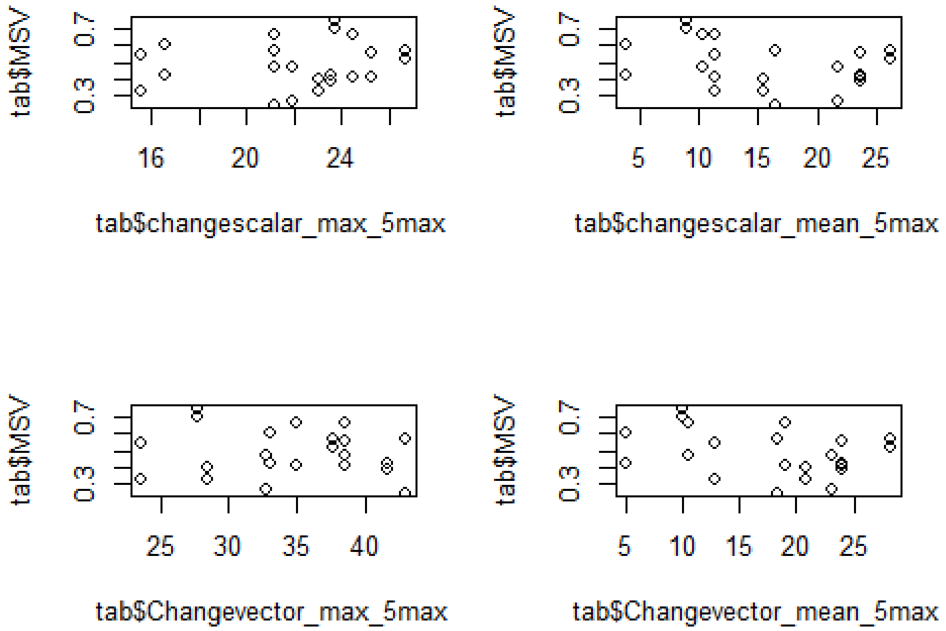


```
plot(tab$MSV~tab$Angle_max_5max)
plot(tab$MSV~tab$Angle_mean_5max)
plot(tab$MSV~tab$Hz_max_5max)
plot(tab$MSV~tab$Hz_mean_5max)
```

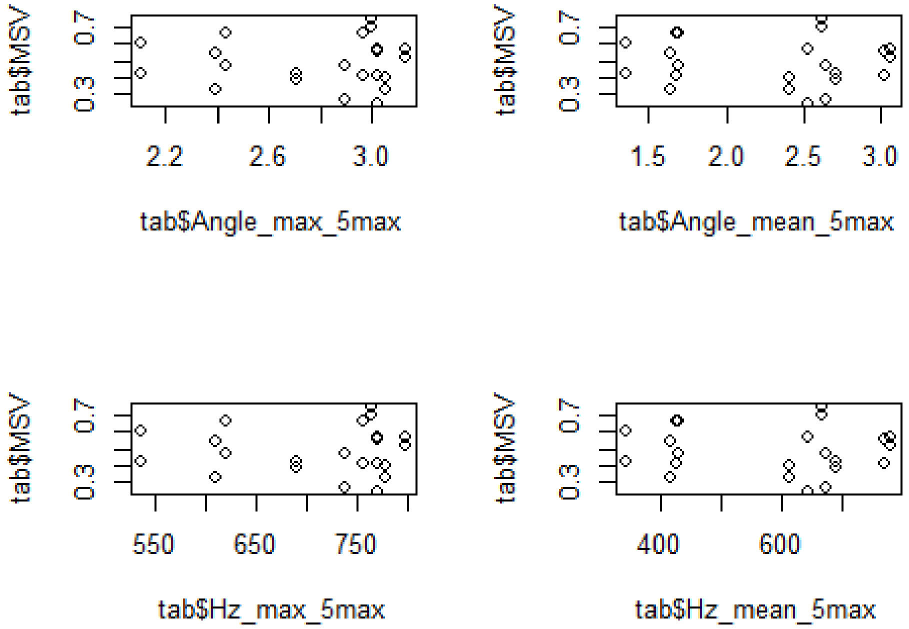

#Scatter plots at threshold 5-Mean

```
par(mfrow = c(2,2))
plot(tab$MSV~tab$Duration.ms._5mean)#*
plot(tab$MSV~tab$scalar_max_5mean)
plot(tab$MSV~tab$scalar_mean_5mean)
plot(tab$MSV~tab$changescalar_max_5mean)
```

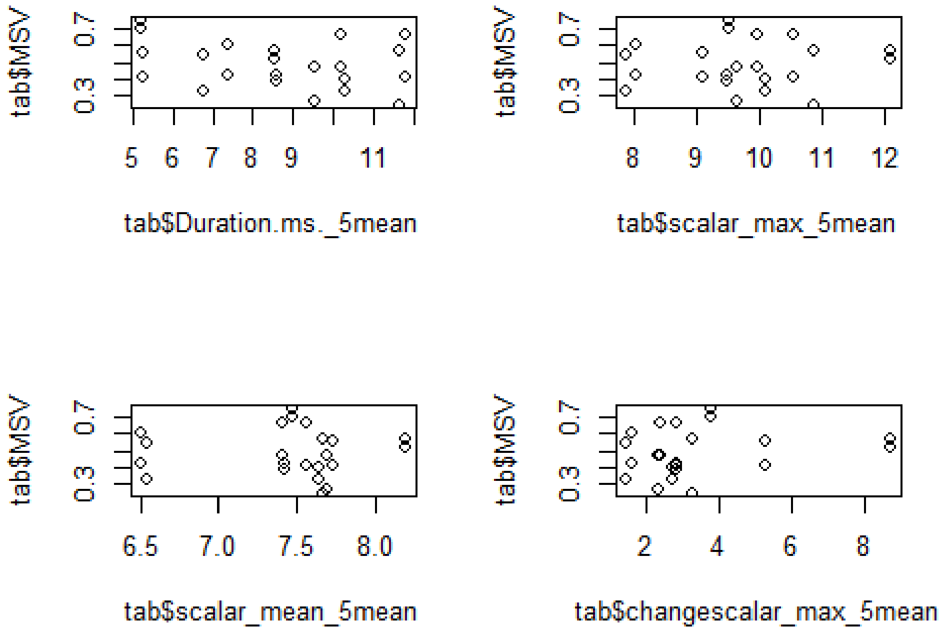


```
plot(tab$MSV~tab$changescalar_mean_5mean)
plot(tab$MSV~tab$Changevector_max_5mean)
plot(tab$MSV~tab$Changevector_mean_5mean)
plot(tab$MSV~tab$Angle_max_5mean)
```

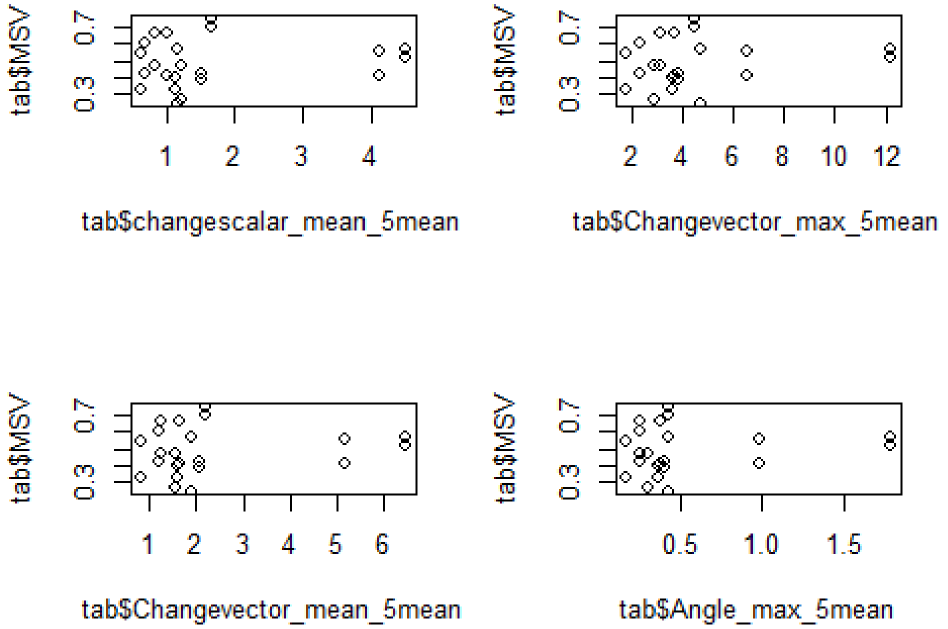


```
plot(tab$MSV~tab$Angle_mean_5mean)
plot(tab$MSV~tab$Hz_max_5mean)
plot(tab$MSV~tab$Hz_mean_5mean)
```

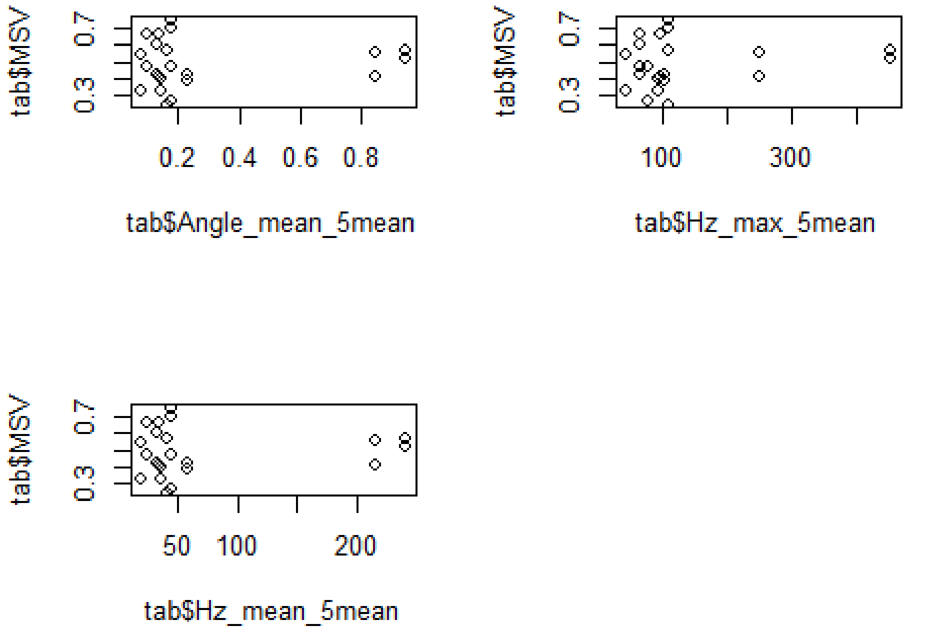

#Scatter plots at threshold 10-Max

```
par(mfrow = c(2,2))
plot(tab$MSV~tab$Events_10max)
plot(tab$MSV~tab$Duration.ms._10max)
plot(tab$MSV~tab$scalar_max_10max)
plot(tab$MSV~tab$scalar_mean_10max)#*
```

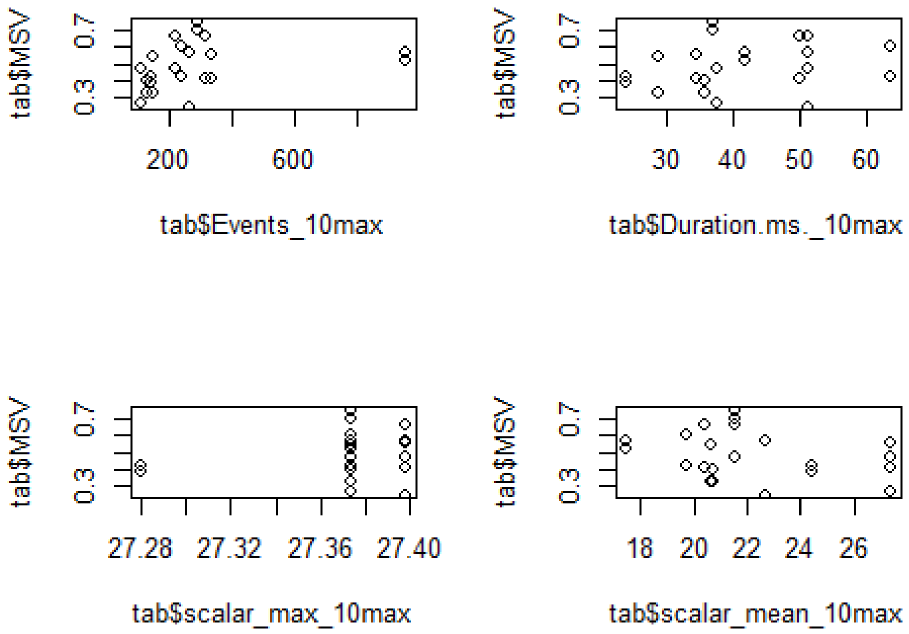


```
plot(tab$MSV~tab$changescalar_max_10max)
plot(tab$MSV~tab$changescalar_mean_10max)#*
plot(tab$MSV~tab$Changevector_max_10max)
plot(tab$MSV~tab$Changevector_mean_10max)#*
```

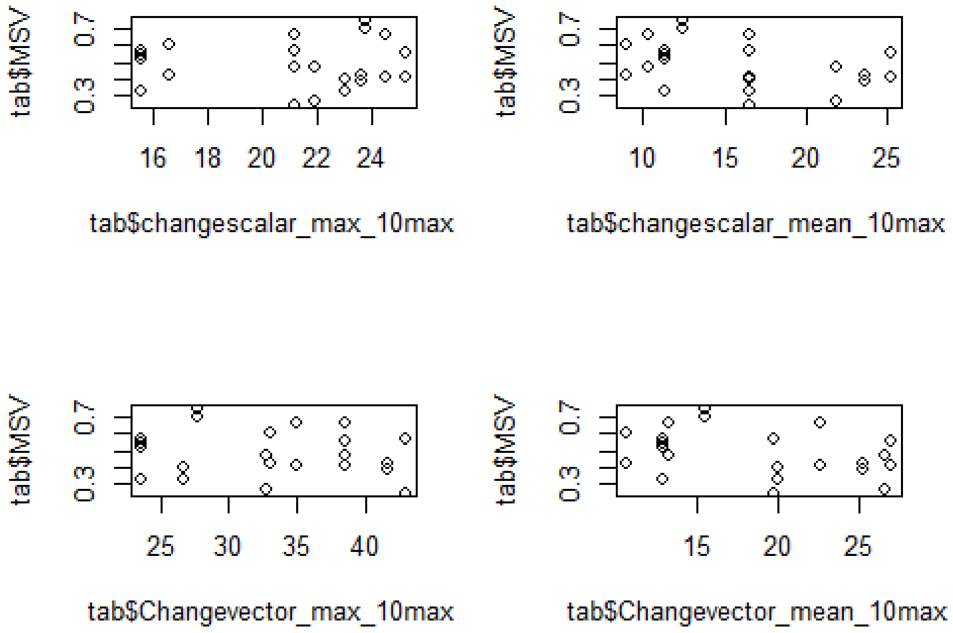


```
plot(tab$MSV~tab$Angle_max_10max)
plot(tab$MSV~tab$Angle_mean_10max)#*
plot(tab$MSV~tab$Hz_max_10max)
plot(tab$MSV~tab$Hz_mean_10max)#*
```

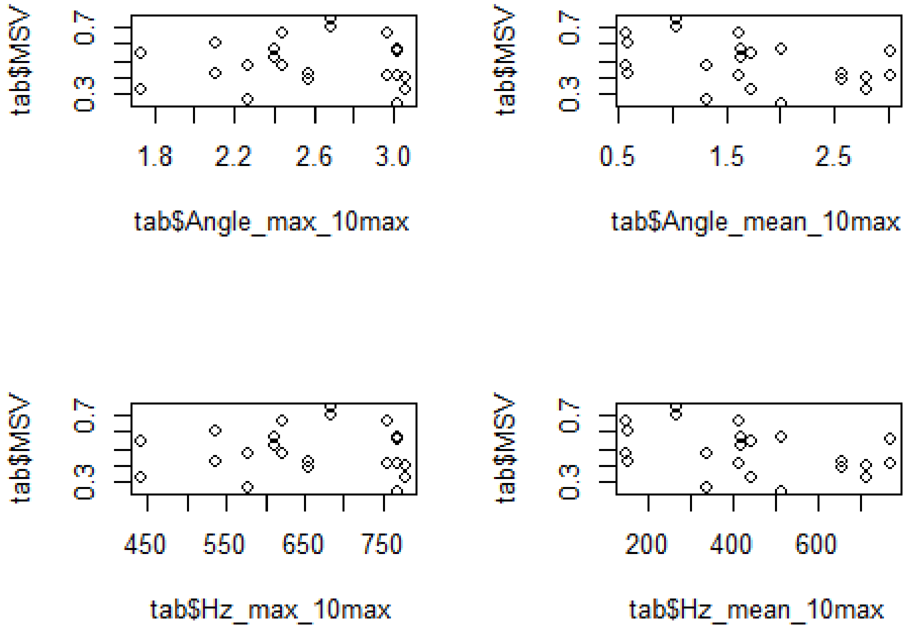

#Scatter plots at threshold 10-Mean

```
par(mfrow = c(2,2))
plot(tab$MSV~tab$Duration.ms._10mean)#*
plot(tab$MSV~tab$scalar_max_10mean)
plot(tab$MSV~tab$scalar_mean_10mean)
plot(tab$MSV~tab$changescalar_max_10mean)
```

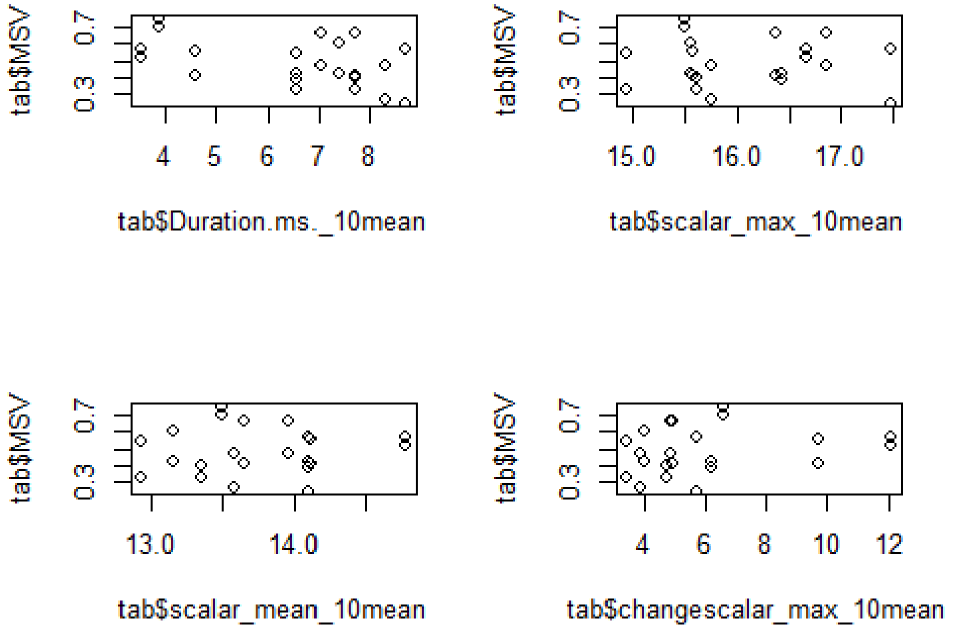


```
plot(tab$MSV~tab$changescalar_mean_10mean)
plot(tab$MSV~tab$Changevector_max_10mean)
plot(tab$MSV~tab$Changevector_mean_10mean)
plot(tab$MSV~tab$Angle_max_10mean)
```

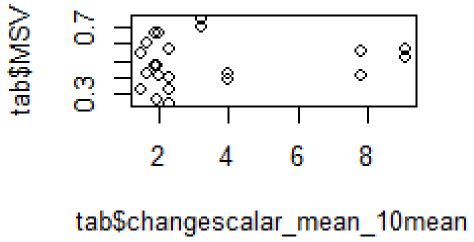

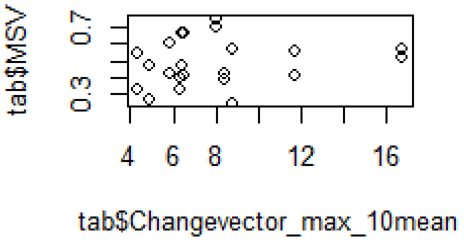

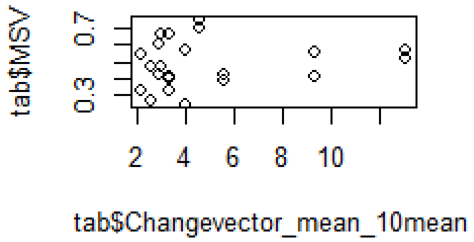

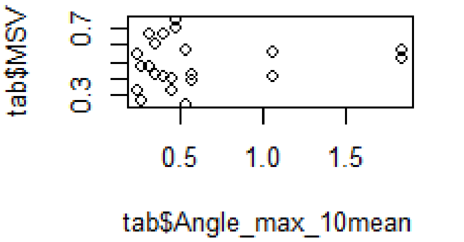


```
plot(tab$MSV~tab$Angle_mean_10mean)
plot(tab$MSV~tab$Hz_max_10mean)
plot(tab$MSV~tab$Hz_mean_10mean)
```

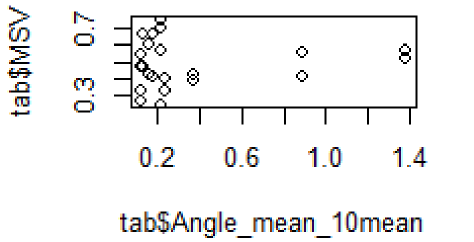

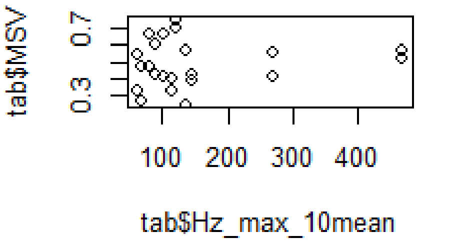

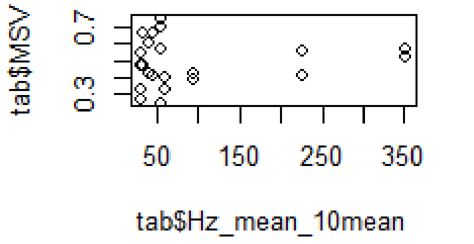

#Scatter plots at threshold 15-Max

```
par(mfrow = c(2,2))
plot(tab$MSV~tab$Events_15max)
plot(tab$MSV~tab$Duration.ms._15max)
plot(tab$MSV~tab$scalar_max_15mean )#*
plot(tab$MSV~tab$scalar_mean_15max)
```

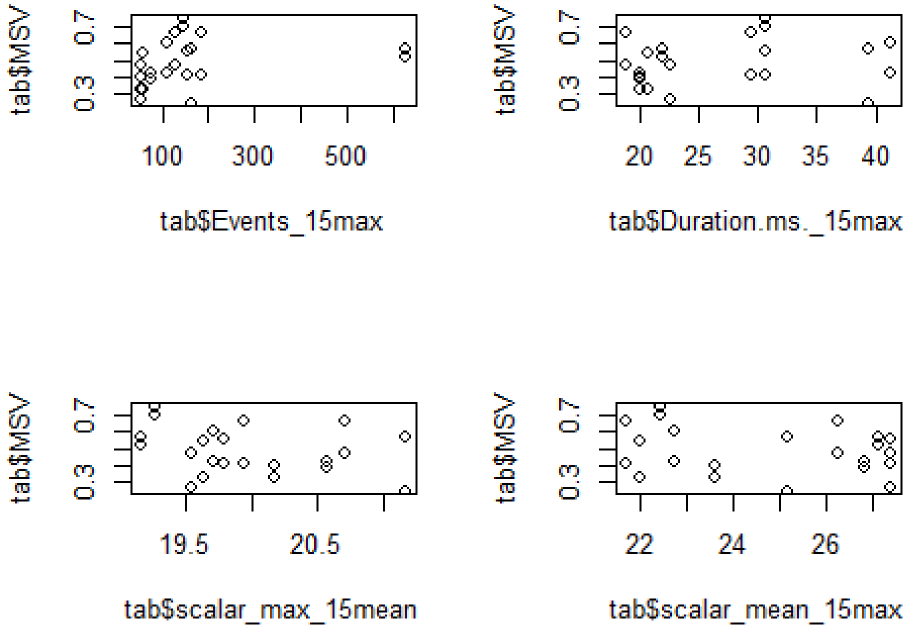


```
plot(tab$MSV~tab$changescalar_max_15max)
plot(tab$MSV~tab$changescalar_mean_15max)
plot(tab$MSV~tab$Changevector_max_15max)
plot(tab$MSV~tab$Changevector_mean_15max)
```

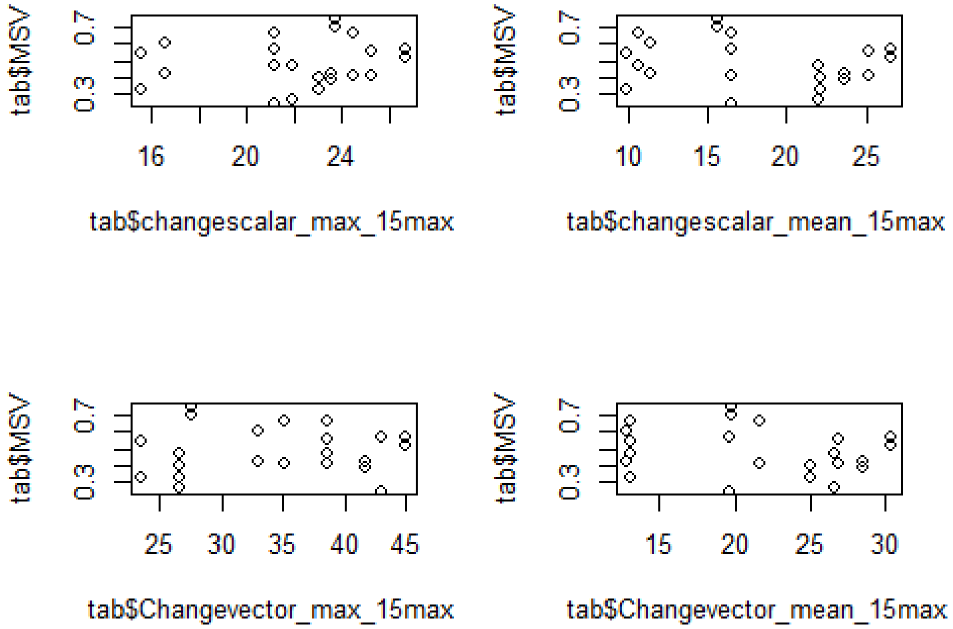


```
plot(tab$MSV~tab$Angle_max_15max)#*
plot(tab$MSV~tab$Angle_mean_15max)
plot(tab$MSV~tab$Hz_max_15max) #*
plot(tab$MSV~tab$Hz_mean_15max)
```

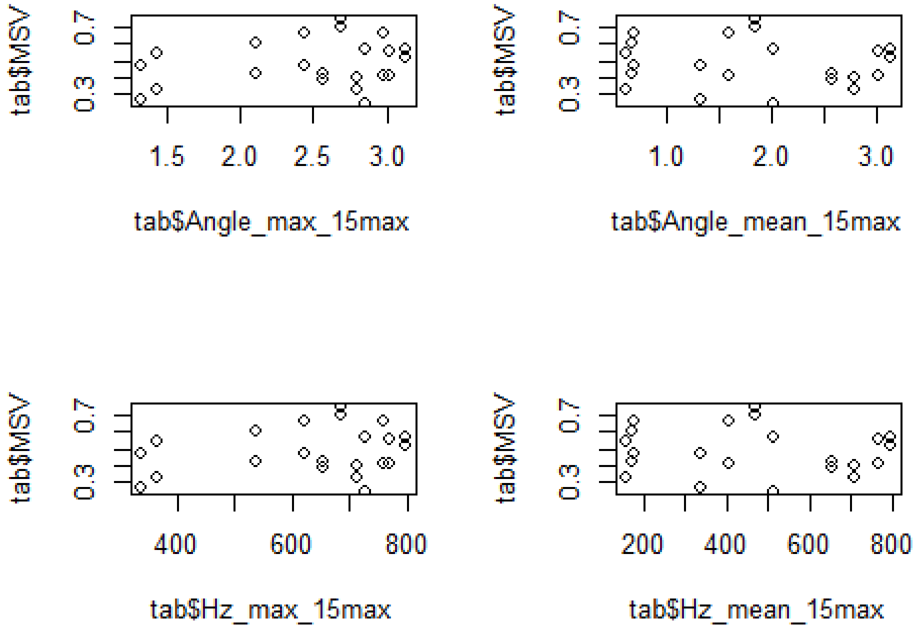

#Scatter plots at threshold 15-Mean

```
par(mfrow = c(2,2))
plot(tab$MSV~tab$Duration.ms._15mean)#*
cor.test(tab$MSV,tab$Duration.ms._15mean)

##
##  Pearson's product-moment correlation
##
## data:  tab$MSV and tab$Duration.ms._15mean
## t = -2.2615, df = 20, p-value = 0.03503
## alternative hypothesis: true correlation is not equal to 0
## 95 percent confidence interval:
##  -0.7333495 -0.0366266
## sample estimates:
##        cor
## -0.4512666

plot(tab$MSV~tab$scalar_max_15mean)
plot(tab$MSV~tab$scalar_mean_15mean)
plot(tab$MSV~tab$changescalar_max_15mean)#*
```

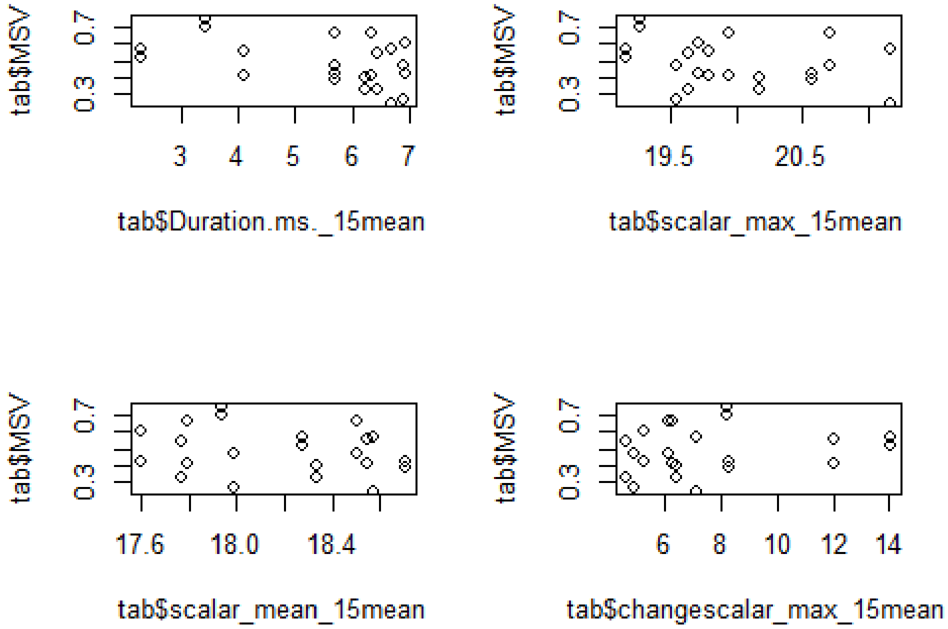


```
plot(tab$MSV~tab$changescalar_mean_15mean)
plot(tab$MSV~tab$Changevector_max_15mean)
plot(tab$MSV~tab$Changevector_mean_15mean)
plot(tab$MSV~tab$Angle_max_15mean)
```

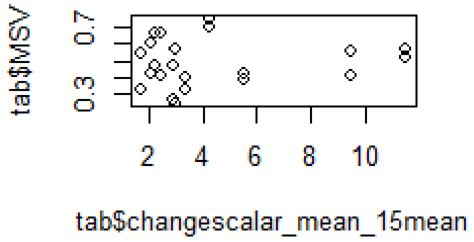

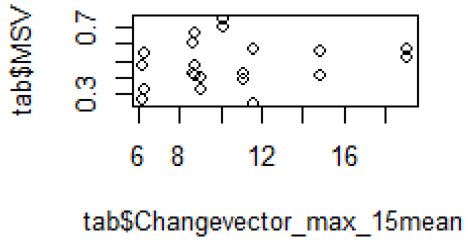

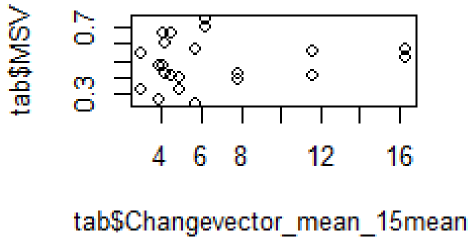

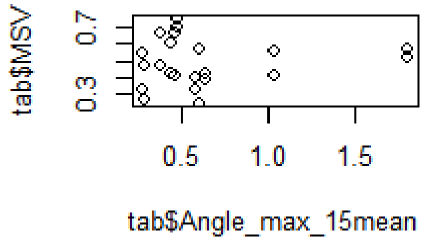


```
plot(tab$MSV~tab$Angle_mean_15mean)
plot(tab$MSV~tab$Hz_max_15mean)
plot(tab$MSV~tab$Hz_mean_15mean)
```

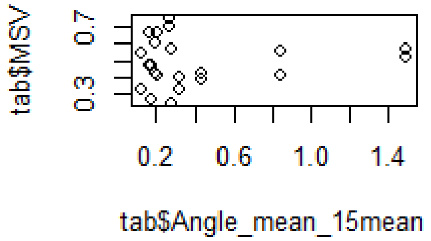

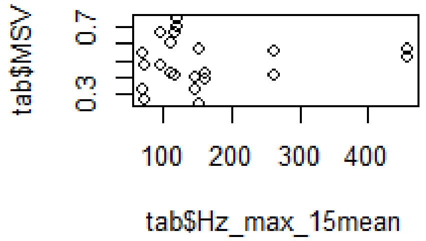

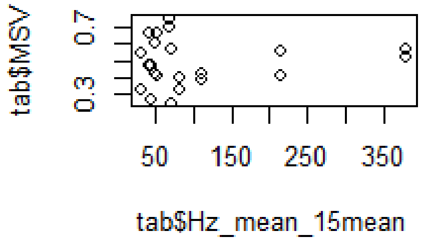

#Scatter plots at threshold 20-Max

```
par(mfrow = c(2,2))
plot(tab$MSV~tab$Events_20mean)
plot(tab$MSV~tab$Duration.ms._20max)#*
plot(tab$MSV~tab$scalar_max_20max)
plot(tab$MSV~tab$scalar_mean_20max)#*
```

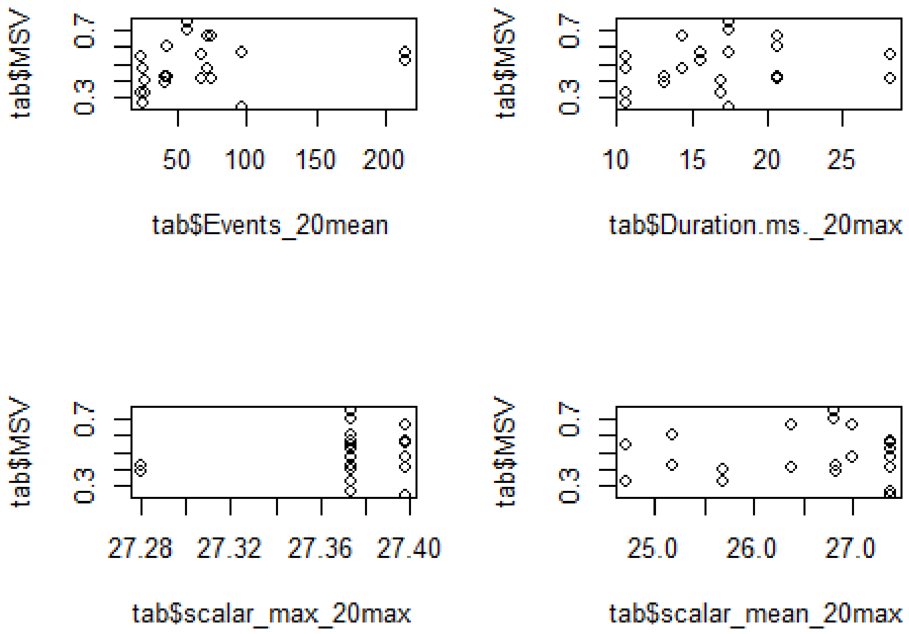


```
plot(tab$MSV~tab$changescalar_max_20max)
plot(tab$MSV~tab$changescalar_mean_20max)
plot(tab$MSV~tab$Changevector_max_20max)#*
plot(tab$MSV~tab$Changevector_mean_20max)
```

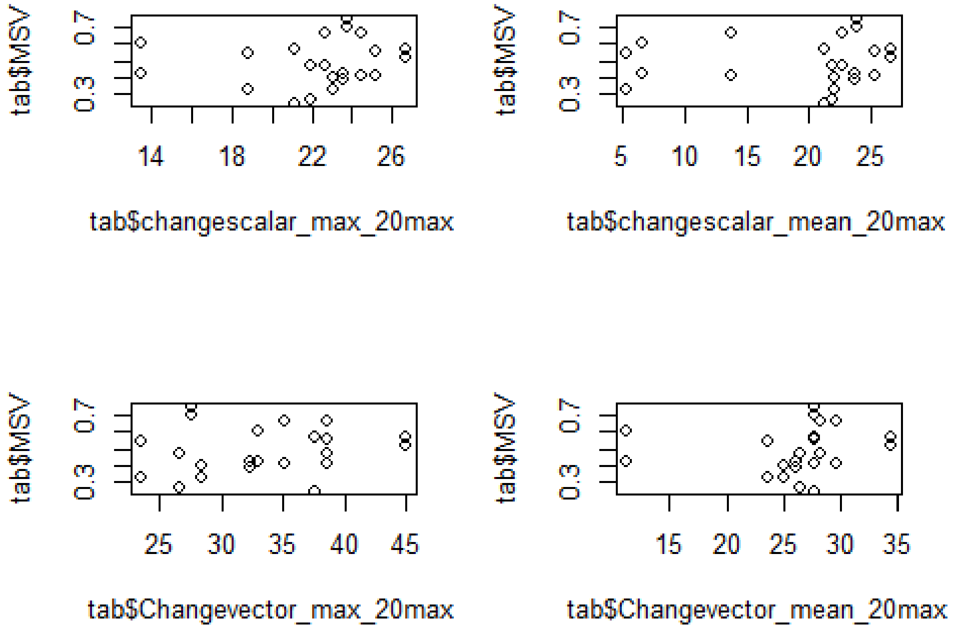


```
plot(tab$MSV~tab$Angle_max_20max)#*
plot(tab$MSV~tab$Angle_mean_20max)
plot(tab$MSV~tab$Hz_max_20max)#*
plot(tab$MSV~tab$Hz_mean_20max)#*
```

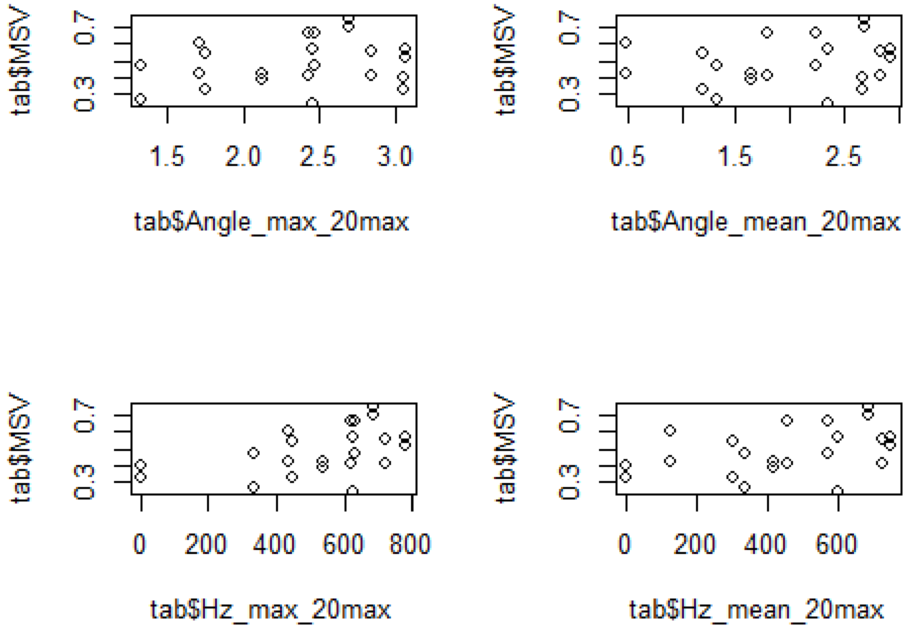

#Scatter plots at threshold 20-Mean

```
par(mfrow = c(2,2))
plot(tab$MSV~tab$Duration.ms._20mean)#*
plot(tab$MSV~tab$scalar_max_20mean)
plot(tab$MSV~tab$scalar_mean_20mean)
plot(tab$MSV~tab$changescalar_max_20mean)
```

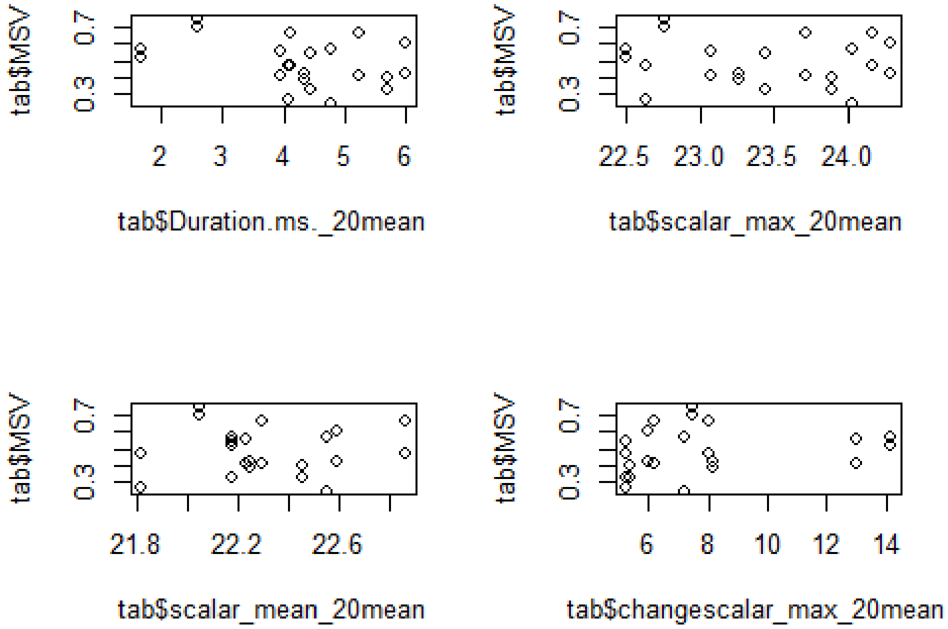


```
plot(tab$MSV~tab$changescalar_mean_20mean)
plot(tab$MSV~tab$Changevector_max_20mean)#*
plot(tab$MSV~tab$Changevector_mean_20mean)
plot(tab$MSV~tab$Angle_max_20mean)
```

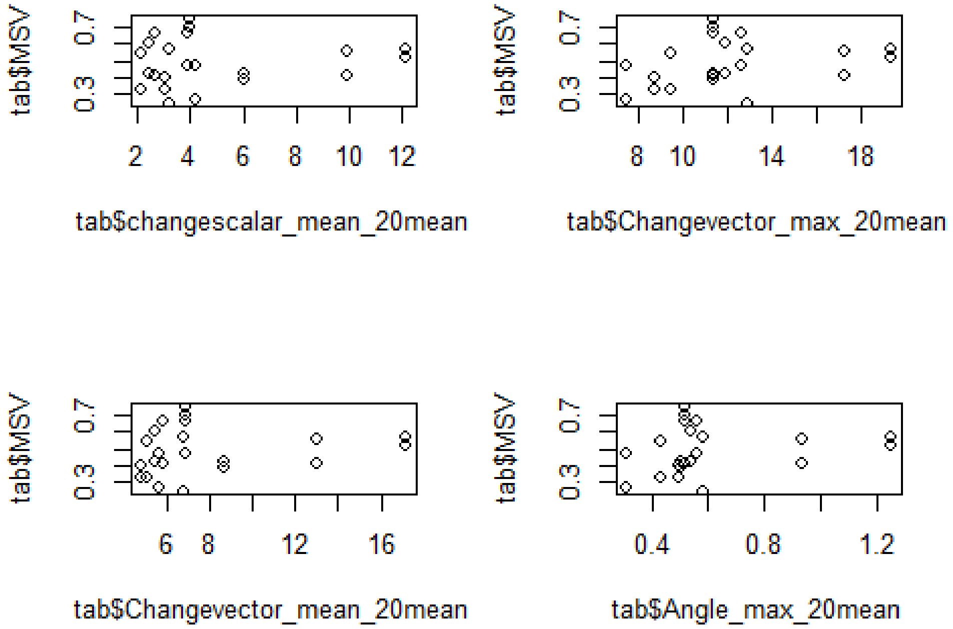


```
plot(tab$MSV~tab$Angle_mean_20mean)
plot(tab$MSV~tab$Hz_max_20mean)#*
plot(tab$MSV~tab$Hz_mean_20mean)
```

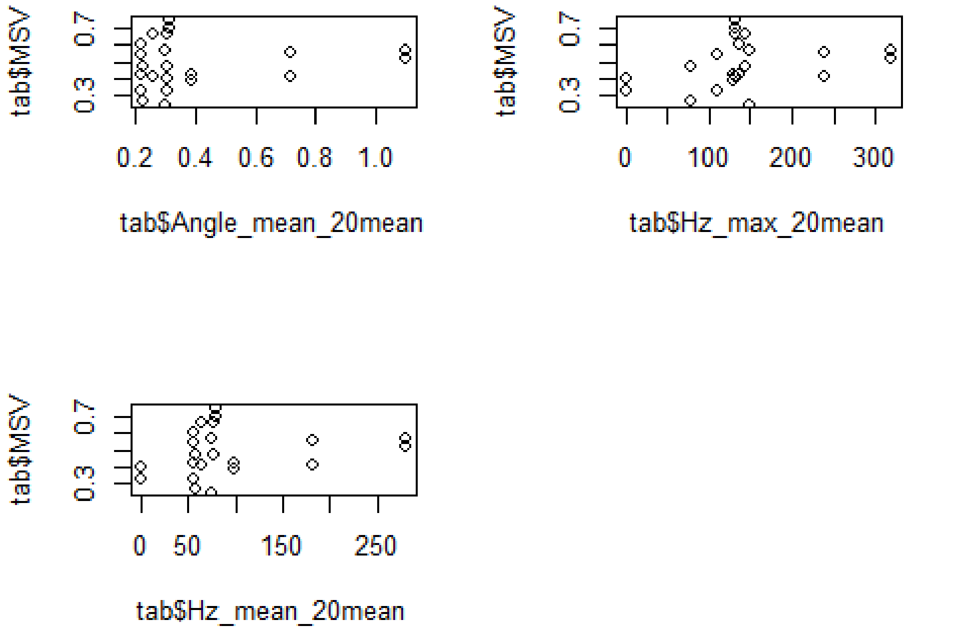

#Models of the impact of shock during transportation, on the Mean Spermathecal Value (MSV)

```
#models threshold 5

fm1 <- lme(MSV  ~ Irradiation + RH._Max + Temp_mean, random=~1|Replicate, data = tab)
fm2 <- lme(MSV  ~ Irradiation + RH._Max + Temp_mean + changescalar_mean_5max + Changevector_mean_5max, random=~1|Replicate,  data = tab)
fm3 <- lme(MSV  ~ Irradiation + RH._Max + Temp_mean + changescalar_mean_5max +Angle_mean_5max ,  random=~1|Replicate,  data = tab)
fm4 <- lme(MSV  ~ Irradiation + RH._Max + Temp_mean + changescalar_mean_5max +Duration.ms._5mean, random=~1|Replicate, data = tab)
fm5 <- lme(MSV  ~ Irradiation + RH._Max + Temp_mean + Duration.ms._5mean, random=~1|Replicate,  data = tab)
fm6 <- lme(MSV  ~ Irradiation + RH._Max + Temp_mean + changescalar_mean_5max + Angle_mean_5max + Duration.ms._5mean, random=~1|Replicate,  data = tab)
fm7 <- lme(MSV  ~ Irradiation + RH._Max + Temp_mean +Changevector_mean_5max,random=~1|Replicate,  data = tab)
fm8 <- lme(MSV  ~ Irradiation + Temp_mean + changescalar_mean_5max + Duration.ms._5max +Angle_mean_5max, random=~1|Replicate, data = tab)

AICc(fm1,fm2,fm3,fm4,fm5,fm6,fm7,fm8)

##     df     AICc
## fm1  6 12.95152
## fm2  8 37.41300
## fm3  8 32.30685
## fm4  8 34.71652
## fm5  7 20.93747
## fm6  9 42.89954
## fm7  7 25.79419
## fm8  8 33.98517

summary(fm1)

## Linear mixed-effects model fit by REML
##   Data: tab
##         AIC      BIC   logLik
##   7.351523 12.69375 2.324239
##
## Random effects:
##  Formula: ~1 | Replicate
##         (Intercept)  Residual
## StdDev: 6.625209e-07 0.1256091
##
## Fixed effects:  MSV ~ Irradiation + RH._Max + Temp_mean
##                     Value  Std.Error DF   t-value p-value
## (Intercept)     0.6345224 0.18657697 13  3.400861  0.0047
## Irradiationyes -0.0719857 0.05355988 13 -1.344022  0.2019
```

```
## RH._Max        -0.0041538 0.00244932 13 -1.695913  0.1137
## Temp_mean       0.0101843 0.00548378 13  1.857177  0.0861
##  Correlation:
##                (Intr) Irrdtn RH._Mx
## Irradiationyes -0.144
## RH._Max        -0.840  0.000
## Temp_mean      -0.376  0.000 -0.145
##
## Standardized Within-Group Residuals:
##        Min         Q1        Med         Q3        Max
## -2.0498404 -0.8338741  0.2317473  0.7122350  1.4496435
##
## Number of Observations: 22
## Number of Groups: 6
```

```
##models for threshold 10

fm11 <- lme(MSV  ~ Irradiation + Temp_mean +
changescalar_mean_10max+Angle_mean_10max, random=~1|Replicate, data = tab)
fm12 <- lme(MSV  ~ Irradiation + Temp_mean + Duration.ms._10mean +
Changevector_mean_10max + Hz_mean_10max , random=~1|Replicate,  data = tab)
fm13 <- lme(MSV  ~ Irradiation + Temp_mean + Duration.ms._10max +
Changevector_mean_10max + Hz_mean_10max , random=~1|Replicate, data = tab)
fm14 <- lme(MSV  ~ Irradiation + Duration.ms._10mean +
changescalar_mean_10max+ Hz_mean_10max , random=~1|Replicate,  data = tab)
fm15 <- lme(MSV  ~ Irradiation + Temp_mean +Angle_mean_10max +
changescalar_mean_10max, random=~1|Replicate,  data = tab)
fm11a <- lme(MSV  ~ Irradiation + Temp_mean + Duration.ms._10max +
Changevector_mean_10max , random=~1|Replicate,  data = tab)
fm11b <- lme(MSV  ~ Irradiation + Temp_mean + Duration.ms._10mean +
changescalar_mean_10max, random=~1|Replicate,  data = tab)
fm11c <- lme(MSV  ~ Irradiation + Temp_mean + Duration.ms._10mean ,
random=~1|Replicate, data = tab)
fm11d <- lme(MSV  ~ Irradiation + Temp_mean +Duration.ms._10mean
+Hz_mean_10max, random=~1|Replicate,  data = tab)

AICc(fm1,fm2,fm3,fm4,fm5,fm6,fm7,fm8,fm11,fm12,fm13,fm14,fm15,fm11a,fm11b,fm1
1c,fm11d)
```

```
##       df     AICc
## fm1    6 12.95152
## fm2    8 37.41300
## fm3    8 32.30685
## fm4    8 34.71652
## fm5    7 20.93747
## fm6    9 42.89954
## fm7    7 25.79419
## fm8    8 33.98517
## fm11   7 20.43579
## fm12   8 34.48290
```

```
## fm13   8 45.03847
## fm14   7 23.49130
## fm15   7 20.43579
## fm11a  7 25.66582
## fm11b  7 14.46886
## fm11c  6  1.42076
## fm11d  7 21.04504
```

```r
summary(fm11c)
```

```
## Linear mixed-effects model fit by REML
##   Data: tab
##         AIC      BIC  logLik
##   -4.17924 1.162991 8.08962
##
## Random effects:
##  Formula: ~1 | Replicate
##          (Intercept)  Residual
## StdDev: 2.046754e-06 0.1014062
##
## Fixed effects:  MSV ~ Irradiation + Temp_mean + Duration.ms._10mean
##                          Value  Std.Error DF   t-value p-value
## (Intercept)          0.6101504 0.10413728 13  5.859097  0.0001
## Irradiationyes      -0.0719857 0.04323976 13 -1.664803  0.1199
## Temp_mean            0.0138606 0.00458120 13  3.025535  0.0097
## Duration.ms._10mean -0.0501513 0.01338896 13 -3.745720  0.0024
##  Correlation:
##                     (Intr) Irrdtn Tmp_mn
## Irradiationyes      -0.208
## Temp_mean           -0.516  0.000
## Duration.ms._10mean -0.619  0.000 -0.293
##
## Standardized Within-Group Residuals:
##        Min         Q1        Med         Q3        Max
## -1.9012259 -0.6347567  0.2285611  0.6252662  1.3244842
##
## Number of Observations: 22
## Number of Groups: 6
```

```r
#

##threshold 15

fm21 <-lme(MSV  ~ Irradiation + Temp_mean + Duration.ms._15mean ,
random=~1|Replicate, data = tab)
fm22 <- lme(MSV  ~ Irradiation + Temp_mean +Angle_mean_15max,
random=~1|Replicate, data = tab)
fm23 <- lme(MSV  ~ Irradiation + Temp_mean +Hz_max_15max ,
random=~1|Replicate,data = tab)
fm24 <-lme(MSV  ~ Irradiation + Temp_mean +Hz_max_15max +Duration.ms._15mean
```

```
, random=~1|Replicate, data = tab)
fm25 <- lme(MSV  ~ Irradiation +Temp_mean + Duration.ms._15mean
+Angle_mean_15max, random=~1|Replicate, data = tab)
fm26 <- lme(MSV  ~ Irradiation +Temp_mean, random=~1|Replicate, data = tab)
fm27 <- lme(MSV  ~ Irradiation +Angle_mean_15max, random=~1|Replicate, data =
tab)
fm28 <- lme(MSV  ~ Irradiation + Duration.ms._15mean , random=~1|Replicate,
data = tab)
fm29 <- lme(MSV  ~ Irradiation +Angle_mean_15max+  Duration.ms._15mean ,
random=~1|Replicate, data = tab)
fm30 <- lme(MSV  ~ Irradiation +Temp_mean +Angle_mean_15max ,
random=~1|Replicate, data = tab)
fm30a <- lme(MSV  ~ Irradiation +Temp_mean + Duration.ms._15mean ,
random=~1|Replicate,data = tab)
fm29a<- lme(MSV  ~ Irradiation + Duration.ms._15mean +scalar_mean_15mean,
random=~1|Replicate, data = tab)

AICc(fm1,fm2,fm3,fm4,fm5,fm6,fm7,fm8,fm13,fm14,fm15,fm11a,fm11b,fm11c,fm11d,f
m21,fm22,fm23,fm24,fm25,fm26,fm27,fm28,fm29,fm30,fm30a,fm29a)

##        df        AICc
## fm1     6 12.95152275
## fm2     8 37.41300203
## fm3     8 32.30685001
## fm4     8 34.71652434
## fm5     7 20.93747101
## fm6     9 42.89953604
## fm7     7 25.79418667
## fm8     8 33.98517465
## fm13    8 45.03846552
## fm14    7 23.49129684
## fm15    7 20.43579190
## fm11a   7 25.66582332
## fm11b   7 14.46886083
## fm11c   6  1.42076048
## fm11d   7 21.04504050
## fm21    6  3.43872987
## fm22    6  9.91050570
## fm23    6 19.58463656
## fm24    7 22.75735837
## fm25    7 10.68372496
## fm26    5  1.70467966
## fm27    5  0.42816364
## fm28    5 -3.14415681
## fm29    6 -0.08972187
## fm30    6  9.91050570
## fm30a   6  3.43872987
## fm29a   6  1.44928901

summary(fm28)
```

```
## Linear mixed-effects model fit by REML
##   Data: tab
##         AIC       BIC   logLik
##   -6.894157 -2.171962 8.447078
##
## Random effects:
##  Formula: ~1 | Replicate
##          (Intercept)  Residual
## StdDev: 1.135568e-06 0.1235004
##
## Fixed effects:  MSV ~ Irradiation + Duration.ms._15mean
##                          Value  Std.Error DF   t-value p-value
## (Intercept)          0.7494934 0.10532507 14  7.116002  0.0000
## Irradiationyes      -0.0719857 0.05266076 14 -1.366970  0.1932
## Duration.ms._15mean -0.0412491 0.01785618 14 -2.310072  0.0366
##  Correlation:
##                     (Intr) Irrdtn
## Irradiationyes      -0.250
## Duration.ms._15mean -0.935  0.000
##
## Standardized Within-Group Residuals:
##         Min          Q1         Med          Q3         Max
## -1.32931727 -0.74322987 -0.08387532  0.66845220  1.73655234
##
## Number of Observations: 22
## Number of Groups: 6
```

```
##Threshold 20

fm31 <- lme(MSV  ~ Irradiation + Temp_mean + Duration.ms._20max +
Angle_mean_20max +Hz_max_20mean, random=~1|Replicate,data = tab)
fm32 <- lme(MSV  ~ Irradiation + Temp_mean +Duration.ms._20mean
+Hz_max_20mean, random=~1|Replicate, data = tab)
fm31a <-lme(MSV  ~ Irradiation + Temp_mean + Changevector_max_20mean +
Hz_max_20max , random=~1|Replicate, data = tab)
fm32a <- lme(MSV  ~ Irradiation + Temp_mean +Changevector_max_20mean
+Angle_mean_20max , random=~1|Replicate,data = tab)
fm32b <- lme(MSV  ~ Irradiation + Temp_mean +  Changevector_max_20max
+Duration.ms._20mean, random=~1|Replicate,data = tab)
fm31b <- lme(MSV  ~ Irradiation + Temp_mean + Changevector_max_20mean +
Hz_max_20max , random=~1|Replicate, data = tab)
fm32c <- lme(MSV  ~ Irradiation + Temp_mean + Hz_max_20max ,
random=~1|Replicate, data = tab)


AICc(fm1,fm2,fm3,fm4,fm5,fm6,fm7,fm8,fm13,fm14,fm15,fm11a,fm11b,fm11c,fm11d,f
m21,fm22,fm23,fm24,fm25,fm26,fm27,fm28,fm29,fm30,fm30a,fm29a,fm31,fm32,fm31a,
fm32a,fm31b,fm32b,fm32c)
```

```
##       df        AICc
## fm1    6 12.95152275
## fm2    8 37.41300203
## fm3    8 32.30685001
## fm4    8 34.71652434
## fm5    7 20.93747101
## fm6    9 42.89953604
## fm7    7 25.79418667
## fm8    8 33.98517465
## fm13   8 45.03846552
## fm14   7 23.49129684
## fm15   7 20.43579190
## fm11a  7 25.66582332
## fm11b  7 14.46886083
## fm11c  6  1.42076048
## fm11d  7 21.04504050
## fm21   6  3.43872987
## fm22   6  9.91050570
## fm23   6 19.58463656
## fm24   7 22.75735837
## fm25   7 10.68372496
## fm26   5  1.70467966
## fm27   5  0.42816364
## fm28   5 -3.14415681
## fm29   6 -0.08972187
## fm30   6  9.91050570
## fm30a  6  3.43872987
## fm29a  6  1.44928901
## fm31   8 38.31850922
## fm32   7 23.96985983
## fm31a  7 29.12183360
## fm32a  7 19.58402679
## fm31b  7 29.12183360
## fm32b  7 19.42850333
## fm32c  6 17.87770235
```

```
summary(fm28)
```

```
## Linear mixed-effects model fit by REML
##   Data: tab
##         AIC       BIC   logLik
##   -6.894157 -2.171962 8.447078
##
## Random effects:
##  Formula: ~1 | Replicate
##          (Intercept)  Residual
## StdDev: 1.135568e-06 0.1235004
##
## Fixed effects:  MSV ~ Irradiation + Duration.ms._15mean
##                           Value  Std.Error DF   t-value p-value
```

```
## (Intercept)          0.7494934 0.10532507 14  7.116002  0.0000
## Irradiationyes      -0.0719857 0.05266076 14 -1.366970  0.1932
## Duration.ms._15mean -0.0412491 0.01785618 14 -2.310072  0.0366
##  Correlation:
##                     (Intr) Irrdtn
## Irradiationyes      -0.250
## Duration.ms._15mean -0.935  0.000
##
## Standardized Within-Group Residuals:
##         Min          Q1         Med          Q3         Max
## -1.32931727 -0.74322987 -0.08387532  0.66845220  1.73655234
##
## Number of Observations: 22
## Number of Groups: 6
```

```
#combination of thresehold
fm41 <- lme(MSV  ~ Irradiation + Temp_mean + Duration.ms._10mean +
changescalar_mean_10max+Angle_mean_15max , random=~1|Replicate, data = tab)
fm42 <- lme(MSV  ~ Irradiation + Temp_mean + Duration.ms._10mean +
changescalar_mean_10max+Hz_mean_10max + Duration.ms._15mean ,
random=~1|Replicate, data = tab)
summary(fm28)
```

```
## Linear mixed-effects model fit by REML
##   Data: tab
##         AIC       BIC   logLik
##   -6.894157 -2.171962 8.447078
##
## Random effects:
##  Formula: ~1 | Replicate
##          (Intercept)  Residual
## StdDev: 1.135568e-06 0.1235004
##
## Fixed effects:  MSV ~ Irradiation + Duration.ms._15mean
##                          Value  Std.Error DF   t-value p-value
## (Intercept)          0.7494934 0.10532507 14  7.116002  0.0000
## Irradiationyes      -0.0719857 0.05266076 14 -1.366970  0.1932
## Duration.ms._15mean -0.0412491 0.01785618 14 -2.310072  0.0366
##  Correlation:
##                     (Intr) Irrdtn
## Irradiationyes      -0.250
## Duration.ms._15mean -0.935  0.000
##
## Standardized Within-Group Residuals:
##         Min          Q1         Med          Q3         Max
## -1.32931727 -0.74322987 -0.08387532  0.66845220  1.73655234
##
## Number of Observations: 22
## Number of Groups: 6
```

```
AICc(fm1,fm2,fm3,fm4,fm5,fm6,fm7,fm8,fm13,fm14,fm15,fm11a,fm11b,fm11c,fm11d,f
m21,fm22,fm23,fm24,fm25,fm26,fm27,fm28,fm29,fm30,fm30a,fm29a,fm31,fm32,fm31a,
fm32a,fm31b,fm32b,fm32c,fm41,fm42)
```

```
##       df        AICc
## fm1    6 12.95152275
## fm2    8 37.41300203
## fm3    8 32.30685001
## fm4    8 34.71652434
## fm5    7 20.93747101
## fm6    9 42.89953604
## fm7    7 25.79418667
## fm8    8 33.98517465
## fm13   8 45.03846552
## fm14   7 23.49129684
## fm15   7 20.43579190
## fm11a  7 25.66582332
## fm11b  7 14.46886083
## fm11c  6  1.42076048
## fm11d  7 21.04504050
## fm21   6  3.43872987
## fm22   6  9.91050570
## fm23   6 19.58463656
## fm24   7 22.75735837
## fm25   7 10.68372496
## fm26   5  1.70467966
## fm27   5  0.42816364
## fm28   5 -3.14415681
## fm29   6 -0.08972187
## fm30   6  9.91050570
## fm30a  6  3.43872987
## fm29a  6  1.44928901
## fm31   8 38.31850922
## fm32   7 23.96985983
## fm31a  7 29.12183360
## fm32a  7 19.58402679
## fm31b  7 29.12183360
## fm32b  7 19.42850333
## fm32c  6 17.87770235
## fm41   8 23.95078897
## fm42   9 44.28677516
```

```
summary(fm28)
```

```
## Linear mixed-effects model fit by REML
##   Data: tab
##         AIC       BIC   logLik
##   -6.894157 -2.171962 8.447078
##
## Random effects:
```

```
##  Formula: ~1 | Replicate
##         (Intercept)  Residual
## StdDev: 1.135568e-06 0.1235004
##
## Fixed effects:  MSV ~ Irradiation + Duration.ms._15mean
##                          Value  Std.Error DF   t-value p-value
## (Intercept)          0.7494934 0.10532507 14  7.116002  0.0000
## Irradiationyes      -0.0719857 0.05266076 14 -1.366970  0.1932
## Duration.ms._15mean -0.0412491 0.01785618 14 -2.310072  0.0366
##  Correlation:
##                     (Intr) Irrdtn
## Irradiationyes      -0.250
## Duration.ms._15mean -0.935  0.000
##
## Standardized Within-Group Residuals:
##         Min          Q1         Med          Q3         Max
## -1.32931727 -0.74322987 -0.08387532  0.66845220  1.73655234
##
## Number of Observations: 22
## Number of Groups: 6

#the best model remains as fm28

##Fig 9e

head(tab)

##     Date Replicate Age     Treatments not_full full  MSV MSV2 shipped
## 1 170510        R1  22 Shipped-110Gy        2    6 0.75 1.50     yes
## 2 170510        R1  22   Shipped-0Gy        4    6 0.70 1.40     yes
## 3 170517        R2  22 Shipped-110Gy       22    5 0.41 0.82     yes
## 4 170517        R2  22   Shipped-0Gy       13   17 0.66 1.32     yes
## 5 170524        R3  22 Shipped-110Gy        6    7 0.60 1.20     yes
## 6 170524        R3  22   Shipped-0Gy       22    4 0.42 0.84     yes
##   Irradiation Events._5max Duration.ms._5max scalar_max_5max
scalar_mean_5max
## 1         yes          909            48.750          27.374
21.43900
## 2          no          909            48.750          27.374
21.43900
## 3         yes          708            87.500          27.398
16.92723
## 4          no          708            87.500          27.398
16.92723
## 5         yes         1187            93.125          27.374
15.43705
## 6          no         1187            93.125          27.374
15.43705
##   changescalar_max_5max changescalar_mean_5max Changevector_max_5max
## 1                23.724                8.94875              27.69156
```

```
## 2                23.724               8.94875             27.69156
## 3                24.477              11.37550             35.06987
## 4                24.477              11.37550             35.06987
## 5                16.627               3.83800             33.00443
## 6                16.627               3.83800             33.00443
##   Changevector_mean_5max Angle_max_5max Angle_mean_5max Hz_max_5max
## 1               9.937386       3.002116        2.617132    764.4826
## 2               9.937386       3.002116        2.617132    764.4826
## 3              19.035967       2.969360        1.679485    756.1414
## 4              19.035967       2.969360        1.679485    756.1414
## 5               5.064434       2.105922        1.356174    536.2686
## 6               5.064434       2.105922        1.356174    536.2686
##   Hz_mean_5max Events_10max Duration.ms._10max scalar_max_10max
## 1     666.4471          288             36.875           27.374
## 2     666.4471          288             36.875           27.374
## 3     427.6775          315             50.000           27.398
## 4     427.6775          315             50.000           27.398
## 5     345.3468          238             63.750           27.374
## 6     345.3468          238             63.750           27.374
##   scalar_mean_10max changescalar_max_10max changescalar_mean_10max
## 1           21.5800                 23.724                  12.473
## 2           21.5800                 23.724                  12.473
## 3           20.3750                 24.477                  16.488
## 4           20.3750                 24.477                  16.488
## 5           19.6895                 16.627                   8.965
## 6           19.6895                 16.627                   8.965
##   Changevector_max_10max Changevector_mean_10max Angle_max_10max
## 1               27.69156                15.45774        2.687392
## 2               27.69156                15.45774        2.687392
## 3               35.06987                22.58486        2.969360
## 4               35.06987                22.58486        2.969360
## 5               33.00443                10.65686        2.105922
## 6               33.00443                10.65686        2.105922
##   Angle_mean_10max Hz_max_10max Hz_mean_10max Events_15max
Duration.ms._15max
## 1        1.0470779     684.3388      266.6362          144
30.625
## 2        1.0470779     684.3388      266.6362          144
30.625
## 3        1.6138687     756.1414      410.9683          183
29.375
## 4        1.6138687     756.1414      410.9683          183
29.375
## 5        0.5851573     536.2686      149.0091          111
41.250
## 6        0.5851573     536.2686      149.0091          111
41.250
##   scalar_max_15max scalar_mean_15max changescalar_max_15max
## 1           27.374          22.42986                 23.724
## 2           27.374          22.42986                 23.724
```

```
## 3          27.398          21.71558                   24.477
## 4          27.398          21.71558                   24.477
## 5          27.374          22.77170                   16.627
## 6          27.374          22.77170                   16.627
##   changescalar_mean_15max Changevector_max_15max Changevector_mean_15max
## 1                  15.667               27.69156                19.75723
## 2                  15.667               27.69156                19.75723
## 3                  16.488               35.06987                21.61531
## 4                  16.488               35.06987                21.61531
## 5                  11.474               33.00443                12.83840
## 6                  11.474               33.00443                12.83840
##   Angle_max_15max Angle_mean_15max Hz_max_15max Hz_mean_15max Events_20max
## 1        2.687392        1.8427590     684.3388      469.2547           57
## 2        2.687392        1.8427590     684.3388      469.2547           57
## 3        2.969360        1.6010884     756.1414      407.7138           75
## 4        2.969360        1.6010884     756.1414      407.7138           75
## 5        2.105922        0.6709018     536.2686      170.8438           42
## 6        2.105922        0.6709018     536.2686      170.8438           42
##   Duration.ms._20max scalar_max_20max scalar_mean_20max
changescalar_max_20max
## 1             17.500           27.374          26.80175
23.724
## 2             17.500           27.374          26.80175
23.724
## 3             20.625           27.398          26.38567
24.477
## 4             20.625           27.398          26.38567
24.477
## 5             20.625           27.374          25.17900
13.480
## 6             20.625           27.374          25.17900
13.480
##   changescalar_mean_20max Changevector_max_20max Changevector_mean_20max
## 1                 23.7240               27.69156                27.69156
## 2                 23.7240               27.69156                27.69156
## 3                 13.7095               35.06987                29.65156
## 4                 13.7095               35.06987                29.65156
## 5                  6.6090               33.00443                11.19072
## 6                  6.6090               33.00443                11.19072
##   Angle_max_20max Angle_mean_20max Hz_max_20max Hz_mean_20max Events_5mean
## 1        2.687392         2.687392     684.3388      684.3388          909
## 2        2.687392         2.687392     684.3388      684.3388          909
## 3        2.433775         1.792941     619.7557      456.5688          708
## 4        2.433775         1.792941     619.7557      456.5688          708
## 5        1.715913         0.489164     436.9538      124.5646         1187
## 6        1.715913         0.489164     436.9538      124.5646         1187
##   Duration.ms._5mean scalar_max_5mean scalar_mean_5mean
changescalar_max_5mean
## 1           5.215209         9.516648          7.473747
3.755884
```

```
## 2          5.215209         9.516648          7.473747
3.755884
## 3         11.785840        10.551797          7.565272
2.816095
## 4         11.785840        10.551797          7.565272
2.816095
## 5          7.348884         8.022198          6.506753
1.632164
## 6          7.348884         8.022198          6.506753
1.632164
##   changescalar_mean_5mean Changevector_max_5mean Changevector_mean_5mean
## 1               1.6630629               4.501180                2.219564
## 2               1.6630629               4.501180                2.219564
## 3               1.0076953               3.691769                1.638802
## 4               1.0076953               3.691769                1.638802
## 5               0.6804169               2.293485                1.194539
## 6               0.6804169               2.293485                1.194539
##   Angle_max_5mean Angle_mean_5mean Hz_max_5mean Hz_mean_5mean
Events._10mean
## 1       0.4296757        0.1755708    109.41603      44.70874
288
## 2       0.4296757        0.1755708    109.41603      44.70874
288
## 3       0.3715939        0.1414327     94.62562      36.01553
315
## 4       0.3715939        0.1414327     94.62562      36.01553
315
## 5       0.2539263        0.1310499     64.66179      33.37158
238
## 6       0.2539263        0.1310499     64.66179      33.37158
238
##   Duration.ms._10mean scalar_max_10mean scalar_mean_10mean
## 1            3.888889          15.49537           13.49979
## 2            3.888889          15.49537           13.49979
## 3            7.706349          16.38244           13.65682
## 4            7.706349          16.38244           13.65682
## 5            7.415966          15.56483           13.15894
## 6            7.415966          15.56483           13.15894
##   changescalar_max_10mean changescalar_mean_10mean Changevector_max_10mean
## 1                6.557340                 3.249119                8.029862
## 2                6.557340                 3.249119                8.029862
## 3                4.928854                 1.971893                6.505797
## 4                4.928854                 1.971893                6.505797
## 5                4.004025                 1.664277                5.891075
## 6                4.004025                 1.664277                5.891075
##   Changevector_mean_10mean Angle_max_10mean Angle_mean_10mean
Hz_max_10mean
## 1                 4.538349        0.4719538         0.2224823
120.1821
## 2                 4.538349        0.4719538         0.2224823
```

```
120.1821
## 3               3.339135        0.4047630         0.1751144
103.0721
## 4               3.339135        0.4047630         0.1751144
103.0721
## 5               2.909783        0.3492638         0.1583697
88.9393
## 6               2.909783        0.3492638         0.1583697
88.9393
##   Hz_mean_10mean Events_15mean Duration.ms._15mean scalar_max_15mean
## 1       56.65466           144            3.441840          19.26462
## 2       56.65466           144            3.441840          19.26462
## 3       44.59251           183            6.342213          19.93954
## 4       44.59251           183            6.342213          19.93954
## 5       40.32851           111            6.920045          19.71768
## 6       40.32851           111            6.920045          19.71768
##   scalar_mean_15mean changescalar_max_15mean changescalar_mean_15mean
## 1           17.94158                8.195410                 4.209463
## 2           17.94158                8.195410                 4.209463
## 3           17.79343                6.271208                 2.424063
## 4           17.79343                6.271208                 2.424063
## 5           17.60073                5.291784                 2.055466
## 6           17.60073                5.291784                 2.055466
##   Changevector_max_15mean Changevector_mean_15mean Angle_max_15mean
## 1               10.145769                 6.194357        0.4732660
## 2               10.145769                 6.194357        0.4732660
## 3                8.773143                 4.485467        0.4584332
## 4                8.773143                 4.485467        0.4584332
## 5                8.644509                 4.178605        0.4437400
## 6                8.644509                 4.178605        0.4437400
##   Angle_mean_15mean Hz_max_15mean Hz_mean_15mean Events_20mean
## 1         0.2644405      120.5162       67.33922            57
## 2         0.2644405      120.5162       67.33922            57
## 3         0.2042285      116.7390       52.00635            75
## 4         0.2042285      116.7390       52.00635            75
## 5         0.1960600      112.9975       49.92626            42
## 6         0.1960600      112.9975       49.92626            42
##   Duration.ms._20mean scalar_max_20mean scalar_mean_20mean
## 1            2.587719          22.76440           22.04857
## 2            2.587719          22.76440           22.04857
## 3            5.250000          23.71752           22.29175
## 4            5.250000          23.71752           22.29175
## 5            5.997024          24.28693           22.58697
## 6            5.997024          24.28693           22.58697
##   changescalar_max_20mean changescalar_mean_20mean Changevector_max_20mean
## 1                7.502070                 3.960703                11.35123
## 2                7.502070                 3.960703                11.35123
## 3                6.190293                 2.676377                11.36833
## 4                6.190293                 2.676377                11.36833
## 5                5.996190                 2.445063                11.97291
```

```
## 6                5.996190                 2.445063                11.97291
##   Changevector_mean_20mean Angle_max_20mean Angle_mean_20mean
Hz_max_20mean
## 1                 6.922532        0.5201012         0.3140733
132.4427
## 2                 6.922532        0.5201012         0.3140733
132.4427
## 3                 5.877764        0.5214004         0.2548023
132.7735
## 4                 5.877764        0.5214004         0.2548023
132.7735
## 5                 5.476046        0.5391966         0.2201107
137.3053
## 6                 5.476046        0.5391966         0.2201107
137.3053
##   Hz_mean_20mean RH._Max Temp_Max RH._mean Temp_mean
## 1       79.97812   47.08    23.50    41.69     19.73
## 2       79.97812   60.14    27.50    41.69     19.73
## 3       64.88487   62.61    29.75    53.55     21.79
## 4       64.88487   68.10    32.00    53.55     21.79
## 5       56.05073   57.37    28.69    53.25     23.29
## 6       56.05073   74.59    19.25    53.25     23.29
```

```
str(tab)
```

```
## 'data.frame':    22 obs. of  110 variables:
##  $ Date                     : int  170510 170510 170517 170517 170524
170524 170531 170531 170607 170607 ...
##  $ Replicate                : chr  "R1" "R1" "R2" "R2" ...
##  $ Age                      : int  22 22 22 22 22 22 22 22 22 22 ...
##  $ Treatments               : chr  "Shipped-110Gy " "Shipped-0Gy "
"Shipped-110Gy " "Shipped-0Gy " ...
##  $ not_full                 : int  2 4 22 13 6 22 4 14 11 17 ...
##  $ full                     : int  6 6 5 17 7 4 0 9 8 22 ...
##  $ MSV                      : num  0.75 0.7 0.41 0.66 0.6 0.42 0.25 0.57
0.47 0.66 ...
##  $ MSV2                     : num  1.5 1.4 0.82 1.32 1.2 0.84 0.5 1.14 0.94
1.32 ...
##  $ shipped                  : chr  "yes" "yes" "yes" "yes" ...
##  $ Irradiation              : chr  "yes" "no" "yes" "no" ...
##  $ Events._5max             : int  909 909 708 708 1187 1187 625 625 606
606 ...
##  $ Duration.ms._5max        : num  48.8 48.8 87.5 87.5 93.1 ...
##  $ scalar_max_5max          : num  27.4 27.4 27.4 27.4 27.4 ...
##  $ scalar_mean_5max         : num  21.4 21.4 16.9 16.9 15.4 ...
##  $ changescalar_max_5max    : num  23.7 23.7 24.5 24.5 16.6 ...
##  $ changescalar_mean_5max   : num  8.95 8.95 11.38 11.38 3.84 ...
##  $ Changevector_max_5max    : num  27.7 27.7 35.1 35.1 33 ...
##  $ Changevector_mean_5max   : num  9.94 9.94 19.04 19.04 5.06 ...
##  $ Angle_max_5max           : num  3 3 2.97 2.97 2.11 ...
```

```
##  $ Angle_mean_5max         : num  2.62 2.62 1.68 1.68 1.36 ...
##  $ Hz_max_5max             : num  764 764 756 756 536 ...
##  $ Hz_mean_5max            : num  666 666 428 428 345 ...
##  $ Events_10max            : int  288 288 315 315 238 238 261 261 219 219
...
##  $ Duration.ms._10max      : num  36.9 36.9 50 50 63.8 ...
##  $ scalar_max_10max        : num  27.4 27.4 27.4 27.4 27.4 ...
##  $ scalar_mean_10max       : num  21.6 21.6 20.4 20.4 19.7 ...
##  $ changescalar_max_10max  : num  23.7 23.7 24.5 24.5 16.6 ...
##  $ changescalar_mean_10max : num  12.47 12.47 16.49 16.49 8.97 ...
##  $ Changevector_max_10max  : num  27.7 27.7 35.1 35.1 33 ...
##  $ Changevector_mean_10max : num  15.5 15.5 22.6 22.6 10.7 ...
##  $ Angle_max_10max         : num  2.69 2.69 2.97 2.97 2.11 ...
##  $ Angle_mean_10max        : num  1.047 1.047 1.614 1.614 0.585 ...
##  $ Hz_max_10max            : num  684 684 756 756 536 ...
##  $ Hz_mean_10max           : num  267 267 411 411 149 ...
##  $ Events_15max            : int  144 144 183 183 111 111 163 163 129 129
...
##  $ Duration.ms._15max      : num  30.6 30.6 29.4 29.4 41.2 ...
##  $ scalar_max_15max        : num  27.4 27.4 27.4 27.4 27.4 ...
##  $ scalar_mean_15max       : num  22.4 22.4 21.7 21.7 22.8 ...
##  $ changescalar_max_15max  : num  23.7 23.7 24.5 24.5 16.6 ...
##  $ changescalar_mean_15max : num  15.7 15.7 16.5 16.5 11.5 ...
##  $ Changevector_max_15max  : num  27.7 27.7 35.1 35.1 33 ...
##  $ Changevector_mean_15max : num  19.8 19.8 21.6 21.6 12.8 ...
##  $ Angle_max_15max         : num  2.69 2.69 2.97 2.97 2.11 ...
##  $ Angle_mean_15max        : num  1.843 1.843 1.601 1.601 0.671 ...
##  $ Hz_max_15max            : num  684 684 756 756 536 ...
##  $ Hz_mean_15max           : num  469 469 408 408 171 ...
##  $ Events_20max            : int  57 57 75 75 42 42 97 97 71 71 ...
##  $ Duration.ms._20max      : num  17.5 17.5 20.6 20.6 20.6 ...
##  $ scalar_max_20max        : num  27.4 27.4 27.4 27.4 27.4 ...
##  $ scalar_mean_20max       : num  26.8 26.8 26.4 26.4 25.2 ...
##  $ changescalar_max_20max  : num  23.7 23.7 24.5 24.5 13.5 ...
##  $ changescalar_mean_20max : num  23.72 23.72 13.71 13.71 6.61 ...
##  $ Changevector_max_20max  : num  27.7 27.7 35.1 35.1 33 ...
##  $ Changevector_mean_20max : num  27.7 27.7 29.7 29.7 11.2 ...
##  $ Angle_max_20max         : num  2.69 2.69 2.43 2.43 1.72 ...
##  $ Angle_mean_20max        : num  2.687 2.687 1.793 1.793 0.489 ...
##  $ Hz_max_20max            : num  684 684 620 620 437 ...
##  $ Hz_mean_20max           : num  684 684 457 457 125 ...
##  $ Events_5mean            : int  909 909 708 708 1187 1187 625 625 606
606 ...
##  $ Duration.ms._5mean      : num  5.22 5.22 11.79 11.79 7.35 ...
##  $ scalar_max_5mean        : num  9.52 9.52 10.55 10.55 8.02 ...
##  $ scalar_mean_5mean       : num  7.47 7.47 7.57 7.57 6.51 ...
##  $ changescalar_max_5mean  : num  3.76 3.76 2.82 2.82 1.63 ...
##  $ changescalar_mean_5mean : num  1.66 1.66 1.01 1.01 0.68 ...
##  $ Changevector_max_5mean  : num  4.5 4.5 3.69 3.69 2.29 ...
##  $ Changevector_mean_5mean : num  2.22 2.22 1.64 1.64 1.19 ...
```

```
##  $ Angle_max_5mean         : num  0.43 0.43 0.372 0.372 0.254 ...
##  $ Angle_mean_5mean        : num  0.176 0.176 0.141 0.141 0.131 ...
##  $ Hz_max_5mean            : num  109.4 109.4 94.6 94.6 64.7 ...
##  $ Hz_mean_5mean           : num  44.7 44.7 36 36 33.4 ...
##  $ Events._10mean          : int  288 288 315 315 238 238 261 261 219 219
...
##  $ Duration.ms._10mean     : num  3.89 3.89 7.71 7.71 7.42 ...
##  $ scalar_max_10mean       : num  15.5 15.5 16.4 16.4 15.6 ...
##  $ scalar_mean_10mean      : num  13.5 13.5 13.7 13.7 13.2 ...
##  $ changescalar_max_10mean : num  6.56 6.56 4.93 4.93 4 ...
##  $ changescalar_mean_10mean: num  3.25 3.25 1.97 1.97 1.66 ...
##  $ Changevector_max_10mean : num  8.03 8.03 6.51 6.51 5.89 ...
##  $ Changevector_mean_10mean: num  4.54 4.54 3.34 3.34 2.91 ...
##  $ Angle_max_10mean        : num  0.472 0.472 0.405 0.405 0.349 ...
##  $ Angle_mean_10mean       : num  0.222 0.222 0.175 0.175 0.158 ...
##  $ Hz_max_10mean           : num  120.2 120.2 103.1 103.1 88.9 ...
##  $ Hz_mean_10mean          : num  56.7 56.7 44.6 44.6 40.3 ...
##  $ Events_15mean           : int  144 144 183 183 111 111 163 163 129 129
...
##  $ Duration.ms._15mean     : num  3.44 3.44 6.34 6.34 6.92 ...
##  $ scalar_max_15mean       : num  19.3 19.3 19.9 19.9 19.7 ...
##  $ scalar_mean_15mean      : num  17.9 17.9 17.8 17.8 17.6 ...
##  $ changescalar_max_15mean : num  8.2 8.2 6.27 6.27 5.29 ...
##  $ changescalar_mean_15mean: num  4.21 4.21 2.42 2.42 2.06 ...
##  $ Changevector_max_15mean : num  10.15 10.15 8.77 8.77 8.64 ...
##  $ Changevector_mean_15mean: num  6.19 6.19 4.49 4.49 4.18 ...
##  $ Angle_max_15mean        : num  0.473 0.473 0.458 0.458 0.444 ...
##  $ Angle_mean_15mean       : num  0.264 0.264 0.204 0.204 0.196 ...
##  $ Hz_max_15mean           : num  121 121 117 117 113 ...
##  $ Hz_mean_15mean          : num  67.3 67.3 52 52 49.9 ...
##  $ Events_20mean           : int  57 57 75 75 42 42 97 97 71 71 ...
##  $ Duration.ms._20mean     : num  2.59 2.59 5.25 5.25 6 ...
##  $ scalar_max_20mean       : num  22.8 22.8 23.7 23.7 24.3 ...
##  $ scalar_mean_20mean      : num  22 22 22.3 22.3 22.6 ...
##  $ changescalar_max_20mean : num  7.5 7.5 6.19 6.19 6 ...
##   [list output truncated]
```

```
summary(fm28)
```

```
## Linear mixed-effects model fit by REML
##   Data: tab
##         AIC       BIC   logLik
##   -6.894157 -2.171962 8.447078
##
## Random effects:
##  Formula: ~1 | Replicate
##         (Intercept)  Residual
## StdDev: 1.135568e-06 0.1235004
##
## Fixed effects:  MSV ~ Irradiation + Duration.ms._15mean
```

```
##                              Value  Std.Error DF   t-value p-value
## (Intercept)          0.7494934 0.10532507 14  7.116002  0.0000
## Irradiationyes      -0.0719857 0.05266076 14 -1.366970  0.1932
## Duration.ms._15mean -0.0412491 0.01785618 14 -2.310072  0.0366
##  Correlation:
##                     (Intr) Irrdtn
## Irradiationyes      -0.250
## Duration.ms._15mean -0.935  0.000
##
## Standardized Within-Group Residuals:
##         Min          Q1         Med          Q3         Max
## -1.32931727 -0.74322987 -0.08387532  0.66845220  1.73655234
##
## Number of Observations: 22
## Number of Groups: 6

plot(MSV ~ fitted(fm28), data = tab)
abline(lm(MSV ~ fitted(fm28), data = tab), col = "red")
```

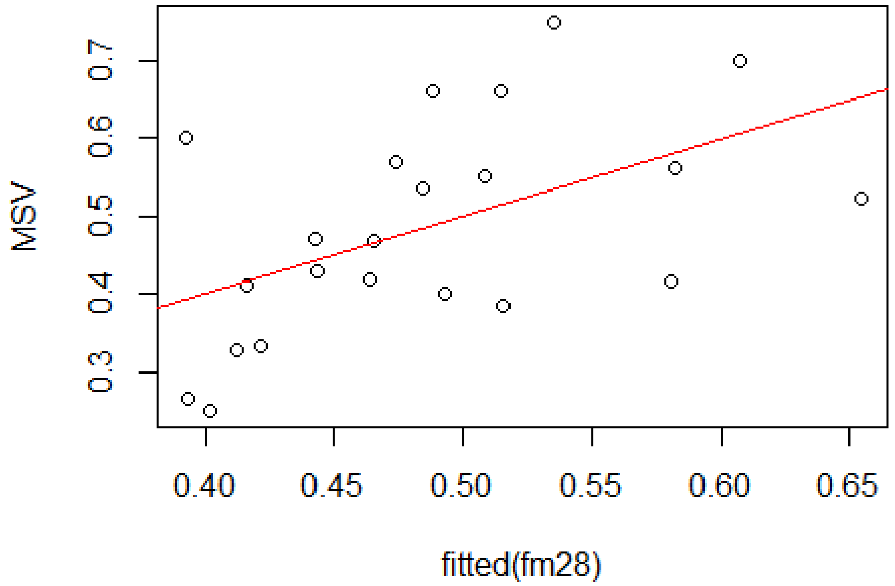


```
cor.test(tab$MSV,fitted(fm28))

##
##  Pearson's product-moment correlation
##
## data:  tab$MSV and fitted(fm28)
## t = 2.754, df = 20, p-value = 0.01224
## alternative hypothesis: true correlation is not equal to 0
```

```
## 95 percent confidence interval:
##  0.1319083 0.7746991
## sample estimates:
##       cor
## 0.5243555
```

```
summary(lm(fitted(fm28)~(tab$MSV)))$r.squared
```

```
## [1] 0.2749486
```

ALL ANALYSIS OF SUPPORTING INFORMATION

Analysis of the statistical relationships of temperature and humidity in/between 22 and 29 day old pupae

```
###### Correlation of temperature and humidity at age 22, 29
tab2 <- read.csv("Temp_RH_22.csv")
head(tab2)
```

```
##   Environmental_.variable_values Age Humidity Temperature
## 1                            Max  22 59.06000    28.28760
## 2                            Min  22 41.32000    17.76260
## 3                        Average  22 49.26442    22.35418
```

```
plot(tab2$Humidity,tab2$Temperature,xlab = "Humidity", ylab = " Temperature
(%)")
```

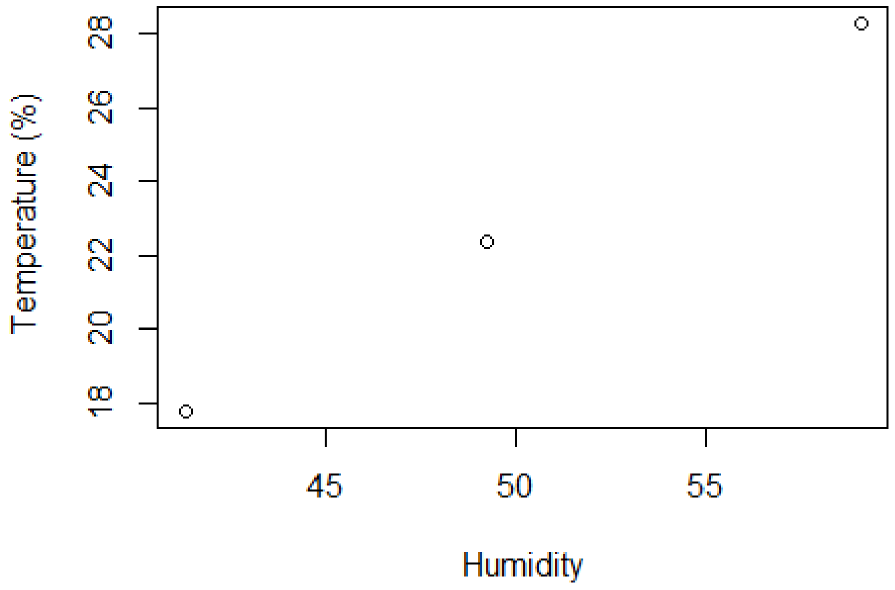


```
cor.test(tab2$Humidity,tab2$Temperature, method = "pearson")
```

```
##
##  Pearson's product-moment correlation
##
## data:  tab2$Humidity and tab2$Temperature
## t = 75.182, df = 1, p-value = 0.008467
## alternative hypothesis: true correlation is not equal to 0
## sample estimates:
##       cor
## 0.9999116
```

```
tab2 <- read.csv("Temp_RH_29_2.csv")
head(tab2)
```

```
##   Environmental_.variable_values Age Humidity Temperature
## 1                            Max  29 78.37667    25.56267
## 2                            Min  29 52.42833     6.50000
## 3                        Average  29 73.79146    13.13604
```

```
plot(tab2$Humidity,tab2$Temperature,xlab = "Humidity", ylab = " Temperature
(%)")
```

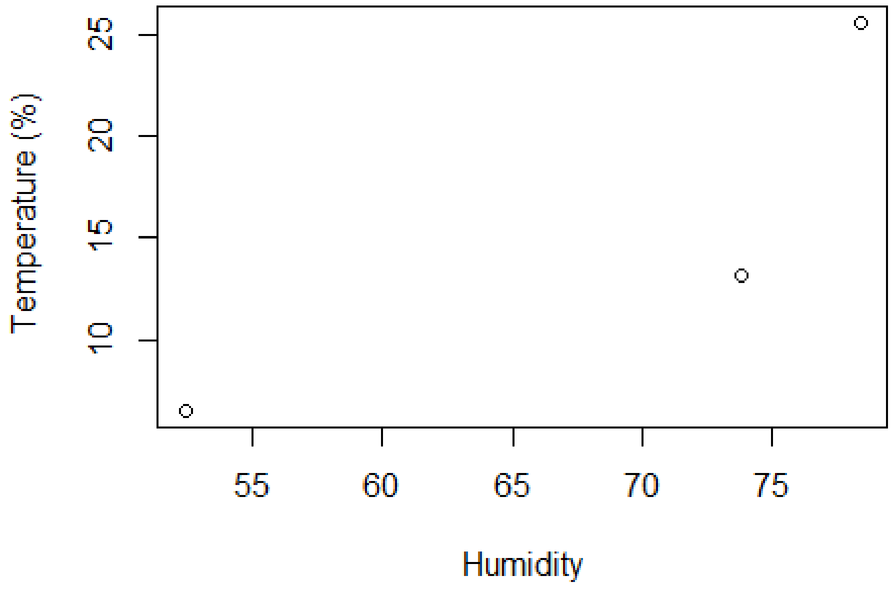


```
cor.test(tab2$Humidity,tab2$Temperature, method = "pearson")
```

```
##
##  Pearson's product-moment correlation
##
## data:  tab2$Humidity and tab2$Temperature
## t = 1.7032, df = 1, p-value = 0.338
```

```
## alternative hypothesis: true correlation is not equal to 0
## sample estimates:
##       cor
## 0.8623514

########## differences of temperature betweeen ages

tab2 <- read.csv("Temp_humidity .csv")
head(tab2)

##   Replicate age     Treatments Max..RH. Min.RH. Average.RH. Max..T. Min.T.
## 1         1  22 Shipped-110Gy     47.08   37.25    41.69370  23.500 15.125
## 2         2  22 Shipped-110Gy     60.14   44.11    53.54735  27.500 17.813
## 3         3  22 Shipped-110Gy     62.61   47.03    53.24609  29.750 19.000
## 4         4  22 Shipped-110Gy     68.10   43.58    50.01517  32.000 19.000
## 5         5  22 Shipped-110Gy     57.37   34.63    47.81979  28.688 17.875
## 6         1  29 Shipped-110Gy     74.59   66.48    72.32000  19.250  6.250
##   Average..T.
## 1    19.72786
## 2    21.79029
## 3    23.28835
## 4    24.58312
## 5    22.38131
## 6    10.22000

boxplot(tab2$Max..T.~ tab2$age ,xlab = "Age", ylab = " Temperature (°C) ")
```

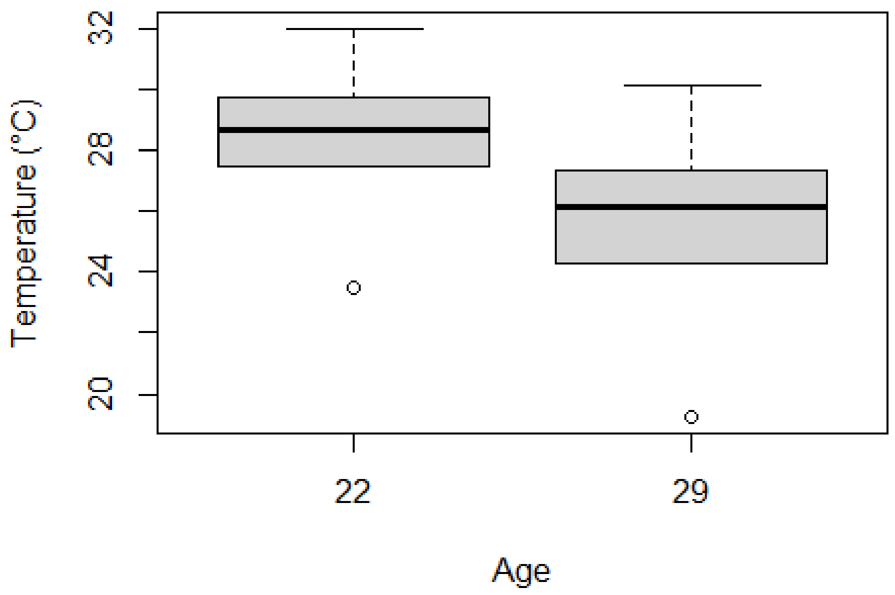

```r
fm2 <- lm (Max..T.~ age, data = tab2)
summary(fm2)
```

```
##
## Call:
## lm(formula = Max..T. ~ age, data = tab2)
##
## Residuals:
##     Min      1Q  Median      3Q     Max
## -6.3127 -1.0186  0.4004  1.6873  4.5623
##
## Coefficients:
##             Estimate Std. Error t value Pr(>|t|)
## (Intercept)  36.8517     7.7965   4.727  0.00108 **
## age          -0.3893     0.2993  -1.301  0.22565
## ---
## Signif. codes:  0 '***' 0.001 '**' 0.01 '*' 0.05 '.' 0.1 ' ' 1
##
## Residual standard error: 3.46 on 9 degrees of freedom
## Multiple R-squared:  0.1583, Adjusted R-squared:  0.06472
## F-statistic: 1.692 on 1 and 9 DF,  p-value: 0.2256
```

```r
boxplot(tab2$Min.T.~ tab2$age ,xlab = "Age", ylab = " Temperature (°C)")
```

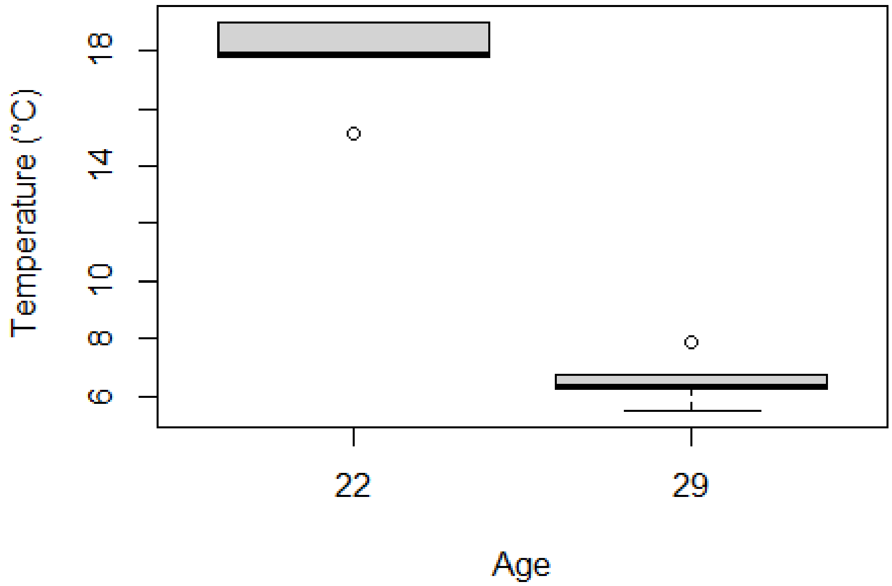


```r
fm2 <- lm (Min.T.~ age, data = tab2)
summary(fm2)
```

```
##
## Call:
## lm(formula = Min.T. ~ age, data = tab2)
##
## Residuals:
##     Min      1Q  Median      3Q     Max
## -2.6376 -0.2500  0.0504  0.7437  1.3750
##
## Coefficients:
##             Estimate Std. Error t value Pr(>|t|)
## (Intercept)  53.1593     2.7219   19.53 1.12e-08 ***
## age          -1.6089     0.1045  -15.40 8.97e-08 ***
## ---
## Signif. codes:  0 '***' 0.001 '**' 0.01 '*' 0.05 '.' 0.1 ' ' 1
##
## Residual standard error: 1.208 on 9 degrees of freedom
## Multiple R-squared:  0.9634, Adjusted R-squared:  0.9594
## F-statistic: 237.2 on 1 and 9 DF,  p-value: 8.974e-08
```

```
boxplot(tab2$Average..T.~ tab2$age ,xlab = "Age", ylab = " Temperature (°C)")
```

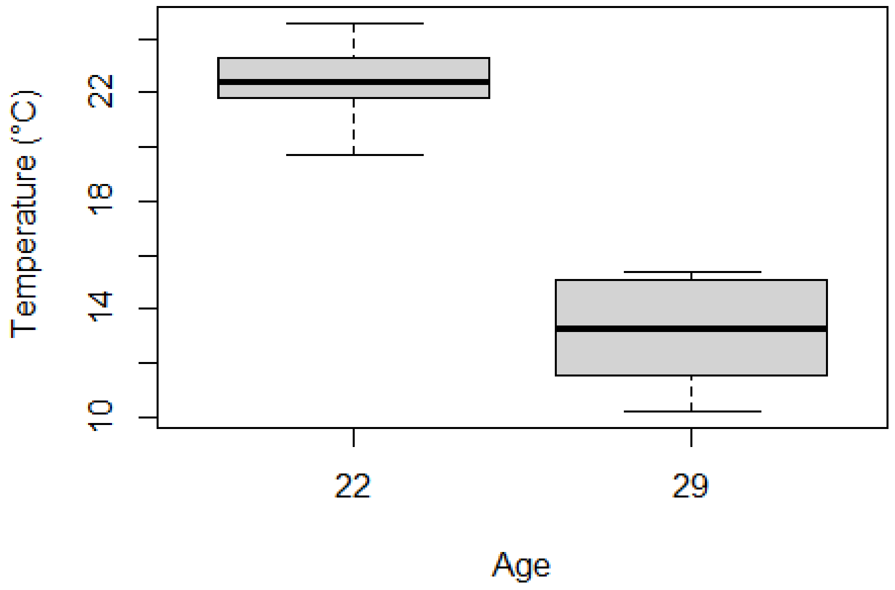


```
fm2 <- lm (Average..T.~ age, data = tab2)
summary(fm2)
```

```
##
## Call:
## lm(formula = Average..T. ~ age, data = tab2)
```

```
##
## Residuals:
##      Min       1Q   Median       3Q      Max
## -2.91604 -1.37423  0.02712  1.72161  2.22893
##
## Coefficients:
##             Estimate Std. Error t value Pr(>|t|)
## (Intercept)  51.3255     4.5313  11.327 1.26e-06 ***
## age          -1.3169     0.1739  -7.571 3.43e-05 ***
## ---
## Signif. codes:  0 '***' 0.001 '**' 0.01 '*' 0.05 '.' 0.1 ' ' 1
##
## Residual standard error: 2.011 on 9 degrees of freedom
## Multiple R-squared:  0.8643, Adjusted R-squared:  0.8492
## F-statistic: 57.32 on 1 and 9 DF,  p-value: 3.428e-05
```

```
################differences of humidity between ages

boxplot(tab2$Max..RH.~ tab2$age ,xlab = "Age", ylab = " Relative Humidity
(RH)")
```

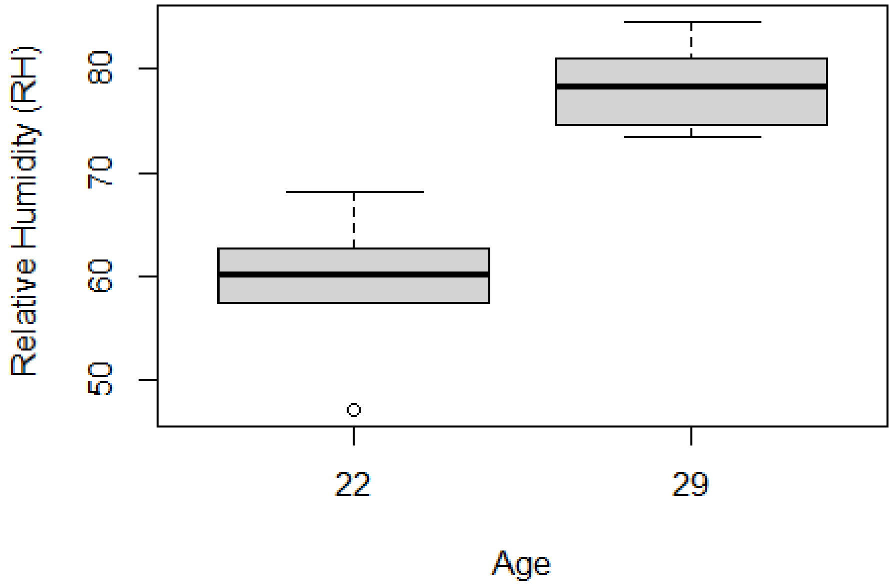


```
fm2 <- lm (Max..RH.~ age, data = tab2)
summary(fm2)

##
## Call:
## lm(formula = Max..RH. ~ age, data = tab2)
```

```
##
## Residuals:
##      Min       1Q   Median       3Q      Max
## -11.9800  -2.7383   0.0433   3.1217   9.0400
##
## Coefficients:
##             Estimate Std. Error t value Pr(>|t|)
## (Intercept)  -1.6495    13.5583  -0.122 0.905840
## age           2.7595     0.5204   5.302 0.000492 ***
## ---
## Signif. codes:  0 '***' 0.001 '**' 0.01 '*' 0.05 '.' 0.1 ' ' 1
##
## Residual standard error: 6.016 on 9 degrees of freedom
## Multiple R-squared:  0.7575, Adjusted R-squared:  0.7306
## F-statistic: 28.12 on 1 and 9 DF,  p-value: 0.0004923
```

```
boxplot(tab2$Min.RH.~ tab2$age ,xlab = "Age", ylab = " Relative Humidity
(RH)")
```

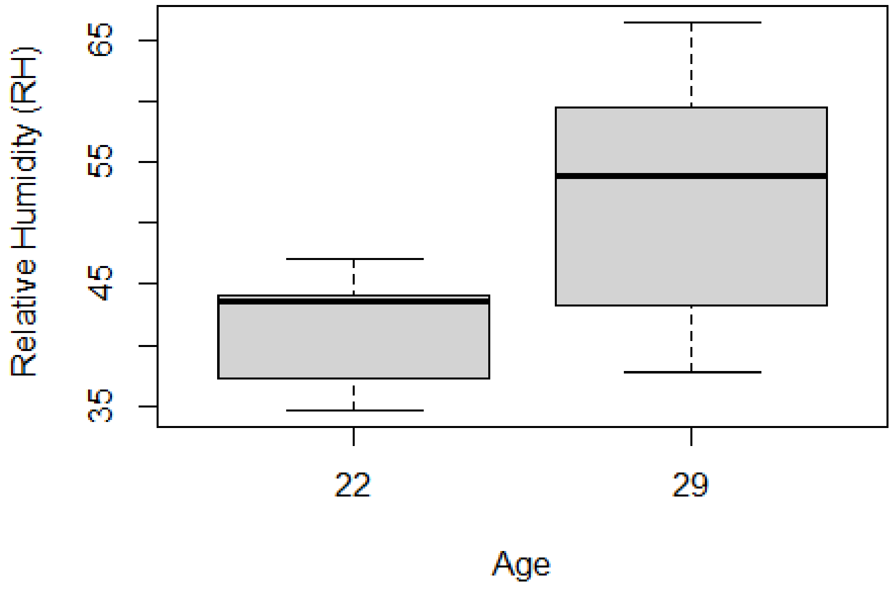


```
fm2 <- lm (Min.RH.~ age, data = tab2)
summary(fm2)
```

```
##
## Call:
## lm(formula = Min.RH. ~ age, data = tab2)
##
## Residuals:
```

```
##     Min      1Q  Median      3Q     Max 
## -14.708  -5.380   2.260   5.346  14.052 
## 
## Coefficients:
##             Estimate Std. Error t value Pr(>|t|)  
## (Intercept)   6.4081    19.6662   0.326   0.7520  
## age           1.5869     0.7549   2.102   0.0649 .
## ---
## Signif. codes:  0 '***' 0.001 '**' 0.01 '*' 0.05 '.' 0.1 ' ' 1
## 
## Residual standard error: 8.726 on 9 degrees of freedom
## Multiple R-squared:  0.3293, Adjusted R-squared:  0.2548 
## F-statistic: 4.419 on 1 and 9 DF,  p-value: 0.06488
```

```
boxplot(tab2$Average.RH.~ tab2$age ,xlab = "Age", ylab = "Relative Humidity (RH)")
```

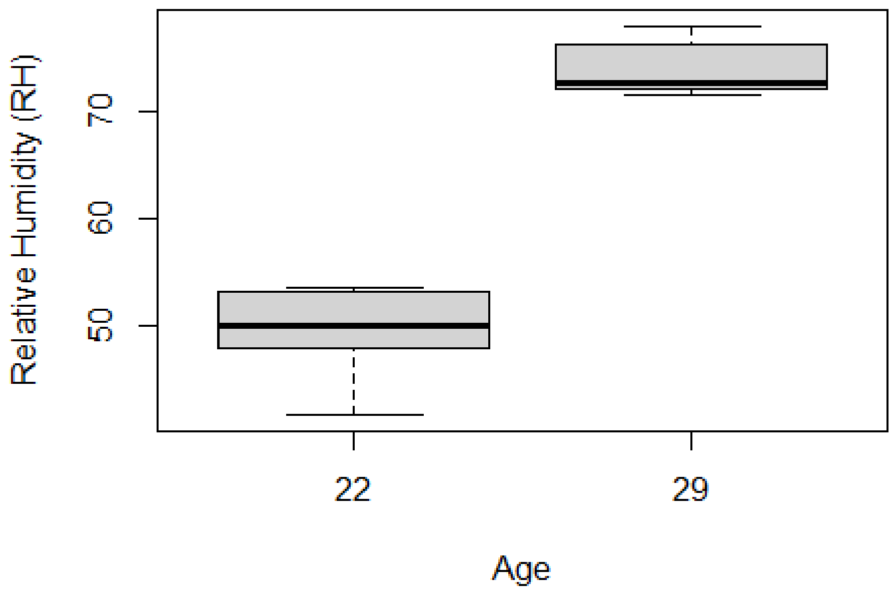


```
fm2 <- lm (Average.RH.~ age, data = tab2)
summary(fm2)
```

```
## 
## Call:
## lm(formula = Average.RH. ~ age, data = tab2)
## 
## Residuals:
##     Min      1Q  Median      3Q     Max 
## -7.5707 -1.6550 -0.9199  3.1798  4.2829
```

```
##
## Coefficients:
##             Estimate Std. Error t value Pr(>|t|)
## (Intercept) -27.8206     8.5051  -3.271  0.00967 **
## age           3.5039     0.3265  10.733 1.98e-06 ***
## ---
## Signif. codes:  0 '***' 0.001 '**' 0.01 '*' 0.05 '.' 0.1 ' ' 1
##
## Residual standard error: 3.774 on 9 degrees of freedom
## Multiple R-squared:  0.9275, Adjusted R-squared:  0.9195
## F-statistic: 115.2 on 1 and 9 DF,  p-value: 1.98e-06
```

```
##########################################################################

data_4<- read.csv("Temp_humidity_2.csv")
head(data_4)
```

```
##   age Environ_values environ
## 1  22          47.08 Max _RH
## 2  22          60.14 Max _RH
## 3  22          62.61 Max _RH
## 4  22          68.10 Max _RH
## 5  22          57.37 Max _RH
## 6  29          74.59 Max _RH
```

```
#### facet wrap with Pupal_age
ggplot(data_4,aes(x=environ ,y=Environ_values,fill=factor(age)))+
  geom_boxplot(alpha=0.3) + geom_jitter(width=0.1,alpha=0.2)+
  labs(fill = "age") +
  geom_point(position=position_jitterdodge(),alpha=0.3) + facet_wrap(~age) +
  theme_bw(base_size = 16)
```

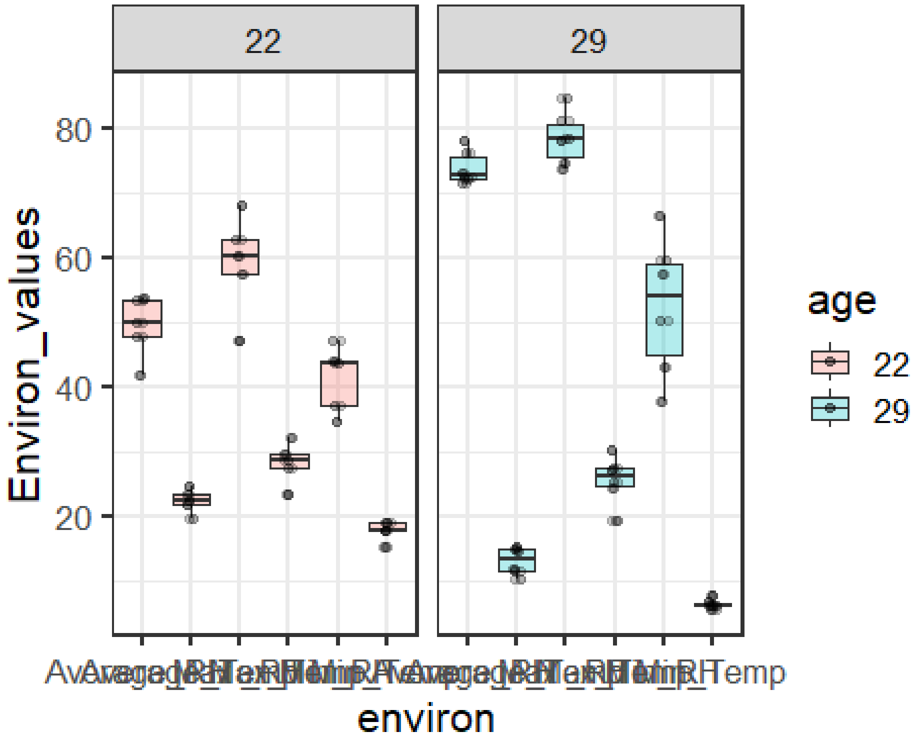


```
#### facet wrap with Environ measure
ggplot(data_4,aes(x=factor(age),y=Environ_values,fill=environ))+
  geom_boxplot(alpha=0.3) + geom_jitter(width=0.1,alpha=0.2)+
  labs(fill = "environ") +
  geom_point(position=position_jitterdodge(),alpha=0.3) +
facet_wrap(~environ) +
  theme_bw(base_size = 16)
```

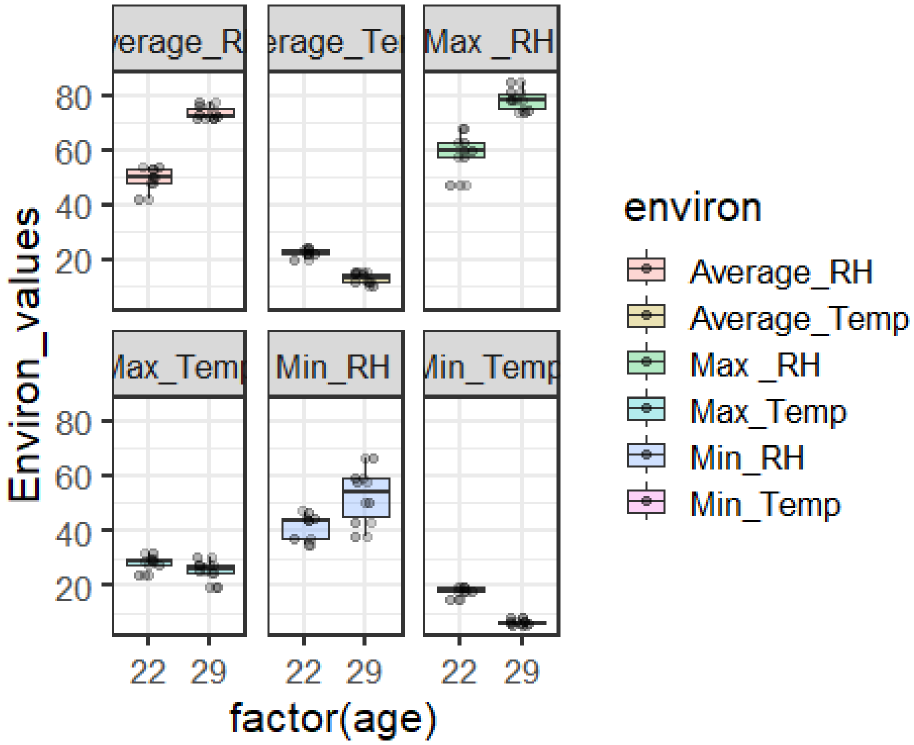

SUPPORTING FIGURES S2-S8

ANALYSIS OF THE IMPACT OF IRRADIATION AND TRASPORTATION(COMBINED DATA FROM 22 AND 29 DAY OLD PUPAE)

Figure S2a and S3a: Emergence rate

```
tabS2a_S3a=read.csv("Figure S2a and S3a.csv")
head(tabS2a_S3a)

##   Replicate Pupal_age      Treatments emerged unemerged shipped irradiated
## 1        R1        29   Shipped-110Gy      81        36       1          1
## 2        R1        29     Shipped-0Gy     102        61       1          0
## 3        R1        29 Unshipped-110Gy      85        36       0          1
## 4        R1        29   Unshipped-0Gy     116        47       0          0
## 5        R2        29   Shipped-110Gy      85        81       1          1
## 6        R2        29     Shipped-0Gy     120        63       1          0
##   chilled   Treatment_age         Treatment
## 1       1  Ship-110Gy _22  4_Shipped-110Gy
## 2       1     Ship-0Gy_22      2_Shipped-0Gy
## 3       1 Unship-110Gy_22 3_Unshipped-110Gy
## 4       1   Unship-0Gy_22   1_Unshipped-0Gy
## 5       1  Ship-110Gy _22  4_Shipped-110Gy
## 6       1     Ship-0Gy_22      2_Shipped-0Gy

Emerg_rate<- tabS2a_S3a$emerged / (tabS2a_S3a$unemerged+ tabS2a_S3a$emerged)
```

```
###Figure S2a

tiff("Figure S2a.tiff", width = 7.5, height =4 , units = 'in', compression =
'lzw',res = 300)
boxplot(Emerg_rate~Treatments,data = tabS2a_S3a,
        ylab = expression(bold("Emergence rate (22 and 29 day pupae)")),
        xlab = expression(bold("Treatments")))
dev.off()

## png
##   2

### Figure S3a

tiff("Figure S3a.tiff", width = 4, height =4 , units = 'in', compression =
'lzw',res = 300)
boxplot(Emerg_rate~Pupal_age,data = tabS2a_S3a,
        ylab = expression(bold("Emergence rate (22 and 29 day pupae)")),
        xlab = expression(bold("Pupal age")))

dev.off()

## png
##   2

###Significance
###Figure s2a
fmS2a <- glmer(cbind(emerged, unemerged) ~ Treatment +(1|Replicate), family =
binomial, data = tabS2a_S3a)
summary(fmS2a )

## Generalized linear mixed model fit by maximum likelihood (Laplace
##   Approximation) [glmerMod]
##  Family: binomial  ( logit )
## Formula: cbind(emerged, unemerged) ~ Treatment + (1 | Replicate)
##    Data: tabS2a_S3a
##
##      AIC      BIC   logLik deviance df.resid
##    647.3    656.3   -318.7    637.3       39
##
## Scaled residuals:
##     Min      1Q  Median      3Q     Max
## -7.3832 -2.7139  0.0433  2.2551  5.5044
##
## Random effects:
##  Groups    Name        Variance Std.Dev.
##  Replicate (Intercept) 0.1537   0.3921
## Number of obs: 44, groups:  Replicate, 6
##
## Fixed effects:
##                            Estimate Std. Error z value Pr(>|z|)
```

```
## (Intercept)                  1.10666    0.16833   6.574 4.89e-11 ***
## Treatment2_Shipped-0Gy      -0.30987    0.06865  -4.514 6.36e-06 ***
## Treatment3_Unshipped-110Gy -0.50653    0.06905  -7.336 2.21e-13 ***
## Treatment4_Shipped-110Gy    -0.48523    0.06897  -7.036 1.98e-12 ***
## ---
## Signif. codes:  0 '***' 0.001 '**' 0.01 '*' 0.05 '.' 0.1 ' ' 1
##
## Correlation of Fixed Effects:
##              (Intr) T2_S-0 T3_U-1
## Trtmn2_S-0G -0.217
## Trt3_U-110G -0.218  0.529
## Trt4_S-110G -0.216  0.529  0.527
```

```
###Figure s3a
fmS3a <- glmer(cbind(emerged, unemerged) ~ Pupal_age +(1|Replicate), family =
binomial, data = tabS2a_S3a)
summary(fmS3a)
```

```
## Generalized linear mixed model fit by maximum likelihood (Laplace
##   Approximation) [glmerMod]
##  Family: binomial  ( logit )
## Formula: cbind(emerged, unemerged) ~ Pupal_age + (1 | Replicate)
##    Data: tabS2a_S3a
##
##      AIC      BIC   logLik deviance df.resid
##    652.3    657.6   -323.1    646.3       41
##
## Scaled residuals:
##    Min     1Q Median     3Q    Max
## -6.194 -2.162  0.466  2.349  4.397
##
## Random effects:
##  Groups    Name        Variance Std.Dev.
##  Replicate (Intercept) 0.1779   0.4218
## Number of obs: 44, groups:  Replicate, 6
##
## Fixed effects:
##              Estimate Std. Error z value Pr(>|z|)
## (Intercept)  2.196021   0.252327   8.703  < 2e-16 ***
## Pupal_age   -0.055409   0.007104  -7.799 6.22e-15 ***
## ---
## Signif. codes:  0 '***' 0.001 '**' 0.01 '*' 0.05 '.' 0.1 ' ' 1
##
## Correlation of Fixed Effects:
##           (Intr)
## Pupal_age -0.723
```

```
###Differences among treatments- non-parametric test
kruskal.test(Emerg_rate~Treatment, tabS2a_S3a)
```

```
##
##  Kruskal-Wallis rank sum test
##
## data:  Emerg_rate by Treatment
## Kruskal-Wallis chi-squared = 4.089, df = 3, p-value = 0.252

#############################################################################
#######################
```

Figure S2b and S3b: Flight propensity

```
tabS2b_S3b= read.csv("Figure S2b and S3b.csv")
head(tabS2b_S3b)

##   Replicate Pupal_age      Treatments out in. shipped irradiated chilled
## 1        R1        29  Shipped-110Gy   31  33       1          1       1
## 2        R1        29    Shipped-0Gy   26  28       1          0       1
## 3        R1        29 Unshipped-110Gy  30  42       0          1       1
## 4        R1        29   Unshipped-0Gy  21   9       0          0       1
## 5        R2        29  Shipped-110Gy   23  24       1          1       1
## 6        R2        29    Shipped-0Gy    9  30       1          0       1
##     Treatment_age          Treatment
## 1  Ship-110Gy _22  4_Shipped-110Gy
## 2    Ship-0Gy_22      2_Shipped-0Gy
## 3 Unship-110Gy_22 3_Unshipped-110Gy
## 4   Unship-0Gy_22    1_Unshipped-0Gy
## 5  Ship-110Gy _22  4_Shipped-110Gy
## 6    Ship-0Gy_22      2_Shipped-0Gy

Flight_rate<- tabS2b_S3b$out / (tabS2b_S3b$in.+ tabS2b_S3b$out)
tabS2b_S3b$Pupal_age<- as.factor(tabS2b_S3b$Pupal_age)

### Figure S2b

tiff("Figure S2b.tiff", width = 7.5, height =4 , units = 'in', compression =
'lzw',res = 300)
boxplot(Flight_rate ~ tabS2b_S3b$Treatments,xlab = "Treatments", ylab =
"flight propensity (22 and 29 day pupae)")
boxplot(Emerg_rate~Treatments,data = tabS2b_S3b,
        ylab = expression(bold("Flight propensity (22 and 29 day)")),
        xlab = expression(bold("Treatments")))

dev.off()

## png
##   2

###Figure S3b

tiff("Figure S3b.tiff", width = 4, height =4 , units = 'in', compression =
'lzw',res = 300)
```

```
boxplot(Flight_rate ~ tabS2b_S3b$Pupal_age,xlab = "Pupal age", ylab = "flight
propensity (Pupal_age)")
boxplot(Emerg_rate~Pupal_age,data = tabS2b_S3b,
        ylab = expression(bold("Flight propensity (22 and 29 day)")),
        xlab = expression(bold("Pupal age")))


dev.off()

## png
##   2

###Significance
##Figure S2b
fmS2b <- glmer(cbind(out, in.) ~ Treatment +(1|Replicate), family = binomial,
data = tabS2b_S3b)
summary(fmS2b)

## Generalized linear mixed model fit by maximum likelihood (Laplace
##   Approximation) [glmerMod]
##  Family: binomial  ( logit )
## Formula: cbind(out, in.) ~ Treatment + (1 | Replicate)
##    Data: tabS2b_S3b
##
##      AIC      BIC   logLik deviance df.resid
##    596.8    605.7   -293.4    586.8       39
##
## Scaled residuals:
##      Min       1Q   Median       3Q      Max
## -10.1211  -2.3898   0.3972   1.8068   5.9453
##
## Random effects:
##  Groups    Name        Variance Std.Dev.
##  Replicate (Intercept) 0.5523   0.7432
## Number of obs: 44, groups:  Replicate, 6
##
## Fixed effects:
##                            Estimate Std. Error z value Pr(>|z|)
## (Intercept)                  2.2107     0.3291   6.717 1.85e-11 ***
## Treatment2_Shipped-0Gy      -0.7844     0.1705  -4.602 4.18e-06 ***
## Treatment3_Unshipped-110Gy  -0.7852     0.1594  -4.927 8.34e-07 ***
## Treatment4_Shipped-110Gy    -0.8828     0.1678  -5.261 1.43e-07 ***
## ---
## Signif. codes:  0 '***' 0.001 '**' 0.01 '*' 0.05 '.' 0.1 ' ' 1
##
## Correlation of Fixed Effects:
##             (Intr) T2_S-0 T3_U-1
## Trtmn2_S-0G -0.286
## Trt3_U-110G -0.295  0.573
## Trt4_S-110G -0.290  0.559  0.583
```

```
###Figure S3b
fmS3b <- glmer(cbind(out, in.) ~ Pupal_age +(1|Replicate), family = binomial,
data = tabS2b_S3b)
summary(fmS3b)

## Generalized linear mixed model fit by maximum likelihood (Laplace
##   Approximation) [glmerMod]
##  Family: binomial  ( logit )
## Formula: cbind(out, in.) ~ Pupal_age + (1 | Replicate)
##    Data: tabS2b_S3b
##
##      AIC      BIC   logLik deviance df.resid
##    304.7    310.1   -149.4    298.7       41
##
## Scaled residuals:
##     Min      1Q  Median      3Q     Max
## -3.2524 -1.5251  0.0579  1.2333  2.9294
##
## Random effects:
##  Groups    Name        Variance Std.Dev.
##  Replicate (Intercept) 0.4185   0.6469
## Number of obs: 44, groups:  Replicate, 6
##
## Fixed effects:
##             Estimate Std. Error z value Pr(>|z|)
## (Intercept)   3.0714     0.2942   10.44   <2e-16 ***
## Pupal_age29  -2.3738     0.1488  -15.96   <2e-16 ***
## ---
## Signif. codes:  0 '***' 0.001 '**' 0.01 '*' 0.05 '.' 0.1 ' ' 1
##
## Correlation of Fixed Effects:
##             (Intr)
## Pupal_age29 -0.387

######## non-parametric

###Differences among treatments- non-parametric test
kruskal.test(Flight_rate~Treatment, tabS2b_S3b)

##
##  Kruskal-Wallis rank sum test
##
## data:  Flight_rate by Treatment
## Kruskal-Wallis chi-squared = 0.62864, df = 3, p-value = 0.8898
```

Figure S2c and S3c: Mating ability

```
tabS2c_S3c= read.csv("Figure S2c and S3c.csv")
head(tabS2c_S3c)
```

```
##   Replicate Pupal_age      Treatments unformed_pairs pairs_formed
intial_pairs
## 1        R1        29  Shipped-110Gy              4           26
30
## 2        R1        29    Shipped-0Gy              9           21
30
## 3        R1        29 Unshipped-110Gy            4           26
30
## 4        R1        29   Unshipped-0Gy             5           25
30
## 5        R2        29  Shipped-110Gy              6           24
30
## 6        R2        29    Shipped-0Gy             14           16
30
##   shipped irradiated chilled   Treatment_age         Treatment
## 1       1          1       1  Ship-110Gy _22  4_Shipped-110Gy
## 2       1          0       1    Ship-0Gy_22      2_Shipped-0Gy
## 3       0          1       1 Unship-110Gy_22 3_Unshipped-110Gy
## 4       0          0       1   Unship-0Gy_22    1_Unshipped-0Gy
## 5       1          1       1  Ship-110Gy _22  4_Shipped-110Gy
## 6       1          0       1    Ship-0Gy_22      2_Shipped-0Gy
```

```
mating_prop<- tabS2c_S3c$pairs_formed  / (tabS2c_S3c$unformed_pairs+
tabS2c_S3c$pairs_formed )
tabS2c_S3c$Pupal_age<- as.factor(tabS2c_S3c$Pupal_age)

### Figure S2c

tiff("Figure S2c.tiff", width = 7.5, height =4 , units = 'in', compression =
'lzw',res = 300)
boxplot(mating_prop ~ tabS2c_S3c$Treatments,xlab = "Treatments", ylab =
"Mating ability")
boxplot(Emerg_rate~Treatments,data = tabS2c_S3c,
        ylab = expression(bold("Mating ability (22 and 29 day)")),
        xlab = expression(bold("Treatments")))

dev.off()

## png
##   2

### Figure S3c

tiff("Figure S3c.tiff", width = 4, height =4 , units = 'in', compression =
'lzw',res = 300)
boxplot(mating_prop ~ tabS2c_S3c$Pupal_age,xlab = "Pupal age", ylab = "Mating
ability")
boxplot(Emerg_rate~Pupal_age,data = tabS2c_S3c,
        ylab = expression(bold("Mating ability (22 and 29 day)")),
        xlab = expression(bold("Pupal age")))
```

```
dev.off()

## png
##   2

###Significance

###Figure S2c
fmS2c <- glmer(cbind(unformed_pairs, pairs_formed)~ Treatment +(1|Replicate),
family = binomial, data = tabS2c_S3c)
summary(fmS2c)

## Generalized linear mixed model fit by maximum likelihood (Laplace
##   Approximation) [glmerMod]
##  Family: binomial  ( logit )
## Formula: cbind(unformed_pairs, pairs_formed) ~ Treatment + (1 | Replicate)
##    Data: tabS2c_S3c
##
##      AIC      BIC   logLik deviance df.resid
##    421.3    430.3   -205.7    411.3       39
##
## Scaled residuals:
##     Min      1Q  Median      3Q     Max
## -4.1602 -1.5384 -0.8928  1.5184  6.4040
##
## Random effects:
##  Groups    Name        Variance Std.Dev.
##  Replicate (Intercept) 0.1834   0.4282
## Number of obs: 44, groups:  Replicate, 6
##
## Fixed effects:
##                           Estimate Std. Error z value Pr(>|z|)
## (Intercept)                -1.6275     0.2281  -7.136 9.62e-13 ***
## Treatment2_Shipped-0Gy      0.5433     0.1874   2.898  0.00375 **
## Treatment3_Unshipped-110Gy   0.5886     0.1871   3.146  0.00165 **
## Treatment4_Shipped-110Gy    1.1524     0.1814   6.352 2.13e-10 ***
## ---
## Signif. codes:  0 '***' 0.001 '**' 0.01 '*' 0.05 '.' 0.1 ' ' 1
##
## Correlation of Fixed Effects:
##              (Intr) T2_S-0 T3_U-1
## Trtmn2_S-0G -0.472
## Trt3_U-110G -0.473  0.573
## Trt4_S-110G -0.494  0.592  0.592

###Figure S3c
fmS3c <- glmer(cbind(unformed_pairs, pairs_formed) ~ Pupal_age
+(1|Replicate), family = binomial, data = tabS2c_S3c)
summary(fmS3c)
```

```
## Generalized linear mixed model fit by maximum likelihood (Laplace
##   Approximation) [glmerMod]
##  Family: binomial  ( logit )
## Formula: cbind(unformed_pairs, pairs_formed) ~ Pupal_age + (1 | Replicate)
##    Data: tabS2c_S3c
##
##      AIC      BIC   logLik deviance df.resid
##    436.5    441.8   -215.2    430.5       41
##
## Scaled residuals:
##     Min      1Q  Median      3Q     Max
## -3.8470 -1.6701 -0.3359  0.9722  5.9324
##
## Random effects:
##  Groups    Name        Variance Std.Dev.
##  Replicate (Intercept) 0.09319  0.3053
## Number of obs: 44, groups:  Replicate, 6
##
## Fixed effects:
##             Estimate Std. Error z value Pr(>|z|)
## (Intercept)  -0.7069     0.1539  -4.592 4.39e-06 ***
## Pupal_age29  -0.6430     0.1322  -4.863 1.15e-06 ***
## ---
## Signif. codes:  0 '***' 0.001 '**' 0.01 '*' 0.05 '.' 0.1 ' ' 1
##
## Correlation of Fixed Effects:
##             (Intr)
## Pupal_age29 -0.385
```

```
###Differences among treatments- non-parametric test
kruskal.test(mating_prop~Treatment, tabS2c_S3c)
```

```
##
##  Kruskal-Wallis rank sum test
##
## data:  mating_prop by Treatment
## Kruskal-Wallis chi-squared = 3.9102, df = 3, p-value = 0.2713
```

Figure S2d and S3d: Insemination rate

```
tabS2d_S3d= read.csv("Figure S2d and S3d.csv")
head(tabS2d_S3d)
```

```
##   Replicate Pupal_age      Treatments Inseminated Empty  MSV shipped
irradiated
## 1        R1        22  Shipped-110Gy           8     0 0.75       1
1
## 2        R1        22    Shipped-0Gy          10     0 0.70       1
0
## 3        R1        22 Unshipped-110Gy          38     1 0.77       0
1
```

```
## 4         R1        22   Unshipped-0Gy          42      0 0.63       0
0
## 5         R2        22  Shipped-110Gy           23      4 0.41       1
1
## 6         R2        22    Shipped-0Gy           27      3 0.66       1
0
##   chilled   Treatment_age         Treatment
## 1       0  Ship-110Gy _22  4_Shipped-110Gy
## 2       0    Ship-0Gy_22      2_Shipped-0Gy
## 3       0 Unship-110Gy_22 3_Unshipped-110Gy
## 4       0   Unship-0Gy_22    1_Unshipped-0Gy
## 5       0  Ship-110Gy _22  4_Shipped-110Gy
## 6       0    Ship-0Gy_22      2_Shipped-0Gy

Insemination_rate<- tabS2d_S3d$Inseminated / (tabS2d_S3d$Empty+
tabS2d_S3d$Inseminated)
tabS2d_S3d$Pupal_age<- as.factor(tabS2d_S3d$Pupal_age)

### Figure S2d

tiff("Figure S2d.tiff", width = 7.5, height =4 , units = 'in', compression =
'lzw',res = 300)
boxplot(Emerg_rate~Treatments,data = tabS2d_S3d,
        ylab = expression(bold("Insemination rate (22 and 29 day)")),
        xlab = expression(bold("Treatments")))
dev.off()

## png
##   2

### Figure S3d

tiff("Figure S3d.tiff", width = 4, height =4 , units = 'in', compression =
'lzw',res = 300)
boxplot(Emerg_rate~Pupal_age,data = tabS2d_S3d,
        ylab = expression(bold("Insemination rate (22 and 29 day)")),
        xlab = expression(bold("Pupal age")))

dev.off()

## png
##   2

###Significance
###Figure S2d
fmS2d <- glmer(cbind(Inseminated,Empty) ~ Treatment +(1|Replicate), family =
binomial, data = tabS2d_S3d)

## boundary (singular) fit: see help('isSingular')

summary(fmS2d)
```

```
## Generalized linear mixed model fit by maximum likelihood (Laplace
##   Approximation) [glmerMod]
##  Family: binomial  ( logit )
## Formula: cbind(Inseminated, Empty) ~ Treatment + (1 | Replicate)
##    Data: tabS2d_S3d
##
##      AIC      BIC   logLik deviance df.resid
##    156.9    165.8    -73.4    146.9       39
##
## Scaled residuals:
##      Min       1Q   Median       3Q      Max
## -2.64472 -1.05628 -0.03329  0.95244  2.02837
##
## Random effects:
##  Groups    Name        Variance Std.Dev.
##  Replicate (Intercept) 0        0
## Number of obs: 44, groups:  Replicate, 7
##
## Fixed effects:
##                            Estimate Std. Error z value Pr(>|z|)
## (Intercept)                  2.3232     0.2139  10.862   <2e-16 ***
## Treatment2_Shipped-0Gy       0.3848     0.3418   1.126   0.2603
## Treatment3_Unshipped-110Gy   0.7355     0.3879   1.896   0.0579 .
## Treatment4_Shipped-110Gy    -0.1542     0.3185  -0.484   0.6284
## ---
## Signif. codes:  0 '***' 0.001 '**' 0.01 '*' 0.05 '.' 0.1 ' ' 1
##
## Correlation of Fixed Effects:
##             (Intr) T2_S-0 T3_U-1
## Trtmn2_S-0G -0.626
## Trt3_U-110G -0.551  0.345
## Trt4_S-110G -0.671  0.420  0.370
## optimizer (Nelder_Mead) convergence code: 0 (OK)
## boundary (singular) fit: see help('isSingular')
```

```
###Figure S3d
fmS3d <- glmer(cbind(Inseminated, Empty) ~ Pupal_age +(1|Replicate), family =
binomial, data = tabS2d_S3d)
summary(fmS3d)
```

```
## Generalized linear mixed model fit by maximum likelihood (Laplace
##   Approximation) [glmerMod]
##  Family: binomial  ( logit )
## Formula: cbind(Inseminated, Empty) ~ Pupal_age + (1 | Replicate)
##    Data: tabS2d_S3d
##
##      AIC      BIC   logLik deviance df.resid
##    148.3    153.7    -71.2    142.3       41
##
## Scaled residuals:
```

```
##     Min      1Q  Median      3Q     Max
## -2.4537 -1.0633  0.2030  0.8258  1.5537
##
## Random effects:
##  Groups    Name        Variance Std.Dev.
##  Replicate (Intercept) 0.004639 0.06811
## Number of obs: 44, groups:  Replicate, 7
##
## Fixed effects:
##             Estimate Std. Error z value Pr(>|z|)
## (Intercept)   3.0274     0.2338  12.951  < 2e-16 ***
## Pupal_age29  -0.8672     0.2744  -3.161  0.00157 **
## ---
## Signif. codes:  0 '***' 0.001 '**' 0.01 '*' 0.05 '.' 0.1 ' ' 1
##
## Correlation of Fixed Effects:
##             (Intr)
## Pupal_age29 -0.819
```

```
###Differences among treatments- non-parametric test

kruskal.test(Insemination_rate~Treatment, tabS2d_S3d)
```

```
##
##  Kruskal-Wallis rank sum test
##
## data:  Insemination_rate by Treatment
## Kruskal-Wallis chi-squared = 2.7584, df = 3, p-value = 0.4304
```

Figure S2e and S3e:Mean spermathecal value (MSV)

```
tabS2e_S3e= read.csv("Figure S2e and S3e.csv")
head(tabS2e_S3e)
```

```
##   Replicate Pupal_age pairs_no      Treatments       MSV shipped
irradiated
## 1        R1        29       26  Shipped-110Gy  0.3333333       1
1
## 2        R1        29       21    Shipped-0Gy  0.4000000       1
0
## 3        R1        29       26 Unshipped-110Gy 0.6406250       0
1
## 4        R1        29       25   Unshipped-0Gy 0.3500000       0
0
## 5        R2        29       24  Shipped-110Gy  0.5520833       1
1
## 6        R2        29       16    Shipped-0Gy  0.4166667       1
0
##   chilled   Treatment_age         Treatment
## 1       1  Ship-110Gy _22  4_Shipped-110Gy
## 2       1    Ship-0Gy_22      2_Shipped-0Gy
```

```
## 3       1 Unship-110Gy_22 3_Unshipped-110Gy
## 4       1   Unship-0Gy_22   1_Unshipped-0Gy
## 5       1  Ship-110Gy _22  4_Shipped-110Gy
## 6       1    Ship-0Gy_22      2_Shipped-0Gy

tabS2e_S3e$Pupal_age<- as.factor(tabS2e_S3e$Pupal_age)

###Figure S2e

tiff("Figure S2e.tiff", width = 7.5, height =4 , units = 'in', compression =
'lzw',res = 300)
boxplot(MSV~Treatments,data = tabS2e_S3e,
        ylab = expression(bold("Mean Spermathecal Value (22 and day old)")),
        xlab = expression(bold("Treatments")))
dev.off()

## png
##   2

### Figure S3e

tiff("Figure S3e.tiff", width = 4, height =4 , units = 'in', compression =
'lzw',res = 300)
boxplot(MSV~Pupal_age,data = tabS2e_S3e,
        ylab = expression(bold("Mean Spermathecal Value (22 and 29 day
old)")),
        xlab = expression(bold("Pupal age")))

dev.off()

## png
##   2

###significance
###Figure S2e
fmS2e <- lme(MSV ~ Treatment, random=~1|Replicate,, data = tabS2e_S3e)
summary(fmS2e)

## Linear mixed-effects model fit by REML
##   Data: tabS2e_S3e
##         AIC     BIC   logLik
##   -17.52898 -7.3957 14.76449
##
## Random effects:
##  Formula: ~1 | Replicate
##         (Intercept)  Residual
## StdDev:  0.02516712 0.1465828
##
## Fixed effects:  MSV ~ Treatment
##                                Value  Std.Error DF   t-value p-value
## (Intercept)                0.5076181 0.04529499 34 11.206938  0.0000
```

```
## Treatment2_Shipped-0Gy      0.0150198 0.06257954 34  0.240011  0.8118
## Treatment3_Unshipped-110Gy -0.0440265 0.06257954 34 -0.703529  0.4865
## Treatment4_Shipped-110Gy   -0.0569659 0.06257954 34 -0.910296  0.3691
##  Correlation:
##                            (Intr) T2_S-0 T3_U-1
## Treatment2_Shipped-0Gy     -0.689
## Treatment3_Unshipped-110Gy -0.689  0.501
## Treatment4_Shipped-110Gy   -0.689  0.501  0.501
##
## Standardized Within-Group Residuals:
##         Min          Q1         Med          Q3         Max
## -2.15760484 -0.79588319 -0.09250725  0.73163679  1.97888178
##
## Number of Observations: 44
## Number of Groups: 7
```

```
###Figure S3e
fmS3e <- lme(MSV ~ Pupal_age, random=~1|Replicate,, data = tabS2e_S3e)


summary(fmS3e)
```

```
## Linear mixed-effects model fit by REML
##   Data: tabS2e_S3e
##         AIC      BIC   logLik
##   -39.80438 -32.8537 23.90219
##
## Random effects:
##  Formula: ~1 | Replicate
##         (Intercept)  Residual
## StdDev:  0.02846677 0.1248484
##
## Fixed effects:  MSV ~ Pupal_age
##                   Value  Std.Error DF   t-value p-value
## (Intercept)  0.5669087 0.03039632 36 18.650569   0e+00
## Pupal_age29 -0.1465068 0.03811183 36 -3.844129   5e-04
##  Correlation:
##             (Intr)
## Pupal_age29 -0.688
##
## Standardized Within-Group Residuals:
##         Min          Q1         Med          Q3         Max
## -2.48552766 -0.74328239  0.02728295  0.81666426  1.62245025
##
## Number of Observations: 44
## Number of Groups: 7
```

```
###Differences among treatments- non-parametric test
kruskal.test(MSV~Treatment, tabS2e_S3e)
```

```
##
##  Kruskal-Wallis rank sum test
##
## data:  MSV by Treatment
## Kruskal-Wallis chi-squared = 2.0944, df = 3, p-value = 0.553
```
